# Field-induced non-linear magnetic responses of all-metal Jellium σ-aromats

**DOI:** 10.1038/s41467-026-74403-3

**Published:** 2026-06-29

**Authors:** Xinglan Deng, John A. Seed, Josef Tomeček, Adam Brookfield, Floriana Tuna, Benjamin L. L. Réant, Ashley J. Wooles, Nikolas Kaltsoyannis, Stephen T. Liddle

**Affiliations:** 1 Department of Chemistry and Centre for Radiochemistry Research, The University of Manchester, Manchester, UK; 2 Department of Chemistry and Photon Science Institute, The University of Manchester, Manchester, UK

**Keywords:** Chemical bonding, Chemical bonding

## Abstract

In recent times all-metal aromaticity has arisen, challenging classical views of aromaticity. All-metal aromaticity invokes metal-metal bonding, but this remains rare for the actinides. Recently, one-electron trithorium superatom clusters exhibiting exalted diamagnetism and hence open-shell Jellium aromaticity have been reported. Those clusters complement closed-shell two-electron congeners, but computational assessments advanced conflicting interpretations. Here, we report one- and two-electron trithorium clusters that all exhibit exalted diamagnetism, demonstrating open- and closed-shell Jellium aromaticities. We show that these Jellium aromats exhibit non-linear magnetic responses, so a foundational assumption of ring current calculations is not met, accounting for the experimental-computational disagreement. This work suggests that while π-aromaticity results from preorganized coherent wave functions, for σ-aromats there may be an electronic reorganization barrier to cohesive wave functions and Jellium aromatic ring currents. This work demonstrates the importance of caution when using ring current calculations to assign aromatic character if the experimental magnetic response is not understood.

## Abstract (web summary)

In recent times all-metal aromaticity has arisen, and for rare actinide examples generated disagreement between experimental and computational characterization methods. Here, the authors report one- and two-electron trithorium clusters that all exhibit exalted diamagnetism, demonstrating open- and closed-shell Jellium aromaticities and resolving the prior experimental-computation disagreement.

## Introduction

In recent times, all-metal aromaticity has emerged^1–12^, and this has challenged classical views of classical organic Huckel π-aromaticity which still lacks a universally-agreed definition^13^. All-metal aromaticity invokes metal-metal bonding, which remains rare for the actinides^7,11,14–21^. However, recently, one-electron trithorium superatom clusters exhibiting exalted diamagnetism were reported^11^, and thus those clusters experimentally evidence open-shell Jellium σ-aromaticity^22^. Those one-electron clusters complement a closed-shell two-electron congener which was also assigned σ-aromatic character^7^, but computational assessments advanced conflicting assessments of the bonding and σ-aromatic assignment^23–32^. Given that aromaticity cannot be directly experimentally measured, only its consequential effects are experimental observables, yet it is often probed using calculations that are not generally independently verifiable, it is important to resolve such a fundamental experimental-computational disagreement.

Here, we report expanded families of one- and two-electron trithorium clusters. Surprisingly, they all exhibit exalted diamagnetism^33,34^, and hence coherent wave functions resulting in open- and closed-shell Jellium σ-aromats, that is they are all superatoms. Through a detailed electronic structure and magnetism study and comparison to classical π-aromats we unravel key differences between σ- and π-aromats, ameliorating the previously irreconcilable experimental-computational discord. We thus highlight fundamental differences between these different types of aromaticity that further elaborates our understanding of the fundamental concept of aromaticity.

## Results

### Synthesis

**Fig. 1:**
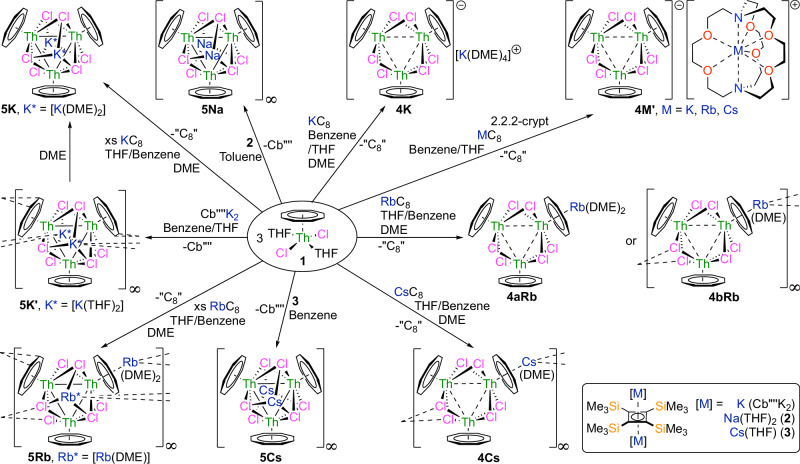
Synthesis of 4M (M = K, Rb, Cs), 4M’ (M = K, Rb, Cs), 5M (M = Na, K, Rb, Cs), and 5K’. The syntheses of **4M’** and **5K’** are reported in references^7^ and^11^, but are included here for completeness. Solid and dashed thorium-thorium bonds indicate three-centre two- and one-electron formulations, respectively.

The three-centre one-electron trithorium clusters [K(DME)_4_][{Th(η^8^-C_8_H_8_)(μ_3_-Cl)_2_}_3_] (**4K**), [{Th(η^8^-C_8_H_8_)(μ_3_-Cl)_2_}_3_{Rb(DME)_2_}] (**4aRb**), and [{Th(η^8^-C_8_H_8_)(μ_3_-Cl)_2_}_3_{Cs(DME)}]_∞_ (**4Cs**) were prepared by stoichiometric MC_8_ (M = K, Rb, Cs) reductions of [Th(η^8^-C_8_H_8_)(Cl)_2_(THF)_2_] (**1**)^35^ in 95:5 benzene:THF solvent mixtures, Fig. 1; the presence of THF is essential for reductions to proceed, but is present at the minimum level to avoid complete decomposition of products. After exposure to DME, all compounds were isolated in low but reproducible crystalline yields of ~7–10%. A polymeric variant of **4aRb**, [{Th(η^8^-C_8_H_8_)(μ_3_-Cl)_2_}_3_{Rb(DME)}]_∞_ (**4bRb**), was also isolated; its solid-state structure was determined, but all analysis was conducted on **4aRb**. Using increased equivalents of MC_8_ (M = K, Rb) afforded the three-centre two-electron trithorium clusters [{Th(η^8^-C_8_H_8_)(μ_3_-Cl)_2_}_3_{K(DME)_2_}_2_] (**5K**) and [{Th(η^8^-C_8_H_8_)(μ_3_-Cl)_2_}_3_{Rb(DME)}{Rb(DME)_2_}]_∞_ (**5Rb**), Fig. 1. By contrast, to access [{Th(η^8^-C_8_H_8_)(μ_3_-Cl)_2_}_3_(Na)_2_]_∞_ (**5Na**) and [{Th(η^8^-C_8_H_8_)(μ_3_-Cl)_2_}_3_(Cs)_2_]_∞_ (**5Cs**), reductions required the use of the soluble cyclobutadienyl reductants [{Na_2_(μ-η^4^:η^4^-C_4_(SiMe_3_)_4_)}(THF)_4_] (**2**) and [Cs_2_{μ-η^4^:η^4^-C_4_(SiMe_3_)_4_}(THF)_2_]_∞_ (**3**), Fig. 1, otherwise no products were isolable or **4Cs** was isolated, respectively. Complexes **5M** (M = Na, K, Rb, Cs) were isolated in crystalline yields of ~15–56%, with the best yields associated with the use of the soluble cyclobutadienyl reagents. All of **4M** and **5M** are dark blue, indicating that they contain low-valent thorium ions. We also isolated the two oxo-centred trithorium decomposition complexes [Na(DME)_3_]_2_[{Th(η^8^-C_8_H_8_)(μ-Cl)_2_}_3_(μ_3_-O)] (**6**) and [{Th(η^8^-C_8_H_8_)(μ-Cl)_2_}_3_(μ_3_-O){Cs(2.2.2-cryptand)}_2_] (**7**), which indirectly support the formulations of **5Na** and **5Cs**.

### Solid-state structures

**Fig. 2:**
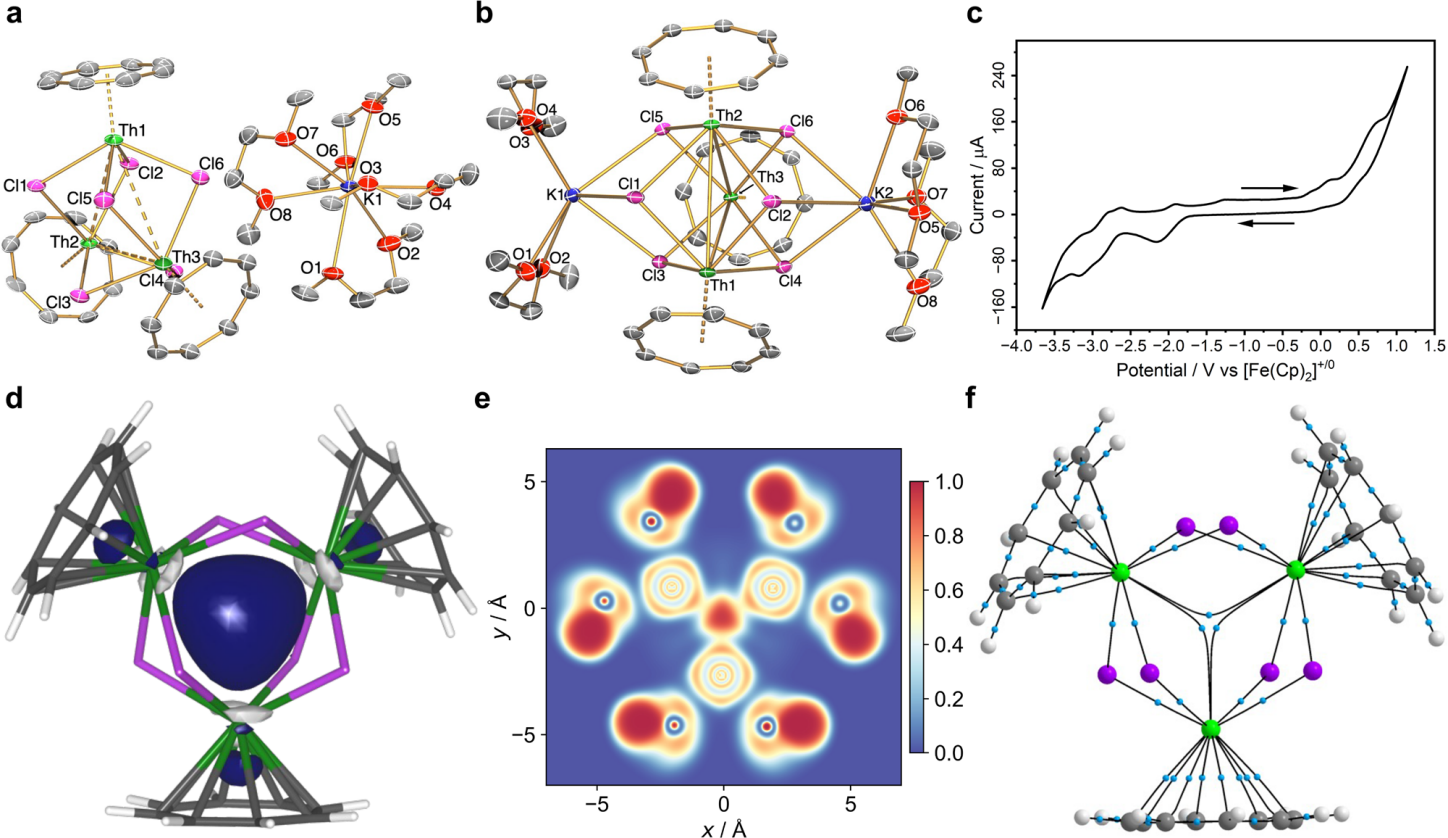
Structural, electrochemical, and computational bonding data for 4K, 5K, and 4K’. **a** solid-state structure of **4K** at 100 K, with 40% probability ellipsoids, selected atom labels, and disorder components omitted for clarity. **b** solid-state structure of **5K** at 150 K, with 40% probability ellipsoids, selected atom labels, and disordered components omitted for clarity. **c** cyclic voltammogram of **4K’** (5.0 mM in THF) recorded after an initial sweep to show all of the 0/–1, –1/–2, –2/–3, –3/–2, –2/–1, and –1/0 redox steps of [{Th(η^8^-C_8_H_8_)(μ_3_-Cl)_2_}_3_]^0^. The arrows indicate the initial and subsequent directions for sweeping the applied potential. Recorded with 50 mM [Bu^n^_4_N][BPh_4_] supporting electrolyte at a sweep rate of 200 mV s^–1^ and referenced to the ferrocene 0/+1 redox couple. **d** Highest occupied molecular orbital (389, –2.93 eV) of **5K** at the 0.05 isosurface level (PBE0 functional, SARC-TZVPP (Th) and def2-TZVPP (C, Cl, H, N, O) ZORA basis sets with D3-BJ dispersion corrections). **e** Electron localization function plot in the xy-plane (*z* = 0) of **5K** (PBE0 functional, SARC-TZVPP (Th) and def2-TZVPP (C, Cl, H, N, O) ZORA basis sets with D3-BJ dispersion corrections). **f** Quantum theory of atoms-in-molecules molecular graph of the bond paths in the core of **5K** (PBE0 functional, SARC-TZVPP (Th) and def2-TZVPP (C, Cl, H, N, O) ZORA basis sets with D3-BJ dispersion corrections). Bond critical points are plotted with an electron density threshold of 0.01 a.u. and are shown as light blue spheres. Ring critical points and the two {K(DME)_2_}^1+^ components are omitted for clarity.

The solid-state structures of **4M** (M = K, Rb, Cs) and **5M** (M = K, Rb) were determined by X-ray diffraction, showcasing the structural variety of the trithorium motif, and **4K** and **5K** are shown in Fig. 2a, b and Supplementary Figs. 3–11. Specifically, complex **4K** is a separated ion pair, **4aRb** has one of the C_8_H_8_ ligands η^8^-coordinated to a (DME)_2_Rb^+^ cation, in polymeric **4Cs** a (DME)Cs^+^ cation bridges trithorium units via η^8^-C_8_H_8_ and μ-Cl interactions, **5K** is a discrete contact ion triple, and polymeric **4Rb** exhibits η^8^-ligation of C_8_H_8_ rings to (DME)_2_Rb^+^ cations and (DME)Rb^+^ ions bridge between the μ_3_-Cl faces of neighbouring trithorium clusters. The Th–Th distances fall in two distinct sets of ranges, for the **4M** compounds the Th–Th distances span the range 4.0535(10)−4.1012(10) Å (mean of 4.076 Å), whereas those of **5M** are 3.9723(16)−3.9987(2) Å (mean of 3.990 Å). Hence, the three-centre two-electron Th–Th bonds are ~0.09 Å shorter than the one-electron congeners, consistent with stronger Th–Th bonding in the former compared to the latter^7,11,21^. All other solid-state metrical data are unexceptional.

### Electrochemical characterization

To confirm the reduced natures of these clusters we examined the electrochemistry of the separated ion pair cluster [K(2.2.2-cryptand)][{Th(η^8^-C_8_H_8_)(μ_3_-Cl)_2_}_3_] (**4K’**)^11^, chosen to interrogate the central trithorium unit free of coordinated group 1 metal effects (Supplementary Figs. 54–57). Starting at a potential of –0.84 V and sweeping to negative potentials, a reduction wave is found at *E*_PC(2)_ = –2.98 V, which is attributed to reduction of [{Th(η^8^-C_8_H_8_)(μ_3_-Cl)_2_}_3_]^1–^ ([**4**]^1–^) to [{Th(η^8^-C_8_H_8_)(μ_3_-Cl)_2_}_3_]^2–^ ([**5**]^2–^). Another reduction wave is observed at *E*_PC(3)_ = –3.18 V, which is tentatively attributed to the formation of [{Th(η^8^-C_8_H_8_)(μ_3_-Cl)_2_}_3_]^3–^. Sweeping back towards positive potentials reveals redox waves at *E*_PA(3)_ = –2.80 V, *E*_PA(2)_ = –2.63 V, and *E*_PA(1)_ = –1.90 V, attributed to sequential oxidations back to [{Th(η^8^-C_8_H_8_)(μ_3_-Cl)_2_}_3_]^0^. On sweeping back to negative potentials, E_PC(1)_, associated with reduction of [{Th(η^8^-C_8_H_8_)(μ_3_-Cl)_2_}_3_]^0^ to [**4**]^1–^, is then observed at –2.12 V. The cyclic voltammogram of the second cycle starting with [{Th(η^8^-C_8_H_8_)(μ_3_-Cl)_2_}_3_]^*n*^ (*n* = 0) and sweeping through *n* = –1, –2, –3, –2, –1, and 0 charge states is shown in Fig. 2c. For **5K**, similar redox potentials are obtained (*E*_PC(1)_ = –2.42; *E*_PC(2)_ = –3.16; *E*_PA(1)_ = –2.18; *E*_PA(2)_ = –2.76 V, Supplementary Figs. 58 and 59), though we could not observe the E_PC(3)/PA(3)_ couple and the E_PC/PA_ values are slightly shifted; this is as expected because in **5K** the K^+^ ions are not sequestered by 2.2.2-cryptand as they are in **4K’** and so may modulate the behaviour of the Th_3_ core under electrochemical conditions. These results are consistent with the one- and two-electron natures of **4M** and **5M** and suggest the formation of a trianion congener which is the subject of ongoing investigations. The observation of reduction waves in the potential range –2.12 to – 3.18 V is consistent with the reducing nature of these clusters^7,11^ and their superatom character that renders these clusters superalkali and superalkalines^36–38^.

### Quantum chemical analysis

The three-centre one-electron singly occupied molecular orbital (SOMO) electronic structure of the trithorium anion component of **4K** ([**4**]^1–^) has been computed elsewhere^11^, however we here computed the electronic structure of **5K**. Density Functional Theory (DFT) molecular orbital (MO), electron localization function (ELF), adaptive natural density partitioning, and electron density of delocalized bond methods all find that the highest occupied molecular orbital (HOMO; with Th Mulliken population analysis yielding 6d/5f/7p/7s contributions of 68/7/13/1%), Fig. 2d, is a doubly-occupied three-centre two-electron trithorium bond similar to that of [{Th(η^8^-C_8_H_8_)(μ_3_-Cl)_2_}_3_{K(THF)_2_}_2_]_∞_ (**5K’**)^7^, and the ELF graph demonstrates metal–metal bonding and an electron localization at the core of **5K**, Fig. 2e. Quantum theory of atoms-in-molecules analysis of **5K**, Fig. 2f, finds highly curved Th–Th bond paths, and three Th–Th bond critical points clustered towards the centre of the trithorium core that are similar to **5K’**^7^, but more curved and centrally-located than in [**4**]^1–^ consistent with their respective two- and one-electron occupancies^11^. The Th–Th bond critical points have *p*(r) = 0.022 a.u. and Laplacian ∇^2^*p*(*r*) = −9 × 10^−3^ a.u. values, which are consistent with **5K’** (cf. for [**4**]^1–^, *p*(*r*) = 0.014 a.u. ∇^2^*p*(*r*) = −7 × 10^−3^ a.u.)^7,11^. The delocalization indices for two-electron **5K** (0.246) and **5K’** (0.251) are similar, and expectedly larger than the corresponding value for one-electron [**4**]^1–^ (0.12). The Th–Th Mayer/Wiberg bond orders for **5K,** **5K’** and [**4**]^1–^ are 0.38/0.41, 0.45/0.45, and 0.27/0.27, respectively.

### Spectroscopic characterization

Raman spectroscopy of powdered **4M** and **5M** (Supplementary Figs. 78–104) is consistent with the presence of Th–Th bonding; the spectrum of **5K** is shown in Fig. 3a. Specifically, three principal bands are generally observed for each complex, at 48–73, 85–92, and 129–143 cm^–1^. These are confirmed by DFT frequency analysis to be bending and symmetric and asymmetric stretching modes, respectively, involving the trithorium core (Supplementary Fig. 171). Although multiple vibrations contribute to each mode, the stretching modes can be traced back to their parent *A* and *E* pure vibrational modes predicted from group theoretical considerations of a trimetallic motif.

**Fig. 3:**
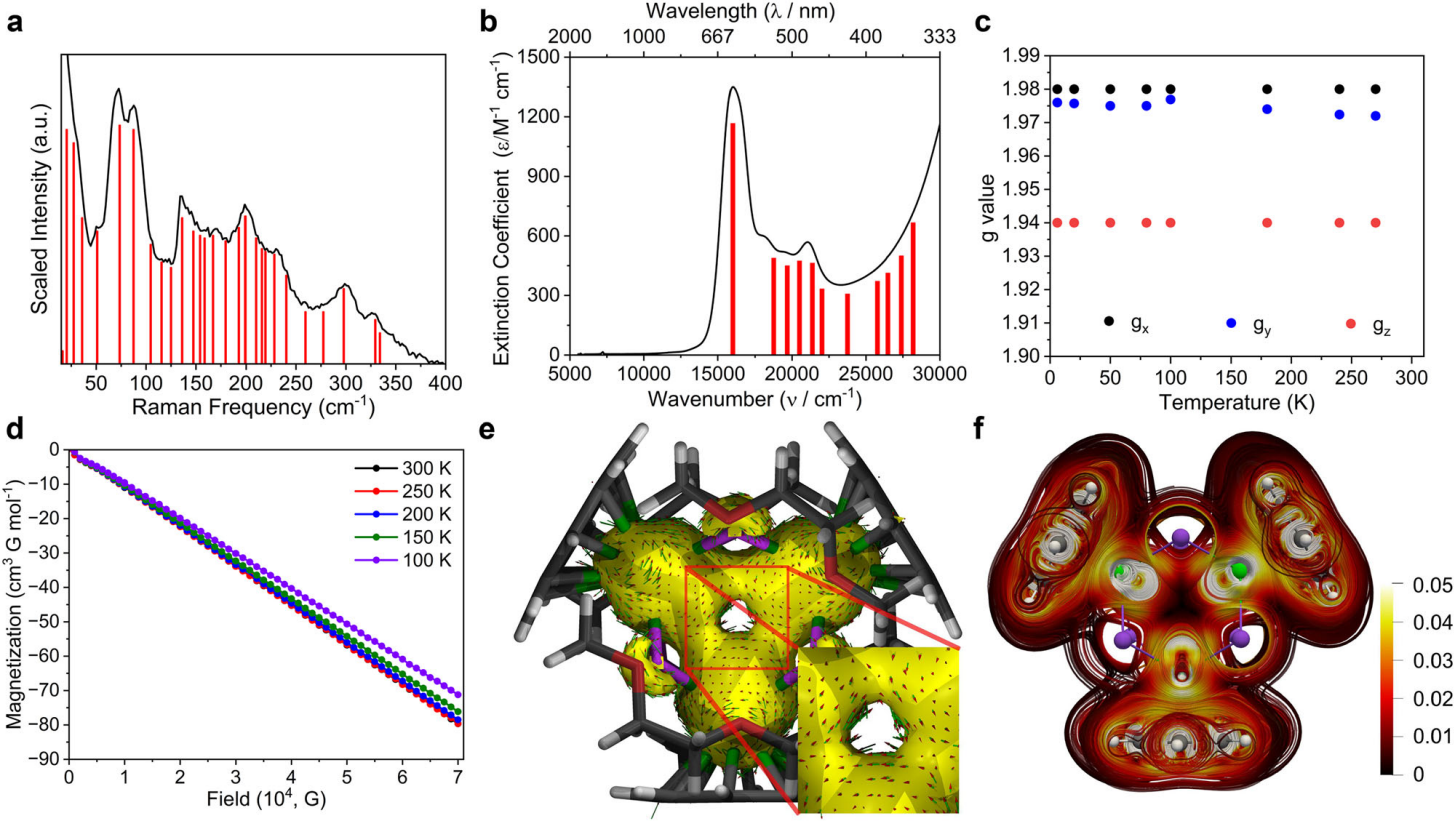
Spectroscopic, magnetic, and computational ring current analyses of 4K and 5K. **a** Raman spectrum (black line) of **5K** with DFT computed bands (red sticks, small Stuttgart-Dresden effective core potential and associated basis set for Th^40^, cc-pVTZ for C, O, and Cl, cc-pVDZ for H, and 6-311 G* for K^42^, together with D3-BJ dispersion corrections). **b** UV-vis-NIR spectrum (black line) of **5K** in DME with TD-DFT computed absorptions (red sticks, ω-B97X functional, SARC-TZVPP (Th) and def2-TZVPP (C, Cl, H, N, O) ZORA basis sets). **c** variable-temperature electron paramagnetic resonance data for **4K** showing the minor variance of *g*-values over the temperature range. **d** isothermal magnetization versus field data per complex of **5K** at 100, 150, 200, 250, and 300 K from 0–70000 Gauss showing the diamagnetic response. Error bars are omitted for clarity. **e** anisotropy of induced current density analysis showing the diatropic ring current in the core of **5K**. **f** induced ring current streamlines for **5K**.

UV-vis-NIR spectroscopy of DME solutions of **4M** and **5M** (Supplementary Figs. 105–110) consistently exhibit absorptions that confirm the presence of Th–Th bonding; that of **5K** is shown in Fig. 3b^7,11^. In particular, absorptions are principally observed at ~16k cm^–1^ (625 nm, *ε* = ~1300–4000 M^–1^ cm^–1^), ~17.5k cm^–1^ (571 nm, *ε* = ~600–1900 M^–1^ cm^–1^), and ~21k cm^–1^ (476 nm, *ε* = ~700–1300 M^–1^ cm^–1^). Time-dependent DFT reveals that these spectral features result from spin-, Laporte-, and parity-allowed transitions from the HOMO, which is predominantly Th 6d character, to vacant MOs of predominantly 5f composition and that are bonding combinations anticipated from trimetallic-cluster MO theory (Supplementary Fig. 173)^39^. These data correspond to inter-valence charge-transfer *Γ*_av_ values of 0.48–0.69, confirming that **4M** and **5M** all exhibit Robin-Day Class III fully valence-delocalized three-centre bonding interactions^40,41^.

We examined the *S* = 1/2 **4K,** **4aRb**, and **4Cs** with frozen solution and powder X-band electron paramagnetic resonance (EPR) spectroscopy (Supplementary Figs. 113–124), and for each find that across the temperature ranges examined (6 to 240–270 K) the complexes consistently exhibit near to axial EPR spectra with *g*_x_ = *g*_y_ (*g*_⊥_) > *g*_z_ (*g*_||_); the *g*-values for **4K** over the recorded temperature range are shown in Fig. 3c. The respective *g*-values are consistently ~1.98 and 1.94, and consistent with low-valent thorium ions^42,43^ and [M(2.2.2-cryptand)][{Th(η^8^-C_8_H_8_)(μ_3_-Cl)_2_}_3_] (**4M’**, M = K, Rb, Cs)^11^, and we note modest variation of the *g*-values over the temperature range, likely resulting from modulation of the Th–Th bonding since the EPR experiment is a direct reporter of excited states that are mixed into the SOMO by spin–orbit coupling under the influence of an applied magnetic field. As noted previously, the *g*_⊥_ > *g*_||_ nature of **4M** is opposite to mononuclear thorium(III) EPR data^11^. This is due to the three-centre one-electron bond of **4M** resulting from three 6d_z2_ orbitals arranged orthogonally to the three-fold principal rotation axis, whereas in mono-thorium(III) complexes the unpaired electron is in a 6d_z2_ type orbital aligned along the principal rotation axis^11,39^.

### Magnetic characterization and analysis

The magnetic susceptibilities of **4M** and **5M** were determined using variable-temperature superconducting quantum interference device (SQUID) magnetometry (Supplementary Figs. 125–138). All of *S* = 1/2 **4M** and *S* = 0 **5M** exhibit diamagnetic susceptibilities (*χ*_D_)^11^, with no evidence for net first-order Zeeman paramagnetic susceptibilities nor second-order Zeeman temperature independent paramagnetism. Isothermal (50–300 K) magnetization (*M*) vs field (*H*) measurements over the 0–70000 Gauss range uniformly reveal negative linear slopes (*R*^2^ = 0.9993–0.9999) with net *χ*_D_ of –797.22 × 10^–6^ to –1739.88 × 10^–6^ cm^3^ mol^–1^ per complex and –112.48 × 10^–6^ to –437.00 × 10^–6^ cm^3^ mol^–1^ per thorium ion at 300 K; see Fig. 3d for the data for **5K**. The magnetization values per thorium are ~5–19 times that expected, and the net *χ*_D_ responses are far larger than expected from cumulative Pascal’s constants (–497.88 × 10^–6^ to –716.48 × 10^–6^ cm^3^ mol^–1^)^44^. By contrast, as we previously reported the corresponding data for [{Th(η^8^-C_8_H_8_)(μ-Cl)_2_}_3_(μ_3_-O)(Cs)_2_(DME)_3_] is within 4.8% of the value predicted by Pascal’s cumulative constants^11^. For **4M** and **5M** this demonstrates exalted diamagnetism^11,33,34,36,37^ (*χ*_D_(expected) – *χ*_D_(found)) values of 268.44 × 10^–6^ to 1242.00 × 10^–6^ cm^3^ mol^–1^, that are ~51–250% increases on the expected *χ*_D_ (cf. cyclohexane, benzene, [14]annulene, [18]annulene, and [**4**]^1–^ are 0, 13.7 × 10^–6^, 267.4 × 10 ^− 6^, 640.1 × 10^–6^, and 688.2–1108.3 × 10^–6^ cm^3^ mol^–1^)^45^. Strikingly, these observations establish that regardless of whether these trithorium clusters are open-shell *S* = 1/2 or closed-shell *S* = 0, all of **4M** and **5M** exhibit exalted diamagnetism, and are therefore open- and closed-shell superatoms^22,37,38^, respectively, with superatom contributions to the exalted diamagnetism of ~34–71%. The observation that both *S* = 0 and 1/2 clusters are superatoms may at first seem counter-intuitive given the classical rules of aromaticity and antiaromaticity, but this is the result of superatomic electronic structures, where the clusters possess 1S^1^ and 1S^2^ magic numbers that sustain open- and closed-shell Jellium aromatic ring currents^22,36–38^.

Anisotropy of induced current density (AICD) calculations reveal a diatropic ring current in the core of **5K**, Fig. 3e and Supplementary Figs. 178 and 179, in line with AICD calculations on **5K’** and [**4**]^1–^^11,28^. Further, isochemical shielding surface (ICSS), an analogue of nucleus independent chemical shift (NICS), produces ICSS(0/1) = –NICS(0/1) values of 15.3/11.9 ppm (cf. 15.4/12.0 and 12.6/9.2 ppm for **5K** and [**4**]^1–^, respectively, Supplementary Fig. 180), which suggests strong aromaticity. The ICSS_*zz*_(0/1) values describe the *zz* component of the magnetic shielding tensor and hence a measure of the aromaticity, which for **5K** are 9.8/1.7 ppm (cf. 7.9/–0.1 and 6.9/–1.87 ppm for **5K’** and [**4**]^1–^, respectively). By excluding potassium cations and associated ligands to enable meaningful comparison, gauge-including magnetically induced current (GIMIC) integrations reveal computed ring current densities ( *j*(r)) of 2.16, 1.74, and 1.81 nA/T for **5K,** **5K’**, and [**4**]^1–^, respectively; the ring current streams of **5K** are illustrated in Fig. 3f. Thus, according to computational analyses diatropic ring currents exist in the trithorium clusters, but they are weak which would commonly be interpreted as signalling weak or no aromaticity. However, the computed magnetic susceptibilities of –734.02 × 10^−6^, –495.62 × 10^−6^, and –459.58 × 10^−6^ cm^3^ mol^−1^ for **5K,** **5K’**, and [**4**]^1–^, respectively, are consistent with the experimentally observed exalted diamagnetism that results from aromaticity.

## Discussion

The *j*(r) values of **5K,** **5K’**, and [**4**]^1–^ result from a complex interplay of paratropic and diatropic currents across these topologically diverse trithorium cores, which renders probing local *j*(r) effects a deterministic exercise since it is to some extent conditional on integration box selection. Different interpretations of the computed ring currents have generated vigorous and contradictory theoretical discussion of whether trithorium clusters are aromatic or not^7,11,23–32^, often focussing on the magnitude of the calculated magnetic response^23,25,27,29,30^. However, associating the magnitude of delocalization with aromaticity or antiaromaticity is problematic due to the absence of a first principles relationship between the amount of delocalization and the aromaticity of a system^46^. On the one hand various theoretical ring current calculations have been used to argue non-aromaticity^23,25,27,29,30^, on the other hand experimentally we find that all of these *S* = 0 and 1/2 clusters unequivocally experimentally exhibit exalted diamagnetism^11^, and hence are self-evidently open- and closed-shell Jellium aromats. It is essential to resolve such a computational-experimental discord, because ring current calculations underpin numerous discussions of aromaticity yet cannot be experimentally verified.

We therefore consider two observations. Firstly, the combined characterization data have consistently found three-centre two- or one-electron valence-delocalized multicentre bonding^7,11,21,24,26,28,31,32^, which when placed in an external field delocalizes around the Th_3_Cl_6_ core to generate superatom ring currents that produce exalted diamagnetism^47,48^. These static and dynamic scenarios can be effectively regarded as ground and excited states, that is there is no contradiction between the relatively localized three-centre bonding of the ground state and the free-electron-like distribution of delocalized electron density in the excited state. In the ground state, electron density in the clusters is distributed in discrete zones, and at low fields these zones do not interact strongly or coherently. As the external field increases, it induces an electronic reorganization that produces a coherent wavefunction that sustains global currents and, consequently, exalted diamagnetism. Thus, these are field-induced superatoms. By definition this is the case for susceptibility – a response to an external field – but it is germane to recognize it explicitly here.

**Fig. 4:**
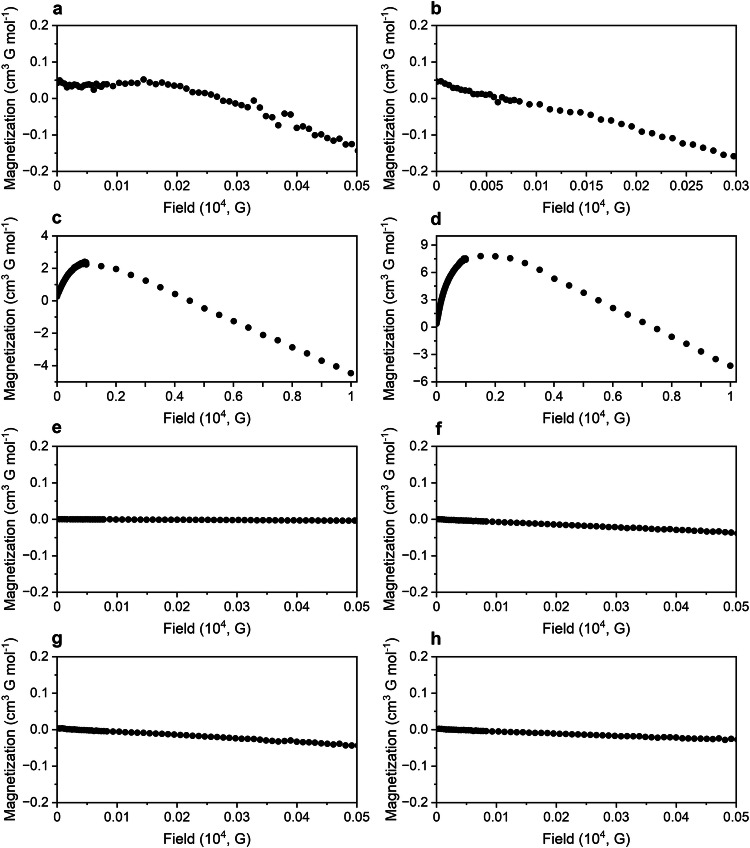
Magnetization vs field data for all-metal σ- and organic π-aromats at 300 K. **a** **4K**. **b** **5K**. **c** **4Cs**. **d** **5Cs**. **e** benzene. **f** naphthalene. **g** anthracene. **h** biphenyl.

Secondly, close inspection of the *M* vs *H* plots for **4M** and **5K** reveals that the negative gradient straight lines often do not initiate at the origin, and indeed often have the appearance of beginning at ~2700–7000 Gauss. Moreover, inspection of the data in the 0–10,000 Gauss range reveals that not only do the magnetization values for **4M** and **5M** generally cross zero at »0 Gauss, they often start with a weak paramagnetic response, and either initially increase before falling rapidly or plateau then fall; the variability of the magnetic responses is shown in Fig. 4a–d which illustrate the profiles for **4K,** **5K,** **4Cs**, and **5Cs**, respectively. Put another way, the magnetic susceptibilities of **4M** and **5M** are not linear and/or begin a substantial way from the origin. A non-linear and/or delayed zero crossing-point response implies a barrier effect, which we propose relates to inducing the electronic reorganization that is required from the relatively localized ground state SOMOs and HOMOs of **4M** and **5M** (Fig. 2d) to the field-induced excited states (Fig. 3e, f), and thus, delocalized ring currents that sustain exalted diamagnetism. Looking more widely, a similar effect can be recognized for one of the few examples of superatom exalted diamagnetism, namely Pt_13_ clusters^49^, suggesting that non-linear induction may be a more general phenomenon for σ-aromats.

To ascertain if the barrier effect is related to the σ-nature of the aromaticity, we probed the *M* vs *H* data for the π-aromats benzene, naphthalene, anthracene, and biphenyl (Supplementary Figs. 139–146), and found *χ*_D_ values of 5.52 × 10 ^− 5^, 9.35 × 10 ^− 5^, 13.14 × 10 ^− 5^, and 10.14 × 10 ^− 5^ cm^3^ mol^–1^, respectively, within 1.9% of literature values^50^. Within experimental error (~24 Gauss), that is due to remanence effects from the magnetometer physics of flux trapping and pinning, and in contrast to **4M** and **5M**, we find that the *χ*_D_ plots for the organic π-aromats essentially start at zero field and display virtually linear magnetization responses, Fig. 4e–h. These data suggest that π-aromats are intrinsically pre-organized to sustain ring currents and aromaticity because they are not localizable, that is the ground to excited state barrier is exceedingly small, but σ-aromats are relatively localized – even the valence-delocalized three-centre bonding of these trithorium clusters occupies a relatively localized zone – and thus need to be induced into fully delocalized forms to sustain global cluster ring currents and hence aromaticity. The implication for theoretical ring current calculations which are effectively conducted at zero field and assume a linear magnetic response thus emerges: in the absence of experimental magnetic susceptibility data, caution should be exercised when making aromaticity assignments if there is the possibility of non-linear or not immediate responses. In other words, ring current calculations may not always fully accommodate the required ground to excited state transition. This may be an essentially insignificant effect for classical π-aromats, but it appears to be significant for all-metal σ-aromats for which a non-linear response may be intrinsically common.

It is instructive to consider the findings here in the context of the lack of a universally-agreed definition of aromaticity. According to IUPAC^51^, the energy criterion is central, and the structural and magnetic criteria are important but not elementary^13^. However, the magnetic criterion is often regarded as being vitally important to making aromaticity assignments, resulting in discussions being based on ring current calculations, yet they are not experimentally independently verifiable. Our findings suggest that using the magnetic criterion as a hard binary criterion for assigning the aromatic character of molecules is likely not advisable when a key assumption of the calculations, that the magnetic susceptibility linearly extrapolates from zero applied field, is not known to be the case experimentally, and especially for all-metal aromats. This study thus underscores and clarifies the parallels and differences between classical organic and emergent all-metal aromaticity, and we hope that these findings will aid in the avoidance of determinism to more fully elaborate and understand aromaticity.

In summary, we have reported one- and two-electron trithorium clusters and demonstrated that they all exhibit exalted diamagnetism due to their superatomic natures, and hence they exhibit open- and closed-shell Jellium aromaticities. These clusters are paramagnetic at low external field, then with increasing field, they switch to exalted diamagnetism. Recognizing field-induced Jellium aromats with non-linear magnetic susceptibilities demonstrates that a foundational assumption of ring current calculations is not met for these clusters, accounting for the previously irreconcilable experimental-computational discord. This work suggests that while π-aromaticity results from preorganized coherent wave functions, for σ-aromats there may be an electronic reorganization barrier to establishing the cohesive wavefunction that sustains global Jellium aromatic ring currents. Thus, this work demonstrates the importance of exercising caution when using ring current calculations alone to assign the aromatic character of compounds if the experimental nature of the magnetic susceptibility response is not understood. We anticipate that our findings will inform the advancement of our fundamental understanding of aromaticity in the widest possible sense.

## Methods

### General experimental details

All manipulations were carried out using Schlenk techniques or an MBraun UniLab glovebox, under an atmosphere of dry dinitrogen or argon. Glassware was dried by overnight storage in a 150 °C oven followed by flame-drying under vacuum (1 × 10^–3^ mbar). Solvents were dried by passage through activated alumina towers and degassed prior to use. All solvents were stored over potassium mirrors except for DME and THF, which were stored over activated 4 Å sieves. Deuterated solvents were distilled from potassium, degassed by three freeze-pump-thaw cycles and stored under a dinitrogen atmosphere. Glassware was silylated prior to use. The compounds ThCl_4_(THF)_3.5_^52^, MC_8_ (M = K, Rb, Cs)^53^, C_8_H_8_K_2_^54^, [Th(η^8^-C_8_H_8_)_2_]^55^, [Th(η^8^-C_8_H_8_)(Cl)_2_(THF)_2_] (**1**)^35^, [Co{η^4^-C_4_(SiMe_3_)_4_}(η^5^-C_5_H_5_)]^56^, [Li_2_{μ-η^4^:η^4^-C_4_(SiMe_3_)_4_}(THF)_2_]^57^, and [C_4_(SiMe_3_)_4_]^58,59^ were prepared by the modified procedures described below. Sodium and caesium metal were purchased from Sigma Aldrich and used as received. NaBPh_4_ (Fluorochem) and [Bu_4_N][Cl] (Merck) were used without further purification. [Fe(η^5^-C_5_Me_5_)_2_] was purchased from Merck and was sublimed prior to use. 2.2.2-cryptand was purchased from Sigma Aldrich and dried under dynamic vacuum (1 × 10^–3^ mbar) at 80 °C for 24 h prior to use. [Bu^n^_4_N][BPh_4_] was prepared from the reaction of NaBPh_4_ and [Bu^n^_4_N][Cl] in H_2_O; the product was extracted with CH_2_Cl_2_ and a crude white powder was afforded after workup which was recrystallized three times from acetone and dried under vacuum (10^–3^ mbar) at 60 °C for 24 hours prior to use. All other reagents were purchased and used as received.

Single crystals were examined on a Rigaku XtalLAB Synergy-S diffractometer, equipped with a HyPix 6000HE photon-counting pixel array detector with mirror-monochromated Mo Kα (λ = 0.71073 Å) or Cu Kα radiation (λ  =  1.54184 Å). Intensities were integrated from a sphere of data recorded on narrow (0.5 or 1.0°) frames by ω rotation. Cell parameters were refined from the observed positions of all strong reflections in each data set. Gaussian grid face-indexed absorption corrections with a beam profile correction were applied. The structures were solved by dual methods using SHELXT^60^. All non-hydrogen atoms were refined by full-matrix least-squares on all unique *F*^2^ values, with anisotropic displacement parameters, with exceptions noted in the respective cif files. Except where noted, hydrogen atoms were refined with constrained geometries and riding thermal parameters; *U*_iso_(H) was set at 1.2 (1.5 for methyl groups) times *U*_eq_ of the parent atom. CrysAlisPro^61^ was used for control and integration, and SHELXL^62^ and Olex2^63^ were employed for structure refinement. ORTEP-3^64^ and POV-Ray^65^ were employed for molecular graphics.

^1^H, ^7^Li{^1^H}, ^13^C{^1^H}, and ^29^Si{^1^H} NMR spectra were recorded on a Bruker 400 spectrometer operating at 400, 155, 101, and 79 MHz, respectively or on a JEOL JNM-ECZ 400 MHz spectrometer operating at 399.78, 155.37, 100.52, and 79.42 MHz, respectively; chemical shifts are quoted in ppm and are relative to tetramethylsilane (^1^H, ^13^C, and ^29^Si), and LiCl (^7^Li). Samples were prepared in the glovebox and placed in J. Young PTFE 5 mm screw-topped borosilicate NMR tubes.

Electrochemical data were measured using an Ivium CompactStat.h20250 24-bit measurement and 20-bit generation Potentiostat/Galvanostat. Data were collected using the IviumSoft 4.1141 software operating in Basic Mode. Measurements were performed using a three-electrode cell in an inert atmosphere glovebox under an atmosphere of Ar with a glassy carbon working electrode (BASi MF-2012), a Ag/AgCl wire reference electrode (prepared by treating Ag wire with an aqueous FeCl_3_ solution), and a platinum wire auxiliary counter electrode (~7.5 cm length, BASi MW-1032) at ambient temperature (~298 K). The working electrode surface was polished prior to each set of experiments and was cleaned in between every scan taken. All data were collected in a positive-feedback IR compensation mode. Electrolyte solutions comprising of 50 mM [Bu^n^_4_N][BPh_4_] and 5.0 mM **4K'**^11^ or **5K** were prepared in dry THF. [Fe(η^5^-C_5_Me_5_)_2_] and was used as the internal reference standard, where *~*3 mg was added to electrolyte solutions at the end of the experiment collection. All potentials are reported versus the [Fe(η^5^-C_5_H_5_)_2_]^1+^/[Fe(η^5^-C_5_H_5_)_2_]^0^ (Fc^+^/Fc), using the conversion of [Fe(η^5^-C_5_Me_5_)_2_] *E*°‘ = –0.5 V versus Fc^+^/Fc for 50 mM [Bu^n^_4_N][BPh_4_] in THF^66^.

FTIR spectra were recorded on a Bruker Alpha spectrometer with a Pt-ATR module in a glovebox.

Raman spectra were recorded on a Horiba XploRA Plus Raman microscope using a 785 nm laser with a power of 1.5 mW. The number of scans and acquisition time were varied for each complex to inhibit sample decomposition. The power was adjusted using a power filter for each complex to inhibit sample decomposition.

UV-vis-NIR spectra were recorded on a Perkin-Elmer Lambda 1050 spectrometer, where data were collected in 1 cm path length cuvettes and were run versus the appropriate reference solvent.

Continuous-wave (c.w.) EPR spectra were measured using an X-band (9.4 GHz) Bruker EMX Plus spectrometer under 100 kHz magnetic field modulation and 2 G modulation amplitude at 293-5 K. Powdered samples were loaded into 3.9 mm (OD) quartz tubes in a glovebox under dinitrogen, and the tubes were flame sealed under vacuum using a MAPP-oxygen torch prior to experiments. Field corrections were applied to the raw data using Bruker strong pitch (*g * =  2.0028) as a reference. Spectra were simulated using the Easyspin 6.0 Software package^67^.

Variable-temperature magnetic moment data were recorded with various applied direct current (DC) external fields on a Quantum Design MPMS3 superconducting quantum interference device magnetometer using doubly recrystallized powdered samples. Measurements were performed in DC scan mode using 40 mm scan length and 6 s scan time. Samples were carefully checked for purity and data reproducibility between several independently prepared batches for each compound examined where great care was taken to ensure accurate weighing of materials. Samples were crushed with a mortar and pestle under an argon atmosphere and immobilized in an eicosane matrix within a borosilicate glass NMR tube to prevent sample reorientation during measurements. The tube was flame-sealed under dynamic vacuum (1 × 10^−3^ mbar) to a length of approximately 3 cm and mounted in the centre of a drinking straw, with the straw fixed to the end of an MPMS3 sample rod. Care was taken to ensure complete thermalization of the sample before each data point was measured by employing delays at each temperature point as well as a slow cooling rate (2.5 K/min from 300 to 100 K; 1 K/min from 100 to 50 K). The sample was held for 30 minutes before isothermal magnetization measurements to account for slow thermal equilibration of the sample. Diamagnetic corrections were applied using tabulated Pascal constants^44^ and measurements were corrected for the effect of the blank sample holders (flame-sealed Wilmad NMR tube and straw) and eicosane matrix. Isothermal magnetization measurements were conducted on calibrated blanks (sealed NMR tubes containing varying masses of eicosane, including no eicosane) to determine the eicosane contribution, which is defined per mg of eicosane used, in addition to the contribution from the NMR tube/straw, which is kept approximately constant from sample to sample. All samples were measured without the use of eicosane to reduce the diamagnetic contribution to the measurement^11^, reduce the likelihood of sample thermal decomposition when the eicosane is melted prior to sealing, and to remove an additional weighing error invoked when adding eicosane to the sample. However, for solely **5K**, we collected magnetometric data without eicosane and with eicosane (**5K-eico**), and find agreement within 6.45%, which is approximately within the measurement error of the instrument. The field was corrected for the remnant field of the superconducting magnet using a Pd reference sample under the same measurement conditions.

Elemental microanalyses were carried out by Mr Martin Jennings and Mrs Anne Davies at the Micro Analytical Laboratory, Department of Chemistry, University of Manchester.

### Modified Synthesis of MC_8_ (where M = K, Rb, Cs)

#### Generic method for the synthesis of the K analogue

A 250 mL round-bottomed Schlenk flask was charged with reagent grade (>99.9%) graphite (5.65 g, 470.4 mmol) and dried under dynamic vacuum (1 × 10 ^− 3^ mbar) at 100 °C for 4 h. In an argon-filled glovebox, freshly cut potassium metal (2.299 g, 58.8 mmol) was added; for Rb and Cs, the quantities were: Rb, 5.026 g, 58.8 mmol; Cs, 7.815 g, 58.8 mmol. The mixture was then heated under an argon atmosphere with a blowtorch whilst agitating causing the potassium metal to melt and intercalation to occur. Heating was continued until the mixture had completely changed colour from black to bronze. Where M = K, this is ~3 h. For M = Rb and Cs, the heating step is ~30–60 min. Once the reaction was complete, it was allowed to cool to room temperature. Yield: 7.87 g, 99%^53,68,69^.

### Modified synthesis of C_8_H_8_K_2_

A 500 mL round-bottomed Schlenk flask was charged with freshly cut potassium metal (3.12 g, 80 mmol) and suspended in THF (200 mL). At room temperature, freshly distilled 1,3,5,7-cyclooctatetraene (4.16 g, 40 mmol) was added slowly resulting in the gradual formation of a dark-brown solution. The mixture was stirred for 12 h before being filtered. Volatiles were removed *in vacuo* to yield a pale-brown, highly pyrophoric solid. Yield: 6.80 g, 93%. A single drop of ^1^H-THF was added to the NMR tube to aid solubility in the C_6_D_6_ NMR solvent. ^1^H NMR (C_6_D_6_/THF, 298 K): δ 6.47 (s, 8H, C_8_*H*_8_) ppm. ^13^C{^1^H} NMR (C_6_D_6_/THF, 298 K): δ 90.61 (s, *C*_8_H_8_) ppm. FTIR (ATR) ν/cm^−1^: 2987 (s), 2903 (w), 2869 (m), 1457 (w), 1425 (m), 1033 (s), 918 (w), 879 (s), 762 (w), 729 (w), 703 (s), 679 (m), 668 (s), 658 (s)^7,11^.

### Modified synthesis of ThCl_4_(THF)_3.5_

A 250 mL Young’s ampoule was charged with ThCl_4_(DME)_2_ (22.047 g, 39.80 mmol)^1^ and the solid dissolved in THF (~150 mL). Note—the volume of THF used is critical for success of the reaction: it must be a large excess. The solution was heated at 60 °C for 24 h, then cooled to room temperature and all volatiles were removed *in vacuo*. Reaction progress was monitored by ^1^H NMR spectroscopy of the solid, which is partially soluble in C_6_D_6_. The solid is initially a mixture of ThCl_4_(DME)_2_ and ThCl_4_(THF)_3.5_. Dissolution of the solid and heating in THF was repeated until the NMR spectrum of the pumped-down solid showed that all DME was removed. This usually requires 3–4 cycles. Once all DME was removed, the pale white solid was washed with hexane (2 × 30 mL), and dried *in vacuo* to give ThCl_4_(THF)_3.5_ as a white powder. Yield: 24.76 g, 99.4%. ^1^H NMR (C_6_D_6_, 298 K): δ 4.21 (s, 4H, -C*H*_2_O), 1.39 (s, 4H, -C*H*_2_CH_2_O) ppm. ^13^C{^1^H} NMR (C_6_D_6_, 298 K): δ 72.61 (s, -*C*H_2_), 25.48 (s, -*C*H_2_) ppm. FTIR (ATR) ν/cm^–1^: 2960 (m), 2896 (m), 1465 (m), 1450 (s), 1348 (s), 1302 (w), 1252 (m), 1192 (w), 1071 (w), 1039 (m), 998 (s), 956 (m), 913 (s), 844 (w), 821 (s), 675 (s), 573 (m). Where applicable, this data matches that reported in the literature^7,11,52^.

### Modified synthesis of [Th(η^8^-C_8_H_8_)_2_]

A 500 mL Young’s ampoule equipped with a PTFE stirrer bar was charged with ThCl_4_(THF)_3.5_ (7.675 g, 12.25 mmol) and C_8_H_8_K_2_ (4.47 g, 24.51 mmol). The mixture was cooled to –78 °C, then THF (100 mL) was added, and the suspension was allowed to warm slowly to room temperature. The resultant yellow reaction mixture was stirred for 48 hours before solvent was removed *in vacuo* to obtain a bright yellow powder (9.11 g). Using a 500 mL Schlenk flask containing toluene (100 mL), [Th(η^8^-C_8_H_8_)_2_] was extracted as a bright yellow microcrystalline powder via Soxhlet extraction for 5 days at 120 °C. Yield: 3.607 g, 67%. ^1^H NMR (D_8_-THF, 298 K): δ 6.58 (s, 16H, C_8_*H*_8_) ppm. ^13^C{^1^H} (D_8_-THF, 298 K): δ 108.06 (s, *C*_8_H_8_) ppm. FTIR (ATR) ν/cm^−1^: 2961 (w), 1317 (w), 1261 (s), 1088 (w), 1025 (m), 964 (w), 897 (m), 856 (w), 801 (s), 743 (m), 696 (s)^7,11,55^.

### Modified synthesis of [Th(η^8^-C_8_H_8_)(Cl)_2_(THF)_2_] (1)

A 500 mL Schlenk flask equipped with a PTFE stirrer bar was charged with ThCl_4_(THF)_3.5_ (5.06 g, 8.08 mmol) and [Th(η^8^-C_8_H_8_)_2_] (3.56 g, 8.08 mmol). THF (100 mL) was added at room temperature with stirring to form a yellow suspension, which was refluxed at 100 °C for four days. The resulting golden suspension was filtered hot through a Celite-packed coarse porosity frit, and the solvent was removed *in vacuo* from the resulting filtrate to yield **1** as an off-white solid which was used without further purification. Yield: 8.36 g, 94%. ^1^H NMR (D_8_-THF, 298 K): δ 6.63 (s, 8H, C_8_*H*_8_), 3.53 (s, 8H, -C*H*_2_), 1.68 (s, 8H, -C*H*_2_) ppm. ^13^C{^1^H} (D_8_-THF, 298 K): δ 102.59 (s, *C*_8_H_8_), 68.39 (s, -*C*H_2_), 26.53 (s, -*C*H_2_) ppm. FTIR (ATR) ν/cm^−1^: 2962 (s), 2900 (w), 1468 (w), 1455 (w), 1343 (w), 1209 (s), 1089 (m), 1008 (s), 904 (m), 852 (m), 797 (s), 717 (s), 669 (m)^7,11,35^.

### Modified synthesis of [Co{η^4^-C_4_(SiMe_3_)_4_}(η^5^-C_5_H_5_)]

A 250 mL round-bottom flask with condenser was charged with a PTFE stirrer bar, [Co(η^5^-C_5_H_5_)(CO)_2_] (5 g, 27.78 mmol), excess of bis(trimethylsilyl)acetylene (120 mL, 558 mmol), and then heated to 180 °C under a flow of N_2_ for seven days. The crude reaction mixture was then allowed to cool to 60 °C before volatiles, including residual Me_3_SiCCSiMe_3_, were removed by placing the mixture under dynamic vacuum for two hours to yield a black slurry. The crude product was extracted into hexane (100 mL) and further purified by passage through a silica column under inert gas, followed by crystallization at −30 °C from a saturated hexane solution, resulting in the isolation of [Co{η^4^-C_4_(SiMe_3_)_4_}(η^5^-C_5_H_5_)] as a yellow powder. Yield: 4.56 g, 44%. ^1^H NMR (C_6_D_6_, 298 K): δ 4.91 (s, 5H, C_5_*H*_5_), 0.24 (s, 36H, Si(C*H*_3_)_3_) ppm. ^13^C{^1^H} (C_6_D_6_, 298 K): δ 81.90 (s, *C*_4_), 79.60 (s, *C*_5_H_5_), 2.36 (s, Si(*C*H_3_)_3_) ppm. ^29^Si{^1^H} NMR (C_6_D_6_, 298 K): δ −7.68 ppm. ATR-IR ν/cm^−1^: 2953 (w), 2897 (w), 1540 (w), 1403 (w), 1327 (w), 1242 (s), 1174 (w), 1109 (w), 1000 (w), 892 (w), 829 (s), 803 (s), 752 (s), 686 (s), 651 (s), 636 (m), 618 (m), 461 (m), 424 (m)^56^.

### Modified synthesis of [Li_2_{μ-η^4^:η^4^-C_4_(SiMe_3_)_4_}(THF)_2_]

In an argon glovebox, a 500 mL Schlenk flask was charged with a glass-coated stirrer bar, [Co{η^4^-C_4_(SiMe_3_)_4_}(η^5^-C_5_H_5_)] (4.0 g, 9.44 mmol) and Li granules (1.0 g, 141.53 mmol). At 0 °C, THF (150 mL) was added with stirring resulting in the formation of an orange solution. *Note* – the addition of THF results in an exotherm so the cold bath must remain at 0 °C for the duration of the addition. After 20 minutes, the reaction mixture was allowed to warm to room temperature, resulting in a gradual colour change to dark green and then finally to red. The reaction mixture was stirred for 14 days with the mixture being carefully sonicated for 10 minutes each day. Volatiles were then removed *in vacuo* to yield a black powder. Soluble residues were extracted into pentane (150 mL) and filtered through a Celite-packed coarse porosity frit to obtain a clear, tan filtrate. Concentration of the filtrate and storage at –30 °C for four days yielded [Li_2_{μ-η^4^:η^4^-C_4_(SiMe_3_)_4_}(THF)_2_] as colourless crystals. Yield: 0.71 g, 15%. ^1^H NMR (C_6_D_6_, 298 K): 3.42 (s, 8H, -C*H*_2_), 1.14 (s, 8H, -C*H*_2_), 0.52 (s, 36H, Si(C*H*_3_)_3_) ppm. ^13^C{^1^H} (C_6_D_6_, 298 K): δ 103.68 (s, *C*_4_), 69.16 (s, -*C*H_2_), 24.95 (s, -*C*H_2_), 5.04 (s, Si(*C*H_3_)_3_) ppm. ^29^Si{^1^H} NMR (C_6_D_6_, 298 K): δ –24.14 ppm. ^7^Li{^1^H} NMR (C_6_D_6_, 298 K): δ –5.08 ppm. ATR-IR ν/cm^−1^: 2932 (w), 2878 (w), 2769 (w), 1594 (w), 1391 (w), 1306 (w), 1227 (w), 1084 (w), 1029 (w), 970 (w), 885 (w), 800 (s), 741 (m), 666 (m), 640 (s), 622 (m), 546 (m), 526 (m), 470 (m), 418 (m)^57–59^.

### Modified synthesis of [C_4_(SiMe_3_)_4_]

A 500 mL Rotaflow ampoule was charged with [Li_2_{μ-η^4^:η^4^-C_4_(SiMe_3_)_4_}(THF)_2_] (0.20 g, 0.4 mmol) and no stirrer bar was added. The ampoule was then submerged in liquid nitrogen (–196 °C) and an excess of 2,3-dibromobutane (1.73 g, 8.0 mmol) was carefully added via syringe under a N_2_ flow. *Important note* – this procedure must be carried out under dinitrogen, not argon, to avoid condensing argon which is an explosion hazard. Upon addition, the liquid nitrogen was removed and the reaction mixture was allowed to thaw under dynamic vacuum to remove the butene byproduct. Once the mixture had thawed, volatiles were removed *in vacuo* and soluble residues were extracted with hexane (20 mL). Removal of solvent *in vacuo* yielded a red solid which was sublimed under dynamic vacuum (~1 × 10 ^− 3^ mbar) at 80 °C onto a cold finger (water, 20 °C) to obtain red-orange crystals of [C_4_(SiMe_3_)_4_]. Yield: 0.058 g, 42%.^1^H NMR (C_6_D_6_, 298 K): δ 0.16 (s, 36H, Si(C*H*_3_)_3_) ppm. ^13^C{^1^H} (C_6_D_6_, 298 K): δ 172.40 (s, *C*_4_), 0.22 (s, Si(*C*H_3_)_3_) ppm. ^29^Si{^1^H} NMR (C_6_D_6_, 298 K): δ –17.04 ppm. ATR-IR ν/cm^−1^: 2953 (m), 2899 (w), 1539 (w), 1401 (w), 1245 (s), 896 (m), 813 (s), 746 (s), 688 (s), 635 (s), 618 (s), 530 (m), 462 (s)^58,59^.

### Synthesis of [{Na_2_(μ-η^4^:η^4^-C_4_(SiMe_3_)_4_)}(THF)_4_] (2)

Using a Young’s ampoule equipped with a glass-coated stirrer bar, sodium metal (41 mg, 1.80 mmol) was added to a stirring solution of [C_4_(SiMe_3_)_4_] (245 mg, 0.72 mmol) in THF (20 mL) at room temperature. The resultant red solution was stirred overnight and then filtered to obtain a dark orange solution. Volatiles were removed *in vacuo* to afford an orange-yellow solid, which was washed three times (3 × 5 mL) with cold (−30 °C) hexane. The resultant solid was dried *in vacuo* to afford a pale-yellow powder. Yellow-orange single crystals suitable for SC-XRD were grown from a saturated solution of **2** in hexane stored at − 30 °C for one week. Yield: 0.204 g, 42%. ^1^H NMR (C_6_D_6_, 298 K): δ 0.47 (s, 36H, Si(C*H*_3_)_3_) ppm. ^13^C{^1^H} (C_6_D_6_, 298 K): δ 106.70 (s, *C*_4_), 5.31 (s, Si(*C*H_3_)_3_) ppm. ^29^Si{^1^H} (C_6_D_6_, 298 K): δ − 31.05 ppm. Anal. Calc. for C_16_H_36_Na_2_Si_4_: C, 49.69; H, 9.38%. Found: C, 50.51; H, 9.23%. ATR-IR ν/cm^−1^: 2943 (m), 2881 (m), 1430 (w), 1231 (m), 1146 (m), 1085 (w), 1041 (m), 939 (w), 889 (w), 813 (s), 750 (m), 655 (m), 604 (w), 528 (w).

### Synthesis of [Cs_2_{μ-η^4^:η^4^-C_4_(SiMe_3_)_4_}(THF)_2_]_∞_ (3)

Using a Young’s ampoule equipped with a glass-coated stirrer bar, caesium metal (155 mg, 1.16 mmol) was added to a stirring solution of [C_4_(SiMe_3_)_4_] (100 mg, 0.29 mmol) in THF (20 mL) at room temperature. The resultant red solution was stirred overnight and then filtered to obtain a dark red solution. Volatiles were removed *in vacuo* to afford a dark red solid, which was washed three times (3 × 5 mL) with cold (−30 °C) hexane. The resultant solid was dried *in vacuo* to afford a rose-pink powder. Orange single crystals suitable for SC-XRD were grown by slow diffusion of hexane into a saturated solution of **3** in THF stored at −30 °C for one week. Yield: 0.045 g, 31%. ^1^H NMR (C_6_D_6_, 298 K): δ 0.44 (s, 36H, Si(C*H*_3_)_3_) ppm. ^13^C{^1^H} (C_6_D_6_, 298 K): δ 4.35 (s, Si(*C*H_3_)_3_) ppm. ^29^Si{^1^H} (C_6_D_6_, 298 K): δ –31.72. ATR-IR ν/cm^−1^: 3078 (w), 2932 (w), 2696 (w), 1576 (w), 1321 (w), 1245 (m), 1214 (m), 1118 (w), 1081 (w), 992 (s), 898 (s), 863 (m), 830 (m), 756 (m), 719 (m), 690 (m), 653 (m), 606 (m), 536 (w), 466 (w).

### Synthesis of [K(DME)_4_][{Th(η^8^-C_8_H_8_)(μ_3_-Cl)_2_}_3_] (4K)

#### Representative procedure for **4K**

A Young’s ampoule equipped with a PTFE stirrer bar was charged with a solid mixture of **1** (200 mg, 0.36 mmol) and KC_8_ (49 mg, 0.36 mmol). At room temperature, a 95:5 combination of benzene:THF (20 mL) was added, and the resultant suspension stirred for three days, during which time a dark blue solid precipitated. Volatiles were removed *in vacuo* and soluble residues were extracted into DME (~10 mL) to obtain a dark blue solution. Removal of volatiles *in vacuo* yielded **4K** as a dark blue powder. Yield: 0.02 g, 10.2% (by Th content). Single crystals of **4K** suitable for SC-XRD were obtained by slow diffusion of pentane into a saturated DME solution at −30 °C. Compound **4K** is completely insoluble in aliphatic and arene solvents and sparingly soluble with limited stability in D_8_-THF. Anal. Calc. for C_40_H_64_Cl_6_KO_8_Th_3_: C, 29.64; H, 3.98%. Found: C, 30.14; H, 3.76%. ^1^H NMR (D_8_-THF, 298 K): 3.43 (s, -C*H*_2_O, 8H), 3.27 (s, -C*H*_3_, 12H) ppm. ^13^C{^1^H} (D_8_-THF, 298 K): 72.9 (s, -C*H*_2_O), 59.1 (s, -*C*H_3_) ppm. The ^1^H and ^13^C{^1^H} signal for the Th-bound C_8_H_8_^-^ is not observed, presumably due to the low-valent nature of the thorium ions in **4K**. ATR-IR ν/cm^−1^: 3027 (w), 2923 (w), 2879 (w), 2810 (w), 1750 (w), 1632 (w), 1449 (w), 1361 (w), 1257(w), 1233 (w), 1188(m), 1075 (s), 1021 (m), 902 (m), 843 (m), 798 (m), 774 (w), 700 (s), 596 (w), 488 (w). Raman ν/cm^−1^ (785 nm): 33 (m), 67 (s), 92 (s), 132 (m), 194 (m), 227 (m), 286 (w), 359 (w), 481 (w), 564 (w), 704 (w), 743 (w). UV-vis-NIR (DME): *λ*_max_ (ν/cm^–1^; ε/M^−1^cm^−1^) = 473 (21110; 900), 549 (18204; 1035), 618 (16181; 2316), 1176 (8501; 81), 1210 (8259; 121), 1374 (7278; 140), 1423 (7023; 145), 1469 (6806; 79), 1707 (5856; 810), 1737 (5756; 1022), 1797 (5562; 418), 1879 (5321; 195), 1956 (5112; 218) nm.

### Synthesis of [{Th(η^8^-C_8_H_8_)(μ_3_-Cl)_2_}_3_{Rb(DME)_2_}] (4aRb)

The procedure is identical to that of **4K** except for the use of RbC_8_ as opposed to KC_8_. Yield: 0.03 g, 7.7% (by Th content). Single crystals of **4aRb** suitable for SC-XRD were obtained by slow diffusion of pentane into a saturated DME solution at −30 °C. Compound **4aRb** is insoluble in aliphatic, arene, and ethereal solvents precluding the acquisition of NMR spectroscopic data. A polymeric structure of **4aRb** was isolated, namely [{Th(η^8^-C_8_H_8_)(μ_3_-Cl)_2_}_3_{Rb(DME)}]_∞_ (**4bRb**), however all analysis was conducted on the monomeric analogue, **4aRb**. Anal. Calc. for C_32_H_44_Cl_6_RbO_4_Th_3_: C, 25.85; H, 2.98%. Found: C, 25.50; H, 3.14%. ATR-IR ν/cm ^− 1^: 3027 (w), 2923 (w), 2879 (w), 2810 (w), 1843 (w), 1755(w), 1627 (w), 1444 (w), 1361 (w), 1242 (w), 1183 (w), 1114 (w), 1080 (m), 1021 (m), 931 (w), 897 (m), 843 (m), 784 (w), 749 (w), 715 (s), 596 (w), 557 (w), 493 (m), 454 (w). Raman ν/cm ^− 1^ (785 nm): 60 (m), 85 (s), 136 (m), 221 (m), 240 (m), 286 (w), 364 (w), 742 (w), 835 (w), 892 (w), 1504 (w). UV-vis-NIR (DME): *λ*_max_ (ν/cm^–1^; ε/M ^− 1^cm ^− 1^) = 473 (21137; 1404), 552 (18115; 1768), 620 (16110; 3861); 1396 (7159; 16) nm.

### Synthesis of [{Th(η^8^-C_8_H_8_)(μ_3_-Cl)_2_}_3_{Cs(DME)}]_∞_ (4Cs)

The procedure is identical to that of **4K** except for the use of CsC_8_ as opposed to KC_8_. Yield: 0.011 g, 6.5% (by Th content). Single crystals of **4Cs** suitable for SC-XRD were obtained by slow diffusion of pentane into a saturated DME solution at −30 °C. Compound **4Cs** is insoluble in aliphatic, arene, and ethereal solvents precluding the acquisition of NMR spectroscopic data. Anal. Calc. for C_28_H_34_Cl_6_CsO_2_Th_3_: C, 26.14; H, 3.06%. Found: C, 26.03; H, 3.20%. ATR-IR ν/cm ^− 1^: 3027 (w), 2919 (w), 2874 (w), 2810 (w), 1853 (w), 1760 (w), 1617 (w), 1567 (w), 1449 (w), 1361 (w), 1321 (w), 1252 (w), 1193 (w), 1085 (m), 1021 (w), 892 (m), 853 (w), 793 (w), 700 (s), 596 (w), 567 (w), 488 (w). Raman ν/cm ^− 1^ (785 nm): 56 (s), 71 (s), 92 (s), 143 (w), 223 (s), 246 (m), 307 (w), 336 (w), 366 (w), 574 (w), 749 (m), 809 (w), 905 (w), 1102 (w), 1365 (s), 1504 (w), 1570 (w), 1993 (w), 2396 (w), 2766 (w). UV-vis-NIR (DME): *λ*_max_ (ν/cm^–1^; ε/M ^− 1^cm ^− 1^) = 470 (21272; 1249), 548 (18244; 1273); 622 (16072; 2511), 1167 (8566; 131), 1179 (8481; 145), 1212 (8248; 186), 1377 (7260; 216), 1422 (7030; 206), 1472 (6790; 125), 1706 (5859; 994); 1730 (5780; 1238), 1792 (5578; 513), 1857 (5385; 315), 1913 (5225; 257), 1969 (5104; 285), 2020 (4950; 201) nm.

### Synthesis of [{Th(η^8^-C_8_H_8_)(μ_3_-Cl)_2_}_3_(Na)_2_]_∞_ (5Na)

Using a 20 mL scintillation vial equipped with a glass-coated stirrer bar, a cold (−30 °C) solution of **2** (68 mg, 0.18 mmol) in toluene (10 mL) was added dropwise to a stirring suspension of **1** (200 mg, 0.36 mmol) in toluene (10 mL) at −30 °C. Upon addition, there was the gradual formation of an olive-green solution, with the concomitant precipitation of a dark blue solid. The reaction mixture was then stored at −30 °C for 24 h, before volatiles were removed *in vacuo* to obtain a dark blue powder, which was washed with cold (−30 °C) pentane (3 × 5 mL). Removal of volatiles *in vacuo* yielded **5Na** as a dark blue powder. Yield: 0.084 g, 54.9% (by Th content). Compound **5Na** is insoluble in aliphatic and arene solvents, and sparingly soluble with very limited stability in ethereal solvents, precluding the acquisition of a solid-state structure, as well as NMR spectroscopic data. Storage of **5Na** in DME for 24 hours results in the formation of [Na(DME)_3_]_2_[{Th(η^8^-C_8_H_8_)(μ-Cl)_2_(μ_3_-O)}_3_] (**6**). Anal. Calc. for C_24_H_24_Cl_6_Na_2_Th_3_·C_5_H_12_: C, 26.01; H, 2.71%. Found: C, 26.12; H, 2.83%. ATR-IR ν/cm ^− 1^: 3023 (w), 2954 (w), 1258 (w), 1247 (w), 1017 (w), 901 (m), 837 (m), 749 (w), 715 (s), 637 (w), 559 (w), 470 (w). Raman ν/cm ^− 1^ (785 nm): 48 (s), 137 (w), 170 (m), 199 (w), 252 (w), 299 (s), 324 (m), 530 (w), 568 (w), 738 (w), 798 (m), 981 (w), 1239 (w), 1358 (s), 1554 (w), 1779 (w). UV-vis-NIR (DME): *λ*_max_ (ν/cm^–1^; ε/M ^− 1^cm ^− 1^) = 406 (24618; 40), 587 (17041; 85), 1740 (5746; 2) nm.

### Synthesis of [{Th(η^8^-C_8_H_8_)(μ_3_-Cl)_2_}_3_{K(DME)_2_}_2_] (5K)

#### Representative procedure for **5K**

A Young’s ampoule equipped with a PTFE stirrer bar was charged with a solid mixture of **1** (200 mg, 0.36 mmol) and KC_8_ (194 mg, 1.44 mmol). At room temperature, a 95:5 combination of benzene:THF (20 mL) was added, and the resultant suspension was stirred for three days, during which time a dark blue solid precipitated. Volatiles were then removed *in vacuo* and soluble residues were extracted into DME (~10 mL) to obtain a dark blue solution. Removal of volatiles *in vacuo* yielded **5K** as a dark blue powder. Yield: 0.046 g, 23% (by Th content). Single crystals of **5K** suitable for SC-XRD were obtained by slow diffusion of pentane into a saturated DME solution at −30 °C. Compounds **5K** is insoluble in aliphatic and arene solvents and sparingly soluble with limited stability in D_8_-THF. Anal. Calc. for C_40_H_64_Cl_6_K_2_O_8_Th_3_: C, 28.94; H, 3.89%. Found: C, 28.68; H, 3.58%. ^1^H NMR (D_8_-THF, 298 K): 5.77 (s, -C_8_*H*_8_, 24H) 3.43 (s, -C*H*_2_O), 3.27 (s, -C*H*_3_) ppm. ^13^C{^1^H} (D_8_-THF, 298 K): 89.4 (s, *C*_8_H_8_), 72.82 (s, -C*H*_2_O), 57.97 (s, -*C*H_3_) ppm. The ^1^H and ^13^C{^1^H} signals for the Th-bound C_8_H_8_^-^ were assigned by comparison to a degraded sample of **5K**. ATR-IR ν/cm ^− 1^: 3017 (w), 2919 (w), 2879 (w), 2810 (w), 1833 (w), 1745 (w), 1454 (w), 1366 (w), 1252 (w), 1183 (w), 1080 (m), 1020 (m), 892 (m), 848 (m), 793 (w), 744 (w), 690 (s), 493 (w), 454 (w). Raman ν/cm ^− 1^ (785 nm): 51 (w), 73 (s), 87 (s), 135 (m), 200 (m), 298 (w), 330 (w), 568 (w), 742 (w), 797 (w), 1094 (w), 1365 (m), 2396 (w), 2761 (w). UV-vis-NIR (DME): *λ*_max_ (ν/cm^–1^; ε/M ^− 1^cm ^− 1^) = 473 (21137; 568), 545 (18335; 586), 615 (16252; 1325), 1386 (7215; 15) nm.

### Synthesis of [{Th(η^8^-C_8_H_8_)(μ_3_-Cl)_2_}_3_{Rb(DME)}{Rb(DME)_2_}]_∞_ (5Rb)

The procedure is identical to that of **5K** except for the use of RbC_8_ as opposed to KC_8_. Yield: 0.032 g, 15% (by Th content). Single crystals of **5Rb** (as a DME lattice solvate) suitable for XRD were obtained by slow diffusion of pentane into a saturated DME solution at −30 °C. Compound **5Rb** is insoluble in in aliphatic, arene, and ethereal solvents, precluding the acquisition of NMR spectroscopic data. Anal. Calc. for C_40_H_64_Cl_6_Rb_2_O_8_Th_3_: C, 27.41; H, 3.68%. Found: C, 27.28; H, 3.18%. ATR-IR ν/cm ^− 1^: 3022 (w), 2914 (w), 2869 (w), 2815 (w), 1843 (w), 1755 (w), 1622 (w), 1558 (w), 1449 (w), 1366 (w), 1316 (w), 1267 (w), 1188 (w), 1090 (m), 1026 (w), 907 (m), 843 (w), 789 (w), 715 (s), 700 (s), 606 (w), 577 (w), 503 (w). Raman ν/cm ^− 1^ (785 nm): 36 (m), 86 (s), 129 (m), 222 (s), 240 (s), 366 (m), 749 (m), 900 (w), 1504 (w). UV-vis-NIR (DME): *λ*_max_ (ν/cm^–1^; ε/M ^− 1^cm ^− 1^) = 473 (21123; 758), 556 (17998; 1199), 621 (16095; 2129), 1379 (7247; 34) nm.

### Synthesis of [{Th(η^8^-C_8_H_8_)(μ_3_-Cl)_2_}_3_(Cs_2_)]_∞_ (5Cs)

Using a 20 mL scintillation vial with a glass-coated stirrer bar, a cold (−30 °C) solution of **3** (171 mg, 0.36 mmol) in benzene (10 mL) was added dropwise to a stirring suspension of **1** (200 mg, 0.36 mmol) in benzene (10 mL) at −30 °C. Upon addition, a dark blue solid precipitated immediately. Stirring was continued for 30 minutes, before volatiles were removed *in vacuo* to obtain a dark blue powder, which was washed with cold (−30 °C) hexane (3 × 5 mL). Removal of volatiles *in vacuo* yielded **5Cs** as a dark blue powder. Yield: 0.1 g, 55.7% (by Th content). Compound **5Cs** is insoluble in aliphatic, arene, and ethereal solvents precluding the acquisition of a solid-state structure, as well as NMR spectroscopic data. Anal. Calc. for C_24_H_24_Cl_6_Cs_2_Th_3_: C, 23.02; H, 1.93%. Found: C, 22,79; H, 2.00%. ATR-IR ν/cm^−1^: 3023 (w), 2954 (w), 2895 (w), 1768 (w), 1540 (w), 1446 (w), 1402 (w), 1316 (w), 1257 (m), 1243 (w), 1211 (w), 1085 (w), 1018 (w), 891 (w), 830 (m), 798 (m), 754 (w), 714 (m), 642 (w), 618 (w), 610 (w). Raman ν/cm^−1^ (785 nm): 49 (w), 64 (w), 137 (w), 171 (w), 200 (w), 256 (w), 300 (w), 327 (w), 530 (w), 564 (w), 739 (w), 800 (w), 1369 (s), 1554 (m), 1862 (w).

### Synthesis of [Na(DME)_3_]_2_[{Th(η^8^-C_8_H_8_)(μ-Cl)_2_}_3_(μ_3_-O)] (6)

Using a 20 mL scintillation vial equipped with a glass-coated stirrer bar, a cold (−30 °C) solution of **2** (68 mg, 0.18 mmol) in toluene (10 mL) was added dropwise to a stirring suspension of **1** (200 mg, 0.36 mmol) in THF (10 mL) at −30 °C. The resultant green solution was stirred for two days, before volatiles were removed *in vacuo* to obtain a brown solid, which was washed with cold (−30 °C) pentane (3 × 5 mL), and the resultant solid dried *in vacuo* for two hours. Soluble residues were then extracted into DME (~10 mL) and storage of the brown solution at −30 °C for 24 hours resulted in the formation of **6** as brown crystals, presumably due to the degradation of **5Na** during synthesis. Yield: 0.054 g, 24.5% (by Th content). Compound **6** is insoluble in aliphatic and arene solvents, and sparingly soluble with limited stability in D_8_-THF. Anal. Calc. for C_48_H_84_Cl_6_Na_2_O_13_Th_3_·0.5(C_5_H_12_): C, 32.61; H, 4.88%. Found: C, 32.53; H, 4.39%. ^1^H NMR (D_8_-THF, 298 K): 6.42 (s, -C_8_*H*_8_, 24H) 3.42 (s, -C*H*_2_O, 6H), 3.27 (s, -C*H*_3_, 9H) ppm. ^13^C{^1^H} (D_8_-THF, 298 K): 102.4 (s, *C*_8_H_8_), 72.9 (s, -C*H*_2_O), 59.1 (s, -*C*H_3_) ppm. ATR-IR ν/cm^−1^: 2950 (m), 2895 (w), 1986 (w), 1856 (w), 1756 (w), 1622 (w), 1541 (w), 1400 (w), 1257 (m) 1241 (s), 1207 (s), 1094 (m), 1016 (m), 961 (m), 884 (s), 809 (s), 754 (s), 710 (s), 679 (s), 638 (s), 617 (m), 553 (w), 532 (m), 494 (m), 421 (s). Raman ν/cm^−1^ (785 nm): 223 (s), 385 (s), 720 (w), 751 (s).

### Synthesis of [{Th(η^8^-C_8_H_8_)(μ-Cl)_2_}_3_(μ_3_-O){Cs(2.2.2-cryptand)}_2_] (7)

Using a Young’s ampoule equipped with a PTFE stirrer bar, a solution of **1** (0.50 g, 0.90 mmol) and 2.2.2-cryptand (0.3388 g, 0.90 mmol) in THF (10 mL) was added to a stirring suspension of CsC_8_ (0.21 g, 0.90 mmol) in benzene (20 mL) at room temperature. The resultant mixture was stirred for 2 days resulting in the gradual formation of an orange-brown solution, with concomitant deposition of a black solid. Soluble residues were extracted into DME (~10 mL) and the resultant dark-brown solution was stored at −30 °C for one week resulted in the formation of dark-brown crystals. Compound **7** is completely insoluble in aliphatic and arene solvents. Yield: 0.014 g, 6.5% (by Th content). ^1^H NMR (D_8_-THF, 298 K): δ 6.58 (s, 8H, Cs-bound C_8_*H*_8_), 6.43 (s, 16H, C_8_*H*_8_), 3.58 (s, 24H, C*H*_2_-cryptand; this resonance could not be definitively integrated due to overlapping with peaks from the D_8_-THF solvent used for the NMR experiments), 3.54 (t, 24H, C*H*_2_-cryptand) 2.55 (t, 24H, C*H*_2_-cryptand) ppm. ^13^C{^1^H} (D_8_-THF, 298 K): δ 102.4 (s, *C*_8_H_8_), 102.3 (s, *C*_8_H_8_), 71.53 (s, *C*H_2_-cryptand), 68.7 (s, *C*H_2_-cryptand), 54.96 (s, *C*H_2_-cryptand) ppm. There are two C_8_H_8_ environments in solution, which implies the bonding motifs observed in the solid-state structure are retained in solution. FTIR (ATR) ν/cm^-1^: 3027 (w), 2863 (m), 2808 (w), 1472 (w), 1456 (w), 1443 (w), 1351 (m), 1295 (m), 1278 (w), 1258 (w), 1173 (w), 1105 (s), 1064 (s), 1021 (w), 978 (s), 940 (m), 902 (w), 847 (w), 830 (w), 816 (w), 748 (w), 702 (s), 595 (w), 577 (w), 567 (w), 550 (w), 492 (w), 448 (w), 414 (w).

### General DFT calculation details

DFT calculations on [{Th(η^8^-C_8_H_8_)(μ_3_-Cl)_2_}_3_{K(THF)_2_}_2_]_∞_ (**5K’**) and the [**4**]^1–^ component of **4M** were reported previously^7,11^. Geometry optimization of **5K** was performed with ORCA 6.0^70^. Relativistic effects were treated using the zeroth-order regular approximation (ZORA)^71^. The PBE0 hybrid exchange-correlation functional was used with Grimme’s D3 dispersion with Becke-Johnson damping parameters (D3-BJ)^72–75^. The SARC-TZVPP basis set was used for Th^76^, and def2-TZVPP for H, C, N, O and Cl^77^; the fitting basis SARC/J was employed to speed up the calculation of integrals using the RIJCOSX approximation^78,79^, and the NOTRAH and TightSCF convergence settings were used. Harmonic vibrational frequency analyses confirmed the absence of imaginary frequencies and hence the geometry-optimized structure of **5K** to be the true local true minimum geometry. Foster–Boys and Pipek–Mezey localization methods were also conducted with ORCA^70^.

The domain-based localized pair natural orbital variant of CCSD (DLPNO-CCSD) was employed to obtain the T_1_ diagnostic value for **5K**, as implemented in ORCA using the following settings^80,81^: SARC-TZVP basis set for Th, def2-SVP for ligand atoms with NormalPNO thresholds and the 2nd order Douglas–Kroll–Hess Hamiltonian^82,83^. The fitting basis sets for RIJCOSX were generated using the AutoAux function. The calculated value for **5K** of T_1_ was 0.0090, justifying the use of single-determinantal DFT approach. DLPNO-CCSD density calculations were not performed due to the high computational cost of obtaining coupled-cluster densities for systems of this size. In our previous work^28^ on a closely related 3c-2e trithorium cluster, we validated PBE0 against DLPNO-CCSD for a [Th_3_Cl_6_]^4+^ model system, yielding very good agreement in QTAIM Th–Th bonding results.

The UV-Vis-NIR spectrum of **5K** was calculated using ORCA 6.0 by employing time-dependent density functional theory (TD-DFT) to calculate 30 excited states. Multiple exchange-correlation functionals were tested (PBE0, CAM-B3LYP, ω-B97X, M06-L and M06-2X); the best match with experiment was obtained with ω-B97X.

The Raman spectrum of **5K** was calculated with Gaussian 16^84^, revision C.01, as described previously^11^, that is using the small Stuttgart–Dresden effective core potential (ECP)^85^ and associated ECP basis set for Th^86^, cc-pVTZ for C, O, and Cl, cc-pVDZ for H^87^, and 6-311 G* for K^88^, together with D3-BJ dispersion corrections. The geometry was reoptimized and the ultrafine integration grid was used. The RMSD between the ORCA and Gaussian geometries is 0.027 Å, and the Th–Th bond lengths are 4.014 and 4.017 Å, respectively. The Gaussian-reoptimized geometry was used only for the Raman and IR spectra calculations. The same settings (functional, basis set, ECP) were used when the Gaussian 16 single-point calculations were needed (calculations with NBO7^89^). In all calculations other than the Raman spectra, the geometry from ORCA was used.

Wavefunction analyses (electron localization function (ELF), bond orders, multicentre indices) were carried out with MultiWFN 3.8^90^ using molden input files from ORCA calculations (generated with the orca_2molden tool). QTAIM topology analysis was performed using AIMAll (version 19.02)^91^.

Adaptive natural density partitioning (AdNDP)^92^ and electron density of delocalized bonds^93^ (EDDB) analyses were performed as described in our recent publication using Gaussian 16 and NBO7 single-point calculations.

Anisotropy of the induced current density (AICD) analysis^94^ was performed using the AICD 3.0.4 software (Herges group) with the underlying Gaussian 16 calculation as described previously^11^.

Iso-chemical shielding surface (ICSS) calculations^95,96^ were performed by running NMR calculations in Gaussian 16 with input files generated by MultiWFN. The settings for Gaussian 16 calculations were the same as in our recent publication^11^.

The gauge-including magnetically induced currents (GIMIC 2.0) analyses^97–100^ were performed using the output files from Gaussian 16 (with the same settings as for AICD calculations). The current densities were calculated numerically with a cubic grid (33 Bohr long) positioned around the molecule with 75 × 75 × 75 grid points. The applied magnetic field was pointing down along the z-axis. In the ring current strength calculations, the integration planes start from the middle of the trithorium triangle and cut through the middle of the Th–Th bond perpendicular to the bond while the magnetic field was pointing down the *z*-axis. The integration plane (Supplementary Fig. 185) extends 18 Bohr above and below, 14 Bohr outside and 2.24 Bohr (i.e., a distance between the centre of the trithorium triangle and the Th–Th midpoint) inside of the Th–Th midpoint with the default grid spacing.

Ring current strengths were evaluated using default integration parameters, which are defined to be sufficiently tight for GIMIC calculations. The integration plane was chosen to fully encompass and extend well beyond the system. To verify numerical stability, we assessed the sensitivity of the results with respect to the integration grid by halving the grid spacing. This test yielded essentially identical ring current strengths (2.1577 vs 2.1580 nA/T for **5K** without {K(DME)_2_}^+^ moieties), confirming that the results are well converged with respect to the integration parameters.

The net isotropic magnetic susceptibilities were calculated using the NMR shielding and magnetic susceptibility module within Gaussian 16^101^.

Electrostatic potential maps were generated with Amsterdam Density Functional (ADF, version 2025.102)^102^ as single-point calculations. PBE0 functional was used with TZ2P basis set^103^. The quality numerical integration was increased to “Good” and scalar ZORA Hamiltonian was used. These settings were also used to generate HOMO with scalar or SOC ZORA Hamiltonian.

## Supplementary information

Supplementary Information

Transparent Peer Review file

## Source data

Source Data

## Data Availability

Crystallographic data for the structures reported in this Article have been deposited at the Cambridge Crystallographic Data Centre, under deposition numbers CCDC 2531345 (**2**), 2531346 (**3**), 2531347 (**4K**), 2531348 (**4aRb**), 2531349 (**4bRb**), 2531350 (**4Cs**), 2531351 (**5K**), 2531352 (**5Rb**) 2531353 (**6**), and 2531354 (**7**). Copies of the data can be obtained free of charge via https://www.ccdc.cam.ac.uk/structures/. All the other data supporting the findings of this study are available within the Article, or its Supplementary Information (Supplementary Figs. 1–185 and Tables 1–22), or from the corresponding authors on request. DFT geometry optimized cartesian coordinates source data are provided with this manuscript. The spectroscopic and magnetic source data in this study have been deposited in the Figshare database [10.48420/32324394]^104^. Source data are provided with this paper.
