# Supplementary material for: Field-induced non-linear magnetic responses of all-metal Jellium σ-aromats
Source: Nat Commun. 2026 Jun 29;17:8117. doi: 10.1038/s41467-026-74403-3 (PMC13458622; doi:10.1038/s41467-026-74403-3)
Supplement: Supplementary file 1 [file 41467_2026_74403_MOESM1_ESM.pdf]

– *Supplementary Information* –

Field-Induced Non-Linear Magnetic Responses of All-Metal Jellium σ-Aromats

Xinglan Deng,[1] John A. Seed,[1] Josef Tomeček,[1] Adam Brookfield,[2] Floriana Tuna,[2]
Benjamin L. L. Réant,[1] Ashley J. Wooles,[1] Nikolas Kaltsoyannis,[1]* Stephen T. Liddle[1]*

[1] Department of Chemistry and Centre for Radiochemistry Research, The University of Manchester, Oxford Road, Manchester, M13 9PL, UK.

[2] Department of Chemistry and Photon Science Institute, The University of Manchester, Oxford Road, Manchester, M13 9PL, UK.

*E-mail: nikolas.kaltsoyannis@manchester.ac.uk; steve.liddle@manchester.ac.uk

Supplementary Text

Salient NMR Details

Compounds **4M** (where M = Rb or Cs) and **5M** (where M = Na, Rb or Cs) are polymeric and insoluble in hydrocarbon, arene, and ethereal solvents precluding the acquisition of NMR spectroscopic data. In contrast, **4K** and **5K** are monomeric and hence ^{1}H and ^{13}C{^{1}H} solution NMR spectra could be acquired. Compounds **4K** and **5K** are insoluble in hydrocarbon and arene solvents, and sparingly soluble with limited stability in D_8-THF. Compounds **4K** and **5K** are more soluble and stable in dimethoxyethane (DME), but not sufficiently so to be rendered practicable, hence D_8-THF was the most appropriate compromise to obtain reliable NMR data. Within the ^{1}H spectrum of **4K**, which possesses an open-shell electronic configuration, several resonances can be visualized in the region anticipated for the C_8H_8 H-atoms (6.0-7.0 ppm). However, no carbon C_8H_8 resonances could be located for **4K** in the ^{13}C{^{1}H} NMR spectra so these resonances are assigned as diamagnetic impurities in agreement to what we have previously observed when measuring NMR spectroscopic data of open-shell trithorium nanoclusters.[1] Overall, the unassignable nature of the C_8H_8 resonances for **4K** reflects the low valent nature of the thorium ions within, and open-shell character of, **4K**, and is in common with other thorium(III)-C_8H_8, as well as titanium(III)-C_8H_8, complexes, in addition to d^1 $S = ½$ systems more generally.[2-9] However, for **5K**, which possesses a closed-shell electronic configuration, the resonances for the C_8H_8 H-atoms can be assigned in both ^{1}H and ^{13}C{^{1}H} NMR spectra. To confirm the assignment, **5K** was measured by multinuclear NMR spectroscopy and the sample then intentionally degraded and remeasured. Upon degradation, there is the loss of the characteristic blue colour of the solution as well as the disappearance of the assigned C_8H_8 resonance for **5K** in both the ^{1}H and ^{13}C{^{1}H} NMR spectra; resonances related to [Th(η^8-C_8H_8)$_2$] and free 1,3,5,7-cyclooctatetraene, which are common decomposition products, are then observed. Whilst the chemical shift value assigned to the C_8H_8 resonance for **5K** (5.77 ppm) is very similar to that reported for free 1,3,5,7-cyclooctatetraene (5.72 ppm in D_8-THF), there is no accompanying resonance for free 1,3,5,7-cyclooctatetraene in the ^{13}C{^{1}H} (~133 ppm) as would be expected. The ^{13}C{^{1}H} chemical shift value of **5K** is very similar to that of K_2COT (89.4 for **5K** vs. 90.6 for K_2COT) and reflects the dianionic nature of the coordinated C_8H_8 moieties. Both of these resonances are absent when **5K** decomposes.

Salient Raman Details

The Raman spectra have common features which can be assigned as follows: (i) bands at ~250 cm^{-1} correspond to symmetric COT-metal stretching and metal-COT tilting modes; (ii) bands at ~380 cm^{-1} correspond to COT CCC in-plane bending modes; (iii) bands at ~750 cm^{-1} are attributed to COT C-C ring breathing modes; (iv) bands at ~1500 cm^{-1} correspond to CC stretching modes; (v) features at ~3000 cm^{-1} correspond to symmetric C-H stretching modes. On occasion, a large broad feature is observed at ~1400 cm^{-1} when using the 785 nm excitation, which results from strong and broad

photoluminescence of the glass microscope slide used for the measurement.[10] Photoluminescence has a much higher probability of occurring than Raman scattering, which is a two-photon process, and hence can overwhelm any Raman signals within that region. For **4M** and **5M**, the signals of interest are below 1000 cm^{-1} meaning any photoluminescence of the glass microscope does not affect the analysis as this broad feature tails off >1000 cm^{-1}. Using lower excitation wavelength (638 or 532 nm) shifts the photoluminescence energy, however the higher energies of these excitation wavelengths result in higher incidences of sample decomposition.

Computational methodology justification

Despite the lack of universal benchmarks for actinide systems, hybrid functionals such as PBE0 have been extensively validated in relevant contexts. Our previous work has benchmarked PBE0 against CCSD(T) calculations,[11] as well as against experimental observables in f-element NMR studies,[12] demonstrating its reliability for describing the electronic structure and magnetic properties of actinide compounds. Furthermore, we have shown the suitability of PBE0 for the 3c-2e trithorium cluster by comparison with DLPNO-CCSD results.[1]

Spin-orbit coupling (SOC) was not included in the present calculations. While SOC can play an important role in actinide systems, its inclusion is incompatible with the presented computational workflow (i.e., the electronic-structure analysis and magnetic-field-including methods). Moreover, our previous study[1] demonstrated that scalar relativistic approaches provide a quantitatively similar description of Th-Th bonding within the QTAIM framework when compared to ZORA+SOC calculations (from ADF). We therefore expect that inclusion of SOC would not significantly affect the key conclusions of this work.

This is further supported by comparison of frontier molecular orbitals obtained from scalar ZORA and ZORA+SOC single-point calculations (ADF: PBE0/TZ2P), which show virtually identical spatial distributions (as shown in Supplementary Figure 183).

Salient TD-DFT Data

For TD-DFT calculations, multiple exchange-correlation functionals were tested (PBE0, CAM-B3LYP, ω-B97X, M06-L and M06-2X) using the PBE0 geometry; the best match with experiment was obtained with ω-B97X. The lowest-energy transitions are at 626 and 624 nm, matching the experimental peak at 625 nm. The second peak in the experimental spectrum is at 475 nm and corresponds to multiple electronic transitions, with calculated excitation wavelengths of 504, 489, 488, 465, 464, 424, and 421 nm. The acceptor natural transition orbitals (NTOs) for the corresponding transitions are shown in Supplementary Figure 173 (all electronic transitions for donor–acceptor pairs start from a HOMO-like donor). Mulliken population analyses of the selected acceptor NTOs indicate a dominant 5f-character (Supplementary Table 20).

AdNDP, $EDDB_F$, MCI, and ELF Details

To study the electron delocalization within **5K**, we utilised adaptive natural density partitioning (AdNDP), electron density of delocalized bonds ($EDDB_F$), and electron localization function (ELF) analyses for consistency with our previous works[1,13,14] and these are shown in Supplementary Figures 175-176. The 3-center MO is found with AdNDP and has an occupation number 1.77 (*cf.*, 1.80 for **5K'**) and this MO is also maintained on performing Foster-Boys and Pipek-Mezey localizations. The $EDDB_F$ search on the Th_3 fragment yields 1.2 electrons (*cf.*, 1.3 for **5K'**). Multicentre index (MCI) of the Th_3 fragment in **5K** is 0.191 (*cf.*, 0.267 for **5K'**). The value of ELF at the center of Th_3 core is 0.946 (*cf.*, 0.951 for **5K'**). Overall, these results indicate that the 3c-2e bonding in **5K** is analogous to **5K'**.

GIMIC commentary

The modulus of the induced current density *J* from the gauge-including magnetically induced currents (GIMIC) calculations is plotted in Supplementary Figure 181, showing regions with diatropic (red) and paratropic (blue) currents, as in our contribution on **[4]**$^{1-}$.[1] The induced currents starting from the middle of the Th_3 core or from one Th atom are shown in Supplementary Figure 182, indicating that there are sustained currents throughout the unit comprising the $[Th_3Cl_6]^{4+}$ and the COT ligands.

The results of ring current integrations with GIMIC are presented in Supplementary Table 22. The effect of DME solvent molecules at **5K** on ring current strength is negligible. Further integrations, focusing, e.g., on the $[Th_3]^{10+}$ core itself, the $[Th_3Cl_6]^{4+}$ fragment, or isolated Cl and COT ligands, were also tested. For the $[Th_3]^{10+}$ and $[Th_3Cl_6]^{4+}$ fragments, both diatropic and paratropic currents are evident; the latter effect is stronger. This is consistent with the cut plot of the *J* modulus, which shows diatropic regions in the Th_3 core, but paratropic regions near the Th atoms appear larger. For individual fragments, the net positive ring current strength appears to be due to the presence of K atoms, which is why we quoted the ring current strength for a fragment without K^+ ions and DME solvent molecules.

Electrostatic potential map of 5K

The electrostatic potential (ESP) was mapped onto the van der Waals isosurface (ρ = 0.001 a.u.) in Supplementary Figure 184, showing the negative regions around COT and Cl^- ligands. This plot shows that the negative electrostatic potential surrounding the trithorium cluster **5K** arises from both the chloride ligands and the COT ligand framework. The ESP plot was also mapped at the electron density isovalue of 0.01 a.u., which allows us to visualise the positive regions of electrostatic potential around the stabilising K^+ ions.

Crystallographic CIF Level A and B Alerts and Explanations

3

Alert level A

PLAT971_ALERT_2_A Check Calcd Resid. Dens. 0.94Ang From Cs3 3.80 eA-3

Author Response: Residual density is close to a heavy atom (Cs) and present despite applying absorption corrections. It is not chemically significant.

Alert level B

PLAT342_ALERT_3_B Low Bond Precision on C-C Bonds .... 0.02773 Ang.

Author Response: low precision is due to poor quality data.

PLAT971_ALERT_2_B Check Calcd Resid. Dens. 1.15Ang From Cs1 3.00 eA-3

Author Response: Residual density is close to a heavy atom (Cs) and present despite applying absorption corrections. It is not chemically significant.

PLAT971_ALERT_2_B Check Calcd Resid. Dens. 0.88Ang From Cs2 2.94 eA-3

Author Response: Residual density is close to a heavy atom (Cs) and present despite applying absorption corrections. It is not chemically significant.

4K

Alert level B

PLAT971_ALERT_2_B Check Calcd Resid. Dens. 0.75Ang From Th2 2.51 eA-3

Author Response: Residual density is close to a heavy atom and present despite applying absorption corrections. It is not chemically significant.

PLAT973_ALERT_2_B Check Calcd Positive Resid. Density on Th2 1.60 eA-3

Author Response: Residual density is close to a heavy atom and present despite applying absorption corrections. It is not chemically significant.

4bRb

Alert level B

PLAT971_ALERT_2_B Check Calcd Resid. Dens. 0.83Ang From Th2 3.46 eA-3

Author Response: Residual density is close to a heavy atom and present despite applying absorption corrections. It is not chemically significant.

PLAT972_ALERT_2_B Check Calcd Resid. Dens. 0.75Ang From Th1 -2.68 eA-3

Author Response: Residual density is close to a heavy atom and present despite applying absorption corrections. It is not chemically significant.

4Cs

Alert level B

PLAT971_ALERT_2_B Check Calcd Resid. Dens. 0.88Ang From Th1 3.38 eA-3

Author Response: Residual density is close to a heavy atom and present despite applying absorption corrections. It is not chemically significant.

PLAT971_ALERT_2_B Check Calcd Resid. Dens. 1.00Ang From Th2 3.31 eA-3

Author Response: Residual density is close to a heavy atom and present despite applying absorption corrections. It is not chemically significant.

PLAT971_ALERT_2_B Check Calcd Resid. Dens. 0.88Ang From Th2 3.22 eA-3

Author Response: Residual density is close to a heavy atom and present despite applying absorption corrections. It is not chemically significant.

PLAT971_ALERT_2_B Check Calcd Resid. Dens. 0.94Ang From Th2 2.86 eA-3

Author Response: Residual density is close to a heavy atom and present despite applying absorption corrections. It is not chemically significant.

PLAT971_ALERT_2_B Check Calcd Resid. Dens. 0.89Ang From Th1 2.73 eA-3

Author Response: Residual density is close to a heavy atom and present despite applying absorption corrections. It is not chemically significant.

PLAT971_ALERT_2_B Check Calcd Resid. Dens. 0.98Ang From Th2 2.71 eA-3

Author Response: Residual density is close to a heavy atom and present despite applying absorption corrections. It is not chemically significant.

PLAT973_ALERT_2_B Check Calcd Positive Resid. Density on Th2 1.96 eA-3

Author Response: Residual density is close to a heavy atom and present despite applying absorption corrections. It is not chemically significant.

PLAT973_ALERT_2_B Check Calcd Positive Resid. Density on Th1 1.81 eA-3

Author Response: Residual density is close to a heavy atom and present despite applying absorption corrections. It is not chemically significant.

5Rb

Alert level A

PLAT971_ALERT_2_A Check Calcd Resid. Dens. 0.81Ang From Th3 5.75 eA-3

Author Response: Residual density is close to a heavy atom and present despite applying absorption corrections. It is not chemically significant.

PLAT971_ALERT_2_A Check Calcd Resid. Dens. 0.85Ang From Th2 5.55 eA-3
Author Response: Residual density is close to a heavy atom and present despite applying absorption corrections. It is not chemically significant.

PLAT971_ALERT_2_A Check Calcd Resid. Dens. 0.81Ang From Th1 5.45 eA-3
Author Response: Residual density is close to a heavy atom and present despite applying absorption corrections. It is not chemically significant.

PLAT971_ALERT_2_A Check Calcd Resid. Dens. 0.92Ang From Th3 4.82 eA-3
Author Response: Residual density is close to a heavy atom and present despite applying absorption corrections. It is not chemically significant.

PLAT971_ALERT_2_A Check Calcd Resid. Dens. 0.90Ang From Th2 4.53 eA-3
Author Response: Residual density is close to a heavy atom and present despite applying absorption corrections. It is not chemically significant.

PLAT971_ALERT_2_A Check Calcd Resid. Dens. 0.96Ang From Th1 4.36 eA-3
Author Response: Residual density is close to a heavy atom and present despite applying absorption corrections. It is not chemically significant.

PLAT971_ALERT_2_A Check Calcd Resid. Dens. 0.92Ang From Th3 4.32 eA-3
Author Response: Residual density is close to a heavy atom and present despite applying absorption corrections. It is not chemically significant.

PLAT971_ALERT_2_A Check Calcd Resid. Dens. 0.90Ang From Th1 4.08 eA-3
Author Response: Residual density is close to a heavy atom and present despite applying absorption corrections. It is not chemically significant.

PLAT971_ALERT_2_A Check Calcd Resid. Dens. 1.00Ang From Th3 4.00 eA-3
Author Response: Residual density is close to a heavy atom and present despite applying absorption corrections. It is not chemically significant.

PLAT971_ALERT_2_A Check Calcd Resid. Dens. 0.85Ang From Th2 3.94 eA-3
Author Response: Residual density is close to a heavy atom and present despite applying absorption corrections. It is not chemically significant.

PLAT971_ALERT_2_A Check Calcd Resid. Dens. 0.92Ang From Th1 3.93 eA-3
Author Response: Residual density is close to a heavy atom and present despite applying absorption corrections. It is not chemically significant.

PLAT971_ALERT_2_A Check Calcd Resid. Dens. 0.91Ang From Th2 3.74 eA-3
Author Response: Residual density is close to a heavy atom and present despite applying absorption corrections. It is not chemically significant.

Alert level B
PLAT342_ALERT_3_B Low Bond Precision on C-C Bonds .... 0.03857 Ang.
Author Response: Low precision is due to high Rint values and relatively high amounts of absorption problems.

PLAT972_ALERT_2_B Check Calcd Resid. Dens. 0.94Ang From Th1 -3.15 eA-3
Author Response: Residual density is close to a heavy atom and present despite applying absorption corrections. It is not chemically significant.

PLAT972_ALERT_2_B Check Calcd Resid. Dens. 0.86Ang From Th3 -3.13 eA-3
Author Response: Residual density is close to a heavy atom and present despite applying absorption corrections. It is not chemically significant.

PLAT972_ALERT_2_B Check Calcd Resid. Dens. 1.63Ang From Th2 -2.87 eA-3
Author Response: Residual density is close to a heavy atom and present despite applying absorption corrections. It is not chemically significant.

PLAT972_ALERT_2_B Check Calcd Resid. Dens. 1.63Ang From Th1 -2.72 eA-3
Author Response: Residual density is close to a heavy atom and present despite applying absorption corrections. It is not chemically significant.

PLAT972_ALERT_2_B Check Calcd Resid. Dens. 1.42Ang From C13 -2.68 eA-3
Author Response: Residual density is close to a heavy atom and present despite applying absorption corrections. It is not chemically significant.

PLAT972_ALERT_2_B Check Calcd Resid. Dens. 1.40Ang From C20 -2.66 eA-3
Author Response: Residual density is close to a heavy atom and present despite applying absorption corrections. It is not chemically significant.

PLAT972_ALERT_2_B Check Calcd Resid. Dens. 0.80Ang From Th1 -2.64 eA-3
Author Response: Residual density is close to a heavy atom and present despite applying absorption corrections. It is not chemically significant.

PLAT972_ALERT_2_B Check Calcd Resid. Dens. 0.93Ang From Th2 -2.54 eA-3
Author Response: Residual density is close to a heavy atom and present despite applying absorption corrections. It is not chemically significant.

PLAT973_ALERT_2_B Check Calcd Positive Resid. Density on Th2 1.51 eA-3
Author Response: Residual density is close to a heavy atom and present despite applying absorption corrections. It is not chemically significant.

6

Alert level A

PLAT971_ALERT_2_A Check Calcd Resid. Dens. 0.89Ang From Th2 4.28 eA-3
Author Response: Residual density is close to a heavy atom and present despite applying absorption corrections. It is not chemically significant.

PLAT971_ALERT_2_A Check Calcd Resid. Dens. 0.72Ang From Th1 3.59 eA-3
Author Response: Residual density is close to a heavy atom and present despite applying absorption corrections. It is not chemically significant.

PLAT971_ALERT_2_A Check Calcd Resid. Dens. 0.84Ang From Th1 3.55 eA-3
Author Response: Residual density is close to a heavy atom and present despite applying absorption corrections. It is not chemically significant.

PLAT973_ALERT_2_A Check Calcd Positive Resid. Density on Th2 2.11 eA-3
Author Response: Residual density is close to a heavy atom and present despite applying absorption corrections. It is not chemically significant.

Alert level B

PLAT342_ALERT_3_B Low Bond Precision on C-C Bonds .... 0.03259 Ang.

Author Response: Low precision is due to disorder through the structure.

PLAT971_ALERT_2_B Check Calcd Resid. Dens. 0.93Ang From Th2 3.49 eA-3
Author Response: Residual density is close to a heavy atom and present.

PLAT971_ALERT_2_B Check Calcd Resid. Dens. 0.91Ang From Th3 3.41 eA-3
Author Response: Residual density is close to a heavy atom and present despite applying absorption corrections. It is not chemically significant.

PLAT971_ALERT_2_B Check Calcd Resid. Dens. 0.95Ang From Th3 3.21 eA-3
Author Response: Residual density is close to a heavy atom and present despite applying absorption corrections. It is not chemically significant.

PLAT972_ALERT_2_B Check Calcd Resid. Dens. 1.29Ang From O1 -2.97 eA-3
Author Response: Residual density is close to a heavy atom and present despite applying absorption corrections. It is not chemically significant.

PLAT972_ALERT_2_B Check Calcd Resid. Dens. 1.54Ang From Th3 -2.90 eA-3
Author Response: Residual density is close to a heavy atom and present despite applying absorption corrections. It is not chemically significant.

PLAT972_ALERT_2_B Check Calcd Resid. Dens. 0.89Ang From Th2 -2.90 eA-3
Author Response: Residual density is close to a heavy atom and present despite applying absorption corrections. It is not chemically significant.

PLAT972_ALERT_2_B Check Calcd Resid. Dens. 1.33Ang From C8 -2.84 eA-3
Author Response: Residual density is close to a heavy atom and present despite applying absorption corrections. It is not chemically significant.

PLAT972_ALERT_2_B Check Calcd Resid. Dens. 0.88Ang From Th2 -2.81 eA-3
Author Response: Residual density is close to a heavy atom and present despite applying absorption corrections. It is not chemically significant.

PLAT972_ALERT_2_B Check Calcd Resid. Dens. 1.16Ang From C16 -2.61 eA-3
Author Response: Residual density is close to a heavy atom and present despite applying absorption corrections. It is not chemically significant.

PLAT972_ALERT_2_B Check Calcd Resid. Dens. 1.62Ang From Th3 -2.60 eA-3
Author Response: Residual density is close to a heavy atom and present despite applying absorption corrections. It is not chemically significant.

PLAT972_ALERT_2_B Check Calcd Resid. Dens. 0.83Ang From Th1 -2.55 eA-3
Author Response: Residual density is close to a heavy atom and present despite applying absorption corrections. It is not chemically significant.

7
Alert level A
PLAT971_ALERT_2_A Check Calcd Resid. Dens. 0.98Ang From Th1 5.65 eA-3
Author Response: Residual density is close to a heavy atom and present despite applying absorption corrections.

PLAT973_ALERT_2_A Check Calcd Positive Resid. Density on Th3 2.44 eA-3

Author Response: Residual density is close to a heavy atom and present despite applying absorption corrections.

PLAT973_ALERT_2_A Check Calcd Positive Resid. Density on Th1 2.02 eA-3
Author Response: Residual density is close to a heavy atom and present despite applying absorption corrections.

Alert level B
PLAT234_ALERT_4_B Large Hirshfeld Difference Cs1 --O6 . 0.28 Ang.
Author Response: The atom assignment is correct and this is a result of poorly resolved cryptand units. Attempts to model over multiple sites led to unstable refinements.

PLAT241_ALERT_2_B High MainResAtom Ueq as Compared to Neighbours O2 Check
Author Response: The atom assignment is correct and this is a result of poorly resolved cryptand units. Attempts to model over multiple sites led to unstable refinements.

PLAT241_ALERT_2_B High MainResAtom Ueq as Compared to Neighbours O5 Check
Author Response: The atom assignment is correct and this is a result of poorly resolved cryptand units. Attempts to model over multiple sites led to unstable refinements.

PLAT342_ALERT_3_B Low Bond Precision on C-C Bonds .... 0.05179 Ang.
Author Response: Low precision is due to high levels of disorder present combined with low data quality.

PLAT971_ALERT_2_B Check Calcd Resid. Dens. 0.83Ang From Th2 3.48 eA-3
Author Response: Residual density is close to a heavy atom and present despite applying absorption corrections.

PLAT971_ALERT_2_B Check Calcd Resid. Dens. 0.78Ang From Th2 3.35 eA-3
Author Response: Residual density is close to a heavy atom and present despite applying absorption corrections.

PLAT971_ALERT_2_B Check Calcd Resid. Dens. 0.77Ang From Th3 3.10 eA-3
Author Response: Residual density is close to a heavy atom and present despite applying absorption corrections.

PLAT971_ALERT_2_B Check Calcd Resid. Dens. 0.91Ang From Th3 2.53 eA-3
Author Response: Residual density is close to a heavy atom and present despite applying absorption corrections.

PLAT972_ALERT_2_B Check Calcd Resid. Dens. 0.76Ang From Th1 -2.85 eA-3
Author Response: Residual density is close to a heavy atom and present despite applying absorption corrections.

PLAT972_ALERT_2_B Check Calcd Resid. Dens. 1.73Ang From Th3 -2.77 eA-3
Author Response: Residual density is close to a heavy atom and present despite applying absorption corrections.

PLAT972_ALERT_2_B Check Calcd Resid. Dens. 1.75Ang From C24 -2.60 eA-3
Author Response: Residual density is close to a heavy atom and present despite applying absorption corrections.

PLAT972_ALERT_2_B Check Calcd Resid. Dens. 0.90Ang From Th2 -2.55 eA-3
Author Response: Residual density is close to a heavy atom and present despite applying absorption corrections.

Supplementary Figures

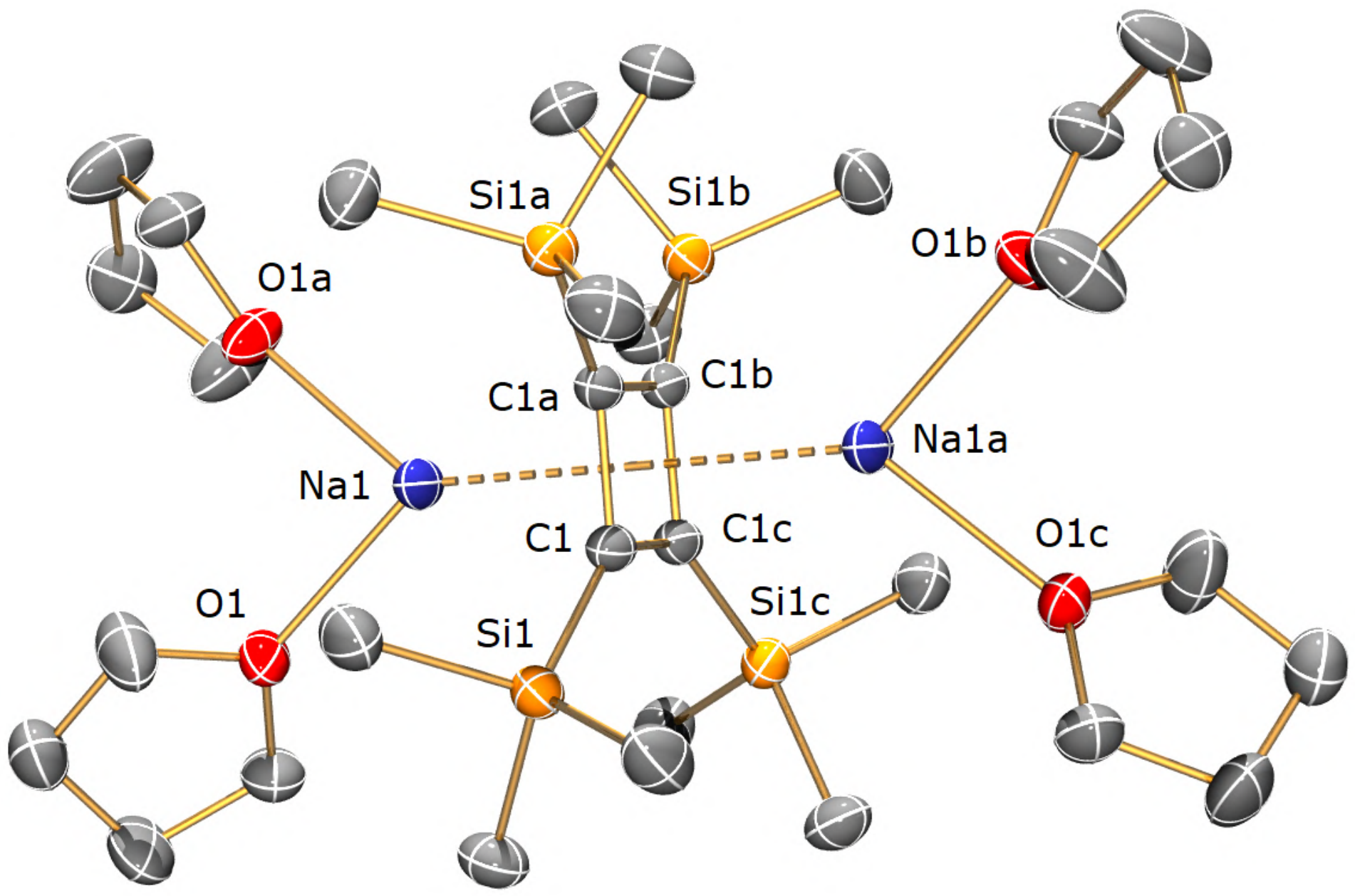

Supplementary Figure 1. Molecular structure of **2** at 100 K with selected atom labels. Displacement ellipsoids are set at 40% and hydrogen atoms are omitted for clarity. CCDC 2531345.

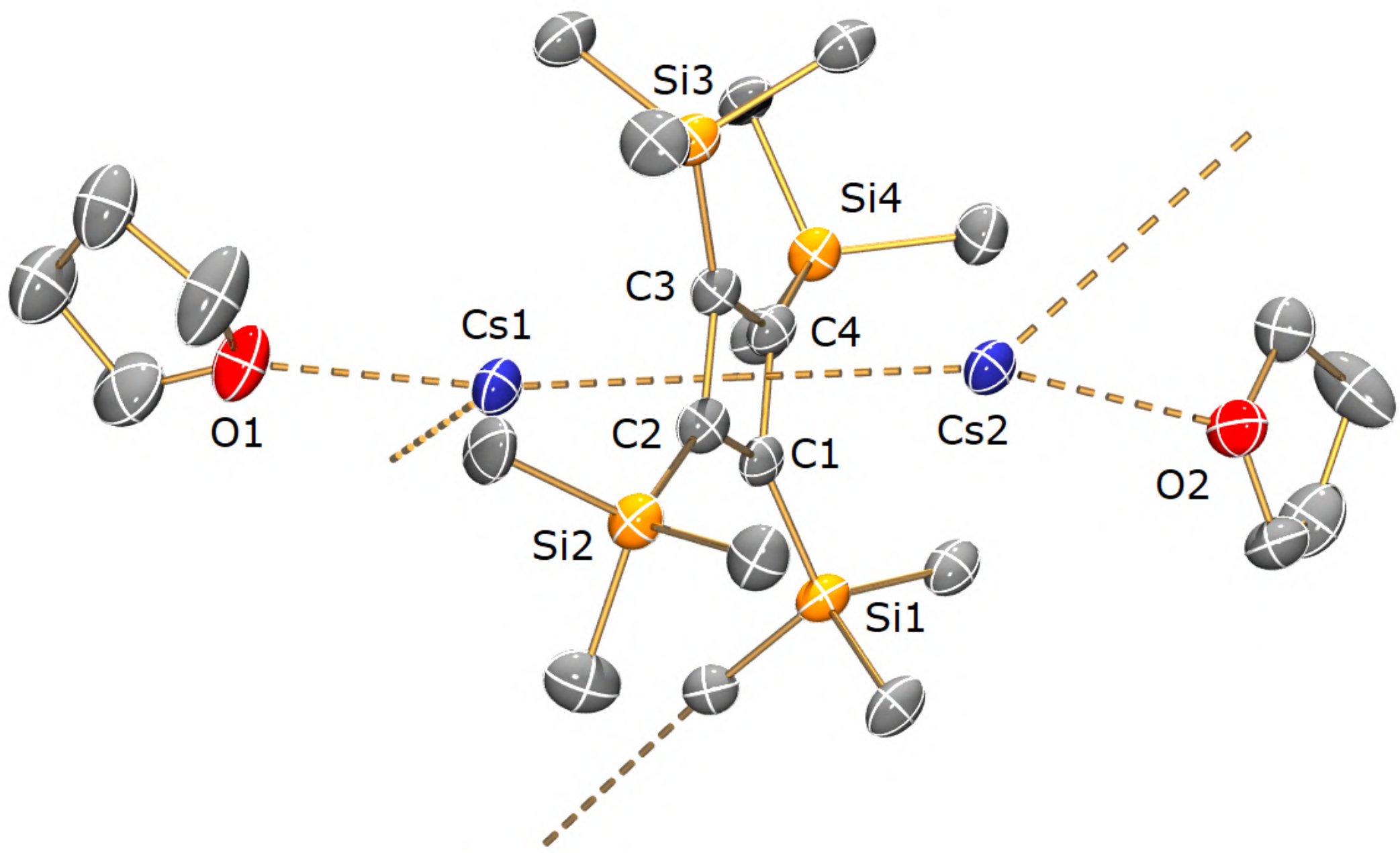

Supplementary Figure 2. Molecular structure of **3** at 100 K with selected atom labels. Displacement ellipsoids are set at 40% and hydrogen atoms are omitted for clarity. CCDC 2531346.

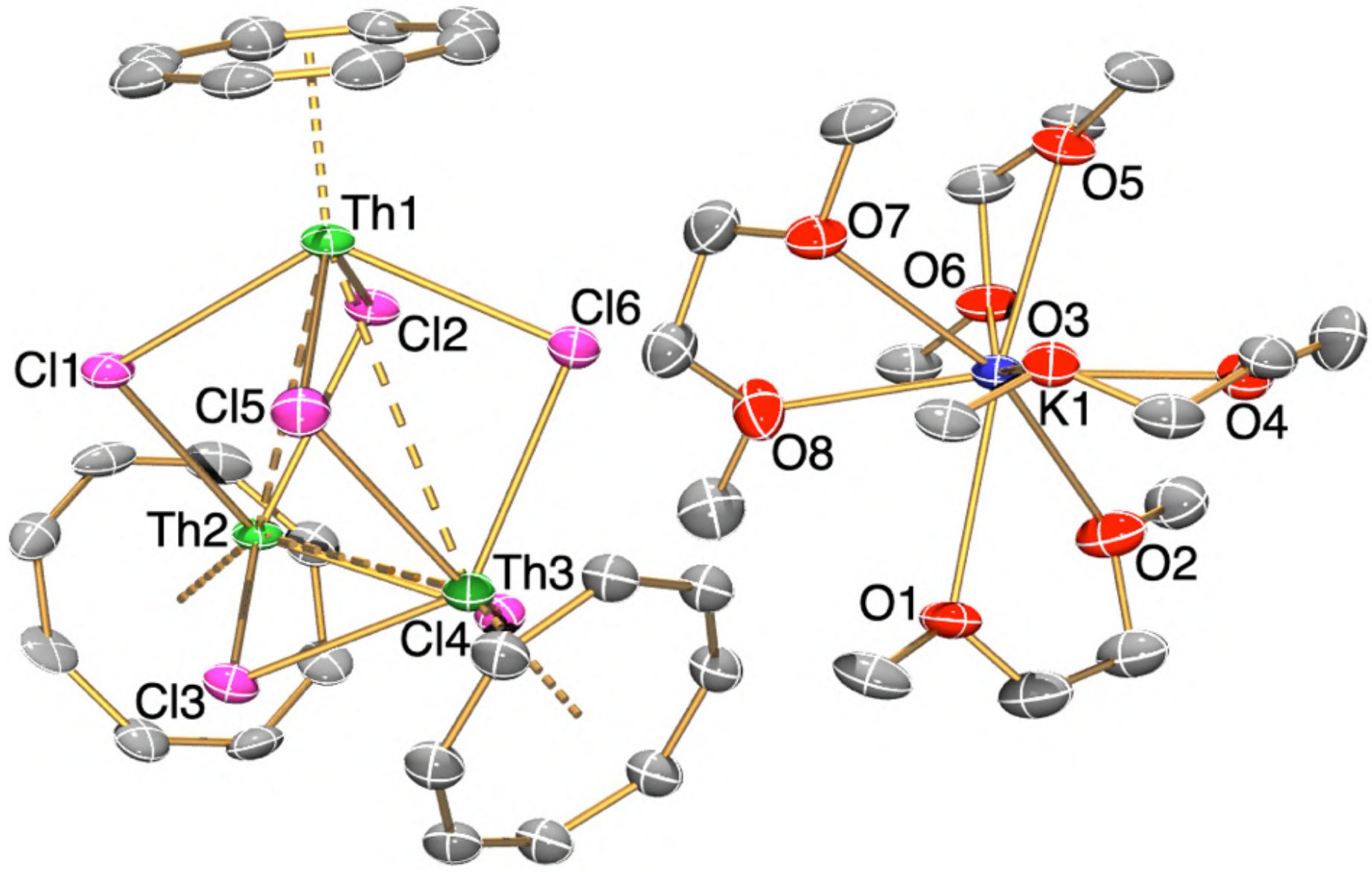

Supplementary Figure 3. Molecular structure of **4K** at 100 K with selected atom labels. Displacement ellipsoids are set at 40% and hydrogen atoms and disorder components are omitted for clarity. CCDC 2531347.

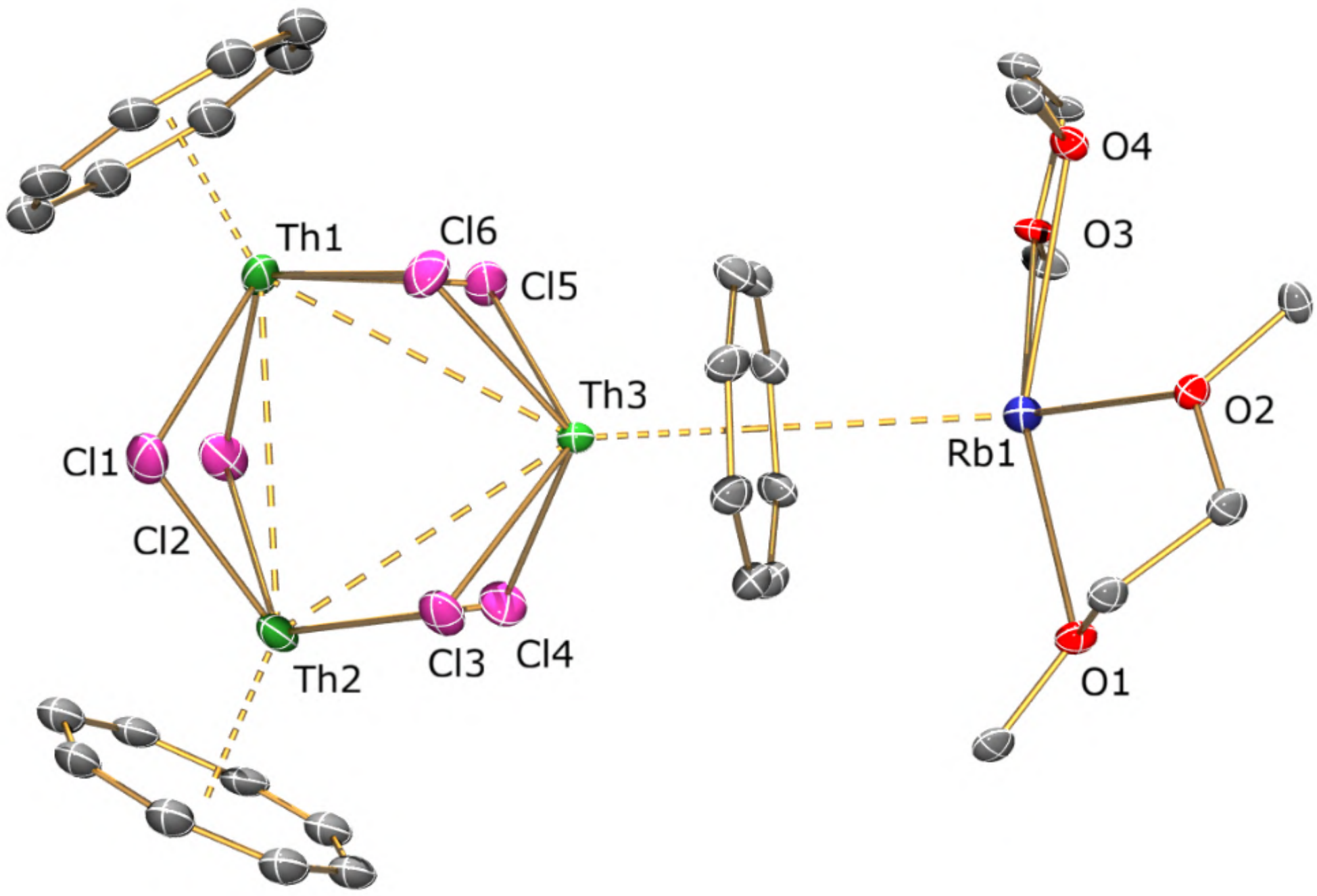

Supplementary Figure 4. Molecular structure of **4aRb** at 103 K with selected atom labels. Displacement ellipsoids are set at 40% and hydrogen atoms and disordered components are omitted for clarity. CCDC 2531348.

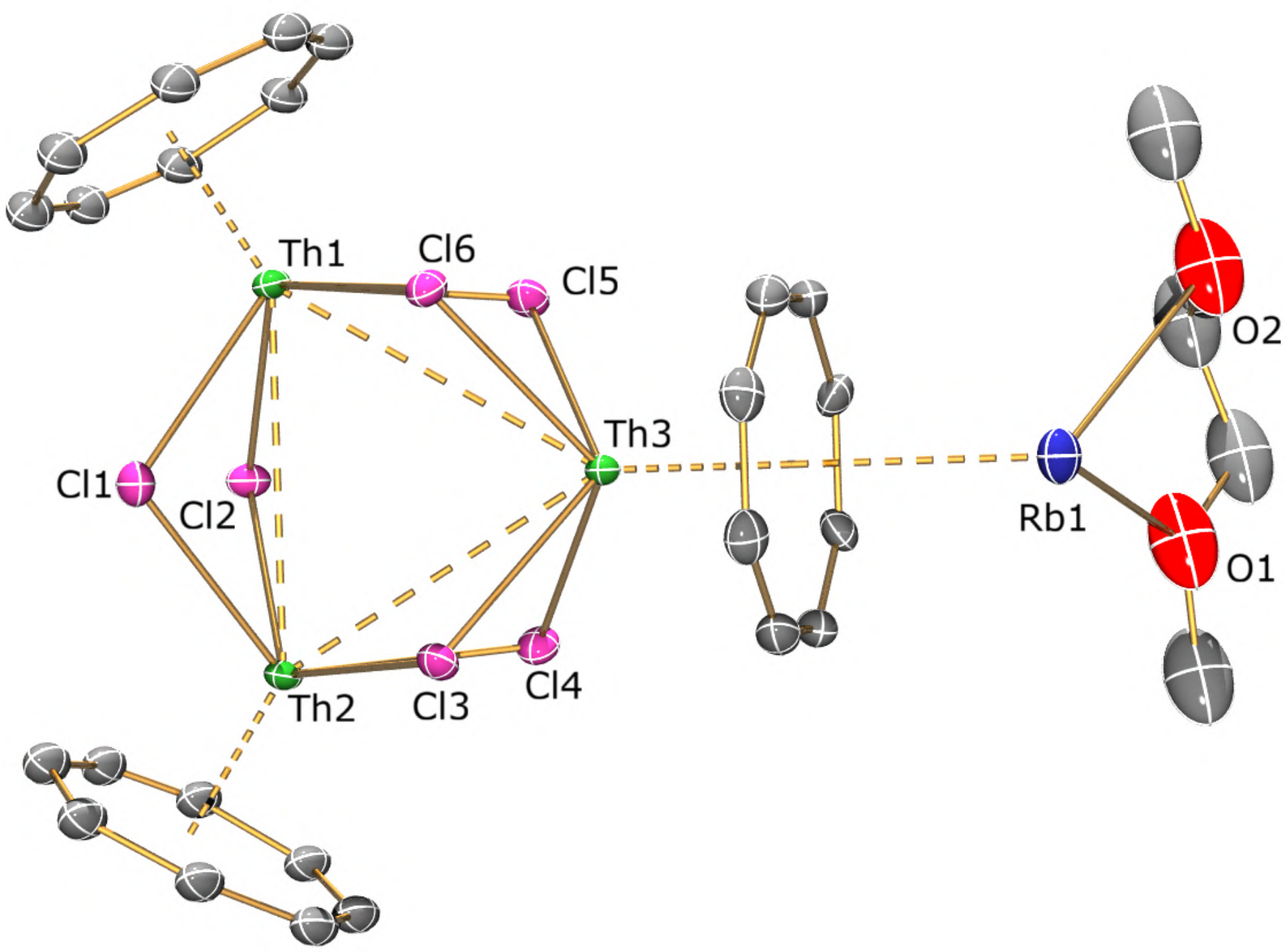

Supplementary Figure 5. Molecular structure of **4bRb** at 150 K with selected atom labels. Displacement ellipsoids are set at 40% and hydrogen atoms and disorder components are omitted for clarity. CCDC 2531349.

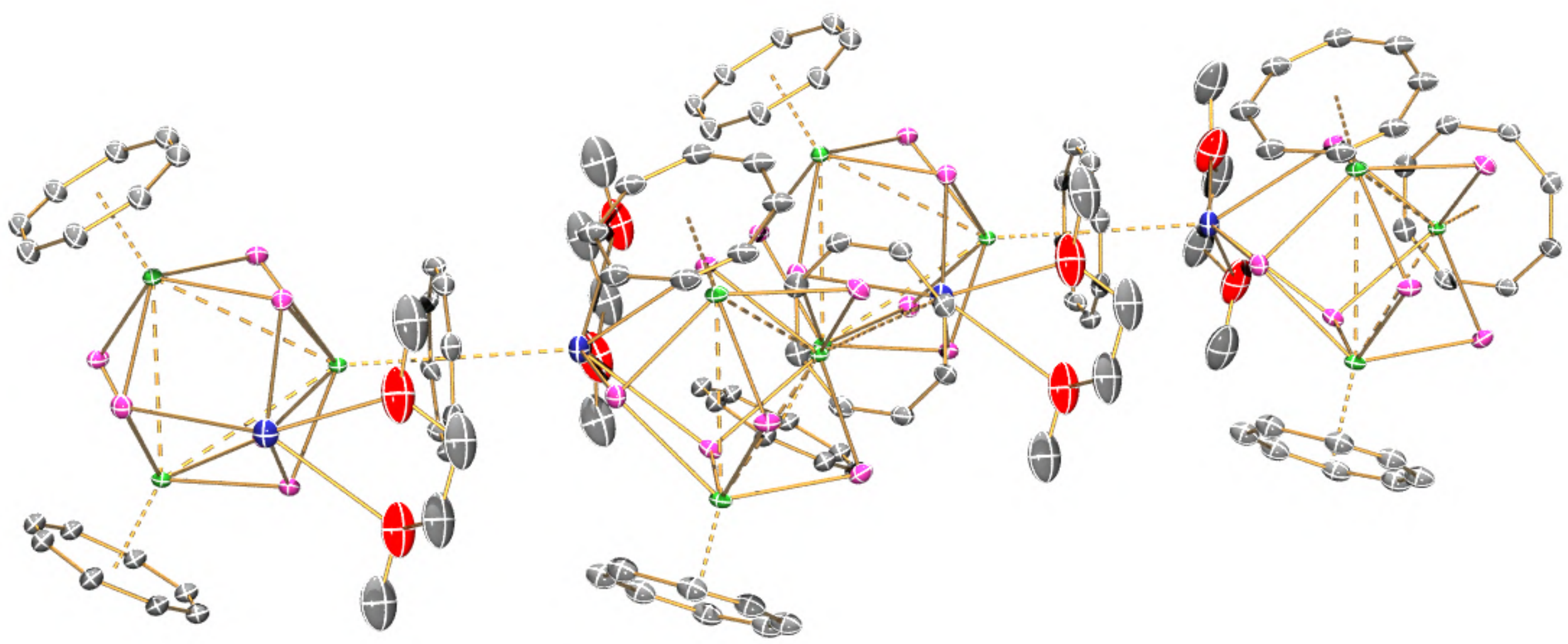

Supplementary Figure 6. Polymeric structure of **4bRb** at 150 K with selected atom labels. Displacement ellipsoids are set at 40% and hydrogen atoms and disorder components are omitted for clarity. Key: thorium, green; chlorine, magenta; rubidium, blue; carbon, grey; oxygen, red. CCDC 2531349.

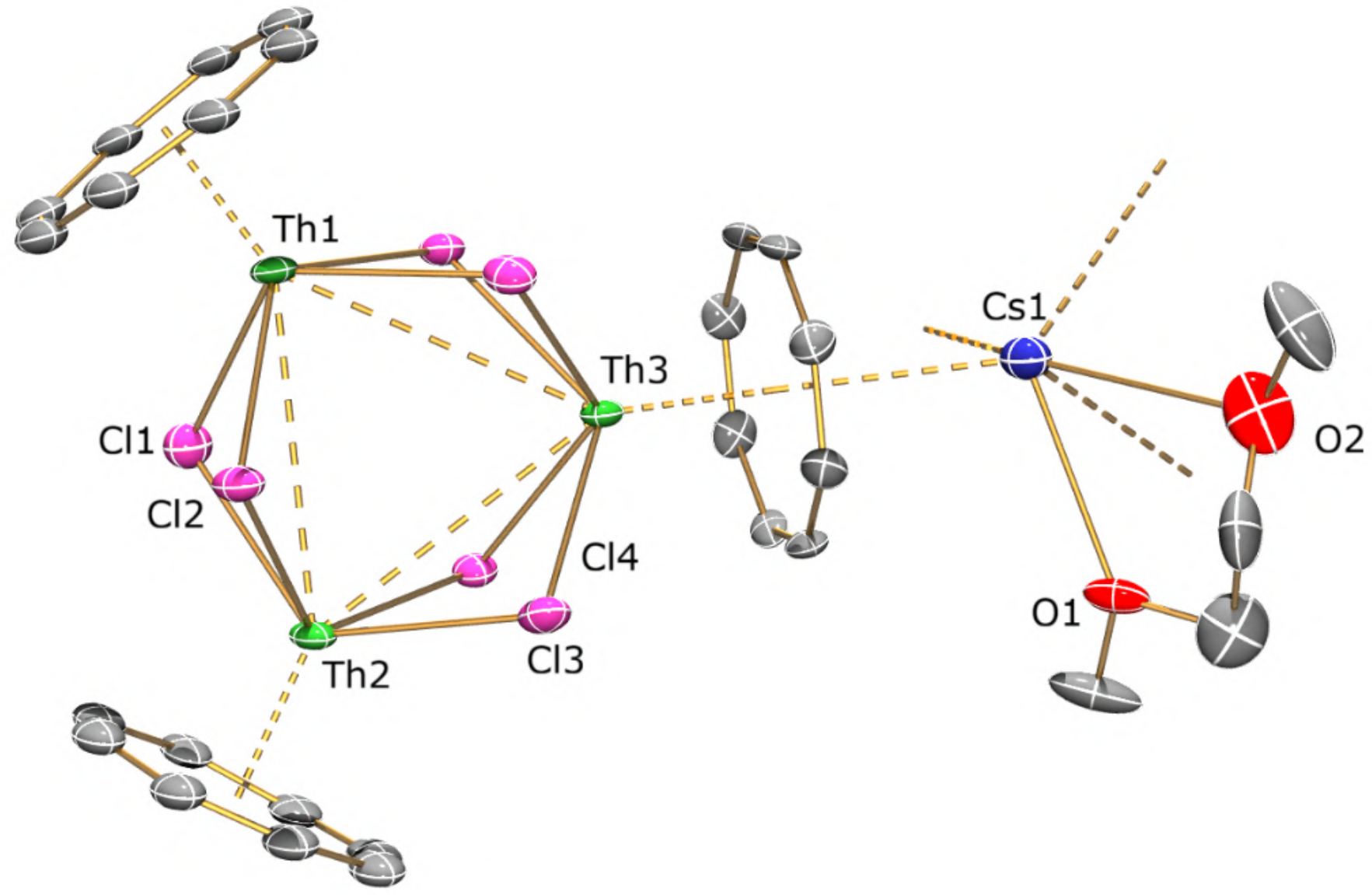

Supplementary Figure 7. Molecular structure of **4Cs** at 100 K with selected atom labels. Displacement ellipsoids are set at 40% and hydrogen atoms and disordered components are omitted for clarity. CCDC 2531350.

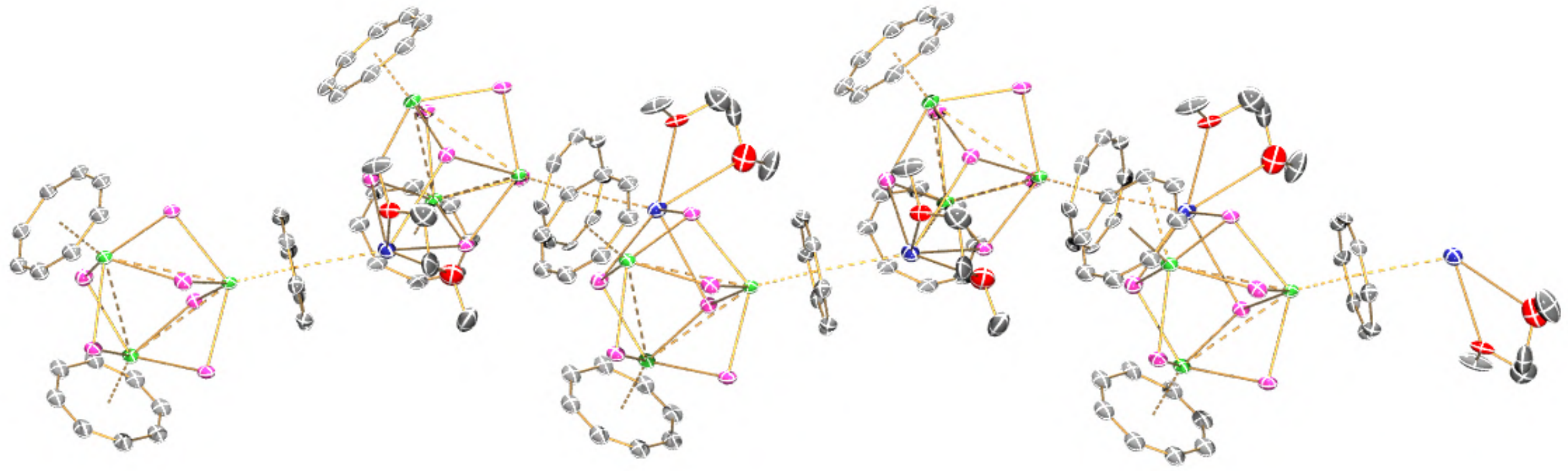

Supplementary Figure 8. Polymeric structure of **4Cs** at 100 K with selected atom labels. Displacement ellipsoids are set at 40% and hydrogen atoms and disordered components are omitted for clarity. Key: thorium, green; chlorine, magenta; caesium, blue; carbon, grey; oxygen, red. CCDC 2531350.

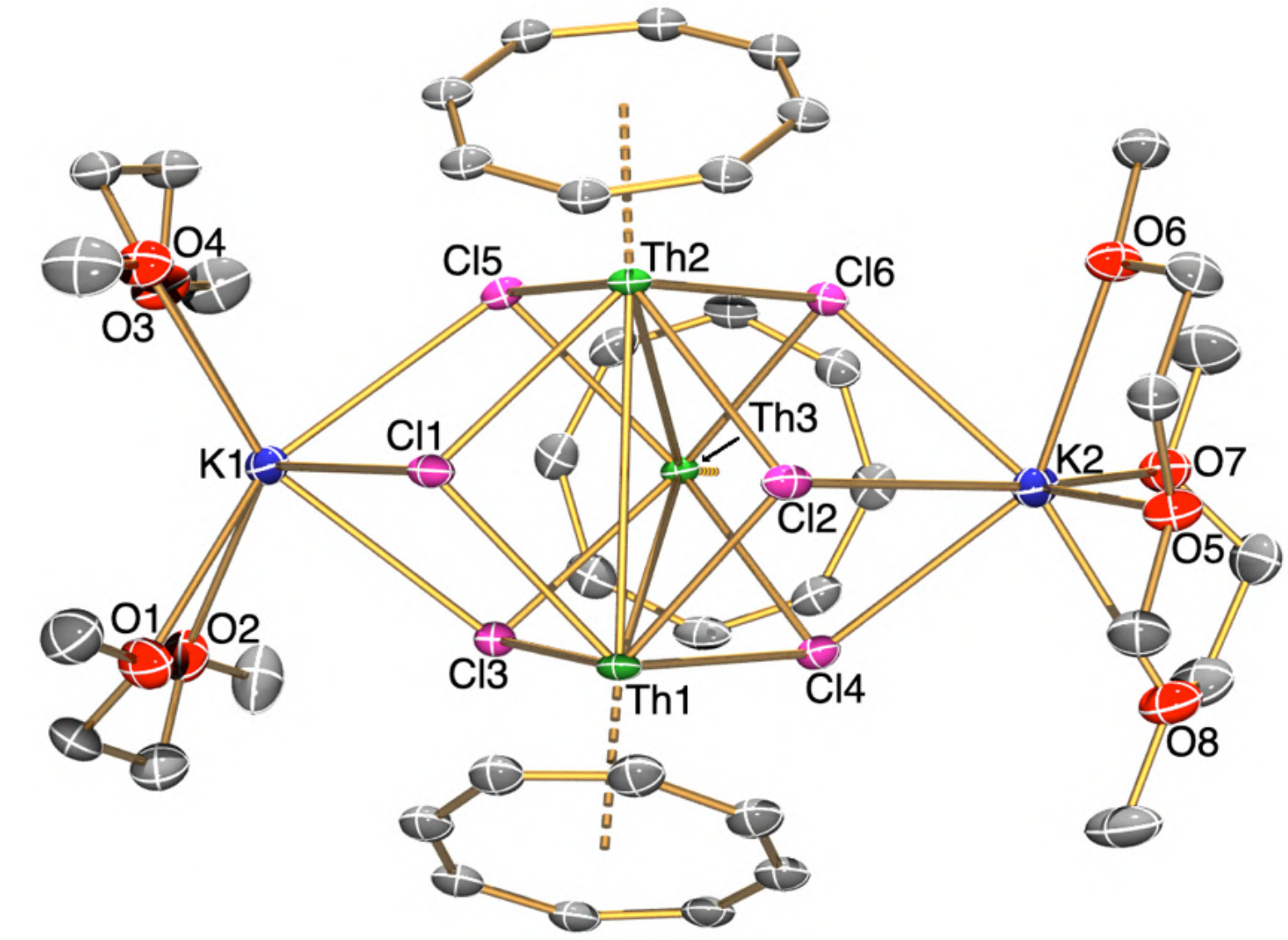

Supplementary Figure 9. Molecular structure of **5K** at 100 K with selected atom labels. Displacement ellipsoids are set at 40% and hydrogen atoms and disordered components are omitted for clarity. CCDC 2531351.

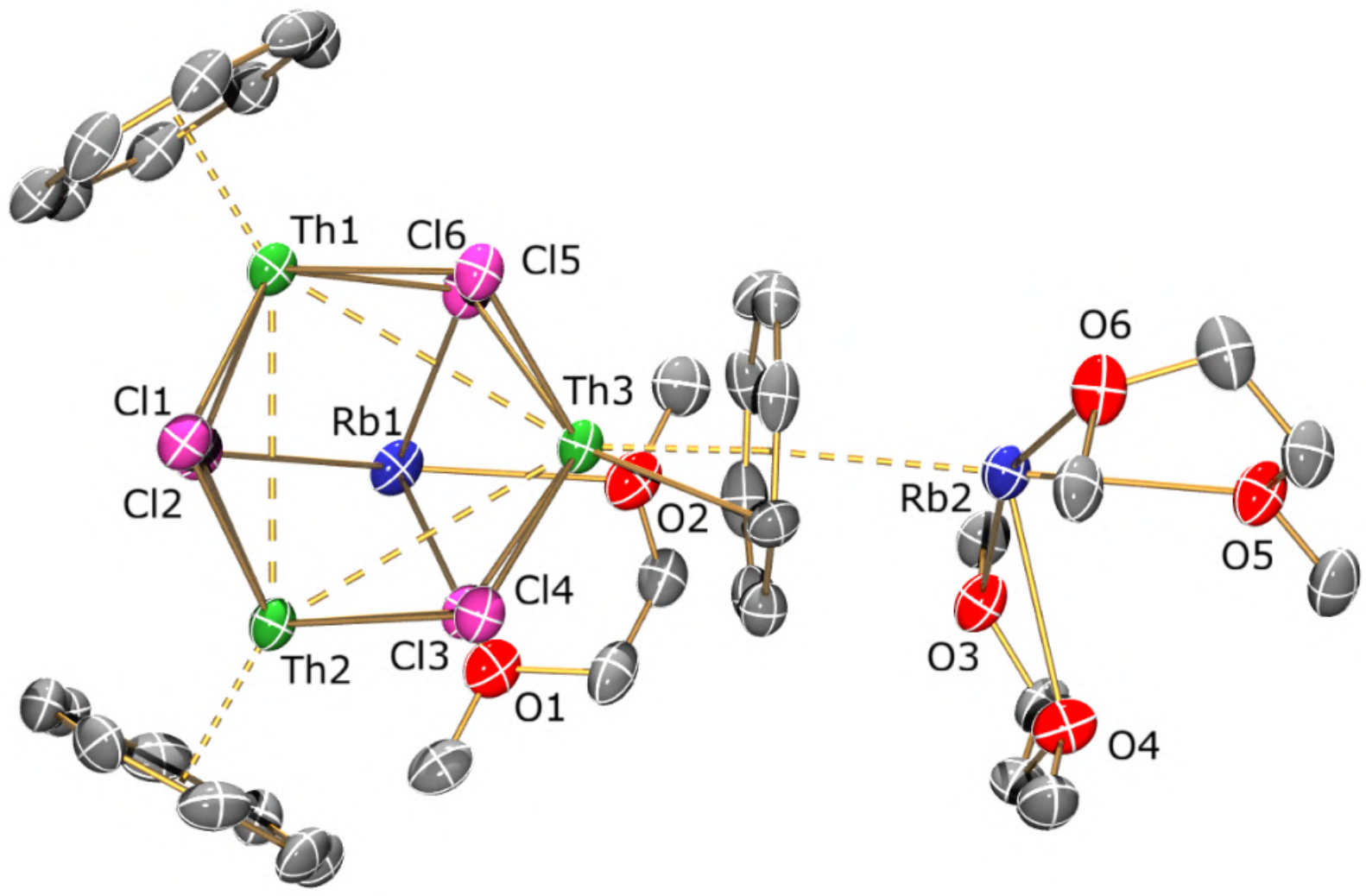

Supplementary Figure 10. Molecular structure of **5Rb** at 100 K with selected atom labels. Displacement ellipsoids are set at 40% and hydrogen atoms and lattice DME solvent are omitted for clarity. CCDC 2531352.

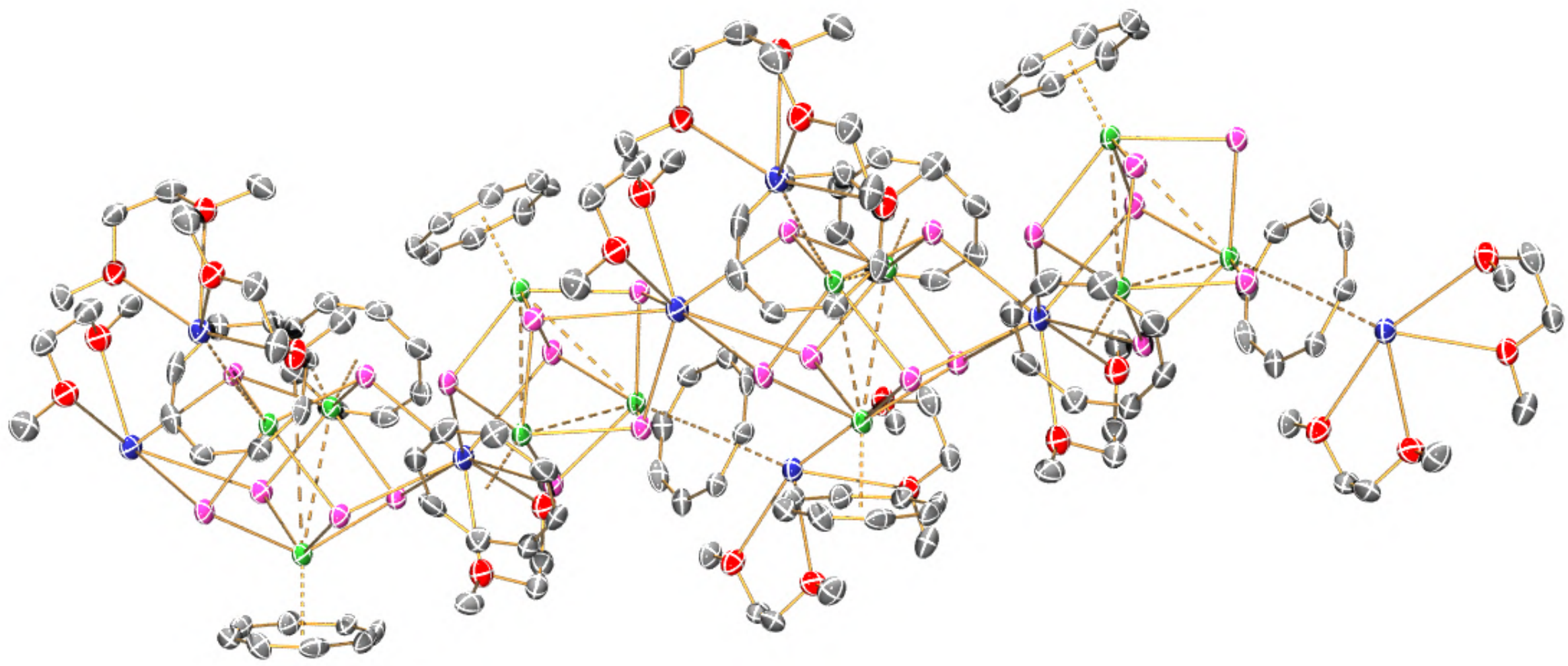

Supplementary Figure 11. Polymeric structure of **5Rb** at 100 K with selected atom labels. Displacement ellipsoids are set at 40% and hydrogen atoms and lattice DME solvent are omitted for clarity. Key: thorium, green; chlorine, magenta; rubidium, blue; carbon, grey; oxygen, red. CCDC 2531352.

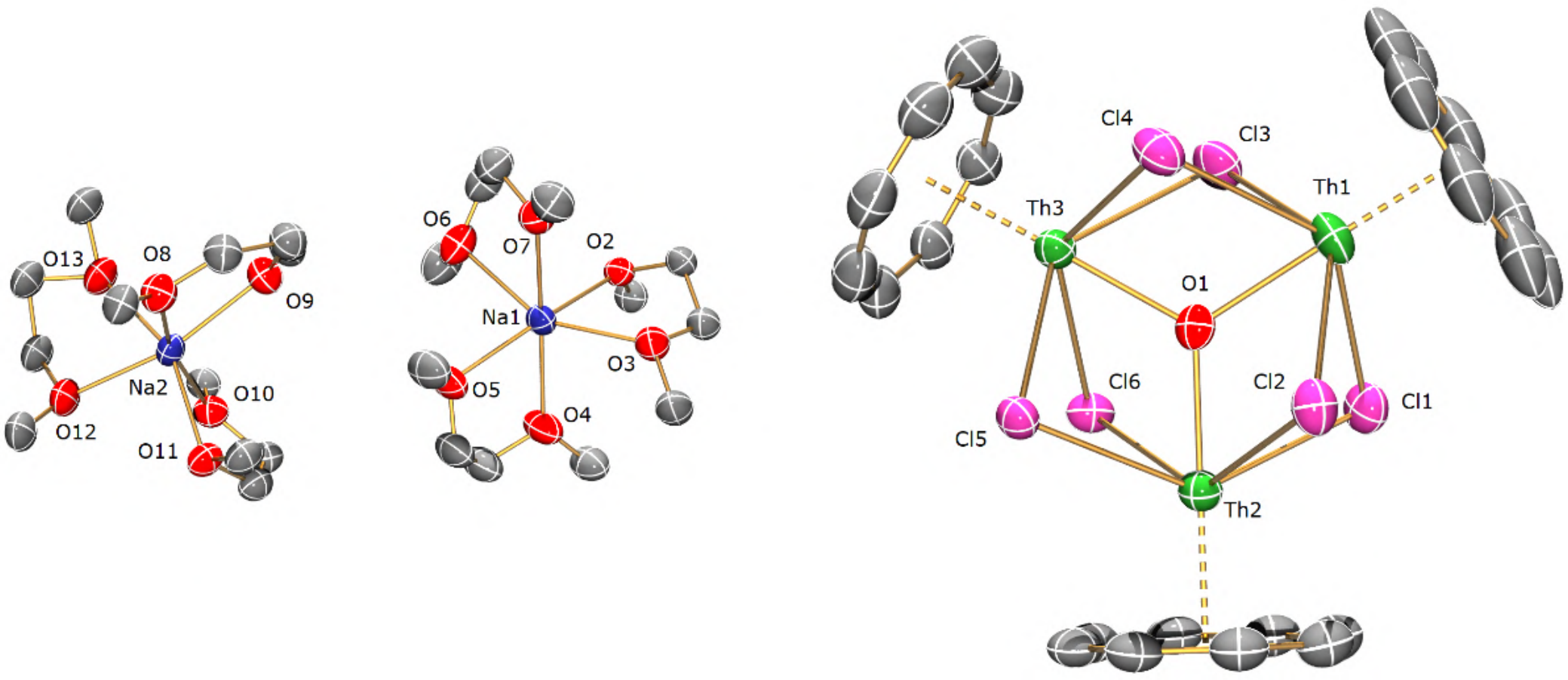

Supplementary Figure 12. Molecular structure of **6** at 100 K with selected atom labels. Displacement ellipsoids are set at 40% and hydrogen atoms and disordered components are omitted for clarity. CCDC 2531353.

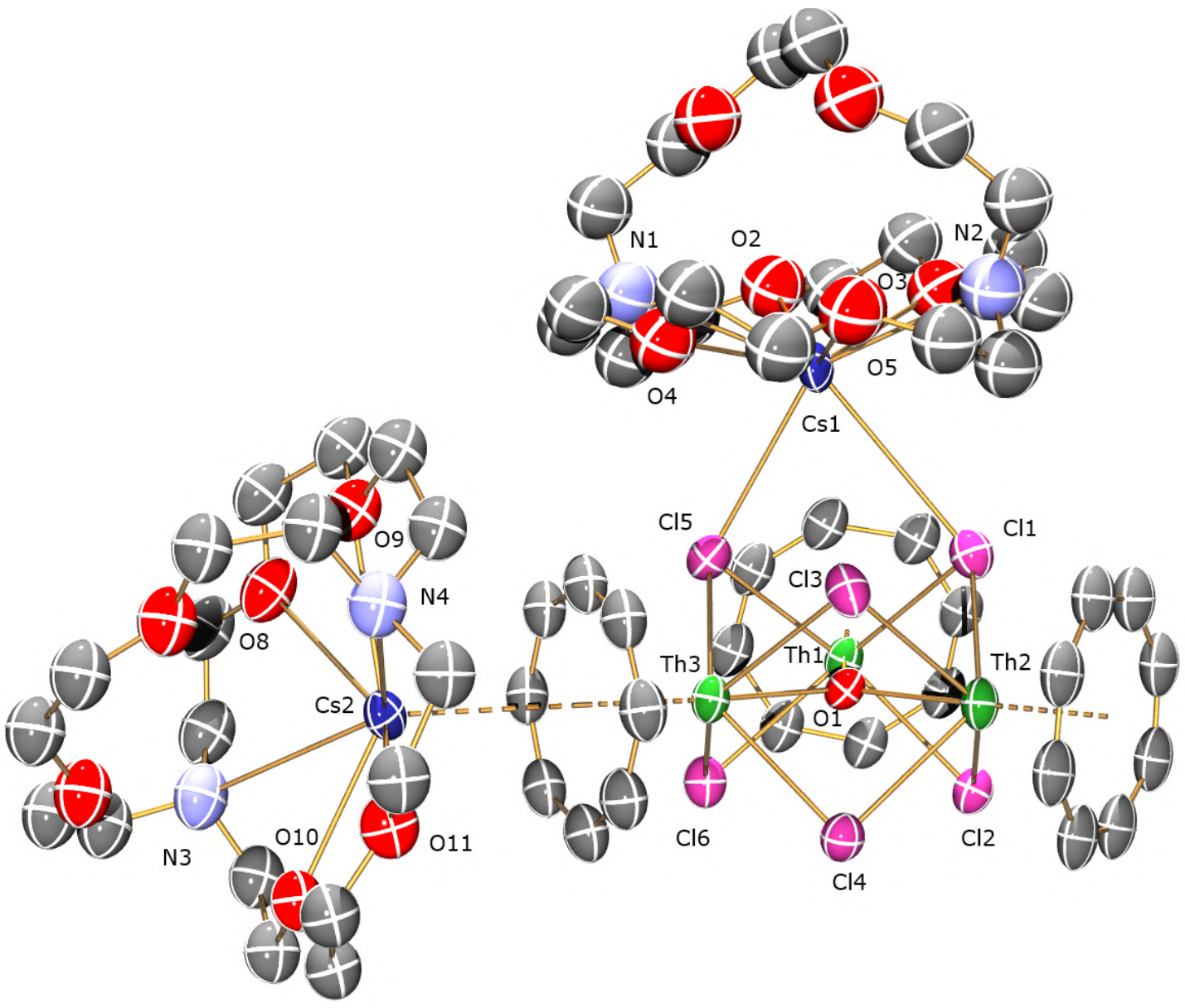

Supplementary Figure 13. Molecular structure of **7** at 100 K with selected atom labels. Displacement ellipsoids are set at 30% and hydrogen atoms and disordered components are omitted for clarity. CCDC 2531354.

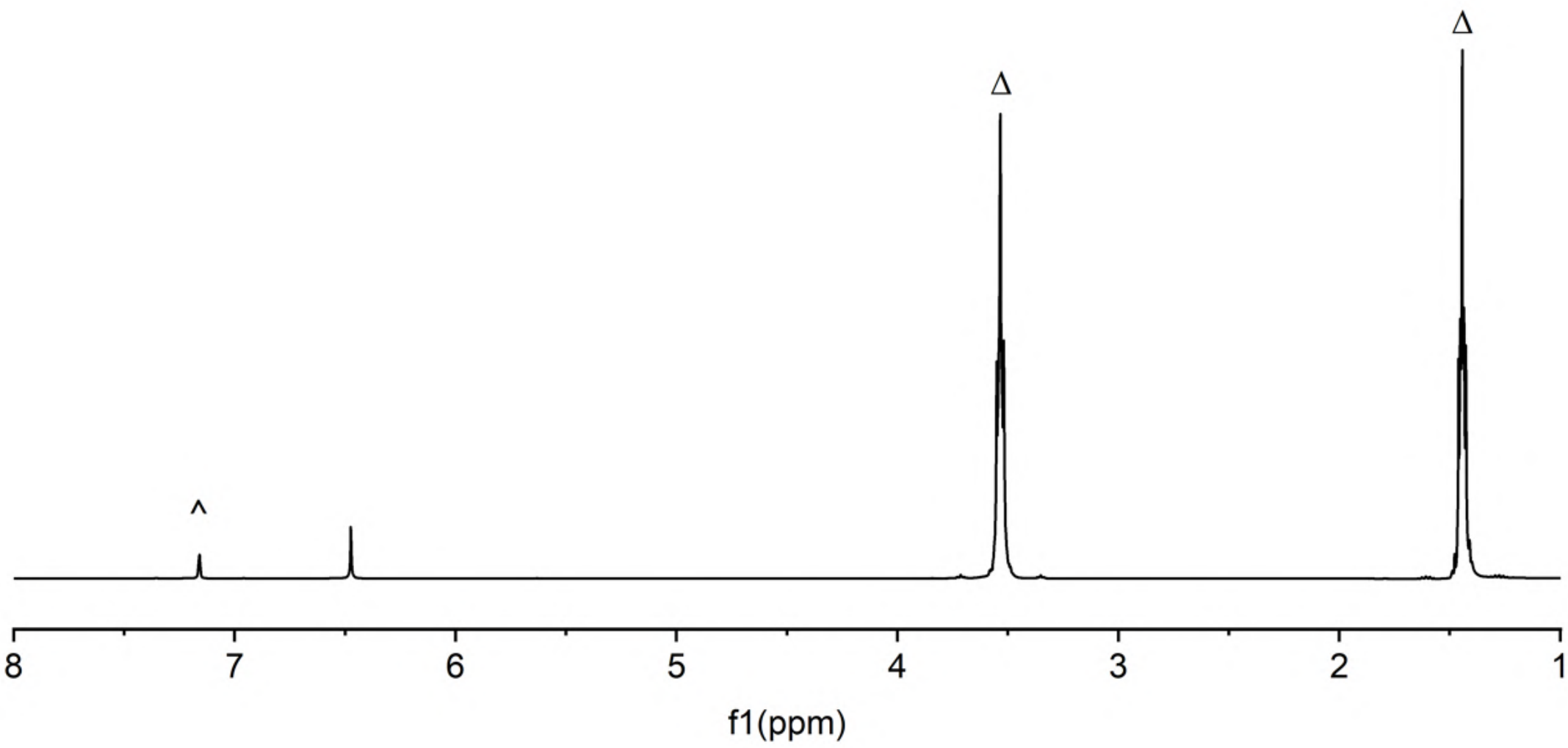

Supplementary Figure 14. ^{1}H NMR (C_6D_6, 298 K) spectrum of $C_8H_8K_2$. Δ = THF, a drop of which was used to aid solubility; ^ = C_6D_6 solvent.

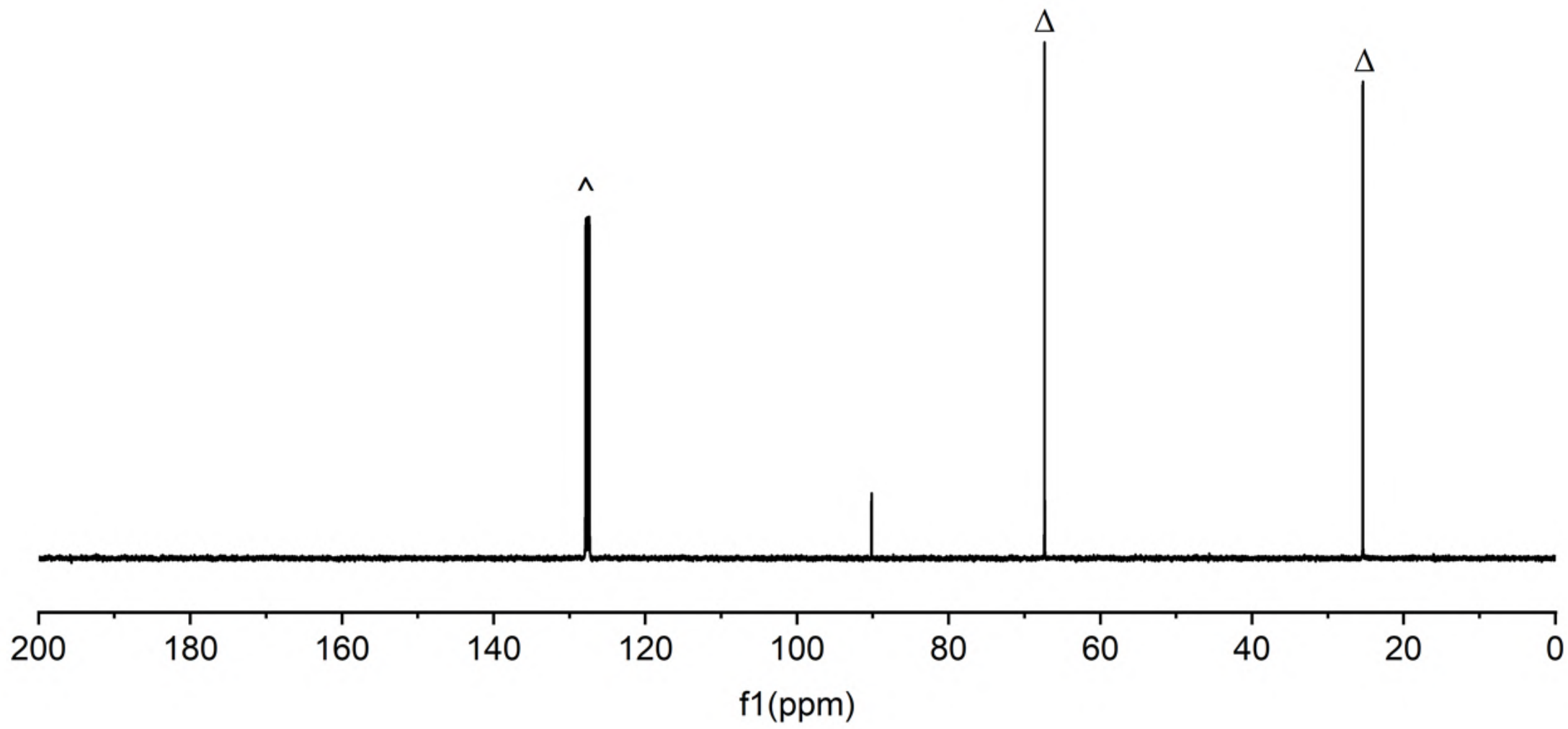

Supplementary Figure 15. ^{13}C NMR (C_6D_6 solvent, 298 K) spectrum of $C_8H_8K_2$. Δ = THF, a drop of which was used to aid solubility; ^ = C_6D_6 solvent.

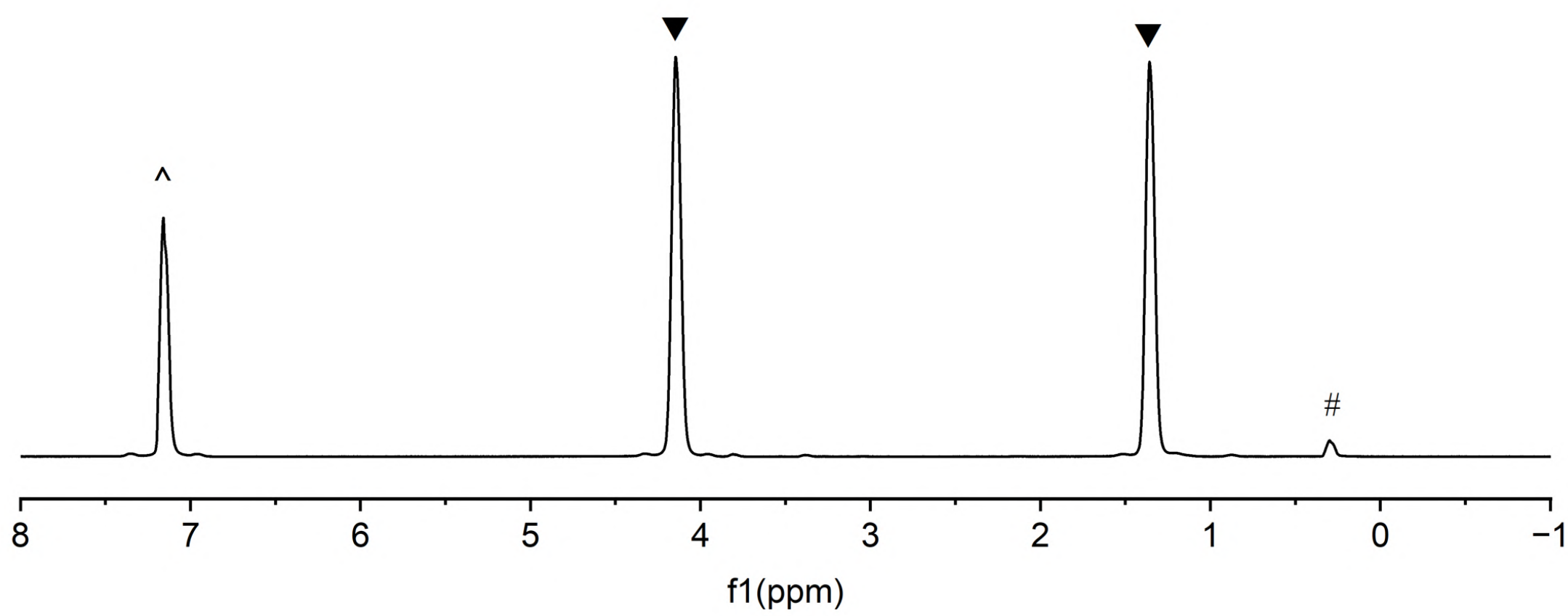

Supplementary Figure 16. ^{1}H NMR (C_6D_6, 298 K) spectrum of $ThCl_4(THF)_{3.5}$. ▼ = coordinated THF; # = silicone grease; ^ = C_6D_6 solvent.

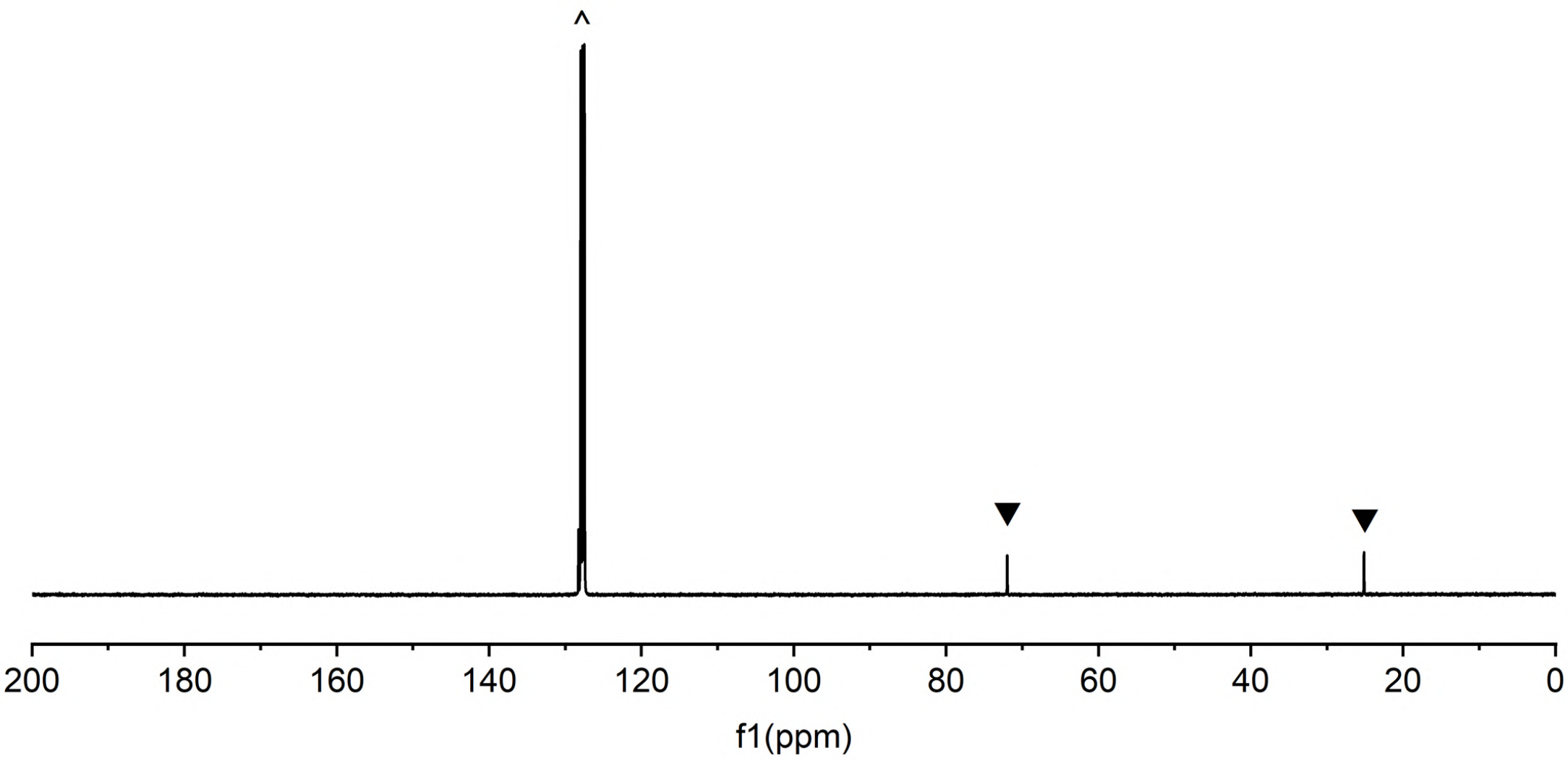

Supplementary Figure 17. $^{13}C\{^{1}H\}$ NMR (C_6D_6, 298 K) spectrum of $ThCl_4(THF)_{3.5}$. ▼ = coordinated THF; # = silicone grease; ^ = C_6D_6 solvent.

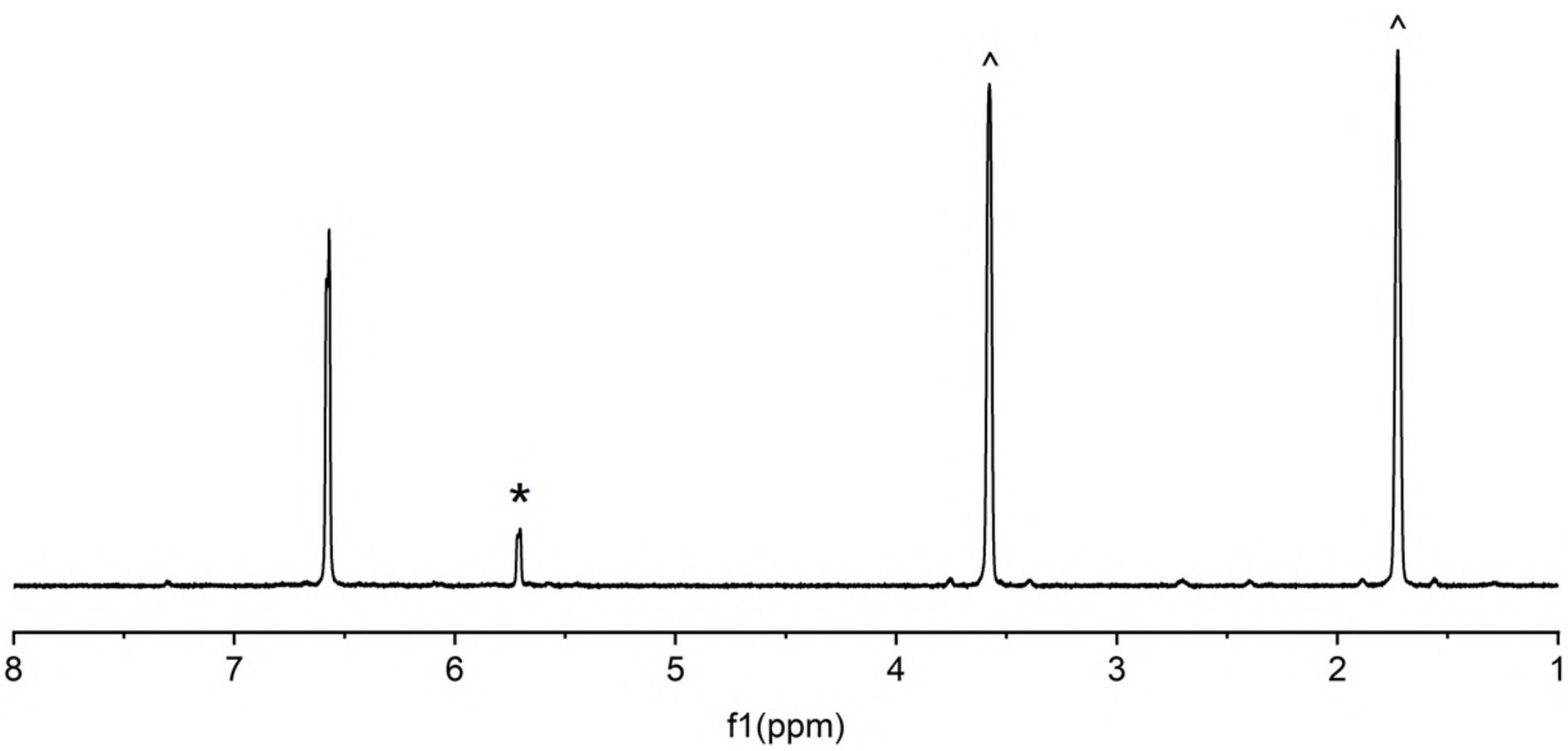

Supplementary Figure 18. ^{1}H NMR (D_8-THF, 298 K) spectrum of $[Th(\eta^8\text{-}C_8H_8)_2]$. The asterisk (*) at ~5.5 ppm denotes a trace impurity of C_8H_8. ^ = D_8-THF solvent.

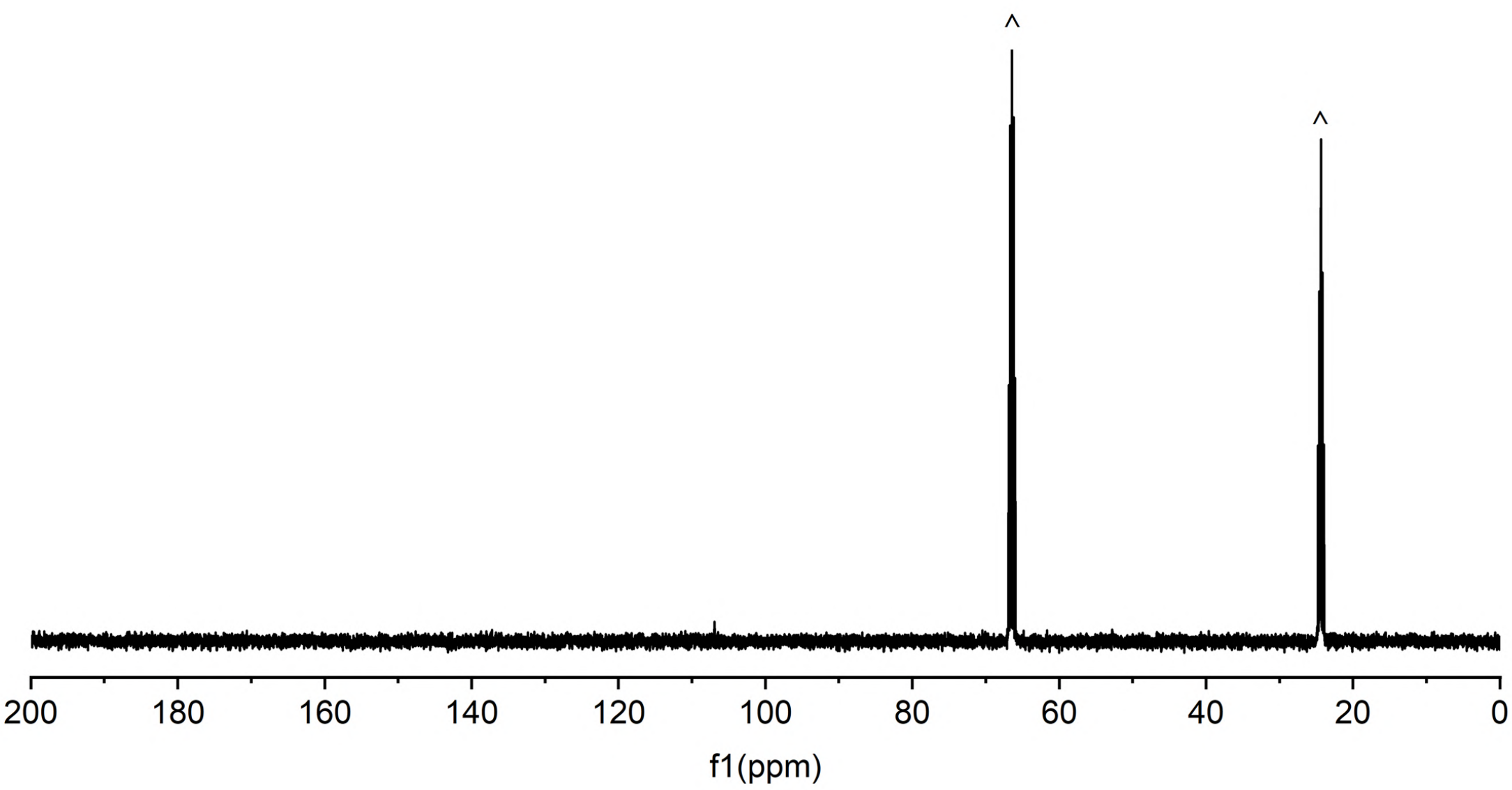

Supplementary Figure 19. $^{13}C\{^{1}H\}$ NMR (D_8-THF, 298 K) spectrum of $[Th(\eta^8\text{-}C_8H_8)_2]$. The weak nature of the signal arises from the poor solubility of $[Th(\eta^8\text{-}C_8H_8)_2]$ even in D_8-THF. ^ = D_8-THF solvent.

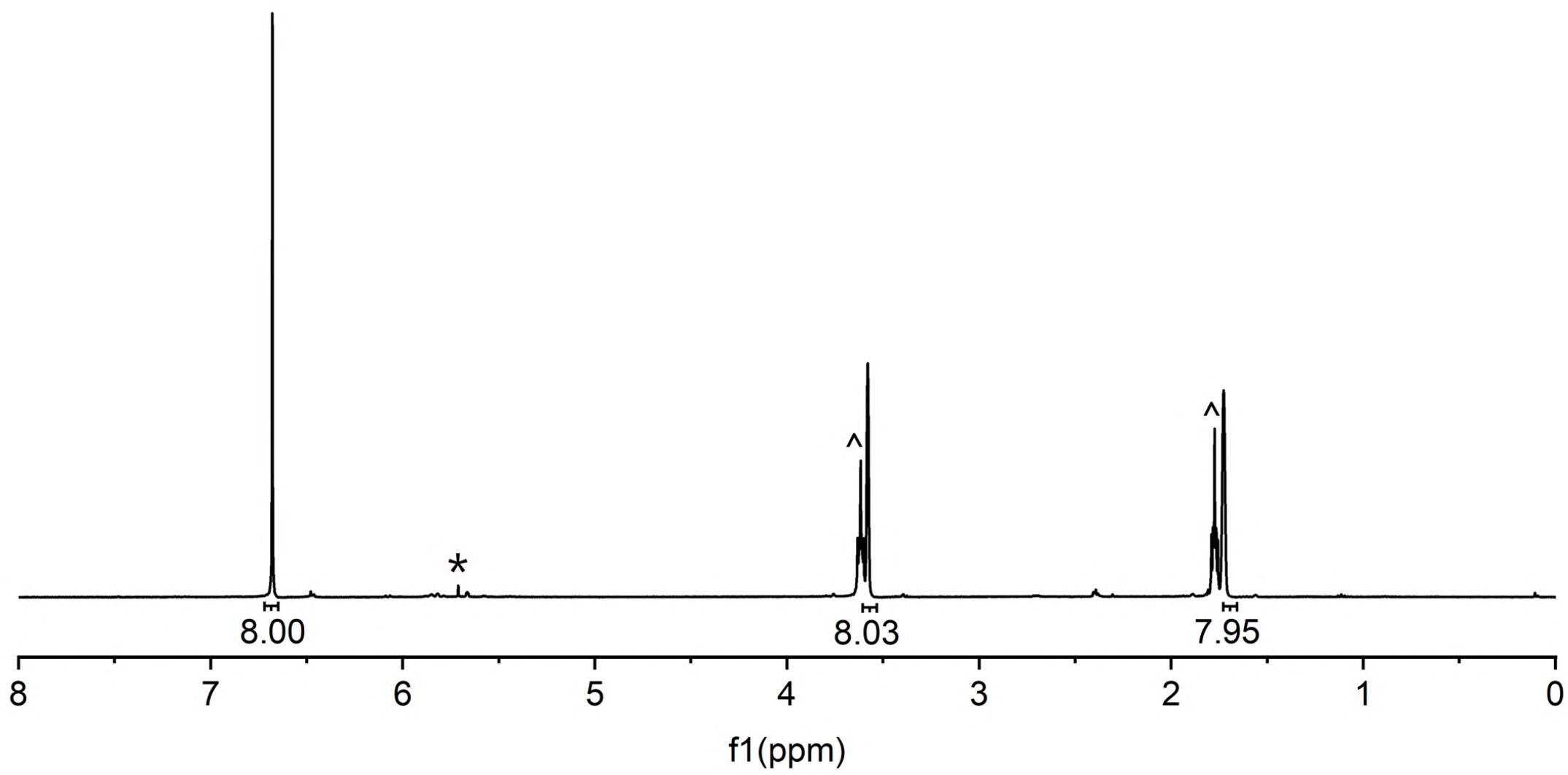

Supplementary Figure 20. ^{1}H NMR (D_8-THF, 298 K) spectrum of **1**. * = trace C_8H_8; ^ = D_8-THF solvent.

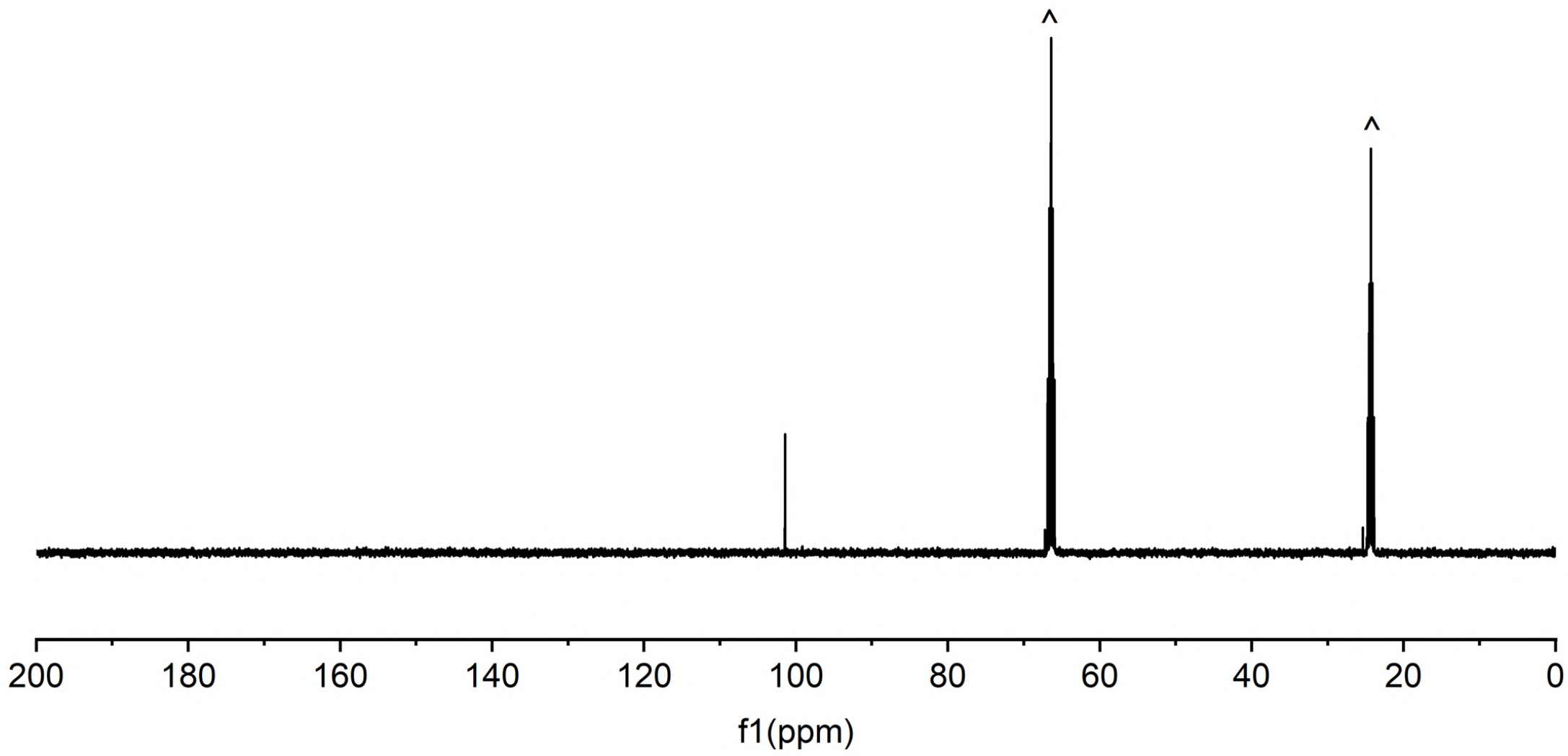

Supplementary Figure 21. $^{13}C\{^{1}H\}$ NMR (D_8-THF, 298 K) spectrum of **1**. ^ = D_8-THF solvent.

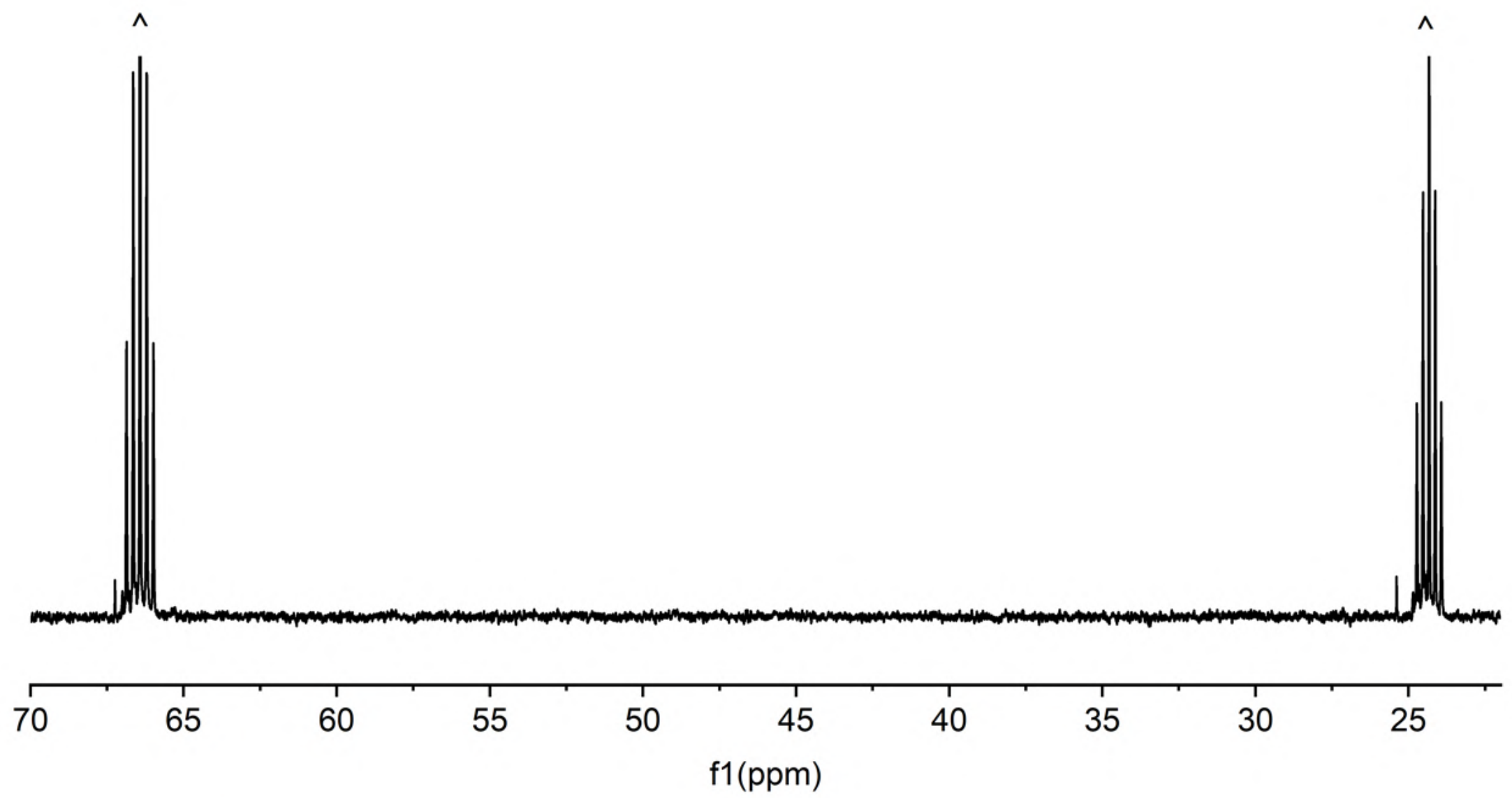

Supplementary Figure 22. Zoom-in of the $^{13}C\{^{1}H\}$ NMR (D_8-THF, 298 K) spectrum of **1** to highlight the small resonances at 68.39 and 26.53 ppm attributed to Th-coordinated THF. ^ = D_8-THF solvent.

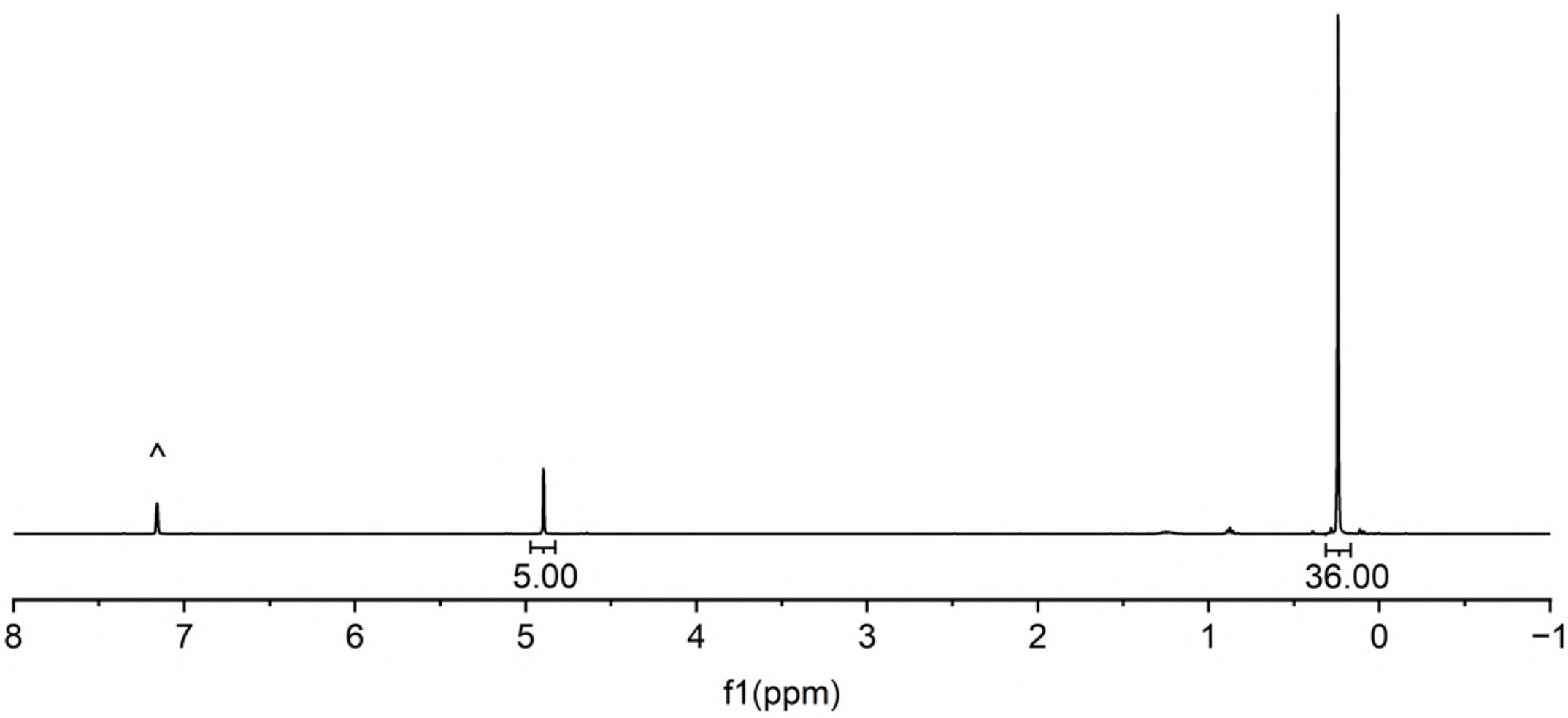

Supplementary Figure 23. ^{1}H NMR (C_6D_6, 298 K) spectrum of [Co{η^{4}-$C_4(SiMe_3)_4$}(η^{5}-C_5H_5)]. ^ = C_6D_6 solvent.

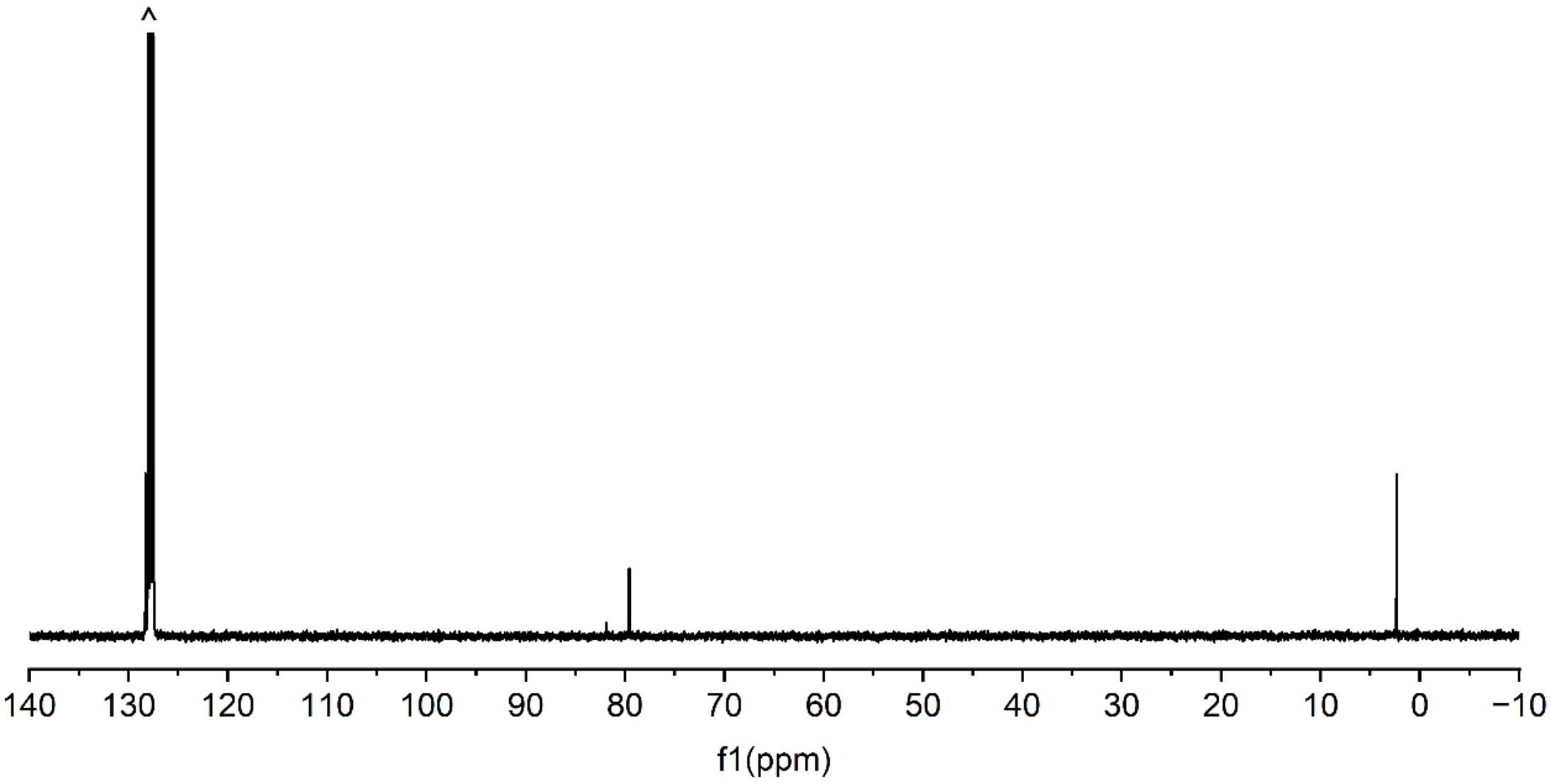

Supplementary Figure 24. $^{13}C\{^{1}H\}$ NMR (C_6D_6, 298 K) spectrum of [Co{η^{4}-$C_4(SiMe_3)_4$}(η^{5}-C_5H_5)]. ^ = C_6D_6 solvent.

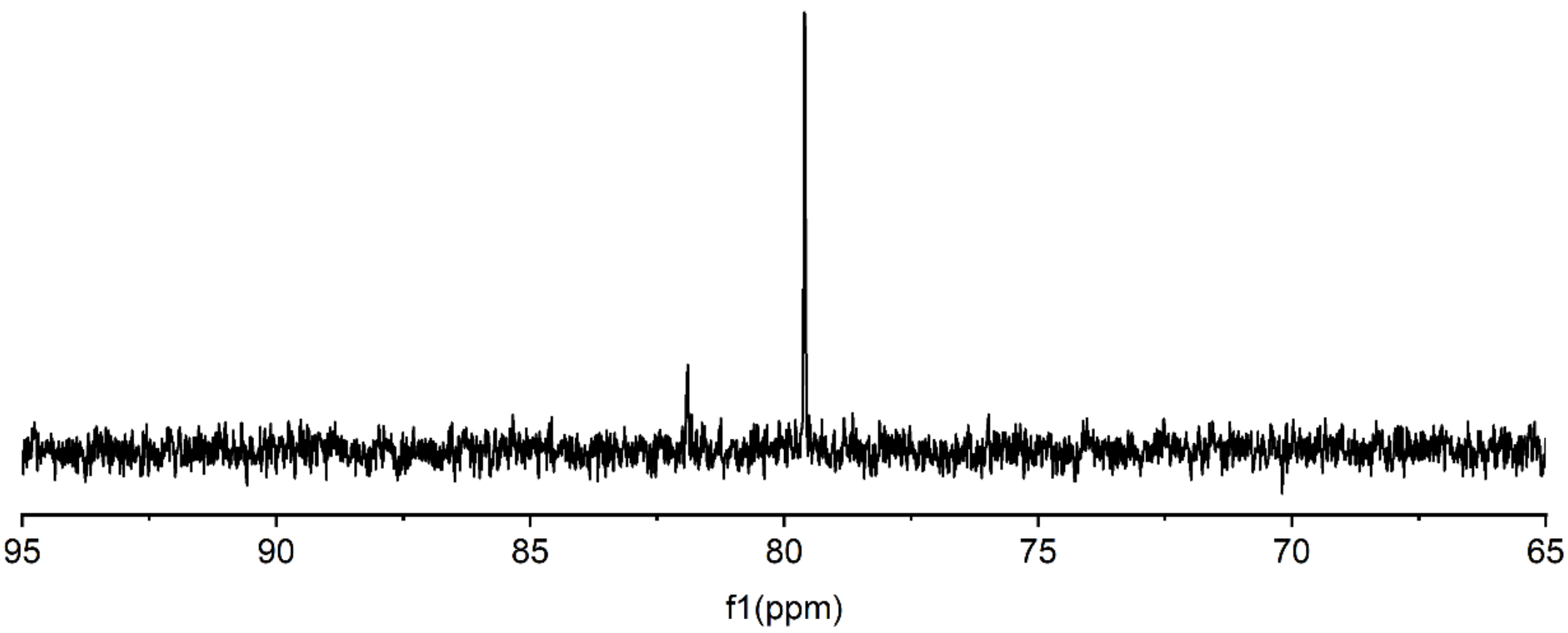

Supplementary Figure 25. Zoom-in of the $^{13}C\{^{1}H\}$ NMR (C_6D_6, 298 K) spectrum of [Co{η^{4}-$C_4(SiMe_3)_4$}(η^{5}-C_5H_5)] to highlight the C_4 resonance at ~81 ppm.

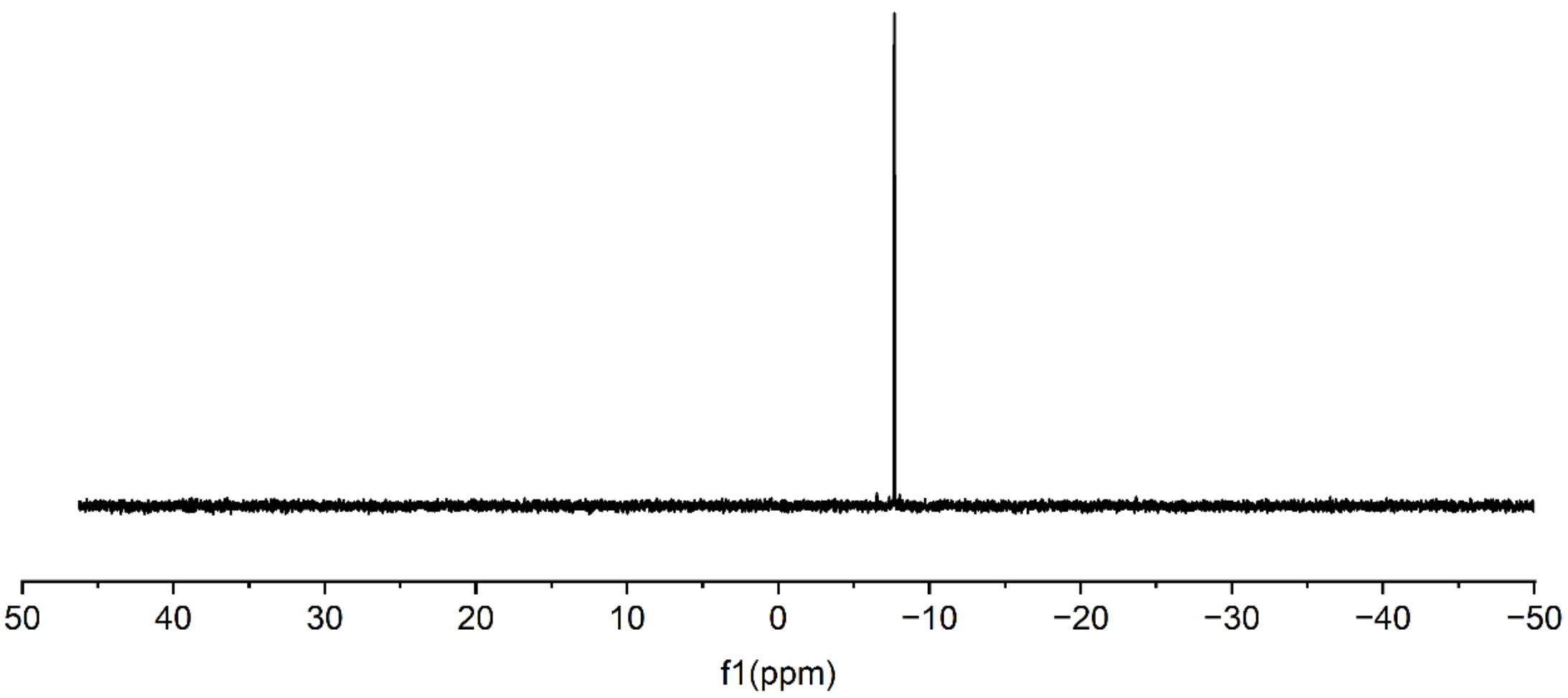

Supplementary Figure 26. $^{29}Si\{^{1}H\}$ NMR (C_6D_6, 298 K) spectrum of [Co{η^4-$C_4(SiMe_3)_4$}(η^5-C_5H_5)]. ^ = C_6D_6 solvent.

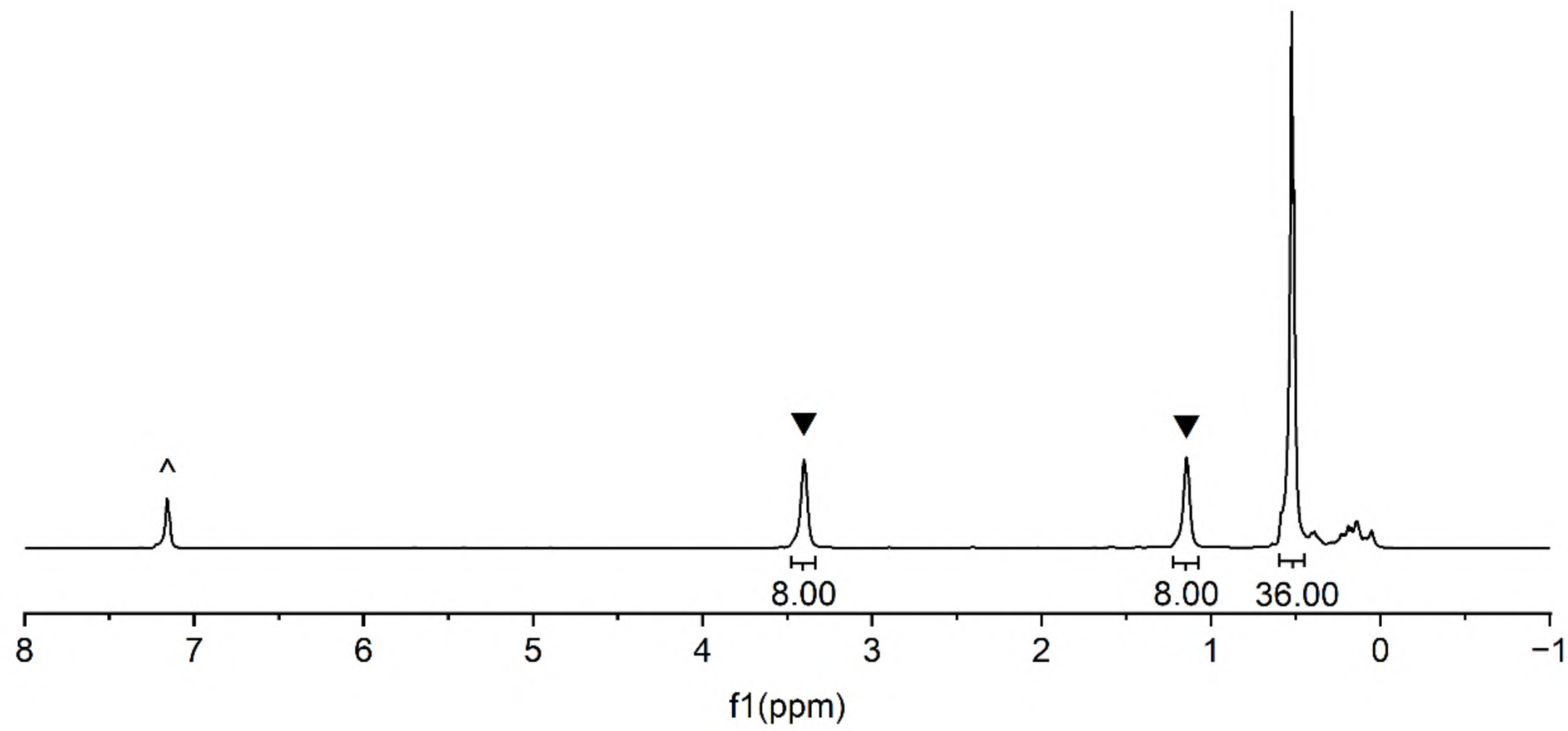

Supplementary Figure 27. ^{1}H NMR (C_6D_6, 298 K) spectrum of [$Li_2$$\{\mu$-$\eta^4$:$\eta^4$-$C_4(SiMe_3)_4\}$$(THF)_2$]. ▼ = coordinated THF; ^ = C_6D_6 solvent.

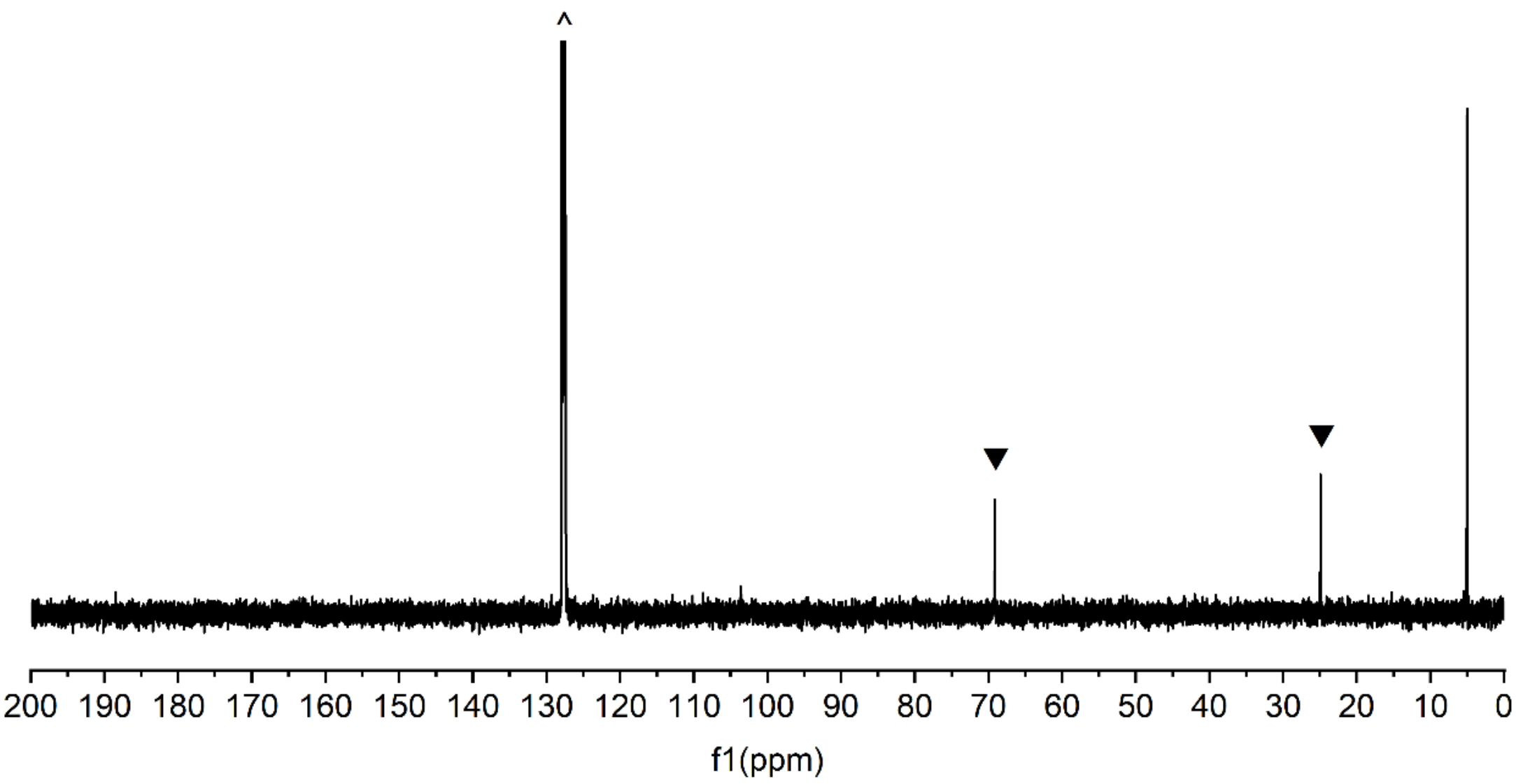

Supplementary Figure 28. ^{13}C NMR (C_6D_6, 298 K) spectrum of [$Li_2$$\{\mu$-$\eta^4$:$\eta^4$-$C_4(SiMe_3)_4\}$$(THF)_2$]. ▼ = coordinated THF; ^ = C_6D_6 solvent.

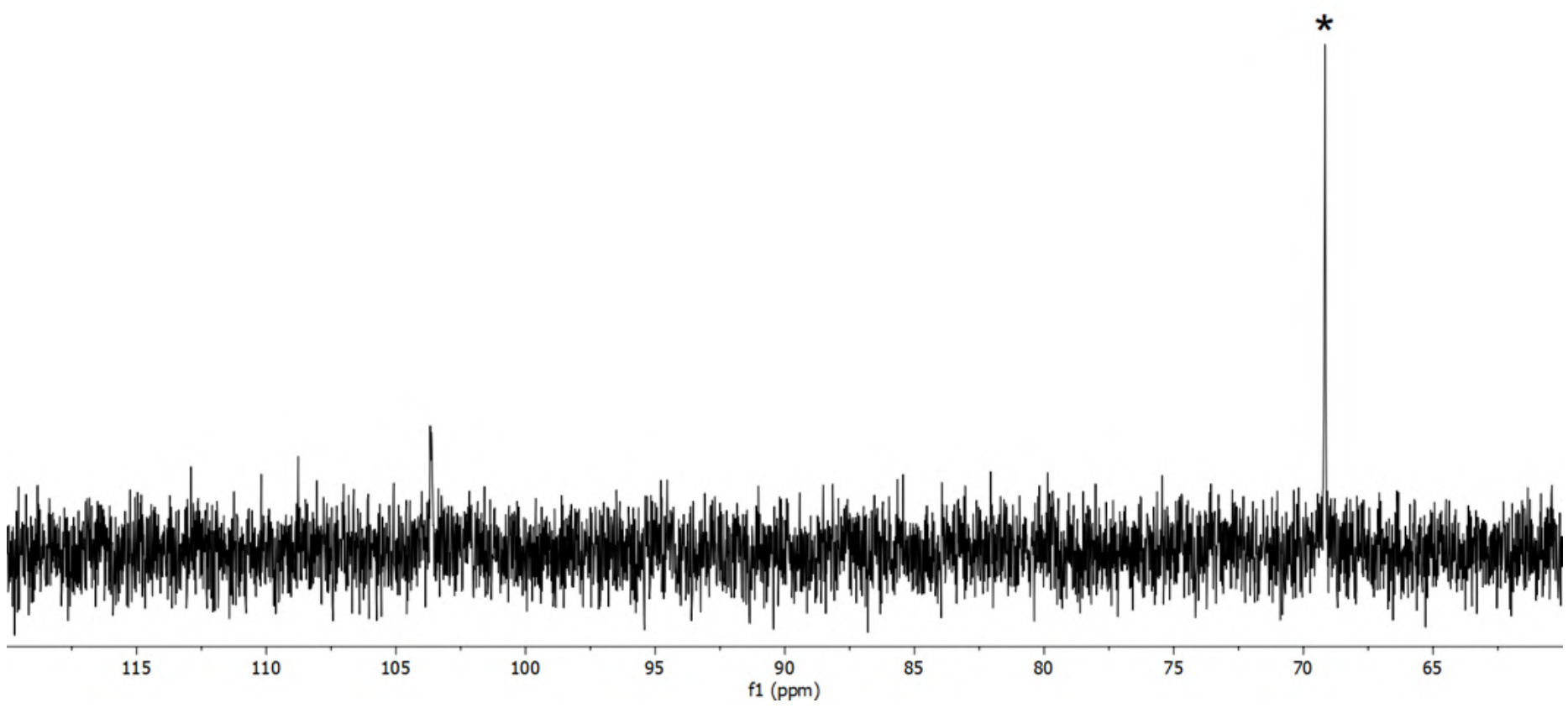

Supplementary Figure 29. Zoom-in of the ^{13}C NMR (D_6-benzene, 298 K) spectrum of $[Li_2\{\mu\text{-}\eta^4\text{:}\eta^4\text{-}C_4(SiMe_3)_4\}(THF)_2]$. * = coordinated THF.

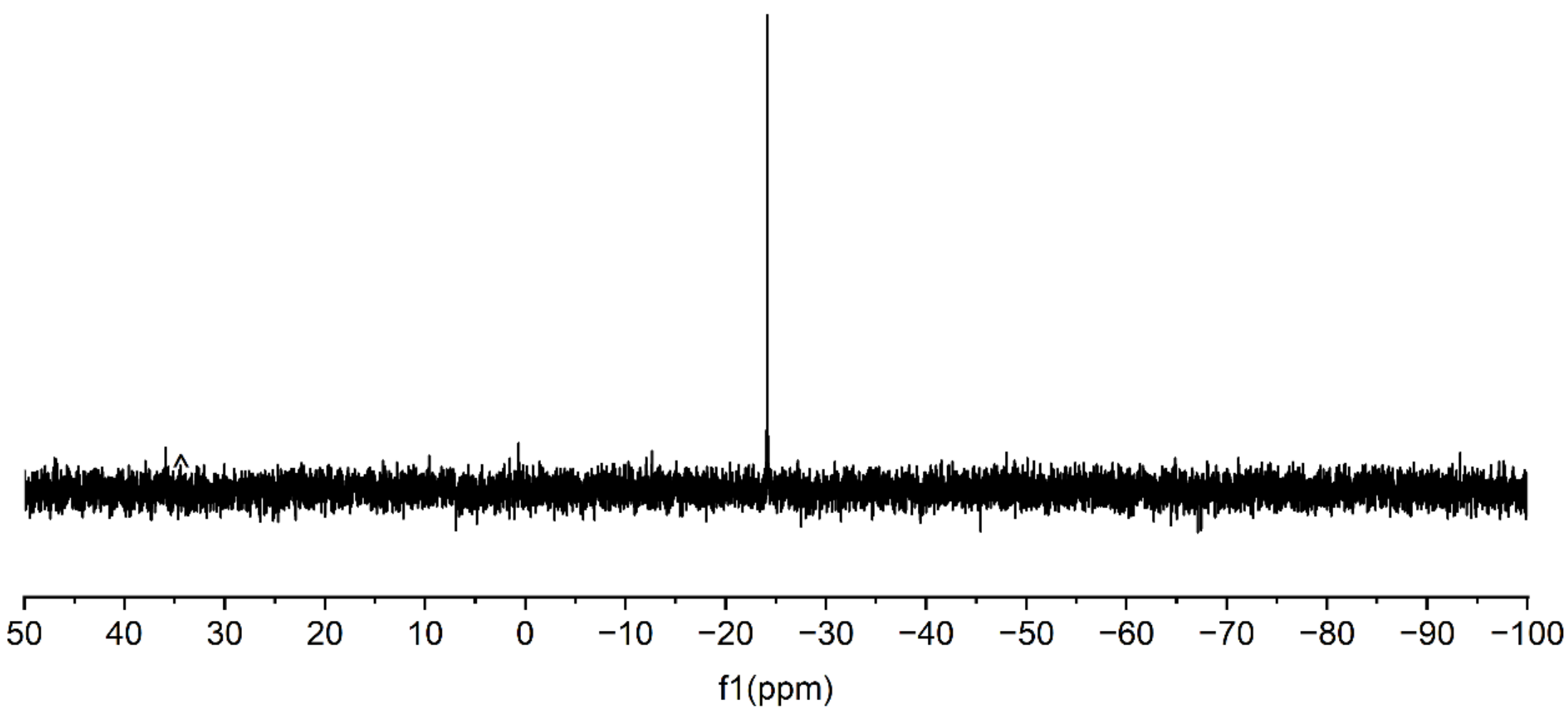

Supplementary Figure 30. ^{29}Si NMR (C_6D_6, 298 K) spectrum of $[Li_2\{\mu\text{-}\eta^4\text{:}\eta^4\text{-}C_4(SiMe_3)_4\}(THF)_2]$.

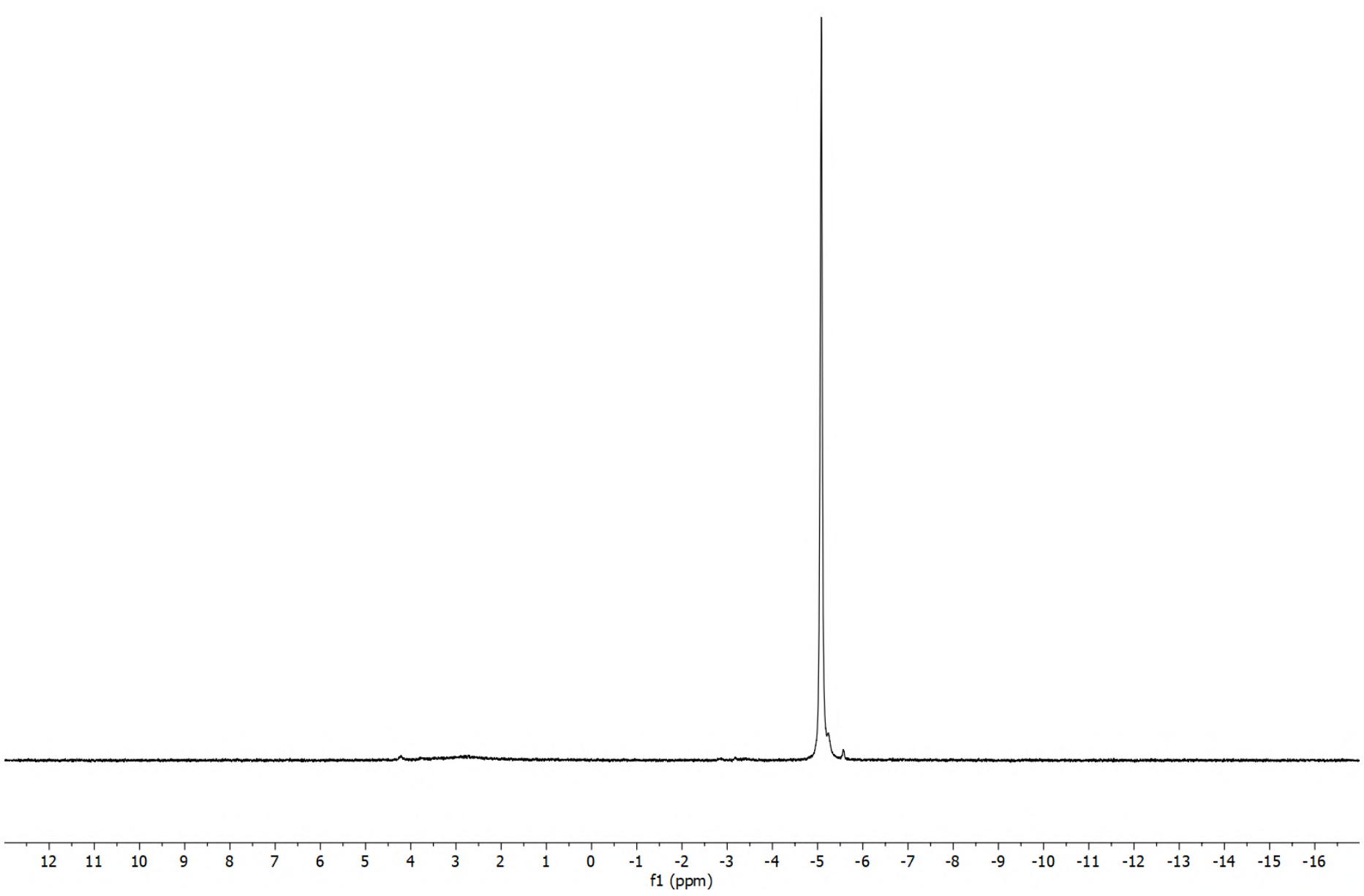

Supplementary Figure 31. ^{7}Li NMR (C_6D_6, 298 K) spectrum of $[Li_2\{\mu\text{-}\eta^4\text{:}\eta^4\text{-}C_4(SiMe_3)_4\}(THF)_2]$.

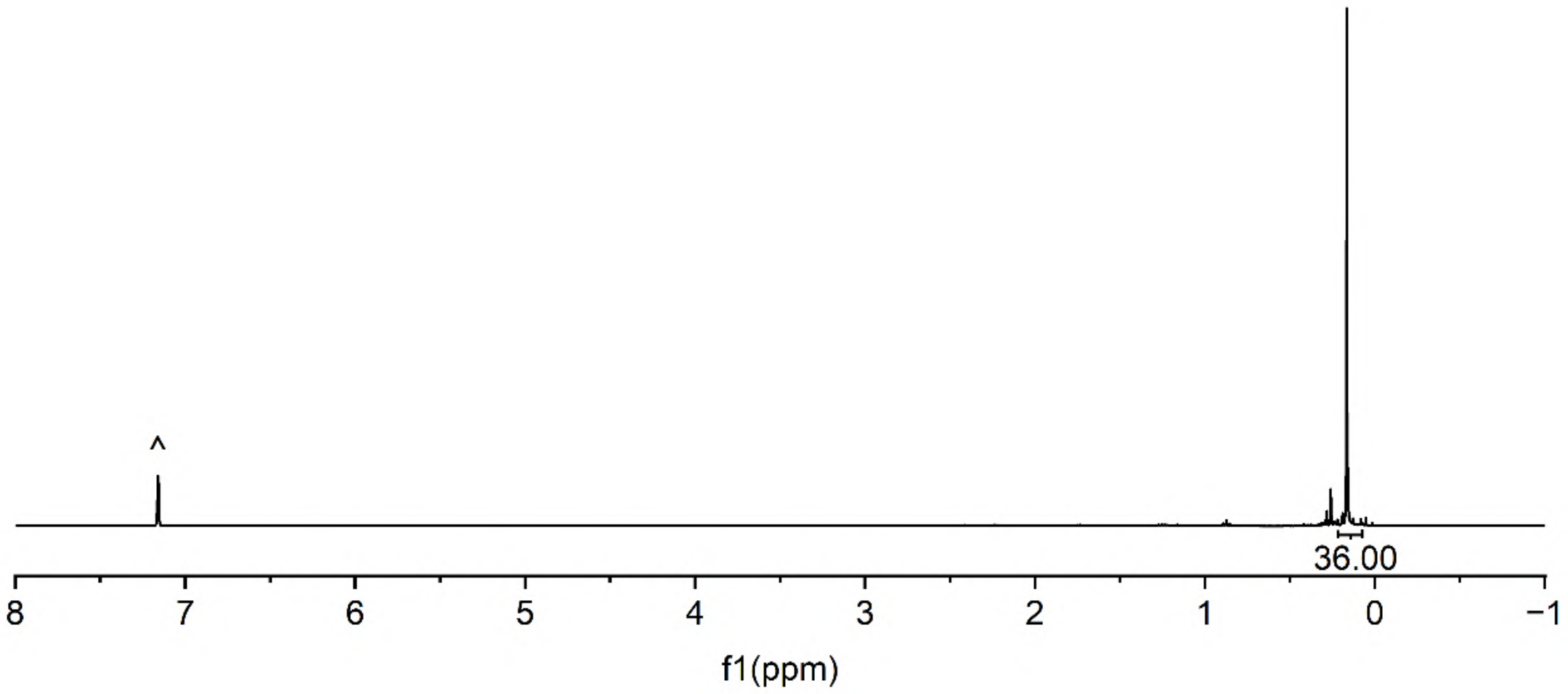

Supplementary Figure 32. ^{1}H NMR (C_6D_6, 298 K) spectrum of [$C_4(SiMe_3)_4$]. ^ = C_6D_6 solvent.

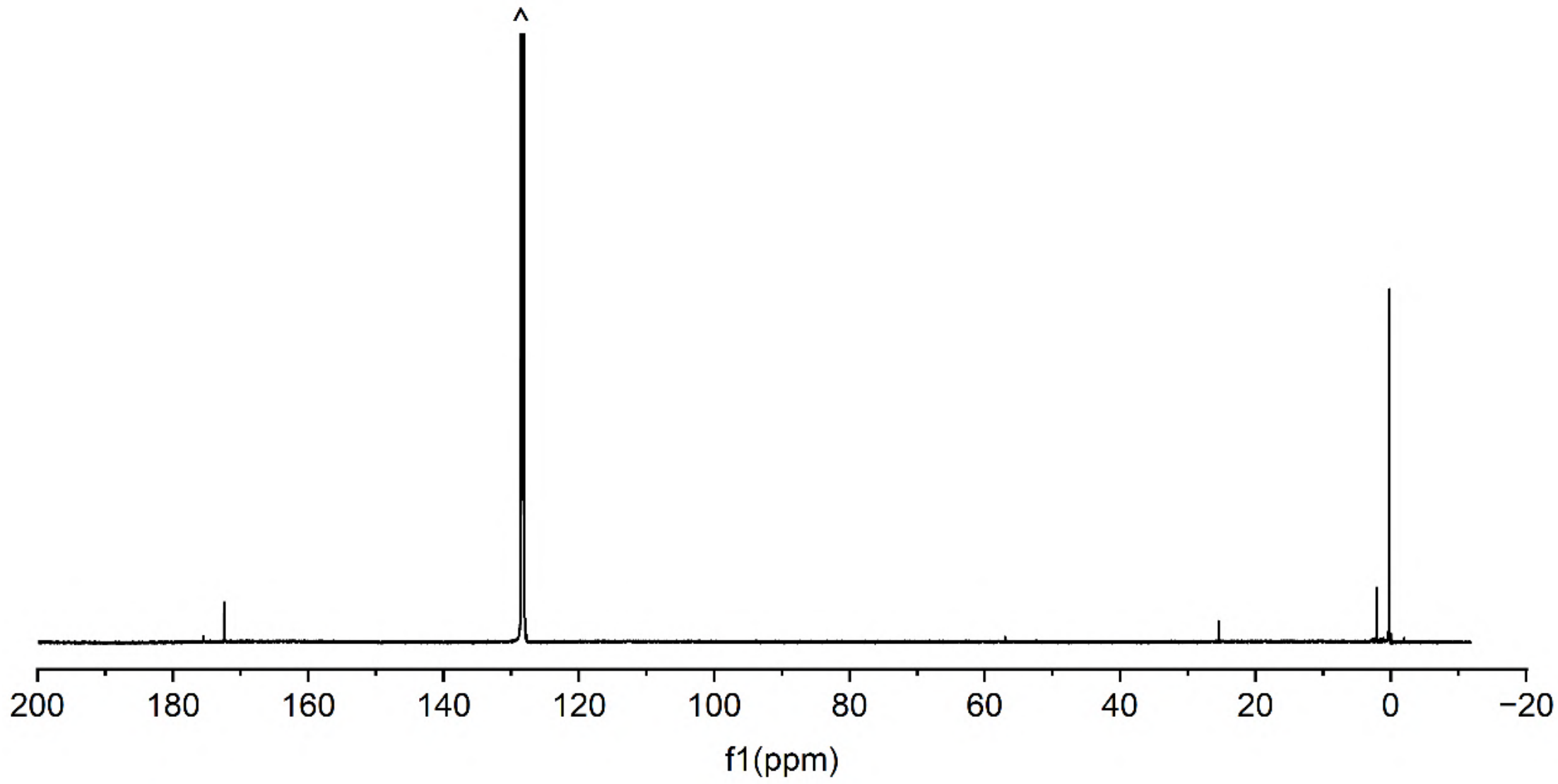

Supplementary Figure 33. ^{13}C NMR (C_6D_6, 298 K) spectrum of [$C_4(SiMe_3)_4$]. ^ = C_6D_6 solvent.

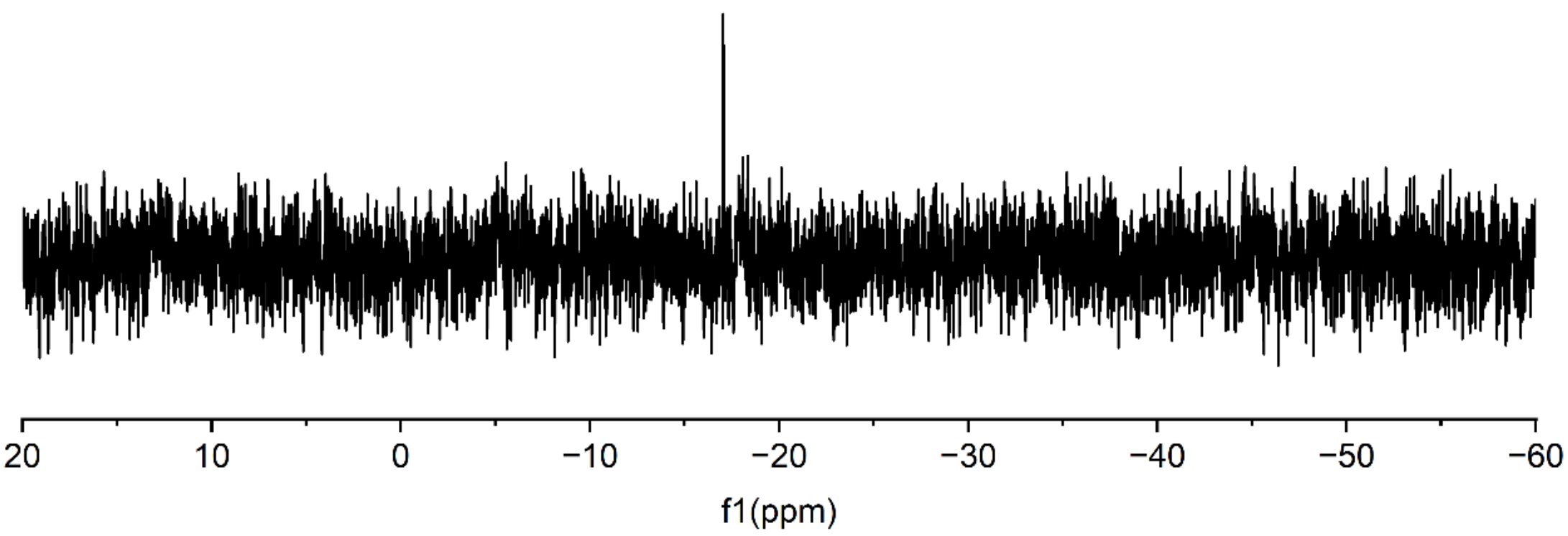

Supplementary Figure 34. $^{29}Si\{^{1}H\}$ NMR (C_6D_6, 298 K) spectrum of [$C_4(SiMe_3)_4$]. ^ = C_6D_6 solvent.

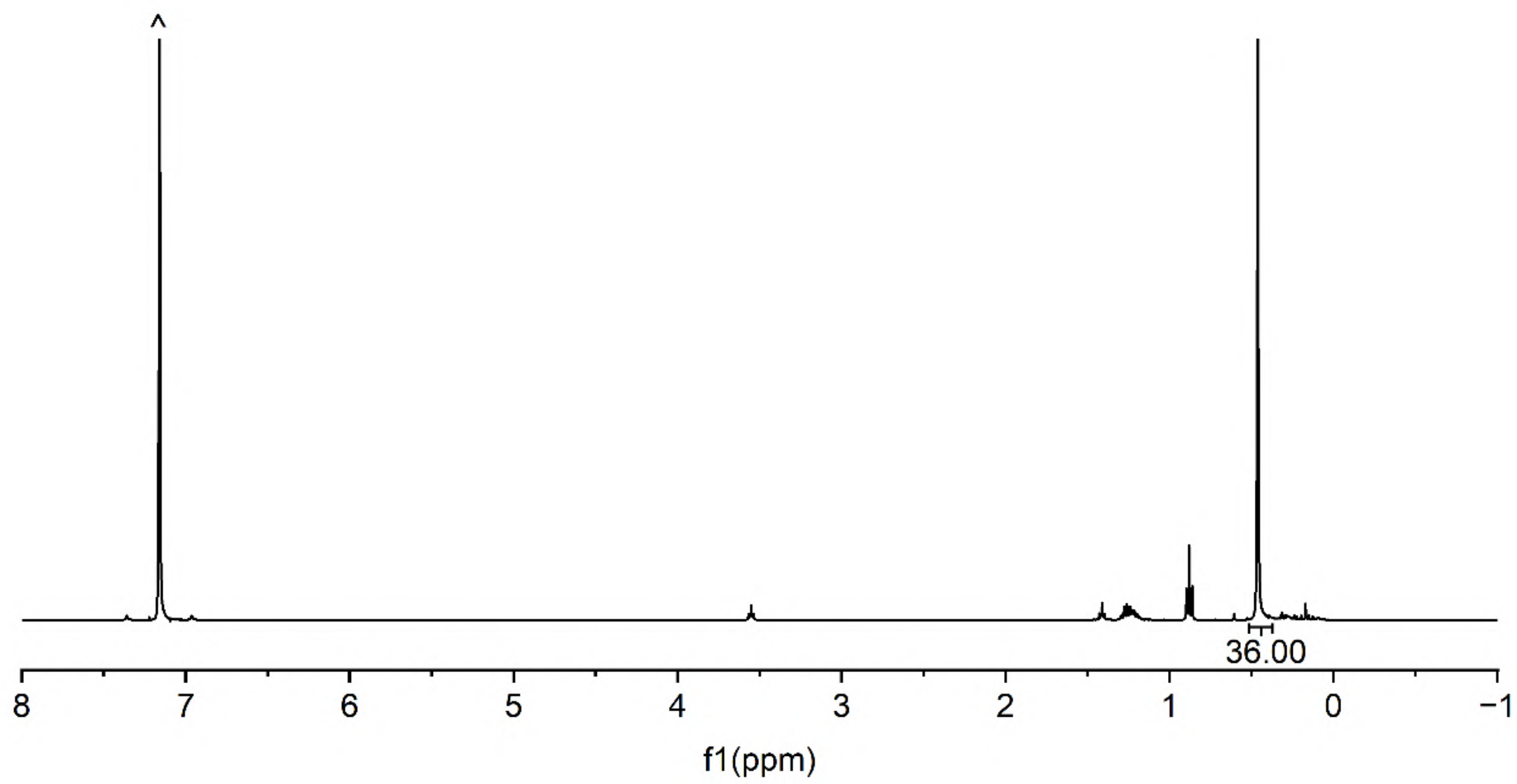

Supplementary Figure 35. ^{1}H NMR (C_6D_6, 298 K) spectrum of **2**. ▼ = coordinated THF; ^ = C_6D_6 solvent.

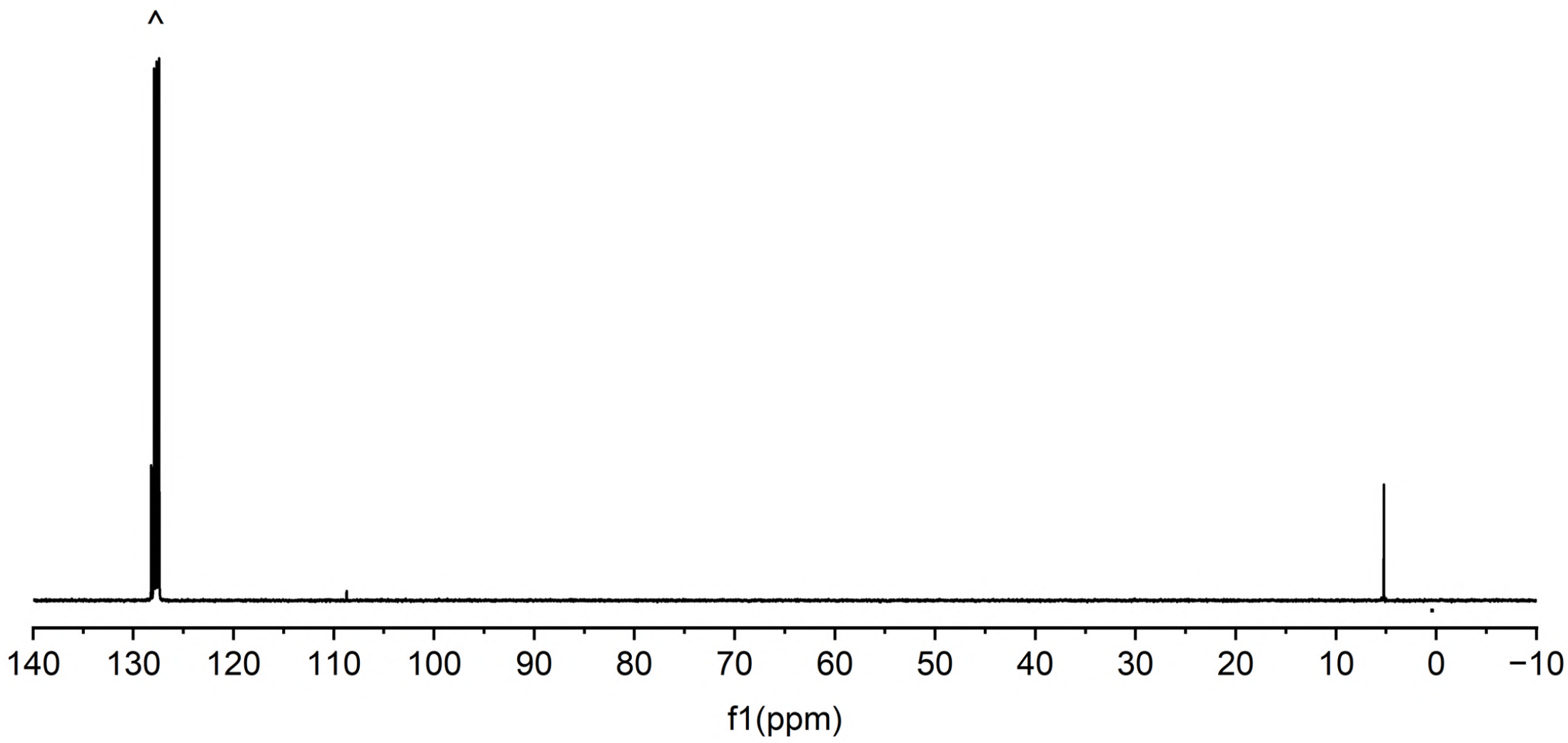

Supplementary Figure 36. $^{13}C\{^{1}H\}$ NMR (C_6D_6, 298 K) spectrum of **2**. ^ = C_6D_6 solvent.

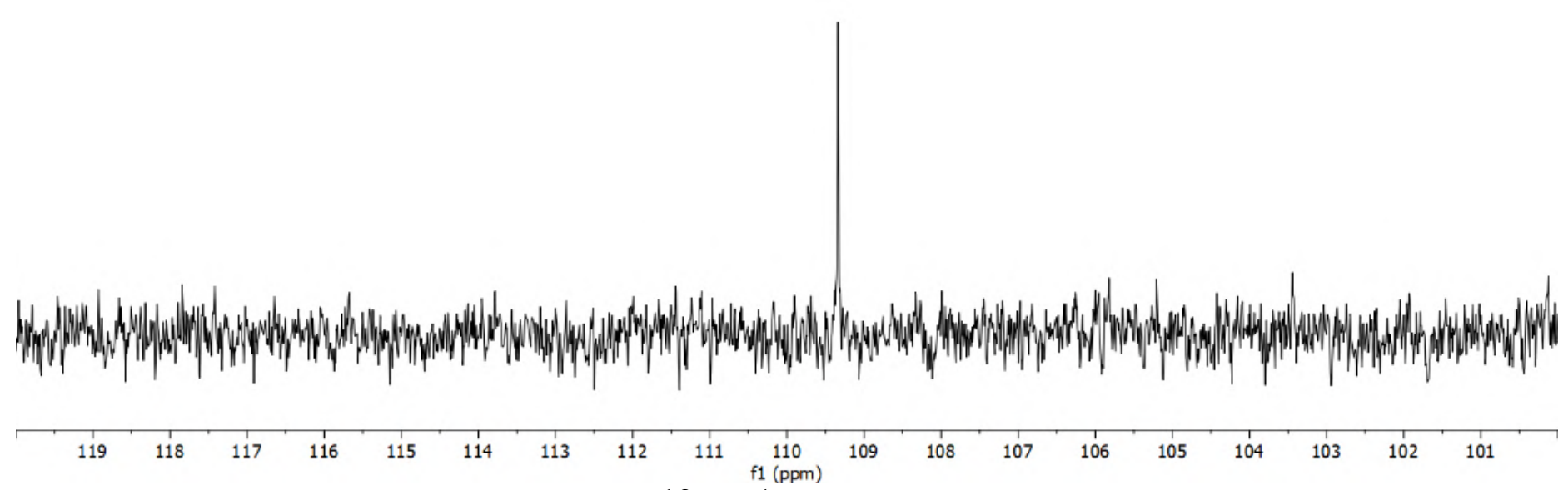

Supplementary Figure 37. Zoom-in of the $^{13}C\{^{1}H\}$ NMR (C_6D_6, 298 K) spectrum of **2**.

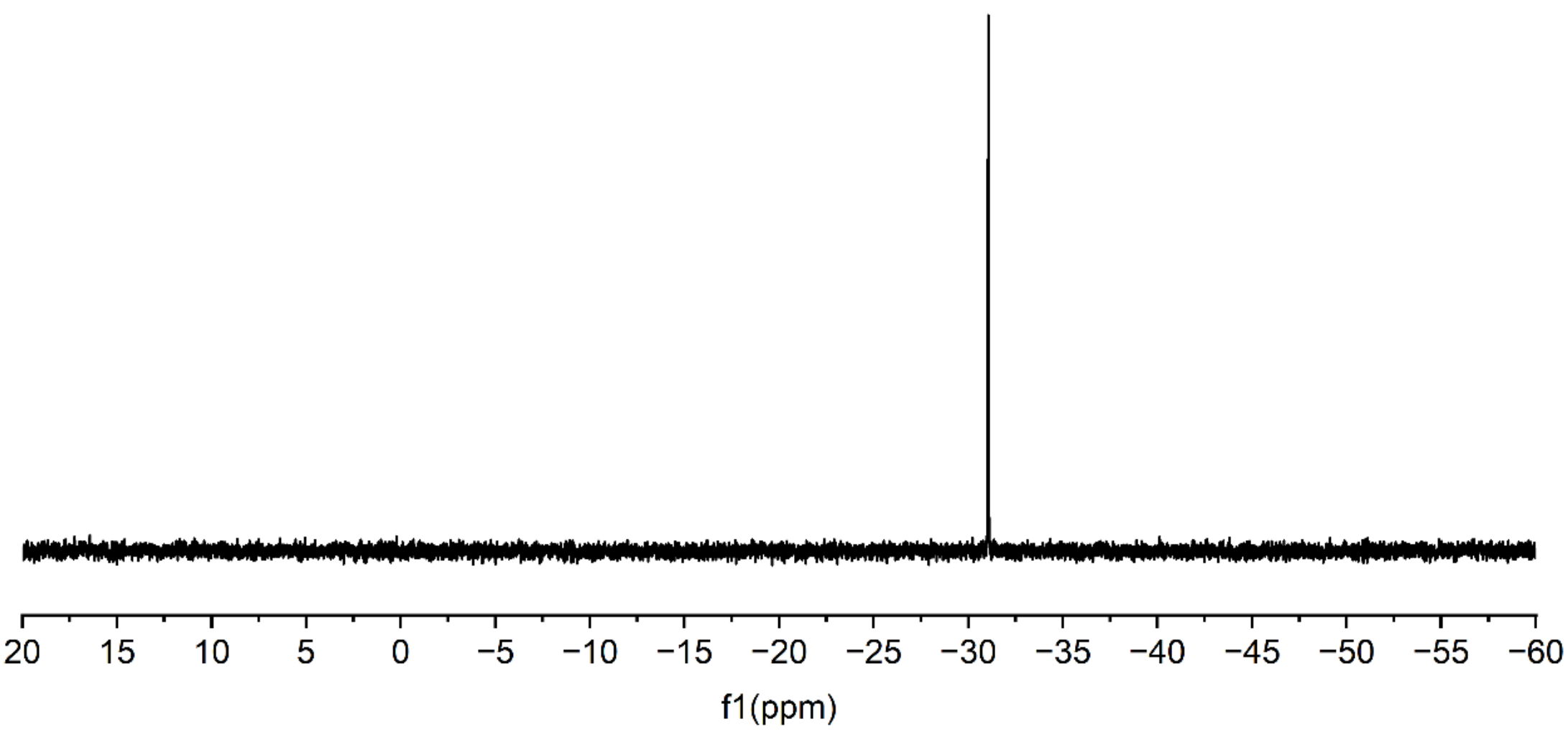

Supplementary Figure 38. $^{29}Si\{^1H\}$ NMR (C_6D_6, 298 K) spectrum of **2**.

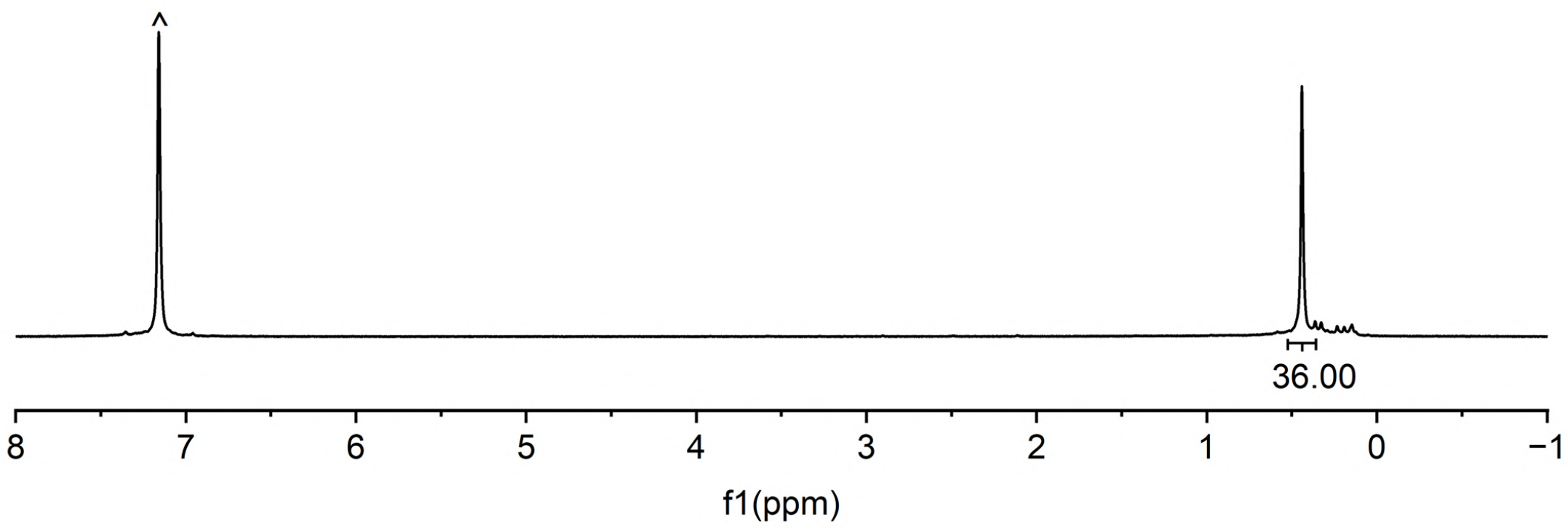

Supplementary Figure 39. ^1H NMR (C_6D_6, 298 K) spectrum of **3**. ^ = C_6D_6 solvent.

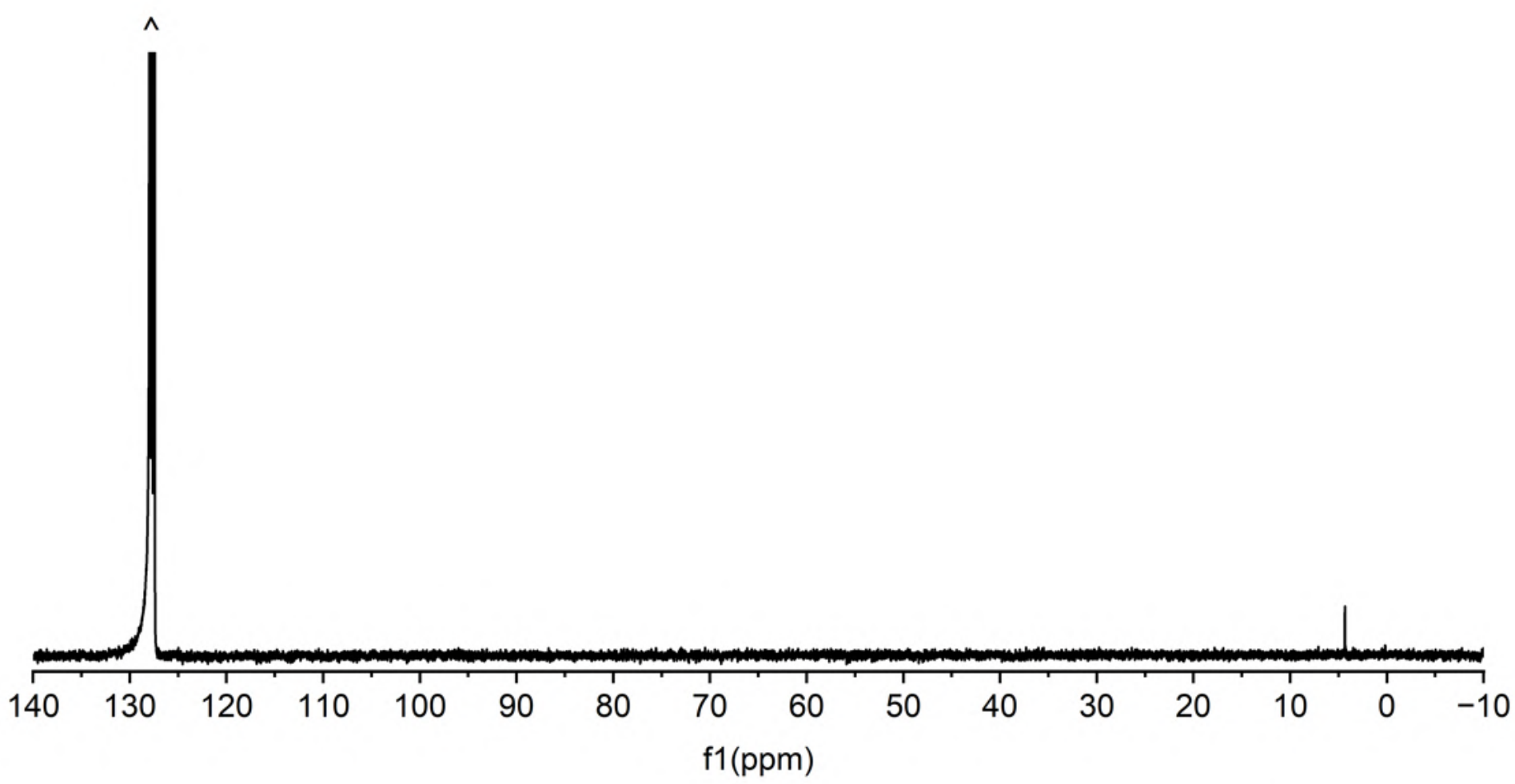

Supplementary Figure 40. $^{13}C\{^1H\}$ NMR (C_6D_6, 298 K) spectrum of **3**. ^ = C_6D_6 solvent.

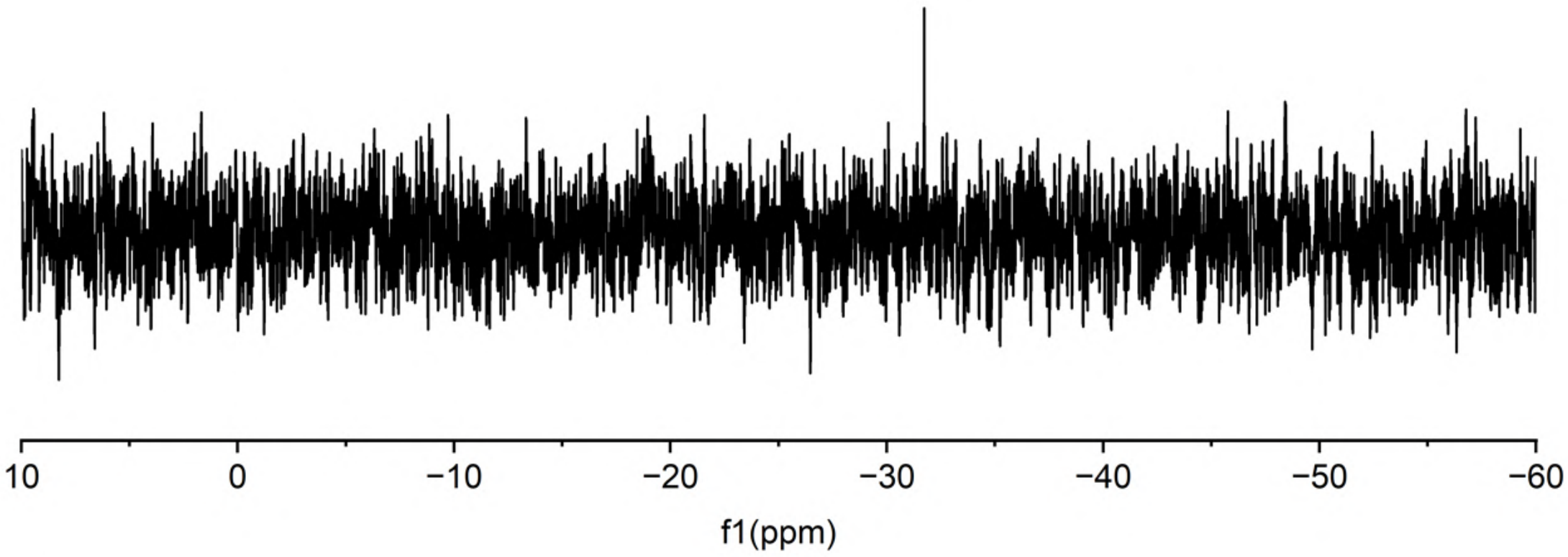

Supplementary Figure 41. $^{29}Si\{^1H\}$ NMR (C_6D_6, 298 K) spectrum of **3**.

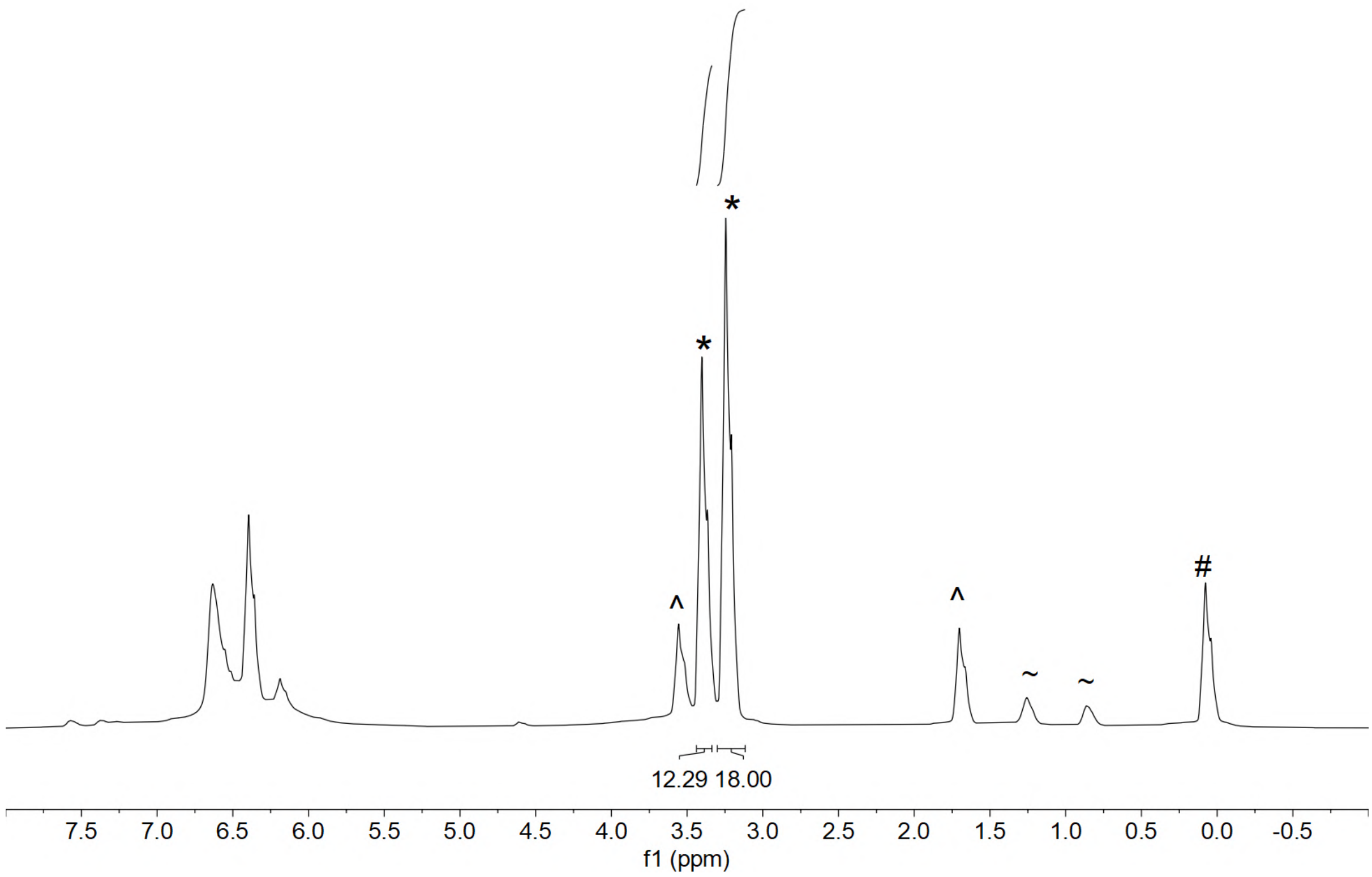

Supplementary Figure 42. ^1H NMR (D_8-THF, 298 K) spectrum of **4K**. * = coordinated DME; # = silicone grease; ^ = D_8-THF; ~ = residual pentane.

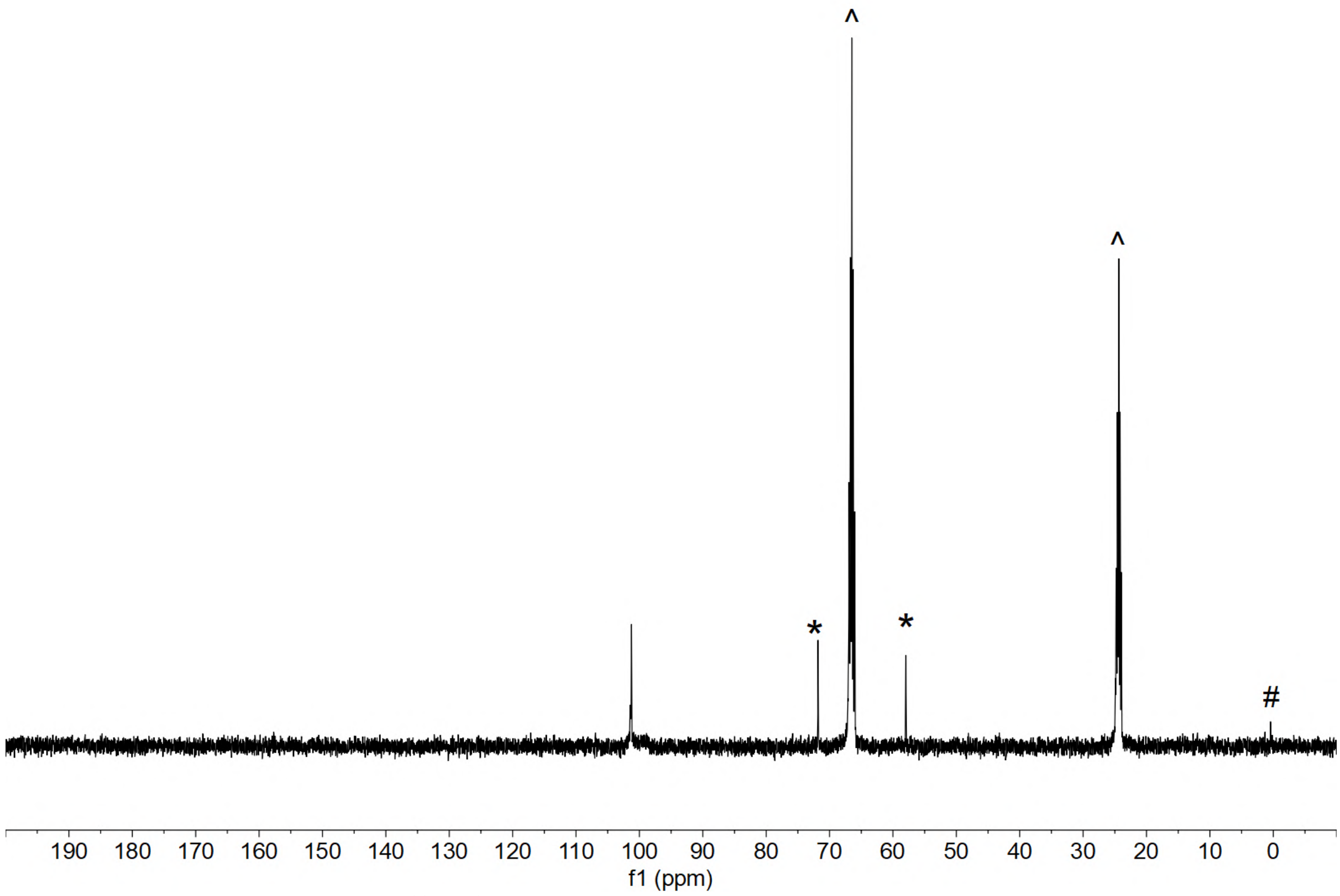

Supplementary Figure 43. $^{13}C\{^{1}H\}$ NMR (D_8-THF, 298 K) spectrum of **4K**. * = coordinated DME; ^ = D_8-THF; # = silicone grease.

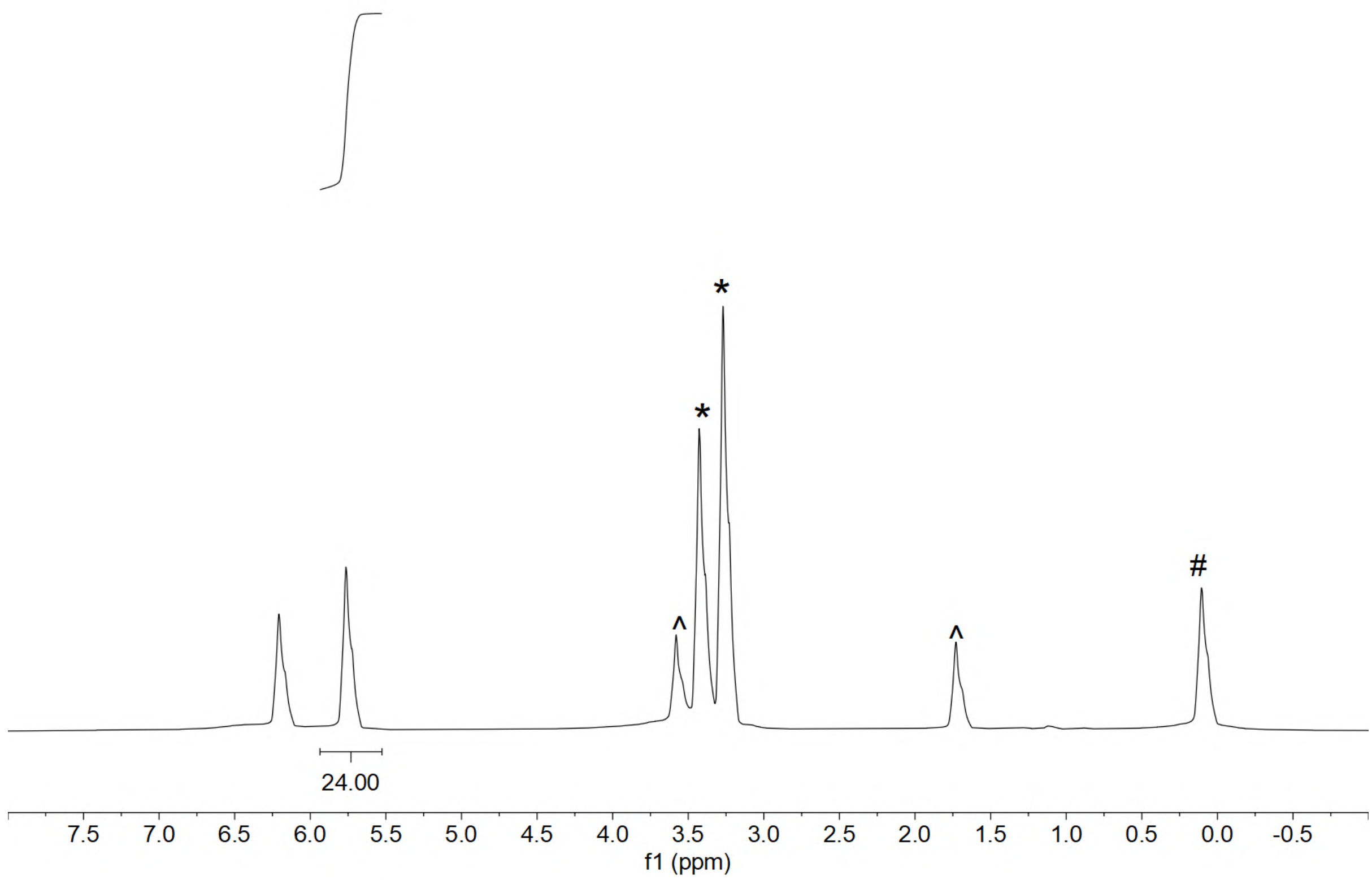

Supplementary Figure 44. ^{1}H NMR (D_8-THF, 298 K) spectrum of **5K**. * = coordinated DME; # = silicone grease; ^ = D_8-THF.

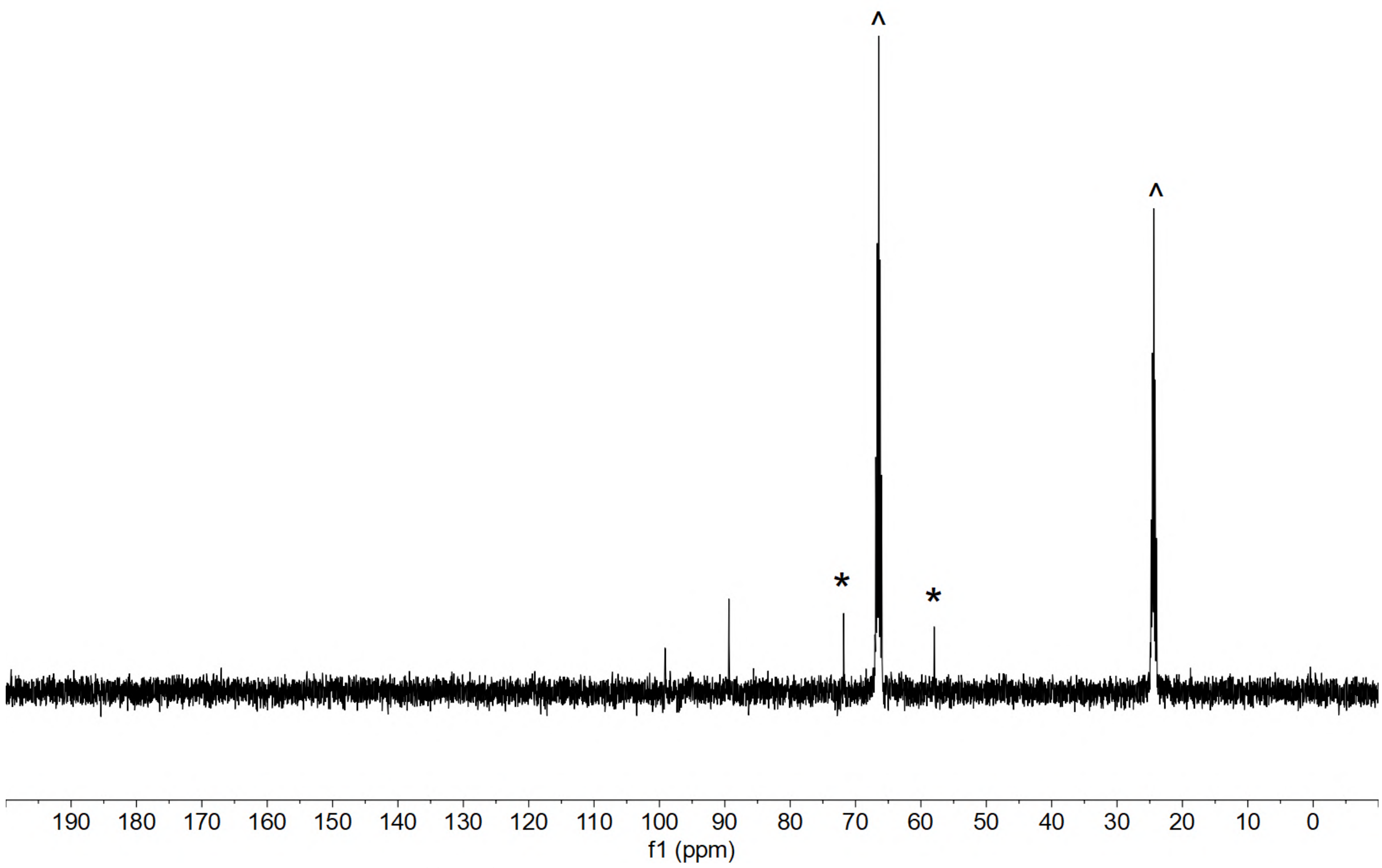

Supplementary Figure 45. $^{13}C\{^{1}H\}$ NMR (D_8-THF, 298 K) spectrum of **5K**. * = coordinated DME; ^ = D_8-THF.

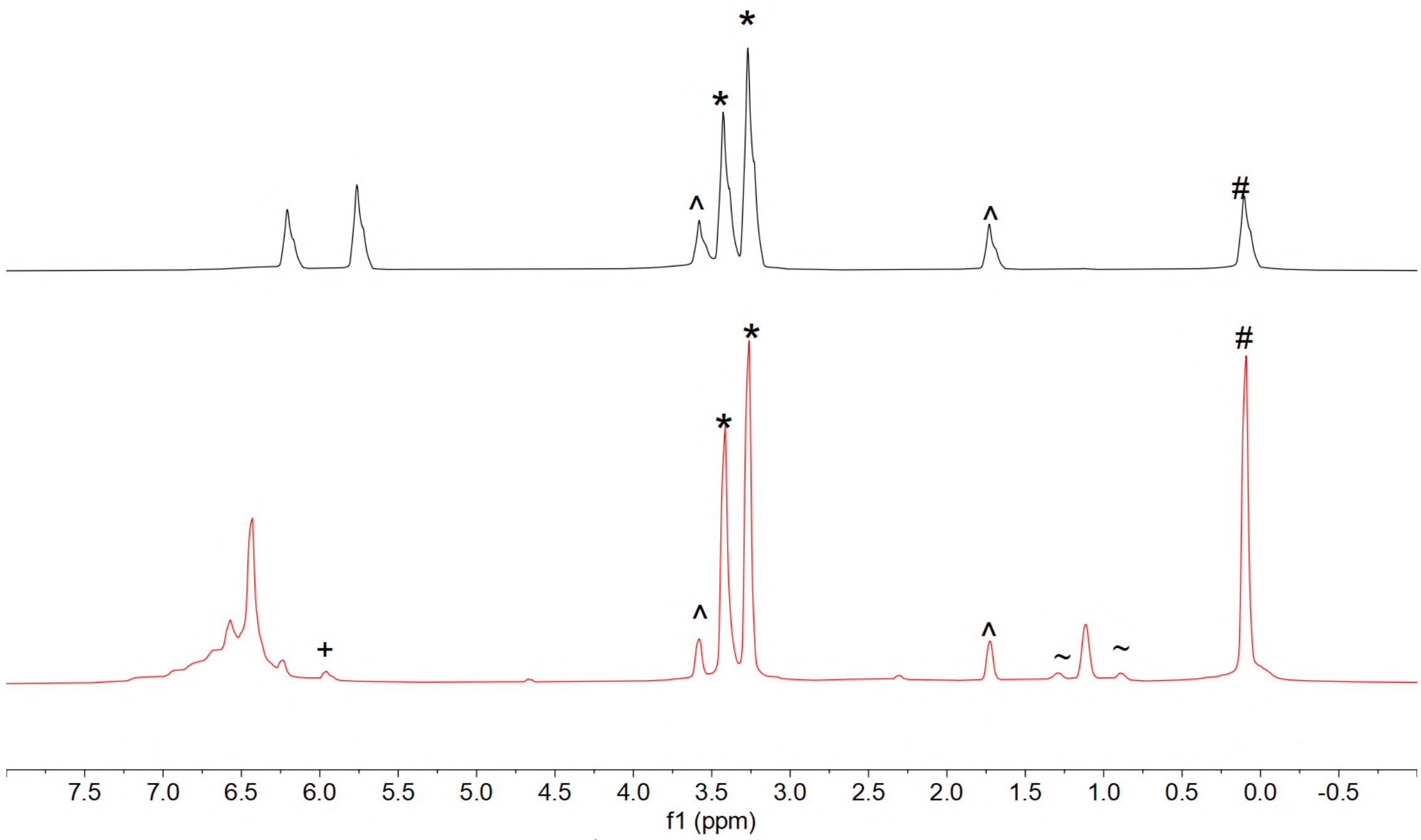

Supplementary Figure 46. Comparative ^{1}H NMR (D_6-benzene, 298 K) spectra of **5K** (top, black) and the same sample of **5K** but intentionally degraded through excessive heating (bottom, red). This excessive heating causes a colour change from dark blue to light brown. Upon degradation, there is the loss of the assigned C_8H_8 resonance for **5K** at ~5.77 ppm and resonances related to $[Th(\eta^8\text{-}C_8H_8)_2]$ and free 1,3,5,7-cyclooctatetraene at ~6.50 and ~5.70 ppm, respectively, which are common decomposition products, could now be observed. * = coordinated DME; ^ = THF solvent; ~ = residual pentane; + = trace C_8H_8; # = silicone grease.

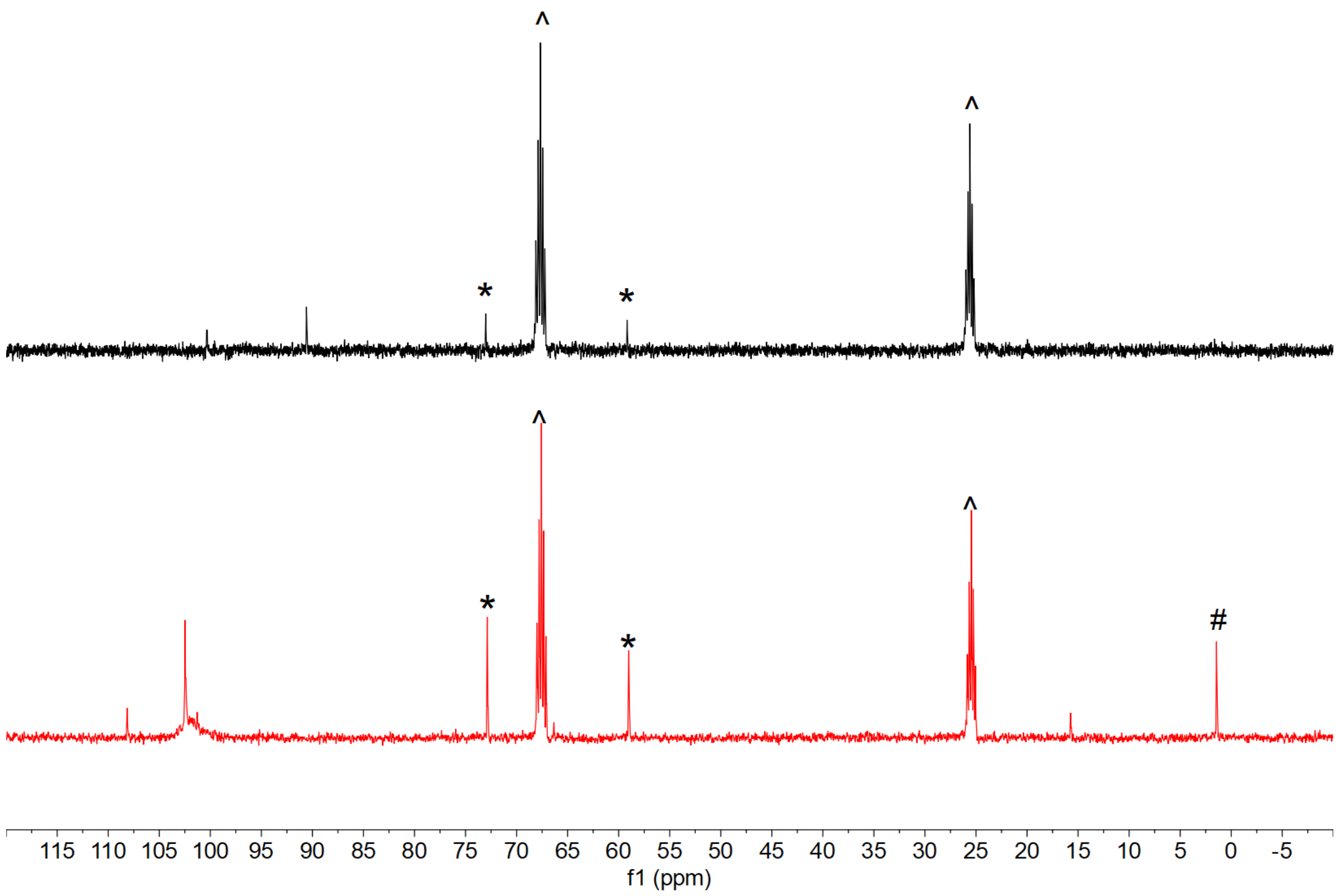

Supplementary Figure 47. Comparative ^{13}C{^{1}H} NMR (D_6-benzene, 298 K) spectra of **5K** (top, black) and the same sample of **5K** but intentionally degraded through excessive heating (bottom, red). This excessive heating causes a colour change from dark blue to light brown. Upon degradation, there is the loss of the assigned C_8H_8 resonance for **5K** at ~89 ppm and the resonance related to [Th(η^8-C_8H_8)$_2$] at ~101 ppm, which is a common decomposition product, could now be observed. This is discussed further in the supplementary text. * = coordinated DME; ^ = THF solvent; ~ = residual pentane; + = trace C_8H_8; # = silicone grease.

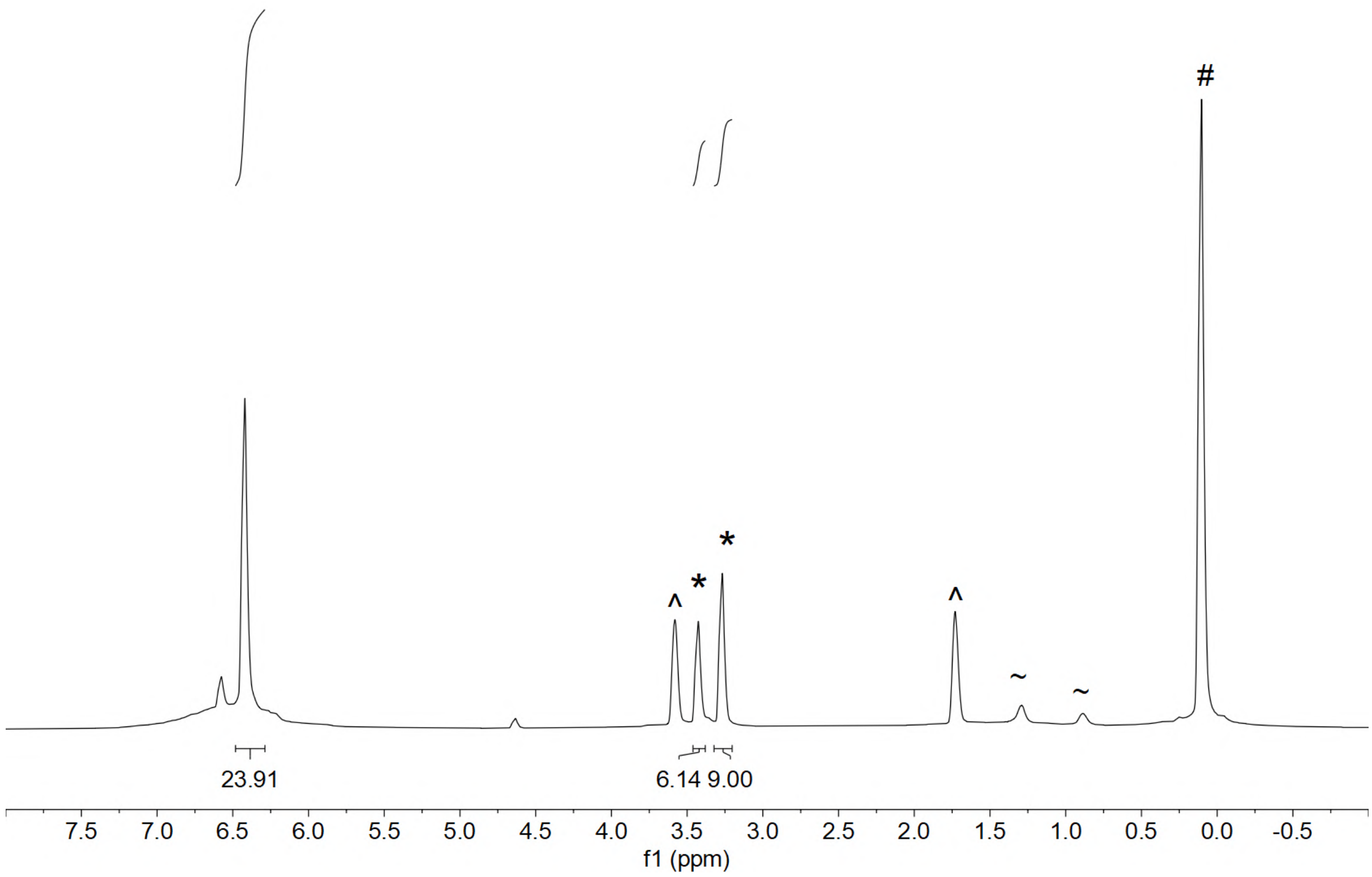

Supplementary Figure 48. ^{1}H NMR (D_8-THF, 298 K) spectrum of **6**. * = coordinated DME; # = silicone grease; ^ = D_8-THF; ~ = residual pentane.

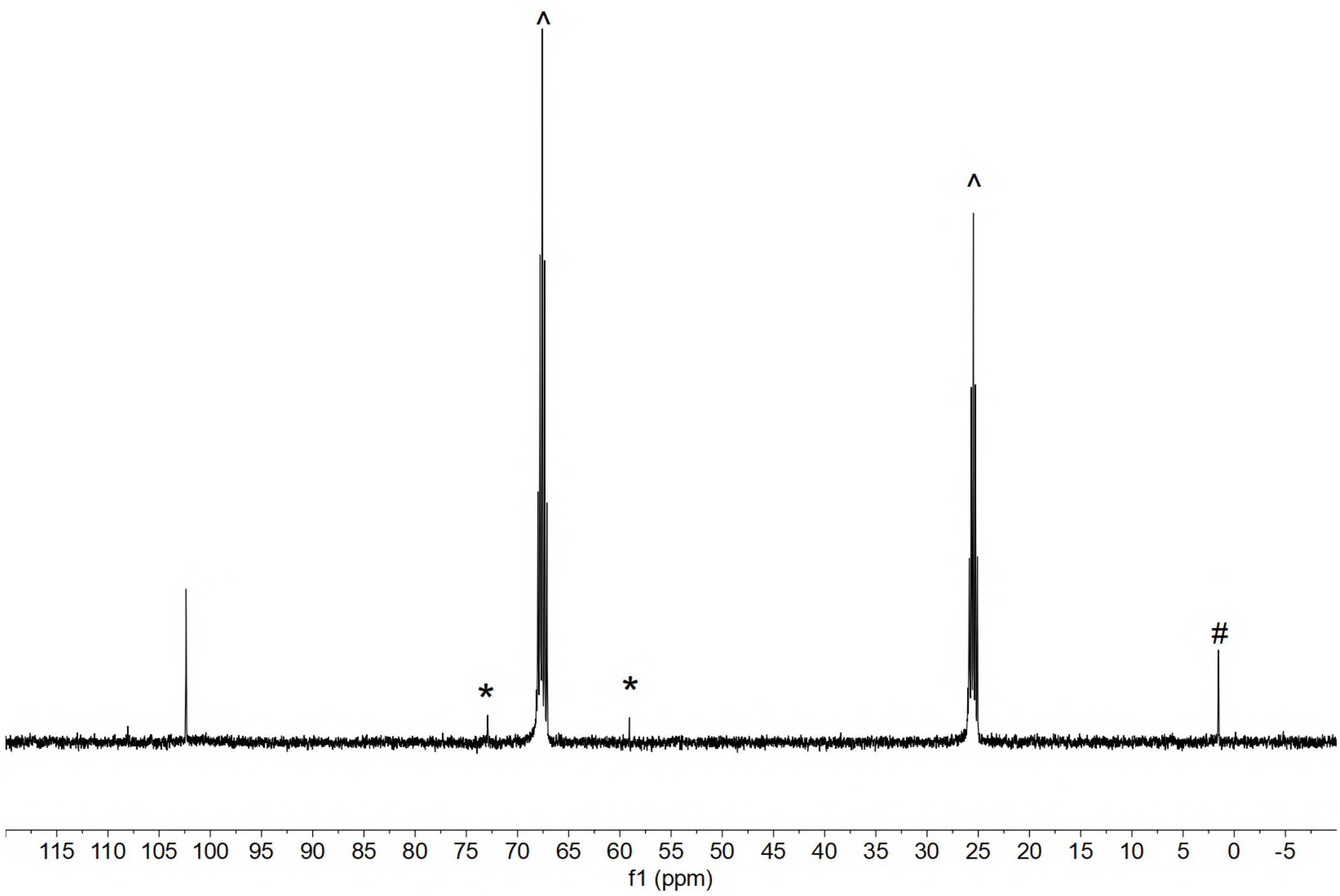

Supplementary Figure 49. $^{13}C\{^1H\}$ NMR (D_8-THF, 298 K) spectrum of **6**. * = coordinated DME; ^ = D_8-THF; # = silicone grease.

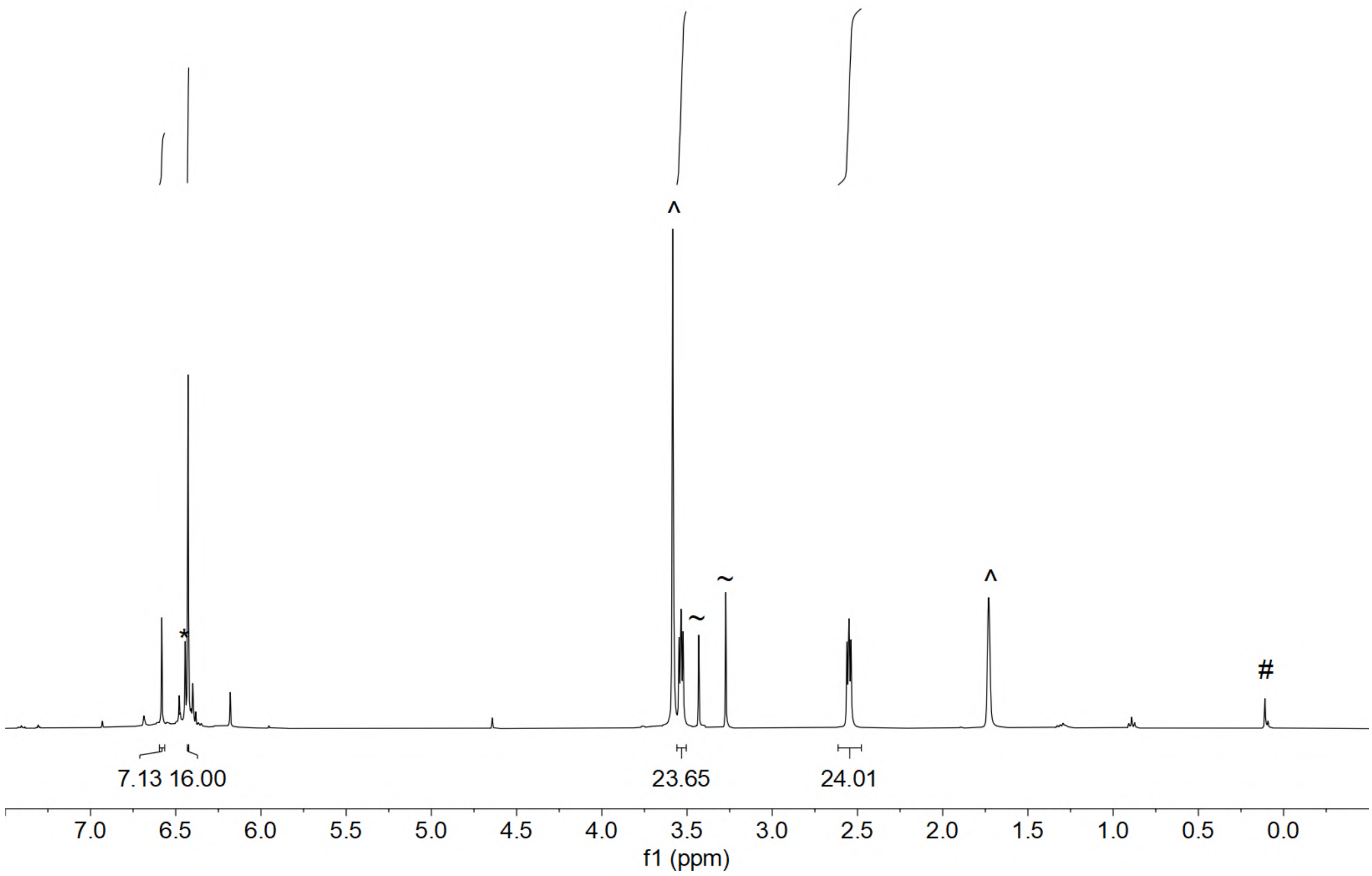

Supplementary Figure 50. ^1H NMR (D_8-THF, 298 K) spectrum of **7**. * = residual [Th(η^8-C_8H_8)$_2$]; # = silicone grease; ^ = D_8-THF; ~ = residual DME.

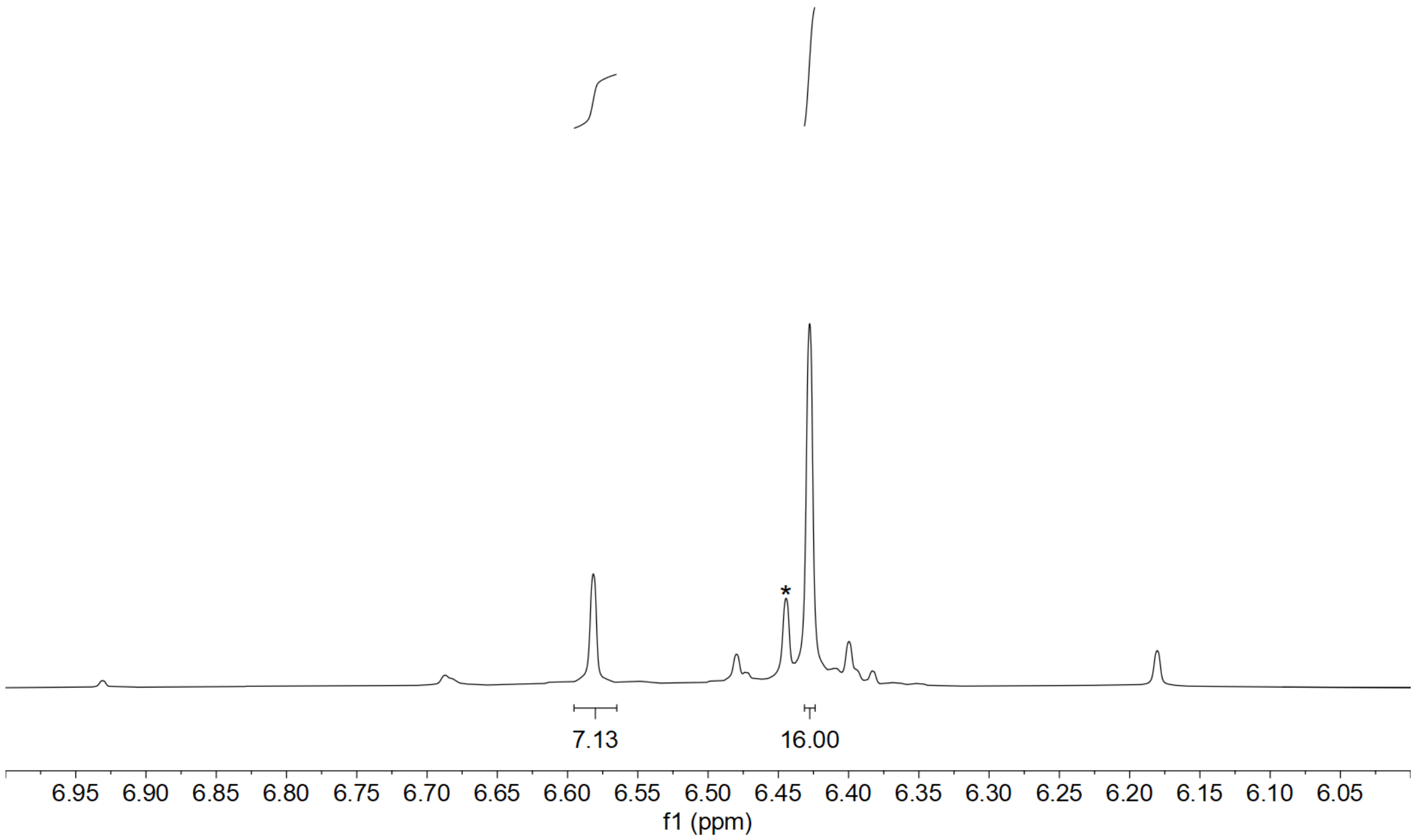

Supplementary Figure 51. Zoom-in of the ^{1}H NMR (D_8-THF, 298 K) spectrum of **7** to highlight the two C_8H_8 environments in solution. * = residual [Th(η^8-C_8H_8)$_2$].

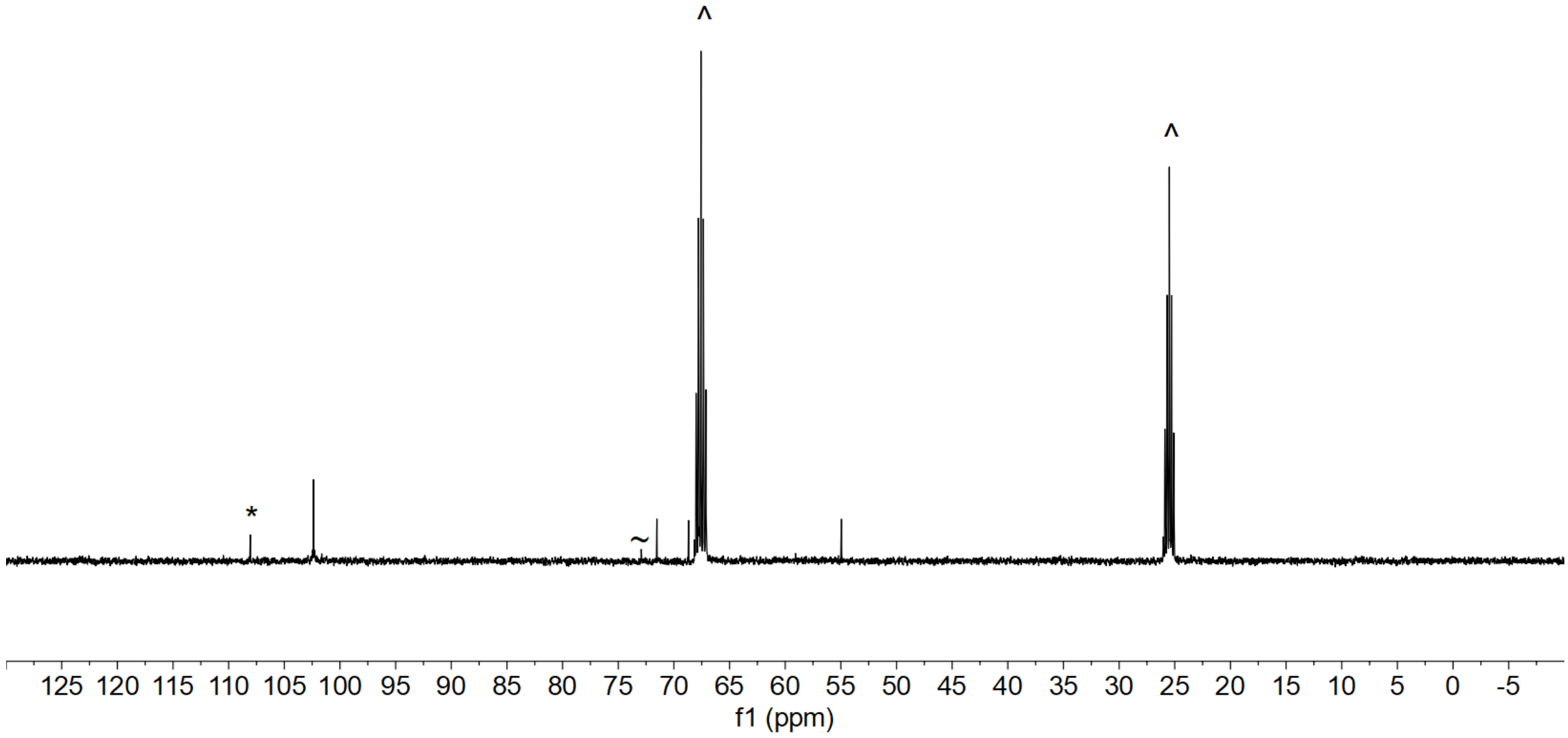

Supplementary Figure 52. $^{13}C\{^{1}H\}$ NMR (D_8-THF, 298 K) spectrum of **7**. * = residual [Th(η^8-C_8H_8)$_2$]; ^ = D_8-THF; ~ = residual DME.

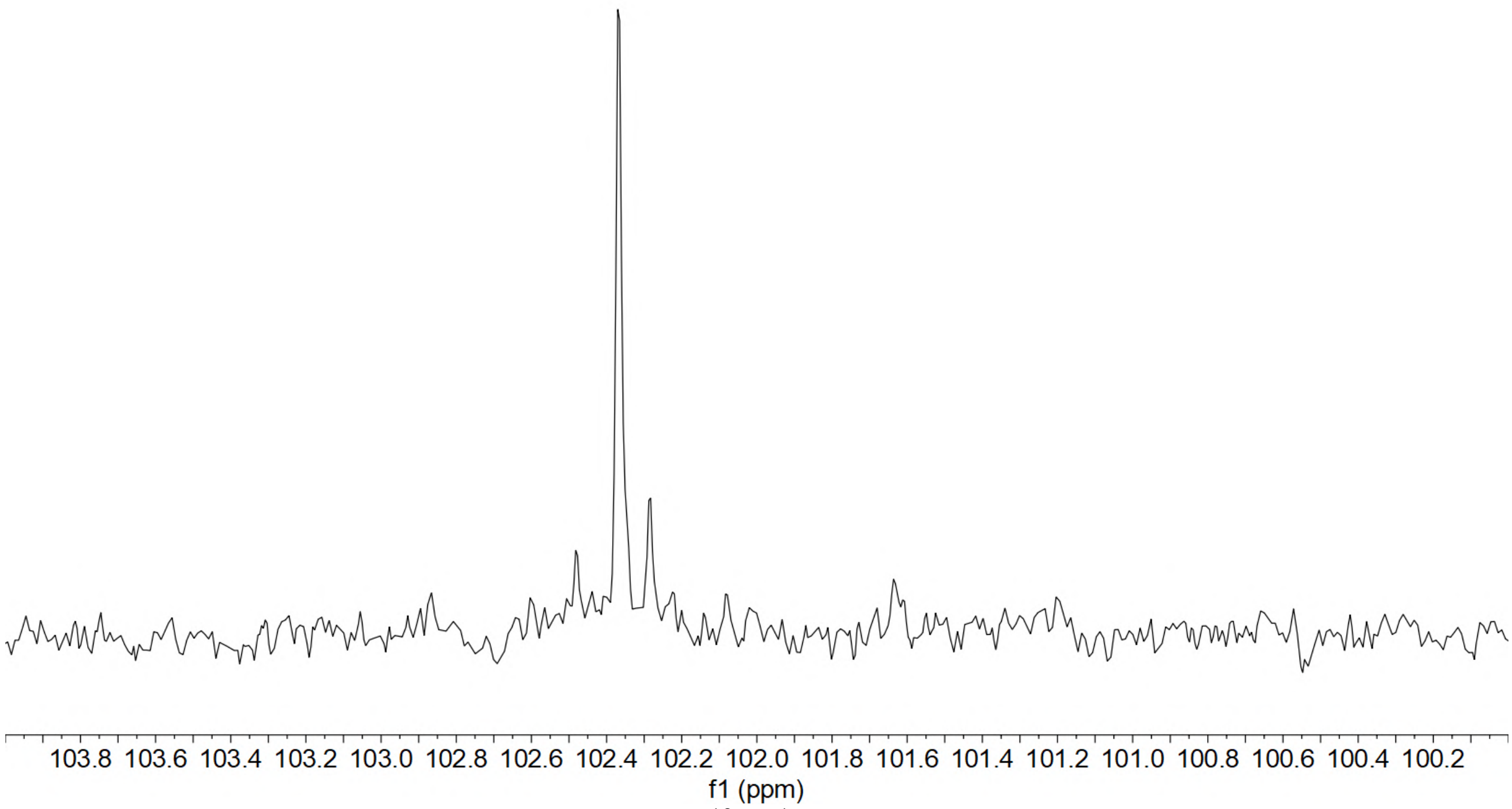

Supplementary Figure 53. Zoom-in of the $^{13}C\{^1H\}$ NMR (D_8-THF, 298 K) spectrum of **7** to highlight the two C_8H_8 environments in solution.

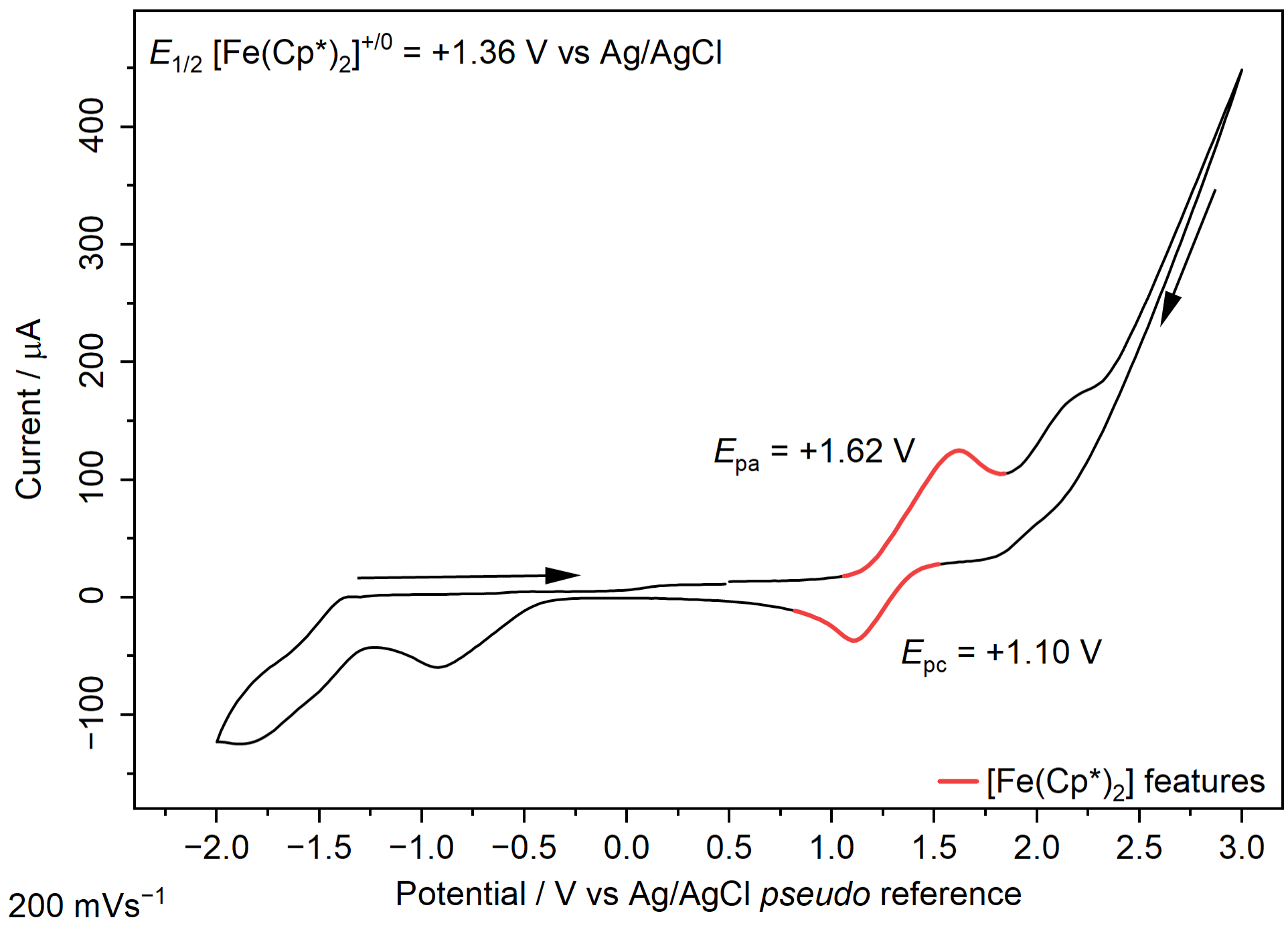

Supplementary Figure 54. Cyclic voltammogram of **4K'** (5.0 mM) with [Fe(η^5-C_5Me_5)$_2$] internal reference (ca. 3 mg) in THF supported by [Bu^n_4N][BPh_4] (50 mM) versus Ag/AgCl pseudo reference at 200 mV s^{-1}.

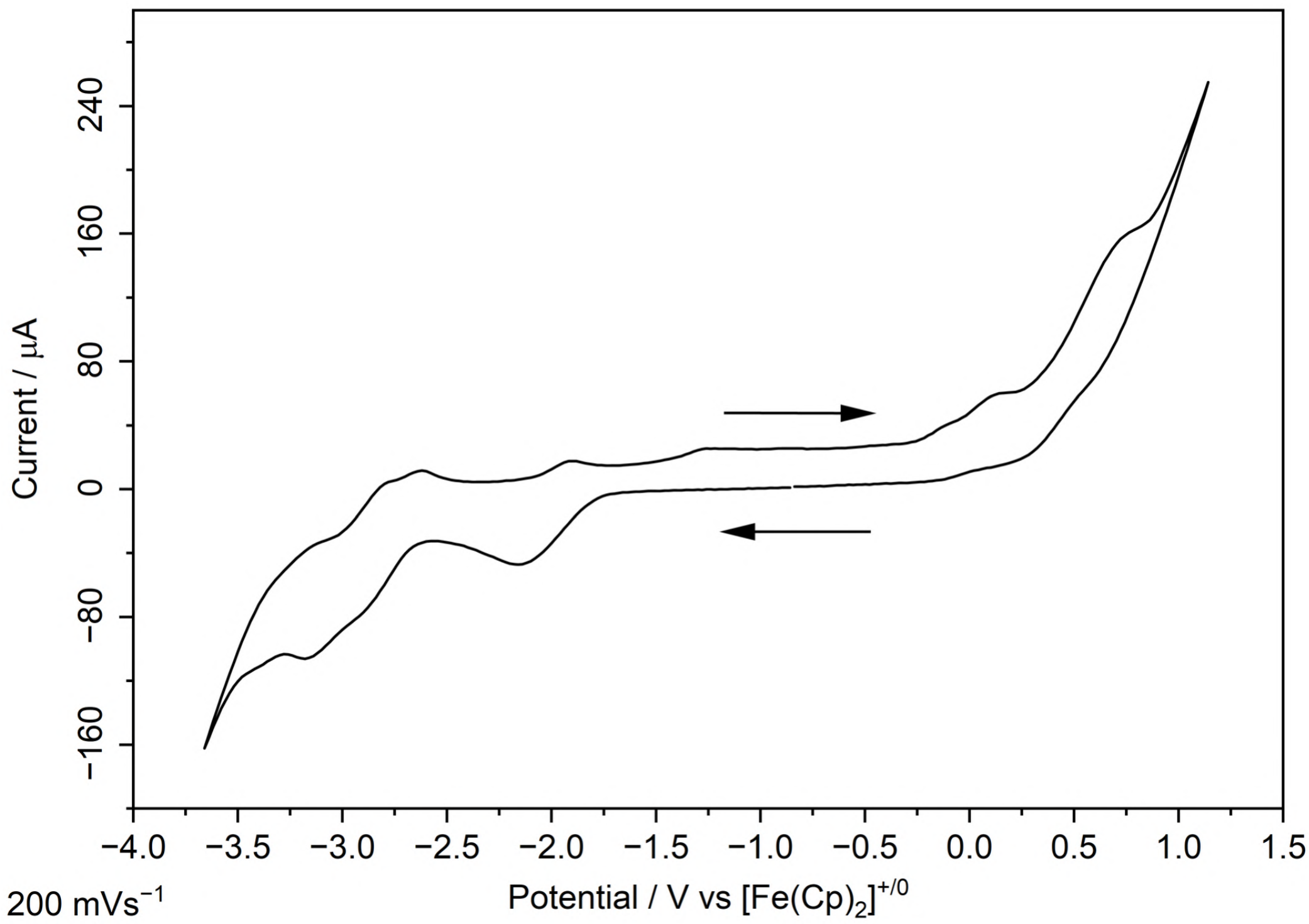

Supplementary Figure 55. Cyclic voltammogram of **4K'** (5.0 mM) in THF supported by $[Bu^n_4N][BPh_4]$ (50 mM) versus the $Fc^{+/0}$ couple at 200 mV s^{-1}.

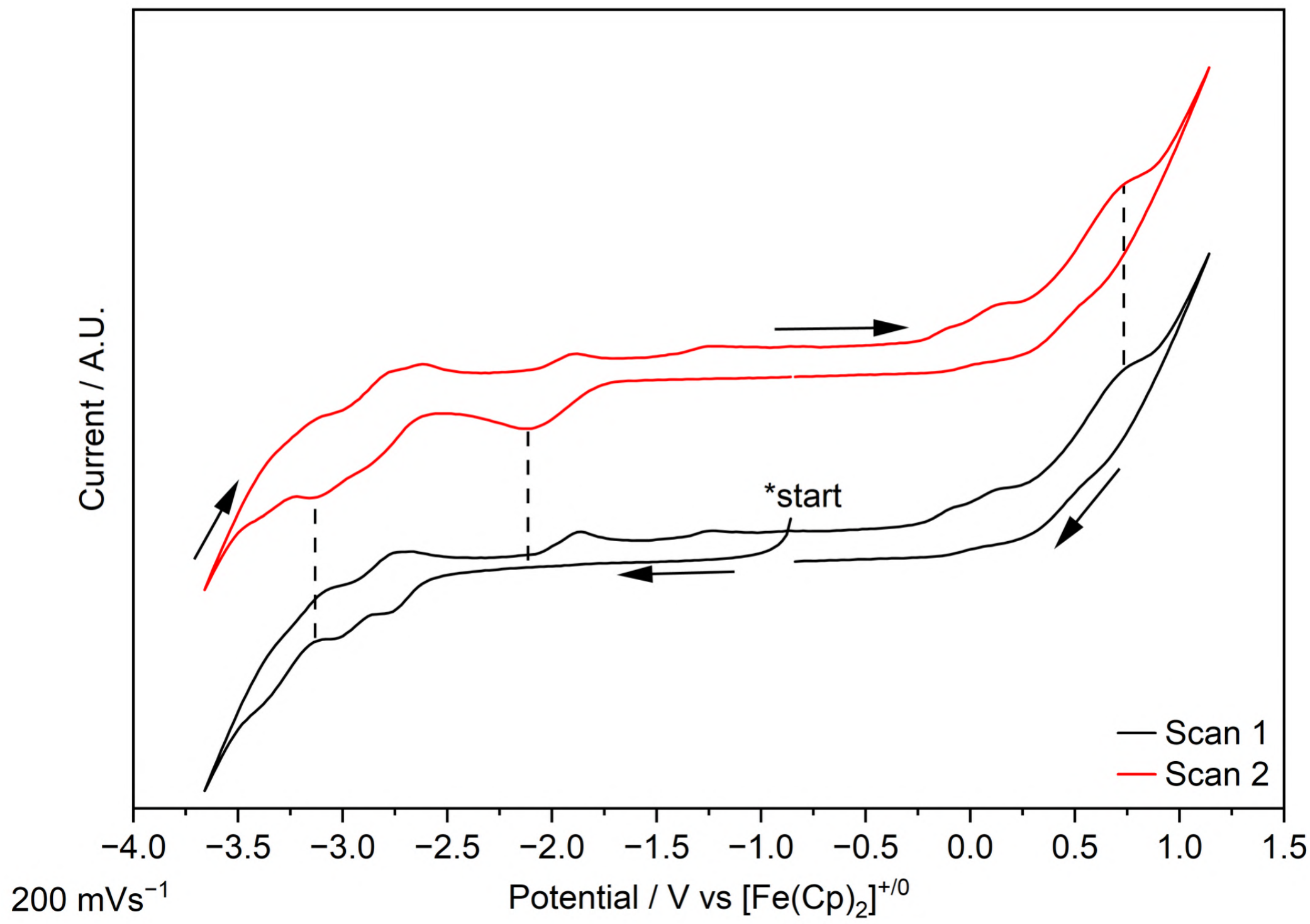

Supplementary Figure 56. Cyclic voltammogram of **4K'** (5.0 mM) in THF supported by $[Bu^n_4N][BPh_4]$ (50 mM) versus the $Fc^{+/0}$ couple at 200 mV s^{-1} scanned in the negative direction.

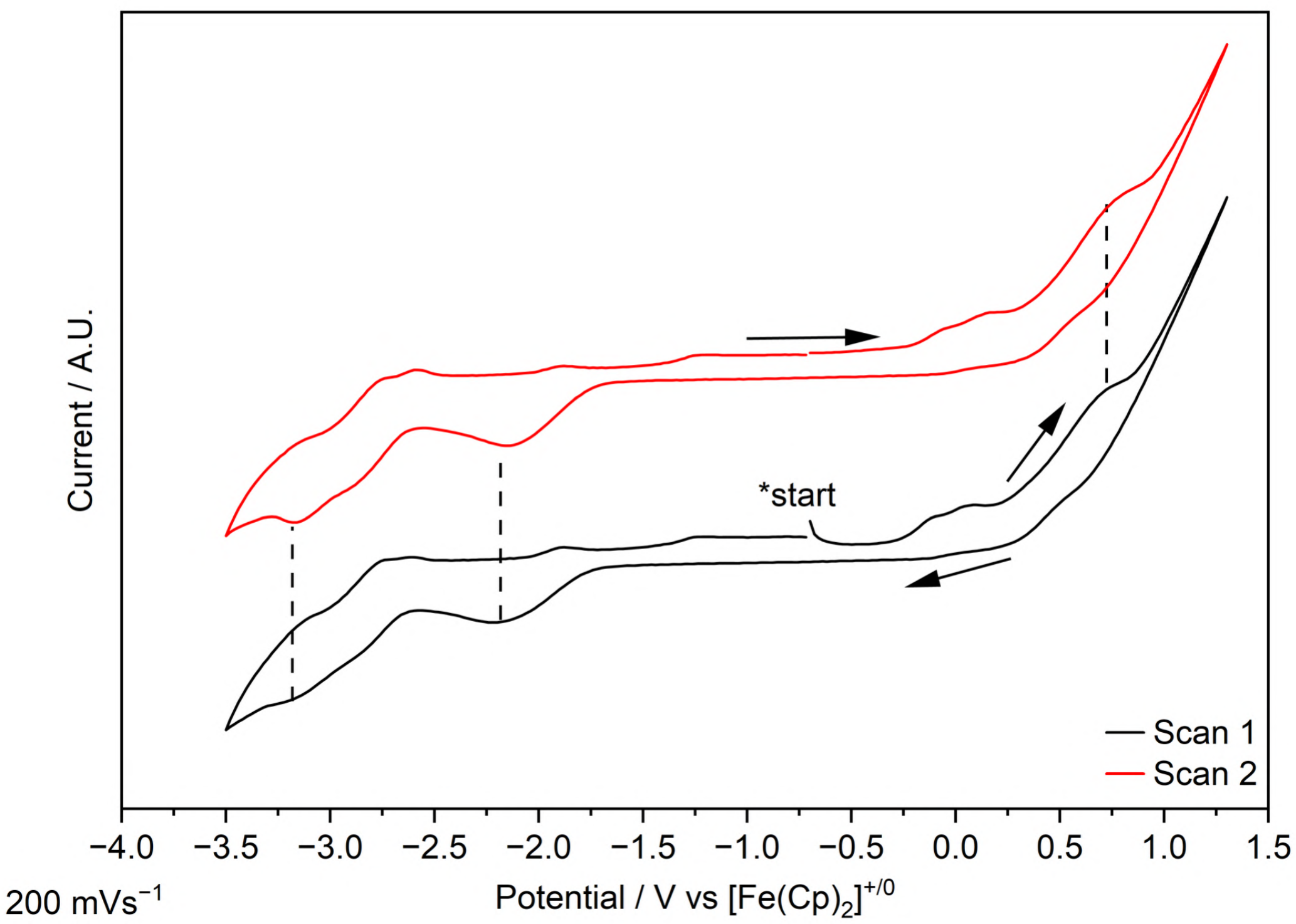

Supplementary Figure 57. Cyclic voltammogram of **4K'** (5.0 mM) in THF supported by [$Bu^n{}_4N$][BPh_4] (50 mM) versus the $Fc^{+/0}$ couple at 200 mV s^{-1} scanned in the positive direction.

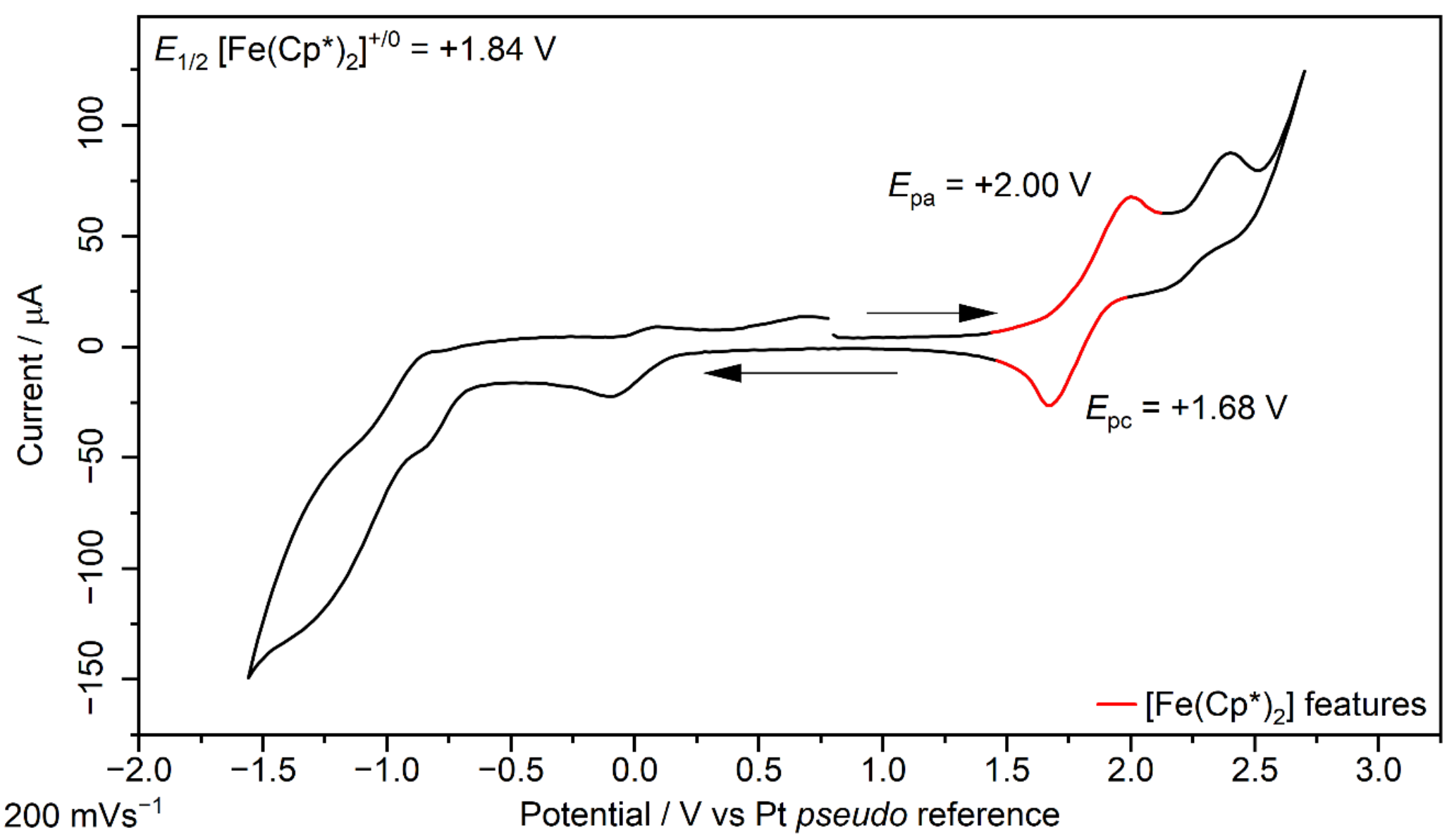

Supplementary Figure 58. Cyclic voltammogram of **5K** (5.0 mM) with [$Fe(\eta^5\text{-}C_5Me_5)_2$] internal reference (ca. 3 mg) in THF supported by [$Bu^n{}_4N$][BPh_4] (50 mM) versus Pt pseudo reference at 200 mV s^{-1}.

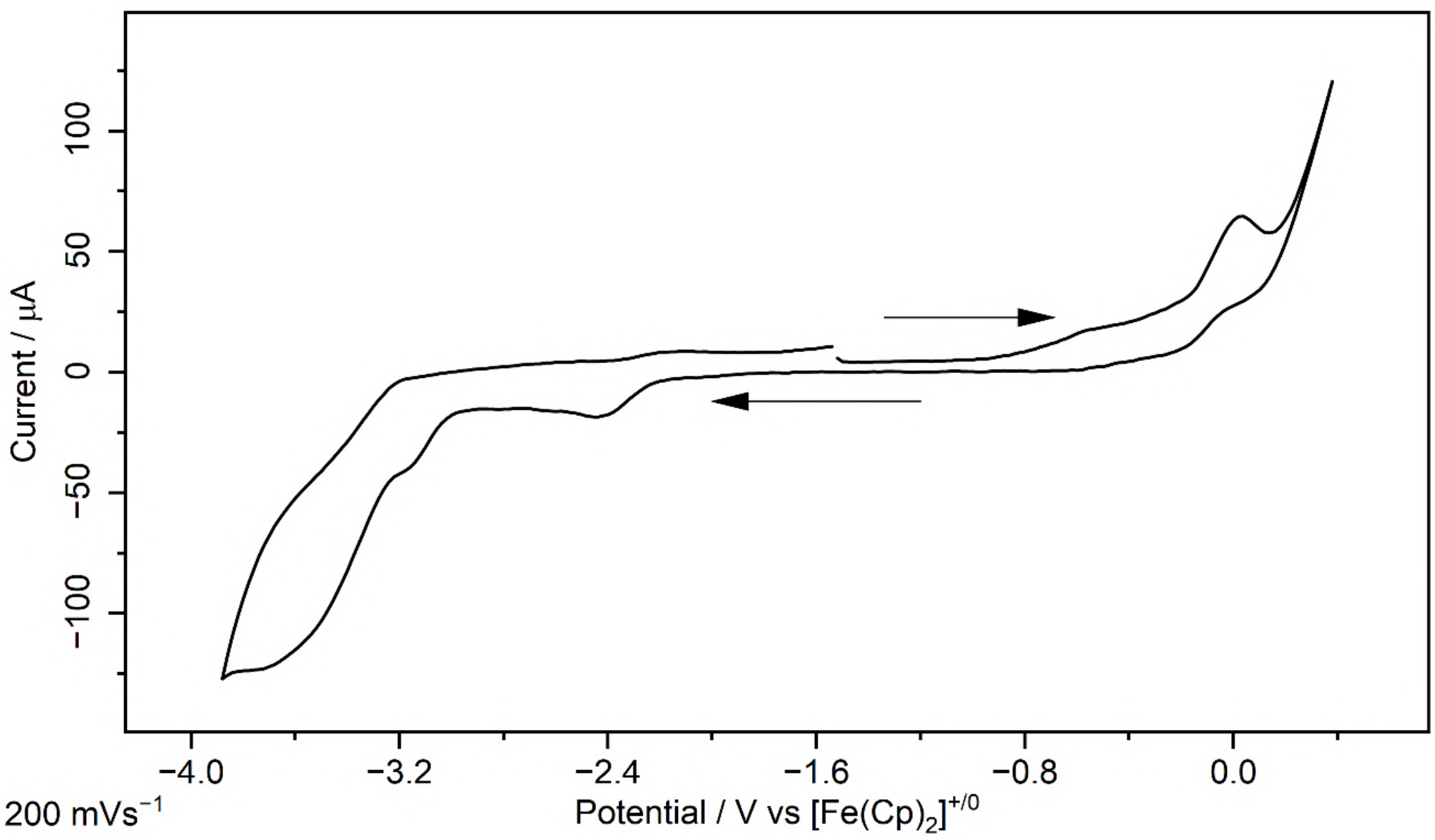

Supplementary Figure 59. Cyclic voltammogram of **5K** (5.0 mM) in THF supported by $[Bu^n{}_4N][BPh_4]$ (50 mM) versus the $Fc^{+/0}$ couple at 200 mV s^{-1}.

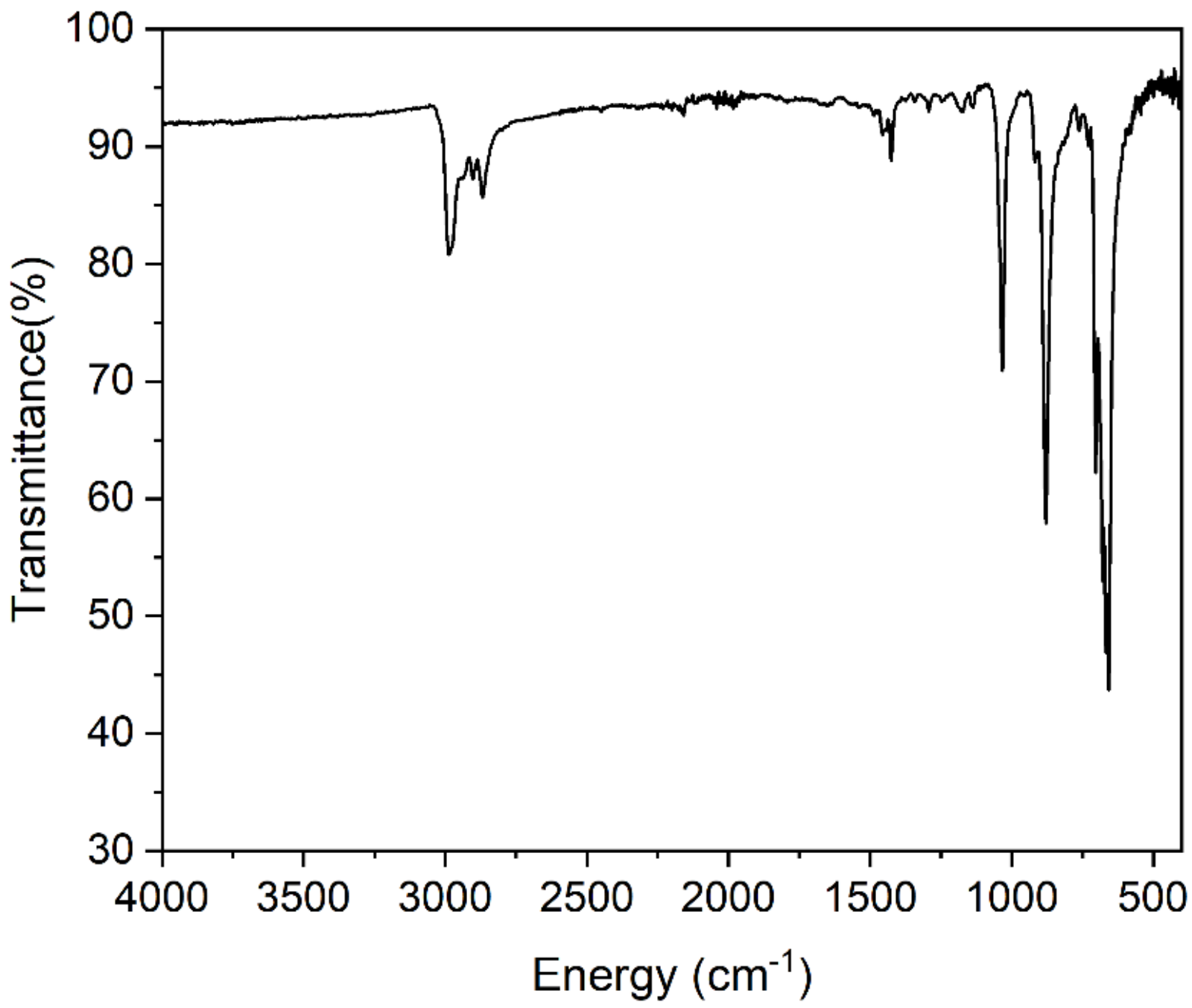

Supplementary Figure 60. ATR-IR spectrum of $C_8H_8K_2$.

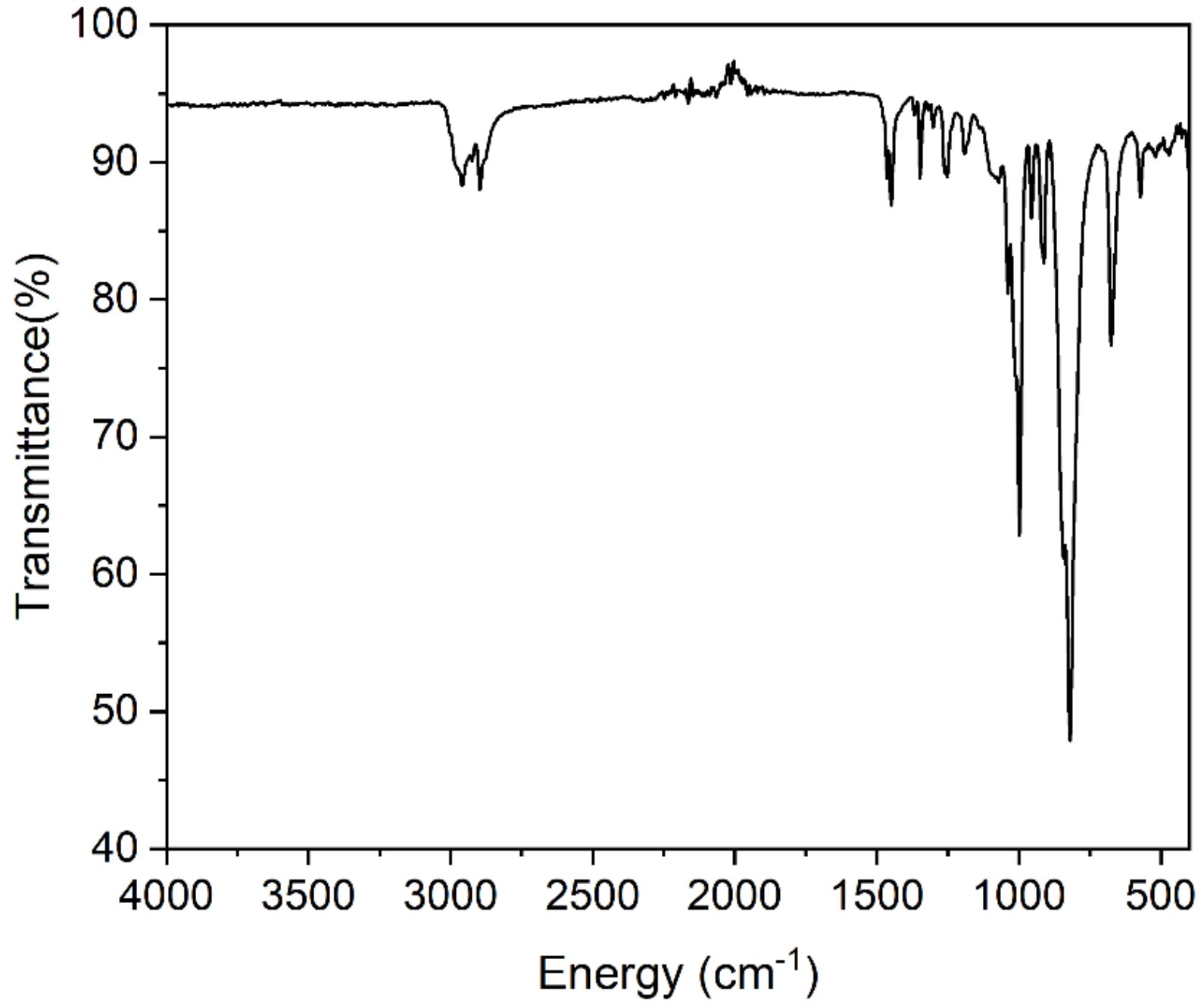

Supplementary Figure 61. ATR-IR spectrum of $ThCl_4(THF)_{3.5}$.

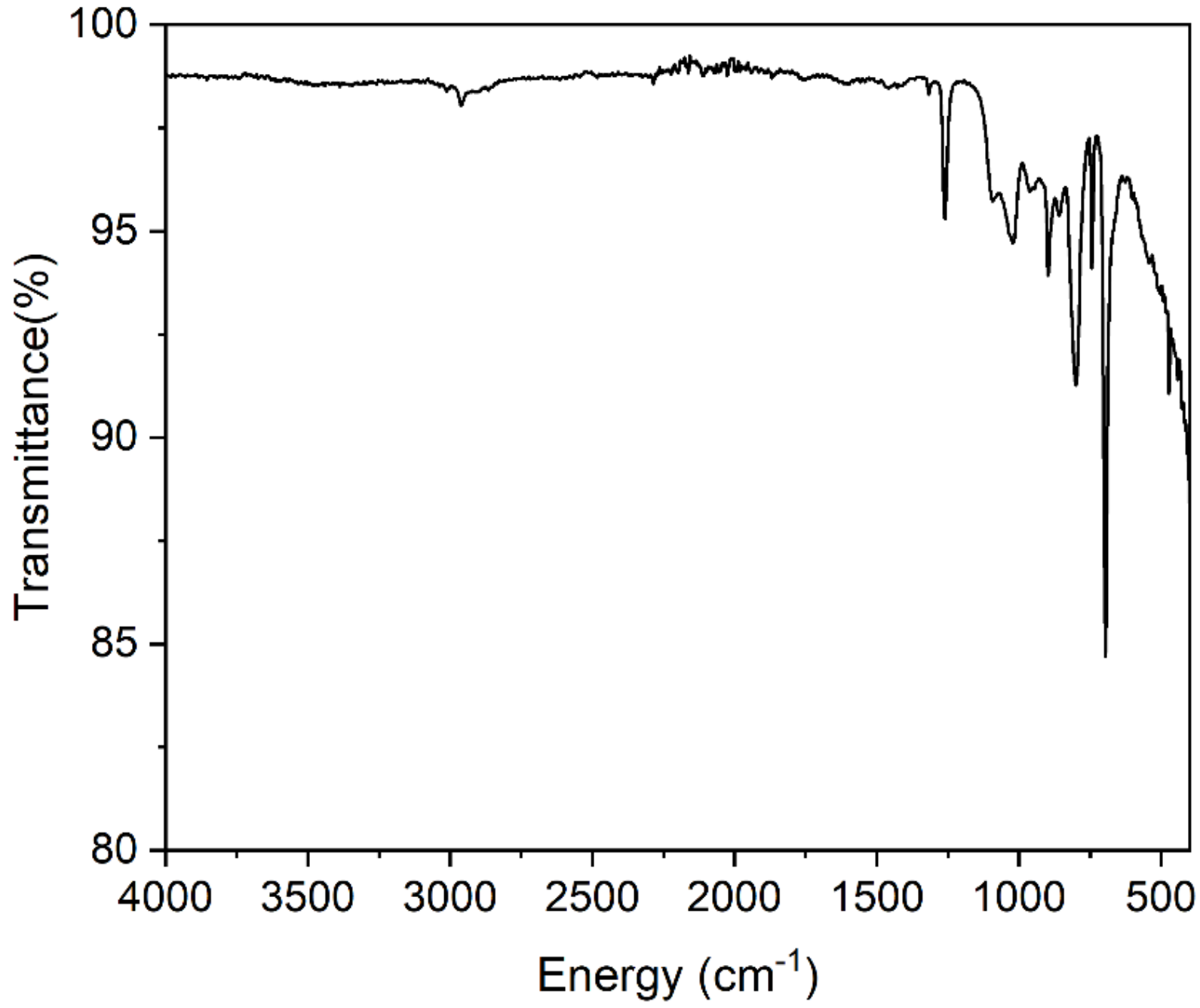

Supplementary Figure 62. ATR-IR spectrum of $[Th(\eta^8-C_8H_8)_2]$.

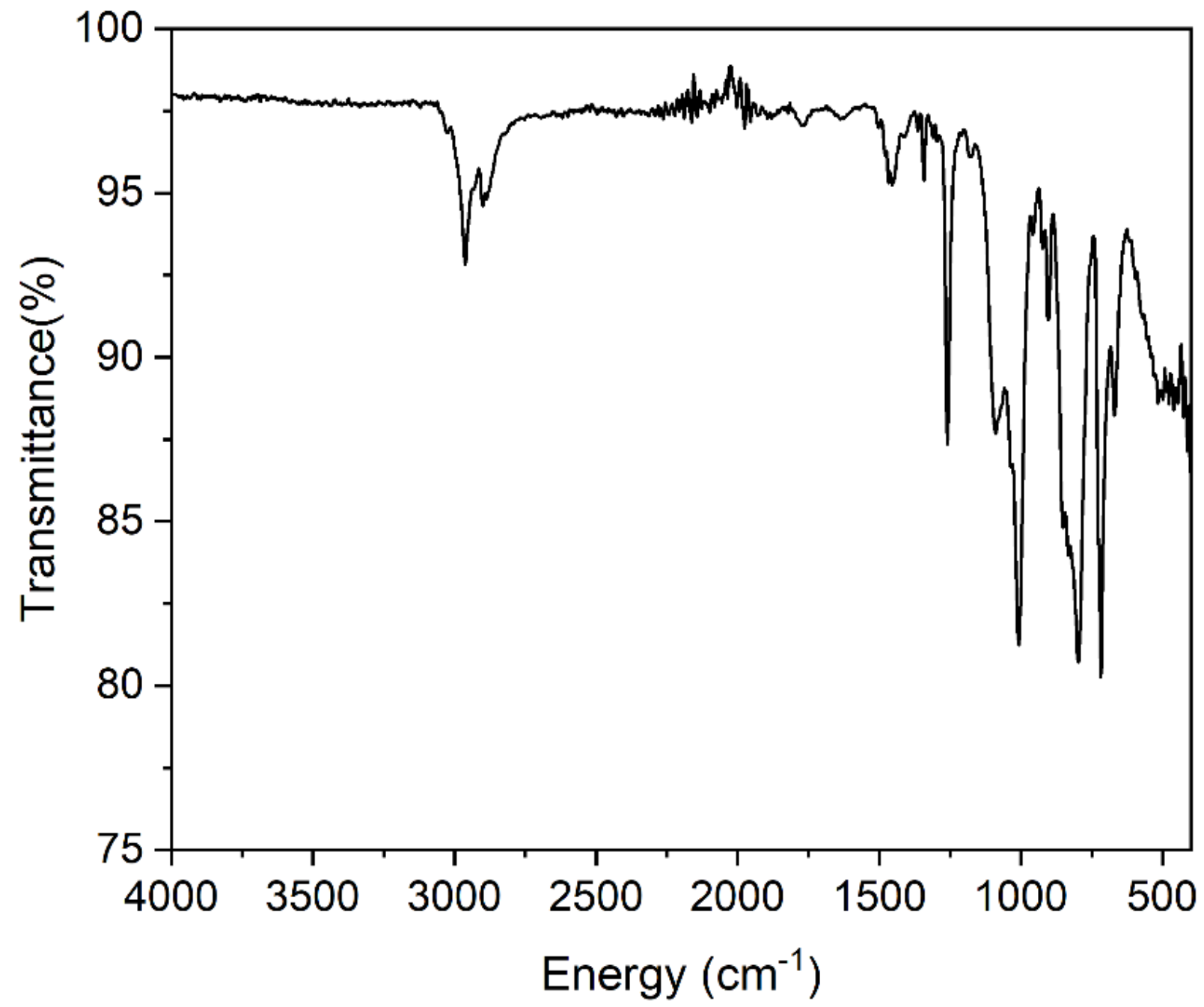

Supplementary Figure 63. ATR-IR spectrum of **1**.

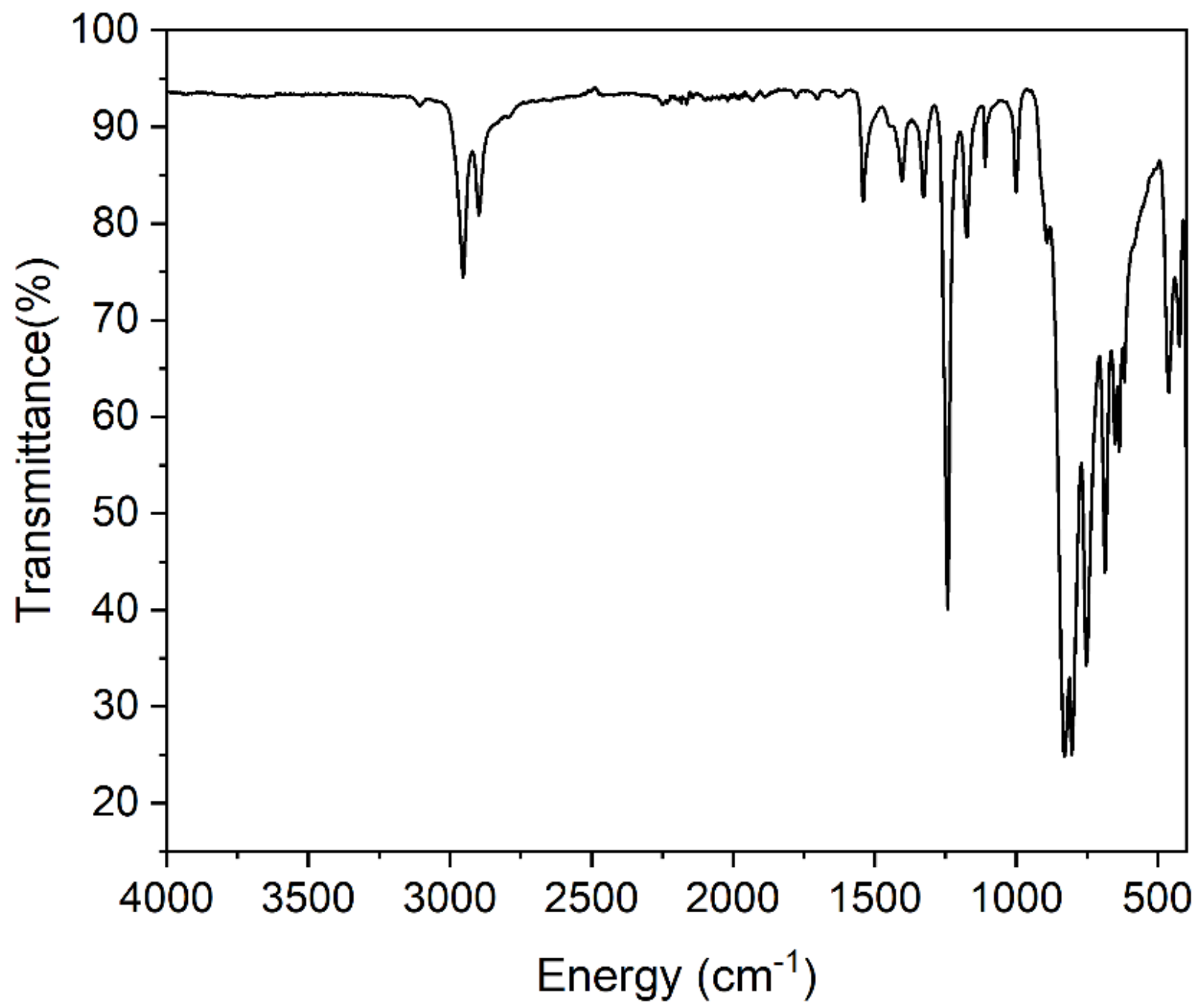

Supplementary Figure 64. ATR-IR spectrum of [Co{η^4-$C_4(SiMe_3)_4$}(η^5-C_5H_5)].

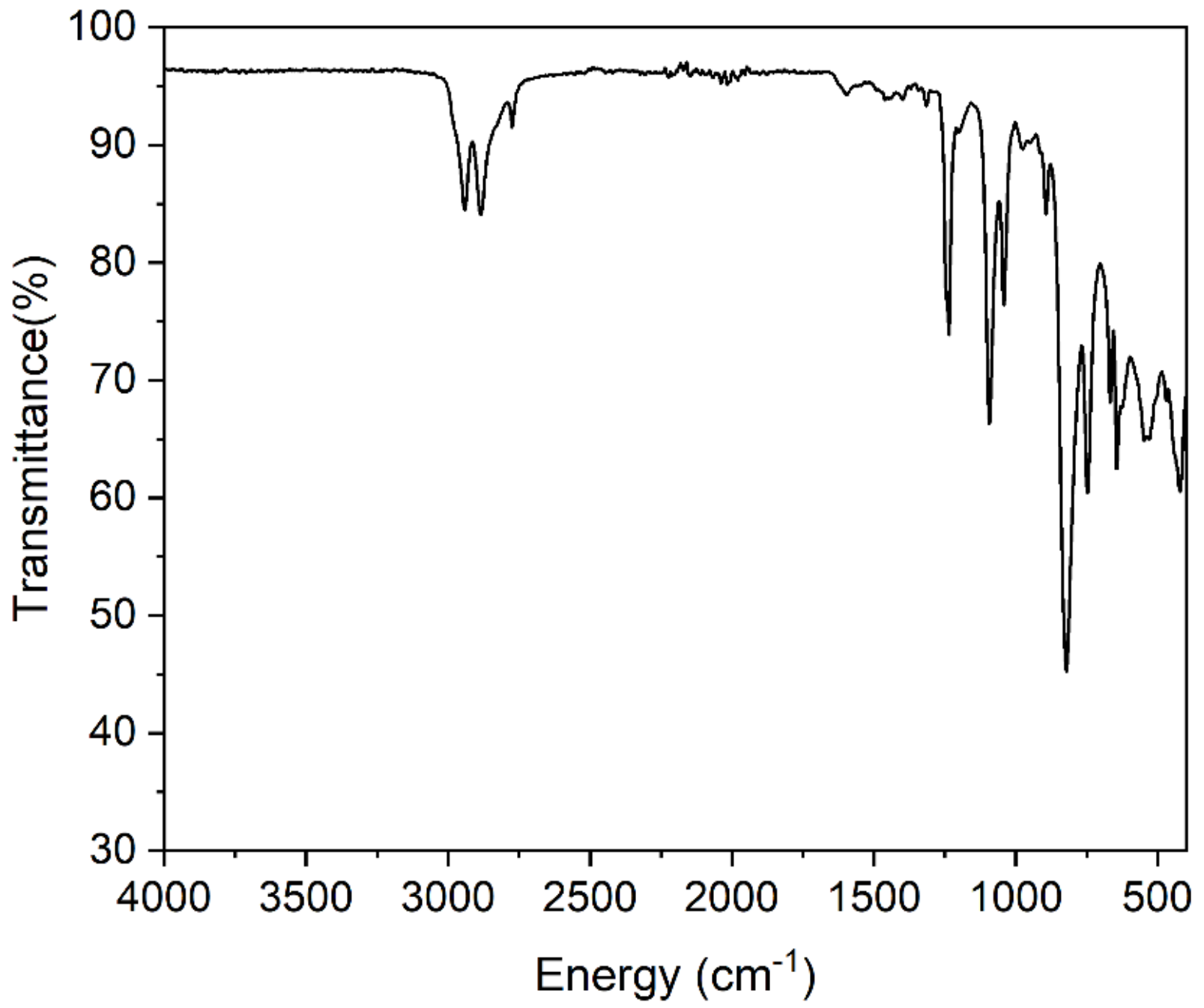

Supplementary Figure 65. ATR-IR spectrum of [Li_2{μ-η[4]:η[4]-$C_4(SiMe_3)_4$}$(THF)_2$].

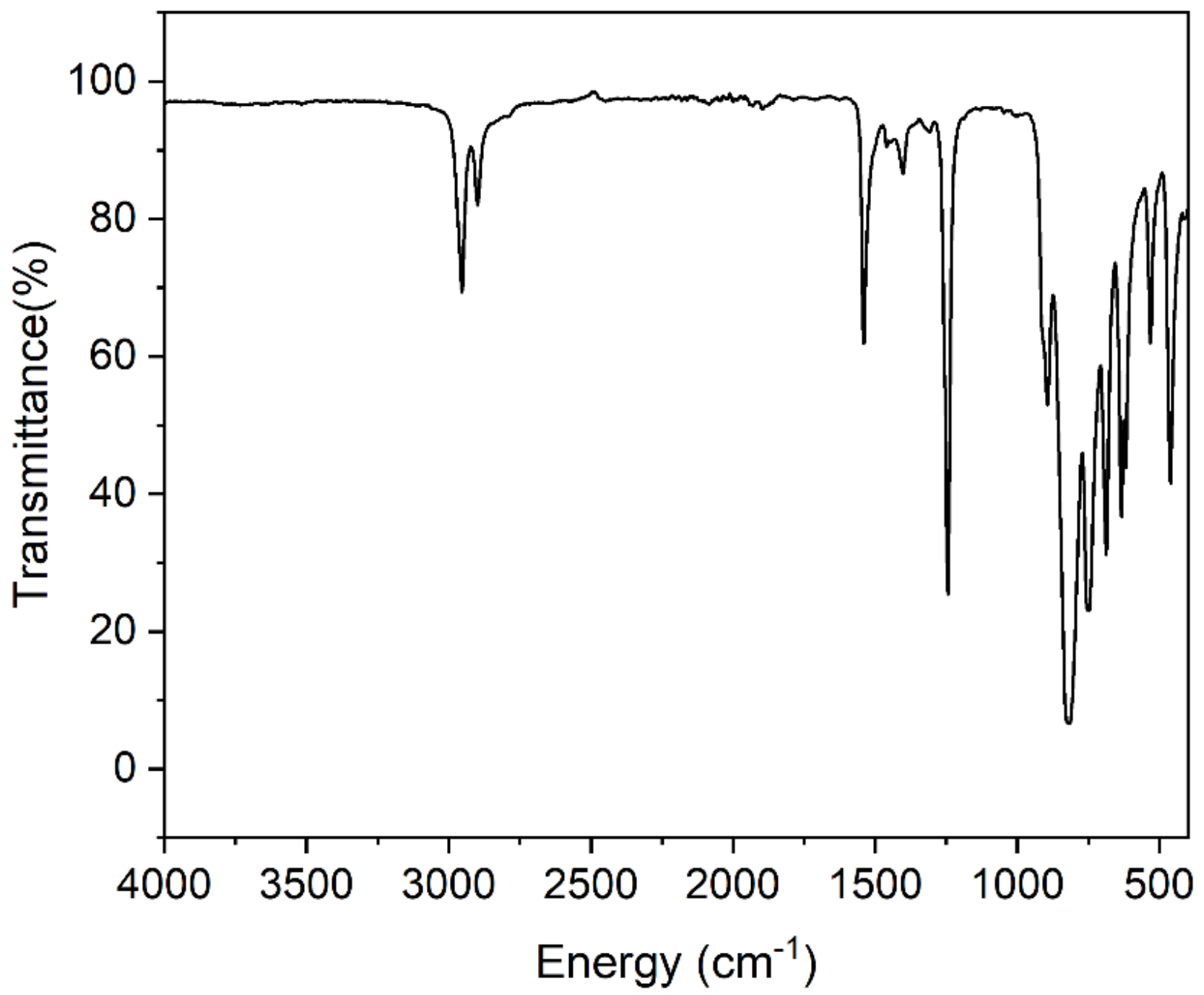

Supplementary Figure 66. ATR-IR spectrum of [$C_4(SiMe_3)_4$].

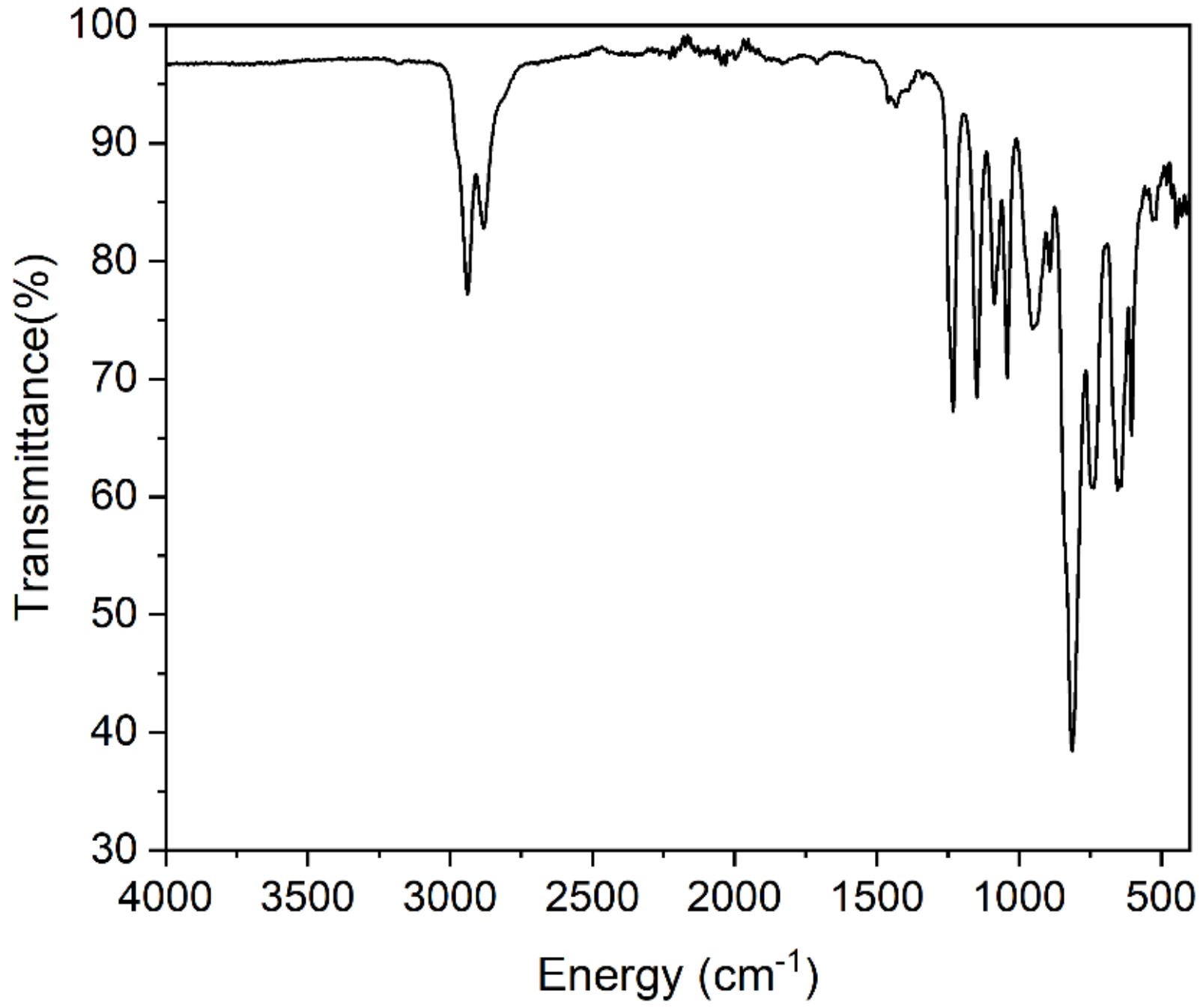

Supplementary Figure 67. ATR-IR spectrum of **2**.

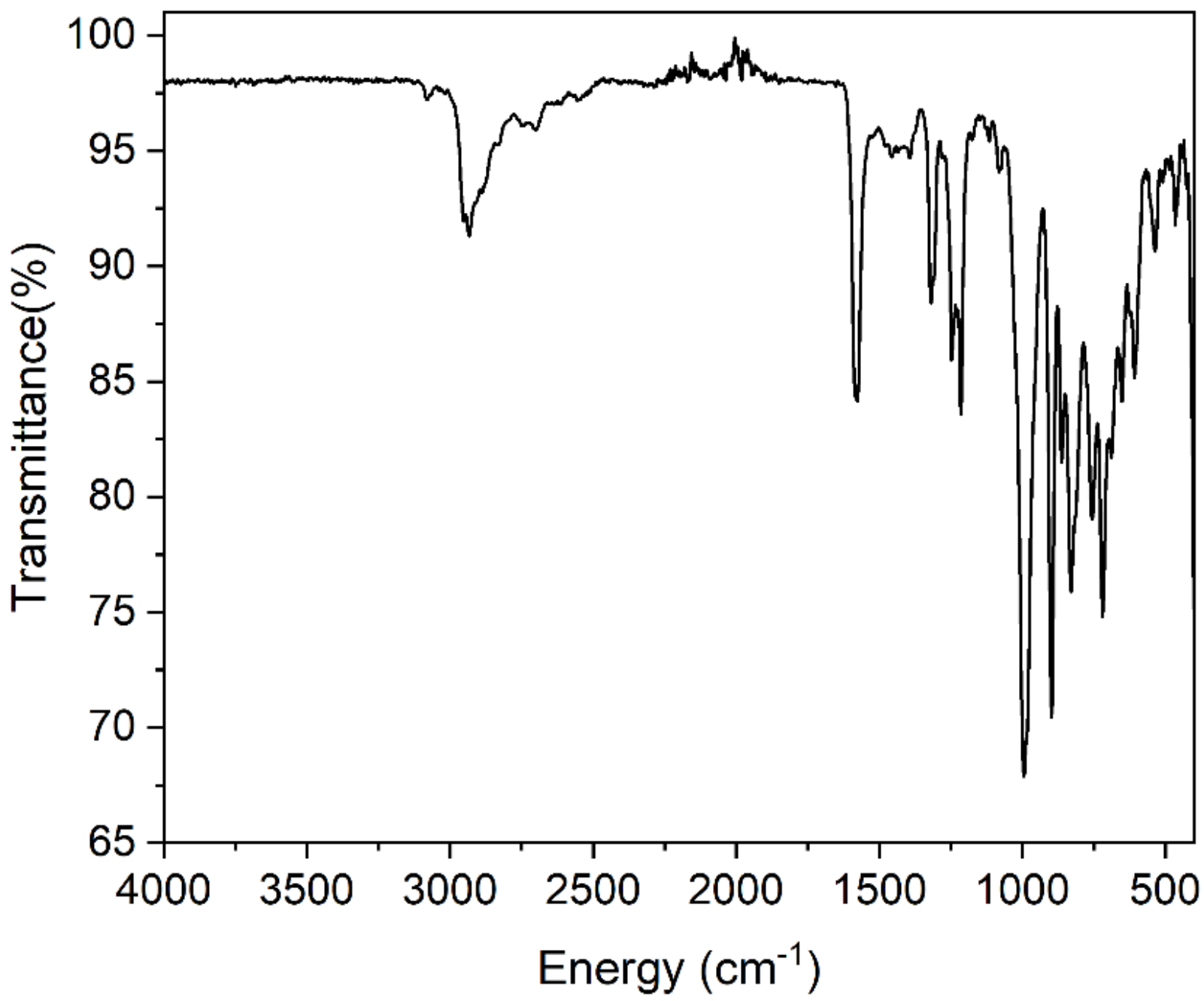

Supplementary Figure 68. ATR-IR spectrum of **3**.

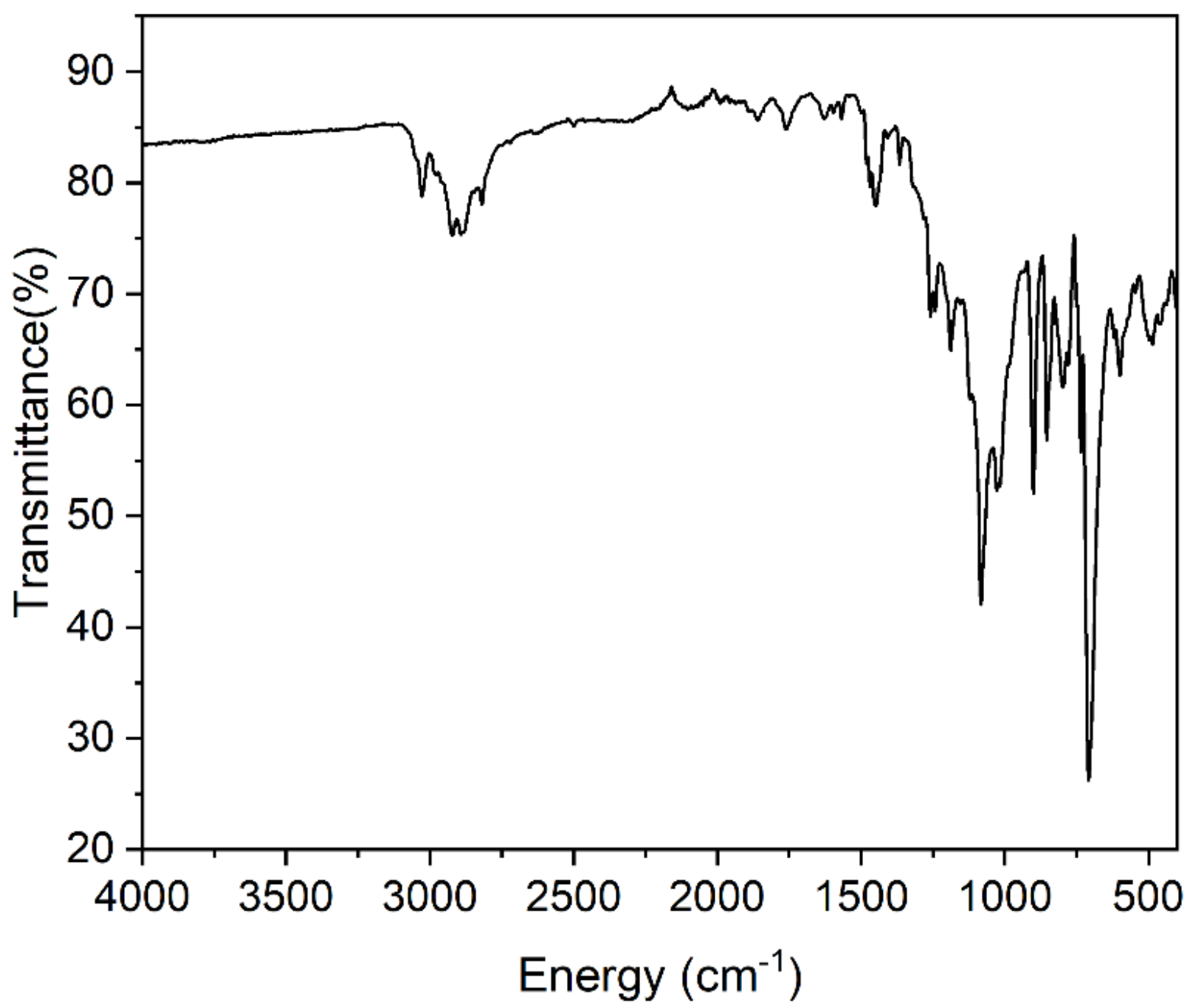

Supplementary Figure 69. ATR-IR spectrum of **4K**.

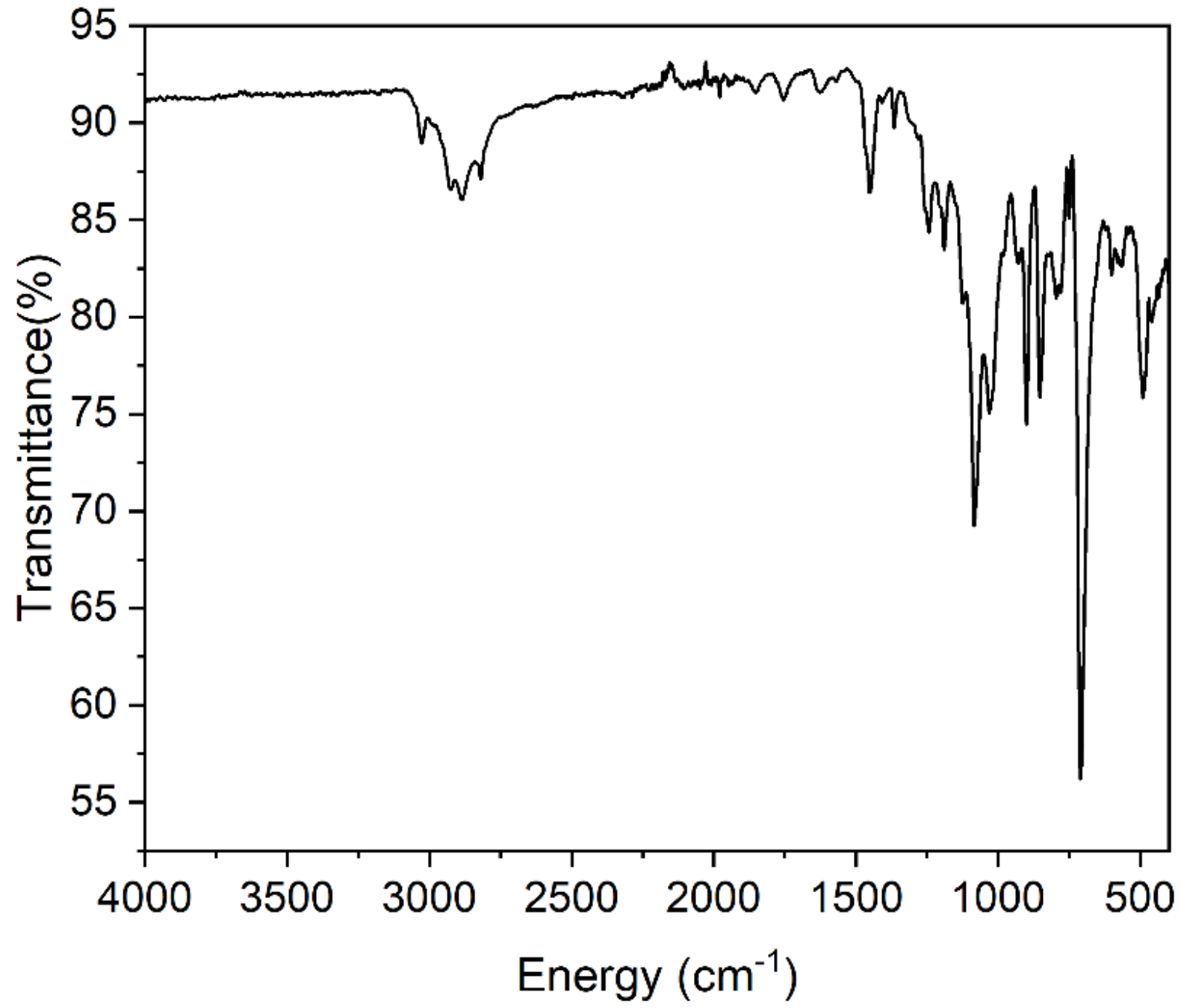

Supplementary Figure 70. ATR-IR spectrum of **4aRb**.

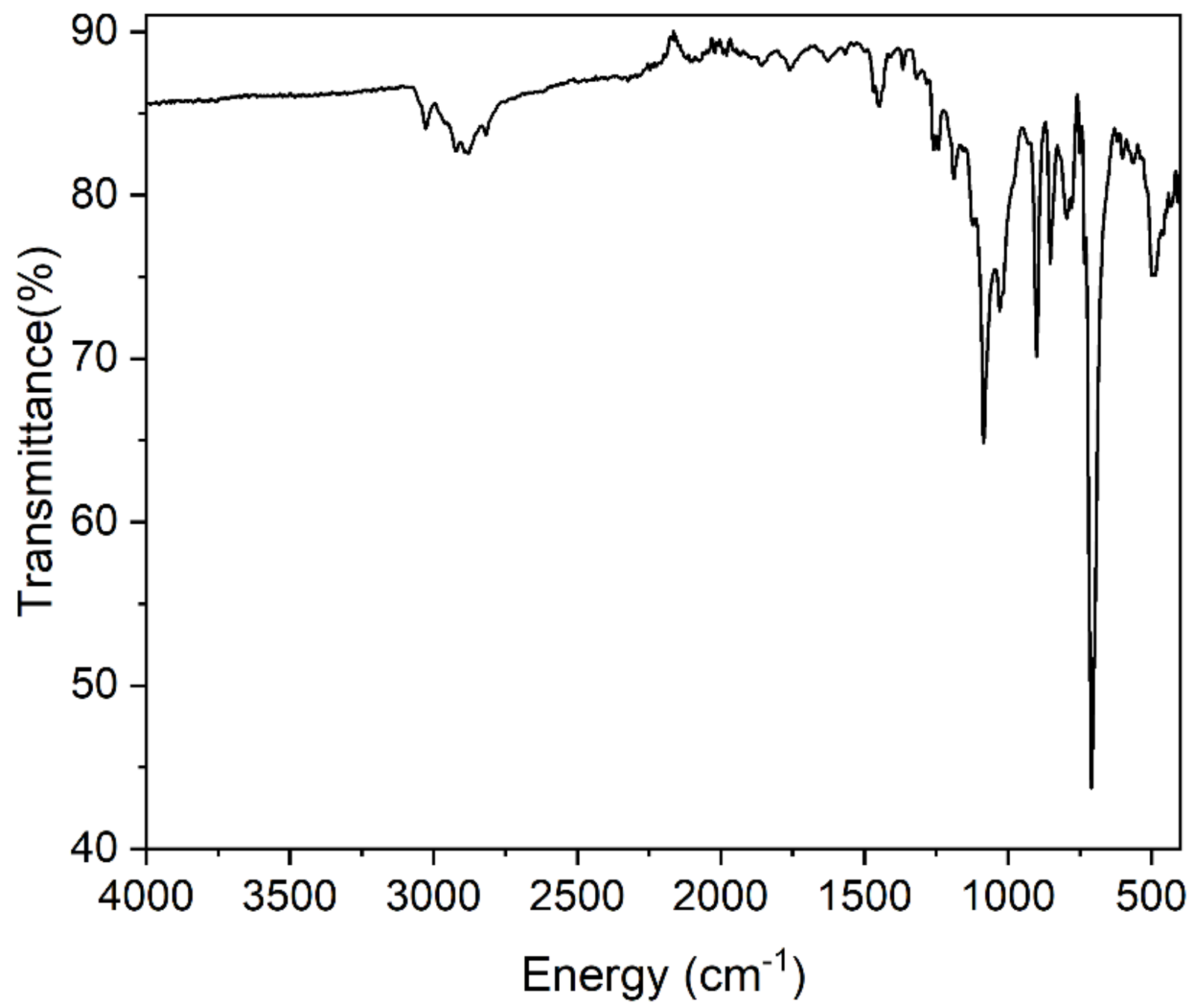

Supplementary Figure 71. ATR-IR spectrum of **4Cs**.

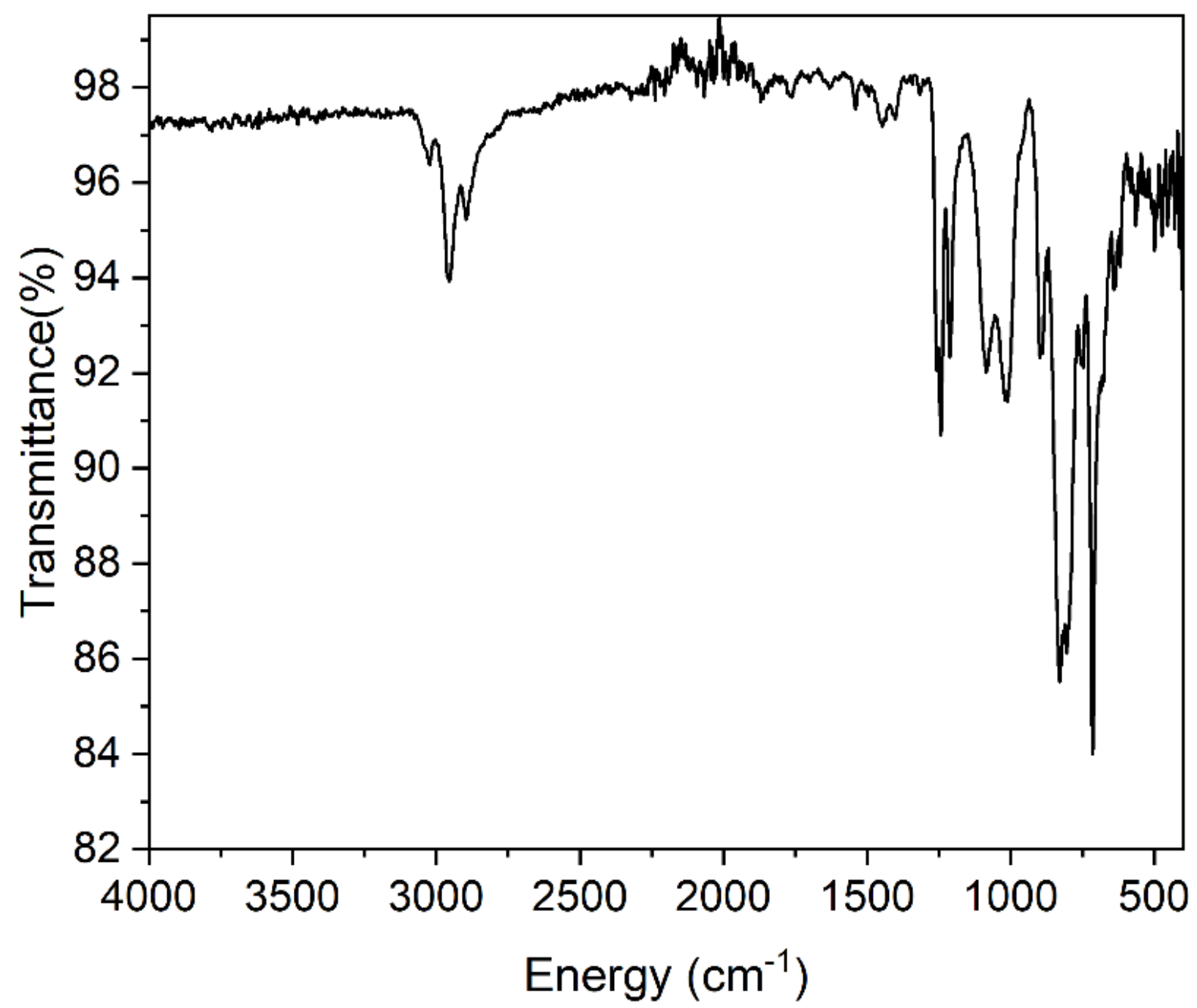

Supplementary Figure 72. ATR-IR spectrum of **5Na**.

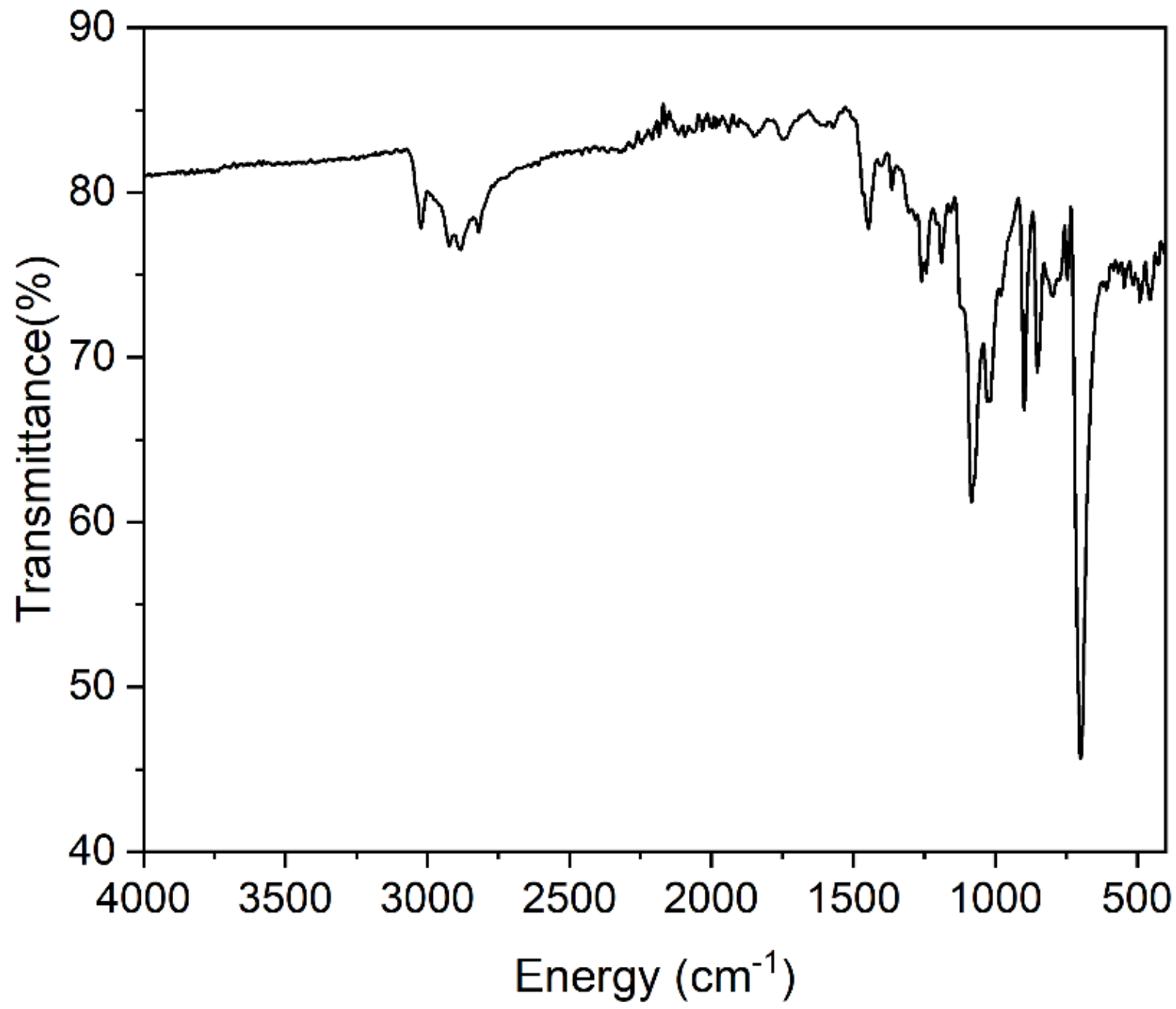

Supplementary Figure 73. ATR-IR spectrum of **5K**.

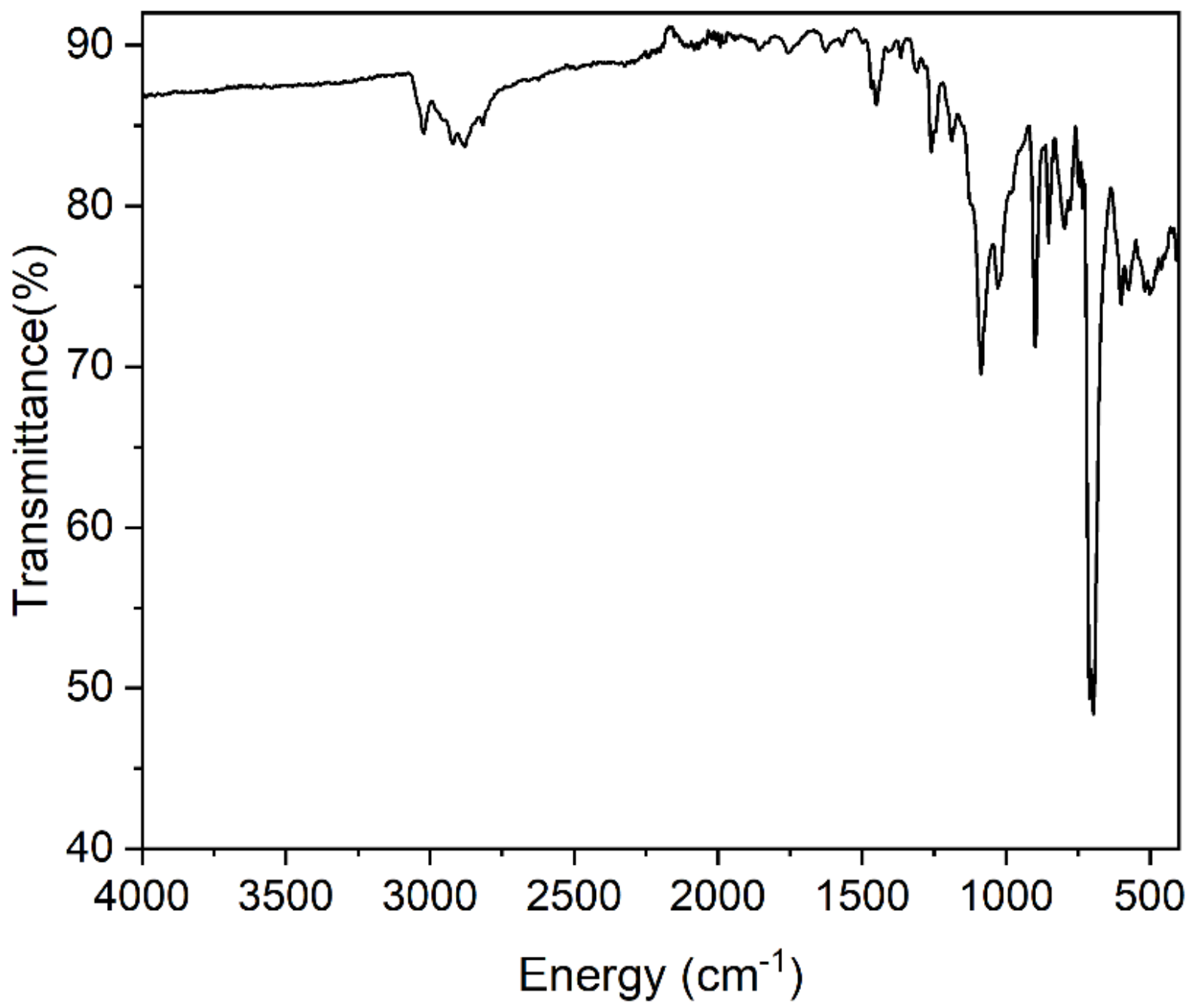

Supplementary Figure 74. ATR-IR spectrum of **5Rb**.

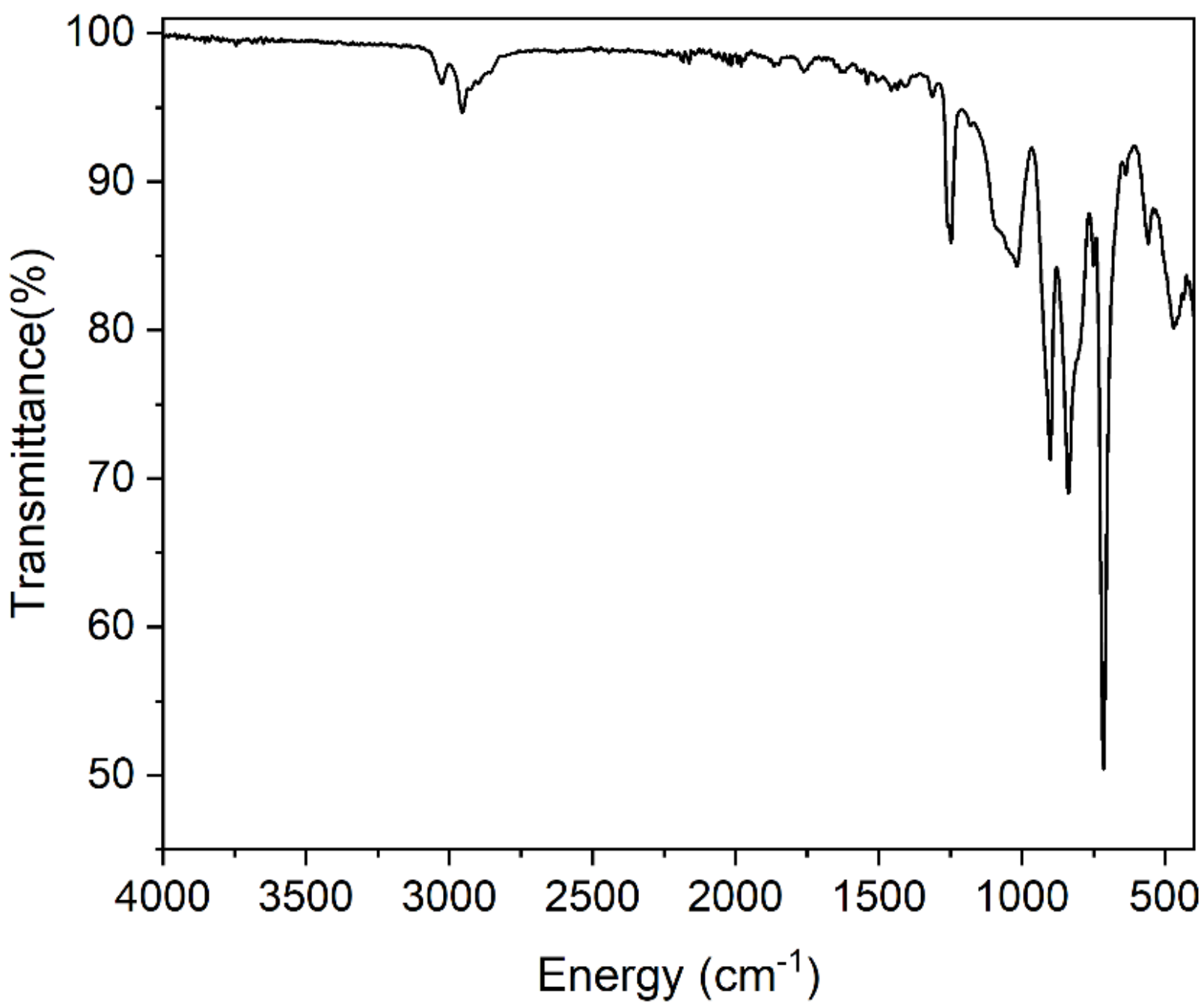

Supplementary Figure 75. ATR-IR spectrum of **5Cs**.

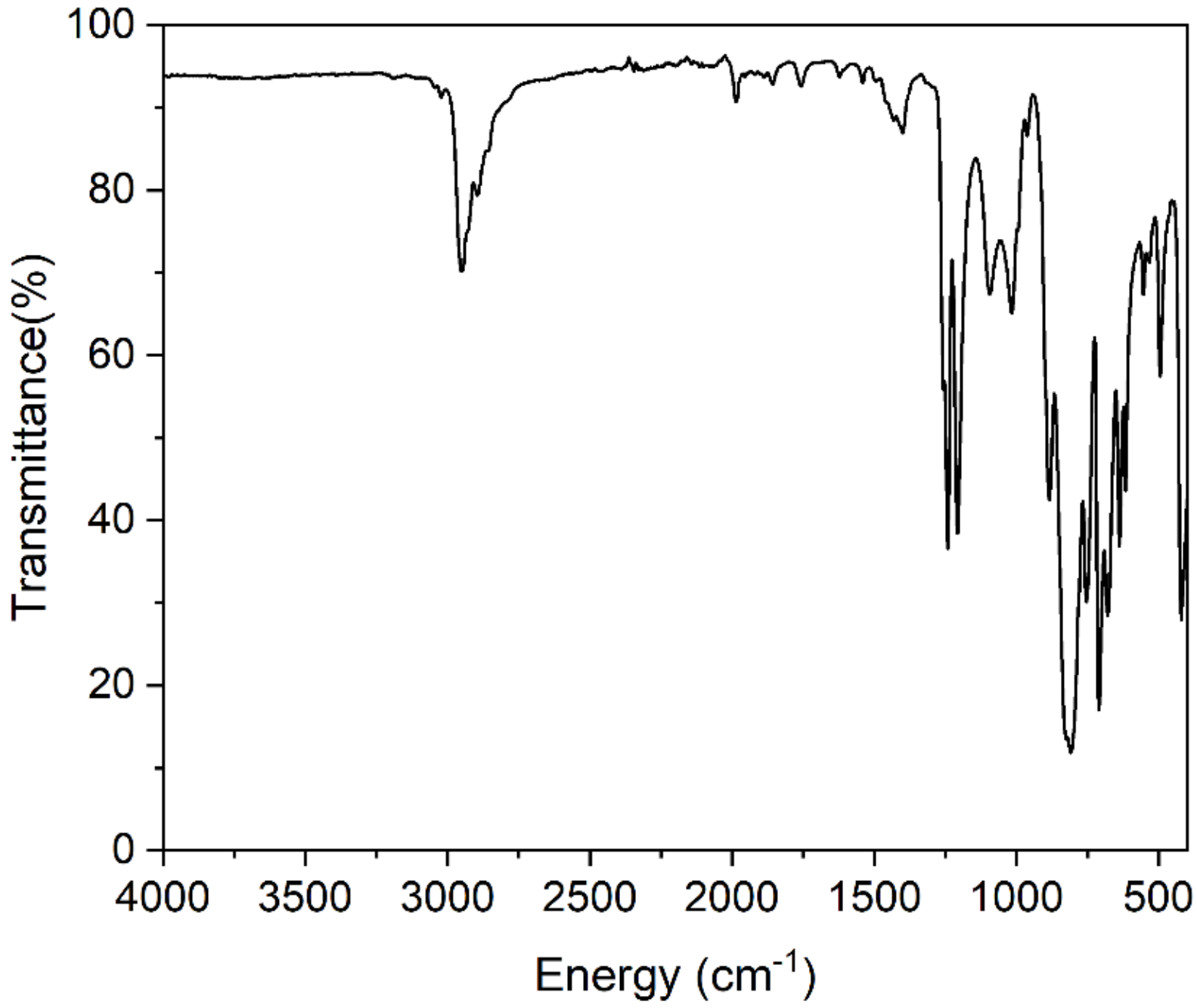

Supplementary Figure 76. ATR-IR spectrum of **6**.

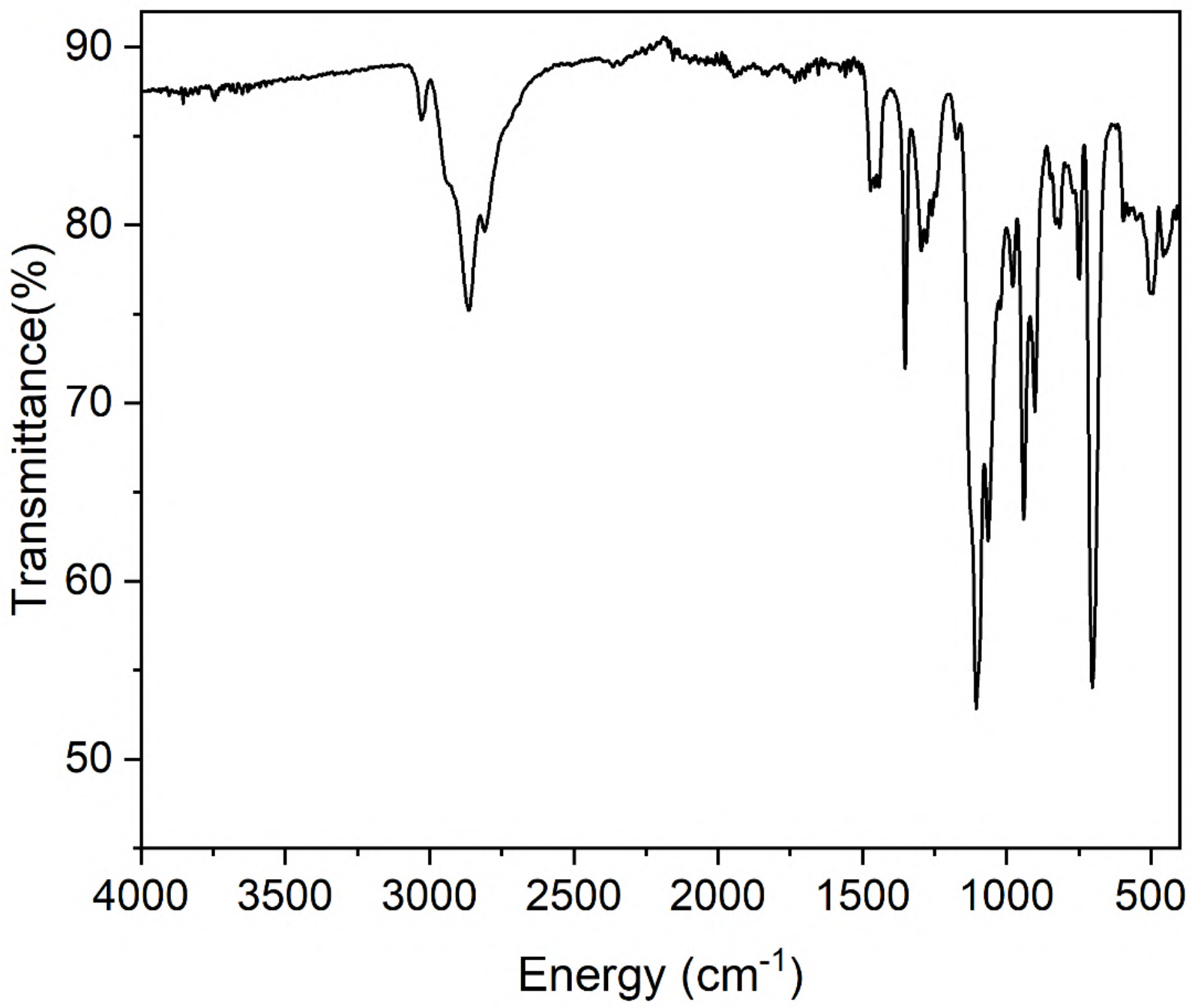

Supplementary Figure 77. ATR-IR spectrum of **7**.

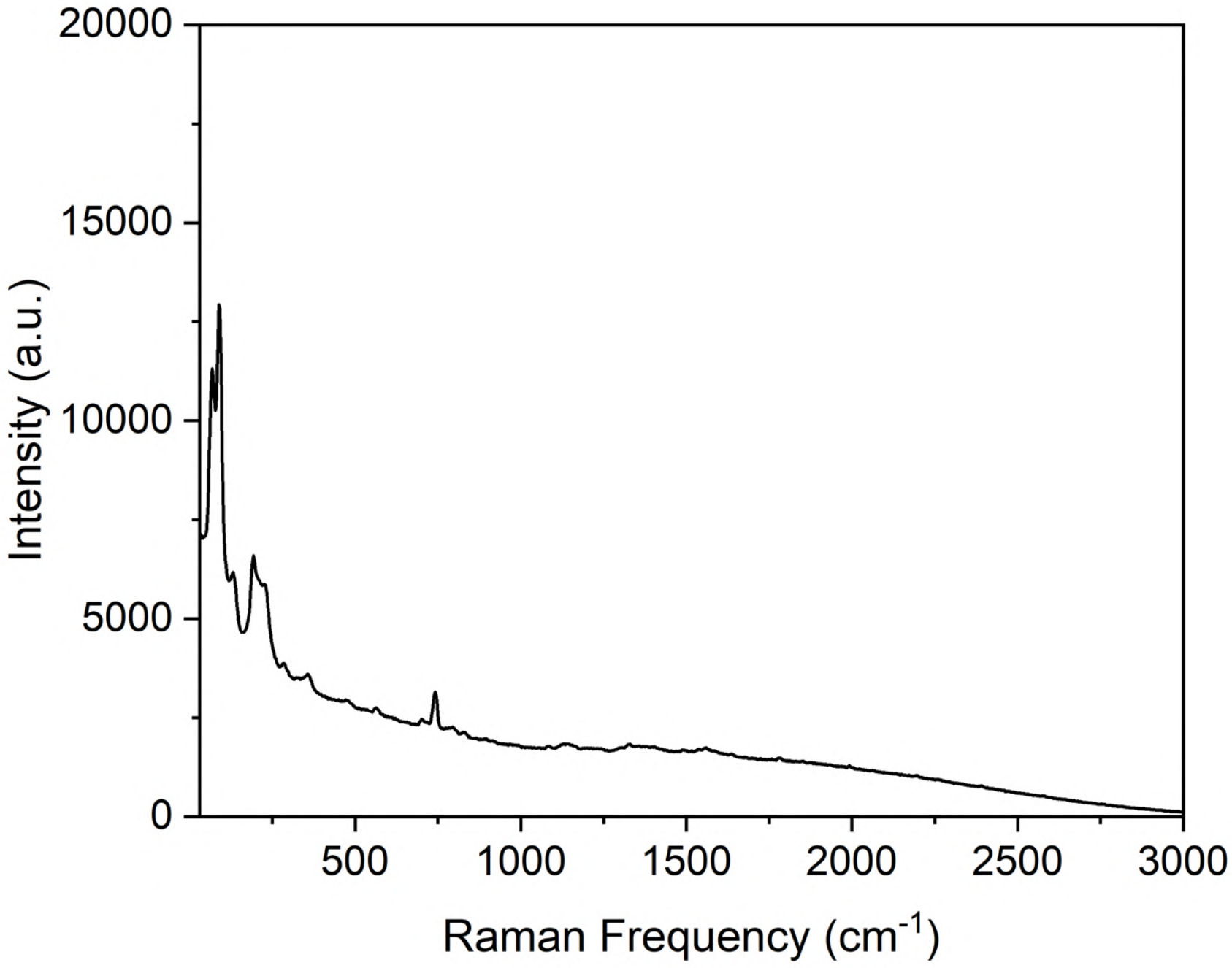

Supplementary Figure 78. Raman spectrum of **4K** using a 785 nm laser over the range 30–3000 cm^{-1}.

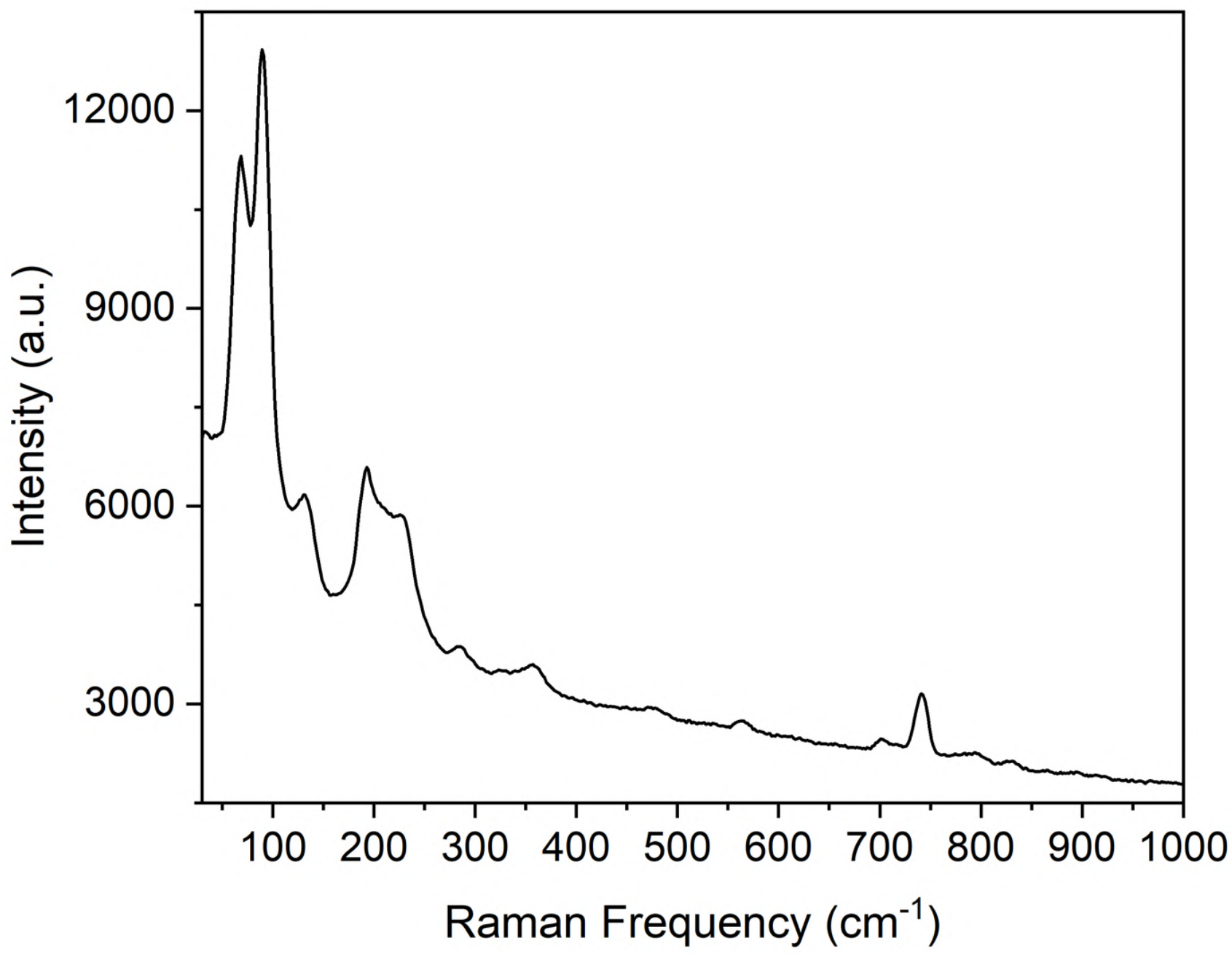

Supplementary Figure 79. Raman spectrum of **4K** using a 785 nm laser zoomed in over the range 30–1000 cm^{-1}.

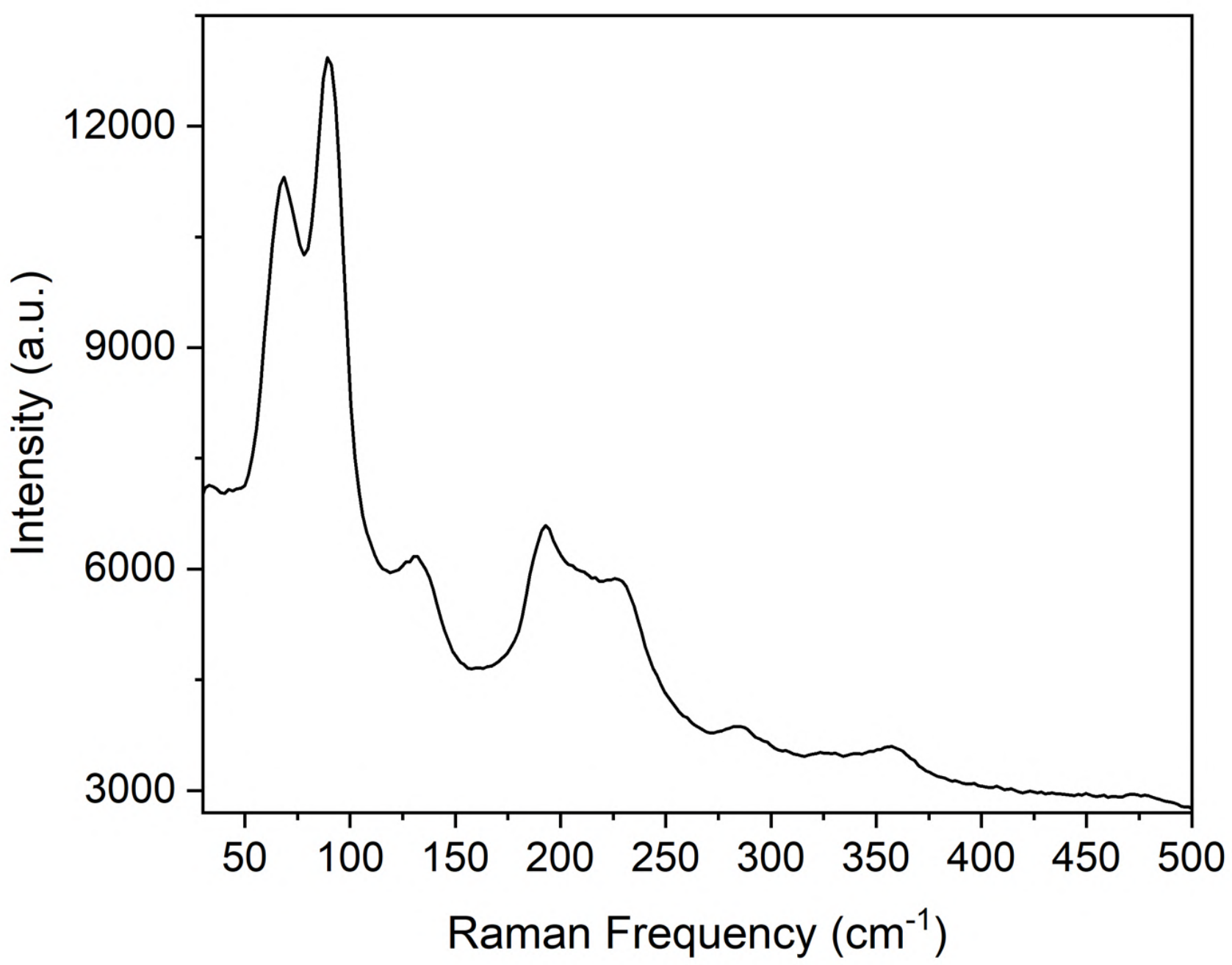

Supplementary Figure 80. Raman spectrum of **4K** using a 785 nm laser zoomed in over the range 30–500 cm^{-1}.

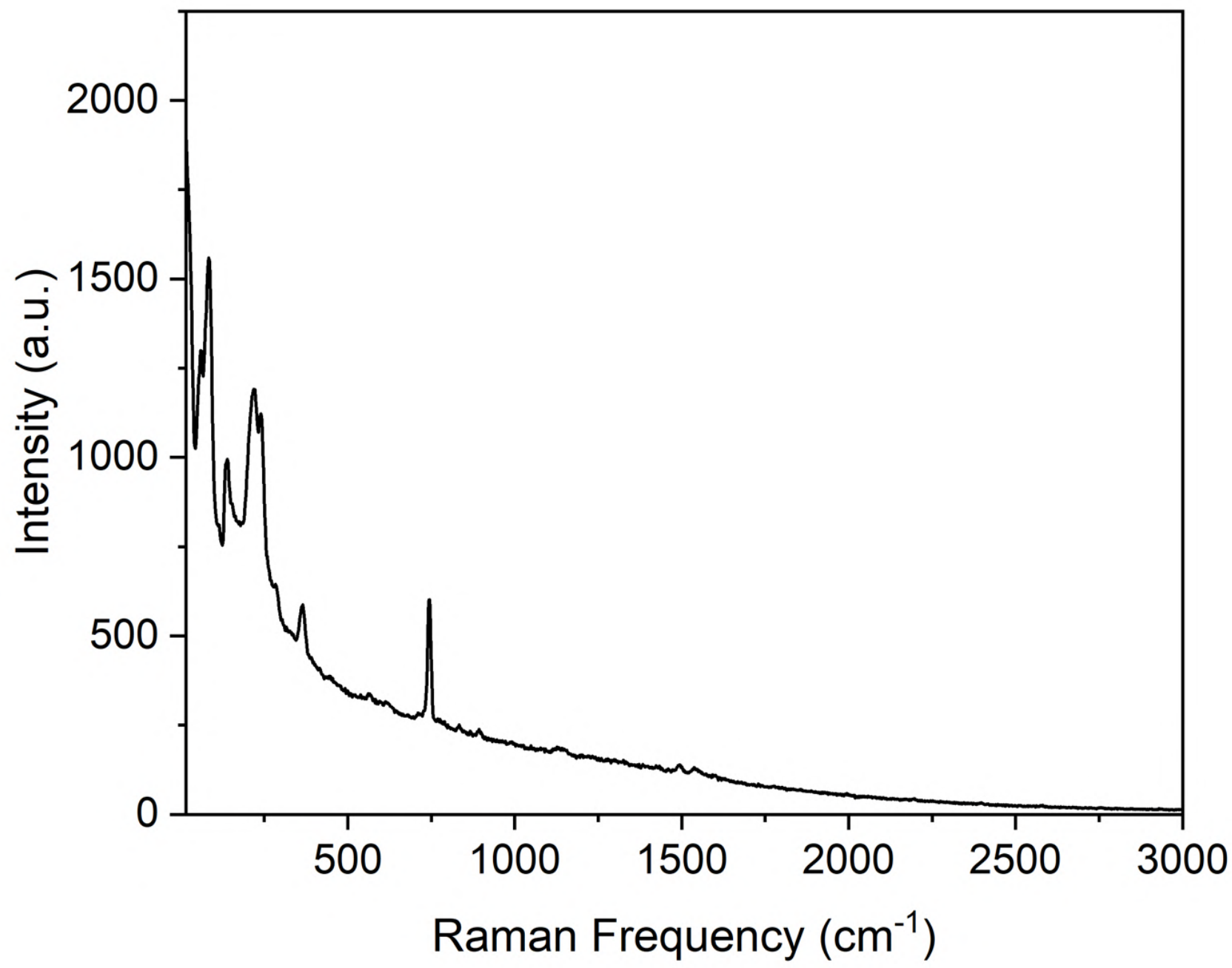

Supplementary Figure 81. Raman spectrum of **4aRb** using a 785 nm laser over the range 30–3000 cm^{-1}.

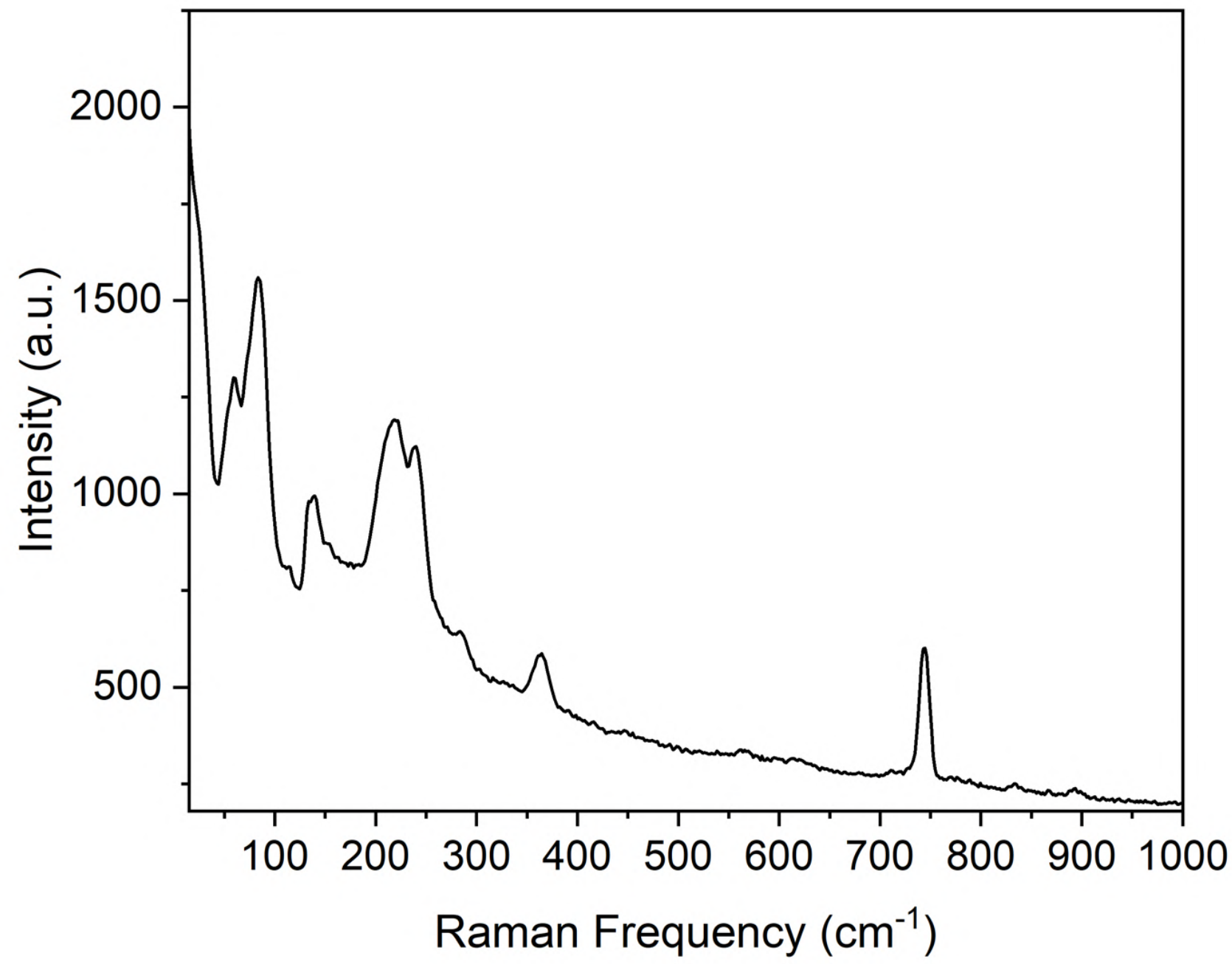

Supplementary Figure 82. Raman spectrum of **4aRb** using a 785 nm laser zoomed in over the range 30–1000 cm^{-1}.

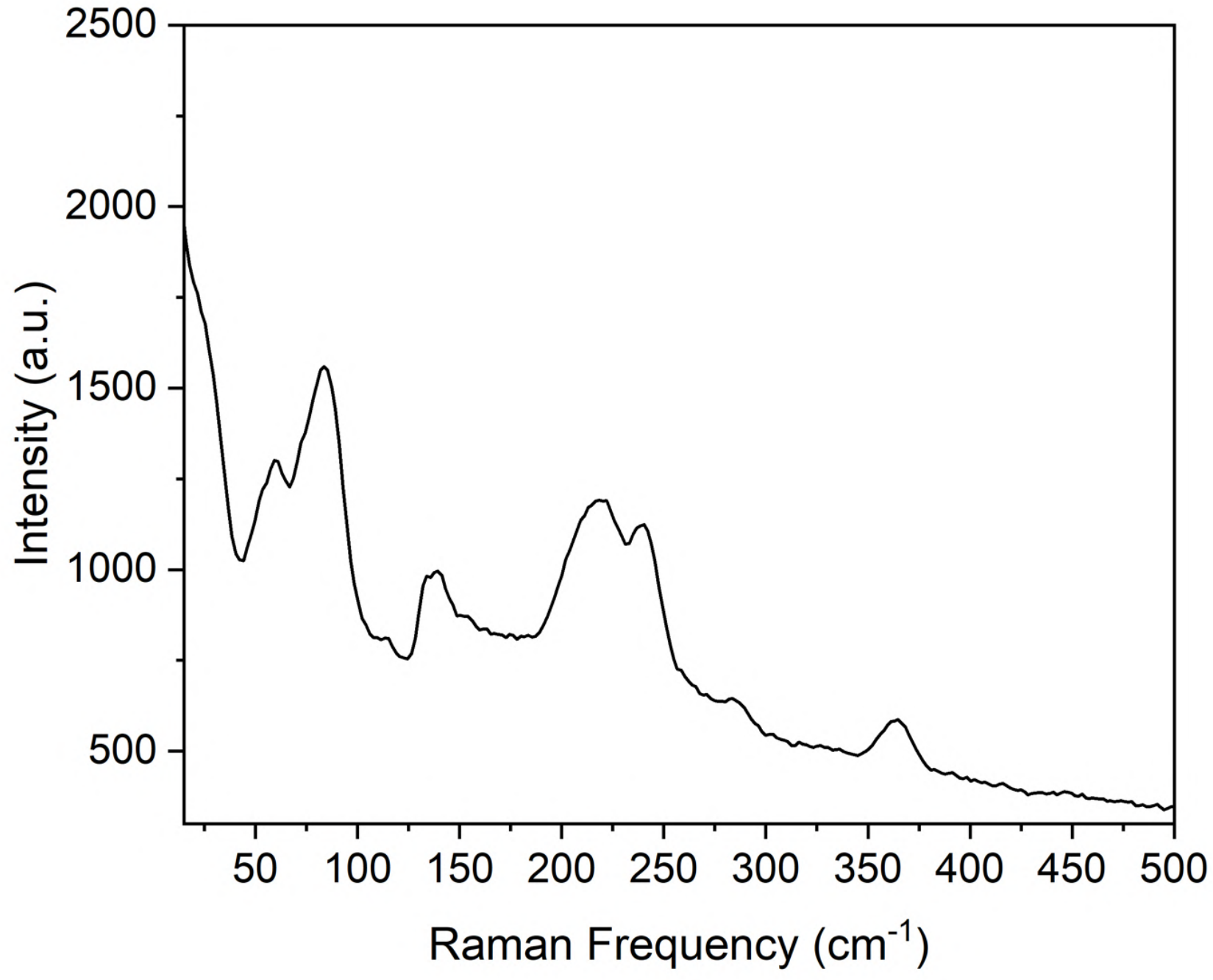

Supplementary Figure 83. Raman spectrum of **4aRb** using a 785 nm laser zoomed in over the range 30–500 cm^{-1}.

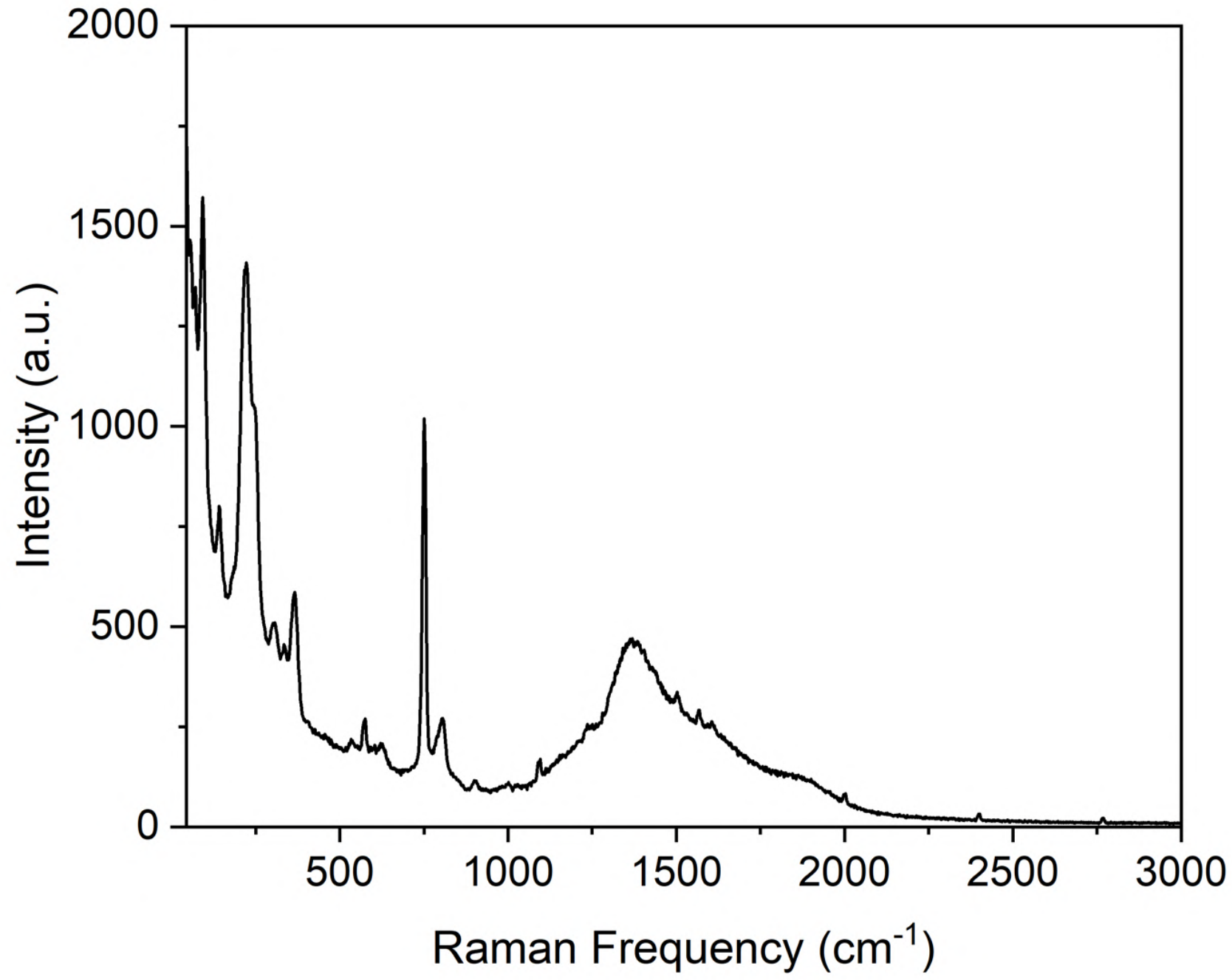

Supplementary Figure 84. Raman spectrum of **4Cs** using a 785 nm laser over the range 30–3000 cm^{-1}. The large broad feature at ~1400 cm^{-1} results from photoluminescence of the glass microscope slide used for the measurement. Further details are provided in the Supplementary Text.

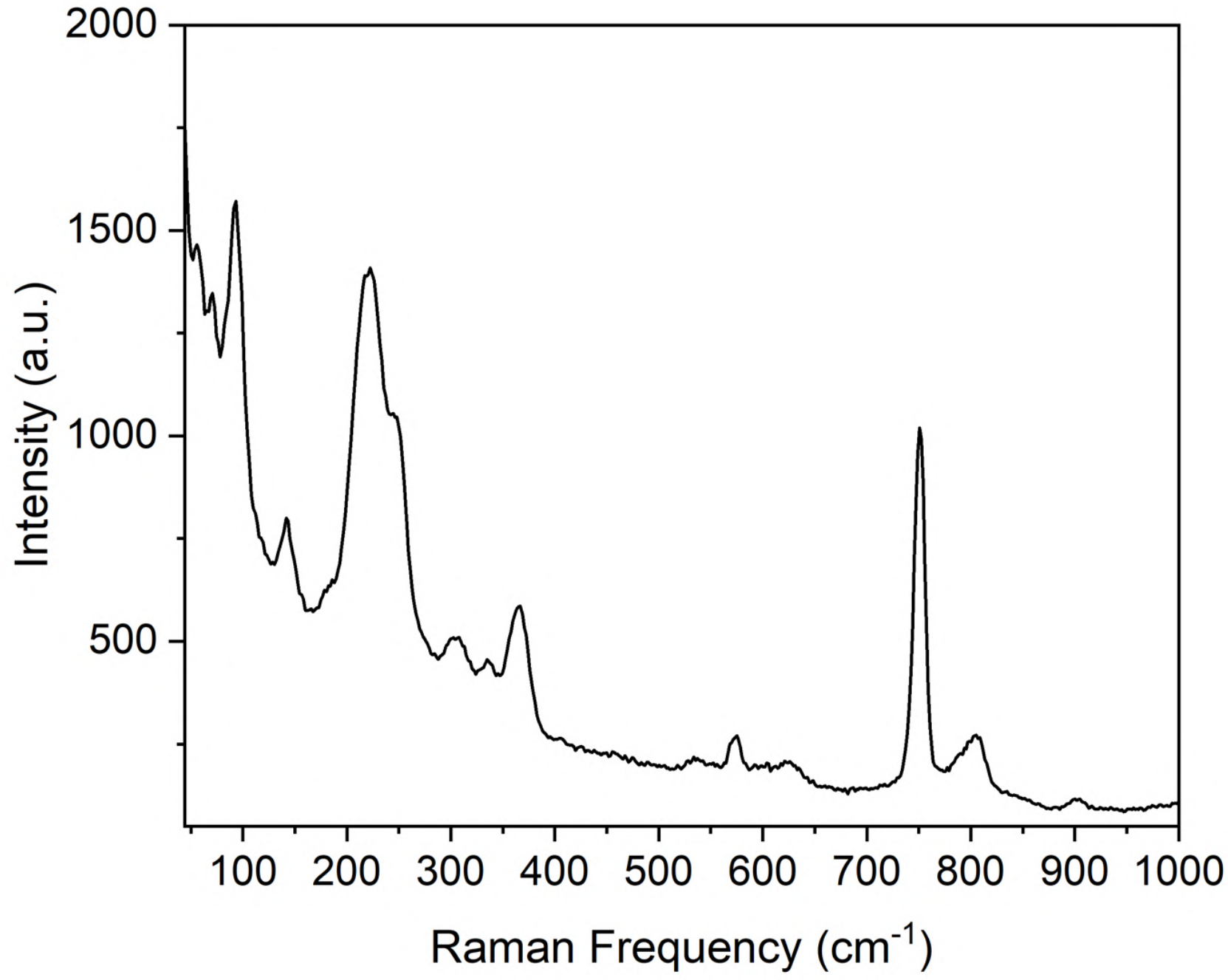

Supplementary Figure 85. Raman spectrum of **4Cs** using a 785 nm laser zoomed in over the range 30–1000 cm^{-1}.

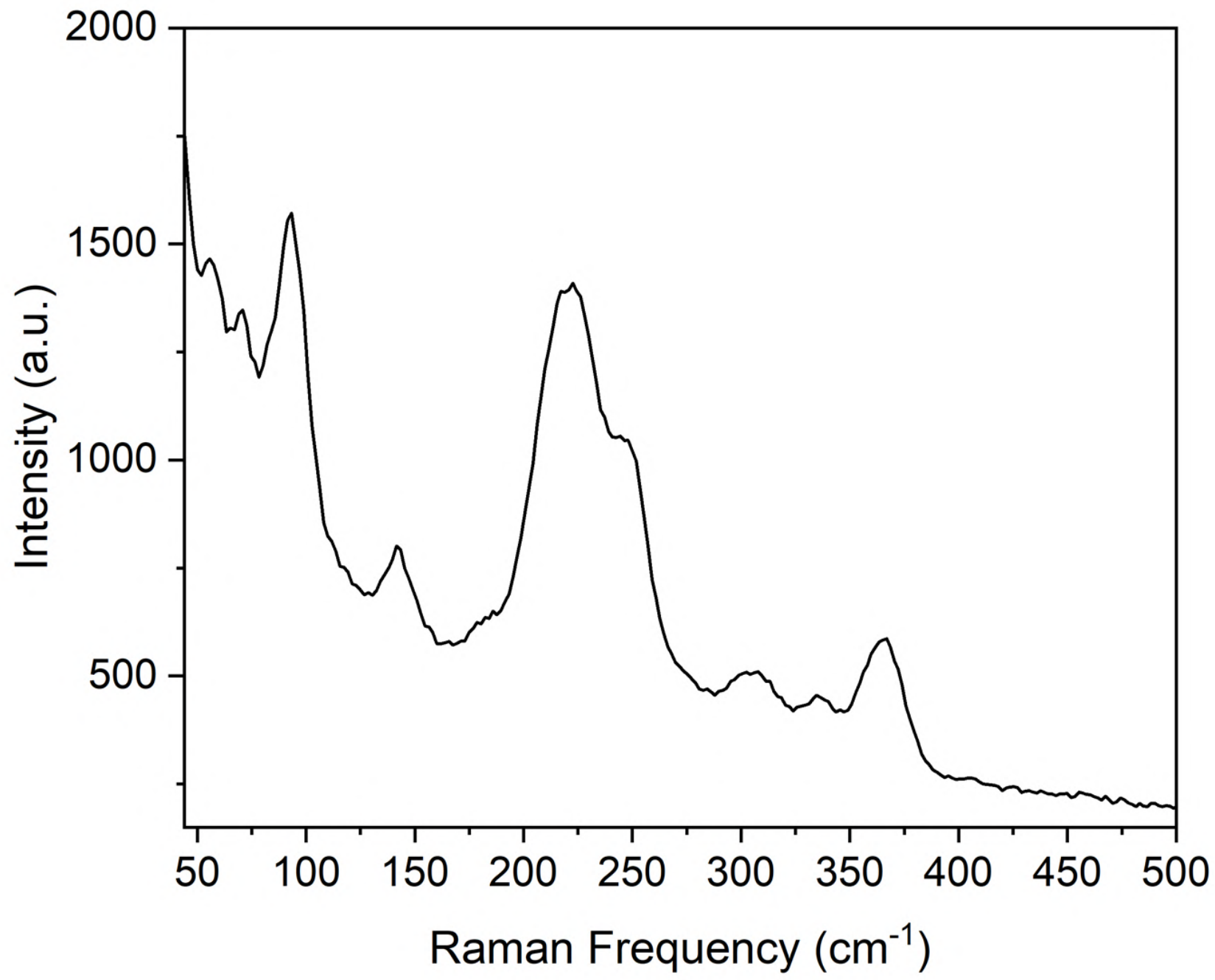

Supplementary Figure 86. Raman spectrum of **4Cs** using a 785 nm laser zoomed in over the range 30–500 cm^{-1}.

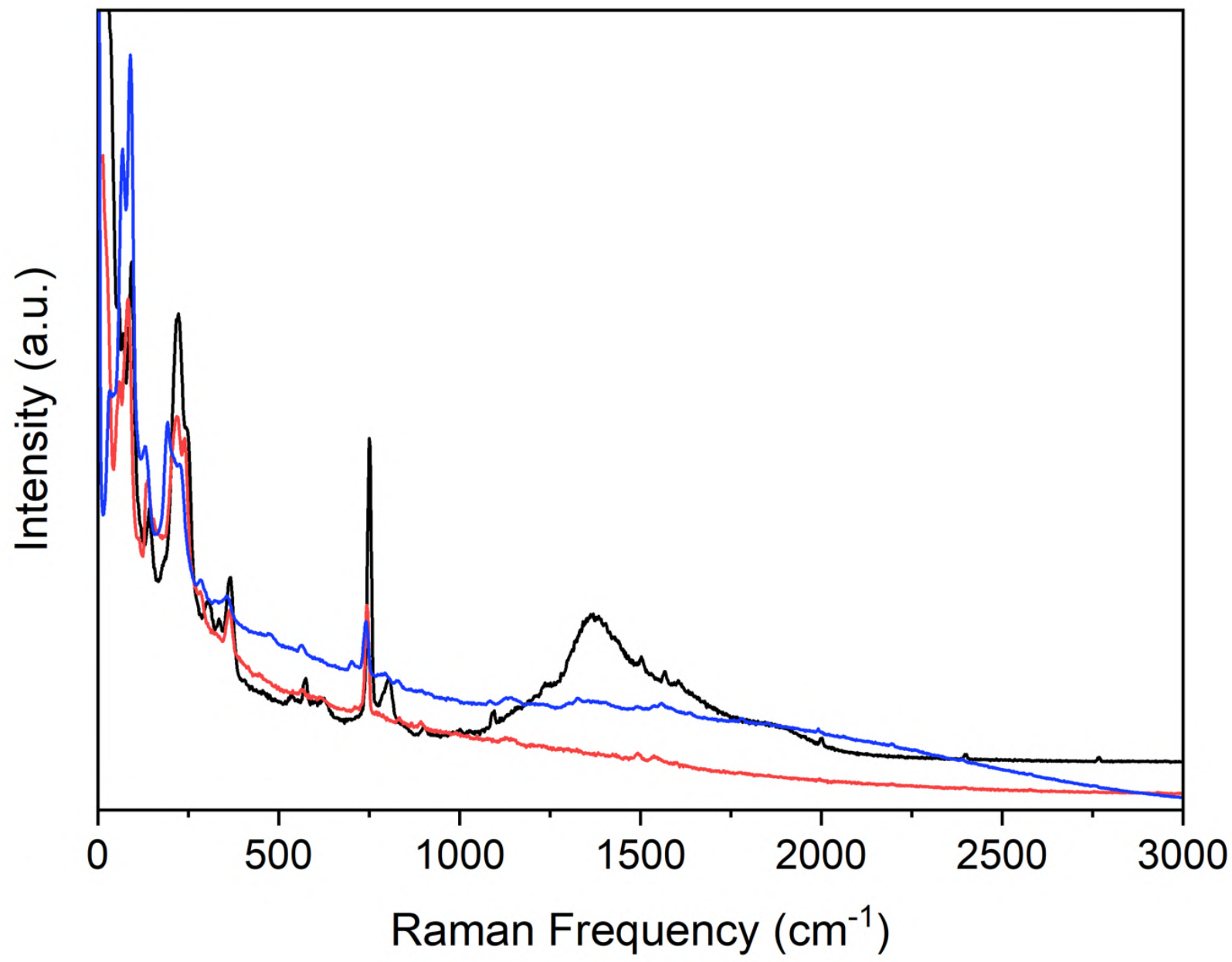

Supplementary Figure 87. Stacked Raman spectra of **4K** (blue), **4aRb** (red), and **4Cs** (black) using a 785 nm laser over the range 30–3000 cm^{-1}.

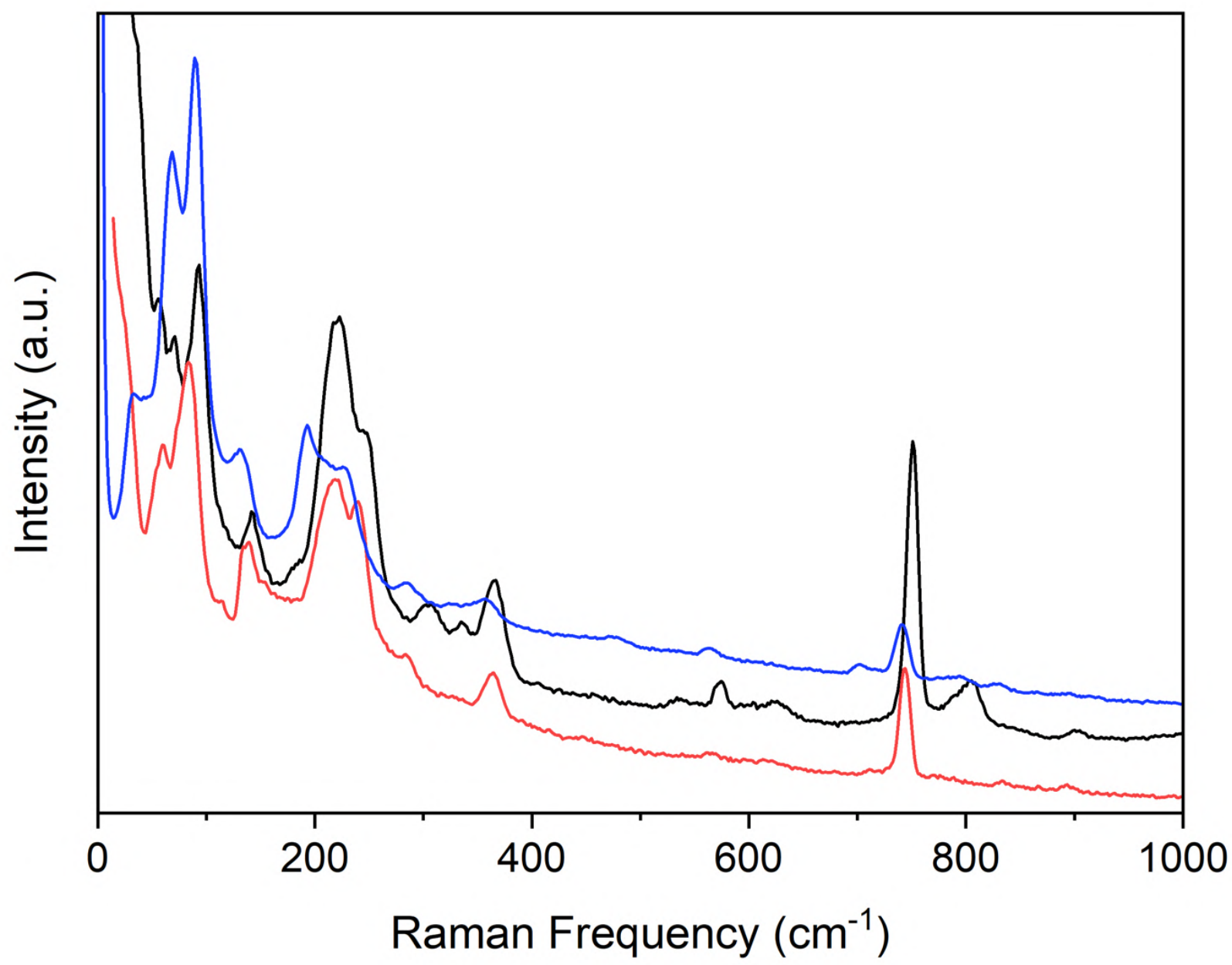

Supplementary Figure 88. Zoom-in of stacked Raman spectra of **4K** (blue), **4aRb** (red), and **4Cs** (black) using a 785 nm laser over the range 30–1000 cm^{-1}.

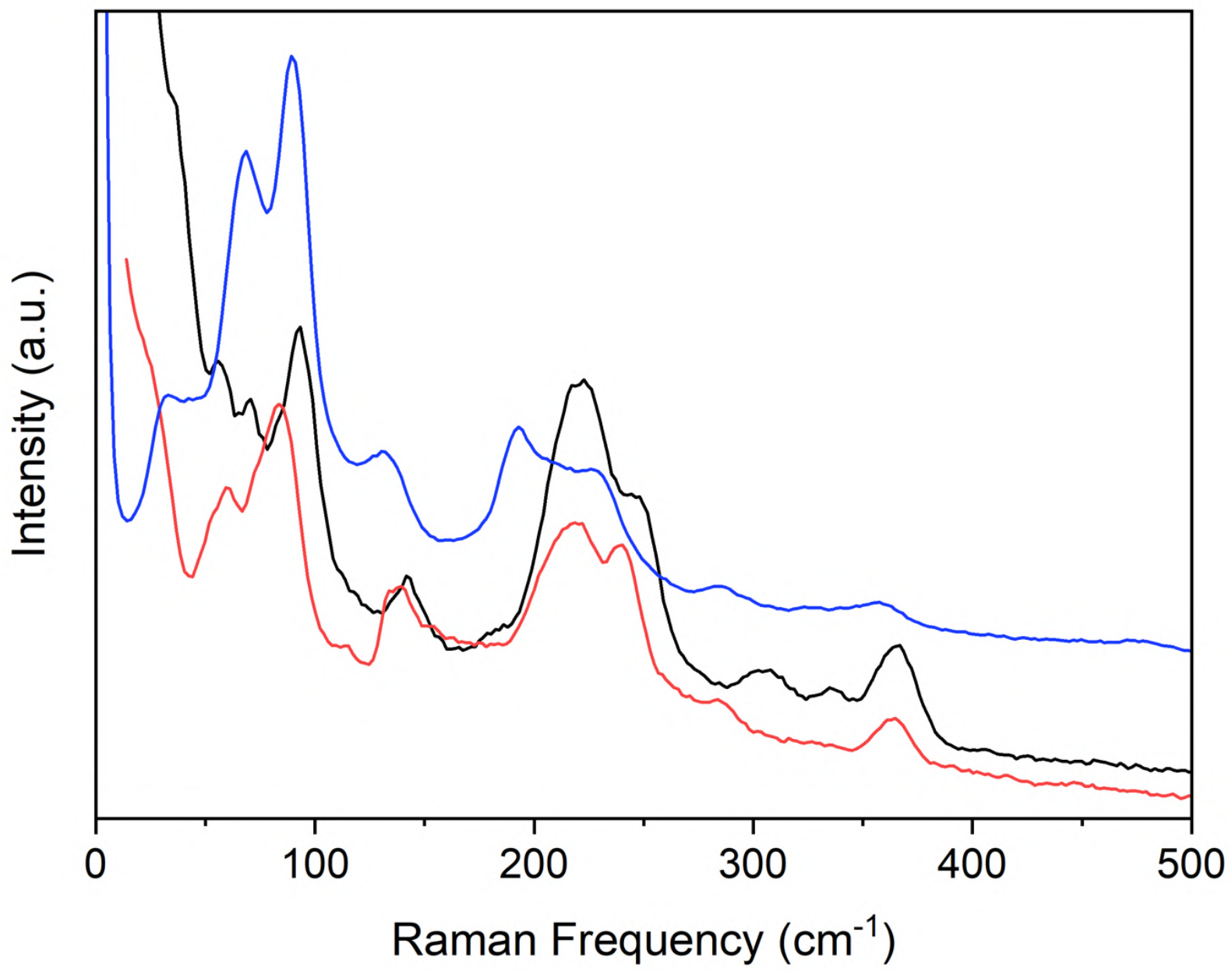

Supplementary Figure 89. Zoom-in of stacked Raman spectra of **4K** (blue), **4aRb** (red), and **4Cs** (black) using a 785 nm laser over the range 30–500 cm^{-1}.

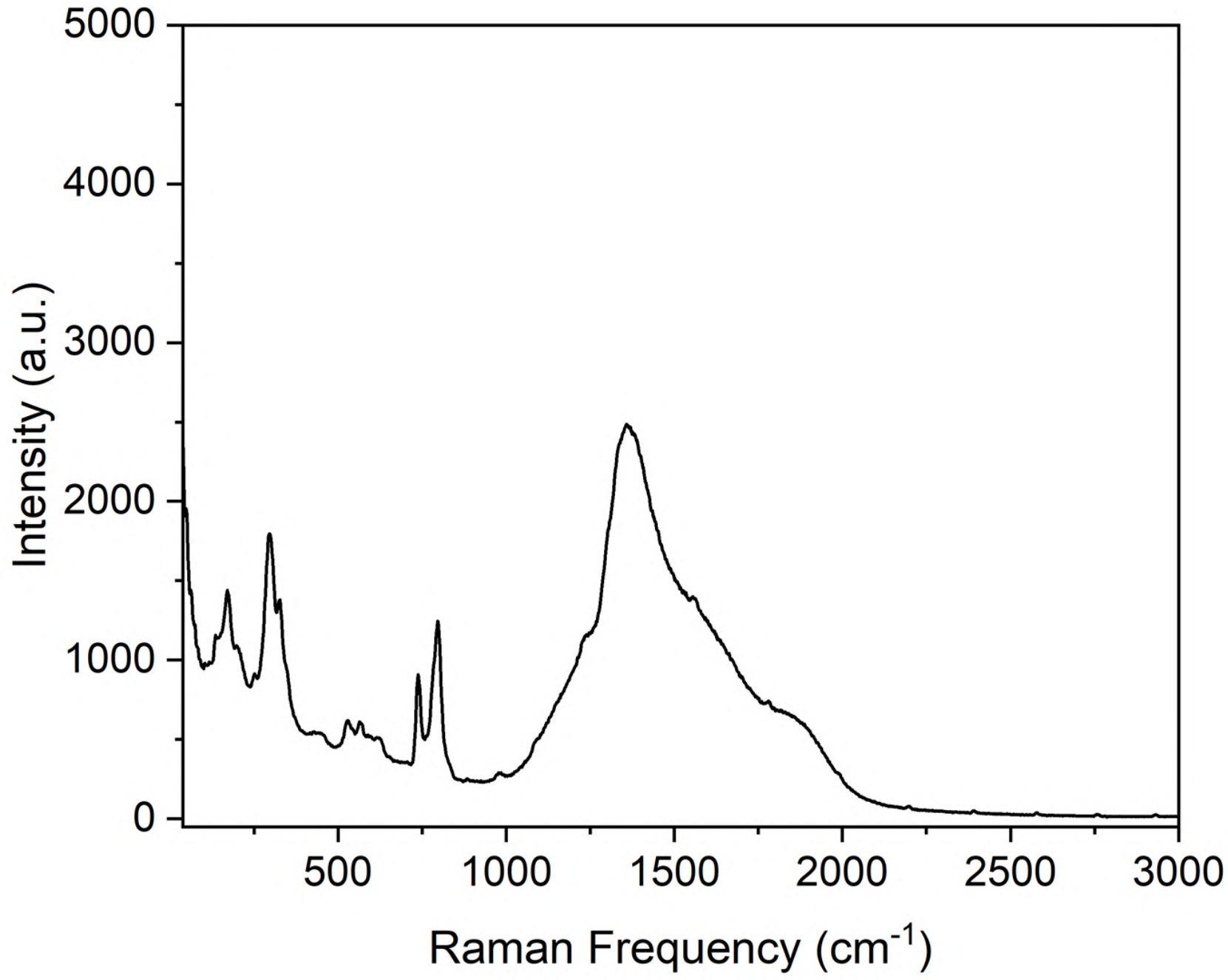

Supplementary Figure 90. Raman spectrum of **5Na** using a 785 nm laser over the range 30–3000 cm^{-1}. The large broad feature at ~1400 cm^{-1} results from photoluminescence of the glass microscope slide used for the measurement. Further details are provided in the Supplementary Text.

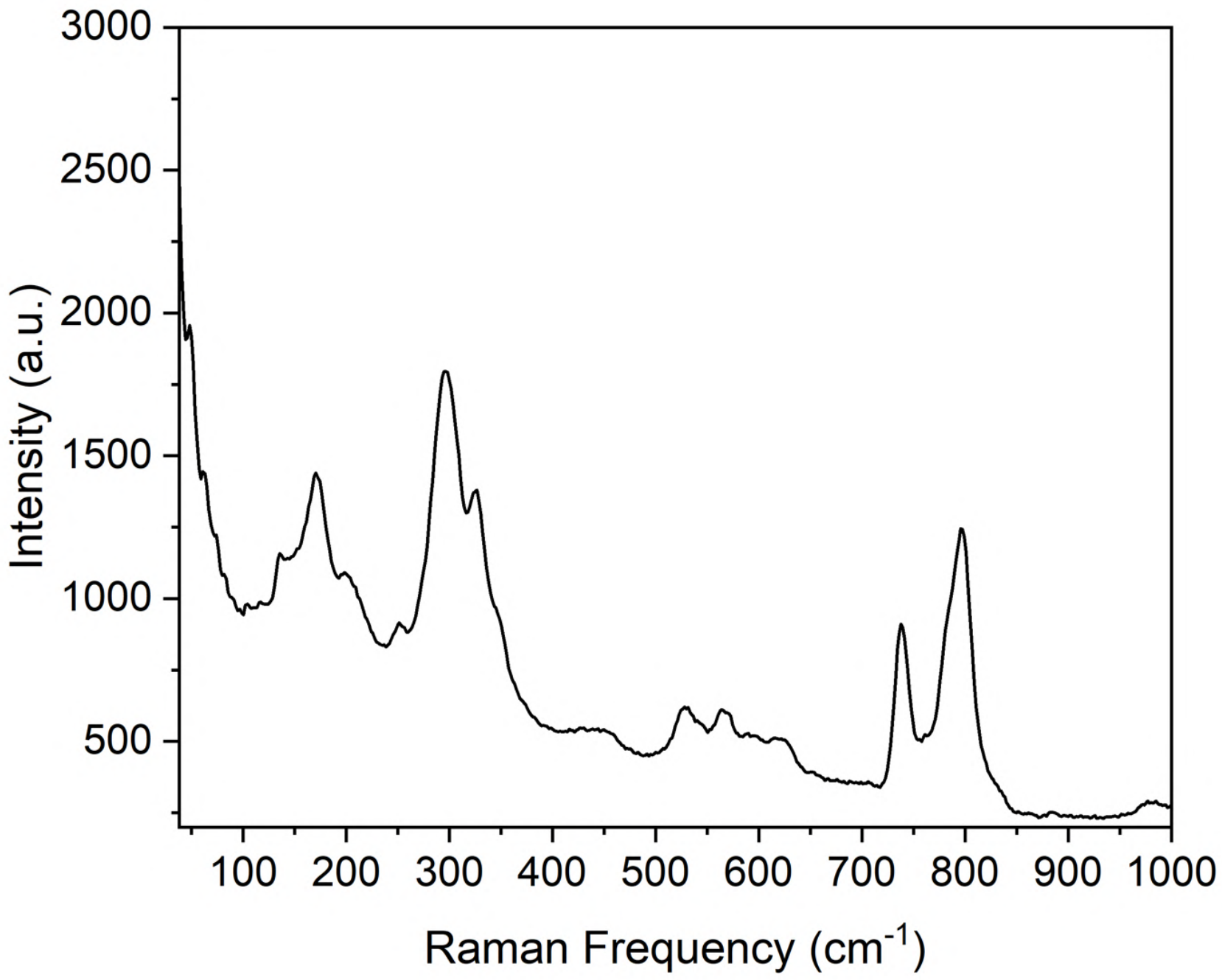

Supplementary Figure 91. Raman spectrum of **5Na** using a 785 nm laser zoomed in over the range 30–1000 cm^{-1}.

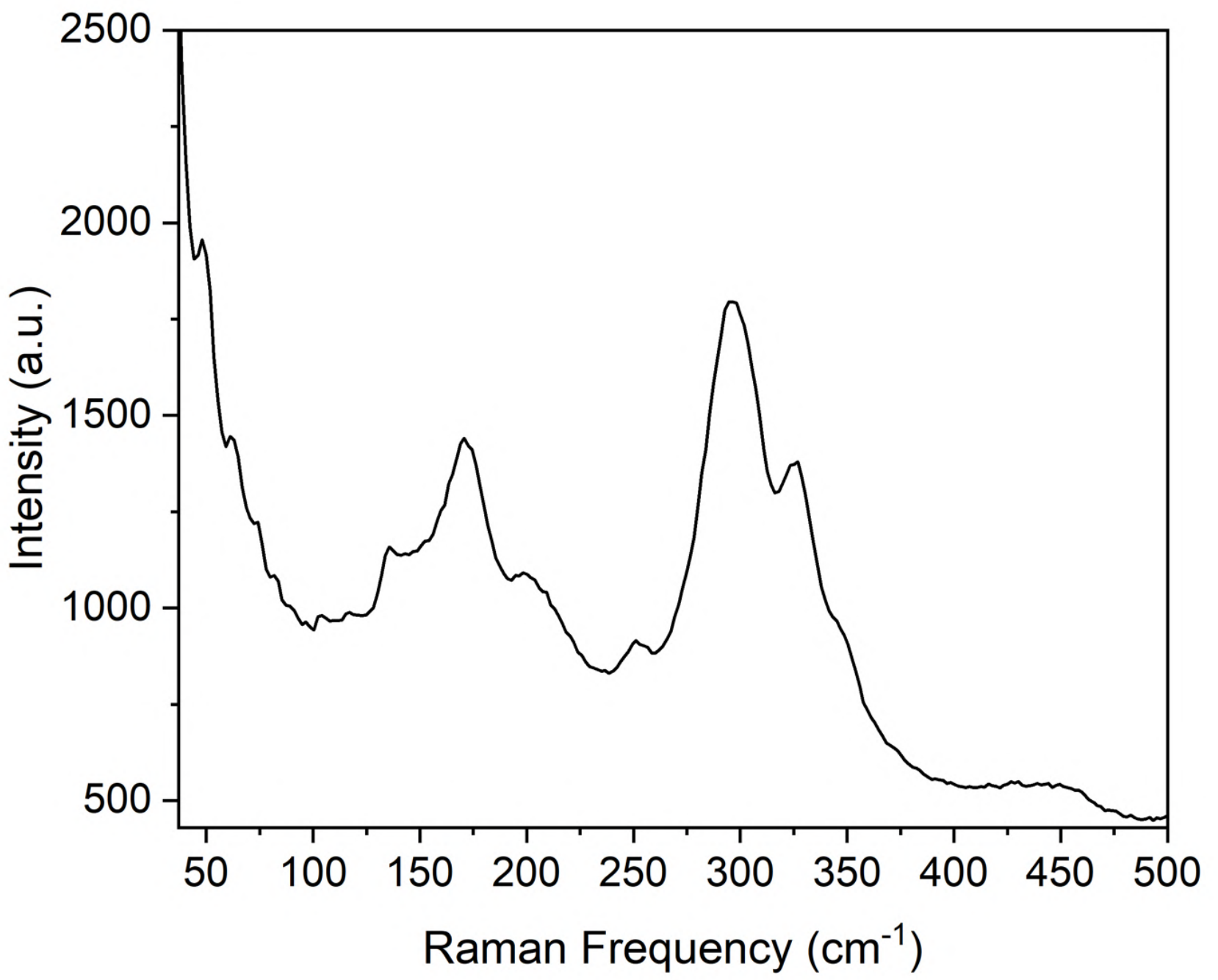

Supplementary Figure 92. Raman spectrum of **5Na** using a 785 nm laser zoomed in over the range 30–500 cm^{-1}.

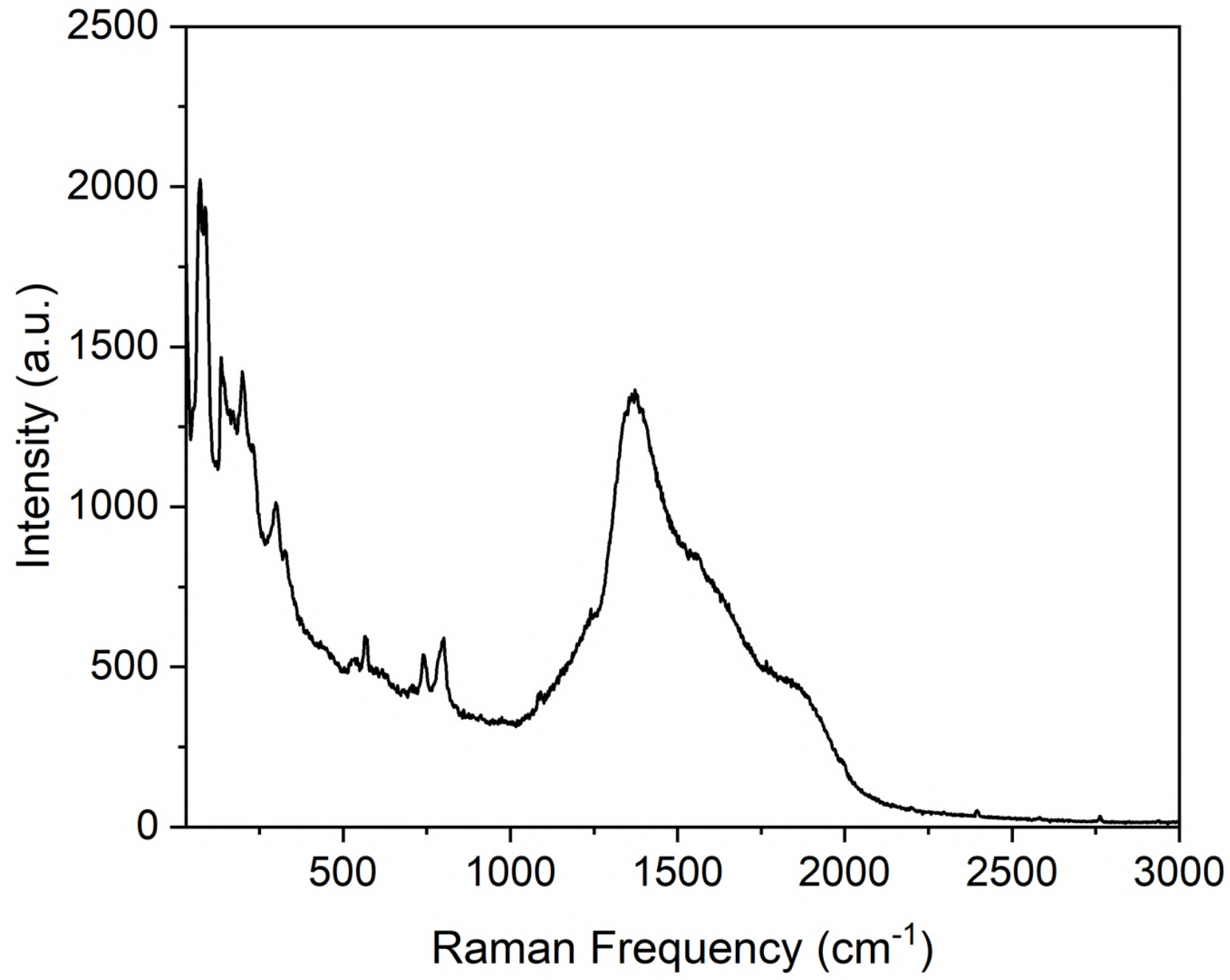

Supplementary Figure 93. Raman spectrum of **5K** using a 785 nm laser over the range 30–3000 cm^{-1}. The large broad feature at ~1400 cm^{-1} results from photoluminescence of the glass microscope slide used for the measurement. Further details are provided in the Supplementary Text.

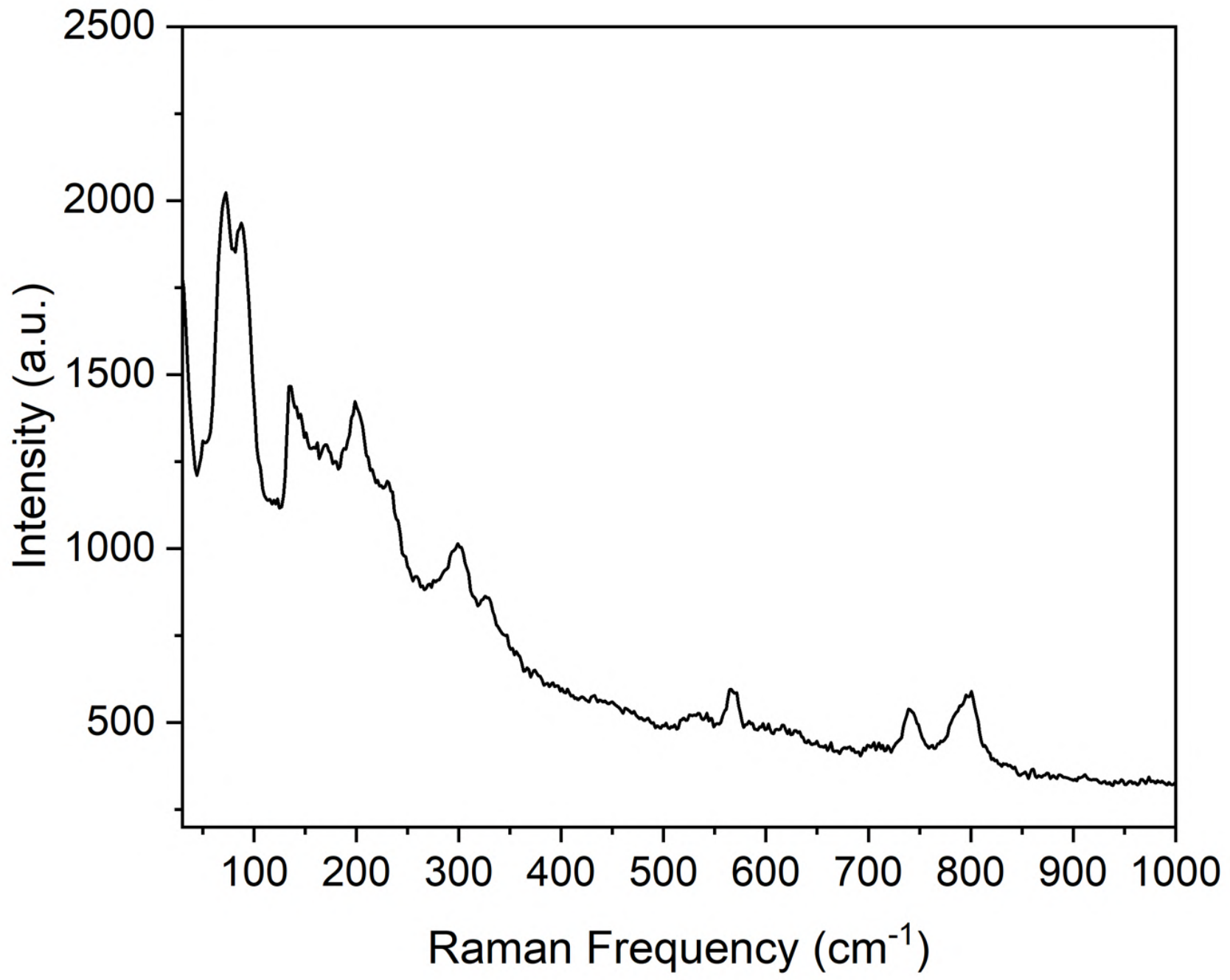

Supplementary Figure 94. Raman spectrum of **5K** using a 785 nm laser zoomed in over the range 30–1000 cm^{-1}.

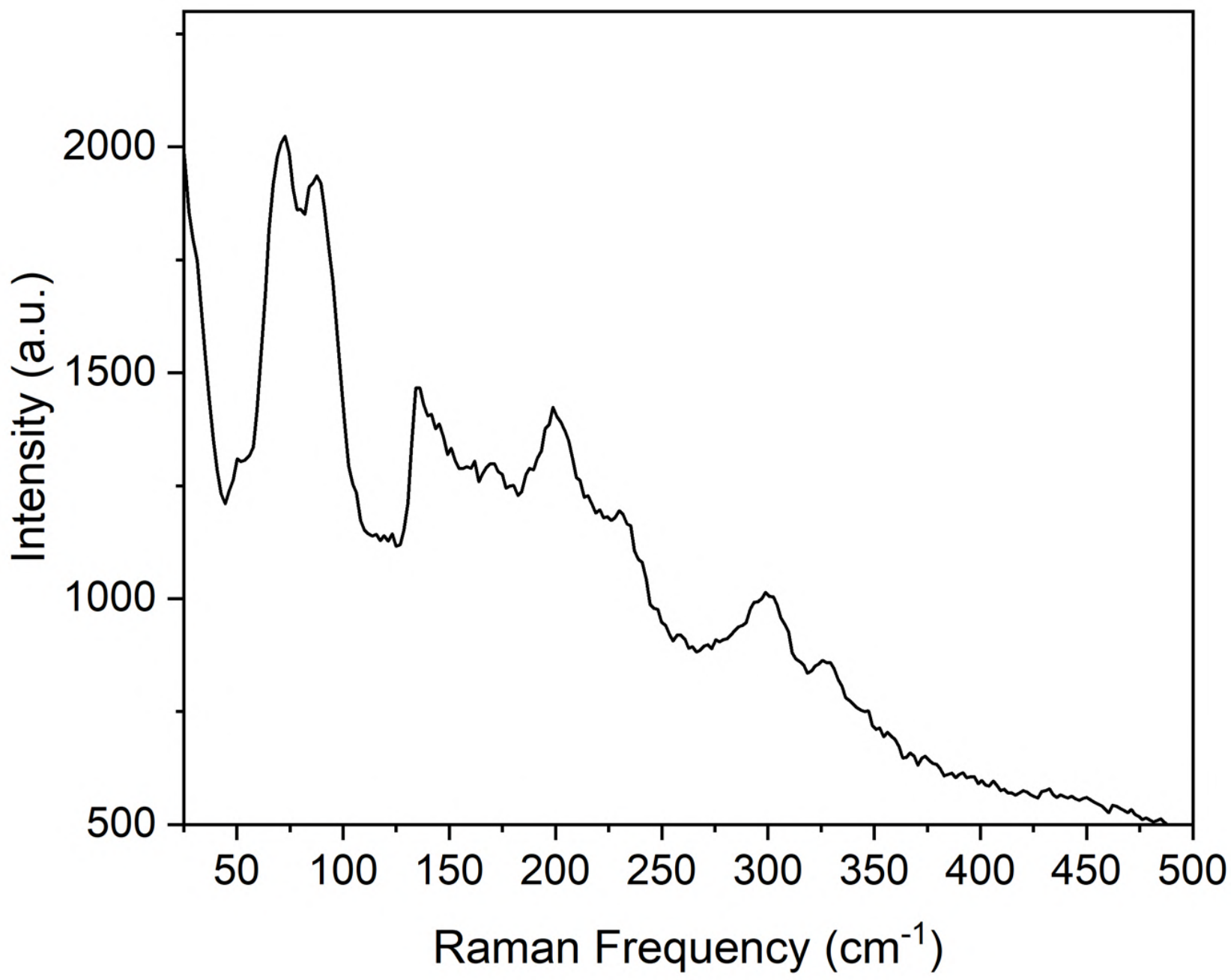

Supplementary Figure 95. Raman spectrum of **5K** using a 785 nm laser zoomed in over the range 30–500 cm^{-1}.

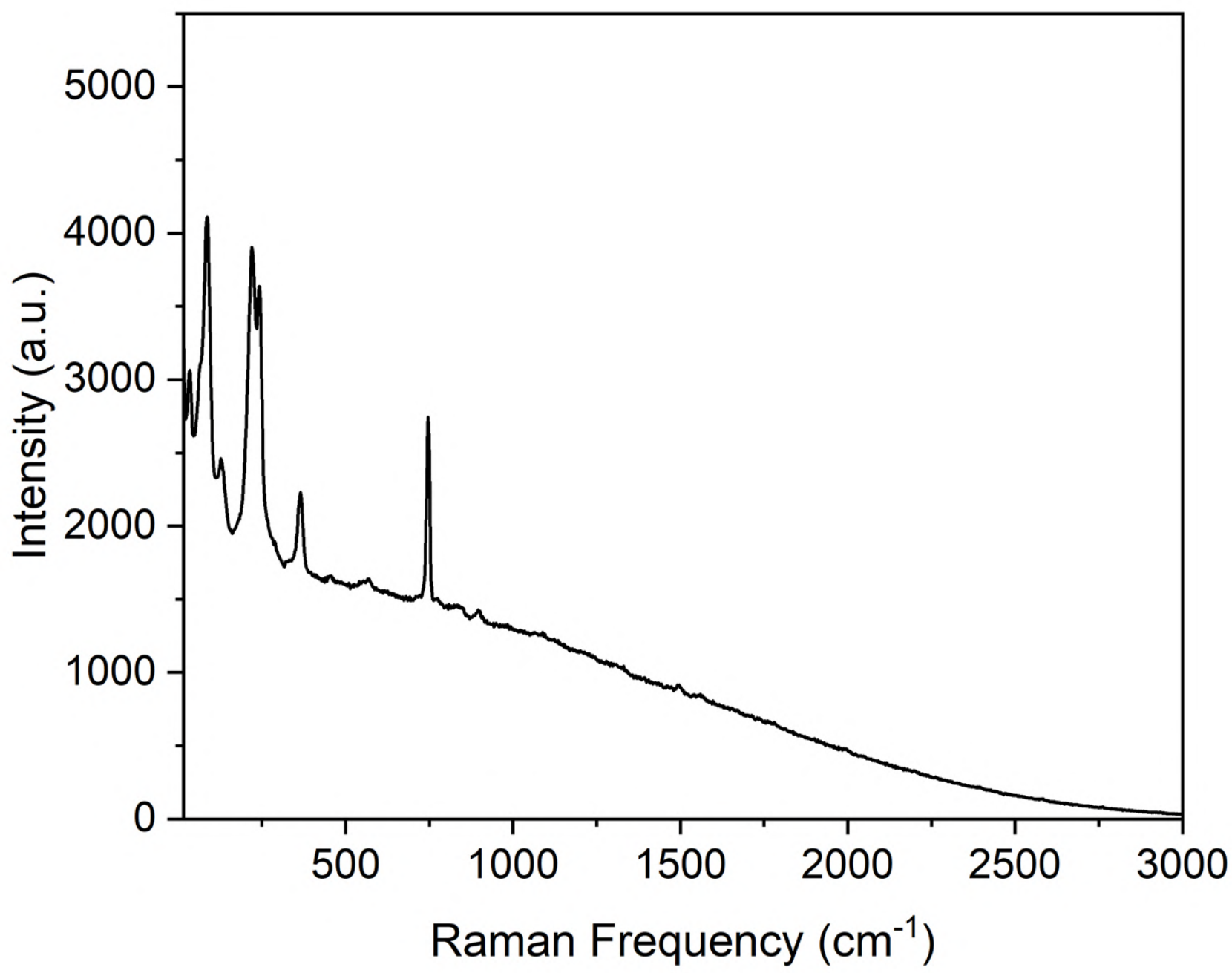

Supplementary Figure 96. Raman spectrum of **5Rb** using a 785 nm laser over the range 30–3000 cm^{-1}.

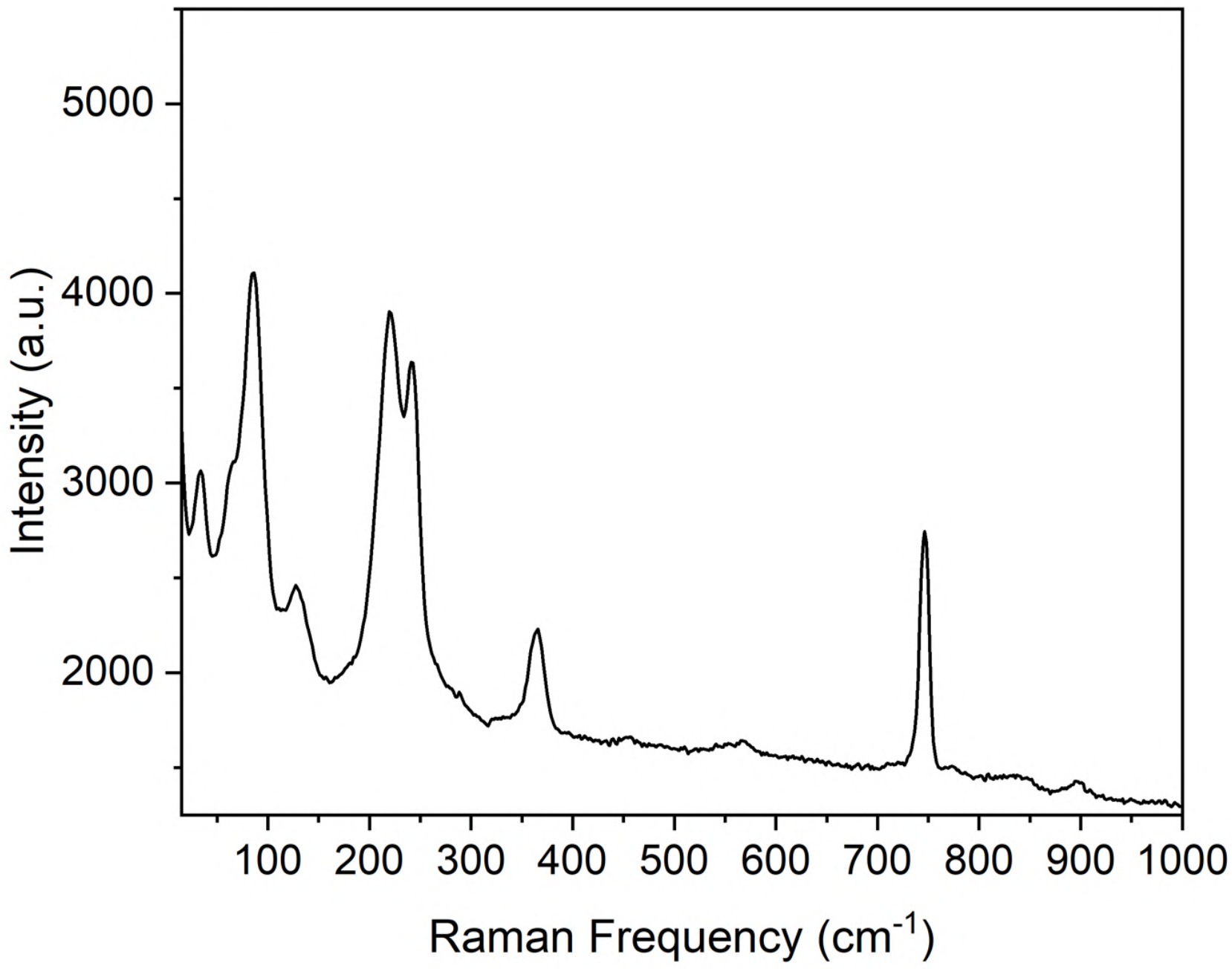

Supplementary Figure 97. Raman spectrum of **5Rb** using a 785 nm laser zoomed in over the range 30–1000 cm^{-1}.

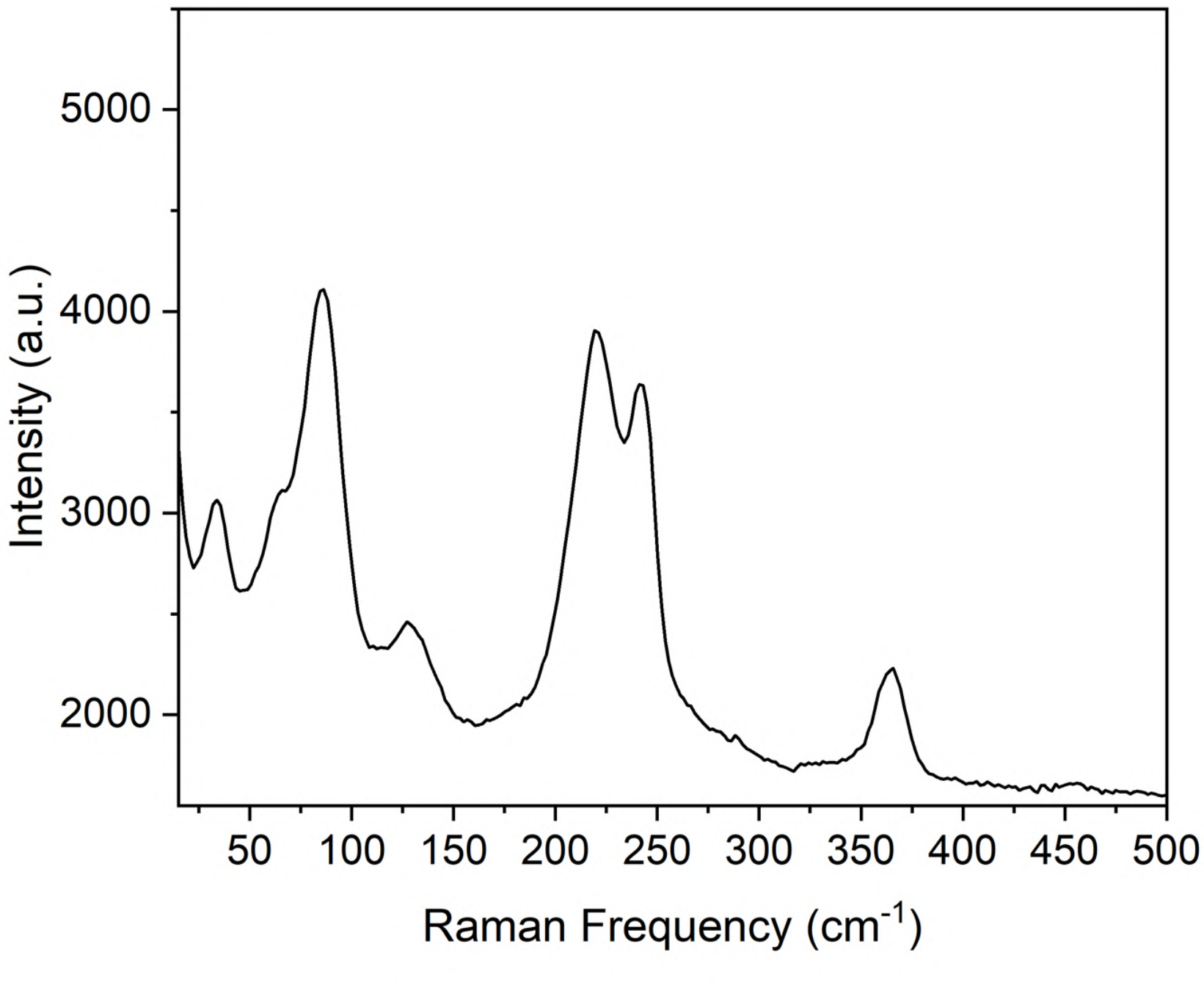

Supplementary Figure 98. Raman spectrum of **5Rb** using a 785 nm laser zoomed in over the range 30–500 cm^{-1}.

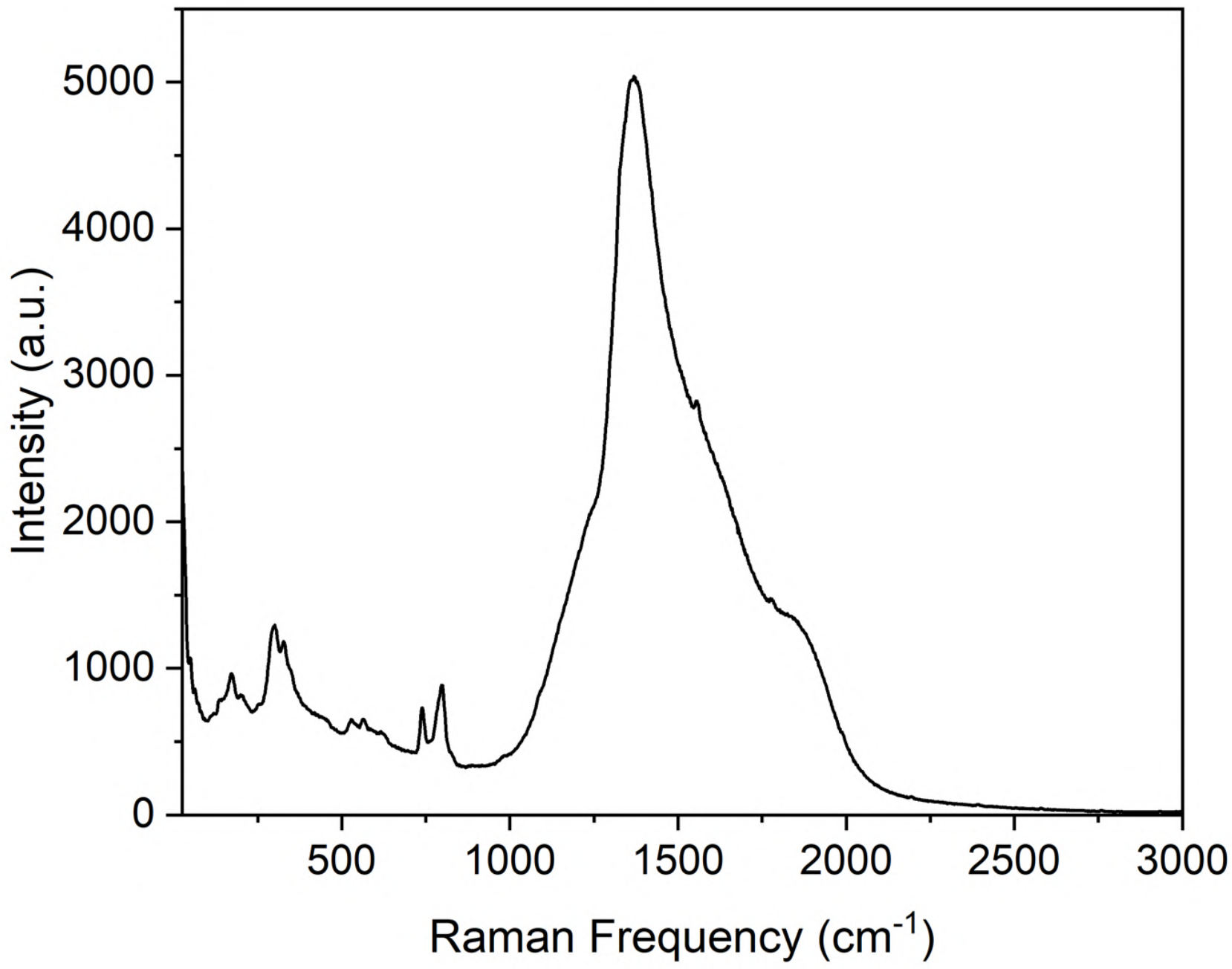

Supplementary Figure 99. Raman spectrum of **5Cs** using a 785 nm laser over the range 30–3000 cm^{-1}. The large broad feature at ~1400 cm^{-1} results from photoluminescence of the glass microscope slide used for the measurement. Further details are provided in the Supplementary Text.

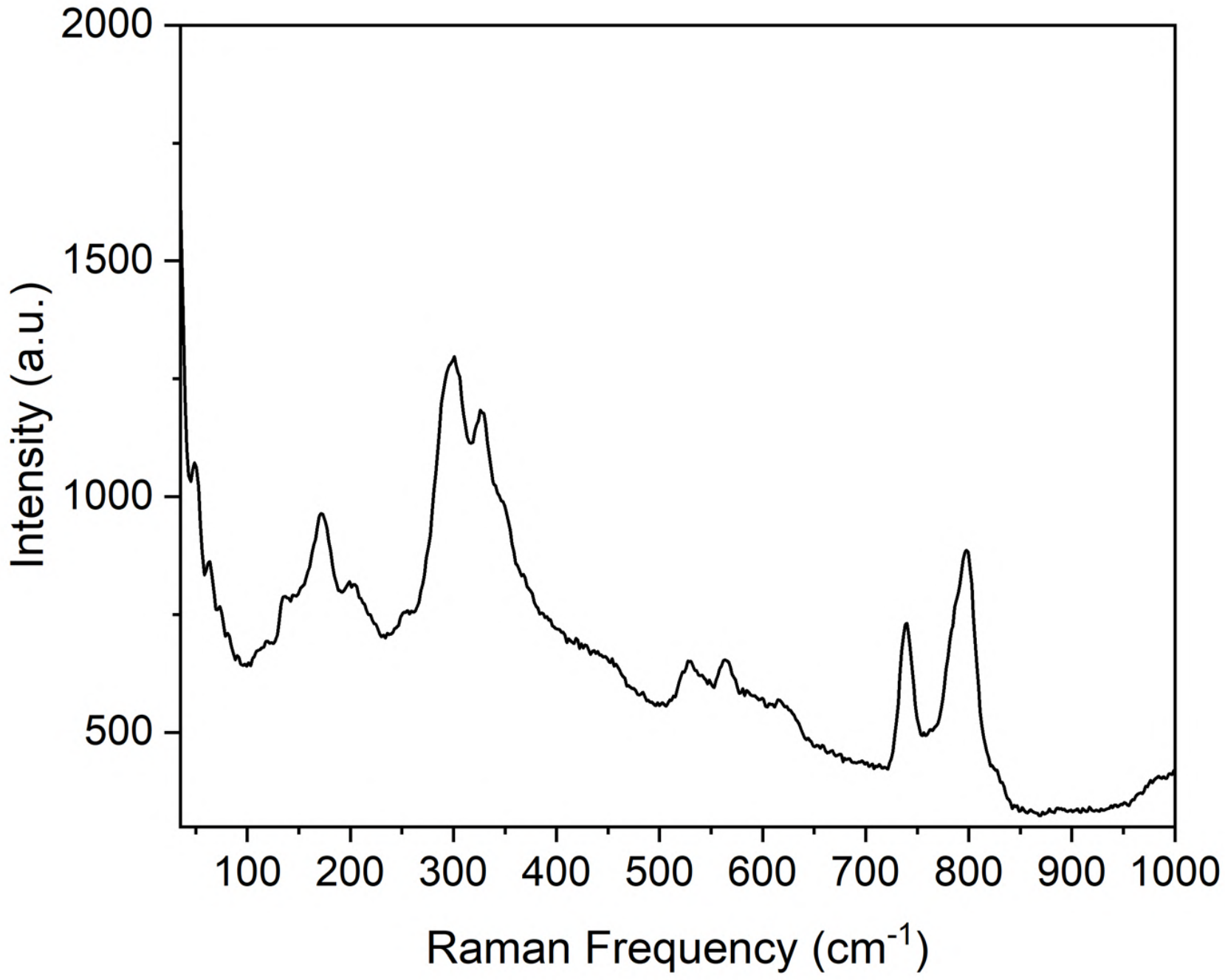

Supplementary Figure 100. Raman spectrum of **5Cs** using a 785 nm laser zoomed in over the range 30–1000 cm^{-1}.

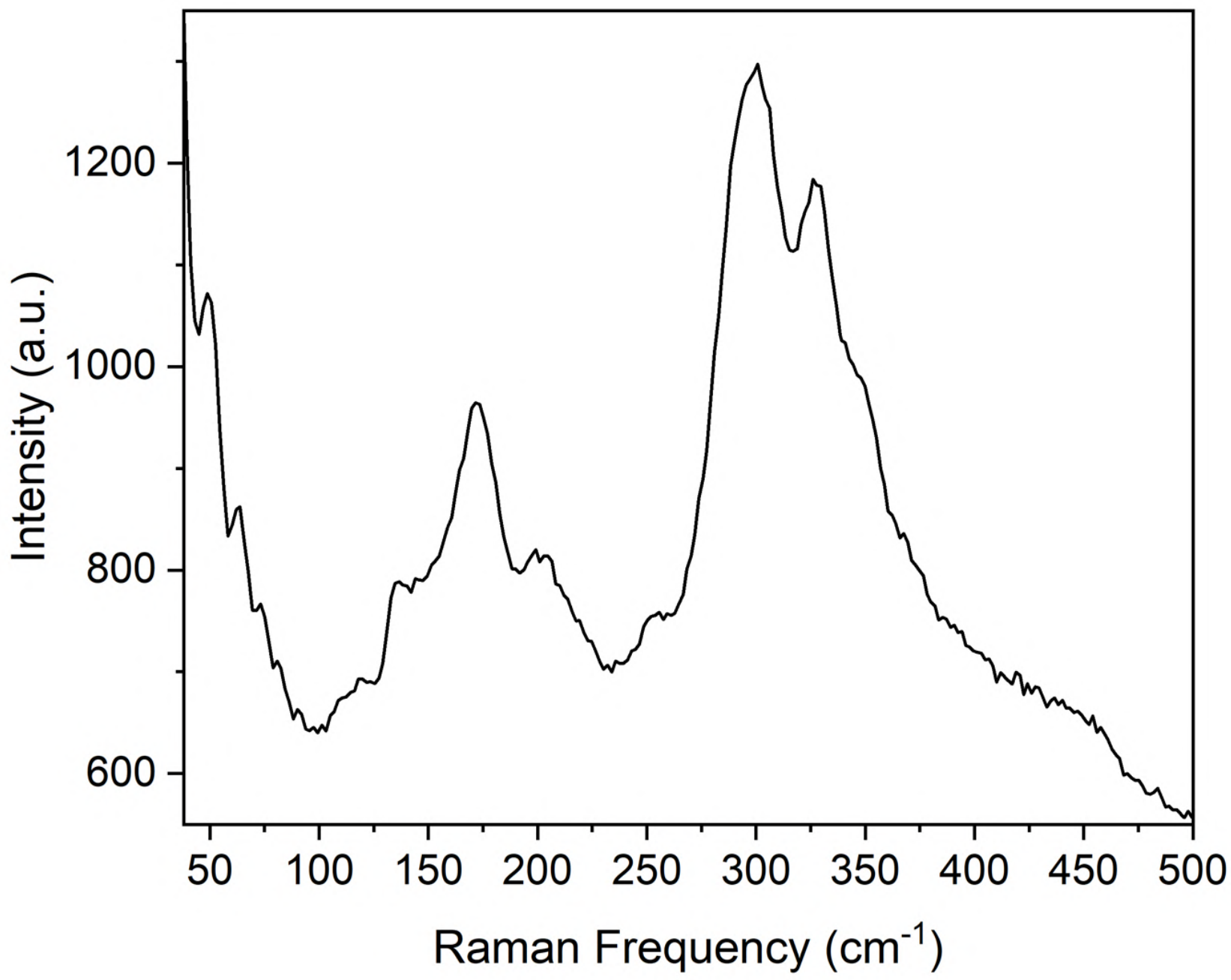

Supplementary Figure 101. Raman spectrum of **5Cs** using a 785 nm laser zoomed in over the range 30–500 cm^{-1}.

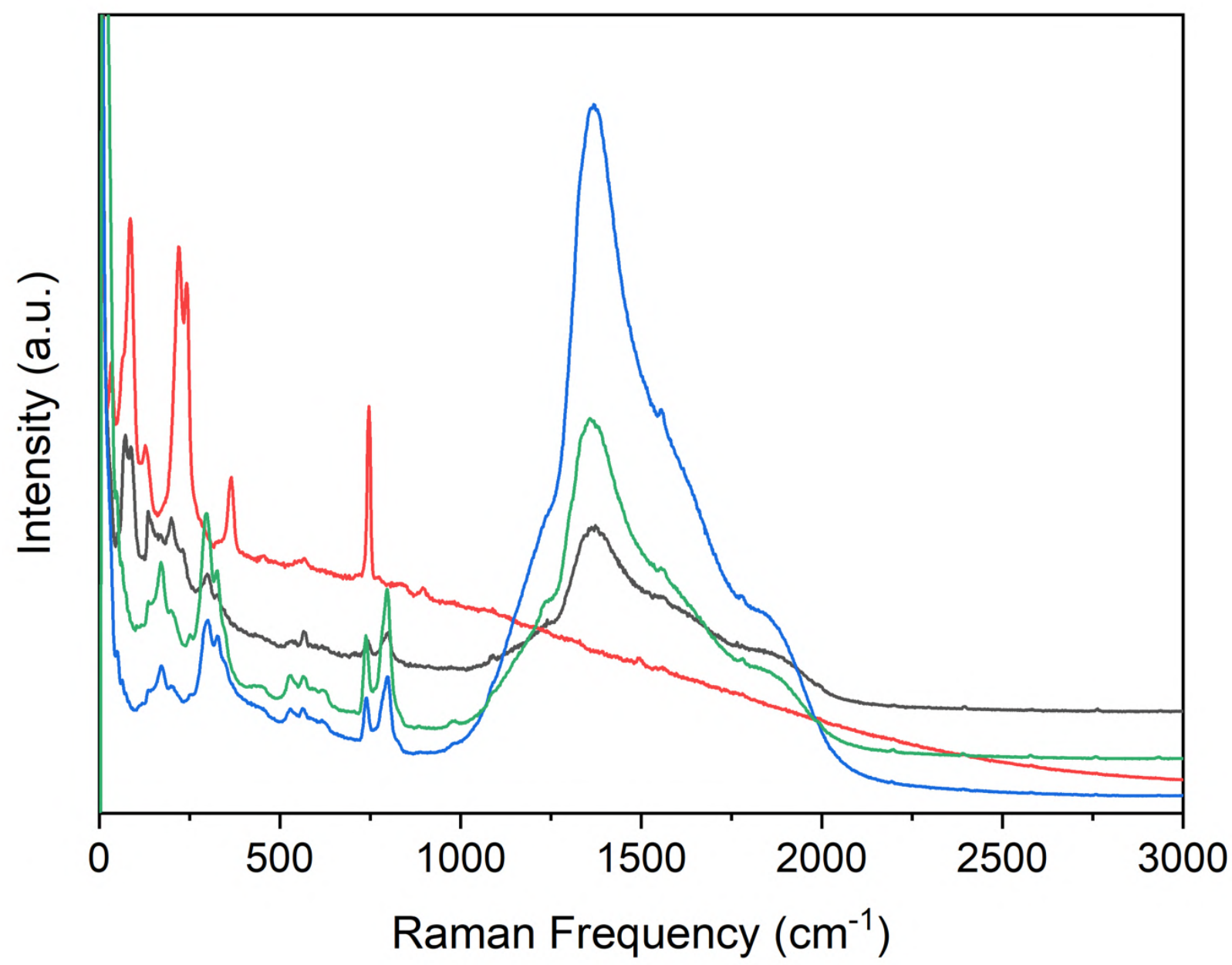

Supplementary Figure 102. Stacked Raman spectra of **5Na** (green), **4K** (black), **4aRb** (red), and **4Cs** (blue) using a 785 nm laser over the range 30–3000 cm^{-1}.

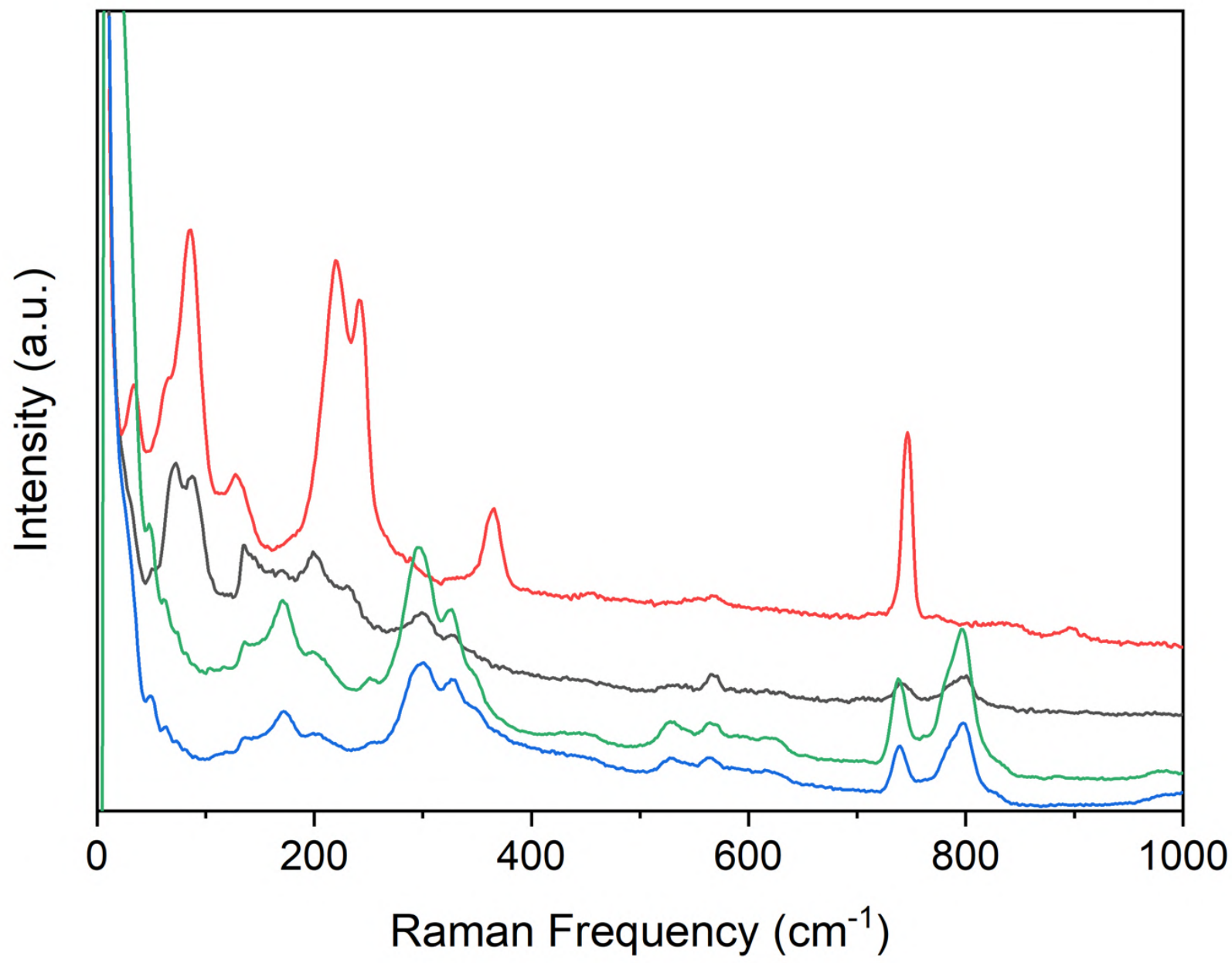

Supplementary Figure 103. Zoom-in of stacked Raman spectra of **5Na** (green), **4K** (black), **4aRb** (red), and **4Cs** (blue) using a 785 nm laser over the range 30–1000 cm^{-1}.

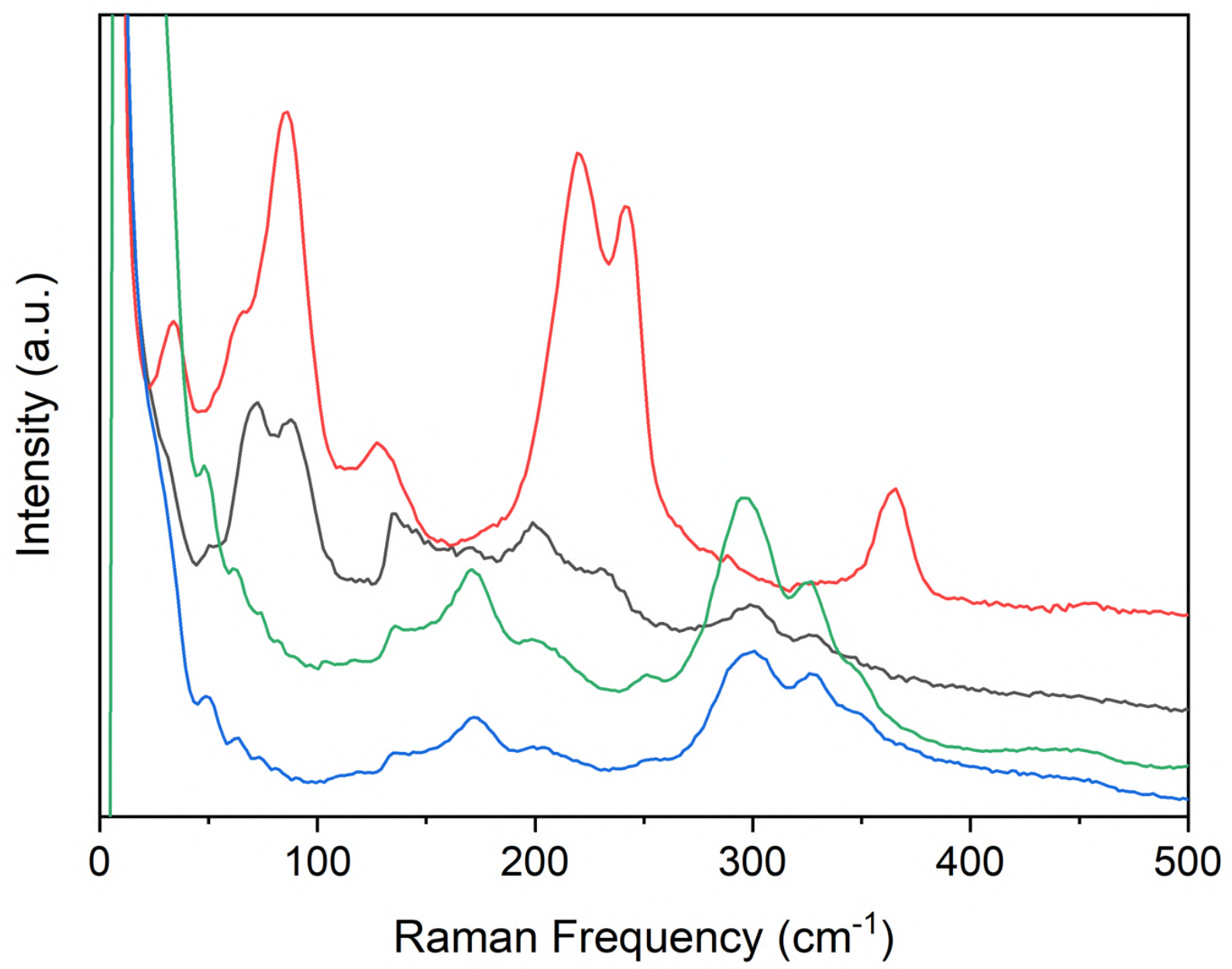

Supplementary Figure 104. Zoom-in of stacked Raman spectra of **5Na** (green), **5K** (black), **5Rb** (red), and **5Cs** (blue) using a 785 nm laser over the range 30–500 cm^{-1}.

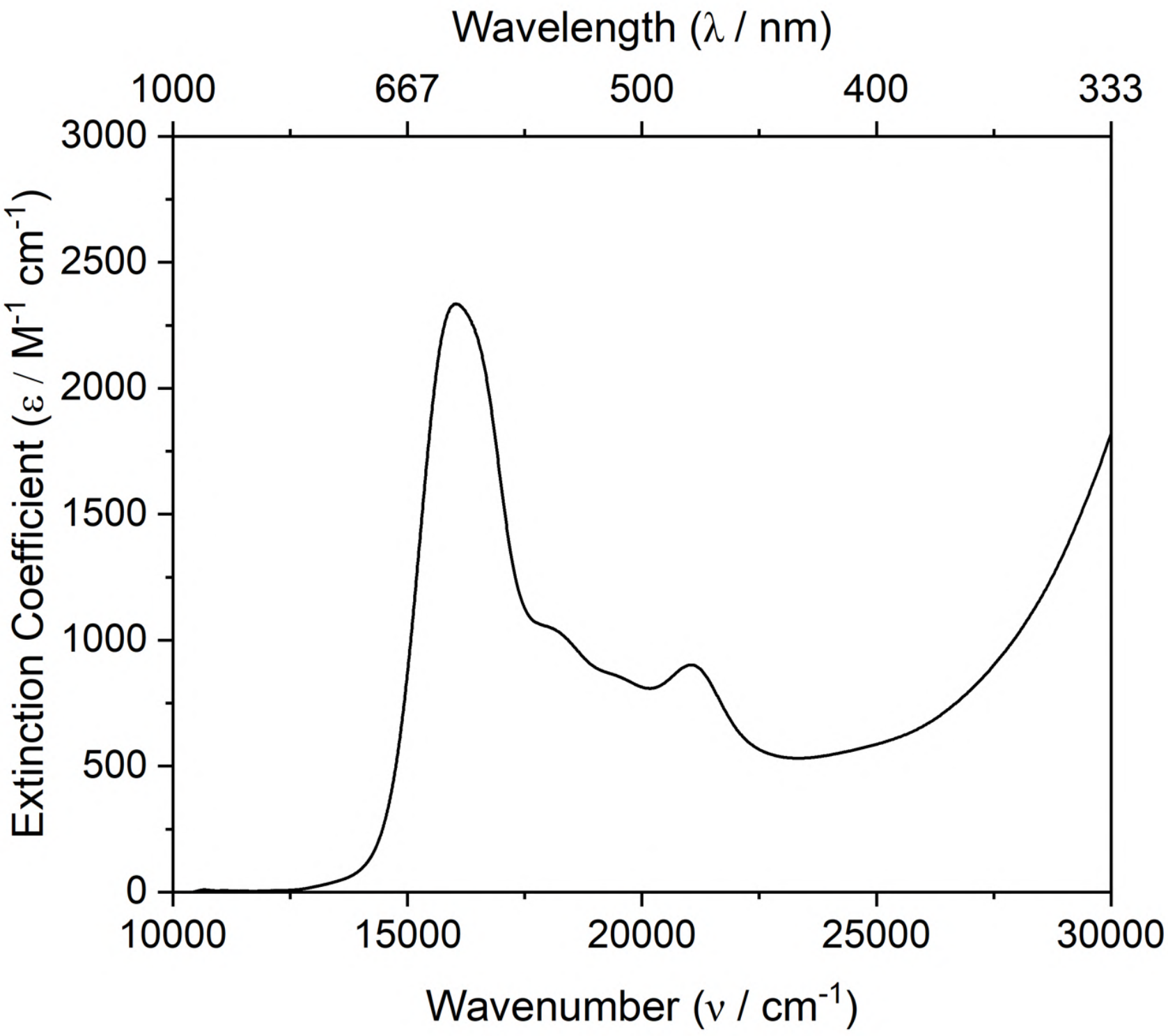

Supplementary Figure 105. UV/Vis/NIR spectrum of **4K** (0.108 mM in DME).

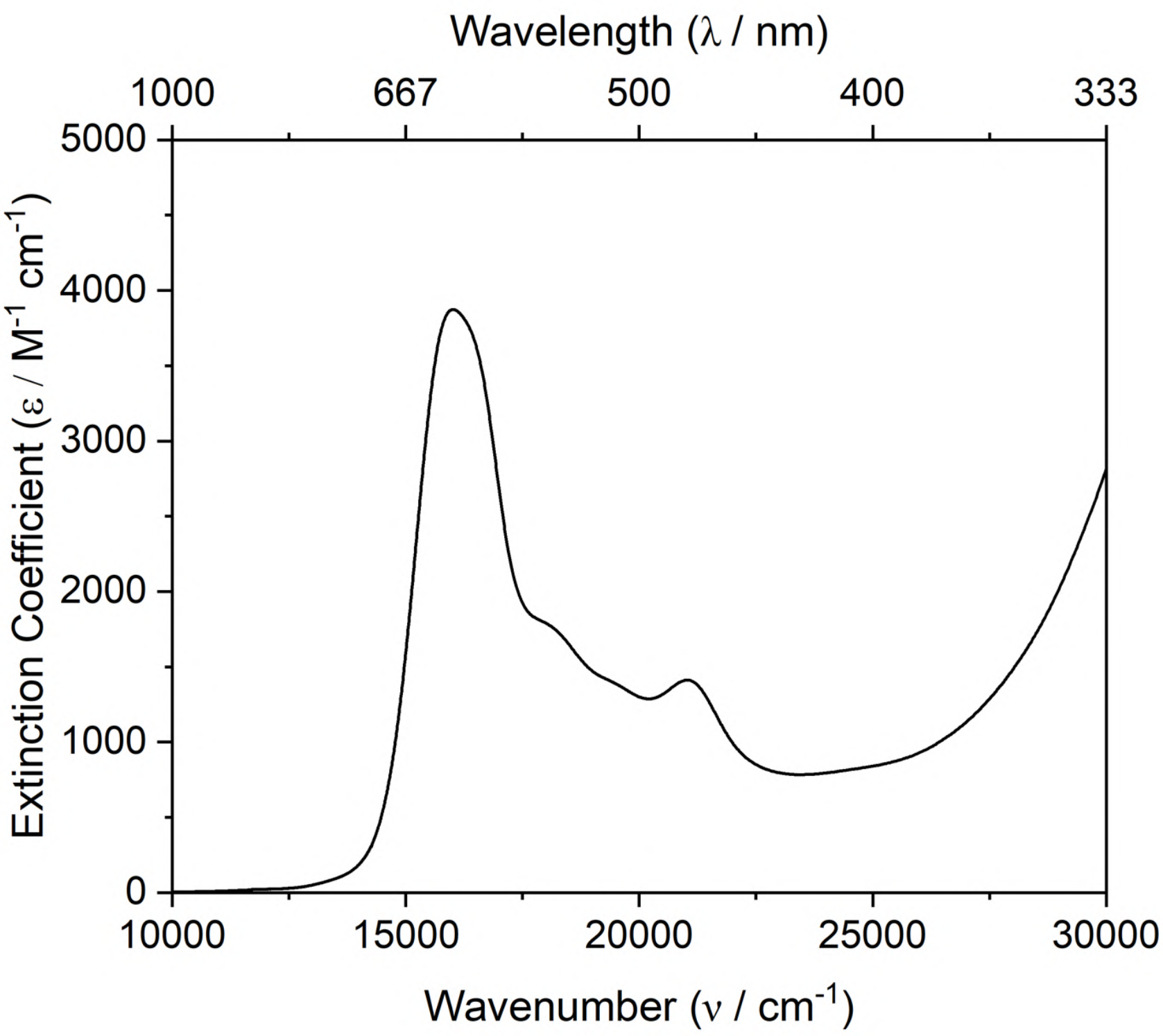

Supplementary Figure 106. UV/Vis/NIR spectrum of **4aRb** (0.334 mM in DME).

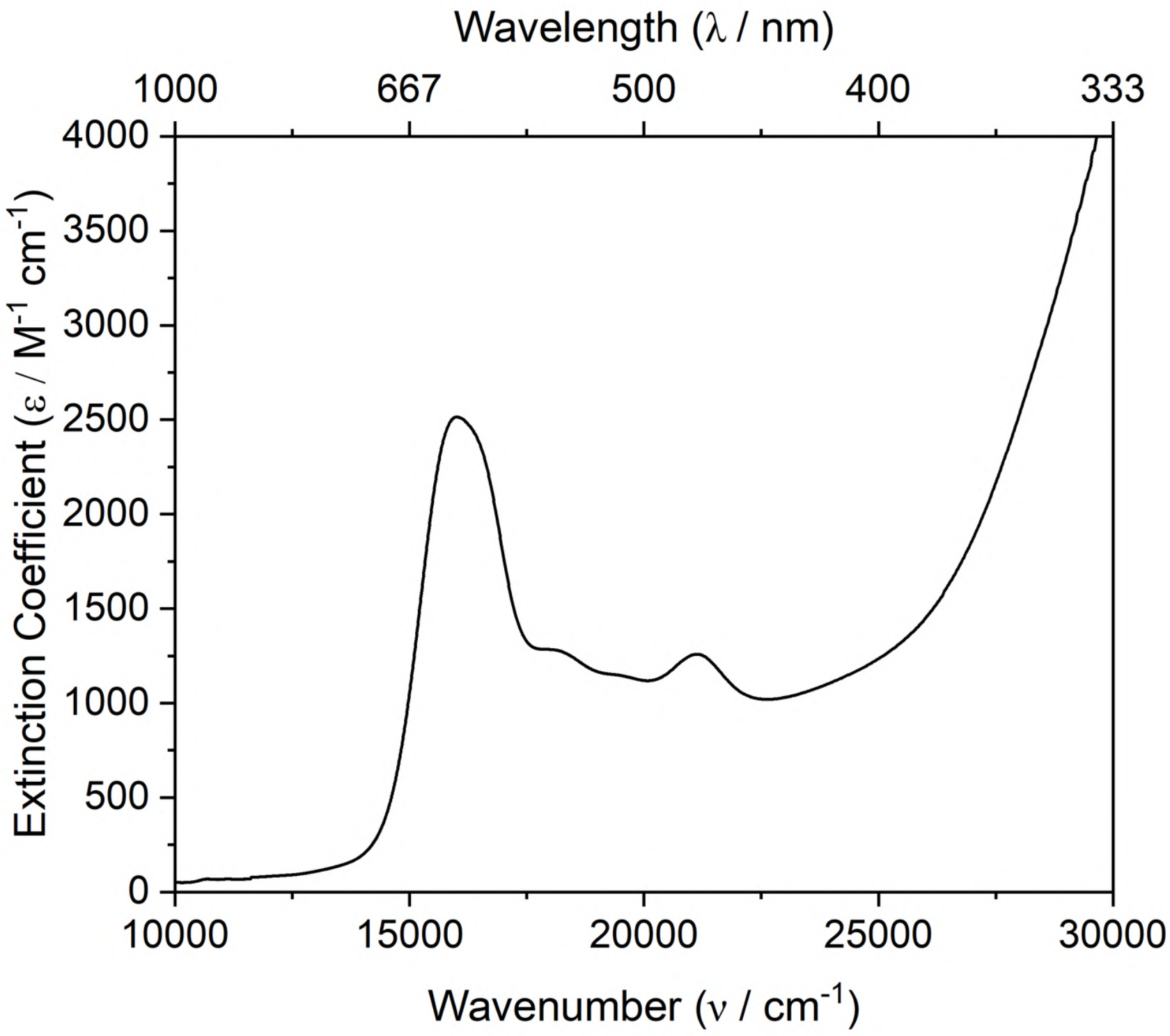

Supplementary Figure 107. UV/Vis/NIR spectrum of **4Cs** (0.124 mM in DME).

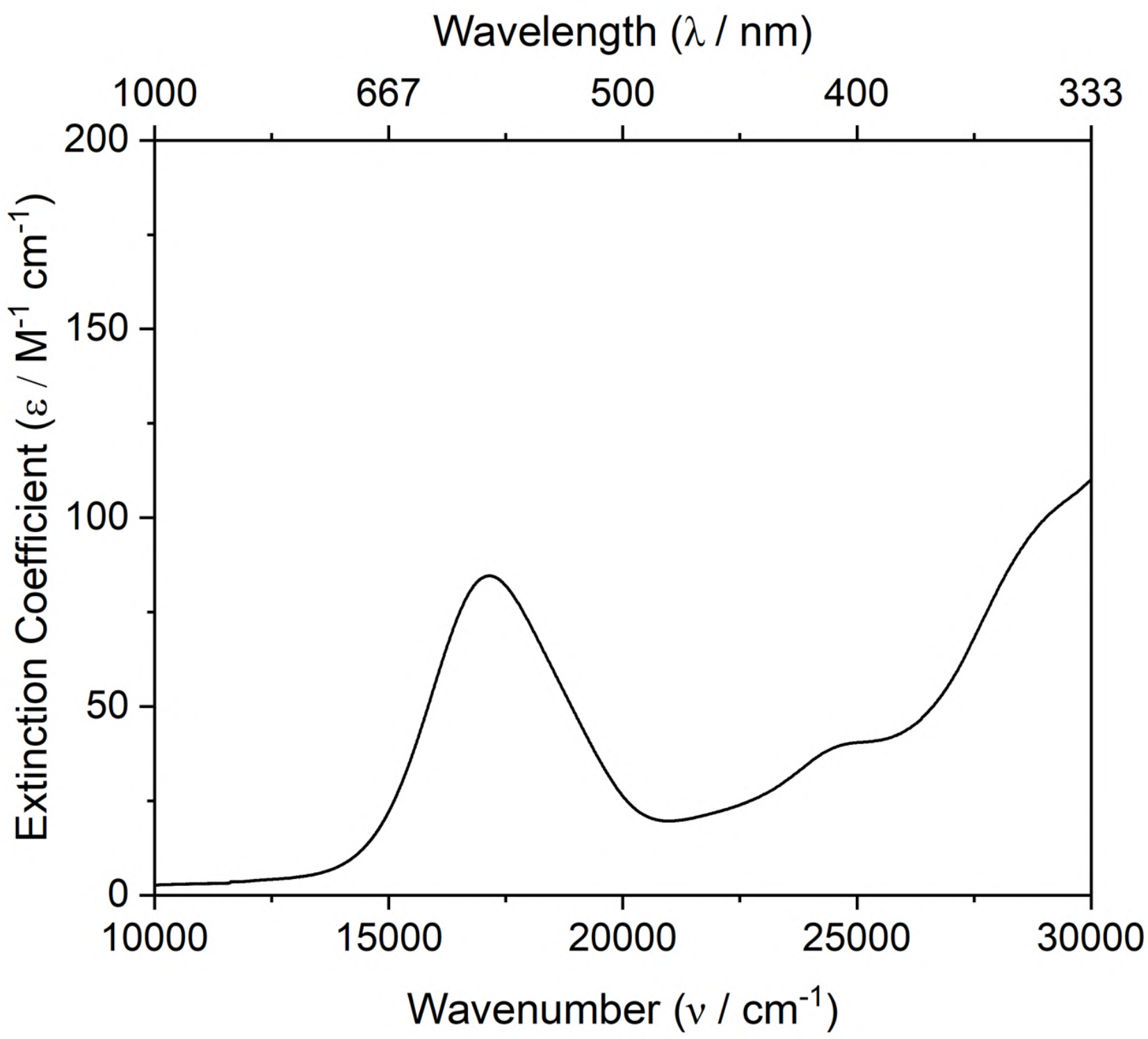

Supplementary Figure 108. UV/Vis/NIR spectrum of **5Na** (0.475 mM in DME). The low extinction coefficient values can be attributed to the limited stability of **5Na** in the DME solvent used for the measurement.

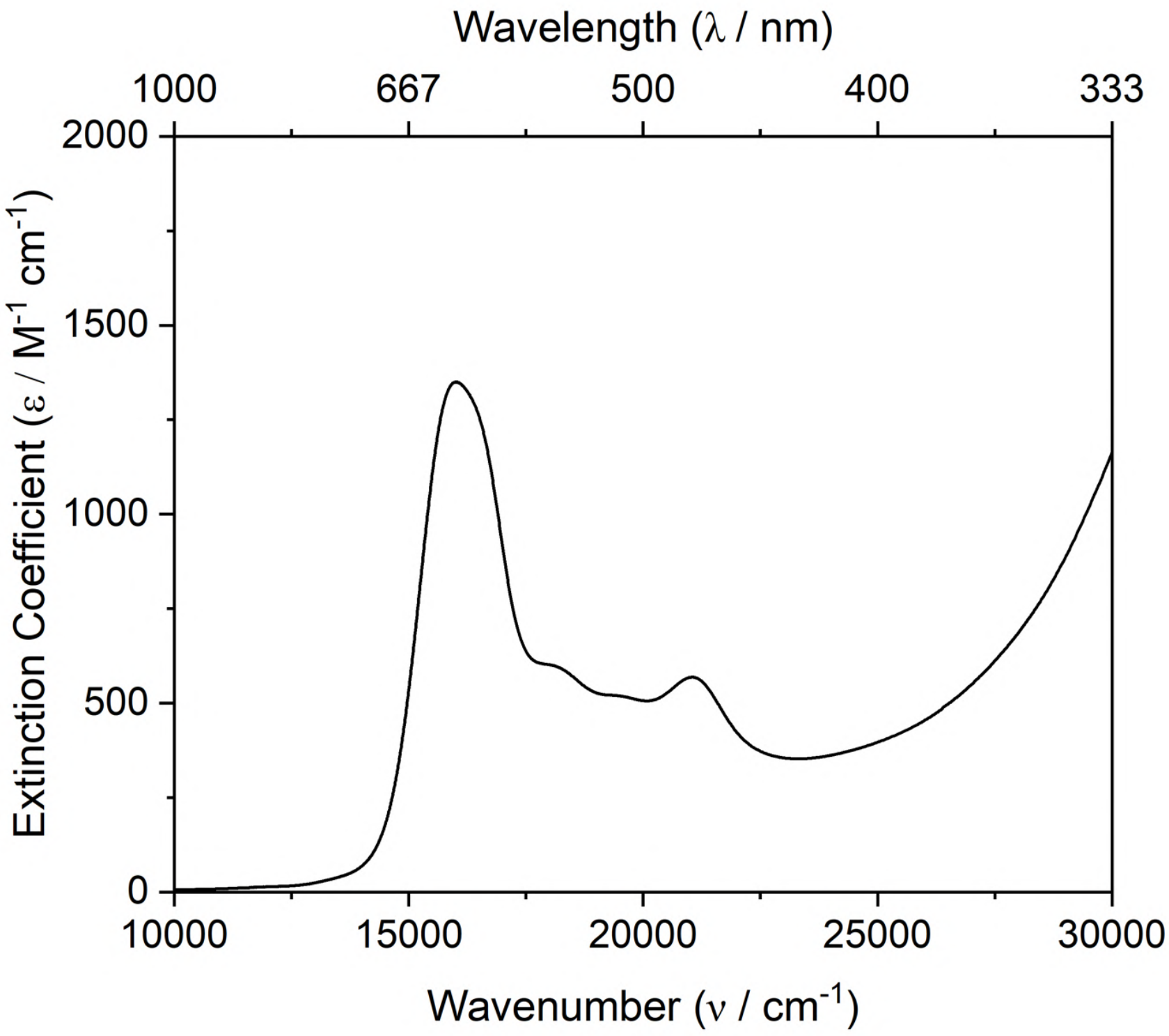

Supplementary Figure 109. UV/Vis/NIR spectrum of **5K** (0.121 mM in DME).

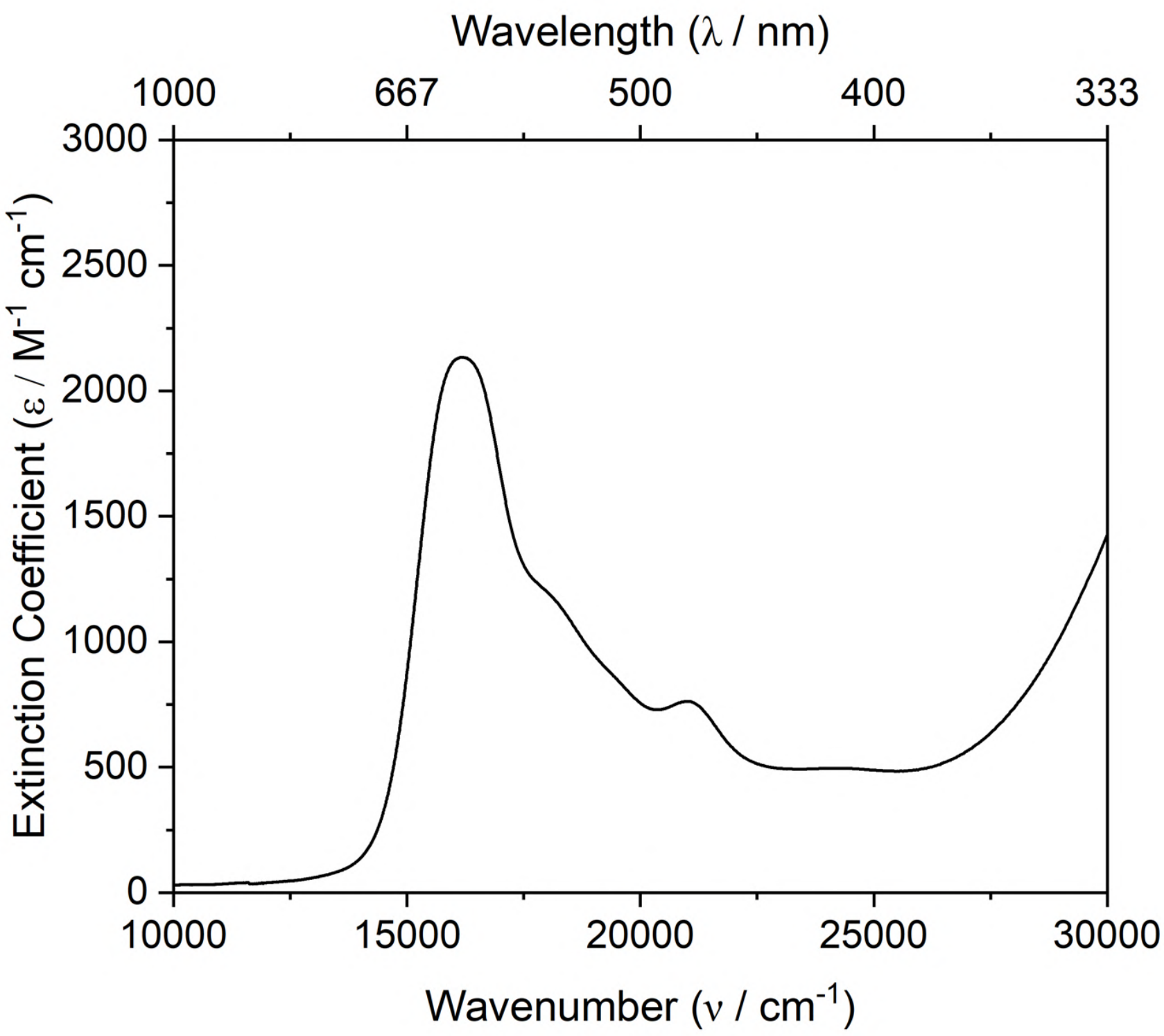

Supplementary Figure 110. UV/Vis/NIR spectrum of **5Rb** (0.294 mM in DME).

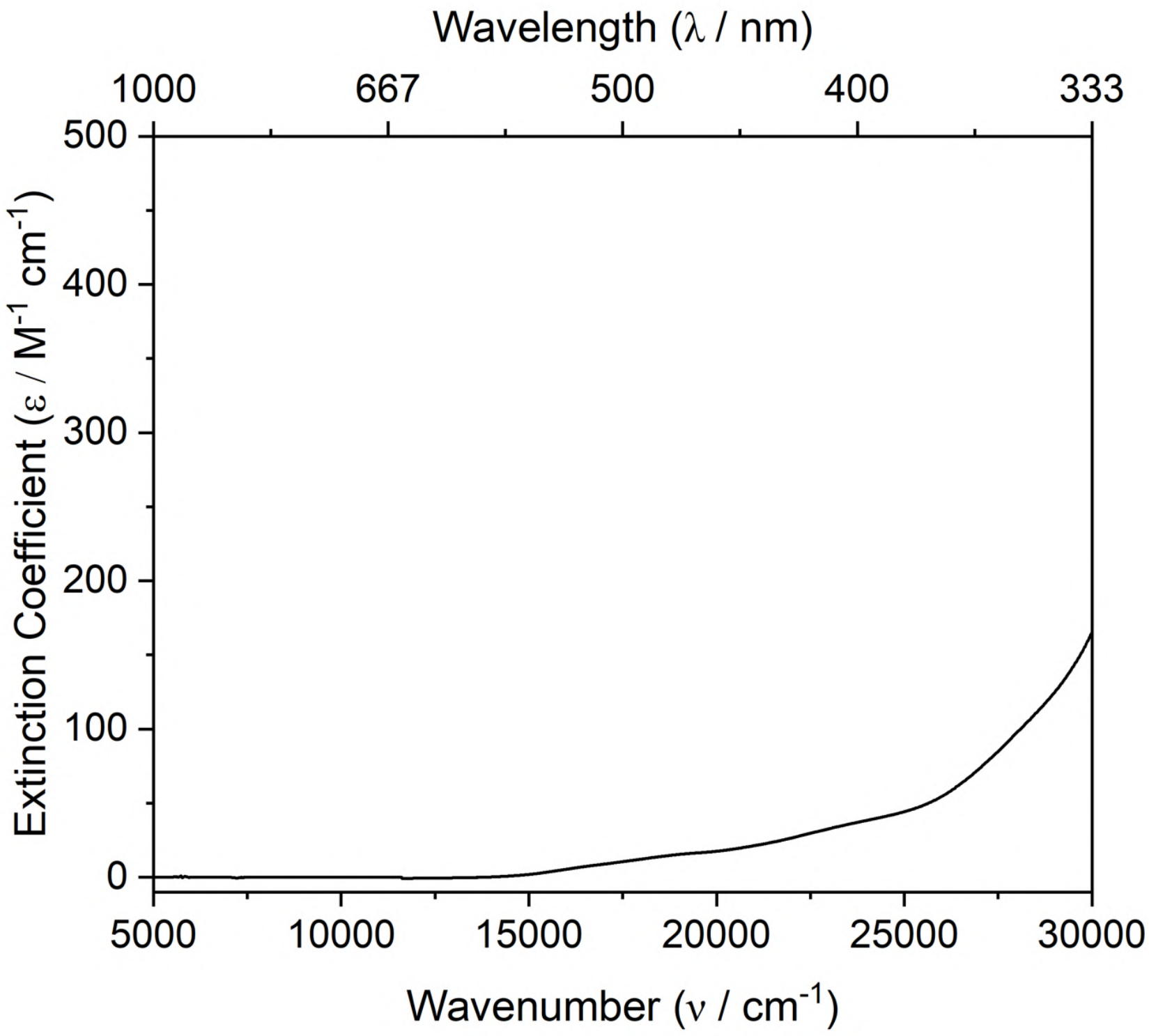

Supplementary Figure 111. UV/Vis/NIR spectrum of **6** (1.245 mM in DME).

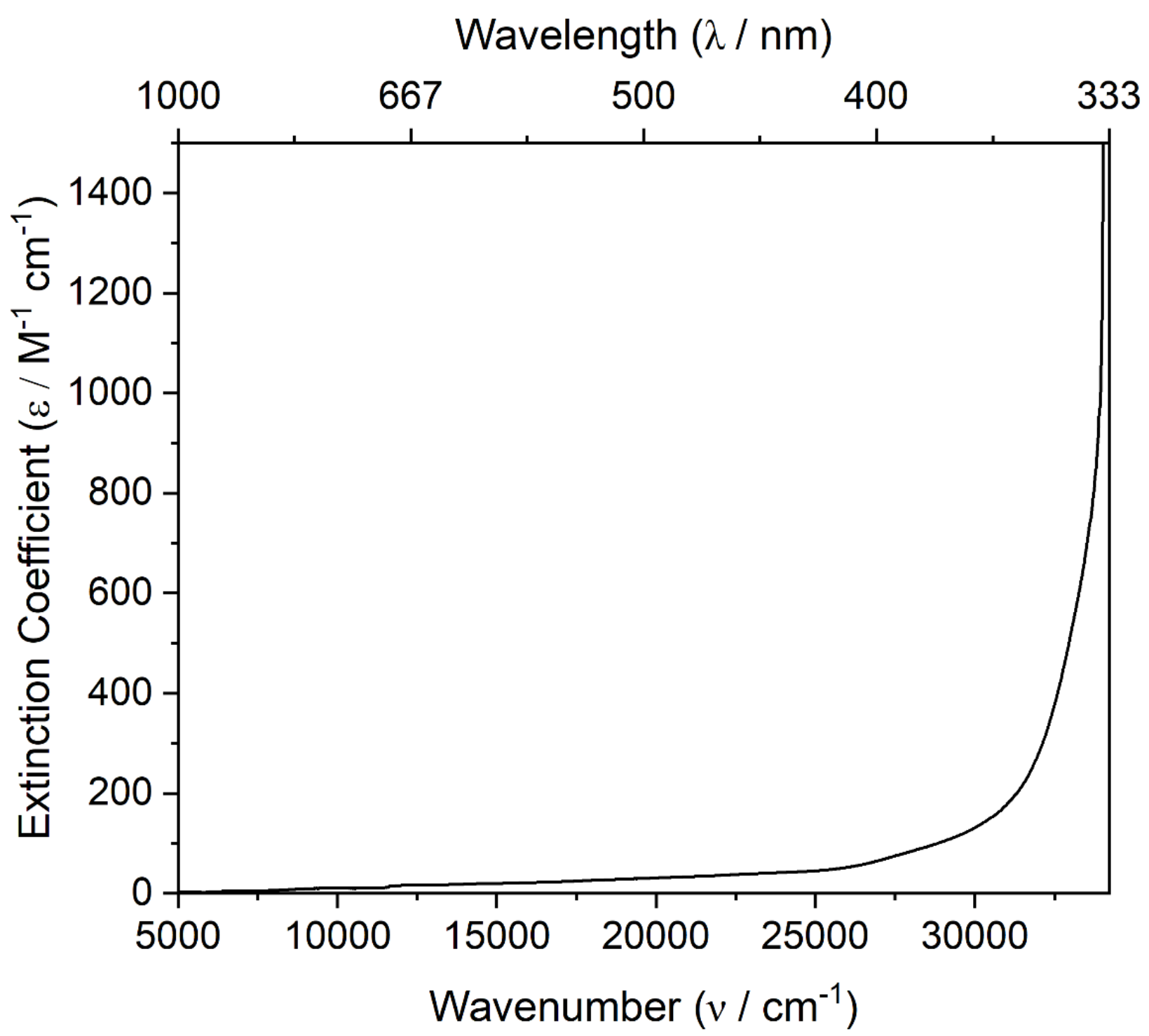

Supplementary Figure 112. UV/Vis/NIR spectrum of **7** (5.355 mM in DME).

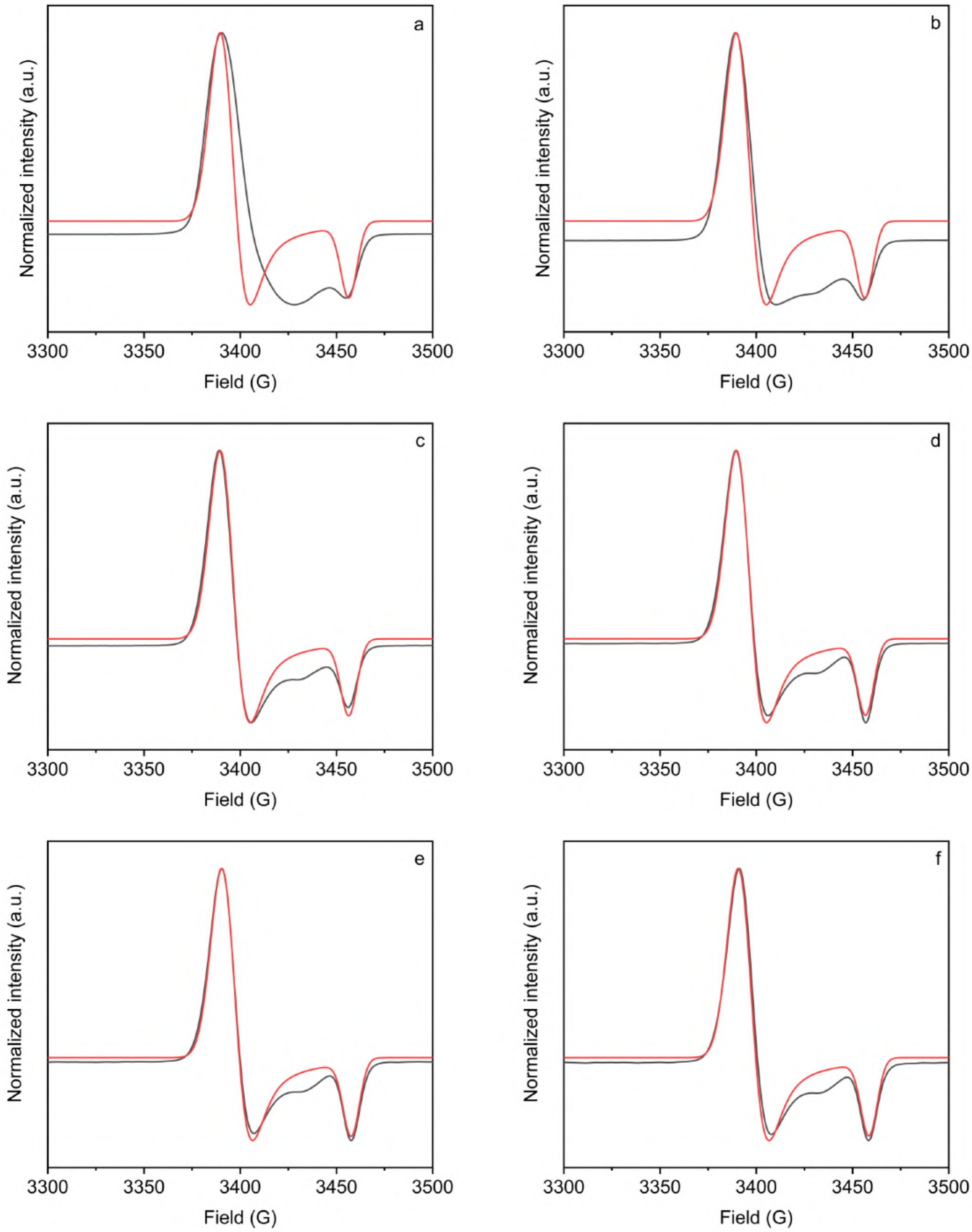

Supplementary Figure 113. X-band (9.4 GHz) EPR spectra of a frozen solution (DME:Toluene. 95:5, 3mM) of **4K** measured at 6 (a), 10 (b), 20 (c), 50 (d), 80 (e), and 100 (f) K (attenuation, 25 dB; modulation frequency, 100 kHz; modulation amplitude, 2 G).

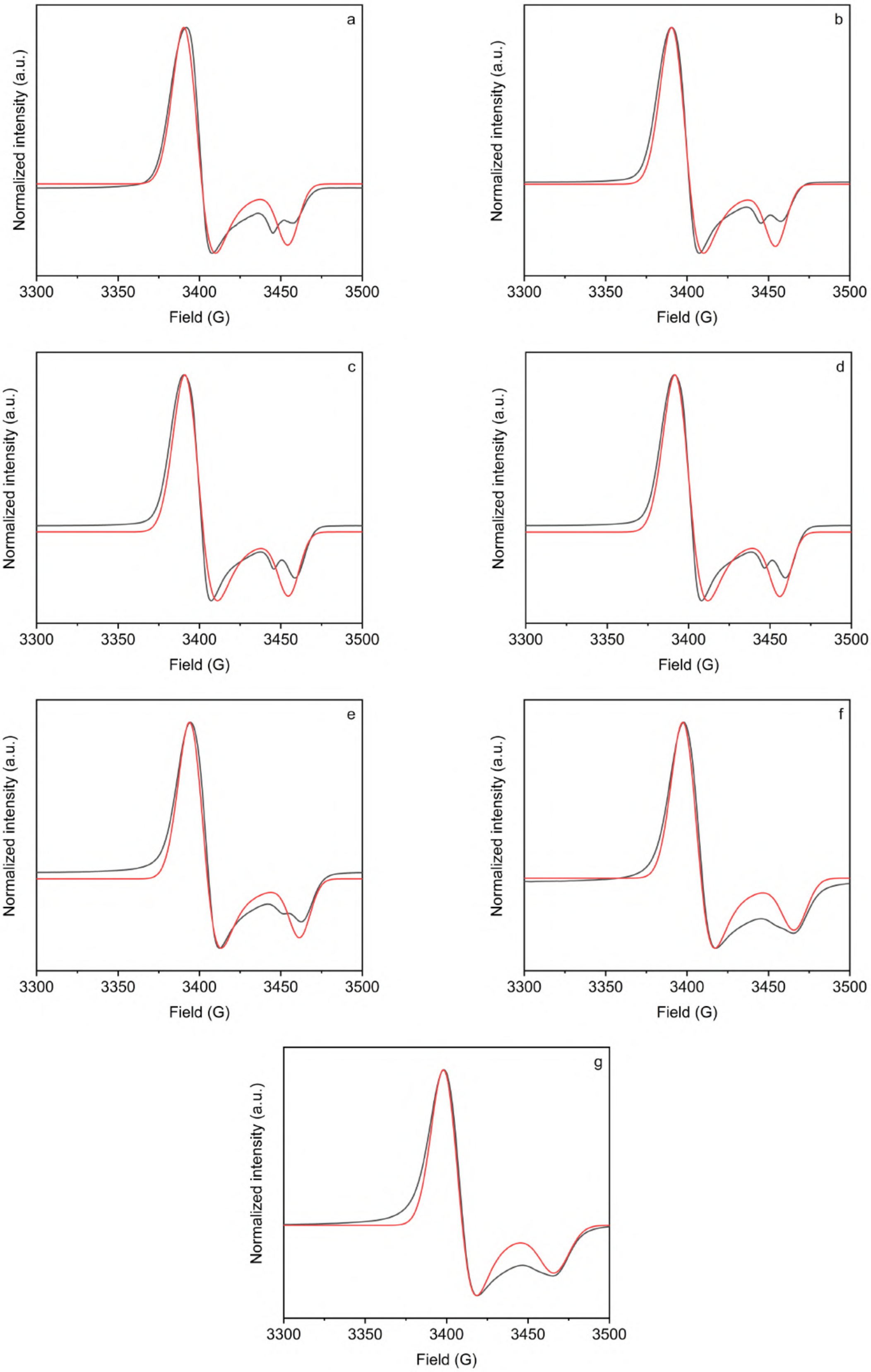

Supplementary Figure 114. X-band (9.4 GHz) EPR spectra of a powdered sample of **4K**, measured at 6 (a), 20 (b), 50 (c), 80 (d), 180 (e), 240 (f), and 270 (g) K, under modulation frequency of 100 kHz, modulation amplitude of 2 G, and microwave attenuation of 25 dB.

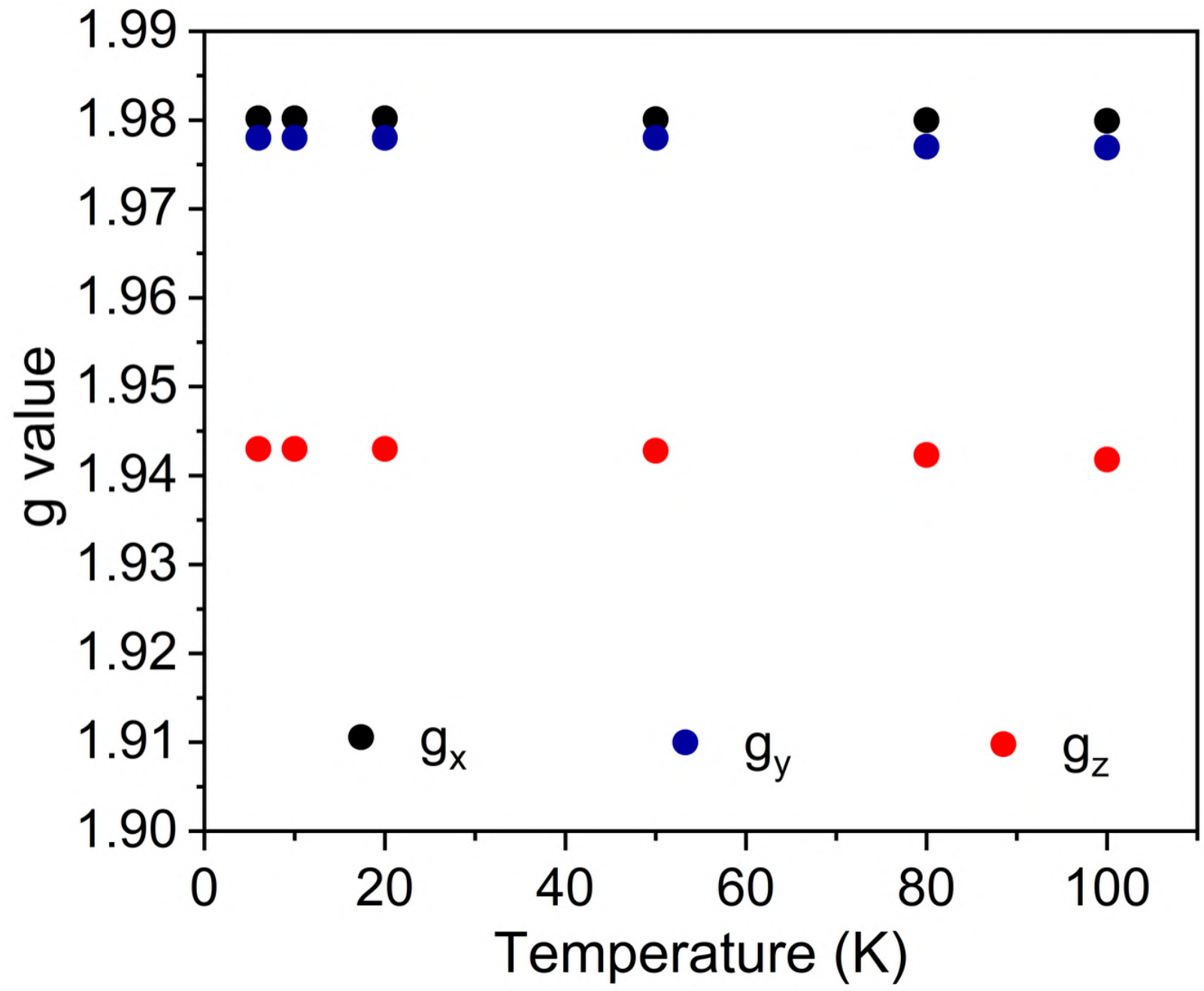

Supplementary Figure 115. Temperature dependence of the extracted *g*-values (g_x = black; g_y = blue; g_z = red) for a frozen solution (DME:Toluene. 95:5, 3mM) of **4K**.

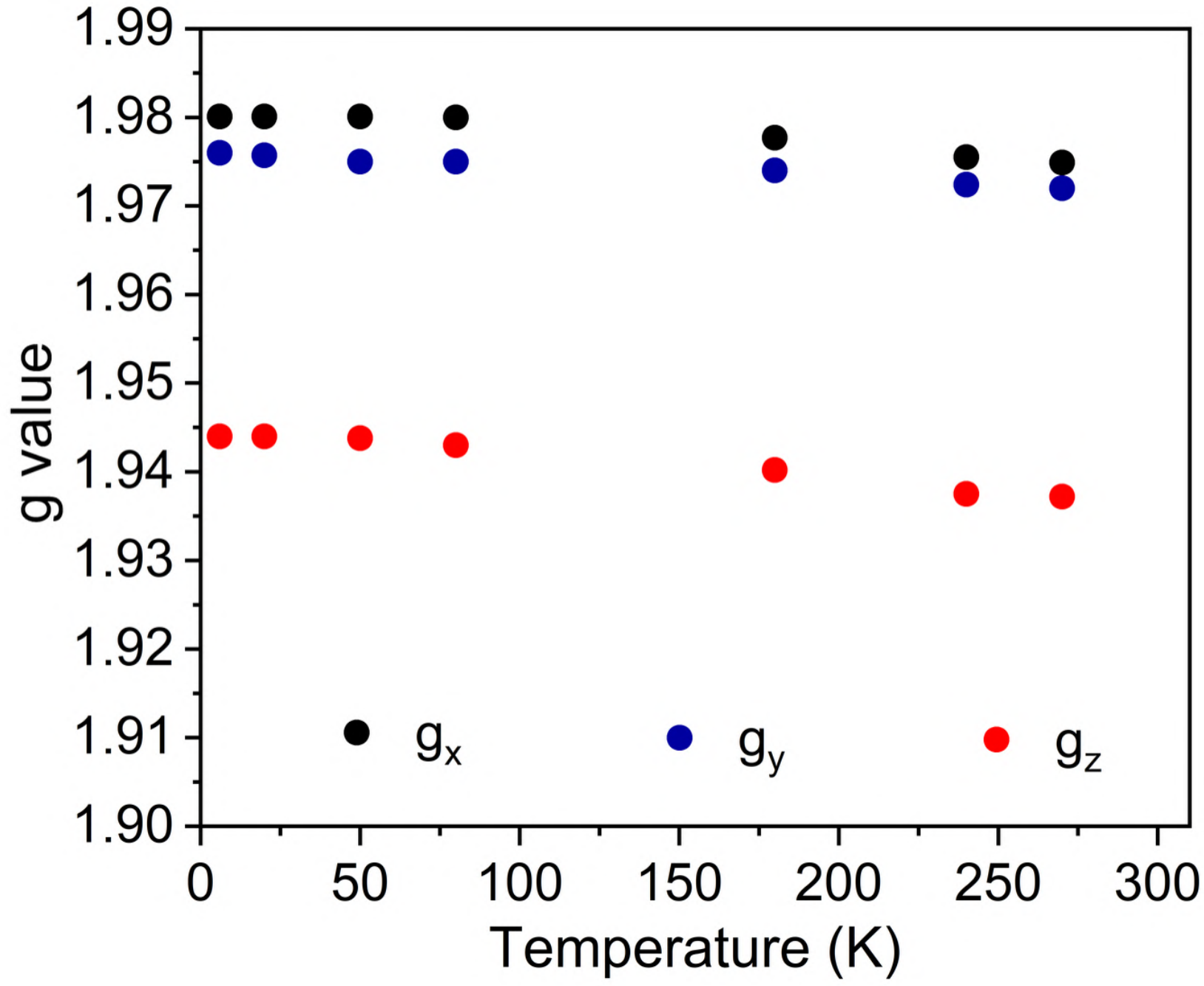

Supplementary Figure 116. Temperature dependence of the extracted *g*-values (g_x = black; g_y = blue; g_z = red) for a powdered sample of **4K**.

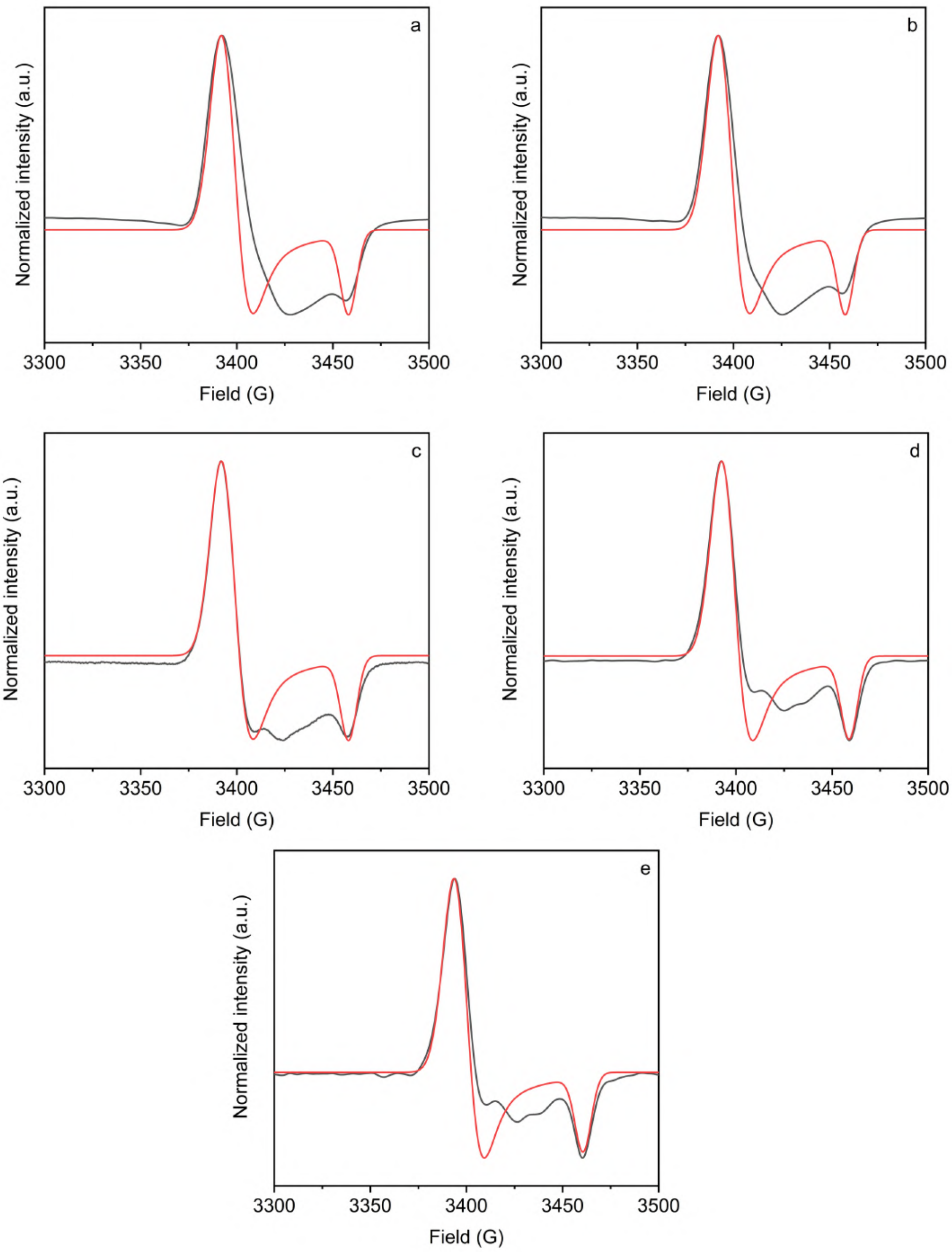

Supplementary Figure 117. X-band (9.4 GHz) EPR spectra of a frozen solution (DME:Toluene. 95:5, 3mM) of **4aRb** measured at 6 (a), 10 (b), 20 (c), 50 (d) and 100 (e) K (attenuation, 25 dB; modulation frequency, 100 kHz; modulation amplitude, 2 G).

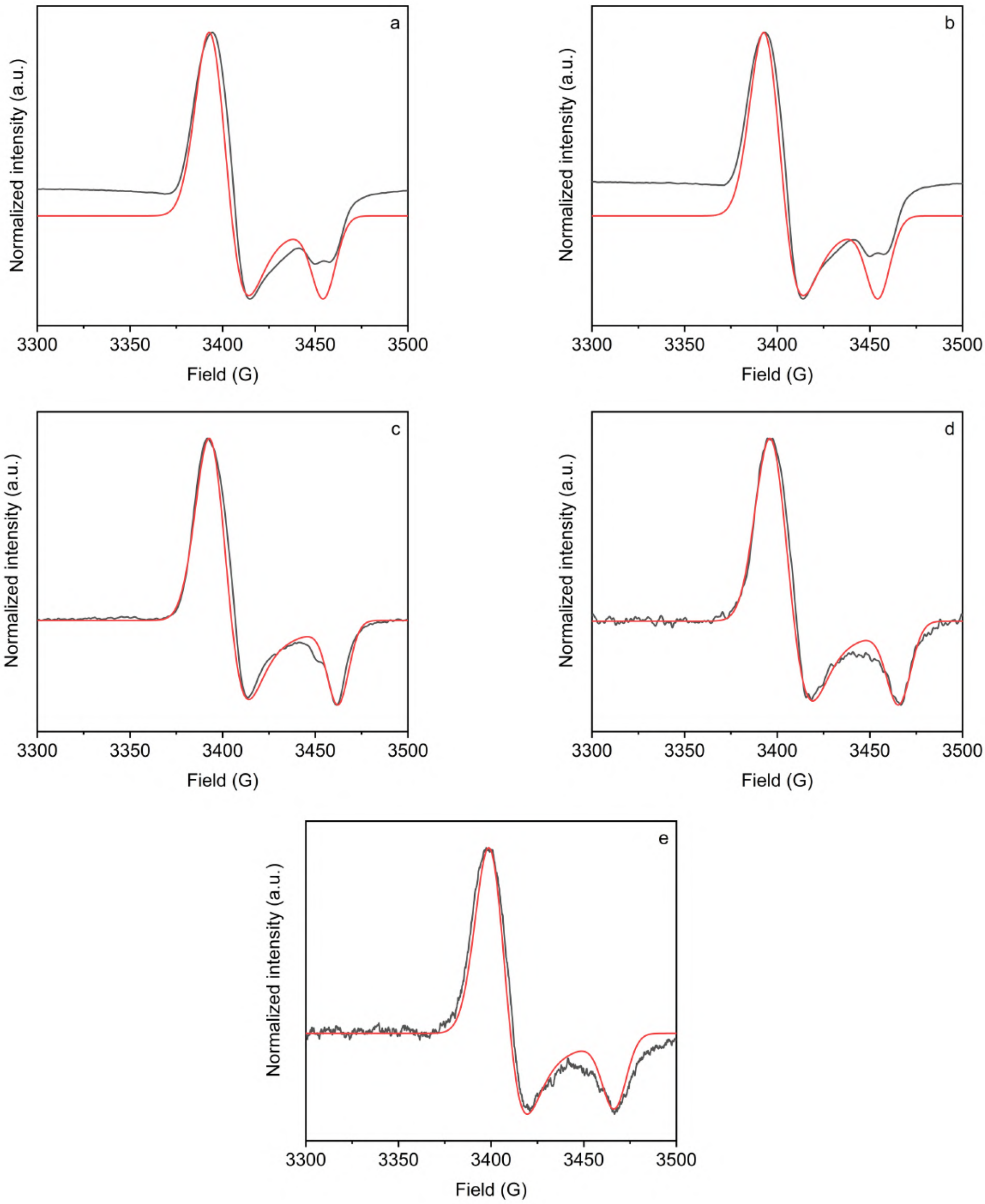

Supplementary Figure 118. X-band (9.4 GHz) EPR spectra of a powdered sample of **4aRb**, measured at 6 (a), 10 (b), 80 (c), 180 (d), and 240 (e) K, under modulation frequency of 100 kHz, modulation amplitude of 2 G, and microwave attenuation of 25 dB.

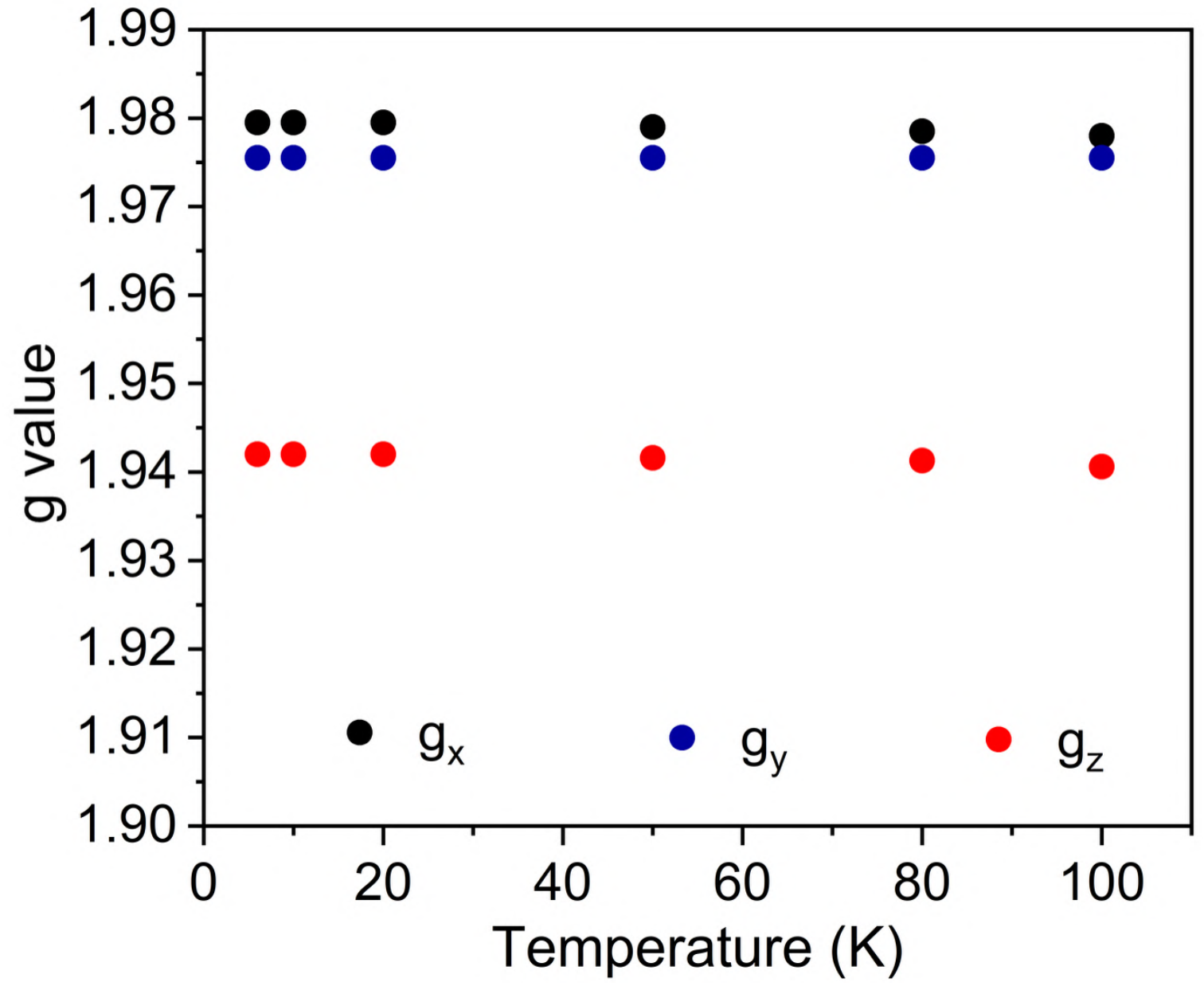

Supplementary Figure 119. Temperature dependence of the extracted *g*-values (g_x = black; g_y = blue; g_z = red) for a frozen solution (DME:Toluene. 95:5, 3mM) of **4aRb**.

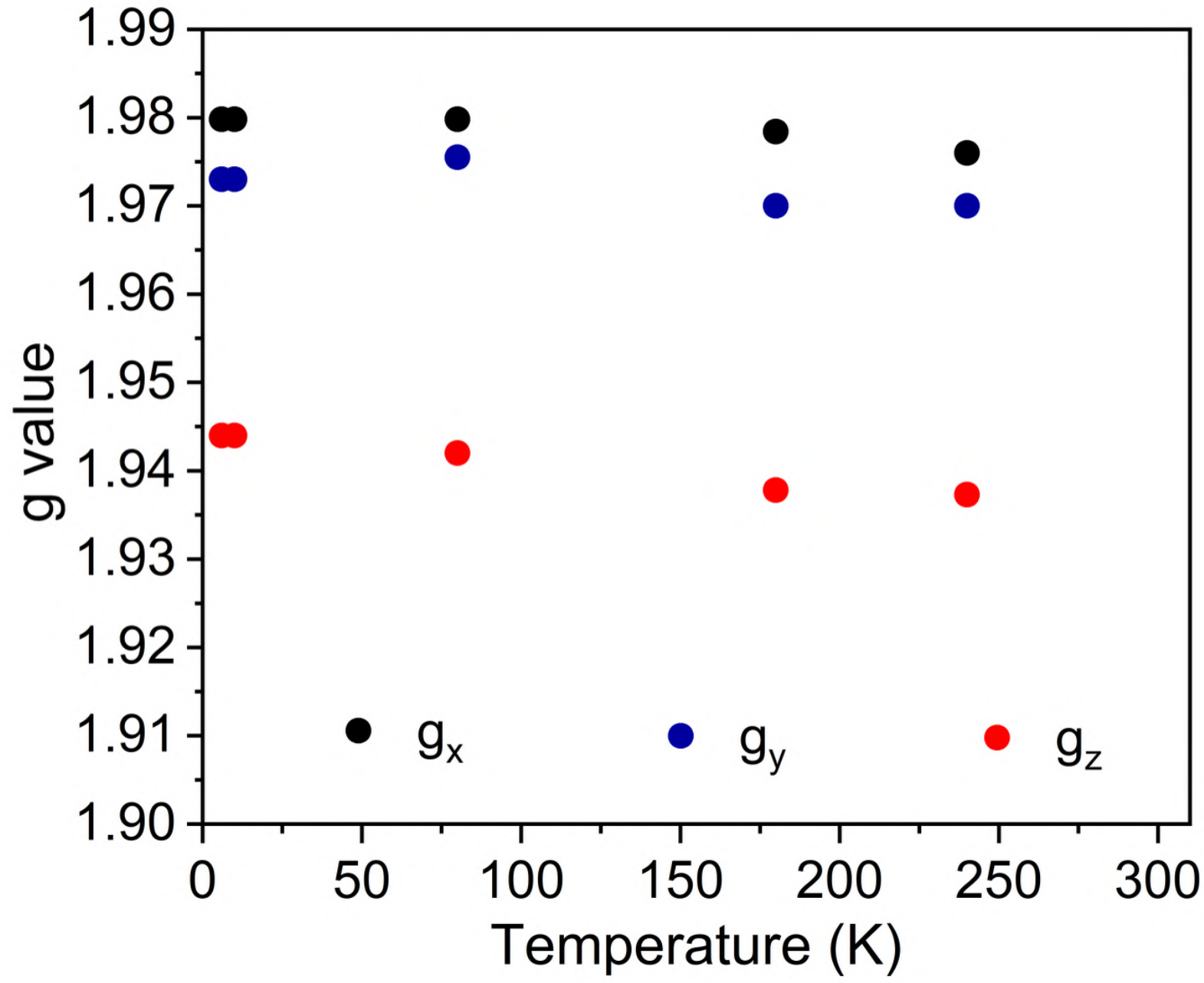

Supplementary Figure 120. Temperature dependence of the extracted *g*-values (g_x = black; g_y = blue; g_z = red) for a powdered sample of **4aRb**.

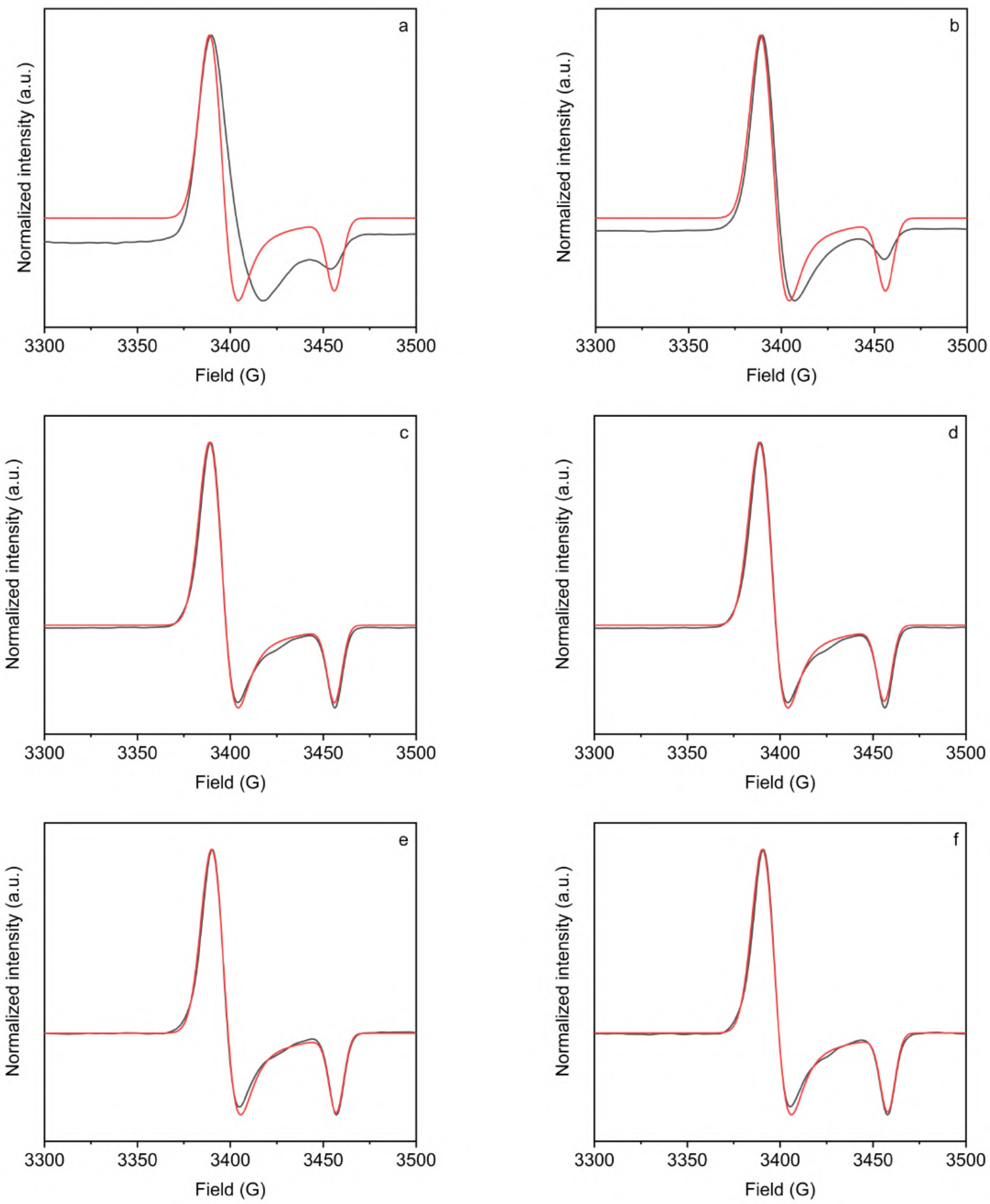

Supplementary Figure 121. X-band (9.4 GHz) EPR spectra of a frozen solution (DME:Toluene. 95:5, 3mM) of **4Cs** measured at 6 (a), 10 (b), 20 (c), 50 (d), 80 (e), and 100 (f) K (attenuation, 25 dB; modulation frequency, 100 kHz; modulation amplitude, 2 G).

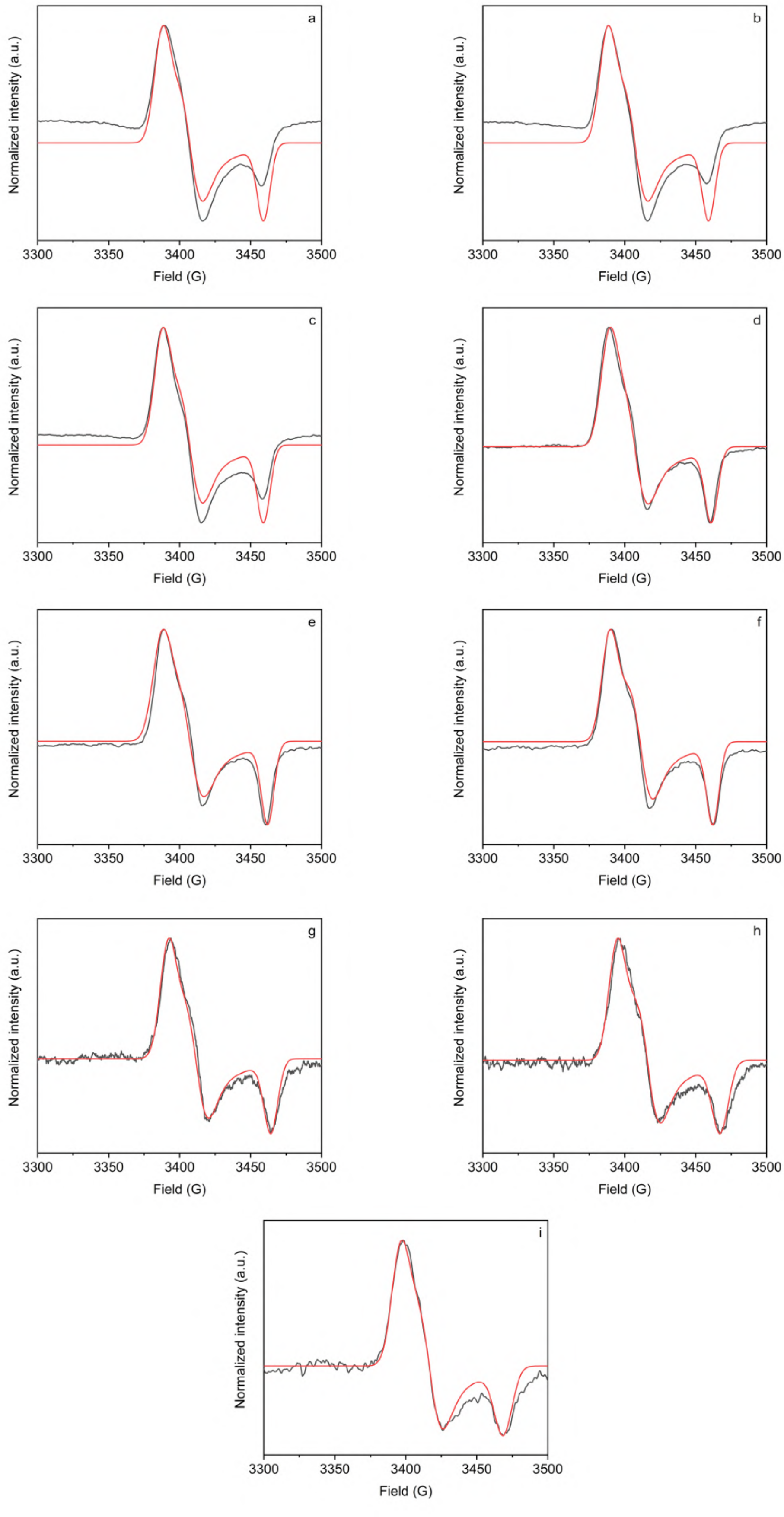

Supplementary Figure 122. X-band (9.4 GHz) EPR spectra of a powdered sample of **4Cs**, measured at 7 (a), 10 (b), 20 (c), 50 (d), 80 (e), 120 (f), 180 (g), 240 (h), and 270 (i) K, under modulation frequency of 100 kHz, modulation amplitude of 2 G, and microwave attenuation of 25 dB.

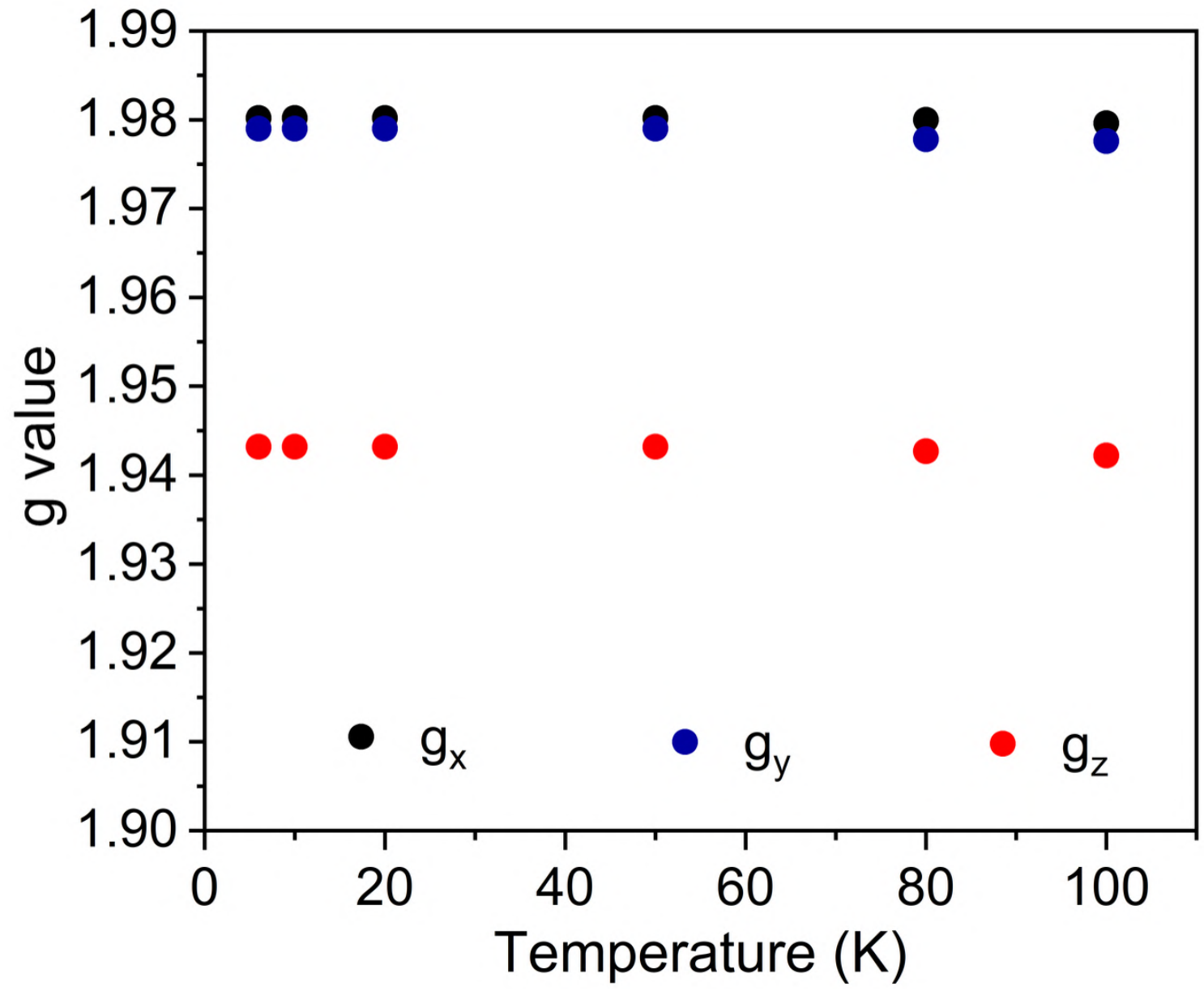

Supplementary Figure 123. Temperature dependence of the extracted *g*-values (g_x = black; g_y = blue; g_z = red) for a frozen solution (DME:Toluene. 95:5, 3mM) of **4Cs**.

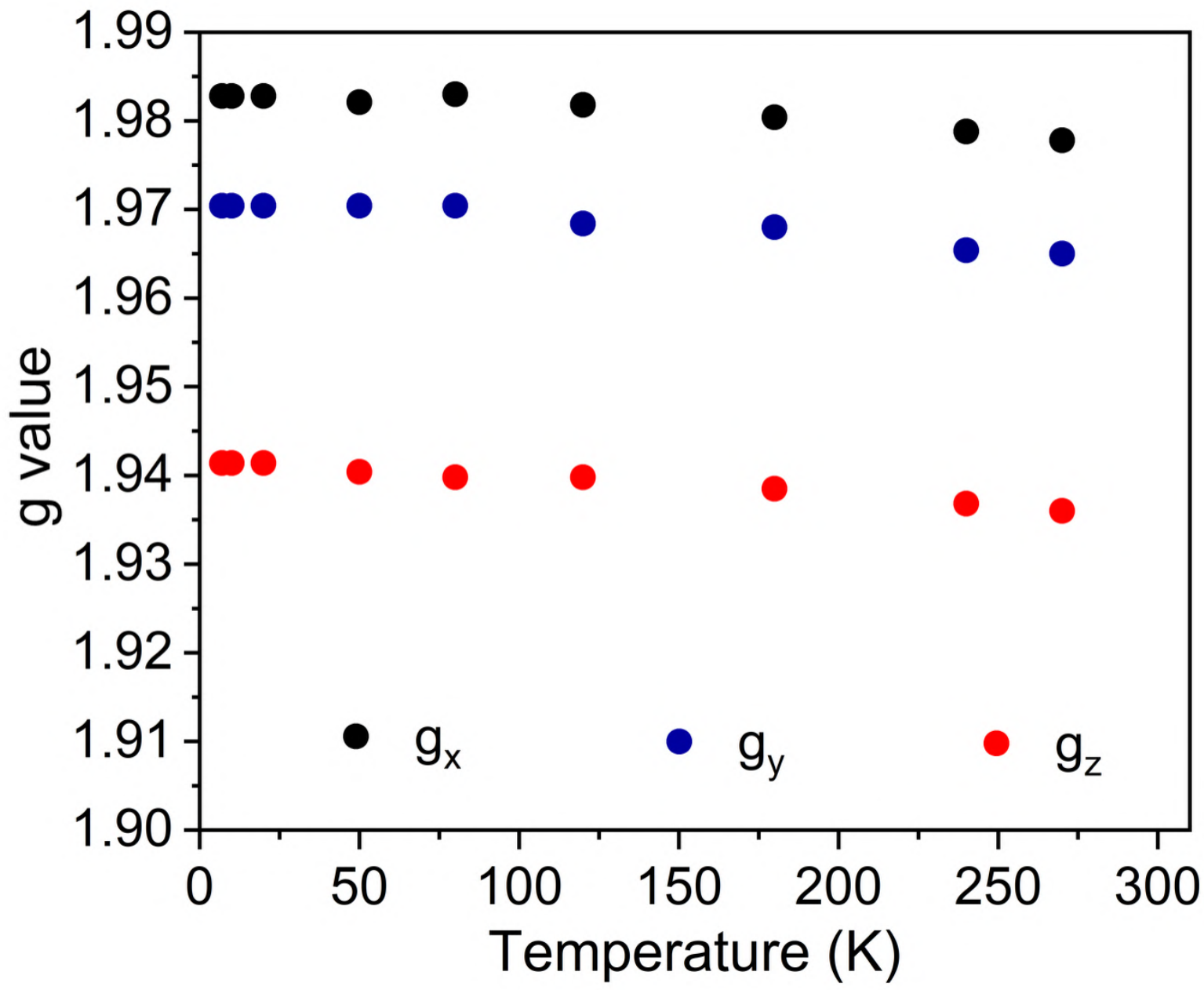

Supplementary Figure 124. Temperature dependence of the extracted *g*-values (g_x = black; g_y = blue; g_z = red) for a powdered sample of **4Cs**.

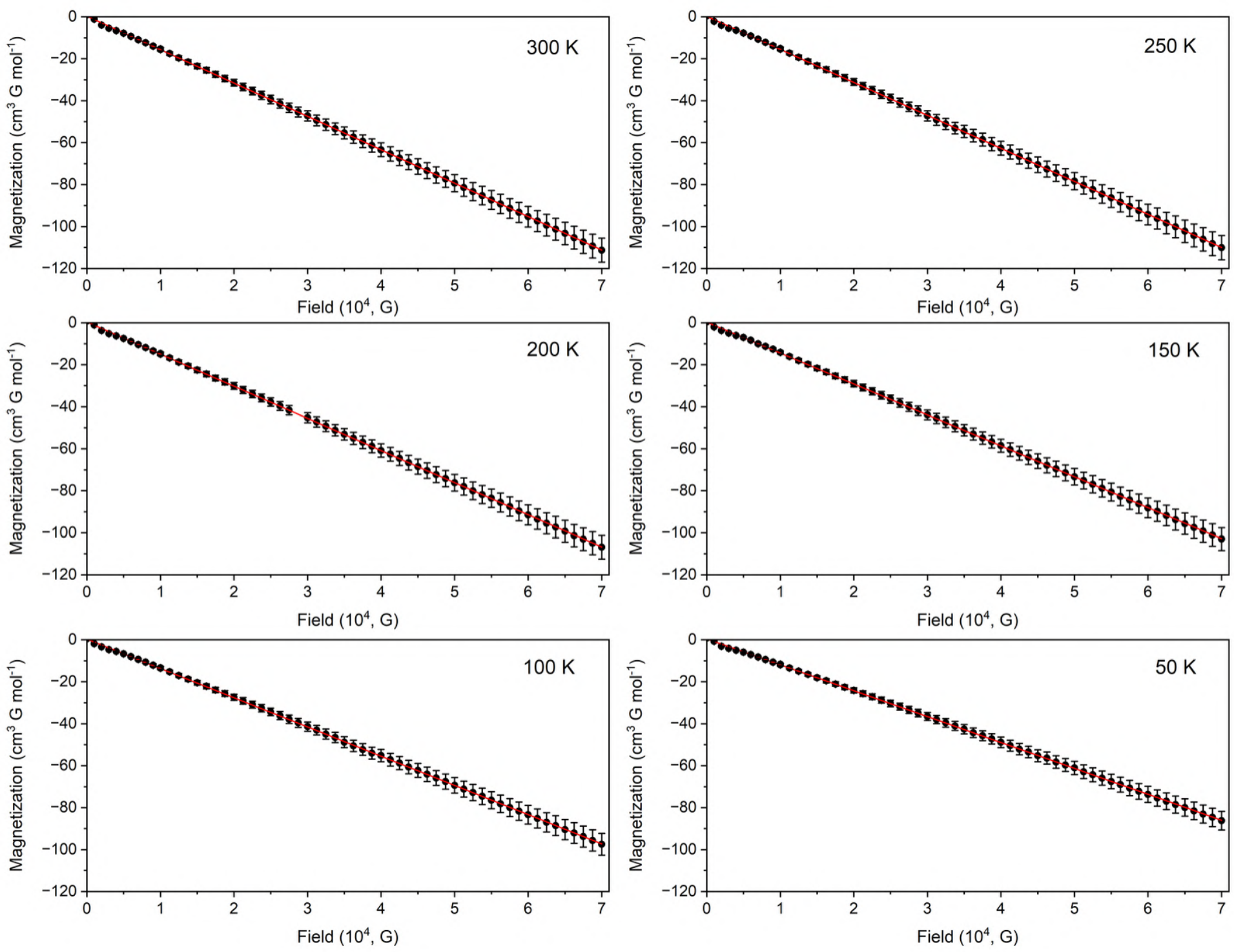

Supplementary Figure 125. Magnetization vs Field data for **4K** at 300 (top left: R^2 = 0.9999; average error = 5.16%), 250 (top right: R^2 = 0.9999; average error = 5.23%), 200 (middle left: R^2 = 0.9999; average error = 5.23%), 150 (middle right: R^2 = 0.9999; average error = 5.30%), 100 (bottom left: R^2 = 0.9999; average error = 5.32%) and 50 (bottom right: R^2 = 0.9999; average error = 5.16%) K from 0-7 Tesla. The straight lines are linear regressions of the data points. Missing field measurements are due to measurement error. Errors derive from the standard deviation of the sampling distribution of the points used (sample population divided by the square root of the number of samples) to compute the reported moment represented as a percentage.

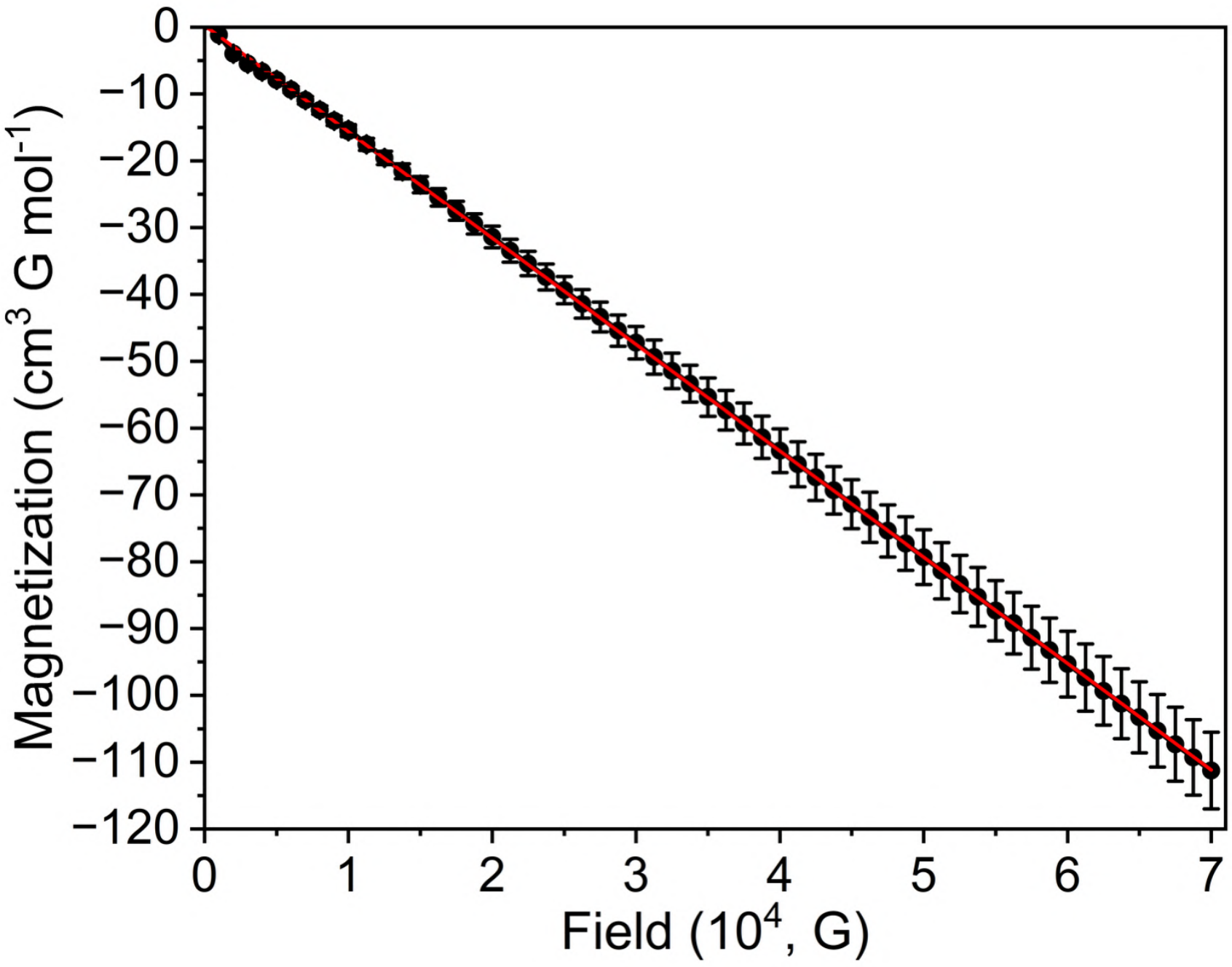

Supplementary Figure 126. Magnetization vs Field data for **4K** (black: average error = 5.16%) at 300 K from 0-7 Tesla. The straight lines are linear regressions of the data points. On a per molecule basis, $\chi_D = -1591.75 \times 10^{-6}$ cm^3 mol^{-1} (R^2 = 0.9999) for **4K**. Errors derive from the standard deviation of the sampling distribution of the points used (sample population divided by the square root of the number of samples) to compute the reported moment represented as a percentage.

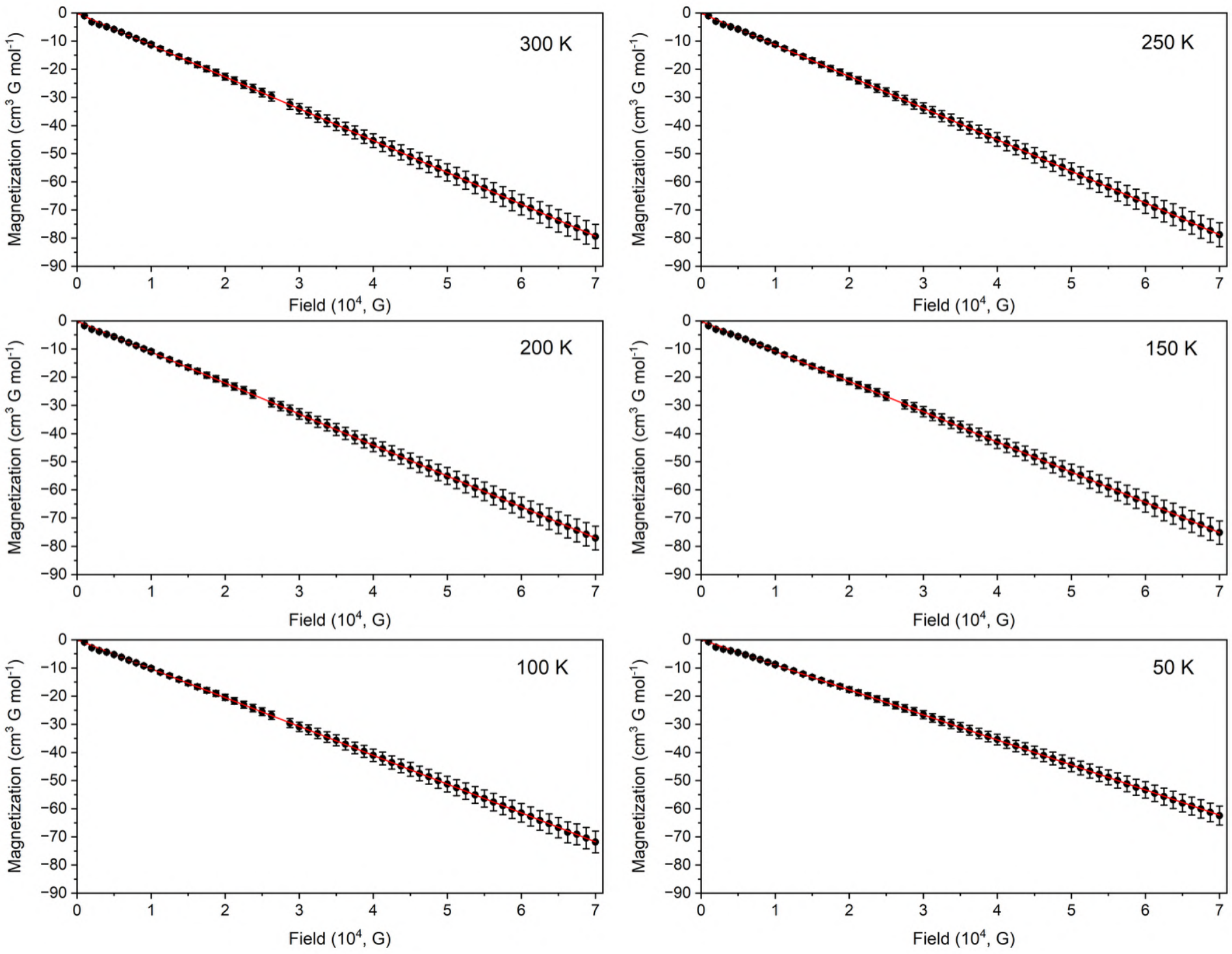

Supplementary Figure 127. Magnetization vs Field data for **4aRb** at 300 (top left: R^2 = 0.9999; average error = 5.39%), 250 (top right: R^2 = 0.9999; average error = 5.38%), 200 (middle left: R^2 = 0.9999; average error = 5.44%), 150 (middle right: R^2 = 0.9999; average error = 5.44%), 100 (bottom left: R^2 = 0.9999; average error = 5.40%) and 50 (bottom right: R^2 = 0.9999; average error = 5.40%) K from 0-7 Tesla. The straight lines are linear regressions of the data points. Missing field measurements are due to measurement error. Errors derive from the standard deviation of the sampling distribution of the points used (sample population divided by the square root of the number of samples) to compute the reported moment represented as a percentage.

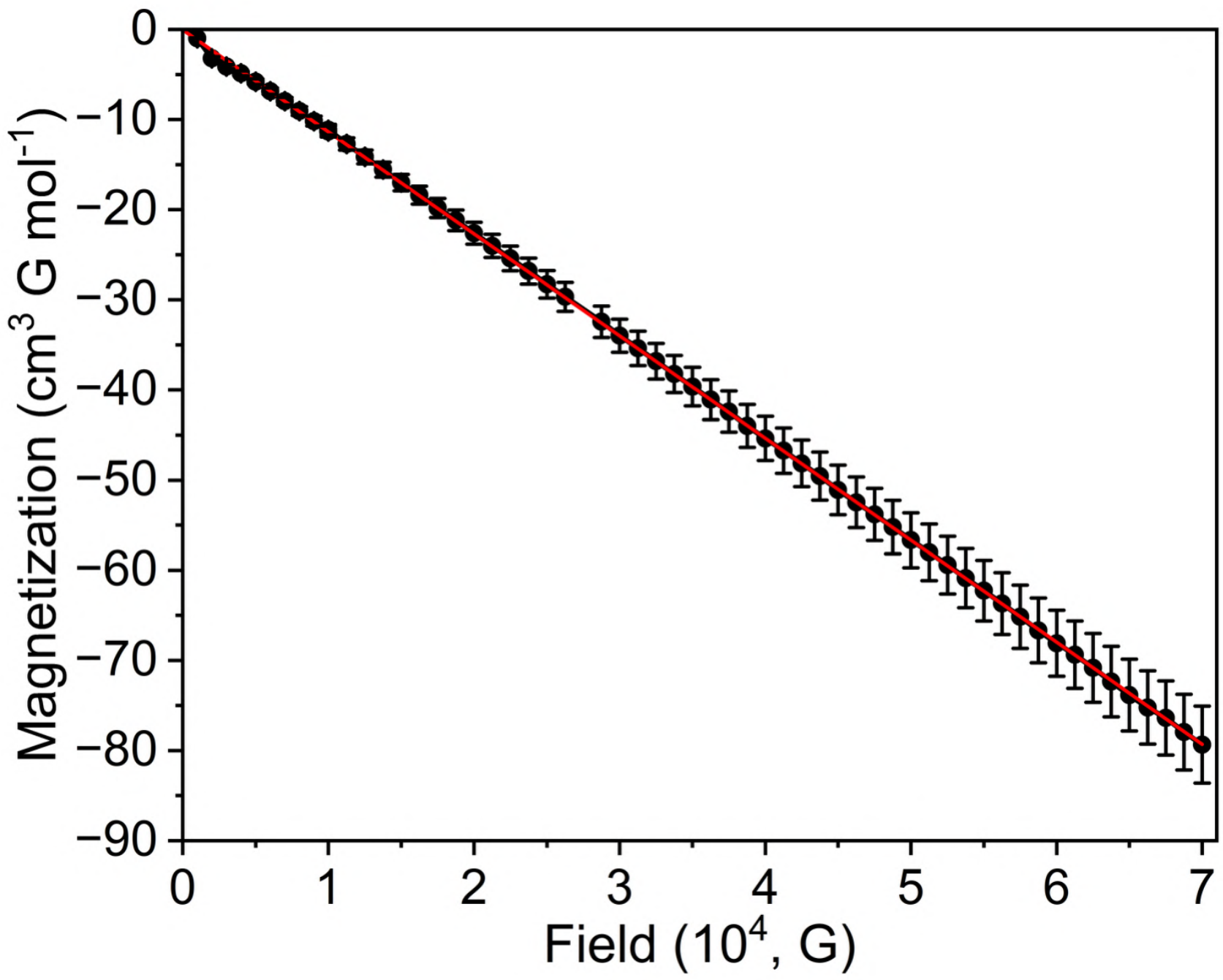

Supplementary Figure 128. Magnetization vs Field data for **4aRb** (black: average error = 5.39%) at 300 K from 0-7 Tesla. The straight lines are linear regressions of the data points. On a per molecule basis, $\chi_D = -1132.43 \times 10^{-6}$ cm^3 mol^{-1} (R^2 = 0.9999) for **4aRb**. Errors derive from the standard deviation of the sampling distribution of the points used (sample population divided by the square root of the number of samples) to compute the reported moment represented as a percentage.

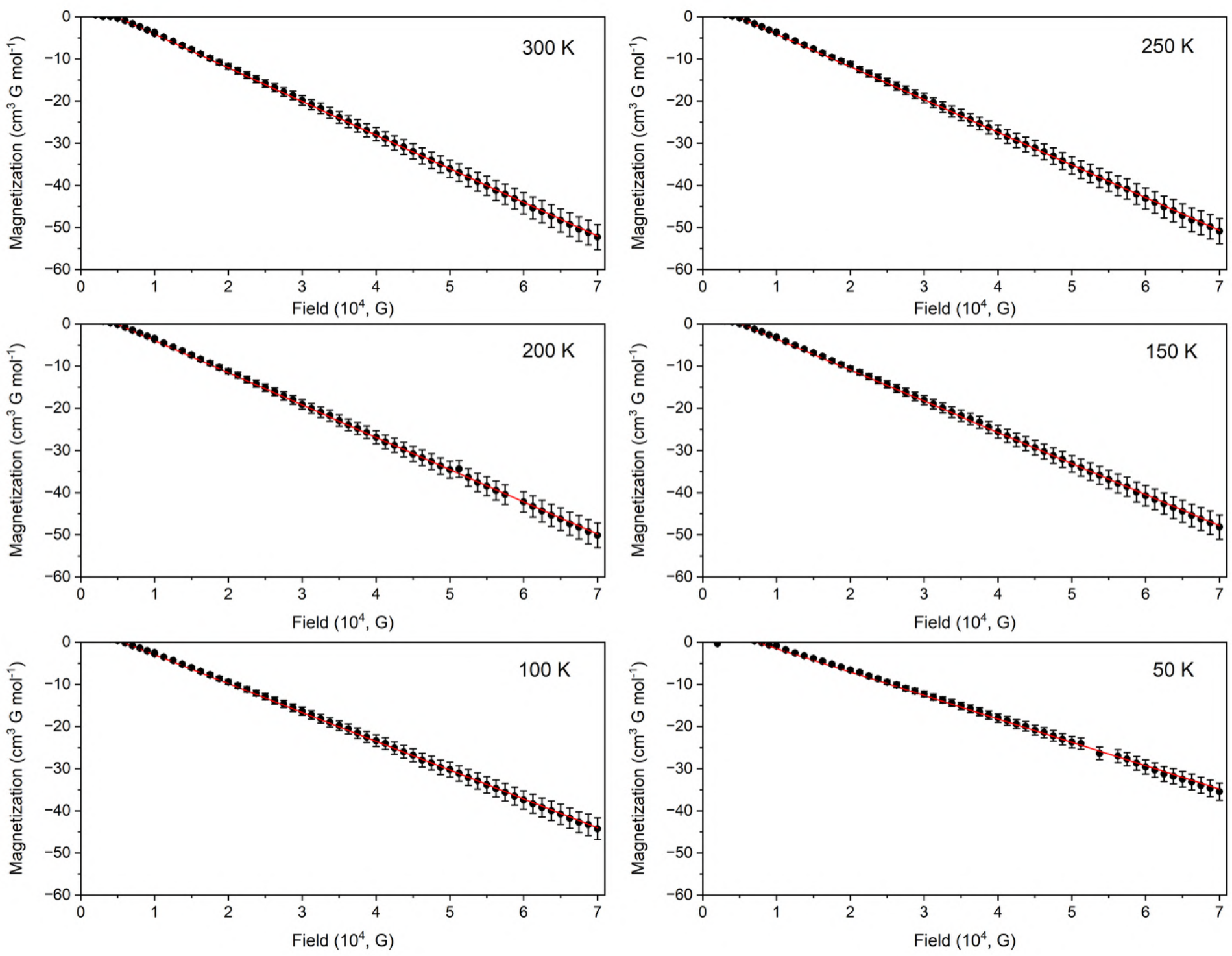

Supplementary Figure 129. Magnetization vs Field data for **4Cs** at 300 (top left: R^2 = 0.9993; average error = 5.73%), 250 (top right: R^2 = 0.9992; average error = 5.83%), 200 (middle left: R^2 = 0.9990; average error = 5.79%), 150 (middle right: R^2 = 0.9989; average error = 5.92%), 100 (bottom left: R^2 = 0.9988; average error = 5.84%) and 50 (bottom right: R^2 = 0.9967; average error = 5.68%) K from 0-7 Tesla. The straight lines are linear regressions of the data points. Missing field measurements are due to measurement error. Errors derive from the standard deviation of the sampling distribution of the points used (sample population divided by the square root of the number of samples) to compute the reported moment represented as a percentage.

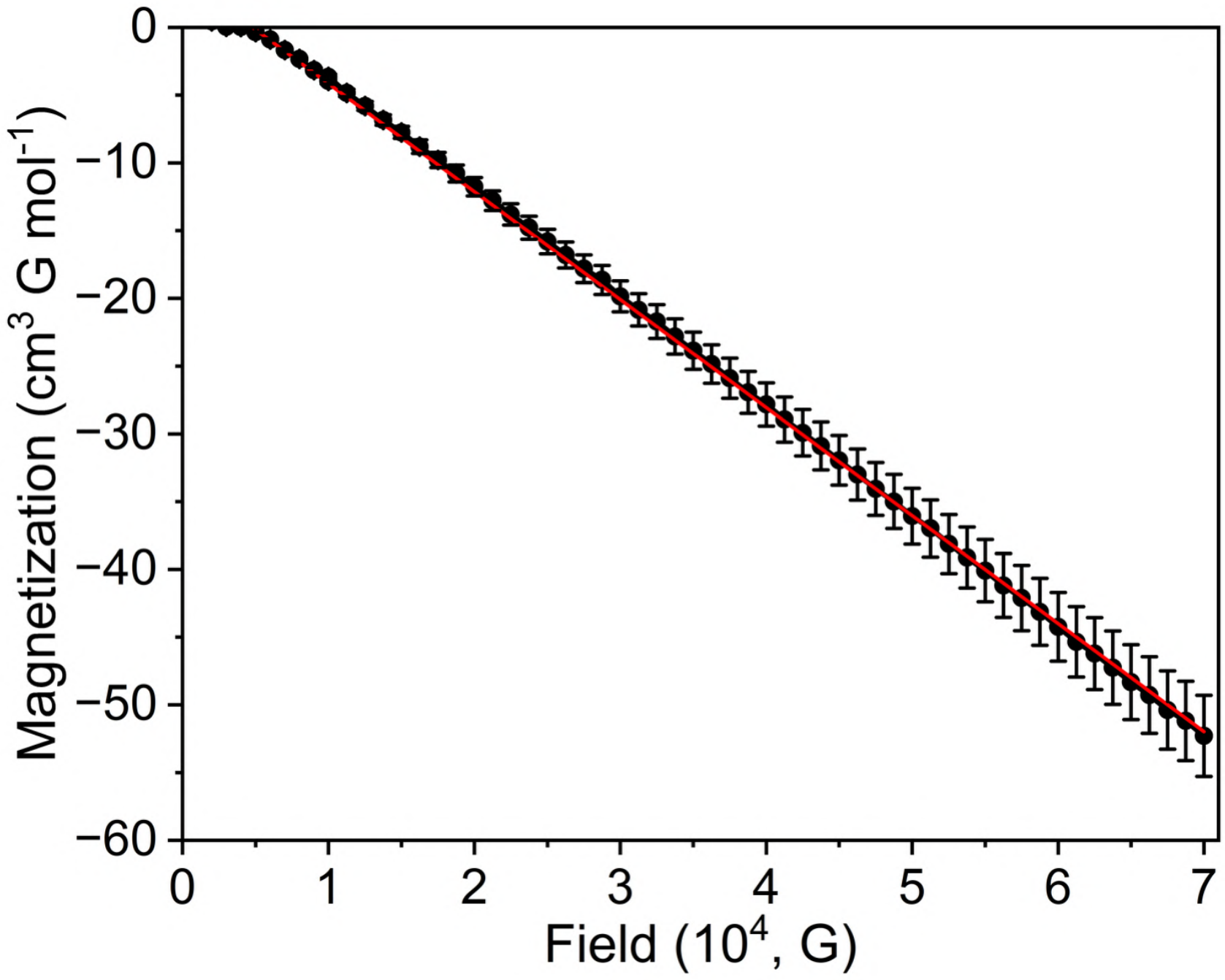

Supplementary Figure 130. Magnetization vs Field data for **4Cs** (black: average error = 5.73%) at 300 K from 0-7 Tesla. The straight lines are linear regressions of the data points. On a per molecule basis, $\chi_D = -797.22 \times 10^{-6}$ cm^3 mol^{-1} (R^2 = 0.9993) for **4Cs**. Errors derive from the standard deviation of the sampling distribution of the points used (sample population divided by the square root of the number of samples) to compute the reported moment represented as a percentage.

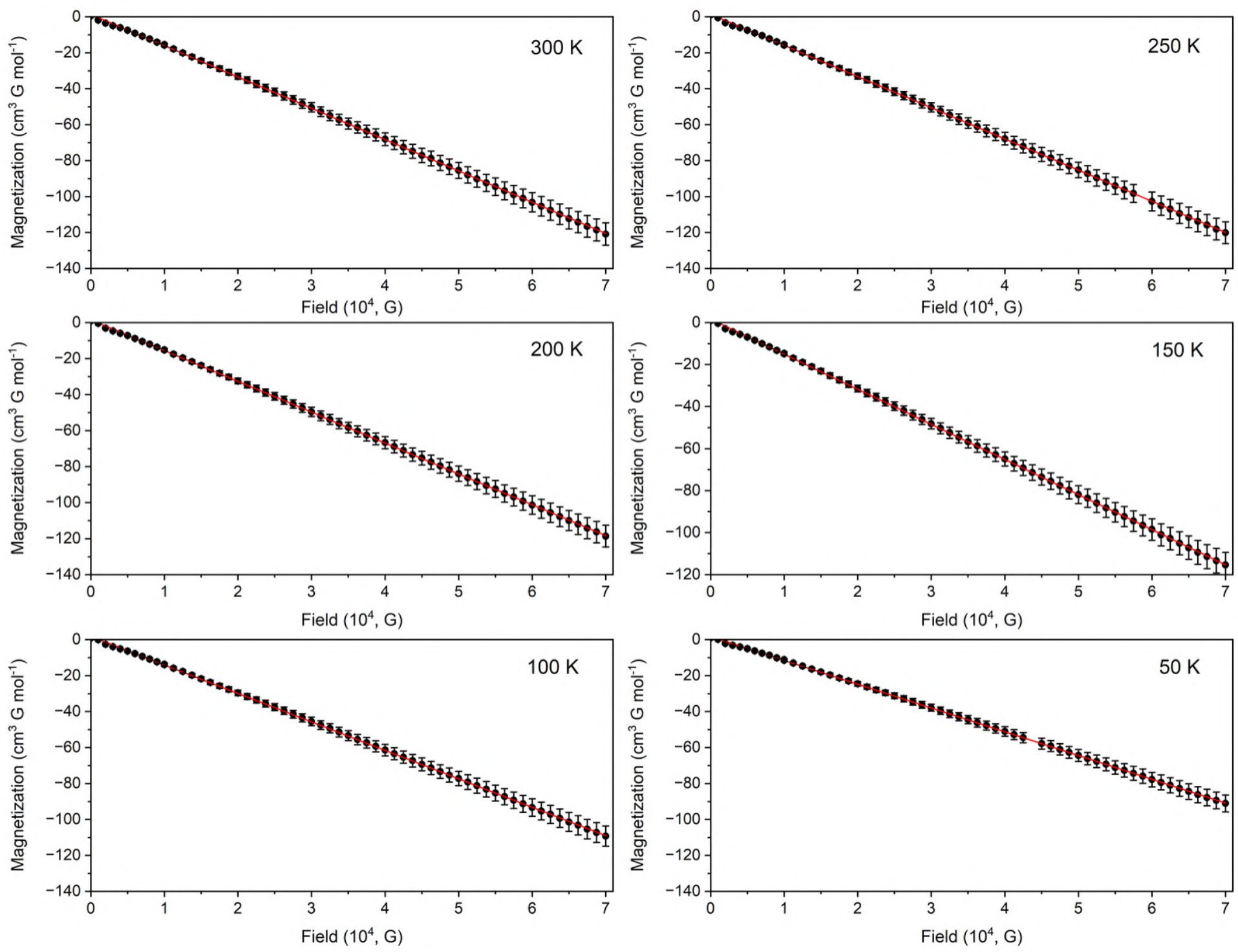

Supplementary Figure 131. Magnetization vs Field data for **5Na** at 300 (top left: R^2 = 0.9999; average error = 5.17%), 250 (top right: R^2 = 0.9999; average error = 5.08%), 200 (middle left: R^2 = 0.9999; average error = 5.07%), 150 (middle right: R^2 = 0.9999; average error = 5.17%), 100 (bottom left: R^2 = 0.9999; average error = 5.17%) and 50 (bottom right: R^2 = 0.9999; average error = 5.19%) K from 0-7 Tesla. The straight lines are linear regressions of the data points. Missing field measurements are due to measurement error. Errors derive from the standard deviation of the sampling distribution of the points used (sample population divided by the square root of the number of samples) to compute the reported moment represented as a percentage.

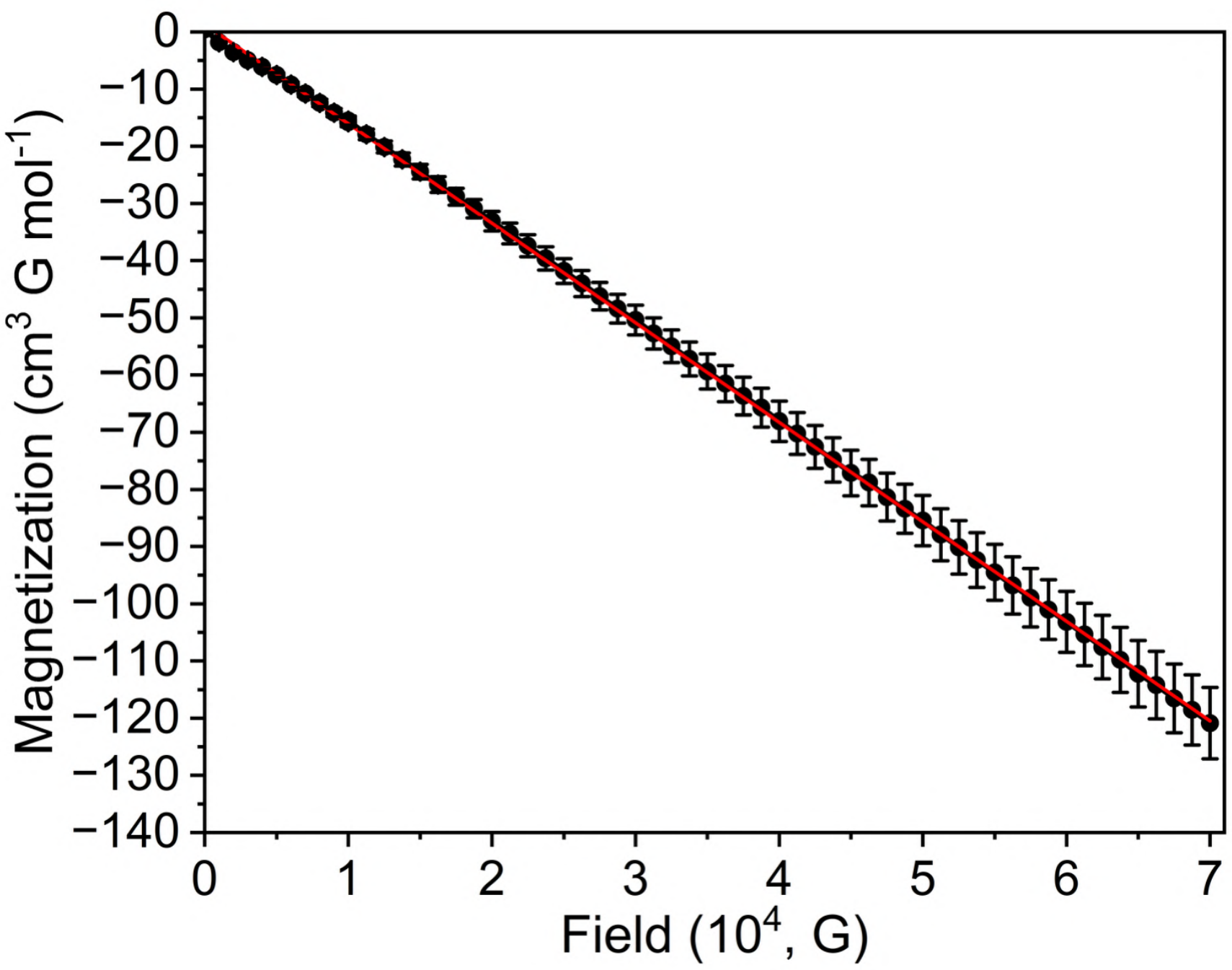

Supplementary Figure 132. Magnetization vs Field data for **5Na** (black: average error = 5.17%) at 300 K from 0-7 Tesla. The straight lines are linear regressions of the data points. On a per molecule basis, $\chi_D = -1739.88 \times 10^{-6}$ cm^3 mol^{-1} (R^2 = 0.9999) for **5Na**. Errors derive from the standard deviation of the sampling distribution of the points used (sample population divided by the square root of the number of samples) to compute the reported moment represented as a percentage.

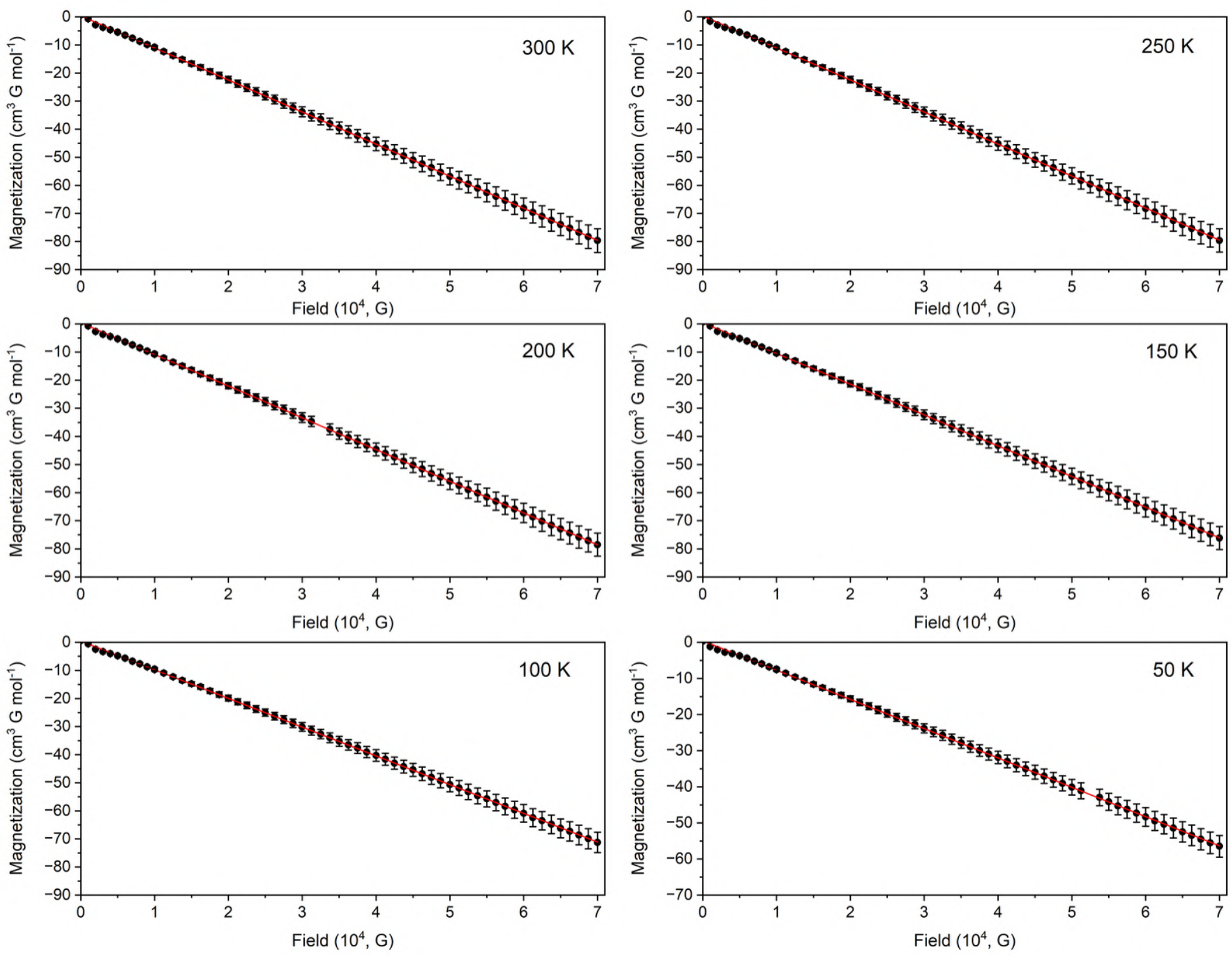

Supplementary Figure 133. Magnetization vs Field data for **5K** at 300 (top left: R^2 = 0.9999; average error = 5.32%), 250 (top right: R^2 = 0.9999; average error = 5.19%), 200 (middle left: R^2 = 0.9999; average error = 5.14%), 150 (middle right: R^2 = 0.9999; average error = 5.13%), 100 (bottom left: R^2 = 0.9999; average error = 5.11%) and 50 (bottom right: R^2 = 0.9999; average error = 5.35%) K from 0-7 Tesla. The straight lines are linear regressions of the data points. Missing field measurements are due to measurement error. Errors derive from the standard deviation of the sampling distribution of the points used (sample population divided by the square root of the number of samples) to compute the reported moment represented as a percentage.

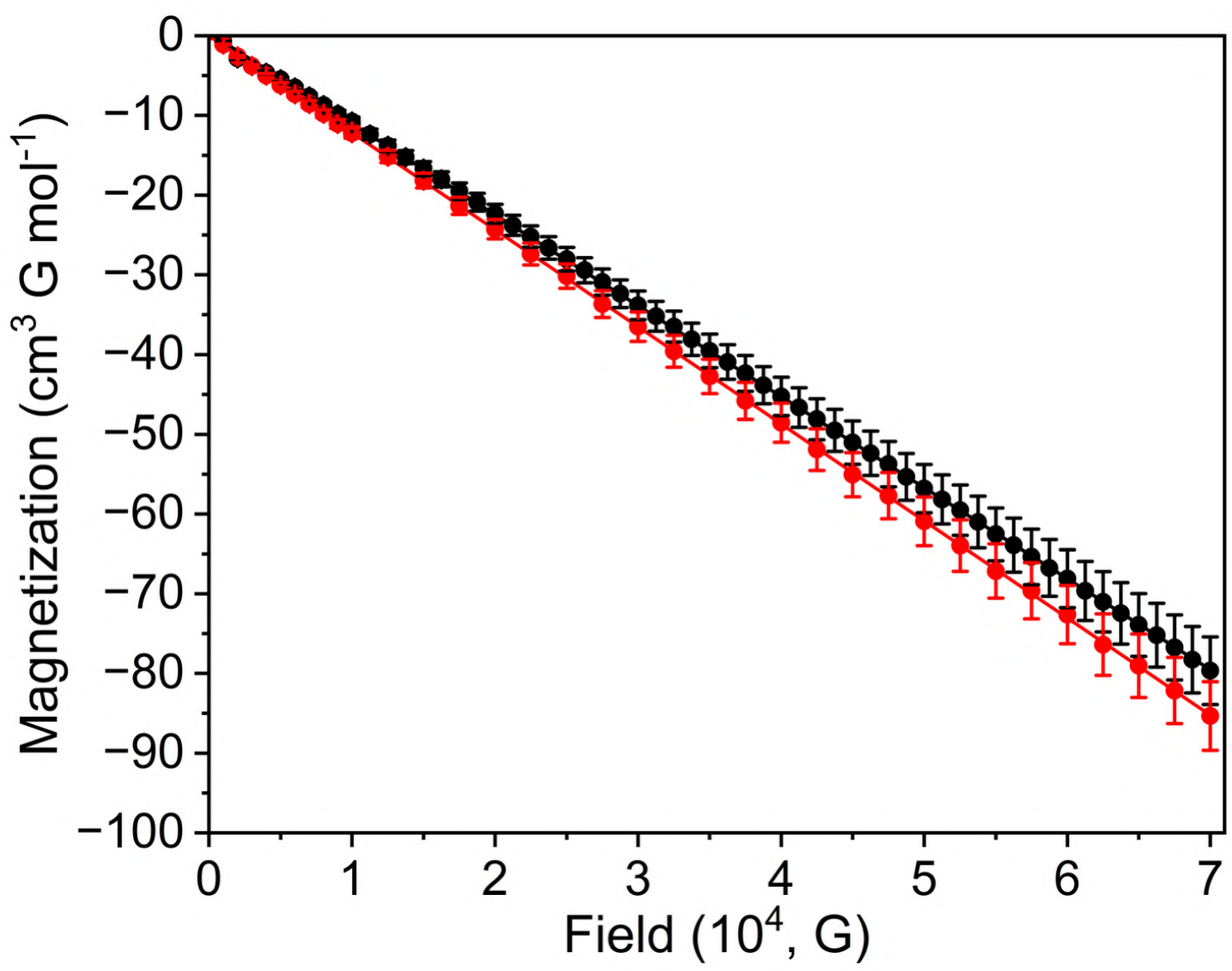

Supplementary Figure 134. Comparative Magnetization vs Field data for **5K** (black: average error = 5.32%) and **5K-eico** (red: average error = 5.05%) at 300 K from 0-7 Tesla. The straight lines are linear regressions of the data points. On a per molecule basis, $\chi_D = -1141.53 \times 10^{-6}$ cm^3 mol^{-1} (R^2 = 0.9999) for **5K** and -1217.58×10^{-6} cm^3 mol^{-1} (R^2 = 0.9999) for **5K-eico**; this is a percentage difference of 6.45%. Errors derive from the standard deviation of the sampling distribution of the points used (sample population divided by the square root of the number of samples) to compute the reported moment represented as a percentage.

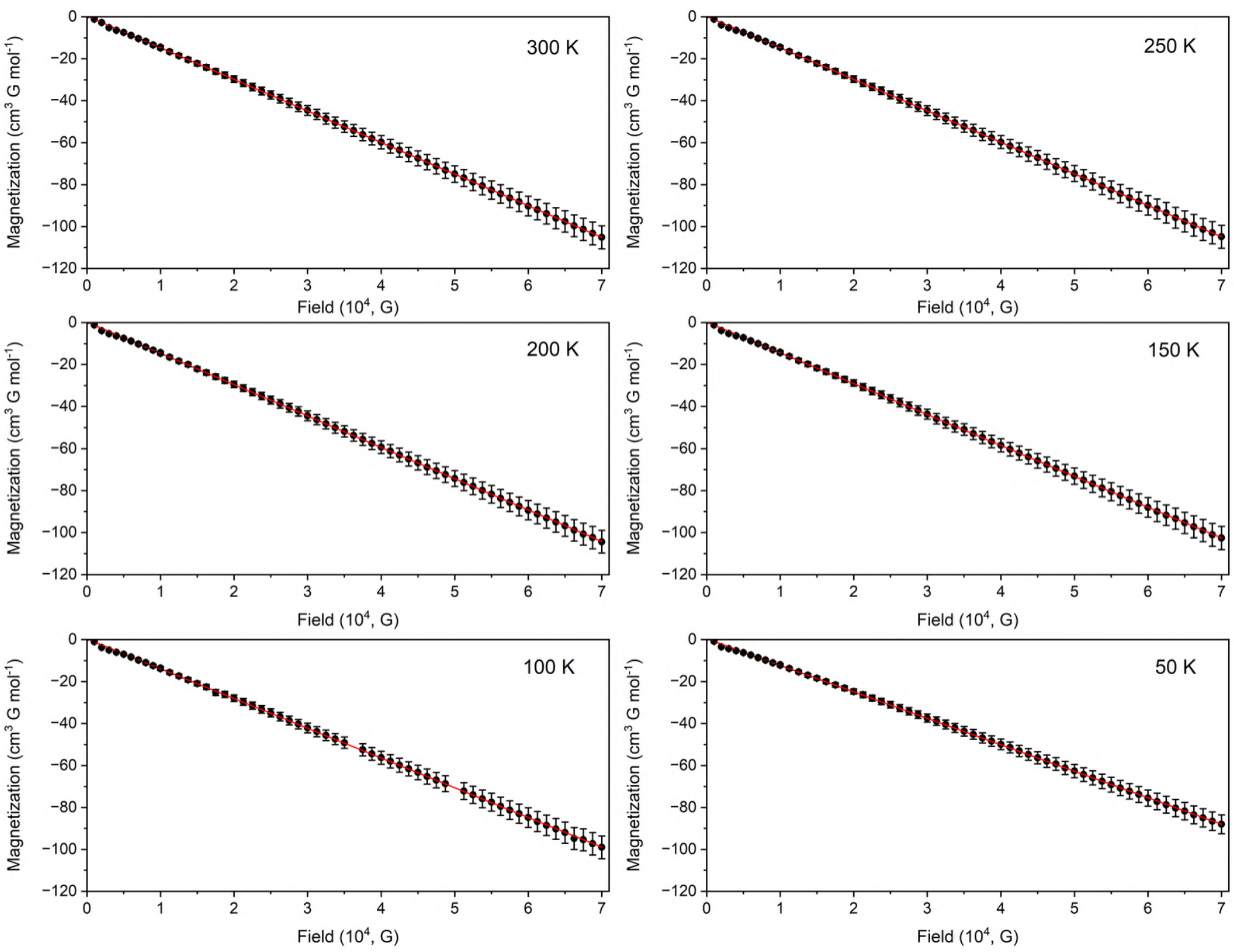

Supplementary Figure 135. Magnetization vs Field data for **5Rb** at 300 (top left: R^2 = 0.9999; average error = 5.24%), 250 (top right: R^2 = 0.9999; average error = 5.18%), 200 (middle left: R^2 = 0.9999; average error = 5.14%), 150 (middle right: R^2 = 0.9999; average error = 5.34%), 100 (bottom left: R^2 = 0.9999; average error = 5.50%) and 50 (bottom right: R^2 = 0.9999; average error = 5.11%) K from 0-7 Tesla. The straight lines are linear regressions of the data points. Missing field measurements are due to measurement error. Errors derive from the standard deviation of the sampling distribution of the points used (sample population divided by the square root of the number of samples) to compute the reported moment represented as a percentage.

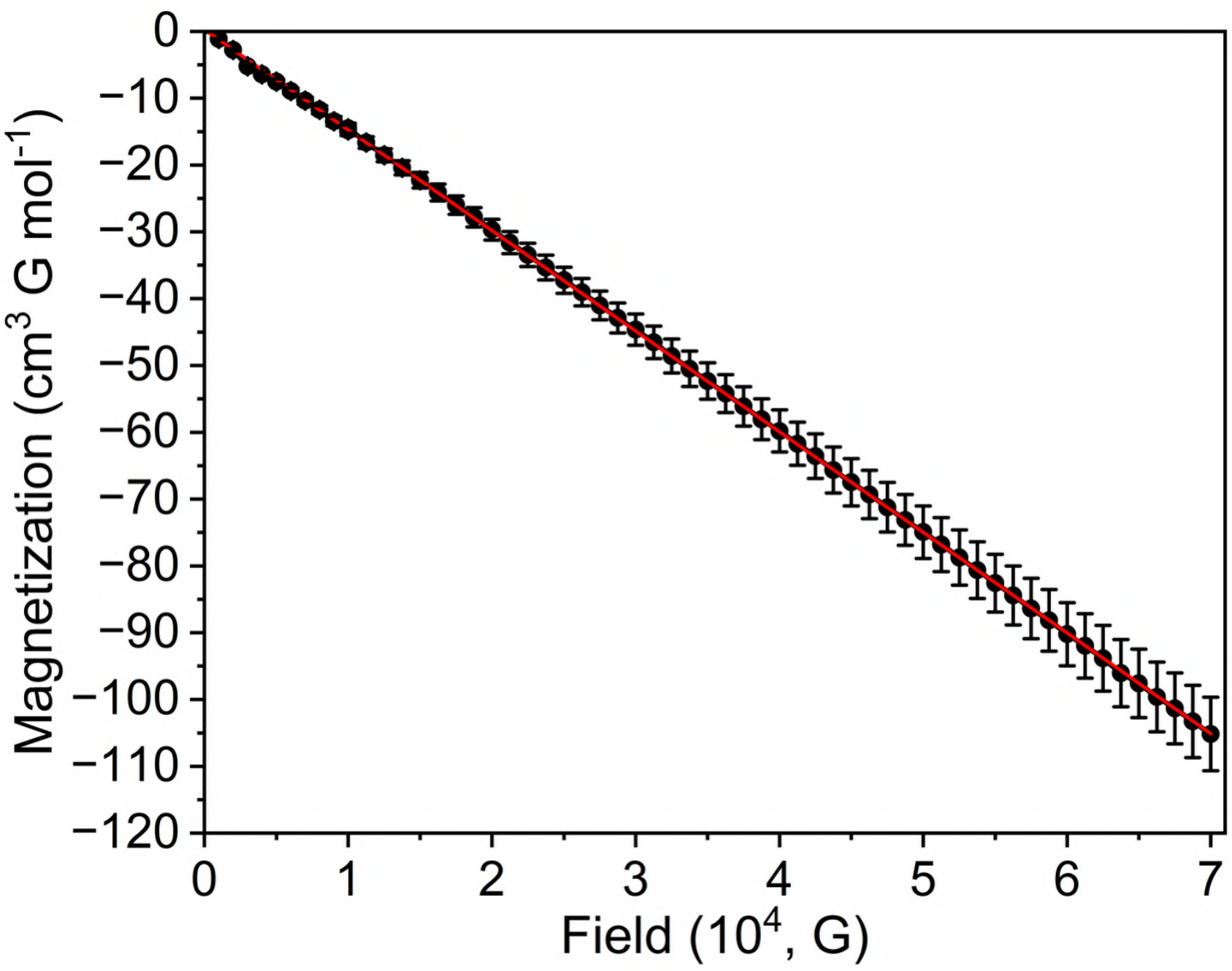

Supplementary Figure 136. Magnetization vs Field data for **5Rb** (black: average error = 5.24%) at 300 K from 0-7 Tesla. The straight lines are linear regressions of the data points. On a per molecule basis, χ_D = –1505.39 × 10^{-6} cm^3 mol^{-1} (R^2 = 0.9999) for **5Rb**. Errors derive from the standard deviation of the sampling distribution of the points used (sample population divided by the square root of the number of samples) to compute the reported moment represented as a percentage.

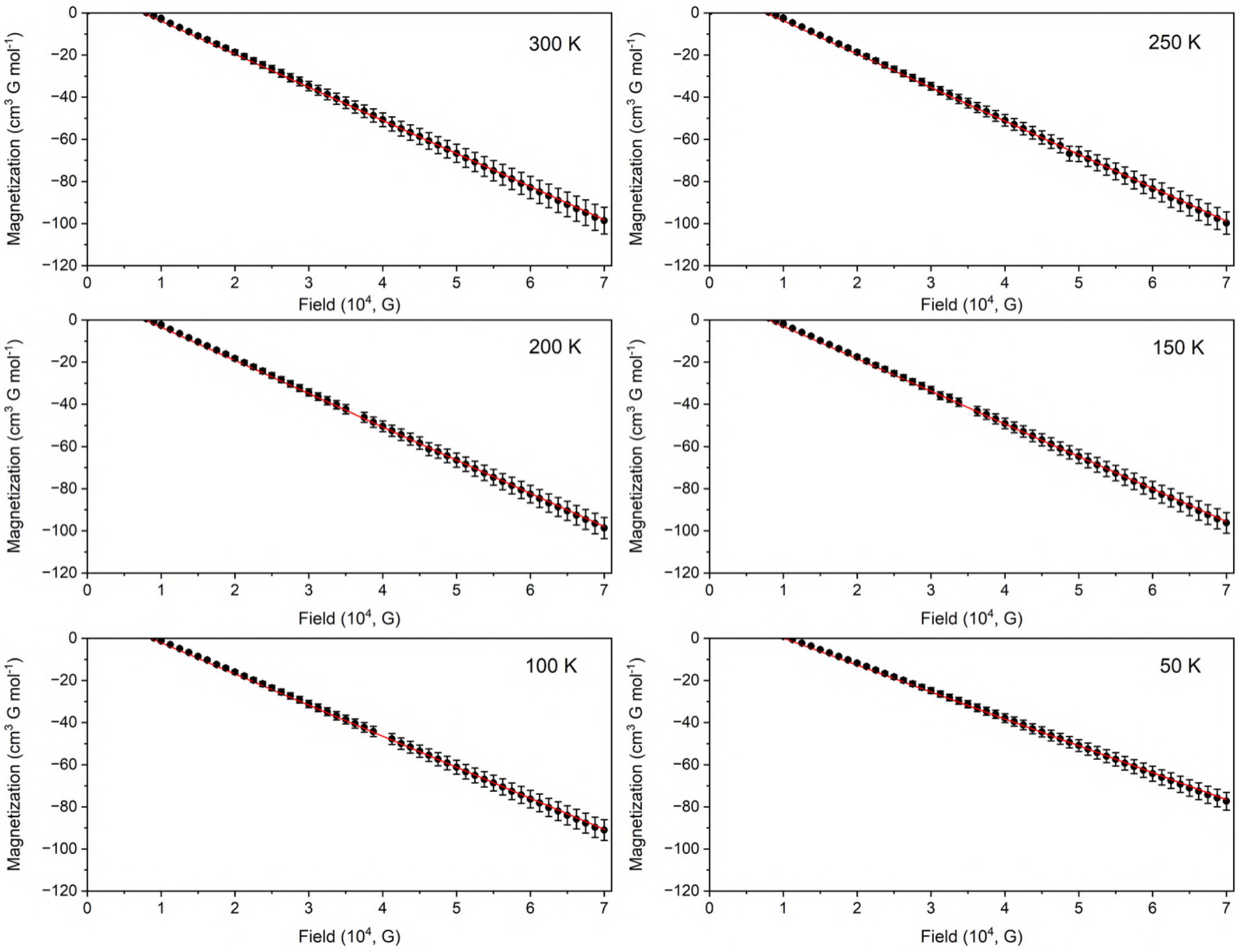

Supplementary Figure 137. Magnetization vs Field data for **5Cs** at 300 (top left: R^2 = 0.9977; average error = 6.42%), 250 (top right: R^2 = 0.9977; average error = 5.31%), 200 (middle left: R^2 = 0.9976; average error = 5.04%), 150 (middle right: R^2 = 0.9975; average error = 5.07%), 100 (bottom left: R^2 = 0.9972; average error = 5.42%) and 50 (bottom right: R^2 = 0.9960; average error = 5.48%) K from 0-7 Tesla. The straight lines are linear regressions of the data points. Missing field measurements are due to measurement error. Errors derive from the standard deviation of the sampling distribution of the points used (sample population divided by the square root of the number of samples) to compute the reported moment represented as a percentage.

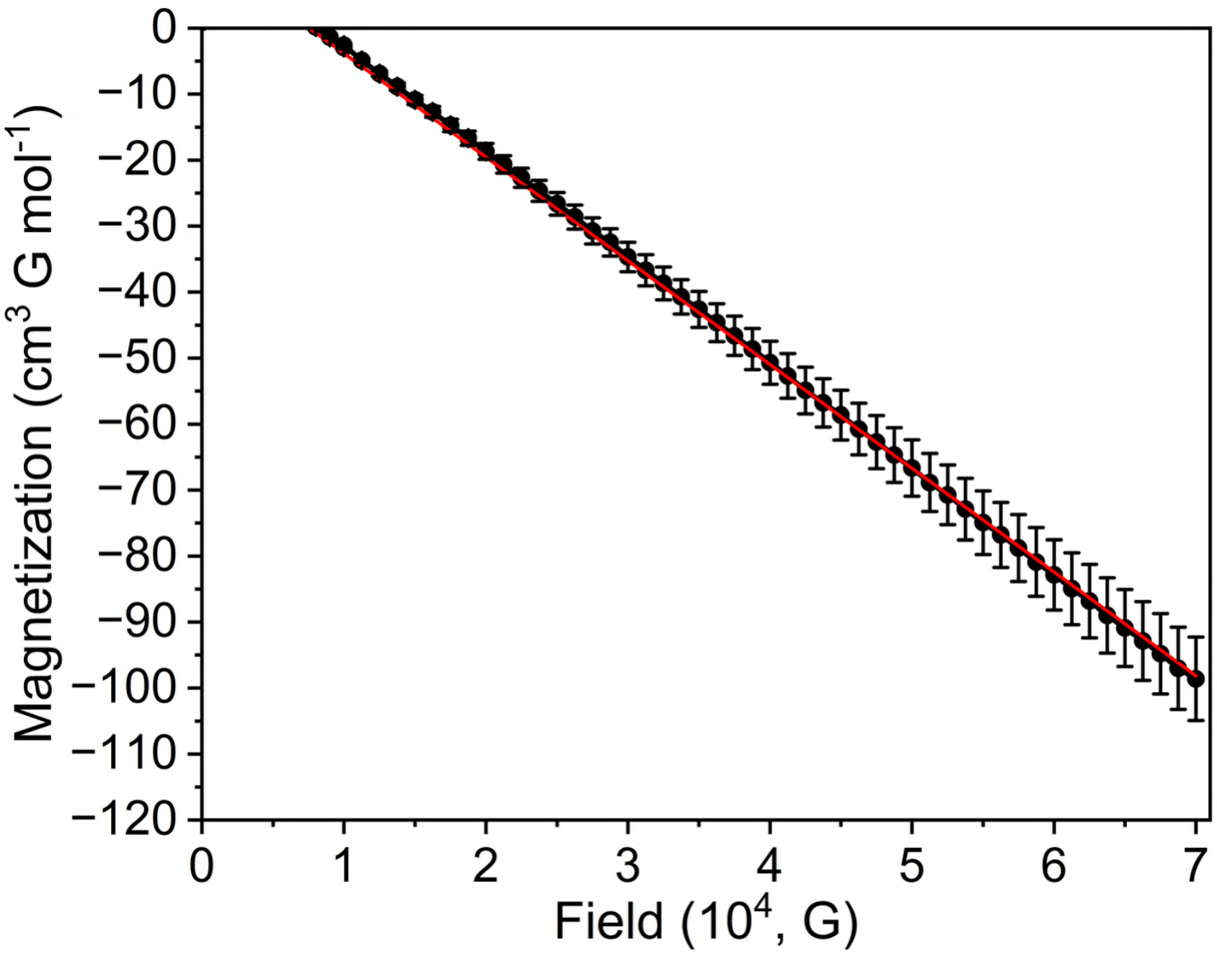

Supplementary Figure 138. Magnetization vs Field data for **5Cs** (black: average error = 6.42%) at 300 K from 0-7 Tesla. The straight lines are linear regressions of the data points. On a per molecule basis, $\chi_D = -1571.97 \times 10^{-6}$ cm^3 mol^{-1} (R^2 = 0.9977) for **5Cs**. Errors derive from the standard deviation of the sampling distribution of the points used (sample population divided by the square root of the number of samples) to compute the reported moment represented as a percentage.

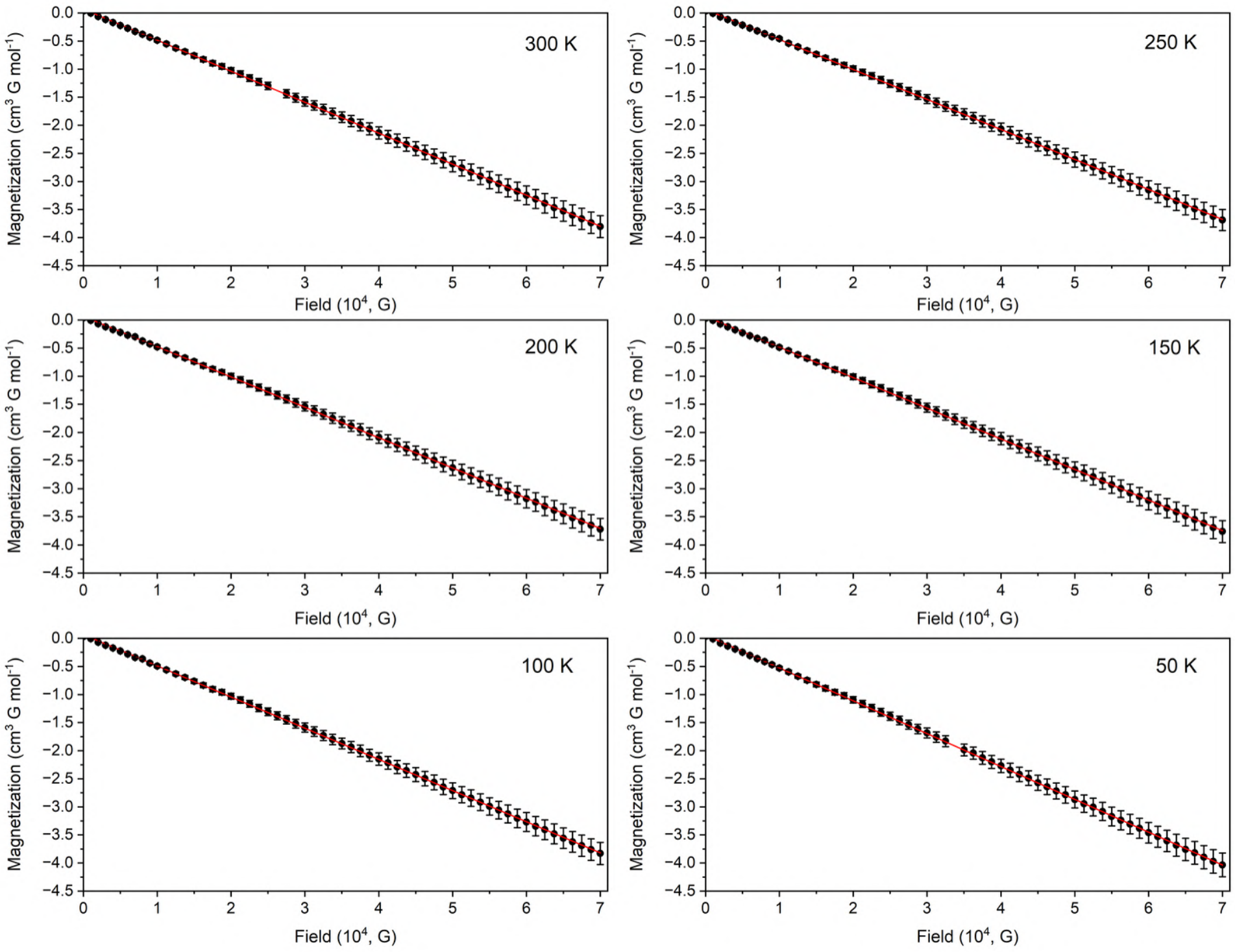

Supplementary Figure 139. Magnetization vs Field data for benzene at 300 (top left: R^2 = 0.9999; average error = 5.13%), 250 (top right: R^2 = 0.9999; average error = 5.14%), 200 (middle left: R^2 = 0.9999; average error = 5.13%), 150 (middle right: R^2 = 0.9999; average error = 5.14%), 100 (bottom left: R^2 = 0.9999; average error = 5.14%) and 50 (bottom right: R^2 = 0.9999; average error = 5.27%) K from 0-7 Tesla. The straight lines are linear regressions of the data points. Missing field measurements are due to measurement error. Errors derive from the standard deviation of the sampling distribution of the points used (sample population divided by the square root of the number of samples) to compute the reported moment represented as a percentage.

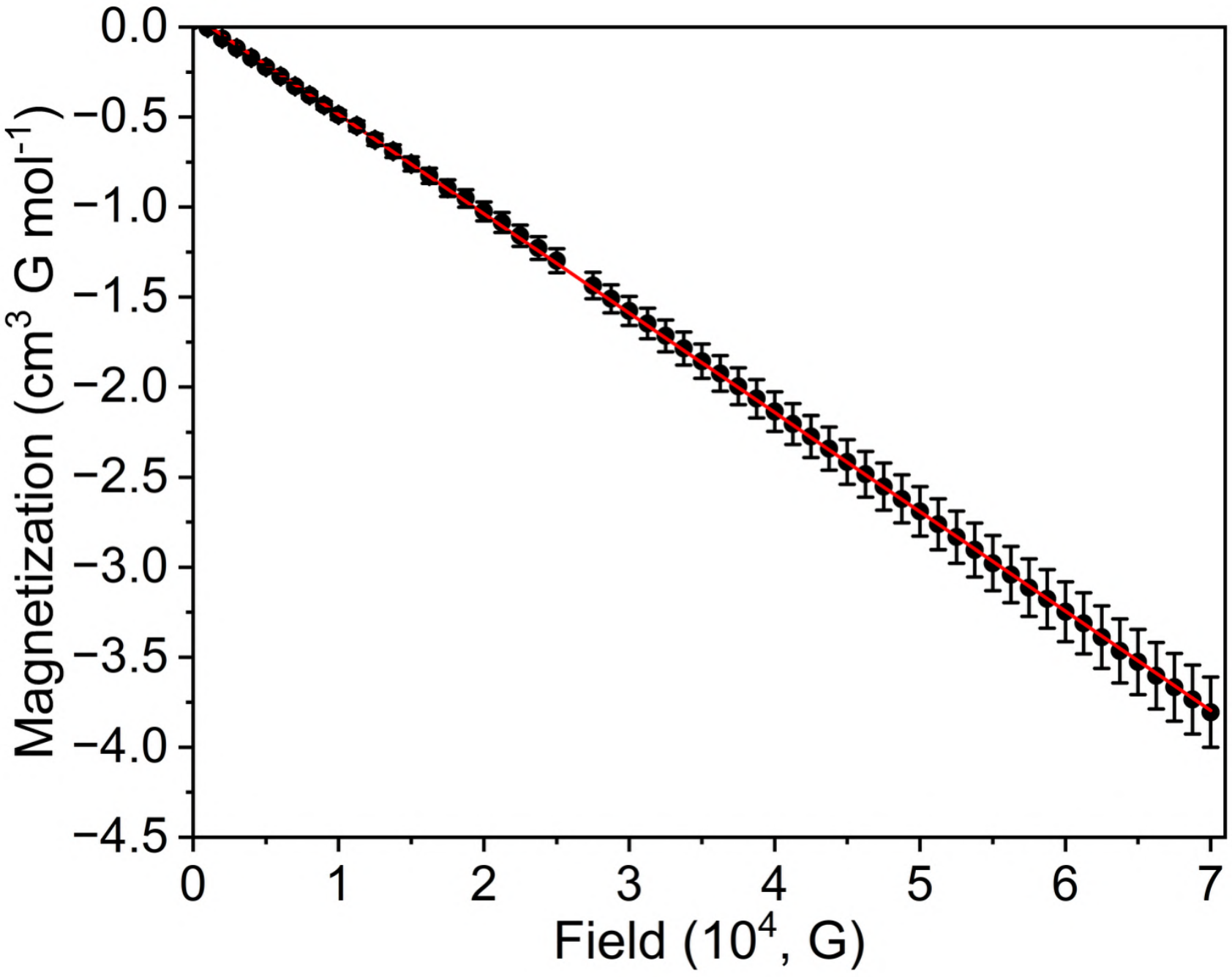

Supplementary Figure 140. Magnetization vs Field data for benzene (black: average error = 5.13%) at 300 K from 0-7 Tesla. The straight lines are linear regressions of the data points. On a per molecule basis, $\chi_D = -55.18 \times 10^{-6}$ cm^3 mol^{-1} (R^2 = 0.9999) for benzene. Errors derive from the standard deviation of the sampling distribution of the points used (sample population divided by the square root of the number of samples) to compute the reported moment represented as a percentage.

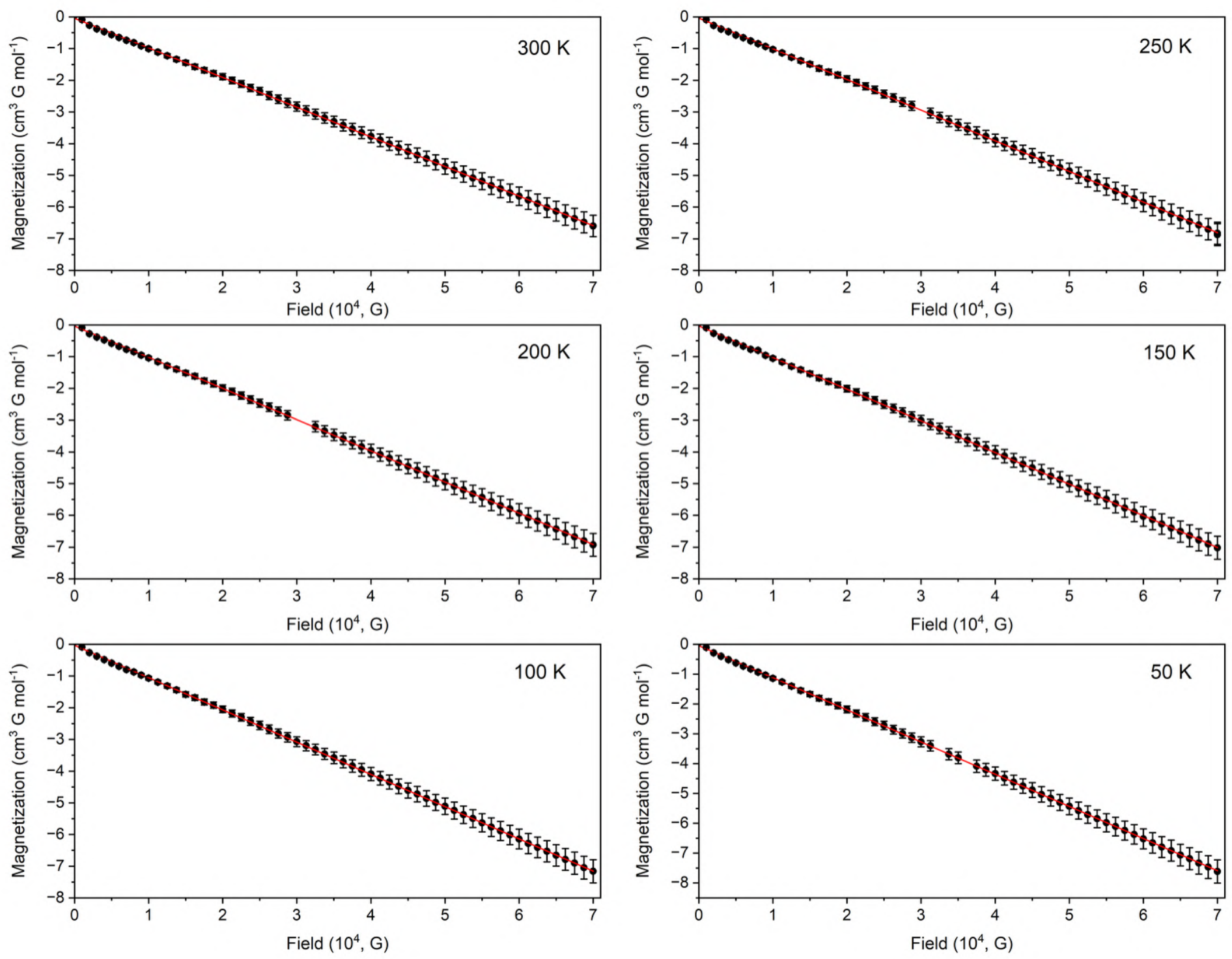

Supplementary Figure 141. Magnetization vs Field data for naphthalene at 300 (top left: R^2 = 0.9999; average error = 5.14%), 250 (top right: R^2 = 0.9999; average error = 5.07%), 200 (middle left: R^2 = 0.9999; average error = 5.12%), 150 (middle right: R^2 = 0.9999; average error = 5.11%), 100 (bottom left: R^2 = 0.9999; average error = 5.10%) and 50 (bottom right: R^2 = 0.9999; average error = 5.11%) K from 0-7 Tesla. The straight lines are linear regressions of the data points. Missing field measurements are due to measurement error. Errors derive from the standard deviation of the sampling distribution of the points used (sample population divided by the square root of the number of samples) to compute the reported moment represented as a percentage.

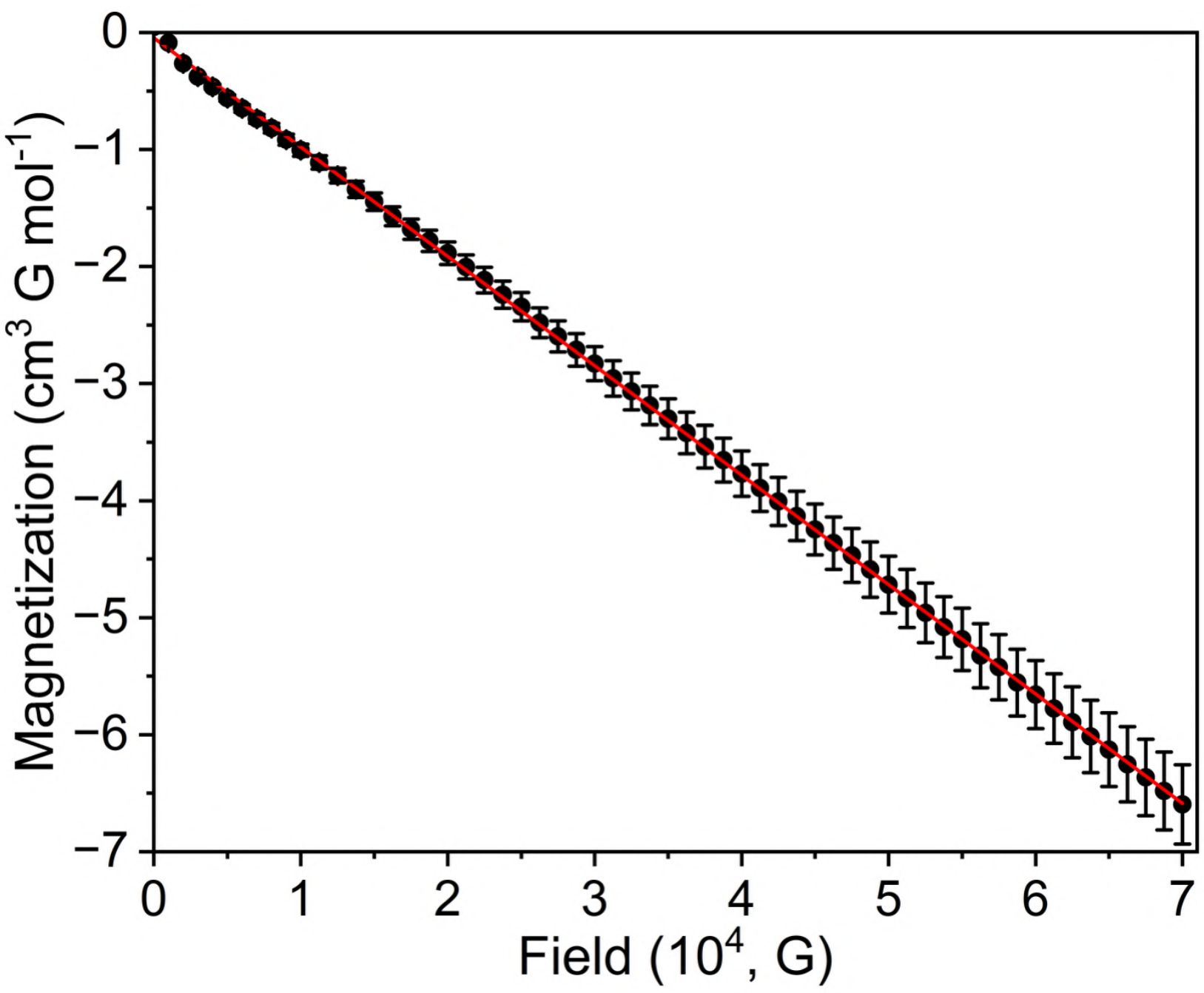

Supplementary Figure 142. Magnetization vs Field data for naphthalene (black: average error = 5.14%) at 300 K from 0-7 Tesla. The straight lines are linear regressions of the data points. On a per molecule basis, $\chi_D = -93.50 \times 10^{-6}$ cm^3 mol^{-1} (R^2 = 0.9999) for naphthalene. Errors derive from the standard deviation of the sampling distribution of the points used (sample population divided by the square root of the number of samples) to compute the reported moment represented as a percentage.

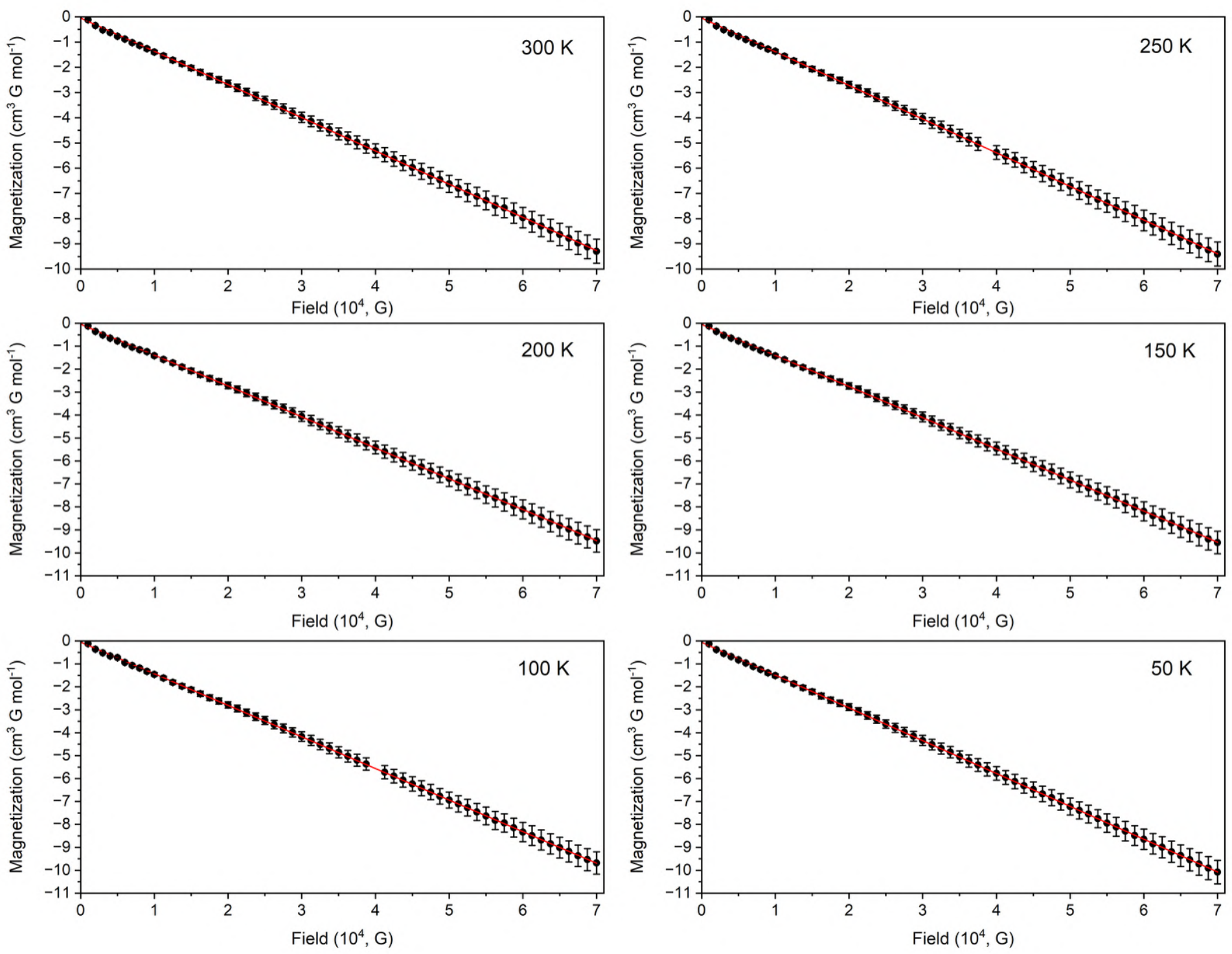

Supplementary Figure 143. Magnetization vs Field data for anthracene at 300 (top left: R^2 = 0.9999; average error = 5.13%), 250 (top right: R^2 = 0.9999; average error = 5.11%), 200 (middle left: R^2 = 0.9999; average error = 5.12%), 150 (middle right: R^2 = 0.9999; average error = 5.11%), 100 (bottom left: R^2 = 0.9999; average error = 5.03%) and 50 (bottom right: R^2 = 0.9999; average error = 5.09%) K from 0-7 Tesla. The straight lines are linear regressions of the data points. Missing field measurements are due to measurement error. Errors derive from the standard deviation of the sampling distribution of the points used (sample population divided by the square root of the number of samples) to compute the reported moment represented as a percentage.

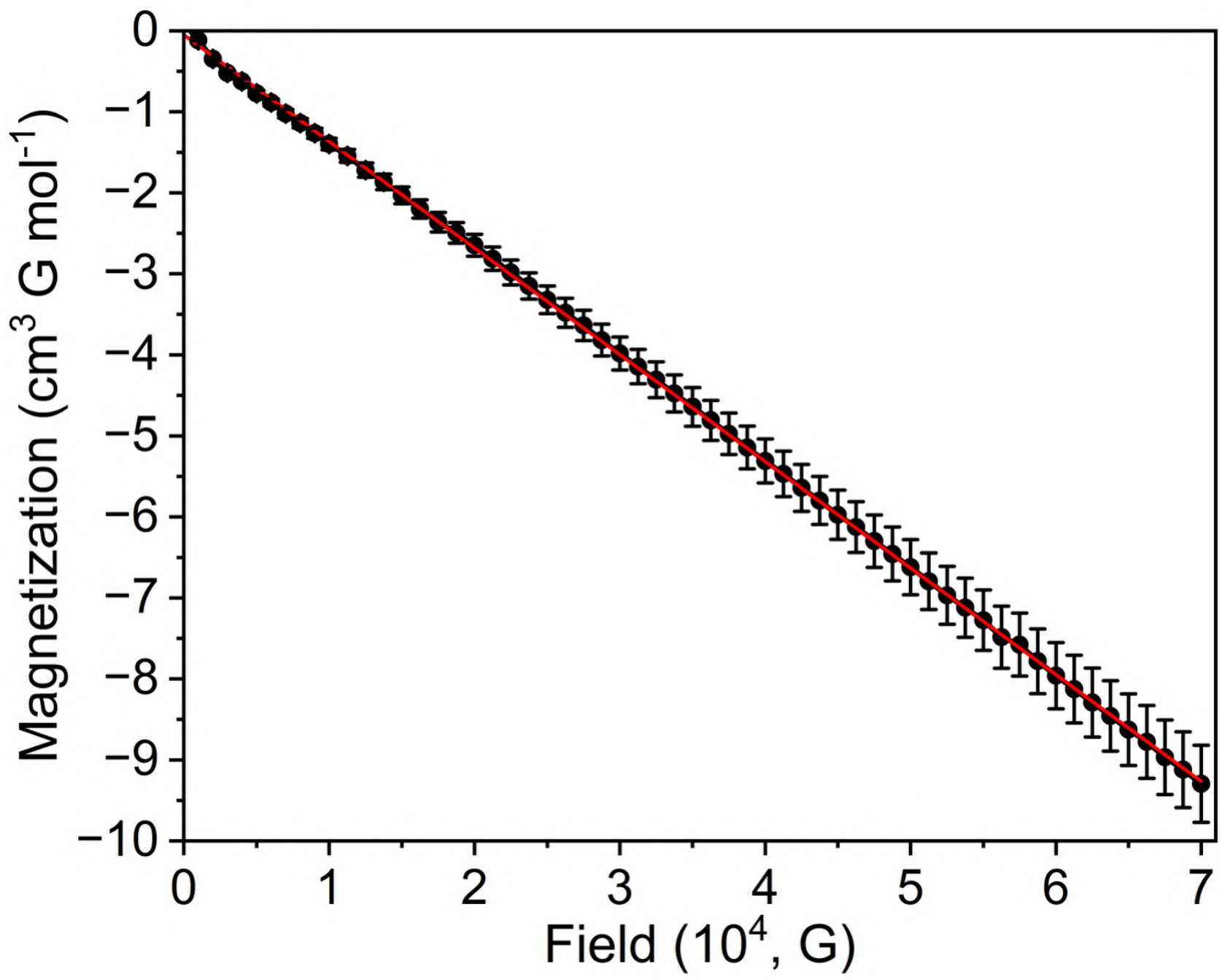

Supplementary Figure 144. Magnetization vs Field data for anthracene (black: average error = 5.13%) at 300 K from 0-7 Tesla. The straight lines are linear regressions of the data points. On a per molecule basis, $\chi_D = -131.37 \times 10^{-6}$ cm^3 mol^{-1} (R^2 = 0.9999) for anthracene. Errors derive from the standard deviation of the sampling distribution of the points used (sample population divided by the square root of the number of samples) to compute the reported moment represented as a percentage.

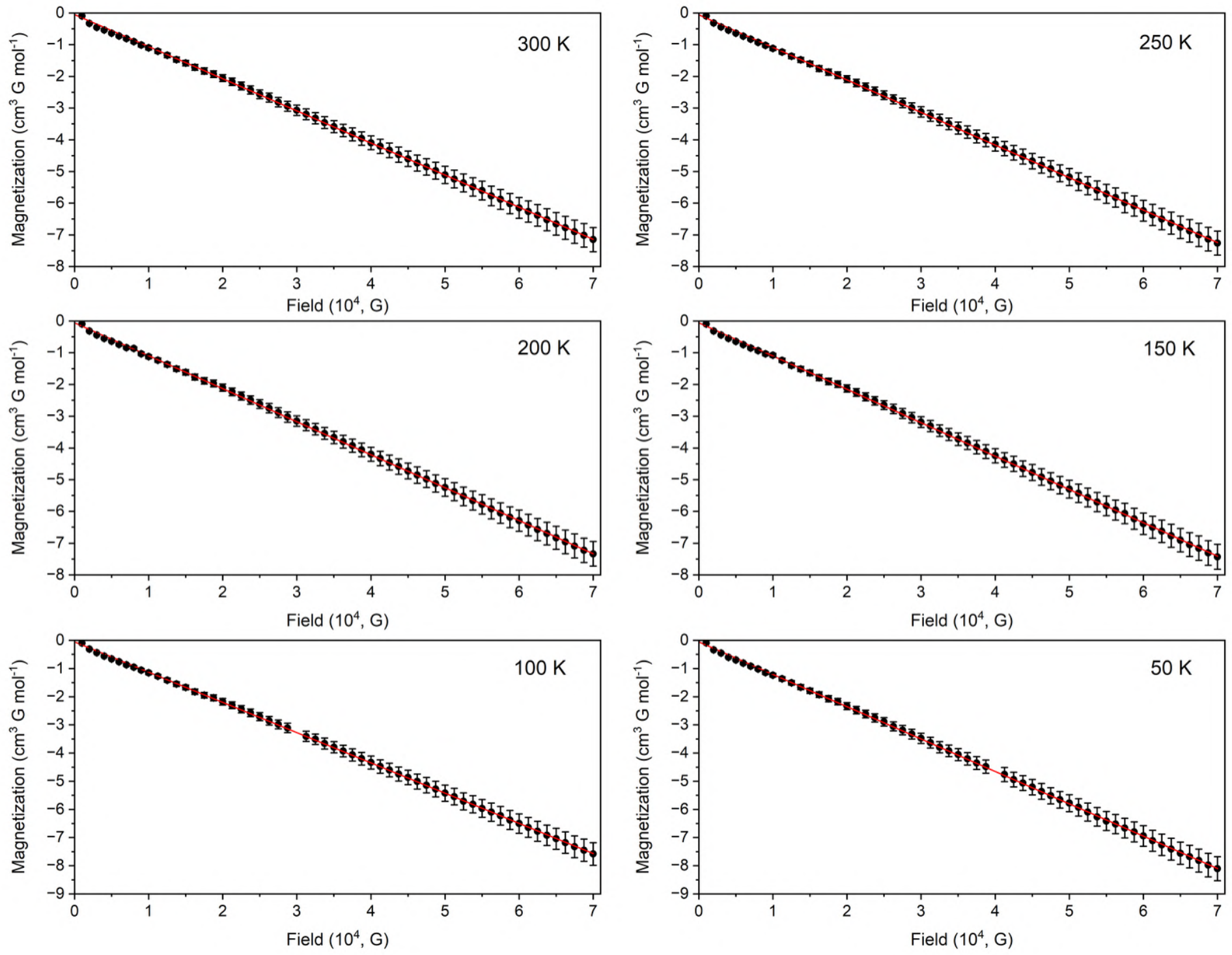

Supplementary Figure 145. Magnetization vs Field data for biphenyl at 300 (top left: R^2 = 0.9999; average error = 5.33%), 250 (top right: R^2 = 0.9999; average error = 5.23%), 200 (middle left: R^2 = 0.9999; average error = 5.30%), 150 (middle right: R^2 = 0.9999; average error = 5.30%), 100 (bottom left: R^2 = 0.9999; average error = 5.28%) and 50 (bottom right: R^2 = 0.9999; average error = 5.25%) K from 0-7 Tesla. The straight lines are linear regressions of the data points. Missing field measurements are due to measurement error. Errors derive from the standard deviation of the sampling distribution of the points used (sample population divided by the square root of the number of samples) to compute the reported moment represented as a percentage.

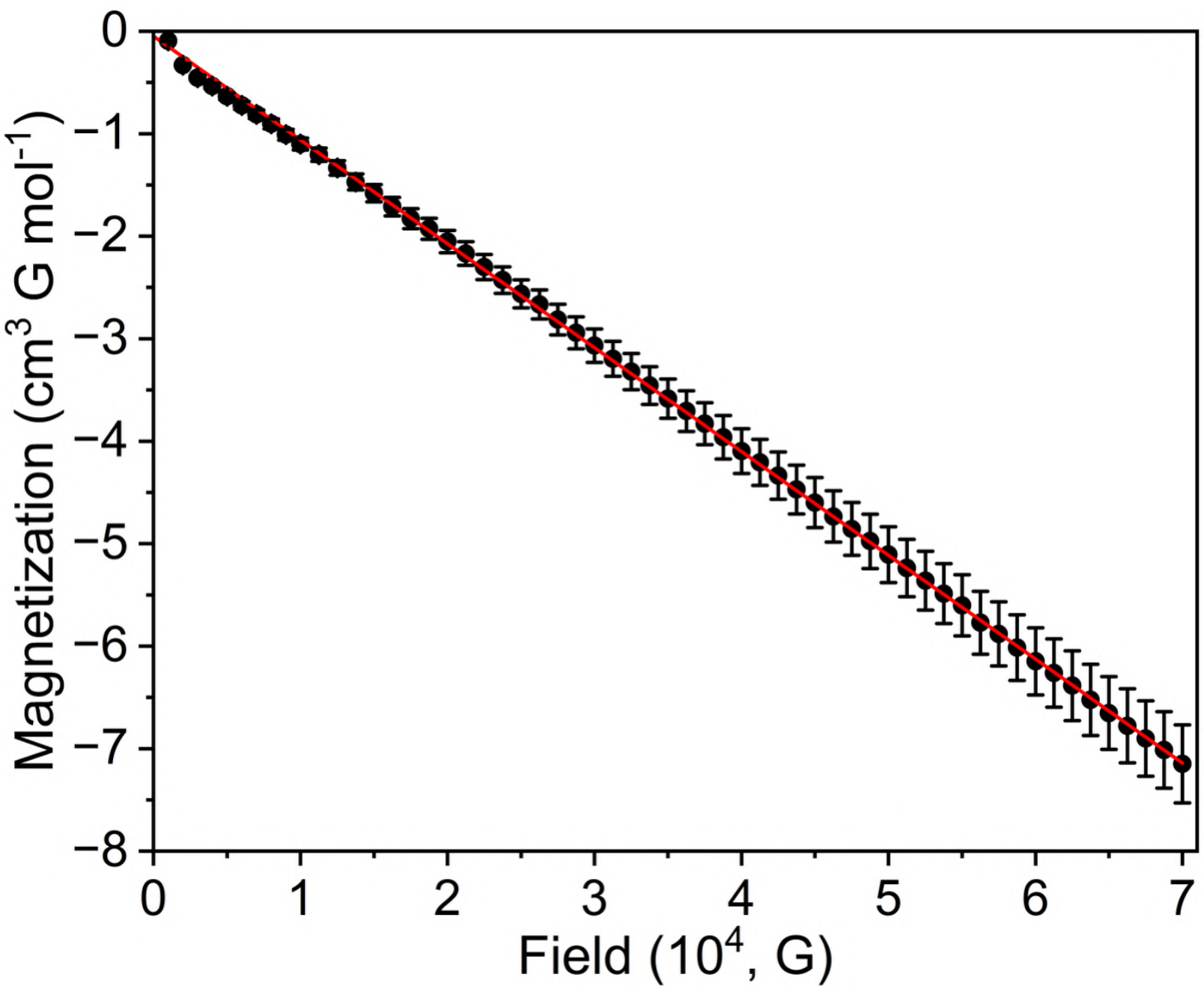

Supplementary Figure 146. Magnetization vs Field data for biphenyl (black: average error = 5.33%) at 300 K from 0-7 Tesla. The straight lines are linear regressions of the data points. On a per molecule basis, $\chi_D = -101.36 \times 10^{-6}$ cm^3 mol^{-1} (R^2 = 0.9999) for biphenyl. Errors derive from the standard deviation of the sampling distribution of the points used (sample population divided by the square root of the number of samples) to compute the reported moment represented as a percentage.

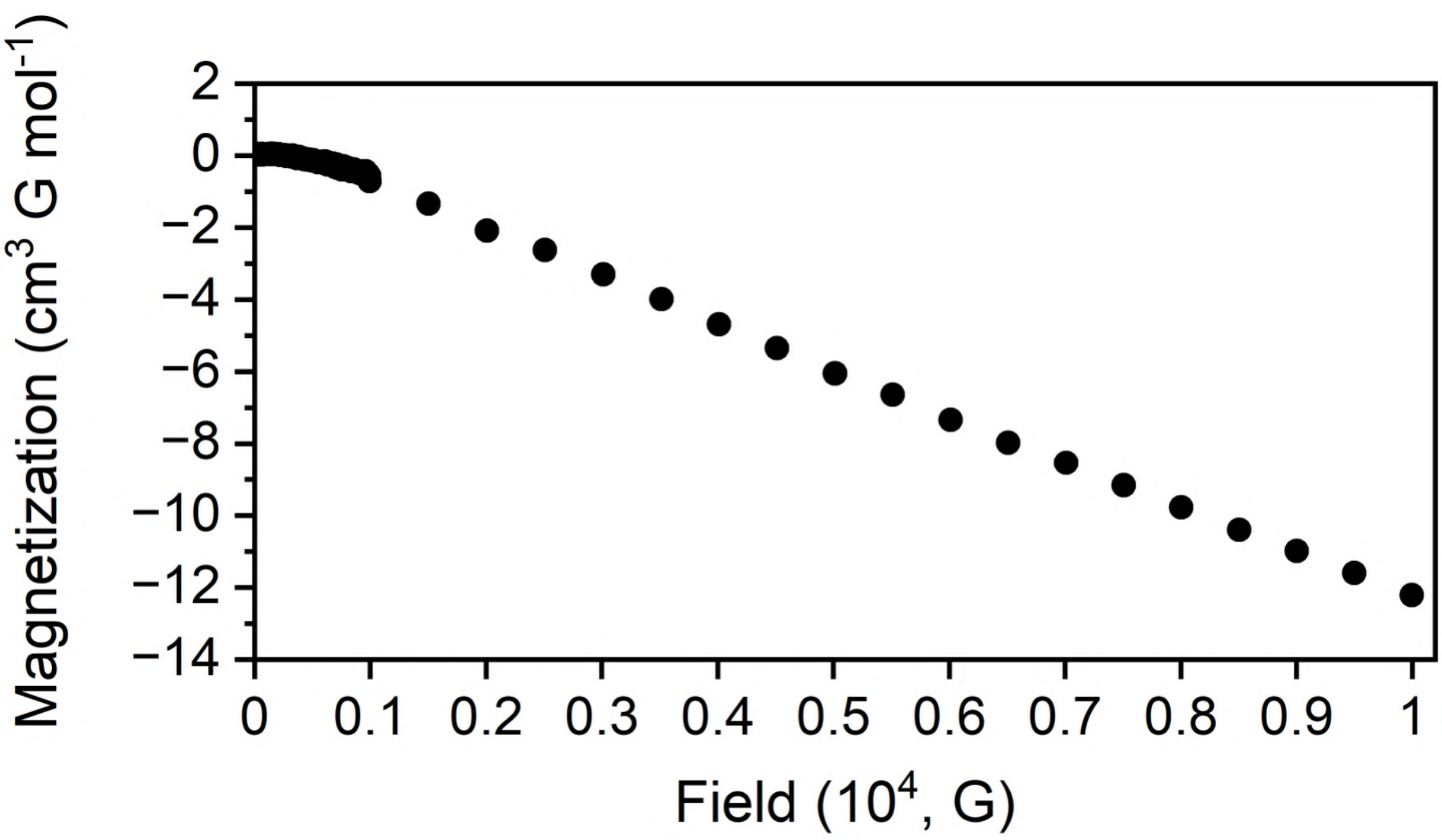

Supplementary Figure 147. Magnetization vs Field data for **4K** at 300 K from 0-1 Tesla. Error bars not displayed for clarity due to large number of data points, especially below 100 Gauss (G). Average percentage errors are as follows: 17.43% (1-69 G) and 6.00% (71-10000 G). The variation in error across the field range derives from the alteration in shape of the raw voltage waveform as the DC moment changes from positive (paramagnetic) to negative (diamagnetic) as the field increases. This change in DC moment moves the raw voltage waveform from a positive single dipole response curve at very low fields, to an asymmetric response curve at intermediate fields, and finally back to a single dipole response curve at higher fields (but now negative), therefore in this intermediate region, the mathematical fit to the raw waveform will be poorer and the error greater. Errors derive from the standard deviation of the sampling distribution of the points used (sample population divided by the square root of the number of samples) to compute the reported moment represented as a percentage.

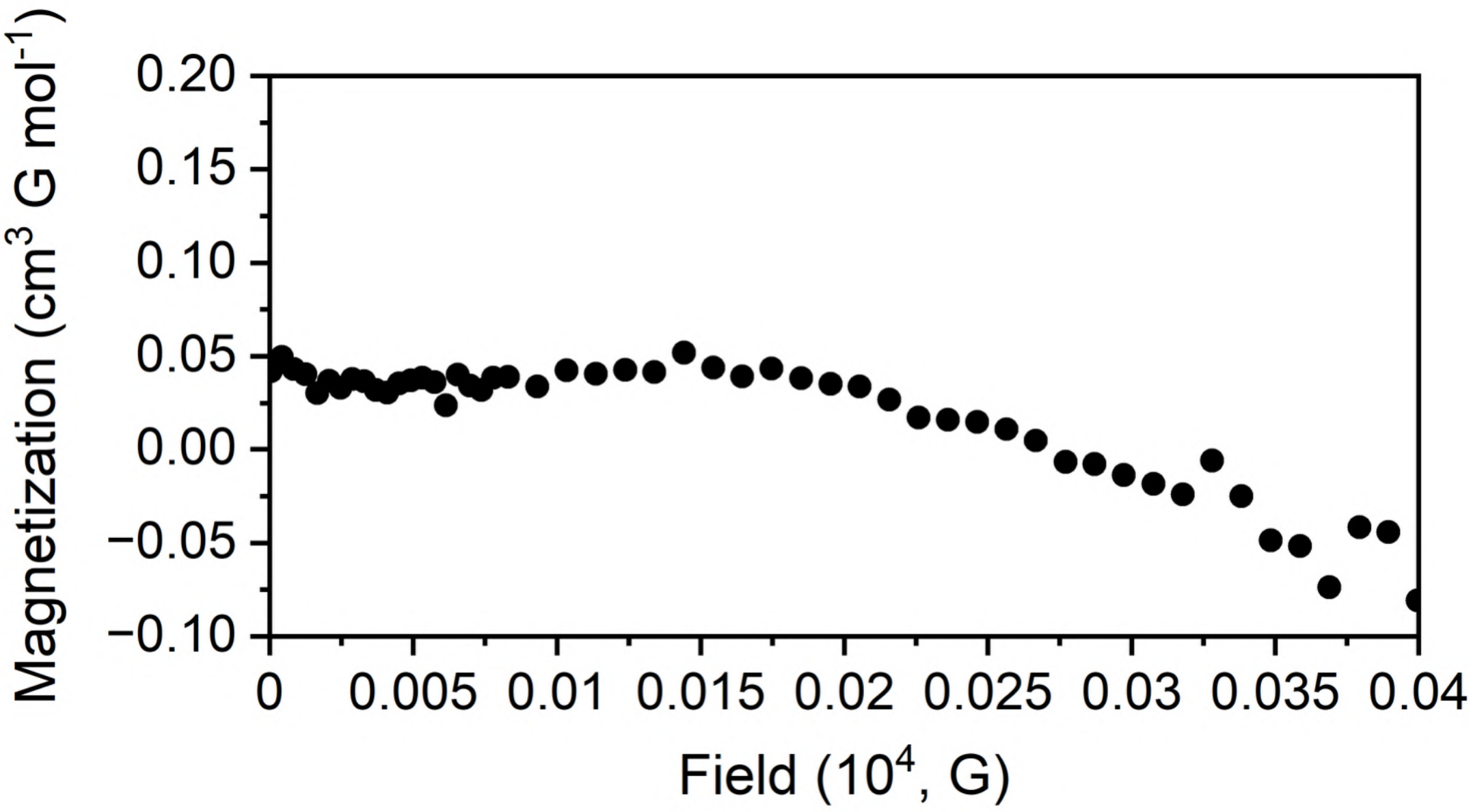

Supplementary Figure 148. Magnetization vs Field data for **4K** at 300 K from 0-0.04 Tesla. Error bars not displayed for clarity due to large number of data points.

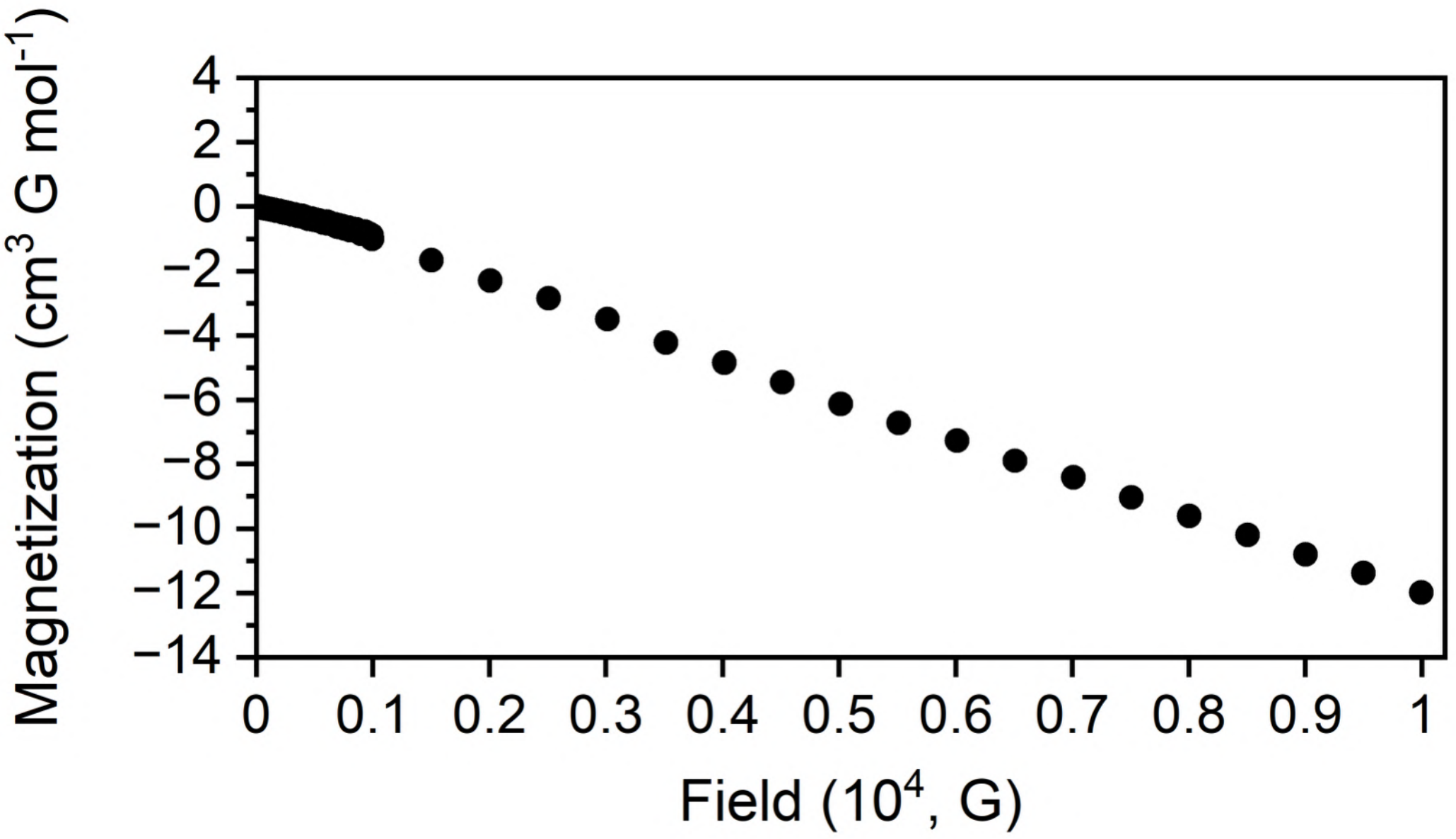

Supplementary Figure 149. Magnetization vs Field data for **4aRb** at 300 K from 0-1 Tesla. Error bars not displayed for clarity due to large number of data points, especially below 100 Gauss (G). Average percentage errors are as follows: 17.11% (1-29 G) and 5.38% (31-10000 G). The variation in error across the field range derives from the alteration in shape of the raw voltage waveform as the DC moment changes from positive (paramagnetic) to negative (diamagnetic) as the field increases. This change in DC moment moves the raw voltage waveform from a positive single dipole response curve at very low fields, to an asymmetric response curve at intermediate fields, and finally back to a single dipole response curve at higher fields (but now negative), therefore in this intermediate region, the mathematical fit to the raw waveform will be poorer and the error greater. Errors derive from the standard deviation of the sampling distribution of the points used (sample population divided by the square root of the number of samples) to compute the reported moment represented as a percentage.

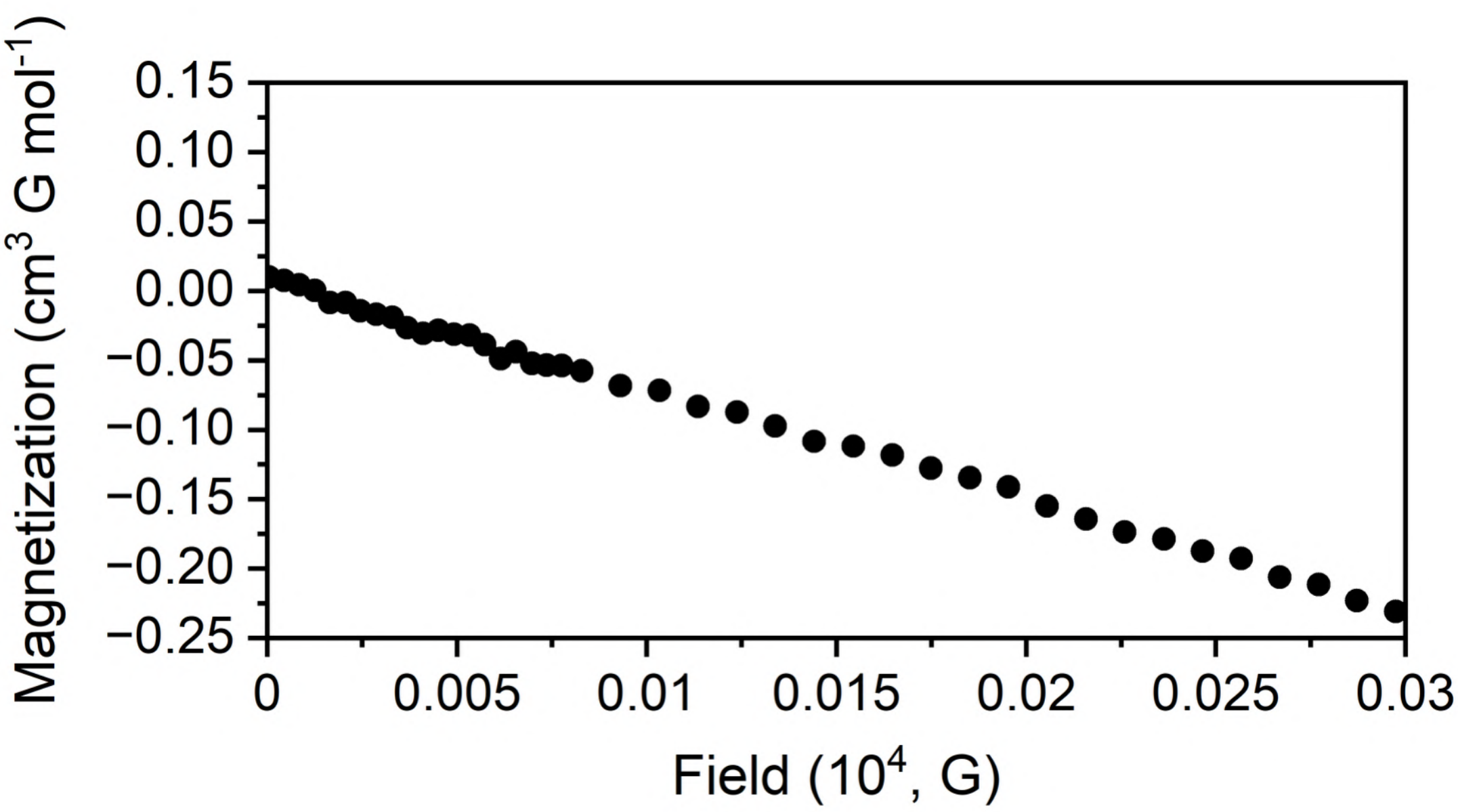

Supplementary Figure 150. Magnetization vs Field data for **4aRb** at 300 K from 0-0.03 Tesla. Error bars not displayed for clarity due to large number of data points.

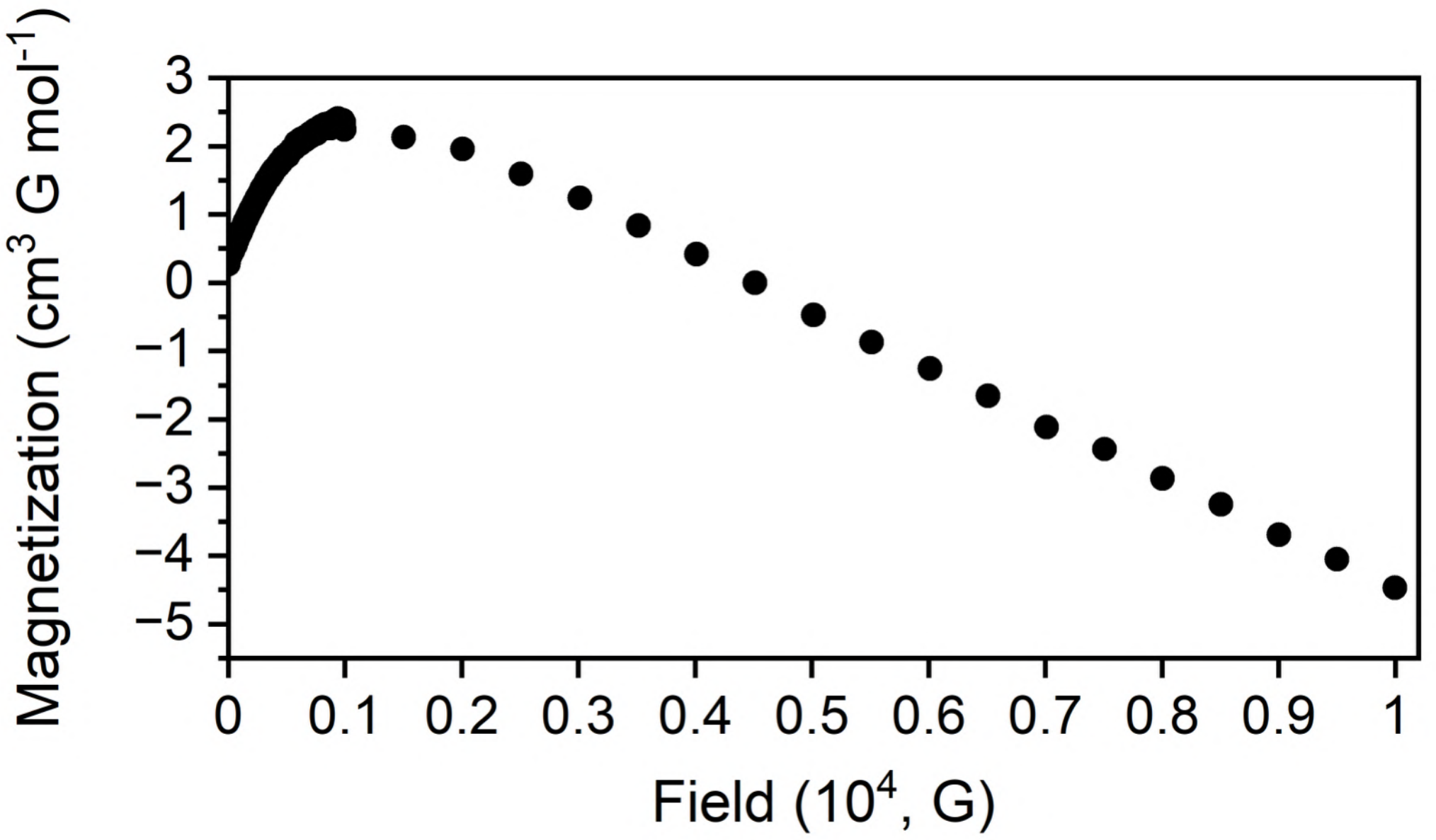

Supplementary Figure 151. Magnetization vs Field data for **4Cs** at 300 K from 0-1 Tesla. Error bars not displayed for clarity due to large number of data points, especially below 100 Gauss (G). Average percentage errors are as follows: 6.69% (2-680 G), 14.78% (685-2000 G), and 6.20% (2250-10000 G). The variation in error across the field range derives from the alteration in shape of the raw voltage waveform as the DC moment changes from positive (paramagnetic) to negative (diamagnetic) as the field increases. This change in DC moment moves the raw voltage waveform from a positive single dipole response curve at very low fields, to an asymmetric response curve at intermediate fields, and finally back to a single dipole response curve at higher fields (but now negative), therefore in this intermediate region, the mathematical fit to the raw waveform will be poorer and the error greater. Errors derive from the standard deviation of the sampling distribution of the points used (sample population divided by the square root of the number of samples) to compute the reported moment represented as a percentage.

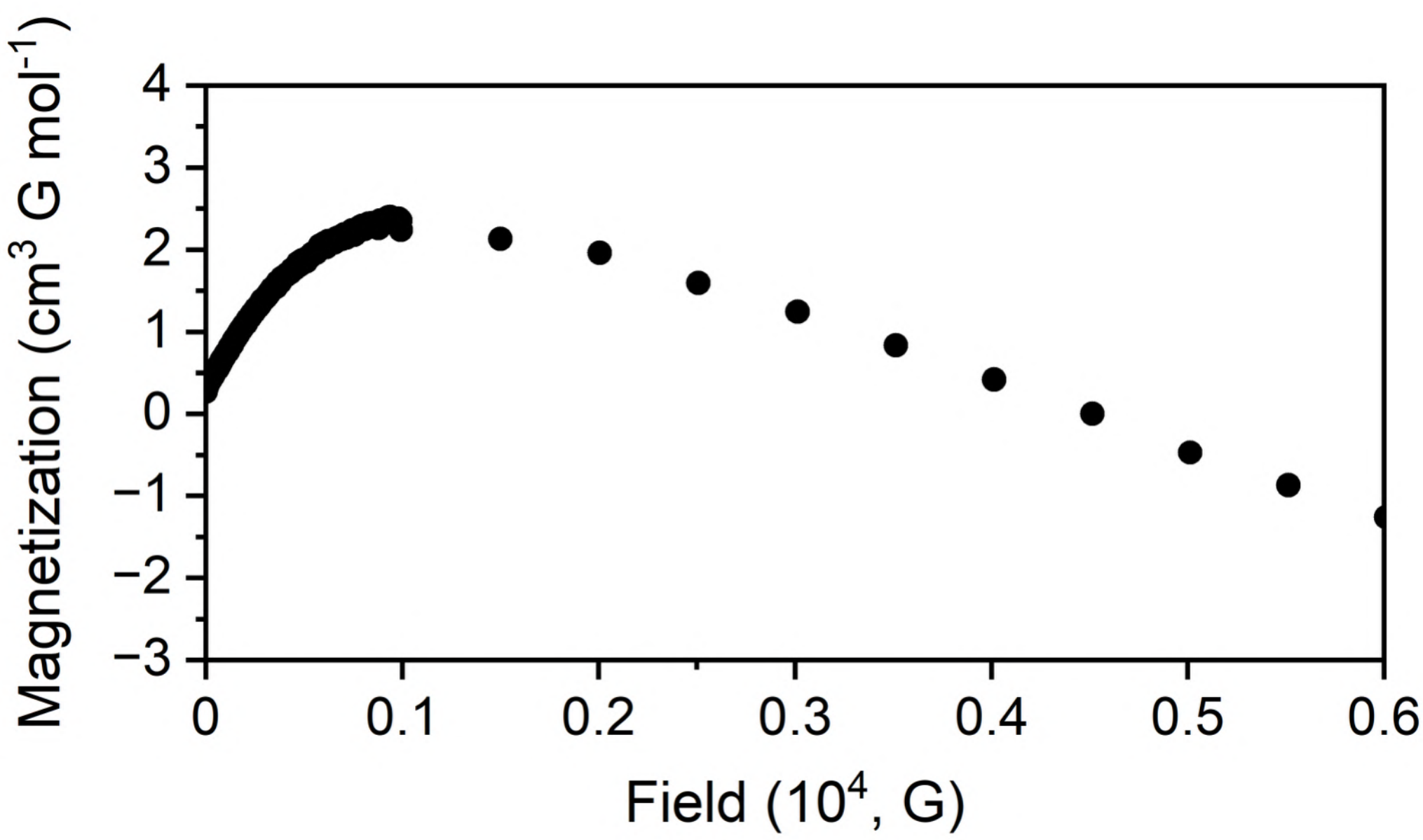

Supplementary Figure 152. Magnetization vs Field data for **4Cs** at 300 K from 0-0.6 Tesla. Error bars not displayed for clarity due to large number of data points.

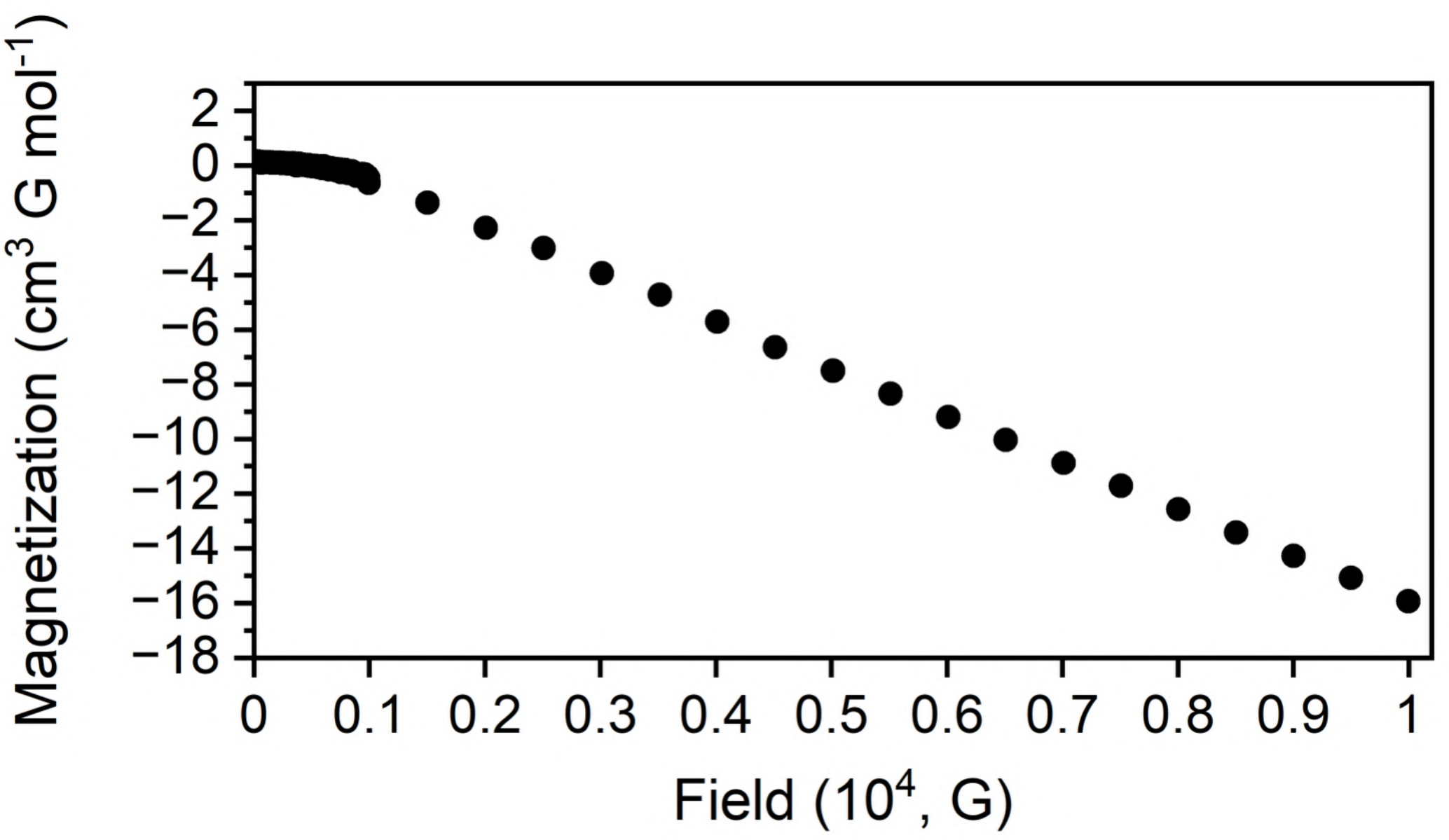

Supplementary Figure 153. Magnetization vs Field data for **5Na** at 300 K from 0-1 Tesla. Error bars not displayed for clarity due to large number of data points, especially below 100 Gauss (G). Average percentage errors are as follows: 7.27% (2-56 G), 35.17% (58-135 G), and 6.10% (140-10000 G). The variation in error across the field range derives from the alteration in shape of the raw voltage waveform as the DC moment changes from positive (paramagnetic) to negative (diamagnetic) as the field increases. This change in DC moment moves the raw voltage waveform from a positive single dipole response curve at very low fields, to an asymmetric response curve at intermediate fields, and finally back to a single dipole response curve at higher fields (but now negative), therefore in this intermediate region, the mathematical fit to the raw waveform will be poorer and the error greater. Errors derive from the standard deviation of the sampling distribution of the points used (sample population divided by the square root of the number of samples) to compute the reported moment represented as a percentage.

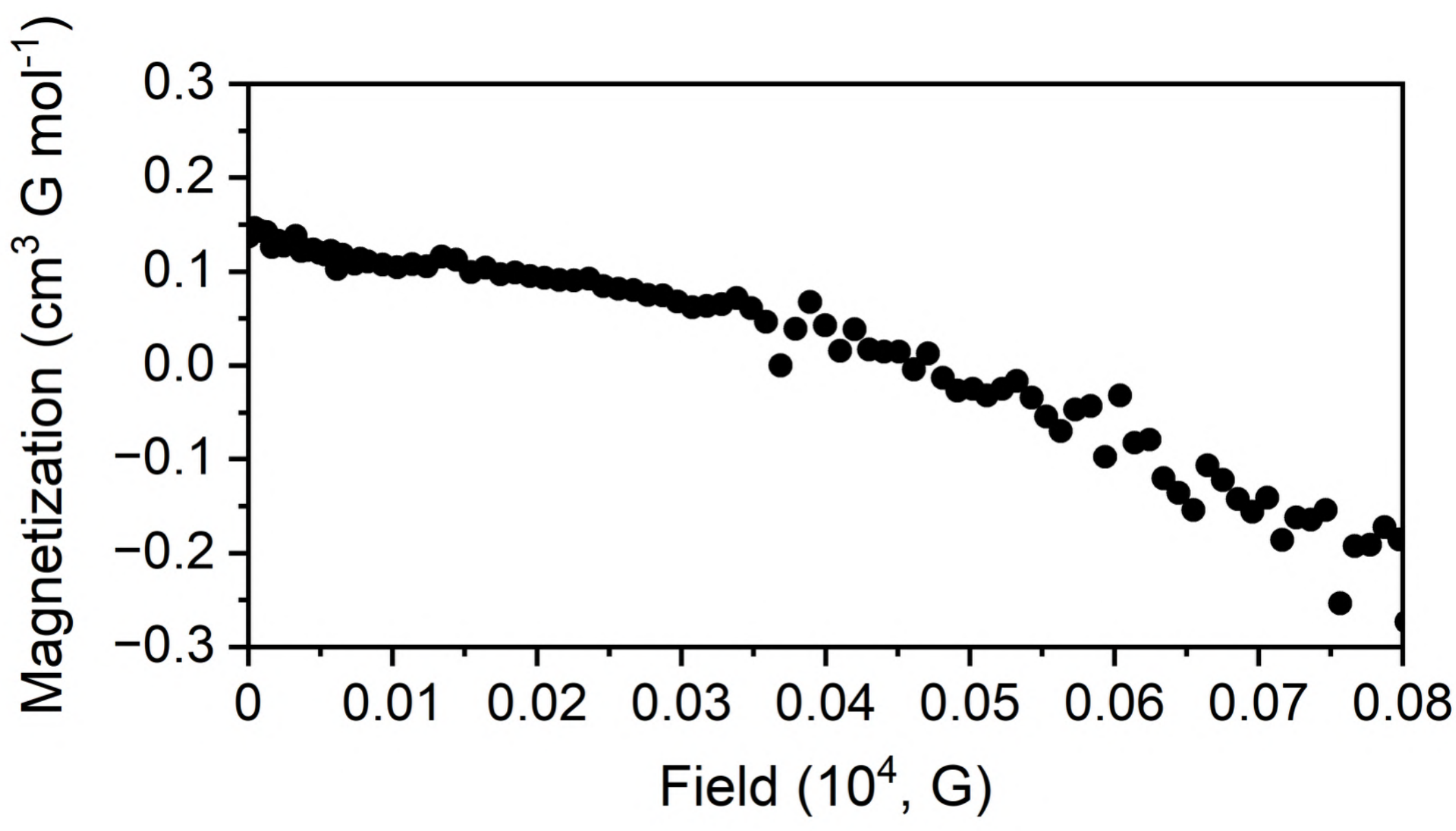

Supplementary Figure 154. Magnetization vs Field data for **5Na** at 300 K from 0-0.08 Tesla. Error bars not displayed for clarity due to large number of data points.

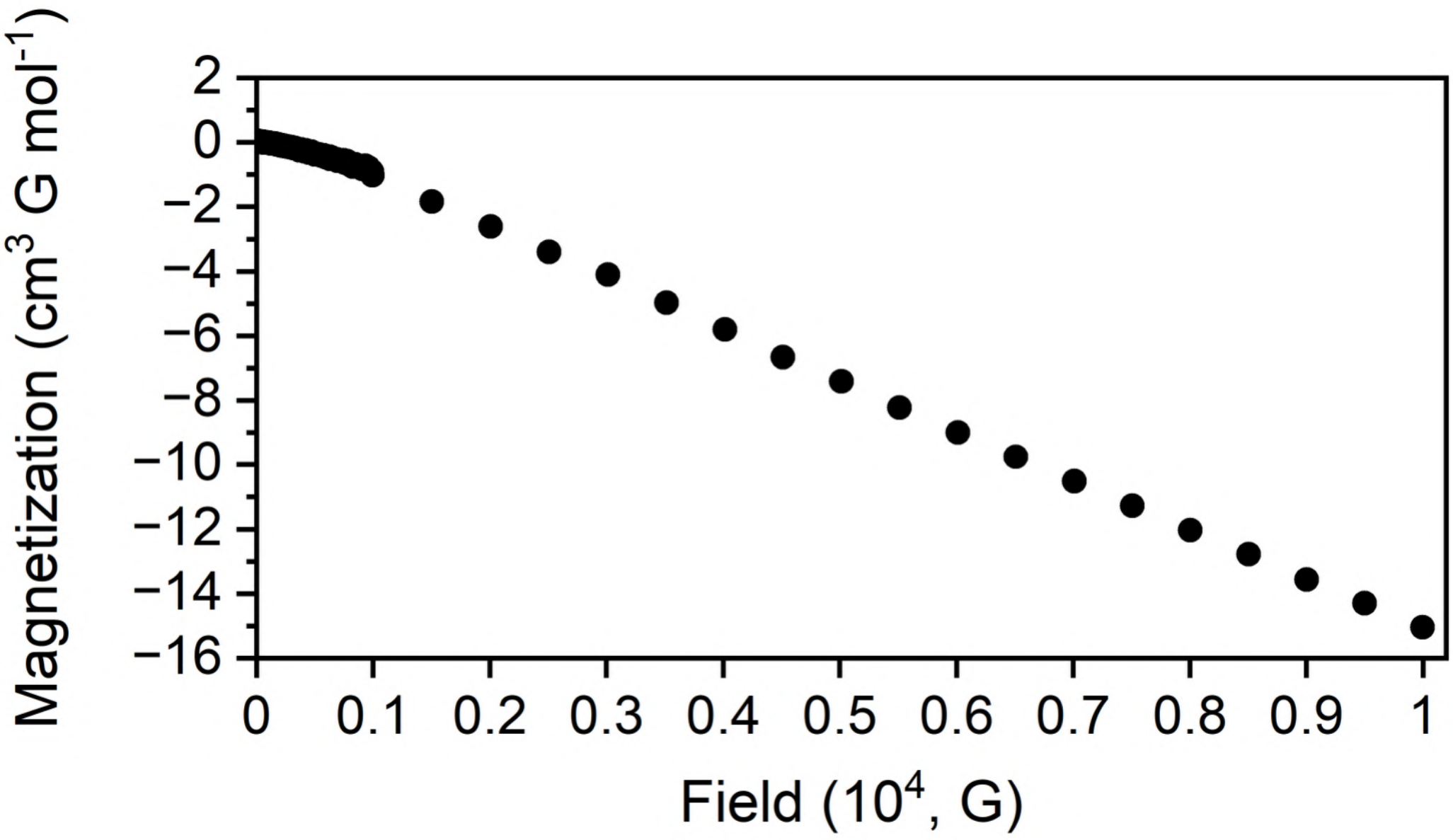

Supplementary Figure 155. Magnetization vs Field data for **5K** at 300 K from 0-1 Tesla. Error bars not displayed for clarity due to large number of data points, especially below 100 Gauss (G). Average percentage errors are as follows: 19.81% (1-53 G) and 5.54% (55-10000 G). The variation in error across the field range derives from the alteration in shape of the raw voltage waveform as the DC moment changes from positive (paramagnetic) to negative (diamagnetic) as the field increases. This change in DC moment moves the raw voltage waveform from a positive single dipole response curve at very low fields, to an asymmetric response curve at intermediate fields, and finally back to a single dipole response curve at higher fields (but now negative), therefore in this intermediate region, the mathematical fit to the raw waveform will be poorer and the error greater. Errors derive from the standard deviation of the sampling distribution of the points used (sample population divided by the square root of the number of samples) to compute the reported moment represented as a percentage.

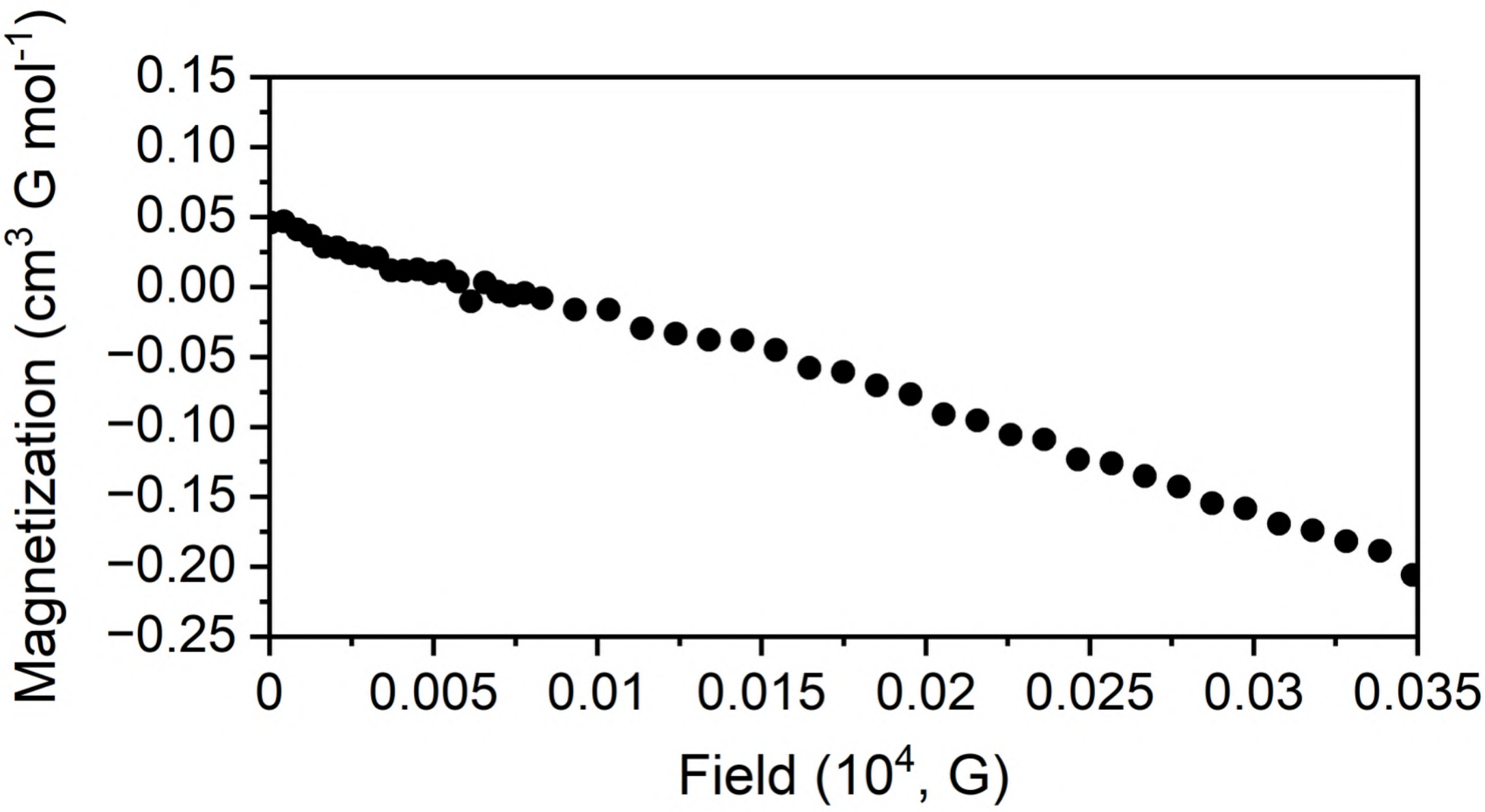

Supplementary Figure 156. Magnetization vs Field data for **5K** at 300 K from 0-0.035 Tesla. Error bars not displayed for clarity due to large number of data points.

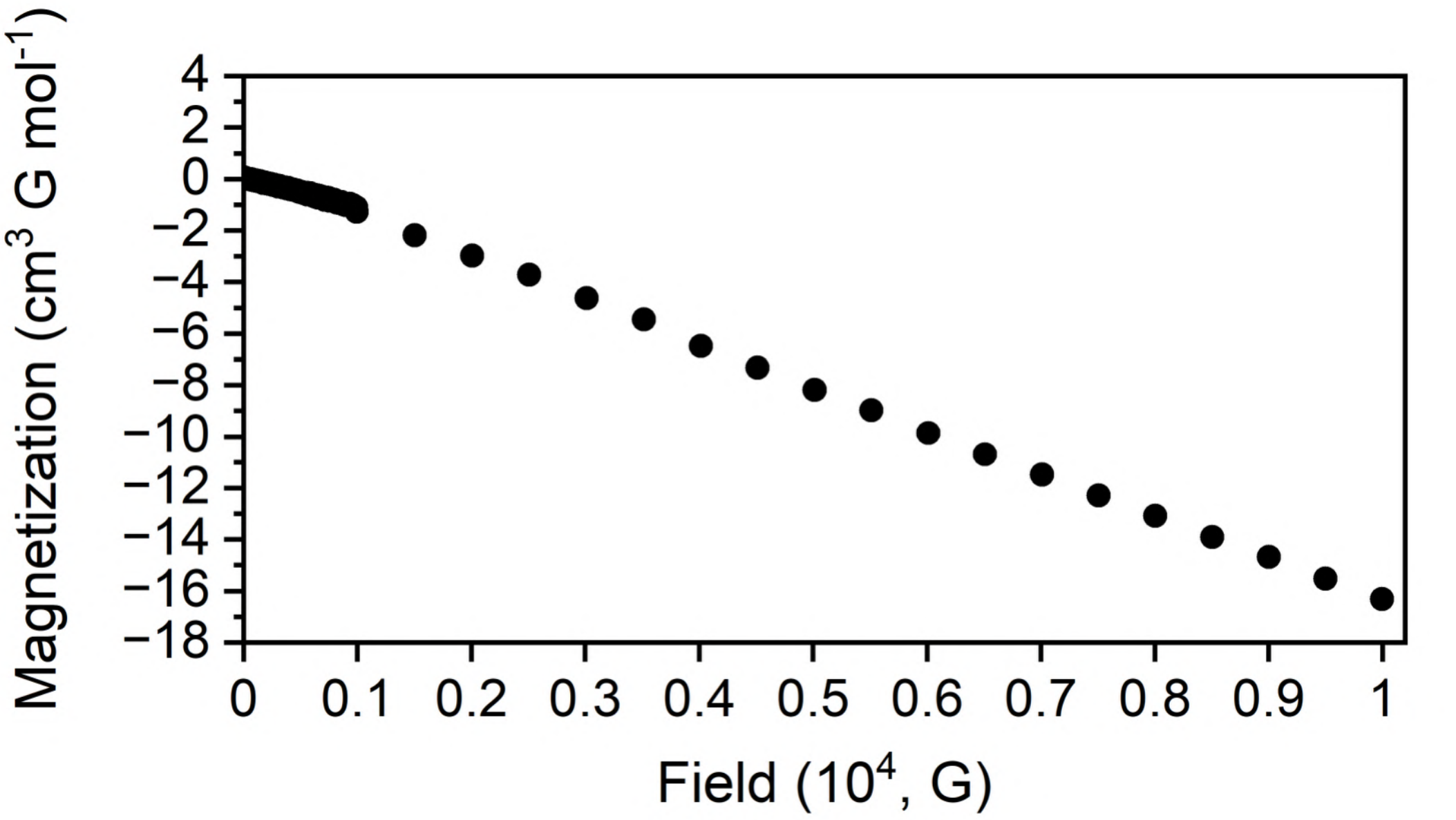

Supplementary Figure 157. Magnetization vs Field data for **5Rb** at 300 K from 0-1 Tesla. Error bars not displayed for clarity due to large number of data points, especially below 100 Gauss (G). Average percentage errors are as follows: 13.05% (1-33 G) and 5.46% (35-10000 G). The variation in error across the field range derives from the alteration in shape of the raw voltage waveform as the DC moment changes from positive (paramagnetic) to negative (diamagnetic) as the field increases. This change in DC moment moves the raw voltage waveform from a positive single dipole response curve at very low fields, to an asymmetric response curve at intermediate fields, and finally back to a single dipole response curve at higher fields (but now negative), therefore in this intermediate region, the mathematical fit to the raw waveform will be poorer and the error greater. Errors derive from the standard deviation of the sampling distribution of the points used (sample population divided by the square root of the number of samples) to compute the reported moment represented as a percentage.

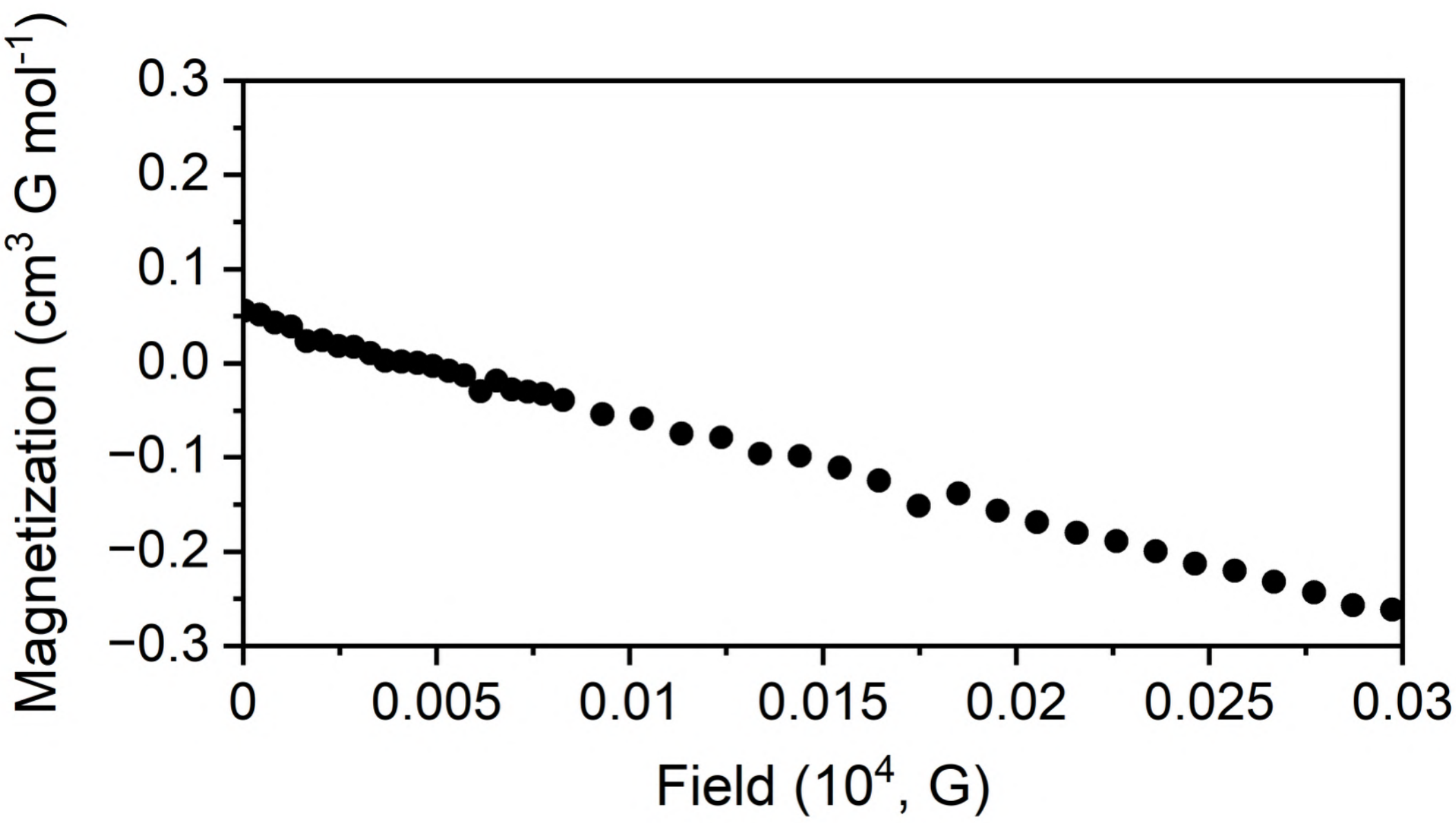

Supplementary Figure 158. Magnetization vs Field data for **5Rb** at 300 K from 0-0.03 Tesla. Error bars not displayed for clarity due to large number of data points.

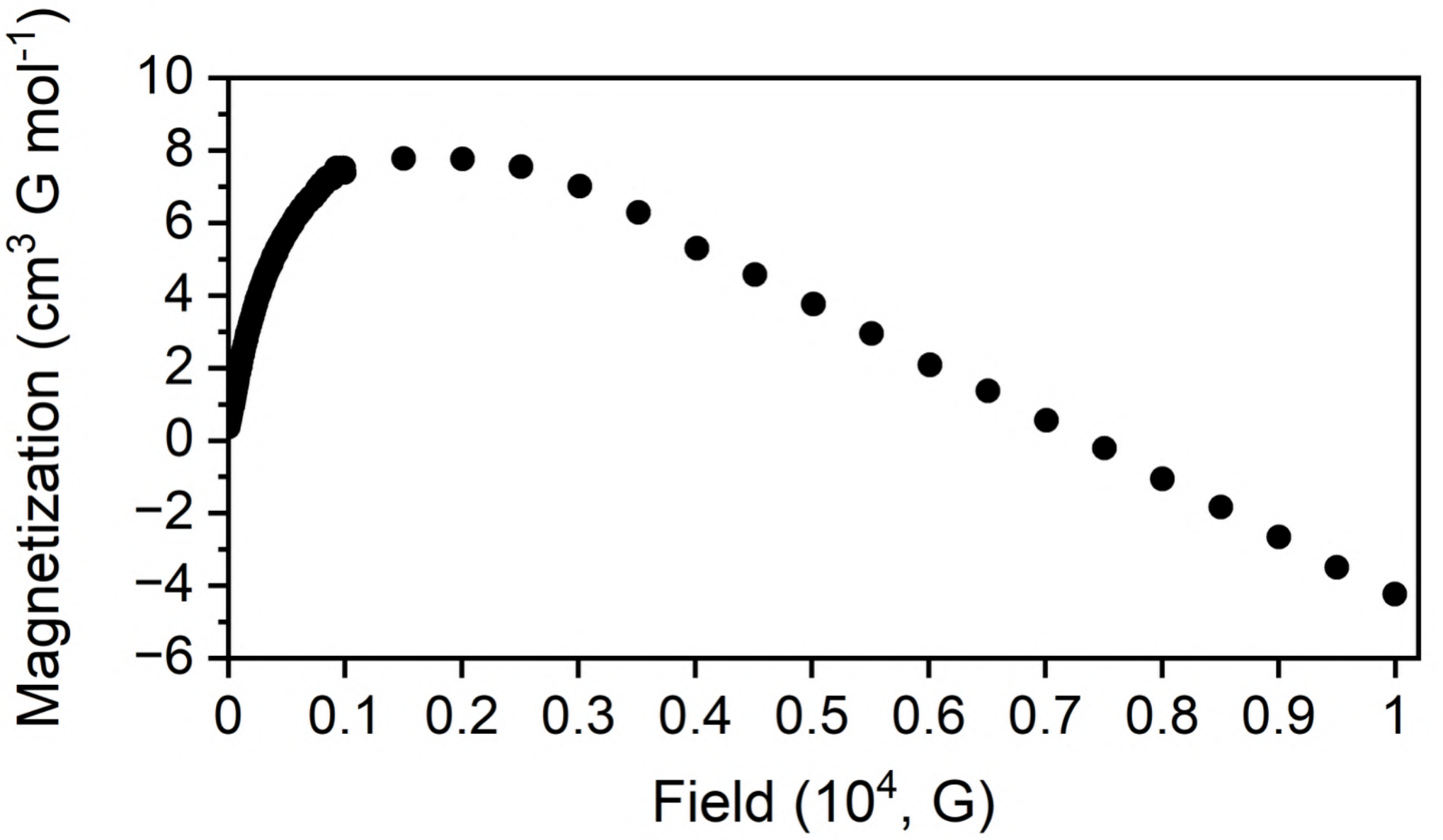

Supplementary Figure 159. Magnetization vs Field data for **5Cs** at 300 K from 0-1 Tesla. Error bars not displayed for clarity due to large number of data points, especially below 100 Gauss (G). Average percentage errors are as follows: 6.21% (2-2250 G), 18.41% (2500-3500 G), and 5.48% (3750-10000 G). The variation in error across the field range derives from the alteration in shape of the raw voltage waveform as the DC moment changes from positive (paramagnetic) to negative (diamagnetic) as the field increases. This change in DC moment moves the raw voltage waveform from a positive single dipole response curve at very low fields, to an asymmetric response curve at intermediate fields, and finally back to a single dipole response curve at higher fields (but now negative), therefore in this intermediate region, the mathematical fit to the raw waveform will be poorer and the error greater. Errors derive from the standard deviation of the sampling distribution of the points used (sample population divided by the square root of the number of samples) to compute the reported moment represented as a percentage.

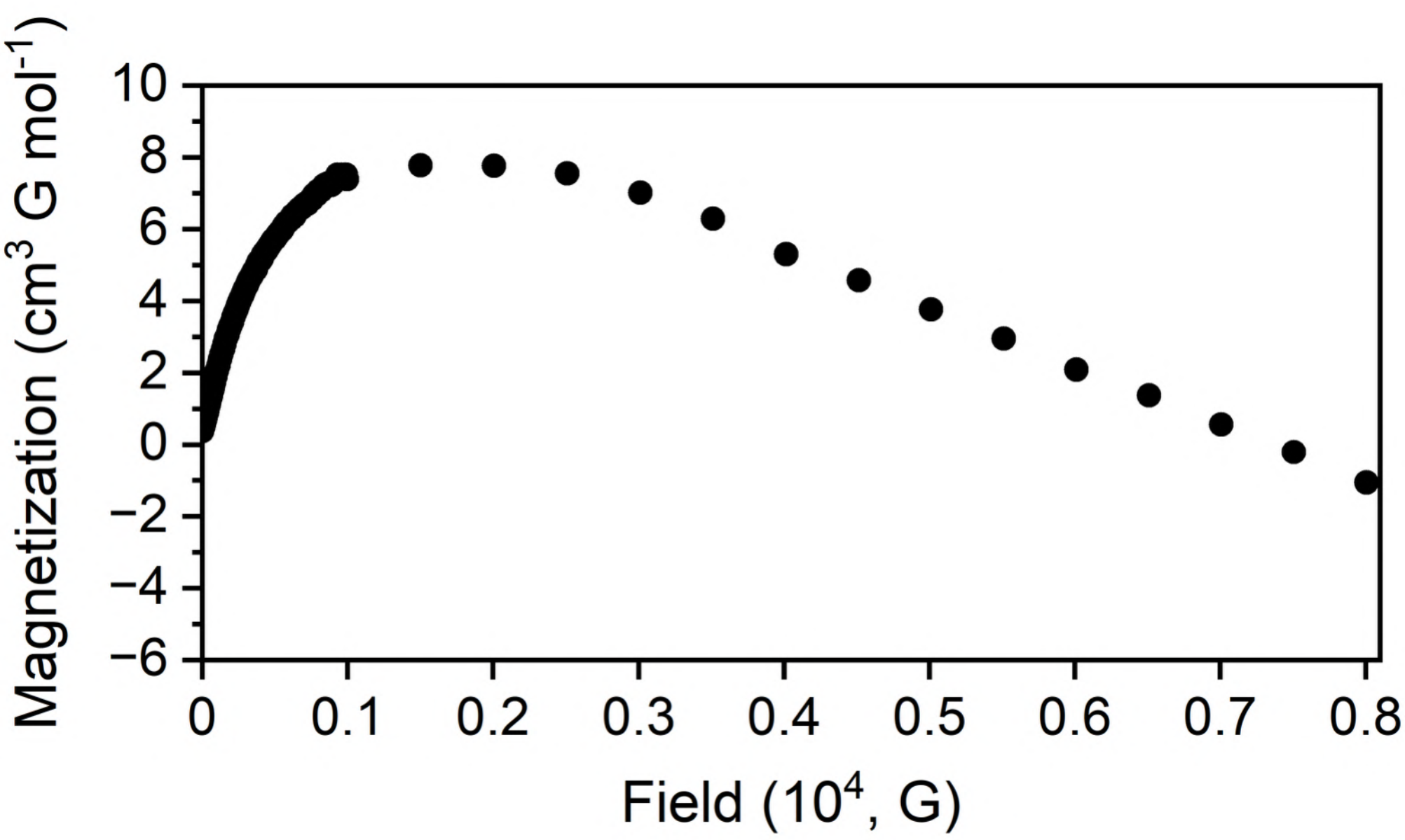

Supplementary Figure 160. Magnetization vs Field data for **5Cs** at 300 K from 0-0.8 Tesla. Error bars not displayed for clarity due to large number of data points.

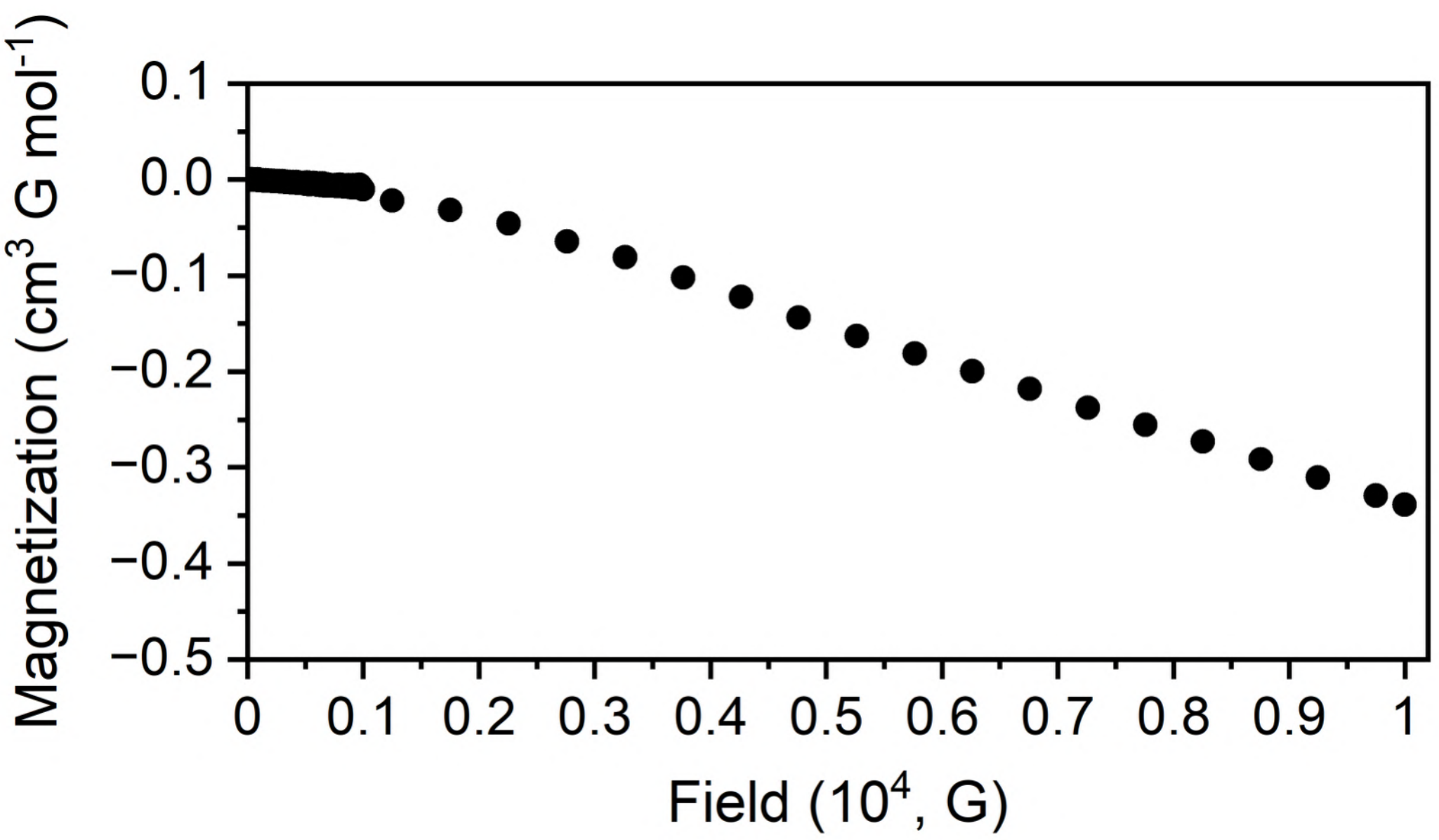

Supplementary Figure 161. Magnetization vs Field data for benzene at 300 K from 0-1 Tesla. Error bars not displayed for clarity due to large number of data points, especially below 100 Gauss (G). Average percentage errors are as follows: 19.78% (3-41 G) and 5.67% (43-10000 G). The variation in error across the field range derives from the alteration in shape of the raw voltage waveform as the DC moment changes from positive (paramagnetic) to negative (diamagnetic) as the field increases. This change in DC moment moves the raw voltage waveform from a positive single dipole response curve at very low fields, to an asymmetric response curve at intermediate fields, and finally back to a single dipole response curve at higher fields (but now negative), therefore in this intermediate region, the mathematical fit to the raw waveform will be poorer and the error greater. Errors derive from the standard deviation of the sampling distribution of the points used (sample population divided by the square root of the number of samples) to compute the reported moment represented as a percentage.

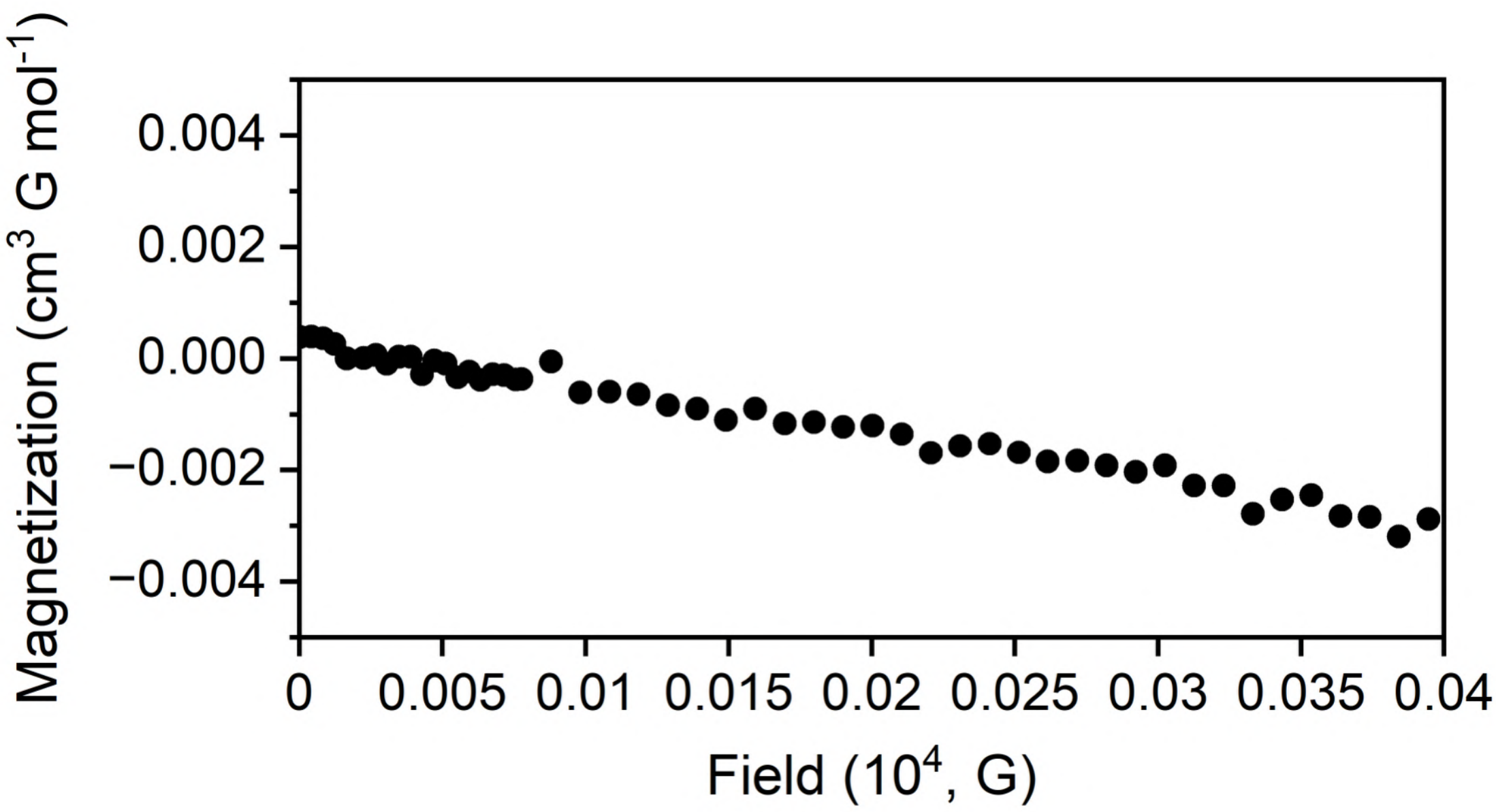

Supplementary Figure 162. Magnetization vs Field data for benzene at 300 K from 0-0.04 Tesla. Error bars not displayed for clarity due to large number of data points.

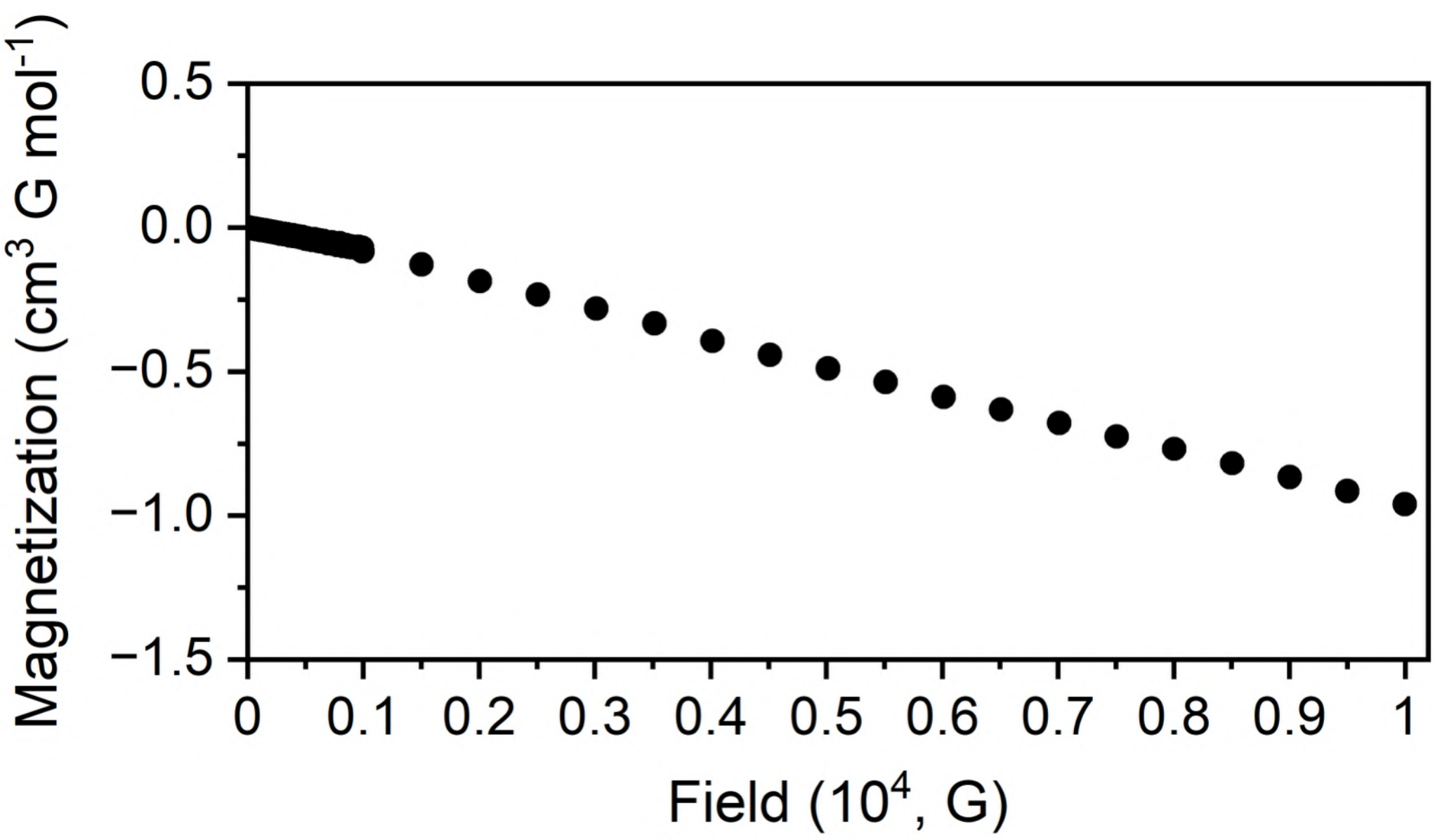

Supplementary Figure 163. Magnetization vs Field data for naphthalene at 300 K from 0-1 Tesla. Error bars not displayed for clarity due to large number of data points, especially below 100 Gauss (G). Average percentage errors are as follows: Average percentage errors are as follows: 13.82% (1-15 G) and 5.47% (17-10000 G). The variation in error across the field range derives from the alteration in shape of the raw voltage waveform as the DC moment changes from positive (paramagnetic) to negative (diamagnetic) as the field increases. This change in DC moment moves the raw voltage waveform from a positive single dipole response curve at very low fields, to an asymmetric response curve at intermediate fields, and finally back to a single dipole response curve at higher fields (but now negative), therefore in this intermediate region, the mathematical fit to the raw waveform will be poorer and the error greater. Errors derive from the standard deviation of the sampling distribution of the points used (sample population divided by the square root of the number of samples) to compute the reported moment represented as a percentage.

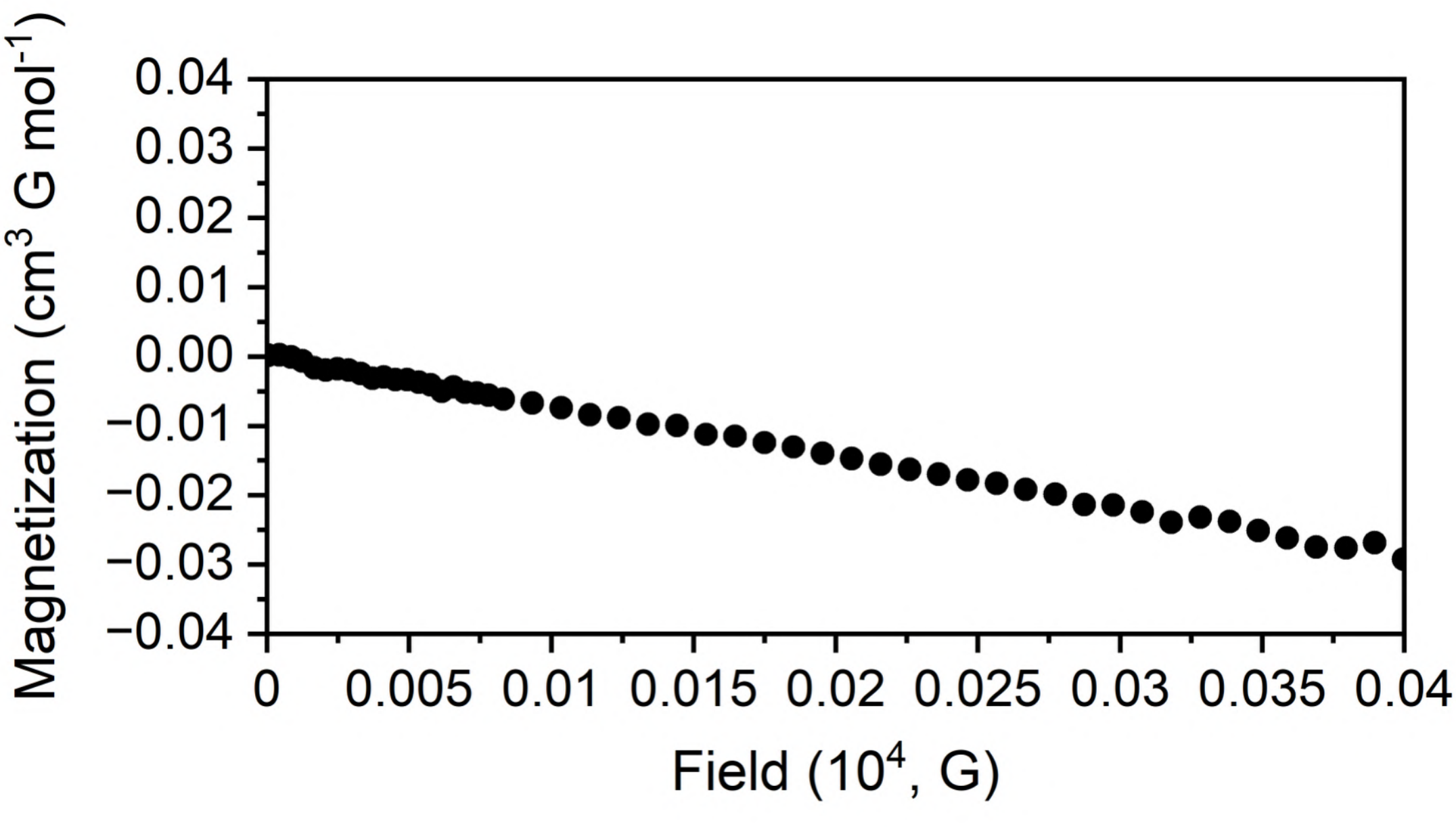

Supplementary Figure 164. Magnetization vs Field data for naphthalene at 300 K from 0-0.04 Tesla. Error bars not displayed for clarity due to large number of data points.

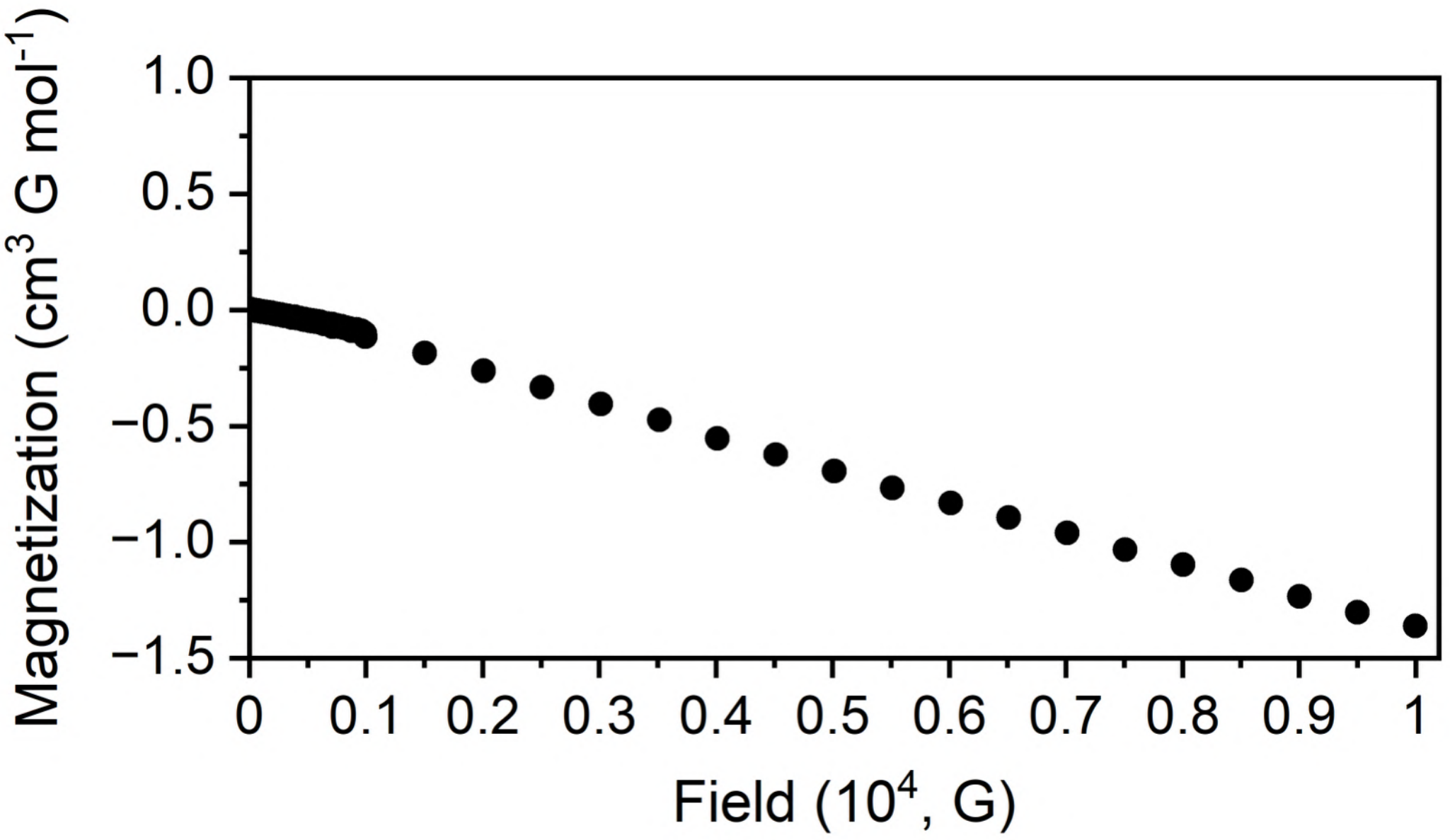

Supplementary Figure 165. Magnetization vs Field data for anthracene at 300 K from 0-1 Tesla. Error bars not displayed for clarity due to large number of data points, especially below 100 Gauss (G). Average percentage errors are as follows: 28.09% (1-49 G) and 5.92% (51-10000 G). The variation in error across the field range derives from the alteration in shape of the raw voltage waveform as the DC moment changes from positive (paramagnetic) to negative (diamagnetic) as the field increases. This change in DC moment moves the raw voltage waveform from a positive single dipole response curve at very low fields, to an asymmetric response curve at intermediate fields, and finally back to a single dipole response curve at higher fields (but now negative), therefore in this intermediate region, the mathematical fit to the raw waveform will be poorer and the error greater. Errors derive from the standard deviation of the sampling distribution of the points used (sample population divided by the square root of the number of samples) to compute the reported moment represented as a percentage.

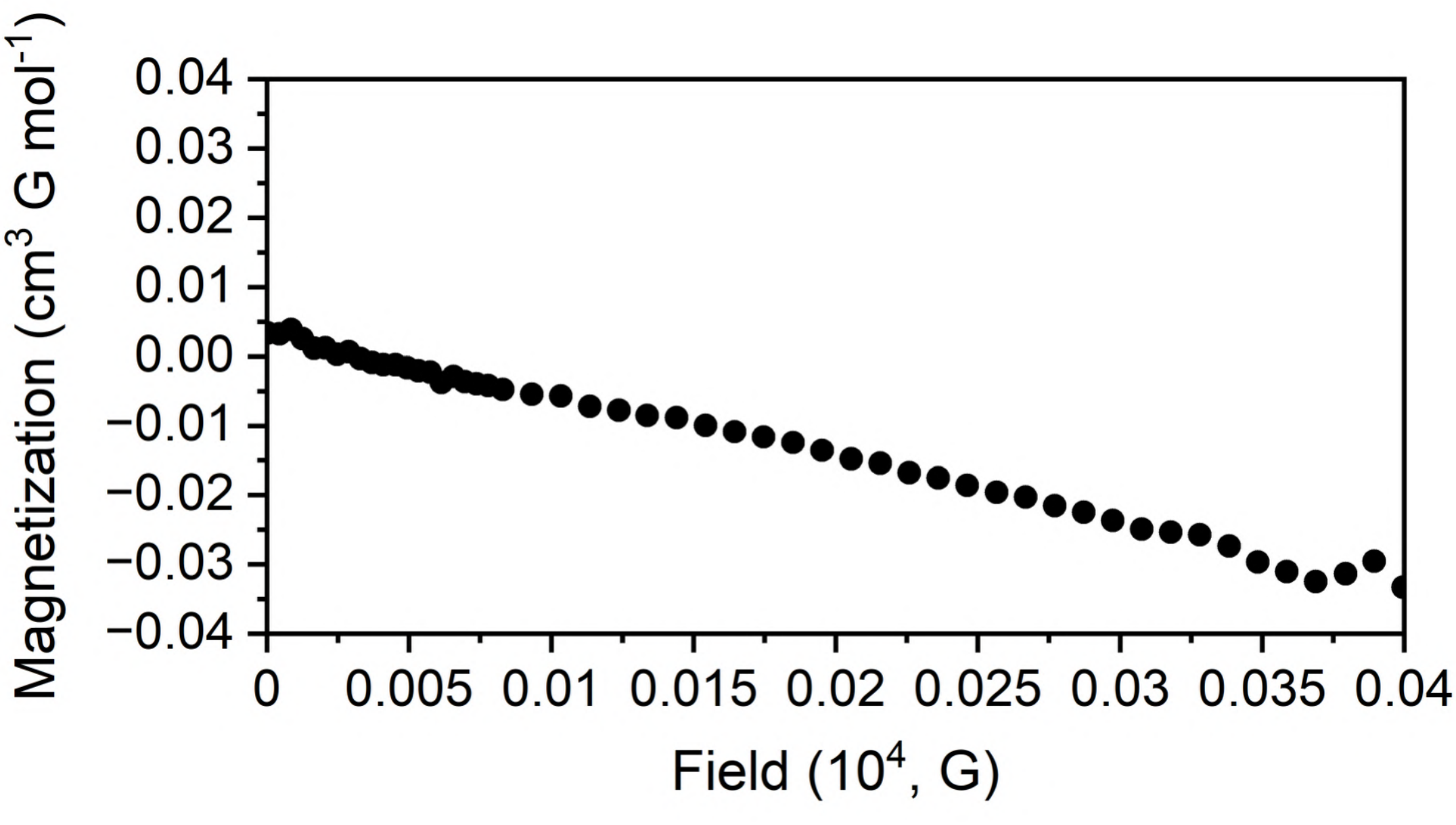

Supplementary Figure 166. Magnetization vs Field data for anthracene at 300 K from 0-0.04 Tesla. Error bars not displayed for clarity due to large number of data points.

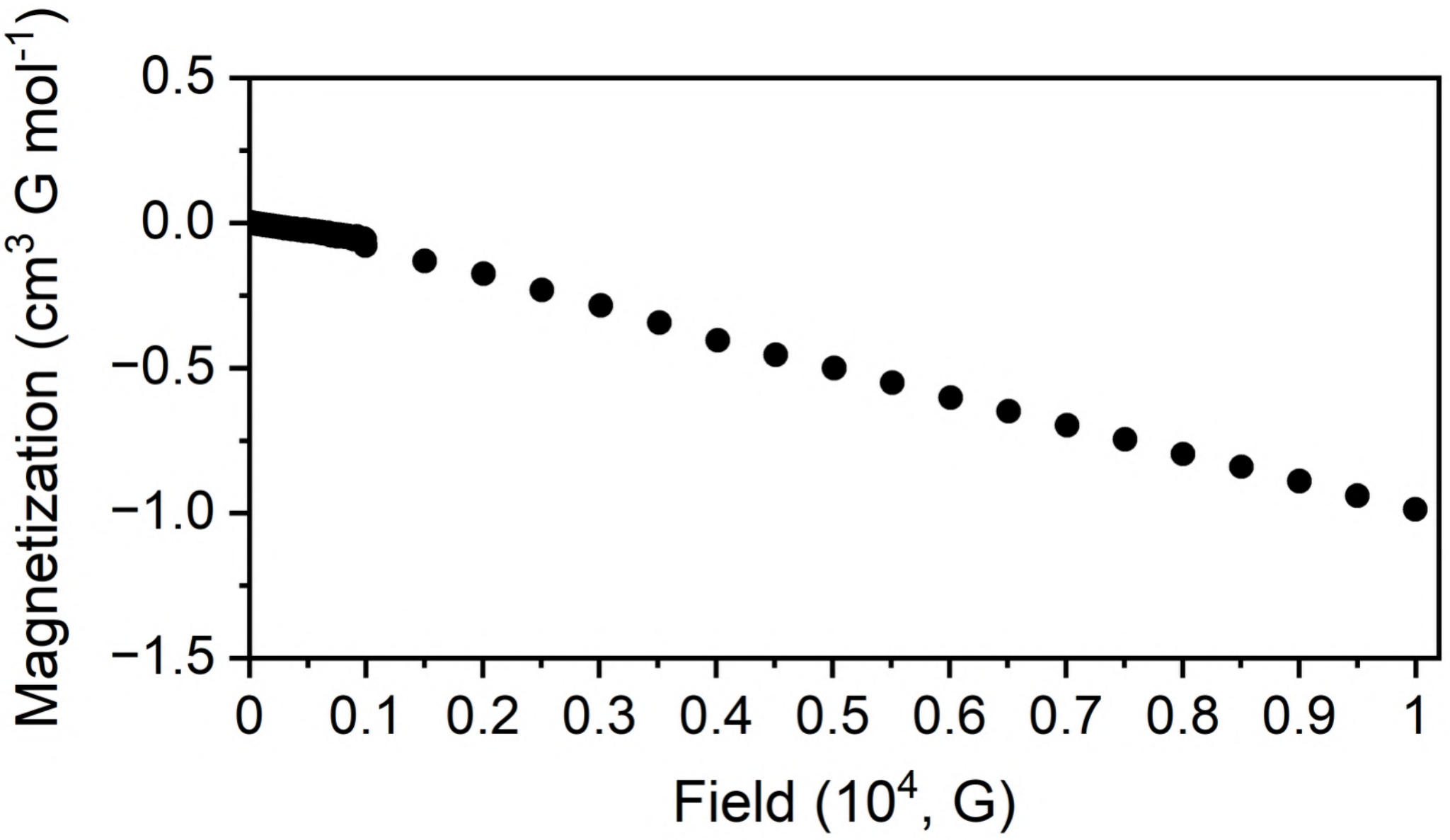

Supplementary Figure 167. Magnetization vs Field data for biphenyl at 300 K from 0-1 Tesla. Error bars not displayed for clarity due to large number of data points, especially below 100 Gauss (G). Average percentage errors are as follows: Average percentage errors are as follows: 19.47% (1-39 G) and 6.32% (41-10000 G). The variation in error across the field range derives from the alteration in shape of the raw voltage waveform as the DC moment changes from positive (paramagnetic) to negative (diamagnetic) as the field increases. This change in DC moment moves the raw voltage waveform from a positive single dipole response curve at very low fields, to an asymmetric response curve at intermediate fields, and finally back to a single dipole response curve at higher fields (but now negative), therefore in this intermediate region, the mathematical fit to the raw waveform will be poorer and the error greater. Errors derive from the standard deviation of the sampling distribution of the points used (sample population divided by the square root of the number of samples) to compute the reported moment represented as a percentage.

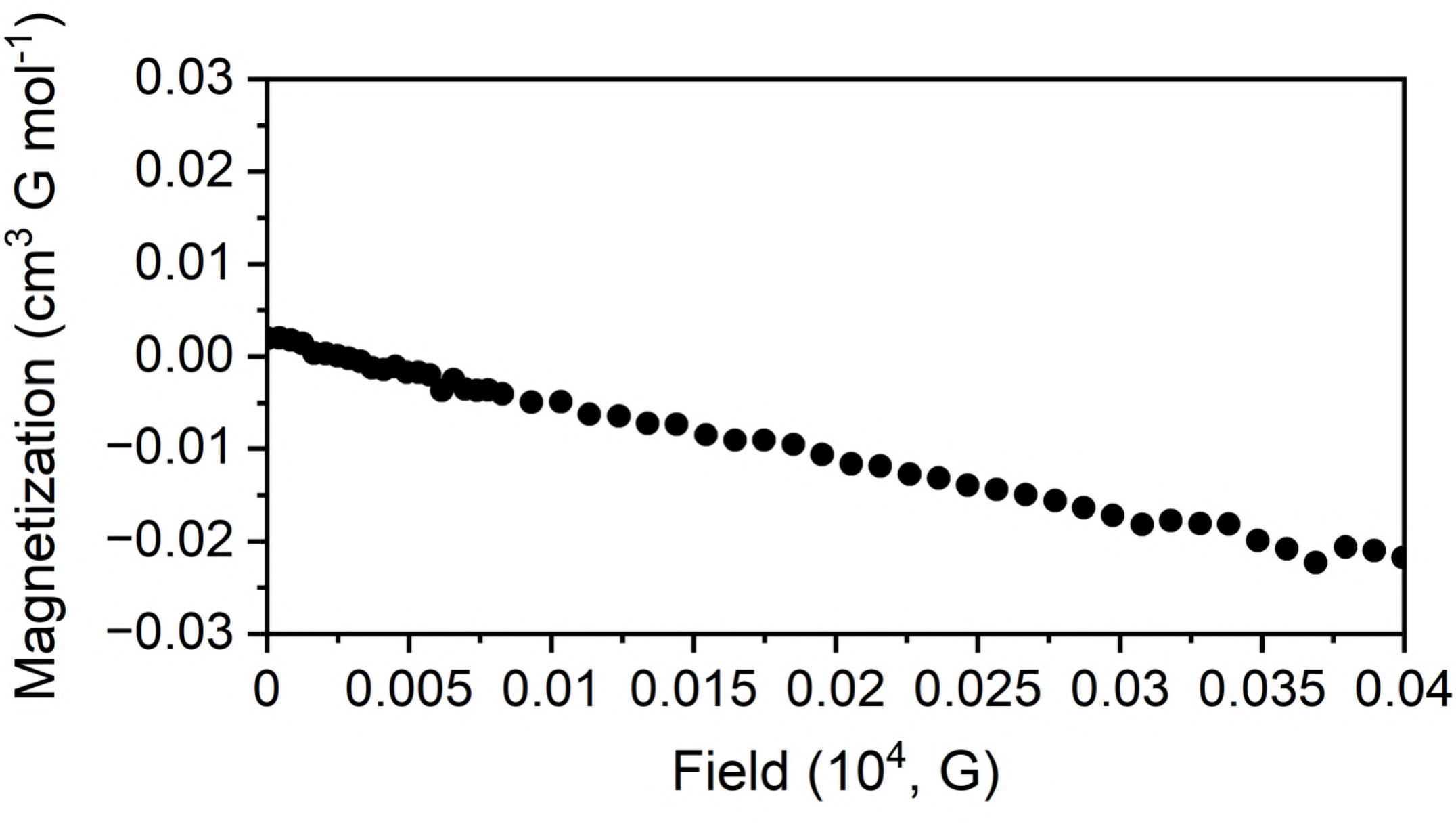

Supplementary Figure 168. Magnetization vs Field data for biphenyl at 300 K from 0-0.04 Tesla. Error bars not displayed for clarity due to large number of data points.

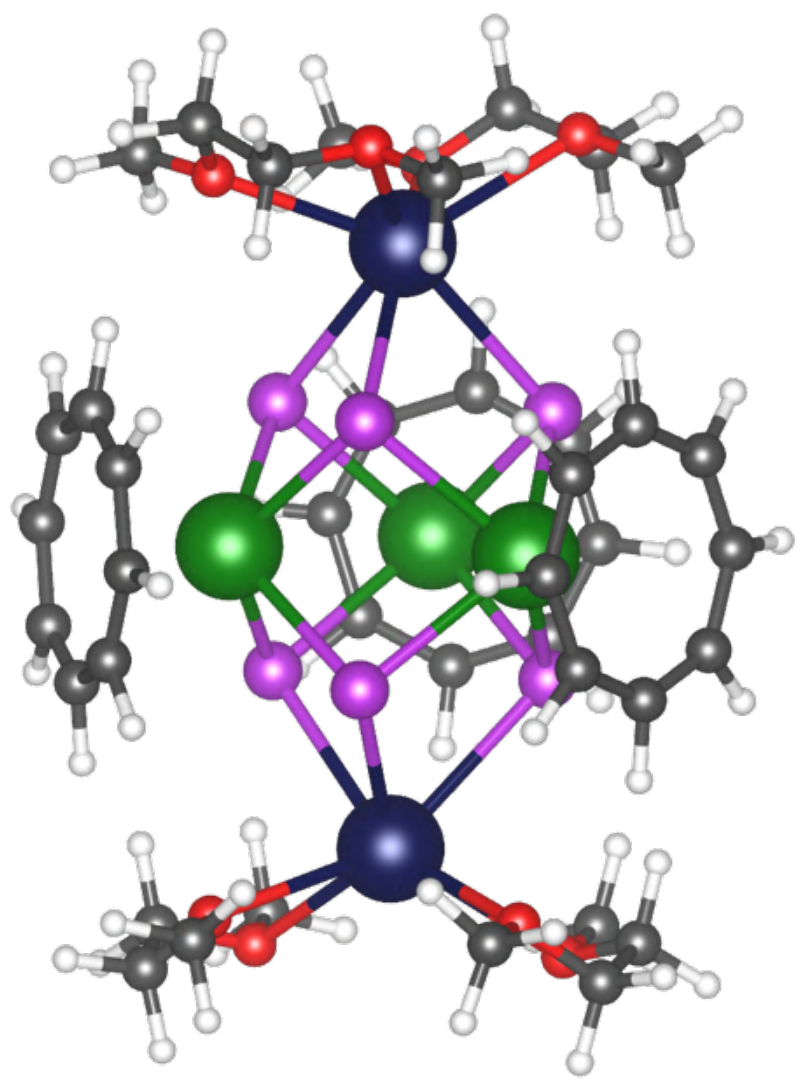

Supplementary Figure 169. The optimized geometry of **5K**. Atom colour key: thorium, green; chloride, violet; potassium, blue; oxygen, red; carbon, grey.

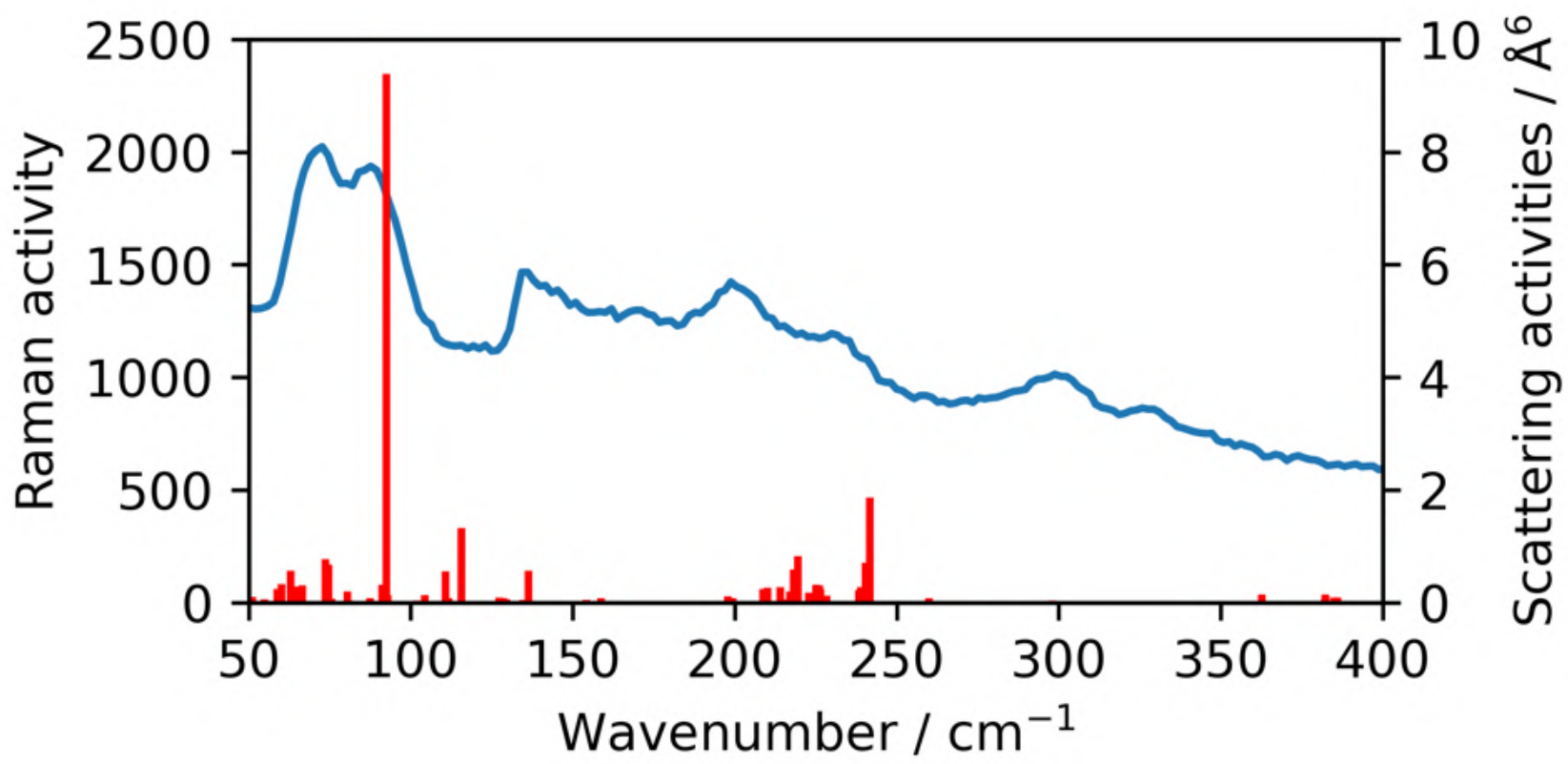

Supplementary Figure 170. Selected portion of the Raman spectrum (blue line) of **5K** with DFT computed inelastic scattering bands (red sticks).

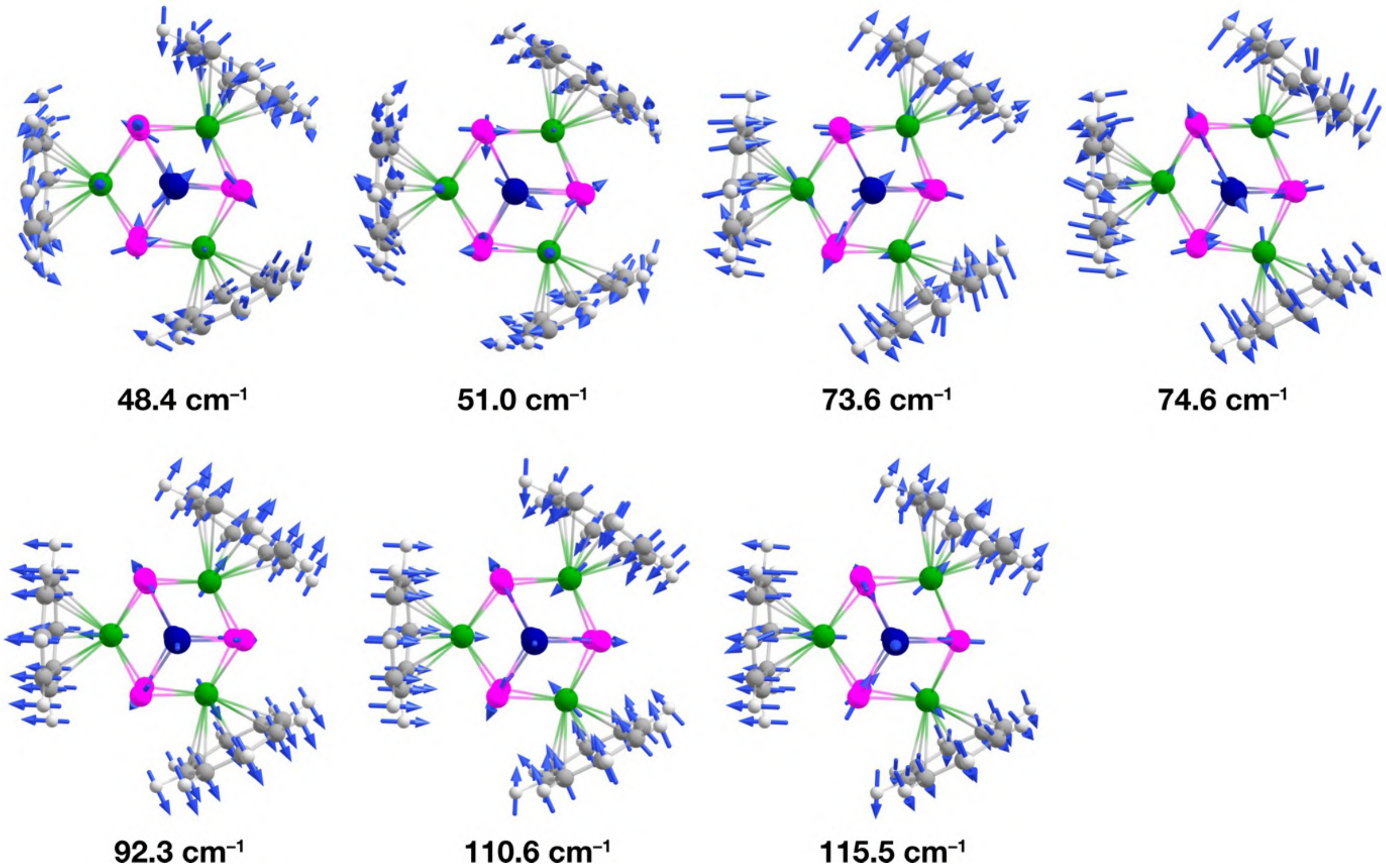

Supplementary Figure 171. Selected Raman modes of **5K** including Th_3 vibrations. The DME ligands are omitted for clarity.

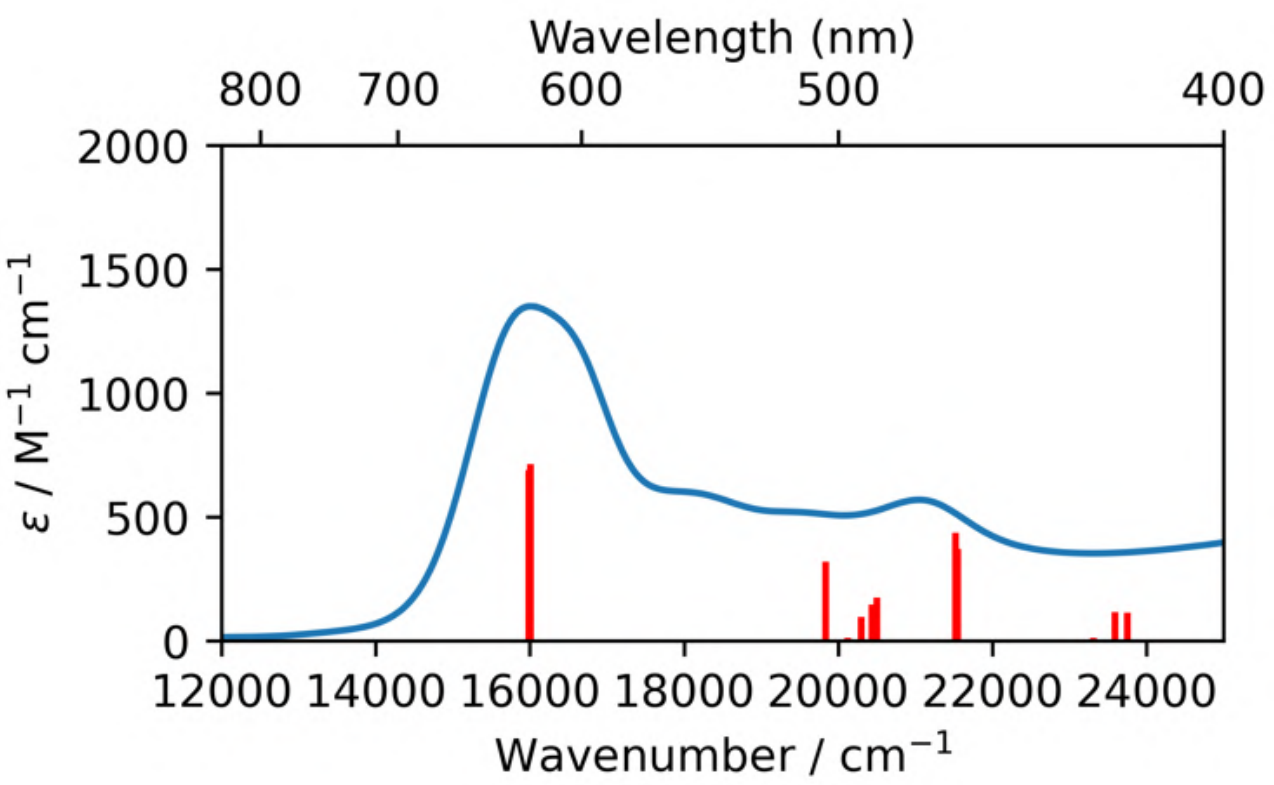

Supplementary Figure 172. The experimental UV-vis-NIR spectrum of **5K** (blue line) and calculated electronic transition energies (red sticks) calculated with the ω-B97X functional.

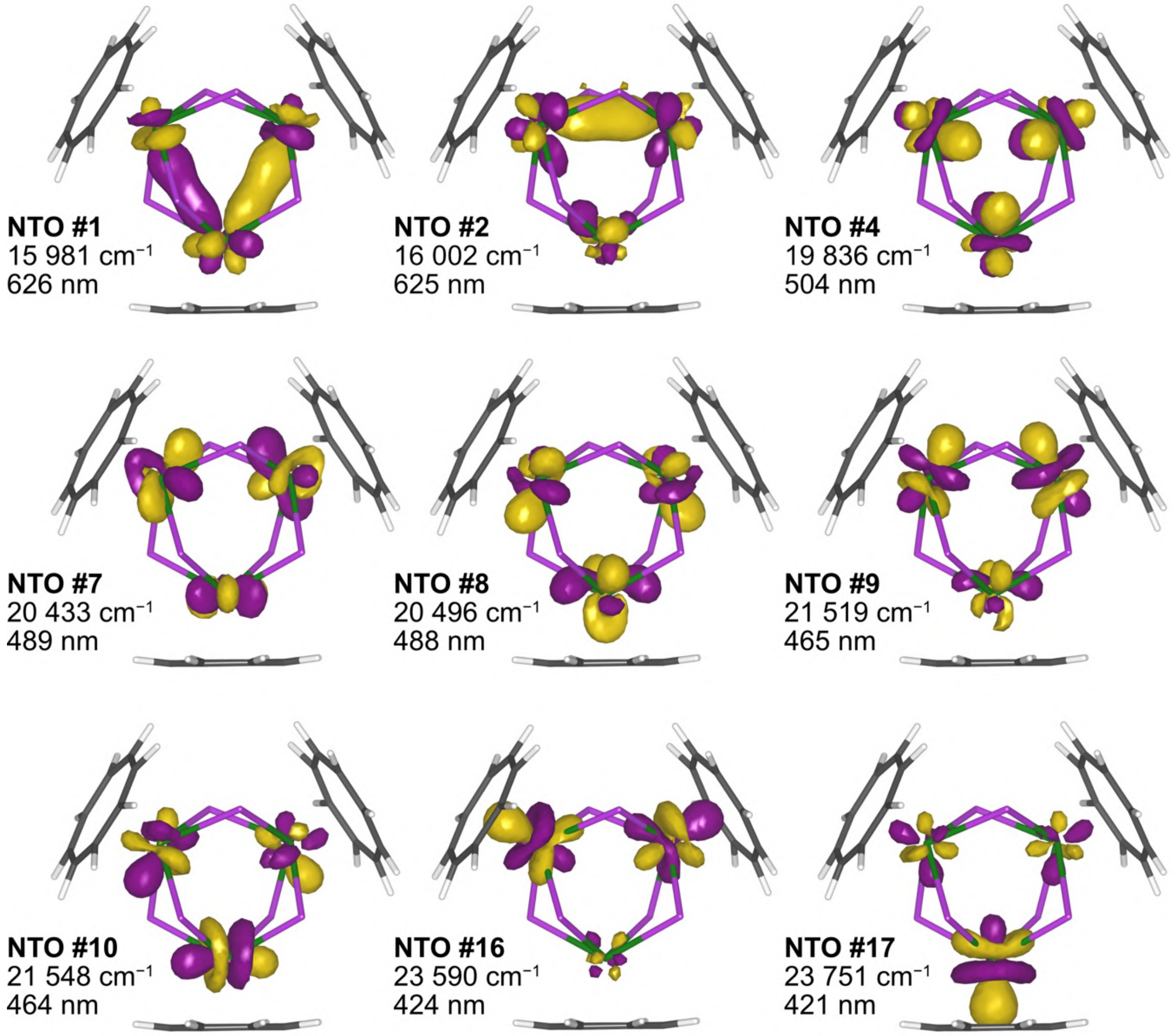

Supplementary Figure 173. Selected acceptor natural transition orbitals (NTOs) of **5K** obtained with the ω-B97X functional, plotted at an isovalue of 0.05 a.u. The $\{K(DME)_2\}^{1+}$ components are omitted for clarity.

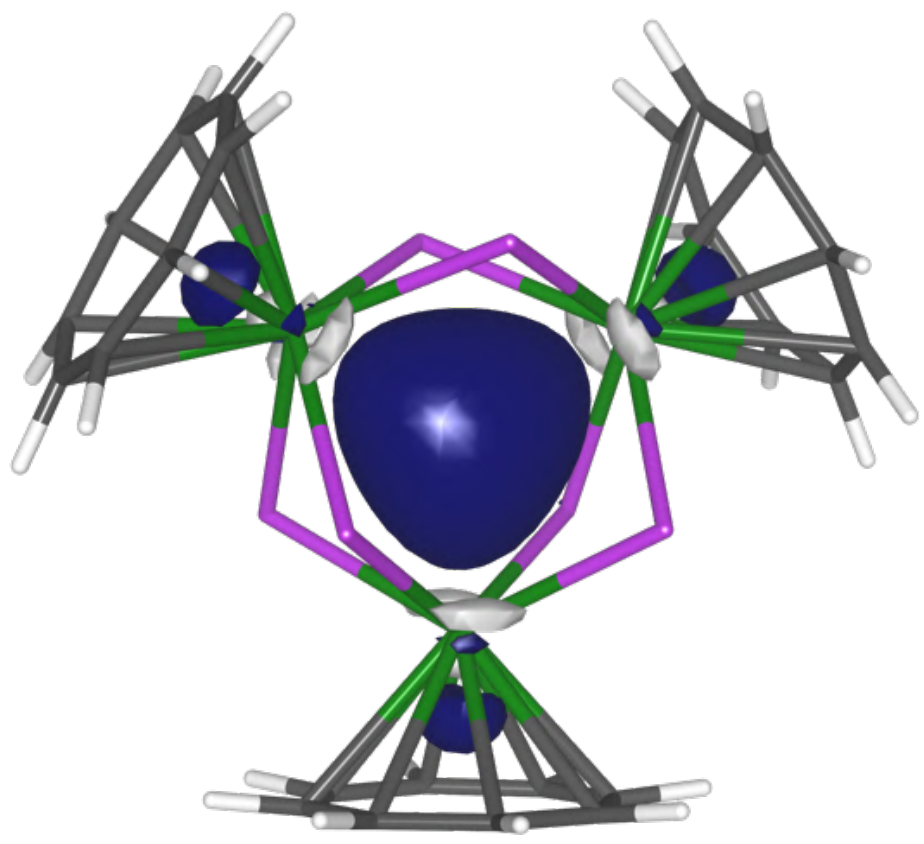

Supplementary Figure 174. HOMO (389, –2.93 ev) of **5K** plotted with an isovalue of 0.05 a.u. with the $\{K(DME)_2\}^{1+}$ components omitted for clarity.

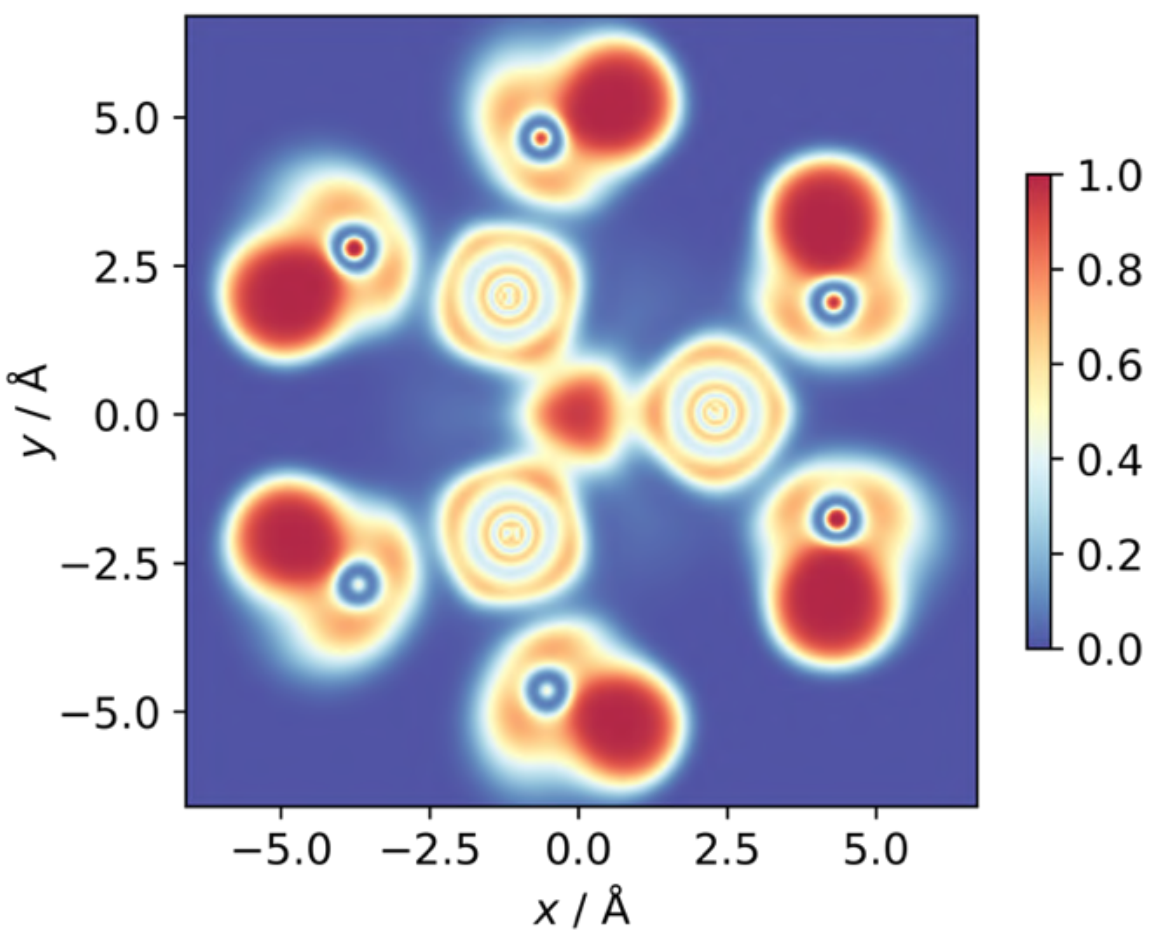

Supplementary Figure 175. ELF plot in the *xy*-plane ($z = 0$) of **5K**.

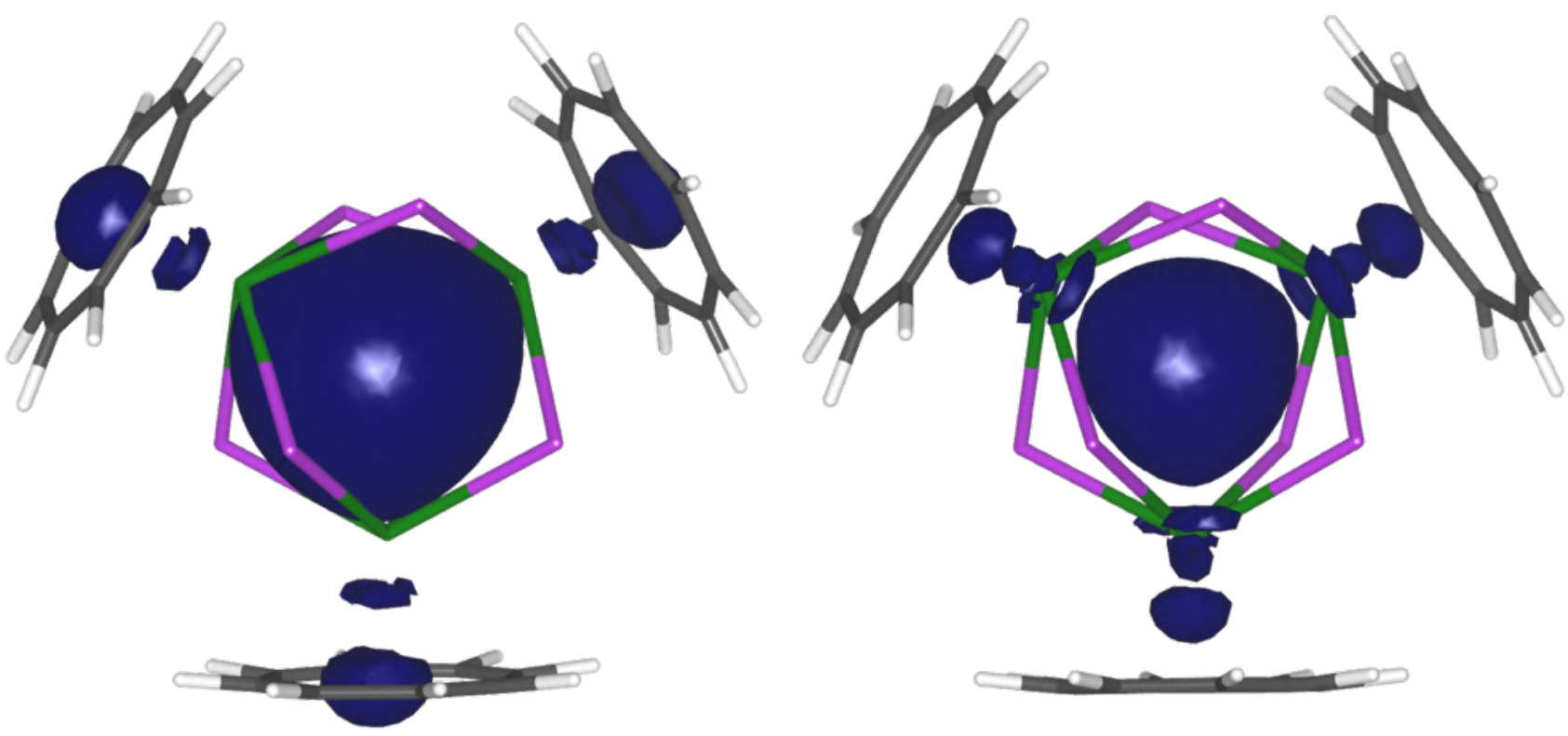

Supplementary Figure 176. Left: The 3-centre Th_3-fragment MO from AdNDP (ON = 1.77) of **5K** plotted at an isovalue of 0.05 a.u. Right: $EDDB_F$ analysis of the Th_3-fragment of **5K** plotted at an isovalue of 0.003 a.u. The $\{K(DME)_2\}^{1+}$ components are omitted for clarity.

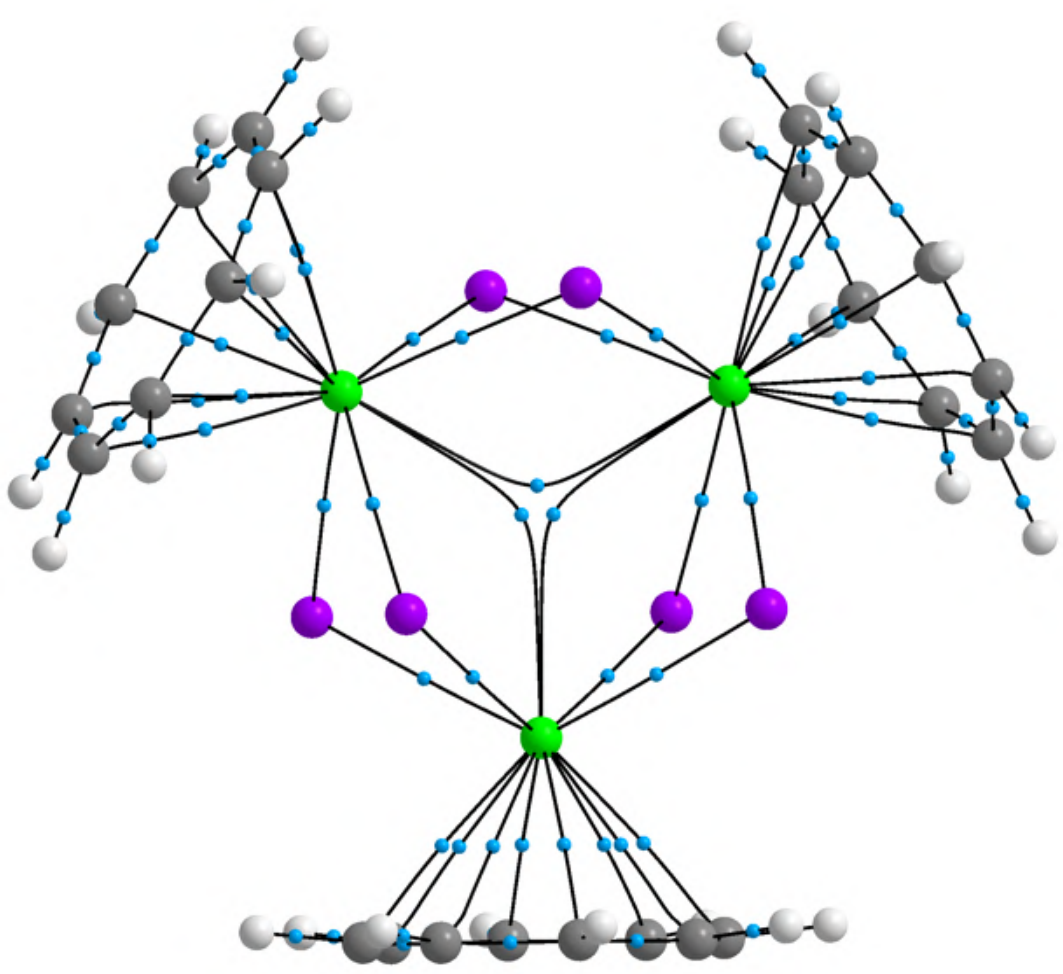

Supplementary Figure 177. QTAIM molecular graph of the bond paths in the core of **5K**. Bond critical points are shown as light blue spheres. The $\{K(DME)_2\}^{1+}$ components are omitted for clarity.

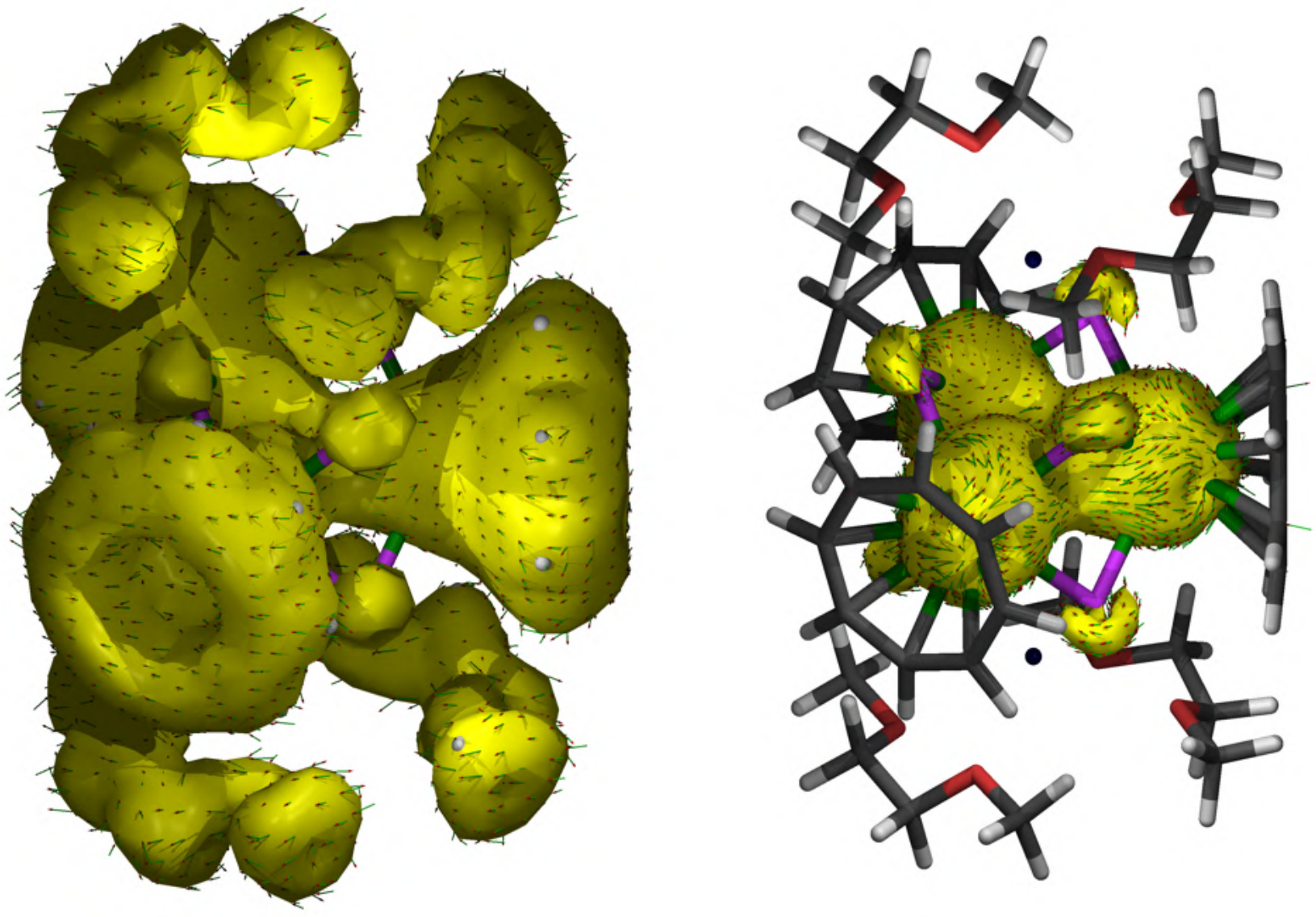

Supplementary Figure 178. Anisotropy of induced current density (AICD) of **5K** plotted at an isovalue of 0.015 a.u. and shown from the side. The magnetic field is pointing down, *i.e.*, along the K···K axis (the K atoms are the dark blue spheres in the middle), perpendicular to the Th_3 plane.

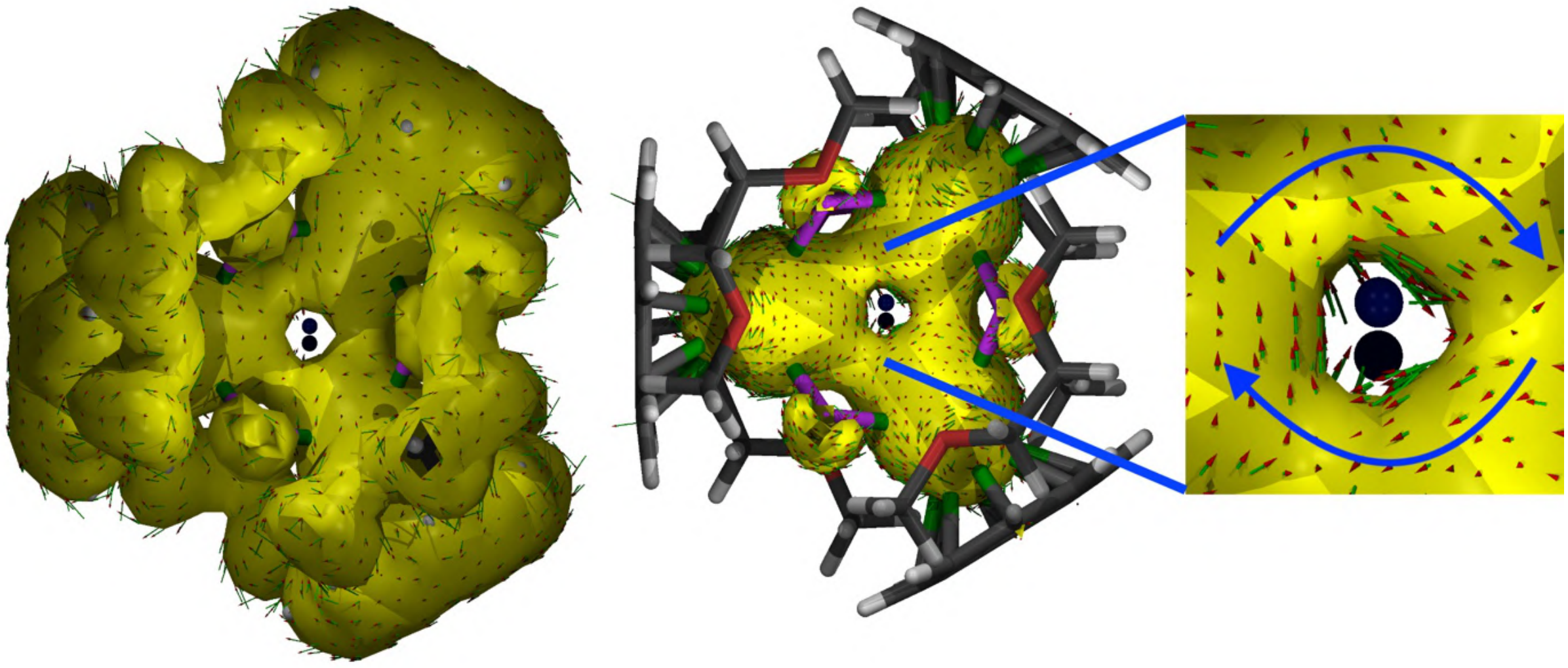

Supplementary Figure 179. Anisotropy of induced current density (AICD) of **5K** (left, full system; right its HOMO) plotted at an isovalue of 0.015 a.u. and shown from the top. The magnetic field is pointing down, *i.e.*, along the K···K axis (the K atoms are the dark blue spheres in the middle), perpendicular to the Th_3 plane.

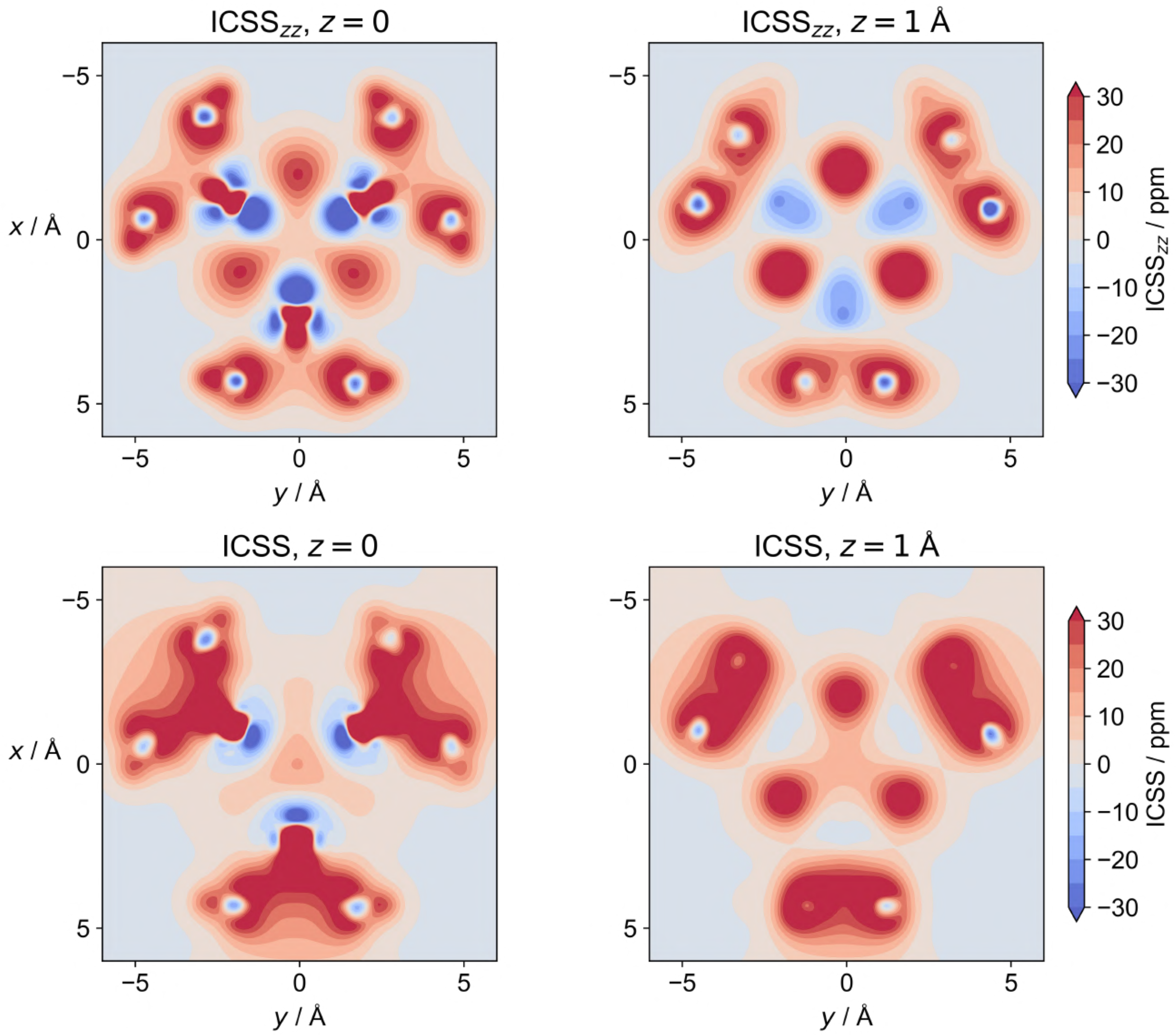

Supplementary Figure 180. The ICSS$_{zz}$ (upper) and ICSS (lower) of for **5K** in the *xy*-plane containing the Th_3 core (left) and 1 Å above the *xy*-plane (right). The red region corresponds to areas of shielding and the blue deshielding.

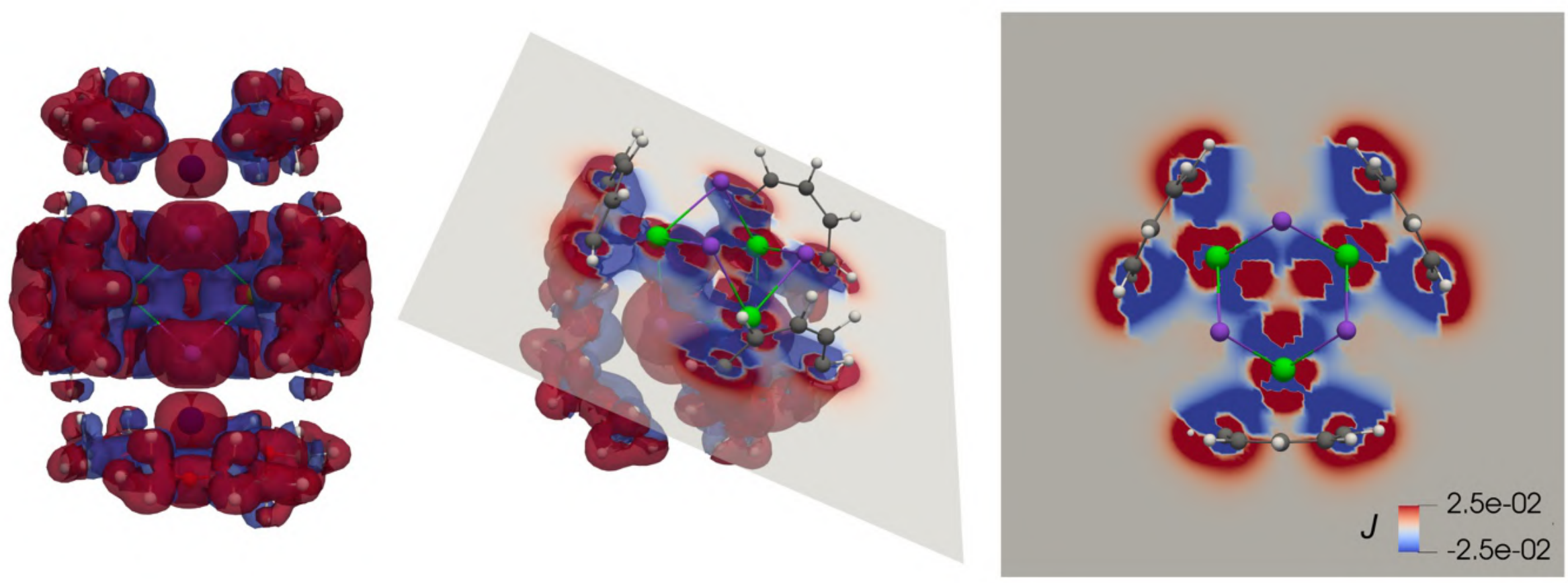

Supplementary Figure 181. Modulus of the current induced densities of **5K** plotted at isovalue of 0.025 a.u. showing diatropic (red) and paratropic (blue) components shown from the side (left) and tiled showing only the lower part of isosurface together with the cut plane passing through the Th_3 triangle (middle); Right: *xz* plane (z = 0) of the modulus of the current induced density of **5K**.

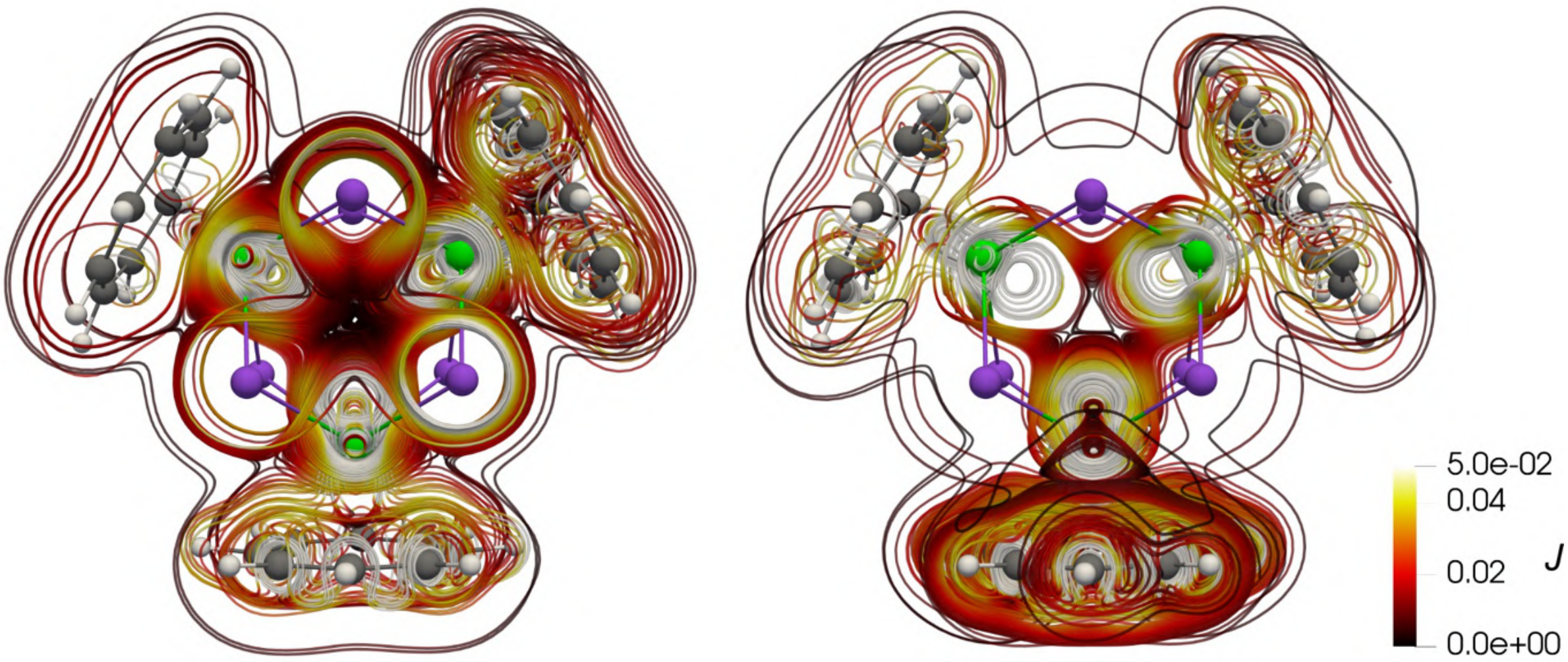

Supplementary Figure 182. Induced current densities in **5K.** Left: the currents are originating from the centre of Th_3 core. Right: the currents are originating from the Th atom on the bottom of figure.

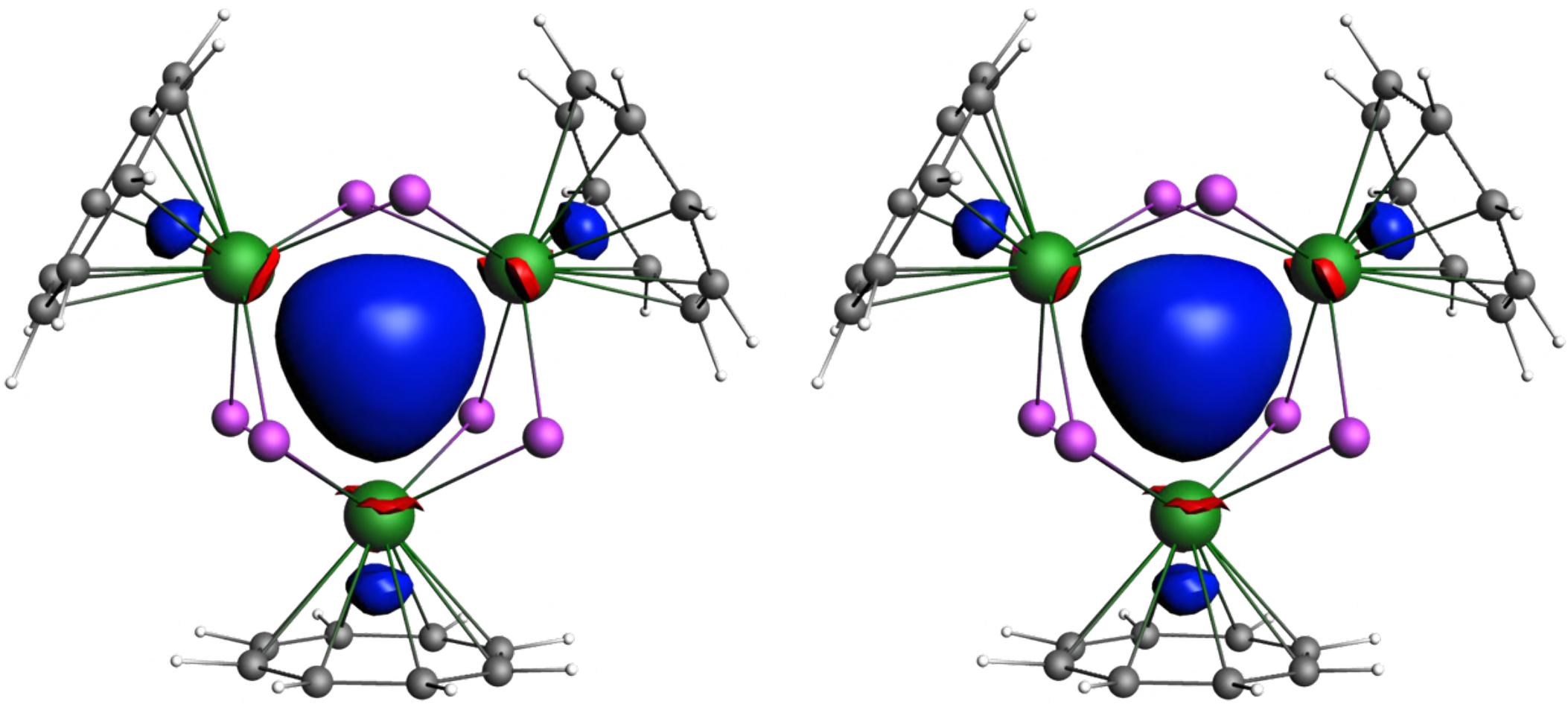

Supplementary Figure 183. HOMO of **5K** (the $\{K(DME)_2\}^+$ components omitted for clarity) plotted at an isovalue of 0.05 a.u., illustrating the qualitatively analogous nature of metal–metal bonding obtained when using scalar ZORA (left) and spin-orbit coupled (SOC) ZORA (right) Hamiltonians.

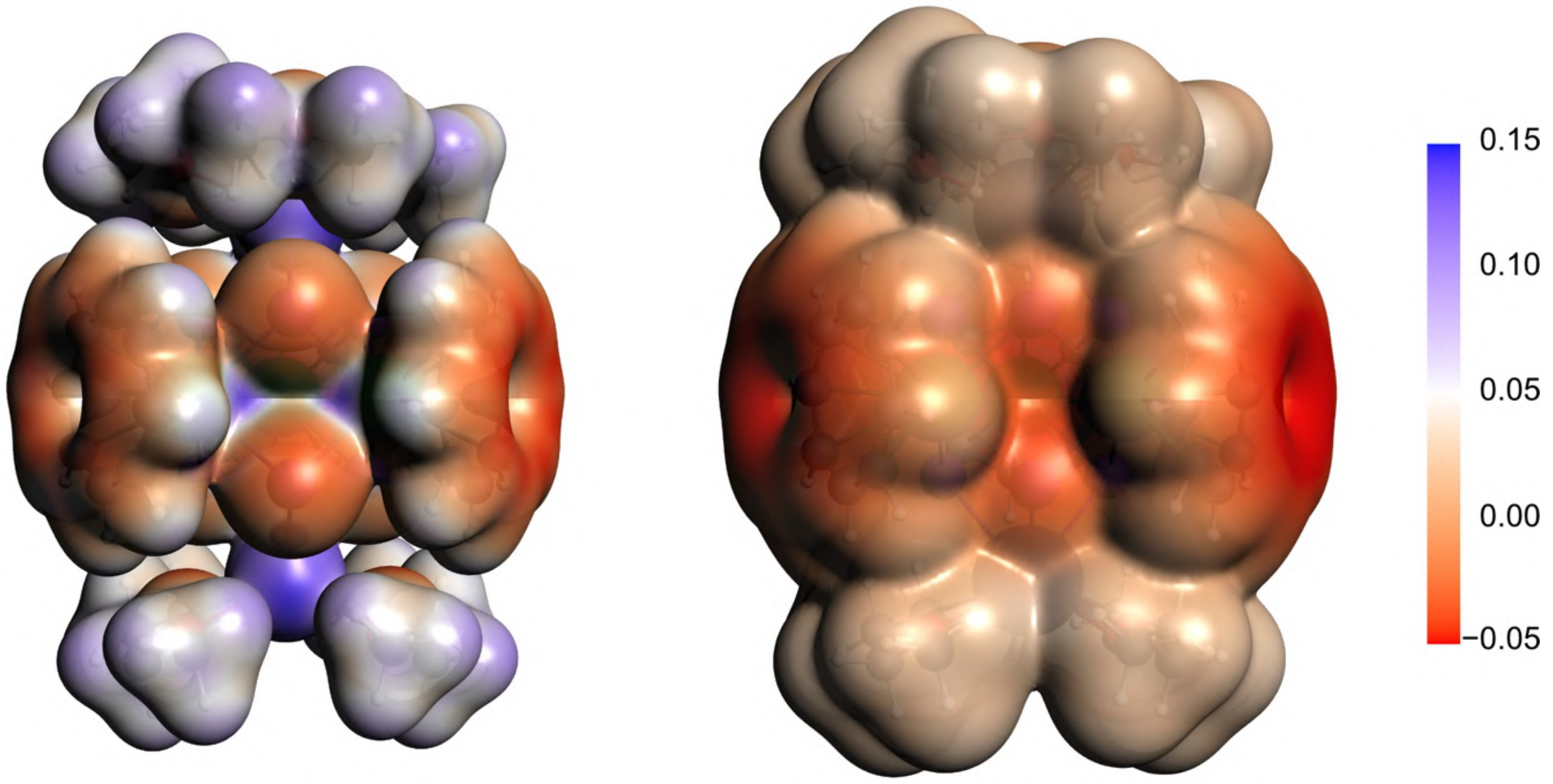

Supplementary Figure 184. Electrostatic potential mapped onto the electron-density isosurface ρ = 0.01 a.u. (left) and ρ = 0.001 a.u. (right) of **5K**.

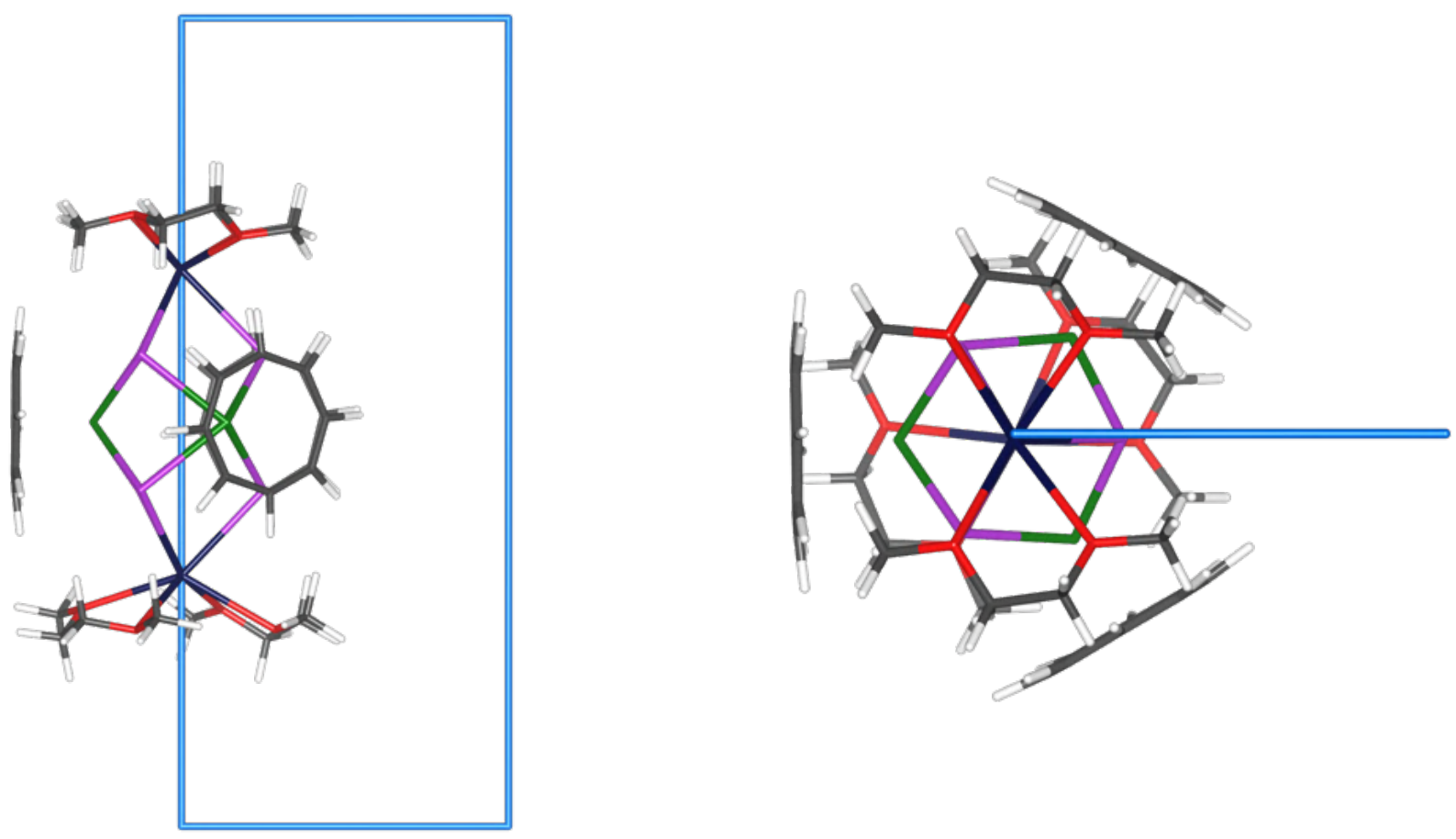

Supplementary Figure 185. The integration plane for ring current strength calculations of **5K** shown as a light blue outline from the side (left) and from the top (right).

Supplementary Tables

Supplementary Table 1. Crystallographic details for 2, 3 and 4K.

	**2**	**3**	**4K**
Formula	$C_{34}H_{72}Na_2O_{4.5}Si_4$	$C_{24}H_{52}Cs_2O_2Si_4$	$C_{40}H_{64}Cl_6KO_8Th_3$
Fw, g mol^{-1}	711.25	750.83	1620.83
Cryst size, mm	0.31 x 0.29 x 0.16	0.211 x 0.198 x 0.132	0.18 × 0.13 × 0.11
Crystal system	tetragonal	triclinic	Triclinic
Space group	*I*-4c2	*P*-1	*P-1*
Collection Temperature (K)	100(2)	100(2)	100(2)
a, (Å)	17.3929(2)	11.3970(11)	12.1079(2)
b, (Å)	17.3929(2)	14.0666(4)	13.7978(2)
c, (Å)	29.0763(7)	17.7481(7)	17.5944(3)
α, (°)	90	66.770(3)	107.8740(10)
β, (°)	90	82.299(7)	103.0260(10)
γ, (°)	90	78.713(6)	103.109(2)
V, (Å^3)	8796.0(3)	2559.0(3)	2582.92(8)
Z	8	3	2
ρ_{calc} g cm^{-3}	1.074	1.462	2.084
μ, mm^{-1}	1.695	18.134	9.050
Reflections measured	17669	24720	52795
Unique reflections, R_{int}	4571, 0.0501	8747, 0.0889	15546, 0.0309
Reflections with $F^2 > 2\sigma(F^2)$	4144	6407	11892
Transmission range	0.169-1.000	0.072-0.589	0.304-0.436
R, $R_w{}^a$ ($F^2 > 2\sigma(F^2)$)	0.0520, 0.1460	0.0941, 0.2572	0.0368, 0.0813
R, $R_w{}^a$ (all data)	0.0565, 0.1500	0.1171, 0.2763	0.0572, 0.0876
S^a	1.122	1.048	1.019
Parameters, Restraints	283, 260	482, 310	773, 1414
Max.,min. diff. map, e Å^{-3}	0.354, -0.203	3.553, -2.016	2.395, -1.338

^{a} Conventional $R = \Sigma||F_o| - |F_c||/\Sigma|F_o|$; $R_w = [\Sigma w(F_o^2 - F_c^2)^2/\Sigma w(F_o^2)^2]^{1/2}$; $S = [\Sigma w(F_o^2 - F_c^2)^2/\text{no. data} - \text{no. params})]^{1/2}$ for all data.

Supplementary Table 2. Crystallographic details for 4M (M = Rb, and Cs).

	**4aRb**	**4bRb**	**4Cs**
Formula	$C_{32}H_{44}Cl_6O_4RbTh_3$	$C_{28}H_{34}Cl_6O_2RbTh_3$	$C_{28}H_{34}Cl_6CsO_2Th_3$
Fw, g mol^{-1}	1486.96	1396.84	1444.28
Cryst size, mm	0.173 x 0.133 x 0.09	0.123 × 0.099 × 0.068	0.207 × 0.124 × 0.083
Crystal system	tetragonal	orthorhombic	orthorhombic
Space group	$P4_32_12$	*P*nma	*P*nma
Collection Temperature (K)	103(5)	150(2)	100(2)
a, (Å)	12.03630(10)	15.7200(4)	16.5109(7)
b, (Å)	12.03630(10)	19.2135(6)	19.3454(5)
c, (Å)	31.9139(2)	12.0843(3)	11.8857(5)
α, (°)	90	90	90
β, (°)	90	90	90
γ, (°)	90	90	90
V, (Å^3)	4623.45(8)	3649.90(17)	3796.4(2)
Z	4	4	4
ρ_{calc} g cm^{-3}	2.136	2.542	2.527
μ, mm^{-1}	35.503	44.866	13.113
Reflections measured	42780	13285	35687
Unique reflections, R_{int}	5018, 0.0790	3944, 0.0921	4300, 0.0981
Reflections with $F^2 > 2\sigma(F^2)$	4935	2970	3616
Transmission range	0.618-1.000	0.576-0.920	0.162-0.729
R, $R_w{}^a$ ($F^2 > 2\sigma$ (F^2))	0.0373, 0.1013	0.0602, 0.1427	0.0761, 0.1450
R, $R_w{}^a$ (all data)	0.0378, 0.1017	0.0867, 0.1529	0.0954, 0.1524
S^a	0.973	0.929	1.155
Parameters, Restraints	427, 1278	289, 932	289, 368
Max.,min. diff. map, e Å^{-3}	2.335, -2.454	2.576, -2.262	3.240, -1.672

[a] Conventional $R = \Sigma||F_o| - |F_c||/\Sigma|F_o|$; $R_w = [\Sigma w(F_o{}^2 - F_c{}^2)^2/\Sigma w(F_o{}^2)^2]^{1/2}$; $S = [\Sigma w(F_o{}^2 - F_c{}^2)^2/\text{no. data} - \text{no. params})]^{1/2}$ for all data.

Supplementary Table 3. Crystallographic details for 5M and 6.

	**5K**	**5Rb**	**6**
Formula	$C_{40}H_{64}Cl_6K_2O_8Th_3$	$C_{40}H_{64}Cl_6O_8Rb_2Th_3$	$C_{48}H_{84}Cl_6Na_2O_{13}Th_3$
Fw, g mol^{-1}	1659.93	1752.67	1823.95
Cryst size, mm	0.18 × 0.14 × 0.05	0.279 × 0.173 × 0.126	0.22 × 0.168 × 0.09
Crystal system	monoclinic	monoclinic	monoclinic
Space group	$P2_1/c$	$P2_1/n$	$P2_1/c$
Collection Temperature (K)	100(2)	100(2)	100(2)
a, (Å)	11.5069(3)	12.8433(3)	12.6537(2)
b, (Å)	36.7998(7)	16.6391(2)	31.7329(6)
c, (Å)	13.2334(2)	25.0223(5)	15.8580(3)
α, (°)	90	90	90
β, (°)	107.501(2)	98.300(2)	99.0331(16)
γ, (°)	90	90	90
V, (Å^3)	5344.3(2)	5291.28(18)	6288.62(19)
Z	4	4	4
ρ_{calc} g cm-^{3}	2.063	2.200	1.926
μ, mm^{-1}	8.826	32.288	25.573
Reflections measured	54053	78722	53975
Unique reflections, R_{int}	13728, 0.0319	9656, 0.1736	11944, 0.1406
Reflections with $F^2 > 2\sigma(F^2)$	12177	8847	8763
Transmission range	0.299-0.667	0.006-0.275	0.053-0.942
R, $R_w{}^a$ (F^2 > 2s(F^2))	0.0232, 0.0395	0.0978, 0.2682	0.0906, 0.2379
R, $R_w{}^a$ (all data)	0.0307, 0.0408	0.1023, 0.2735	0.1150, 0.2615
S^a	1.025	1.055	0.952
Parameters, Restraints	613, 456	509, 6	830, 2178
Max.,min. diff. map, e Å^{-3}	0.863, -0.652	5.461, -3.223	3.622, -3.094

^{a} Conventional $R = \Sigma||F_o| - |F_c||/\Sigma|F_o|$; $R_w = [\Sigma w(F_o^2 - F_c^2)^2/\Sigma w(F_o^2)^2]^{1/2}$; $S = [\Sigma w(F_o^2 - F_c^2)^2$/no. data – no. params)]$^{1/2}$ for all data.

Supplementary Table 4. Crystallographic details for 7.

	**7**
Formula	$C_{60}H_{96}Cl_6Cs_2N_4O_{13}Th_3$
Fw, g mol^{-1}	2256.04
Cryst size, mm	0.335 x 0.061 x 0.037
Crystal system	monoclinic
Space group	$P2_1/c$
Collection Temperature (K)	100(2)
a, (Å)	16.2827(5)
b, (Å)	21.3466(9)
c, (Å)	21.2147(5)
α, (°)	90
β, (°)	95.174(2)
γ, (°)	90
V, (Å^3)	7343.8(4)
Z	4
ρ_{calc} g cm-^{3}	2.041
μ, mm^{-1}	29.497
Reflections measured	58193
Unique reflections, R_{int}	12969, 0.1443
Reflections with $F^2 > 2\sigma(F^2)$	8622
Transmission range	0.308-1.000
R, $R_w{}^a$ ($F^2 > 2s(F^2)$)	0.1122, 0.2921
R, $R_w{}^a$ (all data)	0.1518, 0.3270
S^a	1.003
Parameters, Restraints	867, 758
Max.,min. diff. map, e Å^{-3}	6.387, -2.797

^{a} Conventional $R = \Sigma||F_o| - |F_c||/\Sigma|F_o|$; $R_w = [\Sigma w(F_o{}^2 - F_c{}^2)^2/\Sigma w(F_o{}^2)^2]^{1/2}$; $S = [\Sigma w(F_o{}^2 - F_c{}^2)^2/\text{no. data} - \text{no. params})]^{1/2}$ for all data.

Supplementary Table 5. Selected experimental parameters for Raman spectroscopic measurements of 4M (M = K, Rb, and Cs) and 5M (M = Na, K, Rb, and Cs).

Entry	4K	4aRb	4Cs	5Na	5K	5Rb	5Cs
λ (nm)	785	785	785	785	785	785	785
Power (mW)	1.5	1.5	1.5	1.5	1.5	1.5	1.5
Acquisition time (s)	10	10	5	10	20	5	10
Accumulations	10	10	5	10	5	10	10
Grating (gr/mm)	600	600	600	600	600	600	600
Filter (%)	25	10	25	25	10	25	25
Slit (μm)	100	100	100	100	100	100	100
Hole (μm)	300	300	300	300	300	300	300

Supplementary Table 6. Selected UV/Vis/NIR experimental details and data for 4M (M = K, Rb, and Cs) and 5M (M = Na, K, Rb, and Cs).

Entry	4K	4aRb	4Cs	5K	5Rb	5Na
Solvent	DME	DME	DME	DME	DME	DME
Molarity (mM)	0.108	0.334	0.164	0.475	0.121	0.294
ν_{max} (cm^{-1})	16026	16026	16018	17147	15995	16166
λ_{max} (nm)	624	624	624.3	583.2	625.2	618.6
ε_{max} (M^{-1} cm^{-1})	2336	3874	2515	85	1350	2133
$\Delta\nu_{1/2}$ (cm^{-1})	1877	2100	2556	3290	1988	2097
$\nu°_{1/2}$ (cm^{-1})	6133	6127	6093	6274	6127	6098
Γ[a]	0.69	0.66	0.58	0.48	0.68	0.66

Supplementary Table 7. Extracted g-parameters for 4K from the simulation of X-band frozen solution spectra recorded at 9.4 GHz and 6-100 K.

Temperature (K)	g_x	g_y	g_z
6	1.9802	1.978	1.943
10	1.9802	1.978	1.943
20	1.9802	1.978	1.943
50	1.9801	1.978	1.9428
80	1.98	1.977	1.9423
100	1.9799	1.9769	1.9418

Supplementary Table 8. Extracted g-parameters for 4K from the simulation of X-band powder spectra recorded at 9.4 GHz and 6-270 K.

Temperature (K)	g_x	g_y	g_z
6	1.9801	1.976	1.944
20	1.9801	1.9757	1.944
50	1.9801	1.975	1.9438
80	1.98	1.975	1.943
180	1.9777	1.974	1.9402
240	1.9755	1.9724	1.9375
270	1.9749	1.972	1.9372

Supplementary Table 9. Extracted g-parameters for 4aRb from the simulation of X-band frozen solution spectra recorded at 9.4 GHz and 6-100 K.

**Temperature (K)**	g_x	g_y	g_z
6	1.9795	1.9755	1.942
10	1.9795	1.9755	1.942
20	1.9795	1.9755	1.942
50	1.979	1.9755	1.9416
80	1.9785	1.9755	1.9413
100	1.978	1.9755	1.9406

Supplementary Table 10. Extracted g-parameters for 4aRb from the simulation of X-band powder spectra recorded at 9.4 GHz and 6-240 K.

**Temperature (K)**	g_x	g_y	g_z
6	1.9798	1.973	1.944
10	1.9798	1.973	1.944
80	1.9798	1.9755	1.942
180	1.9784	1.97	1.9378
240	1.976	1.97	1.9373

Supplementary Table 11. Extracted g-parameters for 4Cs from the simulation of X-band frozen solution spectra recorded at 9.4 GHz and 6-100 K.

**Temperature (K)**	g_x	g_y	g_z
6	1.9802	1.979	1.9432
10	1.9802	1.979	1.9432
20	1.9802	1.979	1.9432
50	1.9802	1.9790	1.9432
80	1.98	1.9778	1.9427
100	1.9796	1.9776	1.9422

Supplementary Table 12. Extracted g-parameters for 4Cs from the simulation of X-band powder spectra recorded at 9.4 GHz and 7-270 K.

**Temperature (K)**	g_x	g_y	g_z
7	1.9828	1.9704	1.9414
10	1.9828	1.9704	1.9414
20	1.9828	1.9704	1.9414
50	1.9821	1.9704	1.9404
80	1.983	1.9704	1.9398
120	1.9818	1.9684	1.9398
180	1.9804	1.968	1.9385
240	1.9788	1.9654	1.9368
270	1.9778	1.965	1.936

Supplementary Table 13. Sample information for those used in SQUID magnetometry measurements.

Entry	4K	4aRb	4Cs	5Na
Chemical formula	$C_{40}H_{64}Cl_6KO_8Th_3$	$C_{32}H_{44}Cl_6O_4RbTh_3$	$C_{28}H_{34}Cl_6CsO_2Th_3$	$C_{30}H_{30}Cl_6Na_2Th_3$
Molecular mass (g mol^{-1})	1620.86	1486.99	1444.30	1267.26
Sample mass (mg)	18.6	23.6	18.4	14.3
Eicosane mass (mg)	0	0	0	0

Supplementary Table 14. Sample information for those used in SQUID magnetometry measurements.

Entry	5K	5K-eico	5Rb	5Cs
Chemical formula	$C_{40}H_{64}Cl_6K_2O_8Th_3$	$C_{40}H_{64}Cl_6K_2O_8Th_3$	$C_{36}H_{54}Cl_6O_6Rb_2Th_3$	$C_{24}H_{24}Cl_6Cs_2Th_3$
Molecular mass (g mol^{-1})	1659.83	1659.83	1662.58	1487.09
Sample mass (mg)	22.7	21.9	18.8	14.6
Eicosane mass (mg)	0	7.6	0	0

Supplementary Table 15. Sample information for those used in SQUID magnetometry measurements.

Entry	Anthracene	Benzene	Biphenyl	Naphthalene
Chemical formula	$C_{14}H_{10}$	C_6H_6	$C_{12}H_{10}$	$C_{10}H_8$
Molecular mass (g mol^{-1})	178.23	78.11	154.21	128.1705
Sample mass (mg)	27.7	32.4	22.3	25.8
Eicosane mass (mg)	0	0	0	0

Supplementary Table 16. Selected data for the magnetic properties of 4M and 5M.

Entry	4K	4aRb	4Cs	5Na
χ_D (300 K, whole molecule)[a]	–1591.75E-06	–1132.43E-06	–797.22E-06	–1739.88E-06
R^2 value	0.9999	0.9999	0.9993	0.9998
χ_D (300 K, 1/3 molecule)	–530.58E-06	–377.48E-06	–265.74E-06	–579.96E-06
χ_D (300 K, per Th)	–319.72E-06	–206.95E-06	–112.48E-06	–437.00E-06
Th Pascal's constant	–23.00E-06	–23.00E-06	–23.00E-06	–23.00E-06
Factor increase vs expected	13.901	8.998	4.890	19.000
Expected χ_D (whole molecule)	–701.58E-06	–580.58E-06	–528.78E-06	–497.88E-06
χ_D per 1/3 molecule	–233.86E-06	–193.53E-06	–176.26E-06	–165.96E-06
Expected χ_D of non-Th atoms	–632.58E-06	–511.58E-06	–459.78E-06	–428.88E-06
χ_D per 1/3 of non-Th atoms	–210.86E-06	–170.53E-06	–153.26E-06	–142.96E-06
Superatom contribution (%)	55.92	48.73	33.67	71.38
χ_D Increase vs expected (%)	126.88	95.04	50.77	249.46
Λ (χ_D(expected) – χ_D(Found)	890.17E-06	551.85E-06	268.44E-06	1242.00E-06

[a] χ is given in units of cm^3 mol^{-1}.

Supplementary Table 17. Selected data for the magnetic properties of 5M.

Entry	5K	5K-eico	5Rb	5Cs
χ_D (300 K, whole molecule)[a]	–1141.53E-06	–1217.58E-06	–1505.39E-06	–1571.97E-06
R^2 value	0.9999	0.9999	0.9999	0.9996
χ_D (300 K, 1/3 molecule)	–380.51E-06	–405.86E-06	–501.80E-06	–523.99E-06
χ_D (300 K, per Th)	–164.68E-06	–190.33E-06	–302.34E-06	–380.50E-06
Th Pascal's constant	–23.00E-6	–23.00E-06	–23.00E-06	–23.00E-06
Factor increase vs expected	7.160	8.262	13.145	16.543
Expected χ_D (whole molecule)	–716.48E-06	–716.48E-06	–667.38E-06	–499.48E-06
χ_D per 1/3 molecule	–238.83E-06	–215.11E-06	–222.46E-06	–166.49E-06
Expected χ_D of non-Th atoms	–647.48E-06	–647.48E-06	–598.38E-06	–430.48E-06
χ_D per 1/3 of non-Th atoms	–215.83E.06	–215.83E-06	–199.46E-06	–143.49E-06
Superatom contribution (%)	37.24	41.15	55.67	68.23
χ_D Increase vs expected (%)	59.32	69.94	125.57	214.72
Λ (χ_D(expected) – χ_D(Found)	425.05E-06	501.10E-06	838.01E-06	1072.49E-06

[a] χ is given in units of cm^3 mol^{-1}.

Supplementary Table 18. Selected data for the magnetic properties of anthracene, benzene, biphenyl, and naphthalene.

Entry	Anthracene	Benzene	Biphenyl	Naphthalene
χ_D (300 K)[a]	–131.37E.06	–55.18E.06	–101.36E.06	–93.50E.06
R^2 value	0.9999	0.9999	0.9999	0.9999
Expected χ_D[b]	–130.30E.06	–54.80E.06	–103.30E.06	–91.90E.06
χ_D Increase vs expected (%)	0.82	0.69	–1.90	1.73

[a] χ is given in units of cm^3 mol^{-1}. [b] As reported.[15]

Supplementary Table 19. Comparison of the calculated modes [cm^{-1}] involving the Th$_3$ core in 5K', [4]$^{1-}$, and 5K.[1,13,14]

Mode	5K'	[4]$^{1-}$	5K
bending modes	<50	51.4/51.5	48.4, 51.0
bending modes	70.8, 71.0	67.3/67.5	73.6, 74.6
symmetric breathing mode	77.2	88.7	92.3
antisymmetric breathing mode	107.4	136.0	110.6, 115.5

Supplementary Table 20. Mulliken population analyses of the HOMO of 5K and selected acceptor NTOs with strong oscillator strengths, in terms of the 6d, 5f and 7p-character (%) of MOs and Th metal contributions.

	HOMO	NTO1	NTO2	NTO4	NTO7	NTO8	NTO9	NTO10	NTO16	NTO17
6d	68	26	25	8	19	25	16	13	10	11
5f	7	65	65	85	66	61	71	74	79	79
7p	13	0	0	0	2	2	0	0	0	0
7s	1	0	0	0	1	1	1	1	1	1
Th	89	91	90	93	88	89	88	88	90	91

Supplementary Table 21. The Mulliken populations of KS HOMO of 5K' and 5K, and SOMO of [4]$^{1-}$, given as percentages.[1,13,16]

	[**5**]K$_2$	[**4**]$^{1-}$	**5K**
Th 6d	64	62	68
Th 5f	7	14	7
Th 7p	20	7	13
Th 7s	1	3	1
Th total:	92	86	89

Supplementary Table 22. Ring-current strengths for 5K (nA/T).

**Parameter**	**5K**	**5K** without DMEs	**5K** without K's, DMEs	**$[Th_3]^{10+}$**	**$[Cl_6]^{6-}$**	**$[Th_3Cl_6]^{4+}$**	**$[COT_3]^{6-}$**
Diatropic	148.14	138.69	61.38	0.08	78.21	54.41	0.0228
Paratropic	–73.40	–61.93	–59.22	6.41	–77.21	–56.45	–0.0285
Total integral	74.74	76.76	2.16	-6.33	1.00	–2.04	–0.0057

Supplementary References

1. Seed, J. A. *et al.* Valence-Delocalized Trithorium Nanocluster Superatoms with Open-Shell Exalted Diamagnetism. *Nat. Chem.* **17**, 1035-1041 (2025).
2. Altman, A. B. *et al.* Chemical structure and bonding in a thorium(III)-aluminum heterobimetallic complex. *Chem. Sci.* **9**, 4317-4324 (2018).
3. Nguyen, J. Q. *et al.* Replacing trimethylsilyl with triisopropylsilyl provides crystalline $(C_5H_4SiR_3)_3$Th complexes of Th(III) and Th(II). *Organometallics* **42**, 2927-2937 (2023).
4. Parry, J. S., Cloke, G, Coles, S. J. & Hursthouse, M. B. Synthesis and Characterization of the First Sandwich Complex of Trivalent Thorium: A Structural Comparison with the Uranium Analogue. *J. Am. Chem. Soc.* **121**, 6867-6871 (1999).
5. Van Oven, H. O. & de Liefde Meijer, H. J.Cyclopentadienylcyclooctatetraenetitanium. *J. Organomet. Chem*. **19**, 373-376 (1969).
6. Kool, L. B, Rausch, M. D. & Rogers, R. D. The formation, crystal and molecular structure of (η^5-pentamethylcyclopentadienyl)(η^7-cycloheptatrienyl)titanium and (η^5-pentamethylcyclopentadienyl)(η^8-cyclooctatetraene)titanium. *J. Organomet. Chem*. **297**, 289-299 (1985).
7. Blenkers, J., Bruin, P. & Teuben J. H. Group IV Mixed Sandwich Compounds. *J. Organomet. Chem.* **297**, 61-67 (1985).
8. Horáček, M., Kupfer, V., Thewalt, U., Polášek, M. & Mach, K. Synthesis and structures of paramagnetic binuclear (η^8-1,4-bis(trimethylsilyl)cyclooctatetraenide)titanium(III) chlorides, *J. Organomet. Chem*. **579**, 126-132 (1999).
9. MacKenzie, R. E. *et al.* δ-Bonding modulates the electronic structure of formally divalent nd^1 rare earth arene complexes. *Chem. Sci.*, **15**, 15160-15169 (2024).
10. Tuschel, D. Selecting an Excitation Wavelength for Raman Spectroscopy. *Spectroscopy*. March (2016).
11. Lu, E. *et al*. Emergence of the Structure-Directing Role of f-Orbital Overlap-Driven Covalency *Nat. Commun.* **10**, 634 (2019).
12. Réant, B. L. L. *et al.* ^{29}Si NMR Spectroscopy as a Probe of s- and f-Block Metal(II)-Silanide Bond Covalency. *J. Am. Chem. Soc.* **143**, 9813-9824 (2021).
13. Tomeček, J., Liddle, S. T. & Kaltsoyannis, K. Actinide-Actinide Bonding: Electron Delocalisation and σ-Aromaticity in the Tri-Thorium Cluster $[\{Th(\eta^8\text{-}C_8H_8)(\mu\text{-}Cl)_2\}_3K_2]$. *ChemPhysChem*. **24**, e202300366 (2023).
14. Tomeček, J., Liddle, S. T. & Kaltsoyannis, K. Metal–Metal Bonding in Tri-Actinide Clusters: A DFT Study of $[An_3Cl_6]^z$ (z=1–6) and $[An_3Cl_6Cp_3]^z$ (z=−2–+3; An=Ac, Th, Pa, U, Np, Pu). *ChemPhysChem*. **26**, e202400816 (2025).
15. Dauben Jr, H. J., Wilson, J. D. & Laity, J. L. Diamagnetic Susceptibility Exaltation as a Criterion of Aromaticity. *J. Am. Chem. Soc*. **90**, 811-813 (1968).
16. Boronski, J. T. *et al.* A crystalline tri-thorium cluster with σ-aromatic metal-metal bonding. *Nature* **598**, 72-75 (2021).
