# Supplementary material for: Field-induced non-linear magnetic responses of all-metal Jellium σ-aromats
Source: Nat Commun. 2026 Jun 29;17:8117. doi: 10.1038/s41467-026-74403-3 (PMC13458622; doi:10.1038/s41467-026-74403-3)
Supplement: Supplementary file 2 [file 41467_2026_74403_MOESM2_ESM.pdf]

nature portfolio

Peer Review File

Field-Induced Non-Linear Magnetic Responses of All-Metal Jellium σ-Aromats

Corresponding Author: Professor Stephen Liddle

This file contains all reviewer reports in order by version, followed by all author rebuttals in order by version.

Attachments originally included by the reviewers as part of their assessment can be found at the end of this file.

Version 0:

Reviewer comments:

Reviewer #1

(Remarks to the Author)
please check the attached file.

Reviewer #2

(Remarks to the Author)
The paper by Liddle and Kaltsoyannis team explores the interesting case provided by a ligated Th3 organometallic structure. The magnetic characterization analysis provides an experimental view of the situation, which certainly is a more robust understanding in comparison to theoretical calculations based on dissecting the magnetic behavior in space. In my view, to define the concept of "Aromaticity" on the basis of theoretical results is not proper to define the concept, where magnetic experimental results are more supportive in clarifying the concept. In other words, it is imperative to define the concept from chemical observables first, which to my view, the magnetic response from NMR, EPR, and other magnetic tools, provides a strong basis to understand the phenomena. In this sense, another relevant issue is to generate a more unifying view, where again magnetic response gives the clue. The work is certainly of interest for the Nature Communications readership, and thus is suggested for publication after considering the following minor points:

#1
Since cyclic voltammetry is measured, it can be convenient to describe that the aromatic properties can be further tuned by controlling the overall charge state. in other words, we can have a switch on/off for aromaticity.

#2
An aromatic compound may lead to a harder possibility of removing or adding electrons? In other words, it may lead to shift the values for Eoxidation and Ereduction?

#3
Is it possible to account for the participation or non-participation of d- and f-shells for the Th3 bonding?

#4
Going back to early issues. The coordination of K+ atoms above/below the ring is nice. The authors may consider to plot the electrostatic potential over the van der Waals isosurface (rho isosurfe=0.001a.u.) to account for the negative region above/below the ring. It is exclusively from the Cl- atoms? or it came out from the overall structure?

Reviewer #3

(Remarks to the Author)
This manuscript reports a novel series of trithorium clusters, demonstrating both open- and closed-shell Jellium aromaticity through exalted diamagnetism. The work combines synthesis, spectroscopy, magnetometry, and theoretical analysis, offering important insights into actinide–actinide bonding and the concept of all-metal σ-aromaticity. The manuscript is well

organized and the findings are significant. However, several points should be addressed before publication.
1) What I am concerned about most is the excessive use of the comcepts of aromaticity and jellium. Based on the geometry and chemical bonding analysis, there just should be a 3c-2e covalent bond between the three Th atoms. Jellium model was proposed to describe the metallic bond of metal clusters, which is some what a “free electron gas” model. It should be pointed out that metallic bond of jellium model is , in concept, different from the metal-metal covalent bond in 5K.
2) Full chemical bonding analysis should be given in SI. Orbital composition analysis of the 3c-2e bond should also be given.

Version 1:

Reviewer comments:

Reviewer #1

(Remarks to the Author)
The authors have adequately addressed the concerns raised in the previous round of review. The revised manuscript corrects the spin-state assignment and related textual ambiguities, improves several notational and numerical issues, supplements additional electrochemical data, and clarifies experimental details to enhance reproducibility. The expanded theoretical discussion also strengthens the reliability of the conclusions. In particular, the authors justify the use of PBE0 for actinide systems by referring to prior benchmarks against CCSD(T), DLPNO-CCSD, and experimental NMR data. The omission of spin–orbit coupling is reasonably explained with supporting calculations showing minimal influence on the key conclusions. The DLPNO-CCSD T_1 diagnostic supports the single-reference character of the systems, while the GIMIC convergence tests demonstrate the numerical stability of the ring-current calculations. Additional analyses of orbital composition and electrostatic potential further improve the consistency between the computational and experimental results. Overall, the revised manuscript is clearer, more complete, and more convincing than the original version. I only suggest that the authors perform a final careful proofreading to remove remaining minor typographical or formatting errors, ensure that all newly added Supporting Information figures and tables are properly cited in the main text, and slightly temper statements where further experimental comparison remains beyond the present scope. These are minor issues and do not affect the main conclusions of the work. I therefore support acceptance of the manuscript for publication.

Reviewer #2

(Remarks to the Author)
The manuscript has been improved as suggested by the reviewer. Thus, I recommend publication in the current form.

Reviewer #3

(Remarks to the Author)
I have reviewed the revised paper carefully. All the revisions and comments have been properly addressed, and the quality of the manuscript has been significantly improved. I am now satisfied with the revised version and agree to accept it for publication as is.

Open Access This Peer Review File is licensed under a Creative Commons Attribution 4.0 International License, which permits use, sharing, adaptation, distribution and reproduction in any medium or format, as long as you give appropriate credit to the original author(s) and the source, provide a link to the Creative Commons license, and indicate if changes were made.
In cases where reviewers are anonymous, credit should be given to 'Anonymous Referee' and the source.
The images or other third party material in this Peer Review File are included in the article's Creative Commons license, unless indicated otherwise in a credit line to the material. If material is not included in the article's Creative Commons license and your intended use is not permitted by statutory regulation or exceeds the permitted use, you will need to obtain permission directly from the copyright holder.
To view a copy of this license, visit https://creativecommons.org/licenses/by/4.0/

Reviewer Comments

Reviewer #1

The manuscript presents a combined experimental and theoretical study on the 4M/5M series of heavy element clusters. The work is innovative, providing insights into electronic structure, spin states, magnetic response, and aromaticity. Overall, it represents a significant contribution to the field. The following points are intended as constructive discussion to enhance clarity, reproducibility, and robustness.

1. Experimental Aspects

1. Spin-state assignment: According to the EPR section, 4M species are described as S = 1/2, whereas 5M clusters correspond to two-electron closed-shell systems. The sentence "All of S = 0 4M and S = ½ 5M…" may therefore be reversed. Clarifying this point could help readers follow the discussion more accurately.

RESPONSE: We thank the reviewer for noticing the potential ambiguity. We appreciate that this could result in confusion, so have reversed the sentence to instead state "All of S = 1/2 **4M** and S = 0 **5M**…". and any similar sentences follow suit.

2. Language and notation: Minor editorial issues, such as "superatoms natures", "frequency analyses to", and the corrupted value "495.62.02 Å~ 10−6", could be clarified. Additionally, the UV-vis assignments list 17.5k cm−1 and 21k cm−1 both as 625 nm, which seems inconsistent with the wavenumbers. Addressing these would enhance readability and precision.

RESPONSE: Good spot, these transcription errors been corrected with thanks.

3. Electrochemical data: Only the 4K' species is presented. If feasible, discussing whether its redox behavior may generalize to other 4M/5M congeners, or including at least one additional representative dataset, could provide a more complete picture.

RESPONSE: We thank the reviewer for the suggestion. We are delighted to say that we have been able to collect good quality electrochemical data on **5K** in addition to **4K'**. We observe the $E_{PC(1)}$, $E_{PC(2)}$, $E_{PA(1)}$, and $E_{PA(2)}$ redox couples. Under the measurement conditions we did not observe the $E_{PC(3)}$ and $E_{PA(3)}$ couple for **5K**; if it is present it is obscured by the solvent-breakdown zone. The values are in good agreement to **4K'**, with variations (and indeed the non-observance of the $E_{PC/PA}$ couple) being attributable to the fact the K^+ ions in **5K** are coordinated by the cage so will modulate the redox events of the Th_3 core; indeed, this is why originally we examined **4K'** because there the K^+ are sequestered by 2.2.2-cryptand and so the electrochemistry of **4K'** is probing the Th_3 core in isolation. We have added text to that effect into the paper and added in salient plots to the SI.

4. NMR solvent in synthesis: In the SI section "Modified Synthesis of C8H8K2", the NMR solvent mixture is reported as C6D6/THF. It might be helpful to clarify whether THF was added only to aid solubility, whether it was deuterated, and how the mixture was prepared. This would improve clarity and reproducibility.

RESPONSE: Good point. We used a single drop of 1H-THF added to the NMR tube to aid solubility. These details were provided in the caption of Supplementary Figure 15, but we can see that this isn't clear enough so can be easily missed. As such, we have now included this

information within the experimental procedure of $C_8H_8K_2$, prior to the listing of ^{1}H NMR assignments.

5. Mass-to-mmol conversions: In the SI section "Modified Synthesis of MC8 (M = K, Rb, Cs)", the reported conversions (3.55 g graphite = 818.4 mmol; 1.45 g K = 102.3 mmol) may require confirmation. Ensuring the consistency of these values could enhance reproducibility.

RESPONSE: Good spot. The stoichiometry (~8:1) is correct, but regrettably the masses were incorrectly transcribed. This has now been corrected, and we have also added in the appropriate masses for Rb and Cs to aid reproducibility.

6. Silyl group notation: For compounds 2 and 3, the NMR assignments label the silyl groups as Si(CH3)4, whereas surrounding formulations indicate SiMe3. If possible, checking and aligning these labels would improve consistency.

RESPONSE: Fair point, this was a typographical error and it has now been corrected.

2. Theoretical Aspects

1. Considerations for Electronic Structure Methods and Relativistic Effects

Based on prior studies of actinide systems by Kaltsoyannis, Chem. Rev., 2000, 100, 2731–2765, and Pyykko, Annu. Rev. Phys. Chem., 2012, 63, 45–64, hybrid DFT methods such as PBE0 can provide reasonable results, but their performance for heavy elements is not comprehensively benchmarked. Scalar relativistic effects are often included through approaches like ZORA, yet spin orbit coupling (SOC) can significantly influence electronic and magnetic properties. Therefore, it may be helpful to clarify whether SOC was considered, and, if not, to investigate its impact on key results and possible spin contamination. Considering these points could help make the study more robust and comprehensive.

RESPONSE: We thank the reviewer for this comment. Despite the lack of universal benchmarks for actinide systems, hybrid functionals such as PBE0 have been extensively validated in relevant contexts. Our previous work has benchmarked PBE0 against CCSD(T) calculations (Nat. Commun. 10, 634 (2019)), as well as against experimental observables in actinide NMR studies (J. Am. Chem. Soc. 143, 9813–9824 (2021)), demonstrating its reliability for describing the electronic structure and magnetic properties of actinide compounds. Furthermore, we have shown the suitability of PBE0 for the 3c-2e trithorium cluster by comparison with DLPNO-CCSD results (ChemPhysChem 24, e202300366 (2023)).

Spin-orbit coupling (SOC) was not included in the present calculations. While SOC can play an important role in actinide systems, its inclusion is incompatible with the presented computational workflow (i.e. the electronic-structure analysis and magnetic-field-including methods). Moreover, our previous study (ChemPhysChem (2023)) demonstrated that scalar relativistic approaches provide a quantitatively similar description of Th–Th bonding within the QTAIM framework when compared to ZORA+SOC calculations (from ADF). We therefore expect that inclusion of SOC would not significantly affect the key conclusions of this work.

This is further supported by comparison of frontier molecular orbitals obtained from scalar ZORA and ZORA+SOC single-point calculations (ADF: PBE0/TZ2P), which show virtually identical spatial distributions (as shown in added Supplementary Figure S183).

The SI was modified to include the discussion on both PBE0 and SOC.

2. Using DLPNO-CCSD for Benchmarking DFT DLPNO-CCSD has been shown to provide high accuracy energies and serve as a reliable test for single reference wavefunctions, as discussed by Riplinger & Neese, J. Chem. Phys., 2013, 138, 034106. Previous work by Goerigk & Grimme, Phys. Chem. Chem. Phys., 2011, 13, 6670–6688, illustrates how DLPNOCCSD can be used to benchmark DFT predictions for relative energies and orbital properties. For actinide systems specifically, Liu et al., J. Chem. Theory Comput., 2017, 13, 1143–1156, provides examples of DLPNO-CCSD benchmarking on heavy element clusters. Accordingly, adding a small scale DLPNOCCSD benchmark could help confirm the reliability of DFT results, even on simplified models. Considering this could increase confidence in the reported DFT predictions.

RESPONSE: We thank the reviewer for this comment. In the present work, as stated in the SI DLPNO-CCSD calculations were performed to evaluate the T_1 diagnostic (0.009), confirming the single-reference character of the wavefunction and thereby supporting the use of DFT. However, DLPNO-CCSD density calculations were not performed due to the high computational cost of obtaining coupled-cluster densities for systems of this size. Importantly, in our previous work on a closely related 3c-2e trithorium cluster, we validated PBE0 against DLPNO-CCSD for a $[Th_3Cl_6]^{4+}$ model system (ChemPhysChem 24, e202300366 (2023)), yielding very good agreement in QTAIM Th–Th bonding results. Thus, our previous results provide strong support for the reliability of the PBE0 functional in the present study. This has been also added in the computational details in SI.

3. Reliability of Ring Current Analyses (GIMIC/AICD)
Ring current analyses such as GIMIC or AICD provide valuable insight into aromaticity, as shown by JusÅLelius et al., J. Chem. Phys., 2004, 121, 3952–3963, but results can be sensitive to integration parameters. Based on these experiences, it might be useful to verify convergence with respect to computational settings to ensure that observed trends in ring currents reflect the intrinsic electronic structure rather than numerical artifacts. Accounting for these considerations should make conclusions regarding aromaticity more convincing.

RESPONSE: We thank the reviewer for this comment. In the present work, ring current strengths were evaluated using standard integration parameters, which are defined to be sufficiently tight for GIMIC calculations. The integration plane was chosen to fully encompass and extend well beyond the system.

To verify numerical stability, we assessed the sensitivity of the results with respect to the integration grid by halving the grid spacing. This test yielded essentially identical ring current strengths (2.1577 vs 2.1580 nA/T for **5K** without $\{K(DME)_2\}^+$ moieties), confirming that the results are well converged with respect to the integration parameters. This has been also added in the computational details in the SI and also Supplementary Figure 185.

We therefore conclude that the observed computed ring current trends reflect the intrinsic electronic structure rather than numerical artefacts. We are very happy to have provided further clarifications, though we feel that it is important to not lose sight of a key aspect of our findings, which is that there has been controversy originating from calculations, but now through experiment we have unambiguously identified the source of that disagreement.

4. Analysis of Electronic Structure Under Magnetic Fields
The proposed mechanism involves field-induced electronic responses. Previous studies by Fowler & Steiner, Chem. Phys. Lett., 1986, 132, 159–164, and Sundholm et al., WIREs Comput. Mol. Sci., 2016, 6, 639–678, demonstrate that inclusion of external magnetic fields provides direct evidence for such responses. Following this logic, supplementing the calculations with selected magnetic fields and analyzing orbital reorganization could clarify whether the proposed electronic mechanism is consistent with the computed properties. Incorporating these calculations would likely strengthen the mechanistic arguments in the article.

RESPONSE: We thank the reviewer for the suggestion. Although explicitly including external magnetic fields could, in principle, provide additional insights into field-induced orbital reorganisation, such calculations would require SOC treatment and would be impossible for systems of this size, as we noted in our prior publication in this area (Nat. Chem. 17, 1035-1041 (2025)). The presented conclusions are based on established analysis methods, which provide a consistent and well-defined picture of the bonding and electron delocalisation. Also, while supplementing experimental results with theory is obviously desirable, we feel that the emphasis of the paper should remain on the unambiguous experimental findings.

Overall Assessment
This manuscript thoughtfully integrates experimental and computational approaches to explore heavy element clusters. It provides insights into spin states, electronic delocalization, aromaticity, and magnetic properties. Addressing the points above, particularly clarifying spin assignments, correcting editorial and numerical issues, and considering additional theoretical checks, would make the study even more robust. Overall, the work is innovative and represents a valuable contribution.

RESPONSE: We thank the reviewer for their considered thoughts and support.

Reviewer #2
The paper by Liddle and Kaltsoyannis team explores the interesting case provided by a ligated Th3 organometallic structure. The magnetic characterization analysis provides an experimental view of the situation, which certainly is a more robust understanding in comparison to theoretical calculations based on dissecting the magnetic behavior in space. In my view, to define the concept of "Aromaticity" on the basis of theoretical results is not proper to define the concept, where magnetic experimental results are more supportive in clarifying the concept. In other words, it is imperative to define the concept from chemical observables first, which to my view, the magnetic response from NMR, EPR, and other magnetic tools, provides a strong basis to understand the phenomena. In this sense, another relevant issue is to generate a more unifying view, where again magnetic response gives the clue. The work is certainly of interest for the Nature Communications readership, and thus is suggested for publication after considering the following minor points:

RESPONSE: We thank the reviewer for their considered thoughts and support.

#1
Since cyclic voltammetry is measured, it can be convenient to describe that the aromatic properties can be further tuned by controlling the overall charge state. in other words, we can have a switch on/off for aromaticity.

RESPONSE: The reviewer rightly raises an intriguing possibility, and is one we are looking into though it goes beyond the scope of this paper which is to reconcile the prior experimental-computational discord. As we described in response to R1 we recorded additional electrochemical data on **5K**, and while we aren't able to make exact like-for-like comparisons, since **4K'** has K^+ ions sequestered in 2.2.2-cryptand but in **5K** they are coordinated by the chlorides the data, accepting that there will be variations, the electrochemical data are self-consistent.

#2

An aromatic compound may lead to a harder possibility of removing or adding electrons? In other words, it may lead to shift the values for Eoxidation and Ereduction?

RESPONSE: The reviewer raises a very interesting point. As we explained earlier in response to R1 and in the point above we have added in the electrochemistry of **5K** and there are indeed small changes. However, we are not comparing like-with-like since **4K'** has the K^+ sequestered by 2.2.2-cryptand, whereas in **5K** they are on the cage chlorides, and also both types of cluster are aromatic. That said, given that the **4M** and **5M** series are both aromatic small variations are to be expected. We will need more experimental examples to delve into this matter more fully which is beyond the scope of the present contribution, but we absolutely agree this will be fascinating to examine in more detail in time when we have more compounds that are directly comparable to one another to examine.

#3

Is it possible to account for the participation or non-participation of d- and f-shells for the Th3 bonding?

RESPONSE: We thank the reviewer for this question. The participation of d- and f-orbitals in Th_3 bonding was analysed using MO Mulliken analyses and is reported in the Supporting Information (Table 21). This analysis indicates that the HOMO of **5K** bonding is dominated by 6d character (68%), with only minor contributions from 5f (7%) and 7p (13%) orbitals. This is in-line with what would be expected for the bonding of thorium, that is typically its bonding is dominated by 6d character with modest mixing in of f-character.

For clarity, a brief note summarising this Mulliken population analysis has also been added to the manuscript.

#4

Going back to early issues. The coordination of K+ atoms above/below the ring is nice. The authors may consider to plot the electrostatic potential over the van der Waals isosurface (rho isosurfe=0.001a.u.) to account for the negative region above/below the ring. It is exclusively from the Cl- atoms? or it came out from the overall structure?

RESPONSE: We thank the reviewer for the helpful suggestion. We have computed the electrostatic potential (ESP) mapped onto the van der Waals isosurface ($\rho = 0.001$ a.u.), which is now included in the Supporting Information (Supplementary Figure S184 with supporting explanation). The ESP plot was also mapped at the electron density isovalue of 0.01 a.u. to visualise the positive region around the K^+ ions.

The results show that the negative electrostatic potential in these regions arises from both the chloride ligands and the COT_2 framework.

Reviewer #3

This manuscript reports a novel series of trithorium clusters, demonstrating both open- and closed-shell Jellium aromaticity through exalted diamagnetism. The work combines synthesis, spectroscopy, magnetometry, and theoretical analysis, offering important insights into actinide–actinide bonding and the concept of all-metal σ-aromaticity. The manuscript is well organized and the findings are significant. However, several points should be addressed before publication.

RESPONSE: We thank the reviewer for their considered thoughts and support.

1) What I am concerned about most is the excessive use of the concepts of aromaticity and jellium. Based on the geometry and chemical bonding analysis, there just should be a 3c-2e covalent bond between the three Th atoms. Jellium model was proposed to describe the metallic bond of metal clusters, which is some what a "free electron gas" model. It should be pointed out that metallic bond of jellium model is, in concept, different from the metal-metal covalent bond in 5K.

RESPONSE: We thank the reviewer for highlighting an important point. The reviewer is correct that the ground state of the Th_3 clusters is three-centre bonding, and then the occupancy is one- or two-electron. The reviewer is also correct that the Jellium model pertains to a "free electron gas" model. These two concepts are indeed separate. However, as we described in the manuscript, the exalted diamagnetism, which is the manifestation of closed- or open-shell Jellium aromaticity, may be viewed as an excited state resulting from the application of an external field, which delocalises the electron density around the cluster, a "free electron gas", generating ring currents that have a cohesive wavefunction. In other words, the application of an external magnetic field transforms the localised three-centre bonding into a "free electron gas". Hence, a central message of the manuscript is that these clusters are field-induced superatoms. There is therefore no contradiction, indeed everything is internally consistent. However, clearly this aspect would benefit from further clarification, and therefore we have expanded our explanation in the manuscript to make this point clearer.

2) Full chemical bonding analysis should be given in SI. Orbital composition analysis of the 3c-2e bond should also be given.

RESPONSE: We thank the reviewer for this comment. The main findings from chemical bonding analysis are in the manuscript, and the detailed results, including orbital composition of the 3c-2e bond, are provided in the Supporting Information (Supplementary Figures 167–180/183 and Tables 19–22).

These results are consistent with our previous studies on related systems (Nature 598, 72-75 (2021); ChemPhysChem 24, e202300366 (2023); Nat. Chem. 17, 1035-1041 (2025)) and are therefore briefly summarised in the main text to maintain focus on the new findings of the present work. The Mulliken population analysis of **5K**'s HOMO has been explicitly added to the manuscript.

---End---

Reviewer Comments

Reviewer #1

The authors have adequately addressed the concerns raised in the previous round of review. The revised manuscript corrects the spin-state assignment and related textual ambiguities, improves several notational and numerical issues, supplements additional electrochemical data, and clarifies experimental details to enhance reproducibility. The expanded theoretical discussion also strengthens the reliability of the conclusions. In particular, the authors justify the use of PBE0 for actinide systems by referring to prior benchmarks against CCSD(T), DLPNO-CCSD, and experimental NMR data. The omission of spin–orbit coupling is reasonably explained with supporting calculations showing minimal influence on the key conclusions. The DLPNO-CCSD T_1 diagnostic supports the single-reference character of the systems, while the GIMIC convergence tests demonstrate the numerical stability of the ring-current calculations. Additional analyses of orbital composition and electrostatic potential further improve the consistency between the computational and experimental results.

Overall, the revised manuscript is clearer, more complete, and more convincing than the original version. I only suggest that the authors perform a final careful proofreading to remove remaining minor typographical or formatting errors, ensure that all newly added Supporting Information figures and tables are properly cited in the main text, and slightly temper statements where further experimental comparison remains beyond the present scope. These are minor issues and do not affect the main conclusions of the work. I therefore support acceptance of the manuscript for publication.

RESPONSE: We thank the reviewer for their time, and have checked the manuscript over in-line with their comments.

Reviewer #2

The manuscript has been improved as suggested by the reviewer. Thus, I recommend publication in the current form.

RESPONSE: We thank the reviewer for their time.

Reviewer #3

I have reviewed the revised paper carefully. All the revisions and comments have been properly addressed, and the quality of the manuscript has been significantly improved. I am now satisfied with the revised version and agree to accept it for publication as is.

RESPONSE: We thank the reviewer for their time.

---End---

Reviewer Comments

The manuscript presents a combined experimental and theoretical study on the 4M/5M series of heavy-element clusters. The work is innovative, providing insights into electronic structure, spin states, magnetic response, and aromaticity. Overall, it represents a significant contribution to the field. The following points are intended as constructive discussion to enhance clarity, reproducibility, and robustness.

1. Experimental Aspects

1. *Spin-state assignment:* According to the EPR section, 4M species are described as S = 1/2, whereas 5M clusters correspond to two-electron closed-shell systems. The sentence "All of S = 0 4M and S = 1/2 5M..." may therefore be reversed. Clarifying this point could help readers follow the discussion more accurately.

2. *Language and notation:* Minor editorial issues, such as "superatoms natures", "frequency analysesto", and the corrupted value "$495.62.02 \times 10^{-6}$", could be clarified. Additionally, the UV-vis assignments list 17.5k cm^{-1} and 21k cm^{-1} both as 625 nm, which seems inconsistent with the wavenumbers. Addressing these would enhance readability and precision.

3. *Electrochemical data:* Only the 4K' species is presented. If feasible, discussing whether its redox behavior may generalize to other 4M/5M congeners, or including at least one additional representative dataset, could provide a more complete picture.

4. *NMR solvent in synthesis:* In the SI section "Modified Synthesis of C8H8K2", the NMR solvent mixture is reported as C6D6/THF. It might be helpful to clarify whether THF was added only to aid solubility, whether it was deuterated, and how the mixture was prepared. This would improve clarity and reproducibility.

5. *Mass-to-mmol conversions:* In the SI section "Modified Synthesis of MC8 (M = K, Rb, Cs)", the reported conversions (3.55 g graphite = 818.4 mmol; 1.45 g K = 102.3 mmol) may require confirmation. Ensuring the consistency of these values could enhance reproducibility.

6. *Silyl group notation:* For compounds 2 and 3, the NMR assignments label the silyl groups as $Si(CH_3)_4$, whereas surrounding formulations indicate $SiMe_3$. If possible, checking and aligning these labels would improve consistency.

2. Theoretical Aspects

1. **Considerations for Electronic Structure Methods and Relativistic Effects**
   Based on prior studies of actinide systems by Kaltsoyannis, Chem. Rev., 2000, 100, 2731–2765, and Pyykkö, Annu. Rev. Phys. Chem., 2012, 63, 45–64, hybrid DFT methods such as PBE0 can provide reasonable results, but their performance for heavy elements is not comprehensively benchmarked. Scalar relativistic effects are often included through approaches like ZORA, yet spin orbit coupling (SOC) can significantly influence electronic and magnetic properties. Therefore, it may be helpful to clarify whether SOC was considered, and, if not, to investigate its impact on key results and possible spin contamination. Considering these points could help make the study more robust and comprehensive.

2. **Using DLPNO-CCSD for Benchmarking DFT**
   DLPNO-CCSD has been shown to provide high accuracy energies and serve as a reliable test for single reference wavefunctions, as discussed by Riplinger & Neese, J. Chem. Phys., 2013, 138, 034106. Previous work by Goerigk & Grimme, Phys. Chem. Chem. Phys., 2011, 13, 6670–6688, illustrates how DLPNO-CCSD can be used to benchmark DFT predictions for relative energies and orbital properties. For actinide systems specifically, Liu et al., J. Chem. Theory Comput., 2017, 13, 1143–1156, provides examples of DLPNO-CCSD benchmarking on heavy element clusters. Accordingly, adding a small scale DLPNO-CCSD benchmark could help confirm the reliability of DFT results, even on simplified models. Considering this could increase confidence in the reported DFT predictions.

3. **Reliability of Ring Current Analyses (GIMIC/AICD)**
   Ring current analyses such as GIMIC or AICD provide valuable insight into aromaticity, as shown by Jusélius et al., J. Chem. Phys., 2004, 121, 3952–3963, but results can be sensitive to integration parameters. Based on these experiences, it might be useful to verify convergence with respect to computational settings to ensure that observed trends in ring currents reflect the intrinsic electronic structure rather than numerical artifacts. Accounting for these considerations should make conclusions regarding aromaticity more convincing.

4. **Analysis of Electronic Structure Under Magnetic Fields**
   The proposed mechanism involves field-induced electronic responses. Previous studies by Fowler & Steiner, Chem. Phys. Lett., 1986, 132, 159–164, and Sundholm et al., WIREs Comput. Mol. Sci., 2016, 6, 639–678, demonstrate that inclusion of external magnetic fields provides direct evidence for such responses. Following this logic, supplementing the calculations with selected magnetic fields and analyzing orbital reorganization could clarify whether the proposed electronic mechanism is consistent with the computed properties. Incorporating these calculations would likely strengthen the mechanistic arguments in the article.

Overall Assessment

This manuscript thoughtfully integrates experimental and computational approaches to explore heavy-element clusters. It provides insights into spin states, electronic delocalization, aromaticity, and magnetic properties. Addressing the points above, particularly clarifying spin assignments, correcting editorial and numerical issues, and considering additional theoretical checks, would make the study even more robust.

Overall, the work is innovative and represents a valuable contribution to the field. After these clarifications and minor revisions, it would be suitable for publication.
